# Prevalence of chronic HCV infection in EU/EEA countries in 2019 using multiparameter evidence synthesis

**DOI:** 10.1016/j.lanepe.2023.100792

**Published:** 2023-12-13

**Authors:** Christos Thomadakis, Ilias Gountas, Erika Duffell, Konstantinos Gountas, Benjamin Bluemel, Thomas Seyler, Filippo Maria Pericoli, Irene Kászoni-Rückerl, Ziad El-Khatib, Martin Busch, Irene Schmutterer, Thomas Vanwolleghem, Sofieke Klamer, Els Plettinckx, Laure Mortgat, Dominique Van Beckhoven, Tonka Varleva, Mirjana Lana Kosanovic Licina, Tatjana Nemeth Blazic, Diana Nonković, Fanitsa Theophanous, Vratislav Nemecek, Marek Maly, Peer Brehm Christensen, Susan Cowan, Kristi Rüütel, Henrikki Brummer-Korvenkontio, Cécile Brouard, Gyde Steffen, Amrei Krings, Sandra Dudareva, Ruth Zimmermann, Georgia Nikolopoulou, Zsuzsanna Molnár, Emese Kozma, Magnús Gottfredsson, Niamh Murphy, Loreta A. Kondili, Maria Elena Tosti, Anna Rita Ciccaglione, Barbara Suligoi, Raina Nikiforova, Renate Putnina, Ligita Jancoriene, Carole Seguin-Devaux, Tanya Melillo, Anders Boyd, Marc van der Valk, Eline Op de Coul, Robert Whittaker, Hilde Kløvstad, Małgorzata Stępień, Magdalena Rosińska, Cristina Valente, Rui Tato Marinho, Odette Popovici, Mária Avdičová, Jana Kerlik, Irena Klavs, Mojca Maticic, Asuncion Diaz, Julia del Amo, Josefine Lundberg Ederth, Maria Axelsson, Georgios Nikolopoulos

**Affiliations:** a Medical School, University of Cyprus, Nicosia, Cyprus; b European Centre for Disease Prevention and Control, Stockholm, Sweden; c European Monitoring Centre for Drugs and Drug Addiction, Lisbon, Portugal; d VII/A/11 Communicable Diseases and Disease Control, Federal Ministry of Social Affairs, Health, Care and Consumer Protection, Vienna, Austria; e Institute for Surveillance & Infectious Disease Epidemiology, Austrian Agency for Health and Food Safety (AGES), Vienna, Austria; f Addiction Competence Center, Austrian National Public Health Institute, Vienna, Austria; g Viral Hepatitis Research Group, Laboratory of Experimental Medicine and Pediatrics, University of Antwerp, Antwerp, Belgium; h Department of Gastroenterology and Hepatology, University Hospital Antwerp, Antwerp, Belgium; i Department of Epidemiology and Public Health, Sciensano, Brussels, Belgium; j Scientific Research Institute, Medical University, Pleven, Bulgaria; k Communicable Disease Epidemiology Department, Andrija Stampar Teaching Institute of Public Health, Zagreb, Croatia; l Department for HIV, Sexual and Blood Transmitted Diseases, Reference Center of the Epidemiology of the Ministry of Health, Croatian Institute of Public Health, Zagreb, Croatia; m Teaching Institute of Public Health Split and Dalmatia County, Split, Croatia; n Department of Health Studies, University of Split, Split, Croatia; o Ministry of Health, Nicosia, Cyprus; p National Reference Laboratory for Viral Hepatitis, National Institute of Public Health, Prague, Czech Republic; q Department of Biostatistics, National Institute of Public Health, Prague, Czech Republic; r Department of Infectious Diseases, Odense University Hospital, Odense, Denmark; s Clinical Institute, University of Southern Denmark, Odense, Denmark; t Department of Infectious Disease Epidemiology and Prevention, Statens Serum Institut, Copenhagen, Denmark; u National Institute of Health Development, Tallinn, Estonia; v Finnish Institute for Health and Welfare, Helsinki, Finland; w Santé Publique France, The National Public Health Agency, Saint-Maurice, France; x Department of Infectious Disease Epidemiology, Robert Koch Institute, Berlin, Germany; y National Public Health Organization, Marousi, Greece; z National Center for Public Health and Pharmacy, Budapest, Hungary; aa Faculty of Medicine, School of Health Sciences, University of Iceland, Reykjavík, Iceland; ab Landspitali University Hospital, Reykjavík, Iceland; ac HSE Health Protection Surveillance Centre, Dublin, Ireland; ad National Center for Global Health, Istituto Superiore di Sanità, Rome, Italy; ae UniCamillus-Saint Camillus International University of Health and Medical Sciences, Rome, Italy; af Viral Hepatitis, Oncovirus and Retrovirus Disease Unit, Department of Infectious Diseases, Istituto Superiore di Sanità, Rome, Italy; ag National AIDS Unit, Department of Infectious Diseases, Istituto Superiore di Sanità, Rome, Italy; ah The Centre for Disease Prevention and Control, Riga, Latvia; ai Clinic of Infectious Diseases and Dermatovenerology, Institute of Clinical Medicine, Medical Faculty, Vilnius University, Vilnius, Lithuania; aj Department of Infection and Immunity, Luxembourg Institute of Health, Esch-sur-Alzette, Luxembourg; ak Infectious Disease Prevention and Control Unit, Health Promotion and Disease Prevention Directorate, Department of Health Regulation, Ministry for Health, Gwardamangia, Malta; al Department of Infectious Diseases, Public Health Service of Amsterdam, Amsterdam, the Netherlands; am stichting hiv monitoring, Amsterdam, the Netherlands; an Department of Internal Medicine, Division of Infectious Diseases, Amsterdam Institute for Infection and Immunity, Amsterdam University Medical Centers, University of Amsterdam, Amsterdam, the Netherlands; ao Centre for Infectious Disease Control, National Institute for Public Health and the Environment (RIVM), Bilthoven, the Netherlands; ap Section for Respiratory, Blood-borne and Sexually Transmitted Infections, Department of Infection Control and Vaccines, Norwegian Institute of Public Health, Oslo, Norway; aq Department of Infectious Disease Epidemiology and Surveillance, National Institute of Public Health NIH – National Research Institute, Warsaw, Poland; ar Department of Infectious Diseases, Hospitais da Universidade de Coimbra, Directorate General of Health, Coimbra, Portugal; as Centro Hospitalar Universitário Lisboa Norte, Medical School of Lisbon, Directorate General of Health, Ministry of Health, Lisbon, Portugal; at National Centre for Surveillance and Control of Communicable Diseases, National Institute of Public Health Romania, Bucharest, Romania; au Department of Epidemiology, Regional Authority of Public Health in Banská Bystrica, Banská Bystrica, Slovakia; av National Institute of Public Health, Ljubljana, Slovenia; aw Clinic for Infectious Diseases, University Medical Centre Ljubljana and Faculty of Medicine, University of Ljubljana, Ljubljana, Slovenia; ax National Centre of Epidemiology, Carlos III Health Institute, CIBER in Infectious Diseases (CIBERINFEC), Madrid, Spain; ay Division for HIV, STI, Viral Hepatitis and Tuberculosis Control, Ministry of Health, Madrid, Spain; az Public Health Agency of Sweden, Solna, Sweden

**Keywords:** HCV, Hepatitis C, Chronic hepatitis, Prevalence, Elimination, Europe

## Abstract

**Background:**

Epidemiological data are crucial to monitoring progress towards the 2030 Hepatitis C Virus (HCV) elimination targets. Our aim was to estimate the prevalence of chronic HCV infection (cHCV) in the European Union (EU)/European Economic Area (EEA) countries in 2019.

**Methods:**

Multi-parameter evidence synthesis (MPES) was used to produce national estimates of cHCV defined as: π = π_rec_ρ_rec_ + π_ex_ρ_ex_ + π_non_ρ_non_; π_rec_, π_ex_, and π_non_ represent cHCV prevalence among recent people who inject drugs (PWID), ex-PWID, and non-PWID, respectively, while ρ_rec_, ρ_ex_, and ρ_non_ represent the proportions of these groups in the population. Information sources included the European Centre for Disease Prevention and Control (ECDC) national operational contact points (NCPs) and prevalence database, the European Monitoring Centre for Drugs and Drug Addiction databases, and the published literature.

**Findings:**

The cHCV prevalence in 29 of 30 EU/EEA countries in 2019 was 0.50% [95% Credible Interval (CrI): 0.46%, 0.55%]. The highest cHCV prevalence was observed in the eastern EU/EEA (0.88%; 95% CrI: 0.81%, 0.94%). At least 35.76% (95% CrI: 33.07%, 38.60%) of the overall cHCV prevalence in EU/EEA countries was associated with injecting drugs.

**Interpretation:**

Using MPES and collaborating with ECDC NCPs, we estimated the prevalence of cHCV in the EU/EEA to be low. Some areas experience higher cHCV prevalence while a third of prevalent cHCV infections was attributed to PWID. Further efforts are needed to scale up prevention measures and the diagnosis and treatment of infected individuals, especially in the east of the EU/EEA and among PWID.

**Funding:**

10.13039/501100000805ECDC.

**Research in context**

**Evidence before this study**

In view of the public health need to meet the World Health Organization (WHO) elimination targets for hepatitis C virus (HCV), it is essential to accurately monitor the epidemiological trends of the prevalence of chronic HCV (cHCV) infection. HCV disproportionately affects certain population groups, such as people who inject drugs (PWID), which poses a special challenge to HCV epidemiological studies. In the European Union (EU)/European Economic Area (EEA), many studies among the general population have been conducted and included in a database developed by the European Centre for Disease Prevention and Control (ECDC). Other studies/reports on PWID have also been published and included in the European Monitoring Centre for Drugs and Drug Addiction (EMCDDA) databases. However, these estimates alone cannot be combined to obtain a national estimate of people with HCV unless additional information regarding the proportion of each specific group in the overall population is known. Moreover, many of these studies are based on data on HCV antibodies or are quite old. Thus, the effect of direct-acting antivirals (DAAs) is ignored although they comprise a key tool to control HCV due to the high sustained virological response (SVR) they incur. The Polaris Observatory HCV Collaborators have provided regional and global estimates of HCV prevalence based on a simulation model, which, however, requires probably many additional country-specific parameters. Furthermore, the contribution of injection drug use (IDU) was not estimated. An alternative approach, which has been applied in the field of HCV in the past, is the Multi-Parameter Evidence Synthesis (MPES). MPES is a Bayesian approach that simultaneously combines direct and/or indirect information on different parameters or functions of parameters from diverse sources to derive an overall estimate of a parameter of interest, for example, an estimate of the nationwide prevalence of an infection, along with boundaries of uncertainty.

**Added value of this study**

This study estimated the prevalence of cHCV (the viremic population, i.e., those positive to HCV-RNA or the HCV core antigen) and the contribution of IDU to that estimate for countries in the EU/EEA in 2019. It is the first time that MPES is used at the European level through a harmonized approach across the region involving local experts. We included studies published in previous ECDC and EMCDDA reports or other relevant information suggested by the ECDC national operational contact points and other national experts. The population of each country (15–79 years old) was divided into three main non-overlapping risk groups: recent PWID (those who injected in the last year); ex-PWID (those who last injected more than a year ago); and non-PWID. The overall cHCV prevalence in the population was estimated as a weighted sum of the respective cHCV probabilities. A multi-state Markov model was used to estimate the size of recent and ex-PWID. Adjustment for treatment with DAAs was made where possible. A database that summarizes the results and includes the country-specific reports was created.

**Implications of all the available evidence**

The estimated overall cHCV prevalence among people 15–79 years of age, as of 2019, in 29 out of 30 EU/EEA countries (apart from Lichtenstein) was 0.50%, being higher in Eastern European countries (0.88%) and lower in Western European countries (0.27%). This prevalence estimate corresponded to approximately 1,800,000 viremic infections, with more than a third of them attributed to IDU. This study suggests that, overall, the burden of cHCV, as a percentage, is low in the EU/EEA though it remains considerable in some of its countries and among PWID. Moreover, the total (absolute) number of cHCV cases remains large across the region. Therefore, EU/EEA countries need to continue their efforts to eliminate HCV by scaling up testing alongside harm reduction measures and timely linkage to treatment. Our estimates presented in this analysis could serve as a basis for EU/EEA countries to review and perhaps adapt their local HCV strategies.

## Introduction

Hepatitis C virus (HCV) infection is a major public health problem affecting 0.7% of the world population with 1.4 million new infections and more than 250,000 deaths due to long-term complications of chronic hepatitis C (CHC) in 2020.^1^ In the absence of an effective hepatitis C vaccine,^2^ prevention of new HCV infections, especially through harm reduction measures targeting people who inject drugs (PWID), screening, and effective treatment using direct-acting antivirals (DAAs), remain key measures to reduce HCV incidence and associated morbidity and mortality. DAAs, in particular, have significantly improved treatment outcomes with more than 95% of HCV-infected people achieving sustained virological response (SVR).^3^

Driven by the large CHC burden and the advancements in therapeutics, the World Health Organization (WHO) developed the Global Health Sector Strategy on viral hepatitis targeting elimination of hepatitis as a public health threat by 2030. The ambitious targets include a 65% reduction in deaths related to hepatitis B and C, a 90% reduction in new cases of chronic hepatitis B virus and HCV infection (cHCV), and the diagnosis of 90% of people with hepatitis B and C by 2030.^4^^,^^5^ Absolute targets in the updated 2022–2030 strategy include an HCV incidence of less than 5/100,000 in the general population and less than 2/100 in PWID, and annual HCV-related mortality less than 2/100,000 persons.^6^

An essential step towards HCV elimination is having a clear understanding of the local epidemiology to effectively plan public health strategies and address health-service needs. The varying prevalence of HCV infection in different population groups, however, poses challenges to HCV epidemiology.^7^ Data among PWID in the European Union (EU)/European Economic Area (EEA) countries indicate huge differences in seroprevalence (i.e., prevalence of antibodies to HCV) depending on country and study settings (www.emcdda.europa.eu/data/stats2021/pdu_en; last time accessed October 8th, 2023).^8^ In some countries, HCV seroprevalence is very high (around 80%) among PWID.^8^ HCV prevalence is also disproportionally higher among other populations such as people with migratory background from high endemicity countries^9^ and men who have sex with men (MSM),^8^^,^^10^ compared to the general population.^8^^,^^11^, ^12^, ^13^ Data from general population studies in Europe also reveal variability by country and year of study corresponding to historical differences in the prevalence of risk factors, as well as more recently to the impact of treatment https://www.ecdc.europa.eu/en/infectious-disease-topics/z-disease-list/hepatitis-c/tools/hepatitis-c-prevalence-database; last time accessed October 8th, 2023).^8^^,^^12^ Moreover, HCV elimination has been proceeding at different speed throughout Europe, with some countries on track to achieve the elimination goals set by WHO whilst others still struggle with limited political support and insufficient resources to achieve elimination.^14^ In many EU/EEA countries, the lack of knowledge of the national burden of HCV infection has been hampering the elimination process and should be overcome.

Research groups in many countries have conducted epidemiological studies that measure HCV prevalence in the general population and specific population groups, yet none of these estimates provides a full measure of national prevalence and they need to be combined with additional information regarding the size of each specific group in the population to generate a national estimate of the HCV burden.^15^^,^^16^ Multi-parameter evidence synthesis (MPES) is an approach that simultaneously combines direct and/or indirect information on different parameters or functions of parameters from diverse sources in order to derive an overall estimate of a parameter of interest, for example, an estimate of the nationwide prevalence of an infection.^17^ MPES has been used in studying the epidemiology of chronic infections such as HIV^18^ and HCV.^15^ For example, Sweeting et al. described the method and applied it to calculate the prevalence of serological evidence of HCV infection in England and Wales.^15^

A further challenge to understanding the burden of HCV infection is that whilst there exist published reports of HCV estimates in some EU/EEA countries,^7^ many of these studies only measure the prevalence of antibodies to HCV, not accounting for the treatment of cHCV. This is not sufficient considering both the need of predicting the future burden of disease and the impact of ongoing infections on onward transmission. The available data are often fragmented and potentially incomplete, as, for example, the existing data on key population sizes or the data on viremic infection prevalence. What’s more, the EU/EEA lacks a harmonised and simple approach that involves local experts, makes use of all available information, and would also provide more comparable national estimates of cHCV prevalence needed for monitoring the epidemic and the progress towards the elimination goals. Therefore, the aim of our study was to estimate the prevalence of cHCV infection (viremic population, i.e., positive for HCV-RNA or the HCV core antigen) and the contribution of injection drug use (IDU)—the major route of HCV transmission—to that estimate for countries in the EU/EEA using a systematic and harmonised approach.

## Methods

A simple version of MPES that accounts for uncertainty within a Bayesian framework was applied to each EU/EEA country separately to derive national estimates for 2019. The time point of 2019 was selected for three reasons: i) to produce estimates before services disruptions or other impacts due to the COVID-19 pandemic; ii) because most prevalence estimates available, including those for PWID, were before 2019; and iii) sufficient time had elapsed since the introduction of DAAs in 2014–2015 to account for their widespread rollout and impact. The initial estimates for each country were discussed with the European Centre for Disease Prevention and Control (ECDC) national operational contact points (NCPs) and other national experts, and then refined or improved based on their feedback. Countries were grouped into regions using the definition of the Statistics Division of the United Nations (https://unstats.un.org/unsd/methodology/m49/; last time accessed October 8th, 2023) (Table 1 and Appendix A).

**Table 1:** Estimates of the hepatitis C virus (HCV) chronically infected population (% prevalence and absolute numbers) and the proportion (%) of chronic hepatitis C virus infection (cHCV) attributable to injection drug use in European Union—European Economic Area (EU/EEA) countries.

Country	Median prevalence (%) of cHCV (95% credible interval)	Population with cHCV (95% credible interval)	Least proportion (%) attributable to injection drug use (95% credible interval)
**EU/EEA**	0.50 (0.46, 0.55)	1,782,923 (1,638,132, 1,941,583)	35.76 (33.07, 38.60)
**Eastern Europe**	0.88 (0.81, 0.94)	634,273 (587,854, 684,696)	40.34 (37.14, 43.58)
Bulgaria	1.11 (0.83, 1.60)	62,610 (47,032, 90,766)	62.56 (43.14, 82.76)
Czechia	0.78 (0.55, 1.11)	66,794 (46,853, 94,196)	24.60 (17.22, 35.10)
Hungary	0.23 (0.20, 0.25)	17,984 (15,962, 20,101)	48.31 (42.51, 53.36)
Poland	0.36 (0.27, 0.45)	108,210 (82,261, 137,566)	4.27 (3.16, 5.83)
Romania	2.26 (2.11, 2.41)	348,939 (326,554, 372,034)	46.81 (43.61, 49.97)
Slovakia	0.62 (0.47, 0.78)	27,407 (20,658, 34,501)	87.12 (74.69, 95.18)
**Northern Europe**	0.41 (0.37, 0.45)	120,493 (109,815, 134,393)	70.03 (65.03, 73.98)
Denmark	0.27 (0.25, 0.30)	12,423 (11,262, 13,621)	97.43 (96.05, 98.45)
Estonia	1.71 (1.49, 2.06)	17,634 (15,413, 21,306)	85.48 (71.19, 95.16)
Finland	0.59 (0.53, 0.66)	25,650 (22,801, 28,477)	95.91 (94.66, 96.94)
Iceland	0.10 (0.05, 0.20)	279 (151, 547)	83 (41.63, 100)
Ireland	0.21 (0.13, 0.35)	7844 (4,711, 13,035)	62.31 (37.01, 99.78)
Latvia	0.77 (0.68, 0.87)	11,640 (10,236, 13,090)	81.26 (75.43, 87.61)
Lithuania	1.01 (0.94, 1.09)	22,410 (20,761, 24,139)	25.05 (22.60, 27.68)
Norway	0.22 (0.14, 0.30)	9164 (5,954, 12,631)	76.81 (66.64, 85.48)
Sweden	0.16 (0.07, 0.31)	12,758 (5,174, 24,732)	41.89 (33.35, 53.17)
**Southern Europe**	0.59 (0.50, 0.69)	633,109 (540,571, 737,905)	19.38 (16.66, 22.67)
Croatia	0.74 (0.46, 1.11)	24,274 (15,060, 36,404)	9.31 (4.92, 17.57)
Cyprus	0.19 (0.15, 0.25)	1353 (1,035, 1756)	59.79 (46.03, 73.76)
Greece	0.55 (0.36, 0.80)	46,260 (30,310, 67,042)	11.49 (7.75, 17.72)
Italy	0.96 (0.80, 1.15)	459,000 (379,172, 549,698)	16.68 (13.87, 20.14)
Malta	0.27 (0.20, 0.35)	1083 (812, 1398)	87.56 (76.85, 94.78)
Portugal	0.50 (0.37, 0.78)	41,161 (30,370, 64,216)	67.35 (43.13, 90.12)
Slovenia	0.07 (0.02, 0.14)	1078 (317, 2319)	83.94 (35.54, 99.35)
Spain	0.15 (0.06, 0.27)	54,676 (21,352, 101,774)	15.13 (7.94, 38.84)
**Western Europe**	0.27 (0.20, 0.34)	391,737 (297,454, 504,226)	44.63 (36.45, 53.68)
Austria	0.33 (0.29, 0.38)	23,860 (20,931, 26,854)	93.95 (91.72, 95.72)
Belgium	0.18 (0.09, 0.36)	16,178 (7,737, 31,562)	20.17 (9.76, 42.62)
France	0.29 (0.16, 0.46)	142,921 (77,226, 227,201)	34.28 (20.84, 51.32)
Germany	0.30 (0.21, 0.42)	196,671 (137,554, 279,639)	49.29 (37.66, 62.99)
Luxembourg	0.25 (0.15, 0.39)	1243 (760, 1894)	73.09 (54.36, 88.46)
Netherlands	0.04 (0, 0.16)	6183 (0, 21,759)	26.06 (10.01, 73.86)

**Note:** HCV-RNA prevalence is used as a proxy for chronic HCV infection (cHCV); Population: 15–79 years old; Sub-regions names denote the EU/EEA countries within the United Nations sub-regions; Cyprus was grouped into Southern Europe for this analysis.

## Description of the MPES method

The population of each country (15–79 years old) was divided into three main non-overlapping risk groups:

1. Recent PWID (those who injected in the last year);
2. Ex-PWID (those who last injected more than a year ago);
3. Non-PWID.

The overall prevalence of cHCV in the population, π, using the above approach, was defined as the weighted sum of three probabilities (Equation (1)):

(1) $$\begin{array}{r} \pi =\pi_{rec}\rho_{rec}+\pi_{ex}\rho_{ex}+\pi_{non}\rho_{non} \end{array}$$

The parameters $\pi_{rec}$, $\pi_{ex}$, and $\pi_{non}$ represent cHCV prevalence among recent, ex-PWID, and non-PWID, respectively, while the parameters $\rho_{rec}$, $\rho_{ex}$, and $\rho_{non}$ represent the proportion of recent, ex-PWID, and non-PWID in the overall population ($\rho_{rec}+\rho_{ex}+\rho_{non}=1)$. Several different studies were used to estimate the above-mentioned parameters in each country, and evidence about cHCV prevalence was combined via a unified MPES model fitted under a Bayesian approach using Hamiltonian Monte Carlo through the STAN software. Vague, uniform from 0 to 1, prior distributions were specified for cHCV prevalence. Since most data for cHCV were available in the form of numerator and denominator, independent Binomial distributions were used.

### Proportion of recent PWID, ex-PWID, and non-PWID in the overall population ($\rho_{rec}$, $\rho_{ex},\rho_{non}$)

To estimate the number of recent and ex-PWID in 2019, a stochastic, multi-state Markov model was used, representing the non-PWID, recent PWID, and ex-PWID groups. The structure of the model, which is a modification of a method proposed by McDonald et al.,^16^ is described in detail in Appendix A. In brief, the model tracks the number of recent PWID, ex-PWID, and ever-PWID (both recent and ex-PWID), every year, accounting for ageing and the PWID-related excess mortality. The model was calibrated to match the number of recent and/or ever PWID reported either in the review of Grebely et al.^19^ or in the European Monitoring Centre for Drugs and Drug Addiction (EMCDDA) statistical bulletin (www.emcdda.europa.eu/data/stats2021/pdu_en; last time accessed October 8th, 2023) and/or Barometer (www.emcdda.europa.eu/publications/html/viral-hepatitis-elimination-barometer_en; last time accessed October 8th, 2023), preferring studies using capture-recapture sampling, or estimates provided by the NCPs and other national experts.

Through the model, the numbers of recent and ex-PWID in the population of each country in 2019 were estimated. The size of non-PWID was estimated by subtracting the number of recent and ex-PWID from the total size of the population of each country (15–79 years old) derived from Eurostat. Finally, $\rho_{rec}$, $\rho_{ex},\text{and}\;\rho_{non}$ were estimated by dividing the above-mentioned numbers by the total population (15–79 years old) of each country.

### cHCV prevalence ($\pi_{rec},\pi_{ex},\pi_{non}$)

#### Spontaneous viral clearance

When available reported data referred to the prevalence of antibodies to HCV and no HCV-RNA prevalence information was available, the spontaneous viral clearance probability of 0.26 [95% Confidence Interval (CI): 0.22, 0.29] was applied to estimate the prevalence of cHCV in each group.^20^ Uncertainty in this estimate was also considered through a Normal distribution, with its mean and standard deviation informed by the results published in the study of Micallef et al.^20^ More information can be found in Appendix A.

#### Prevalence of cHCV among recent PWID ($\pi_{rec})$

The prevalence of cHCV in recent PWID was informed by studies provided by the ECDC NCPs. If there was no input by NCPs, we used information from studies included in the EMCDDA statistical bulletin/Barometer (www.emcdda.europa.eu/data/stats2021/pdu_en; www.emcdda.europa.eu/publications/html/viral-hepatitis-elimination-barometer_en; last time accessed October 8th, 2023). These data were derived from, for instance, cross-sectional surveys, respondent-driven surveys, or testing of PWID at drug treatment facilities. Finally, if there were no studies in the EMCDDA platforms, we used the review of Grebely et al.^19^

#### Prevalence of cHCV among ex-PWID ($\pi_{ex}$)

Direct information on cHCV prevalence among ex-PWID was scarce and often unavailable in the literature. To overcome this issue, data on cHCV prevalence among ever PWID (i.e., indirect information on both recent and ex-PWID) obtained through the EMCDDA databases or other sources suggested by the NCPs were used.^18^ Data were obtained from, for instance, cross-sectional surveys, samples from individuals in opioid agonist therapy, or drug treatment centers. However, cHCV prevalence among ever PWID provides information on a mixture of the parameters of interest, i.e., $\pi_{rec}$ and $\pi_{ex}$. Unless additional information was provided, we used the estimated proportions of the risk groups in the population produced by the Markov model as the mixture proportions (i.e., $\frac{\rho_{rec}}{\rho_{rec}+\rho_{ex}}$ and $\frac{\rho_{ex}}{\rho_{rec}+\rho_{ex}}$, respectively). Under the above assumptions, $\pi_{ex}$ could be indirectly computed by applying the formula below (Equation (2)):

(2) $$\begin{array}{r} \pi_{ex}=\left(\pi_{ever}-\frac{\rho_{rec}}{\rho_{rec}+\rho_{ex}}\pi_{rec}\right)\times \frac{\rho_{rec}+\rho_{ex}}{\rho_{ex}} \end{array}$$

However, when data on the cHCV prevalence among ex-PWID were available (e.g., Spain), we estimated $\pi_{ex}$ directly. Moreover, when the mixture proportion of recent PWID among ever PWID was known or reliably estimated (e.g., France), we took this into account (information can also be found in Appendix A).

#### Prevalence of cHCV among non-PWID ($\pi_{non}$)

Estimates of cHCV prevalence in the non-PWID population were retrieved from published and unpublished national data, available from the ECDC hepatitis C prevalence database (https://www.ecdc.europa.eu/en/infectious-disease-topics/z-disease-list/hepatitis-c/tools/hepatitis-c-prevalence-database; last time accessed October 8th, 2023) and/or informed by NCPs, covering data published between 2005 and June 2021.^12^ Studies in the ECDC database among the general population and pregnant women had been assessed for quality using several factors and a risk of bias score had been assigned. Specifically, for general population studies, the domains age, gender, sampling method and response rate, and geographical coverage were considered to quantify the risk of bias. Pre-determined points were awarded in each domain. A total quality score was calculated by summing the values in each domain, with the bias score ranging between 0 and 6 and higher scores representing higher-quality studies.

When higher-quality studies, according to the above-mentioned score, among the general population were available for a certain country (score≥4), their estimates of cHCV prevalence among non-PWID were used. If there was no high-quality evidence, estimates from general population-based studies with a lower quality score (score < 4) were used. If no general population-based prevalence estimates were available, studies on pregnant women were used to obtain proxy HCV prevalence figures. Finally, if studies on pregnant women were also unavailable, first-time blood donor data were used. To avoid overlapping of PWID and non-PWID groups and thus to estimate the prevalence of cHCV more accurately in the non-drug-using population, the methods and results of general population-based studies or their proxies were carefully reviewed to remove data referring to PWID where possible. Thus, to the best of our knowledge, cHCV data derived from the general population studies refer to non-PWID, avoiding overlapping with PWID as much as possible. When multiple studies from the general population were included, we also conducted random-effect analyses to account for potential within-country heterogeneity. The results of the random-effect analyses are presented in the supplementary material (Appendix B).

### Adjustment for treatment with DAAs

Treatment adjustment was made if cHCV prevalence data were available before 2019. The total number of individuals treated with DAAs, if available, was provided by the NCPs. For countries where the distribution of the risk groups among the treated population was not available, it was assumed that the distribution of the risk groups among those treated was that of their corresponding distribution among the cHCV population, i.e., Pr(RecentPWID|cHCV), Pr(Ex-PWID|cHCV), and Pr(Non-PWID|cHCV), as estimated by the MPES model using the Bayes rule when information on DAAs was ignored. When DAAs data were available for some of the three groups, the corresponding number of individuals cured by DAAs after the year of the study was stochastically subtracted from the respective cHCV population. To do so, the SVR rates and their uncertainty were also taken into consideration through a prior distribution based on well-known published studies.^21^^,^^22^

### Sensitivity analysis—people with migratory background

Additional sensitivity analyses including people with migratory background from high endemicity countries, as a separate group, were performed based on data from a technical report from ECDC.^23^ The results of these analyses are included in country-specific reports (Appendix B) and should be interpreted with caution due to potential overlapping between groups.

### Database and detailed report for each country

More detailed information about the methodology is available in the Appendix A. Country-specific reports providing in-depth insights into the methodology and data sources employed for each country are available in Appendix B. To further facilitate access to the results, a website has also been developed. An interactive EU/EEA map that summarizes the results and all country-specific reports are also available at http://hcveurope.eu/.

The study follows the Guidelines for Accurate and Transparent Health Estimates Reporting (GATHER statement–https://www.who.int/data/gather; last time accessed October 8th, 2023).^24^

### Role of the funding source

The funding source was not involved in study design; in the collection, analysis, and interpretation of data; in the writing of the report; and in the decision to submit the paper for publication.

## Results

### cHCV prevalence (%)

**Fig. 1:**
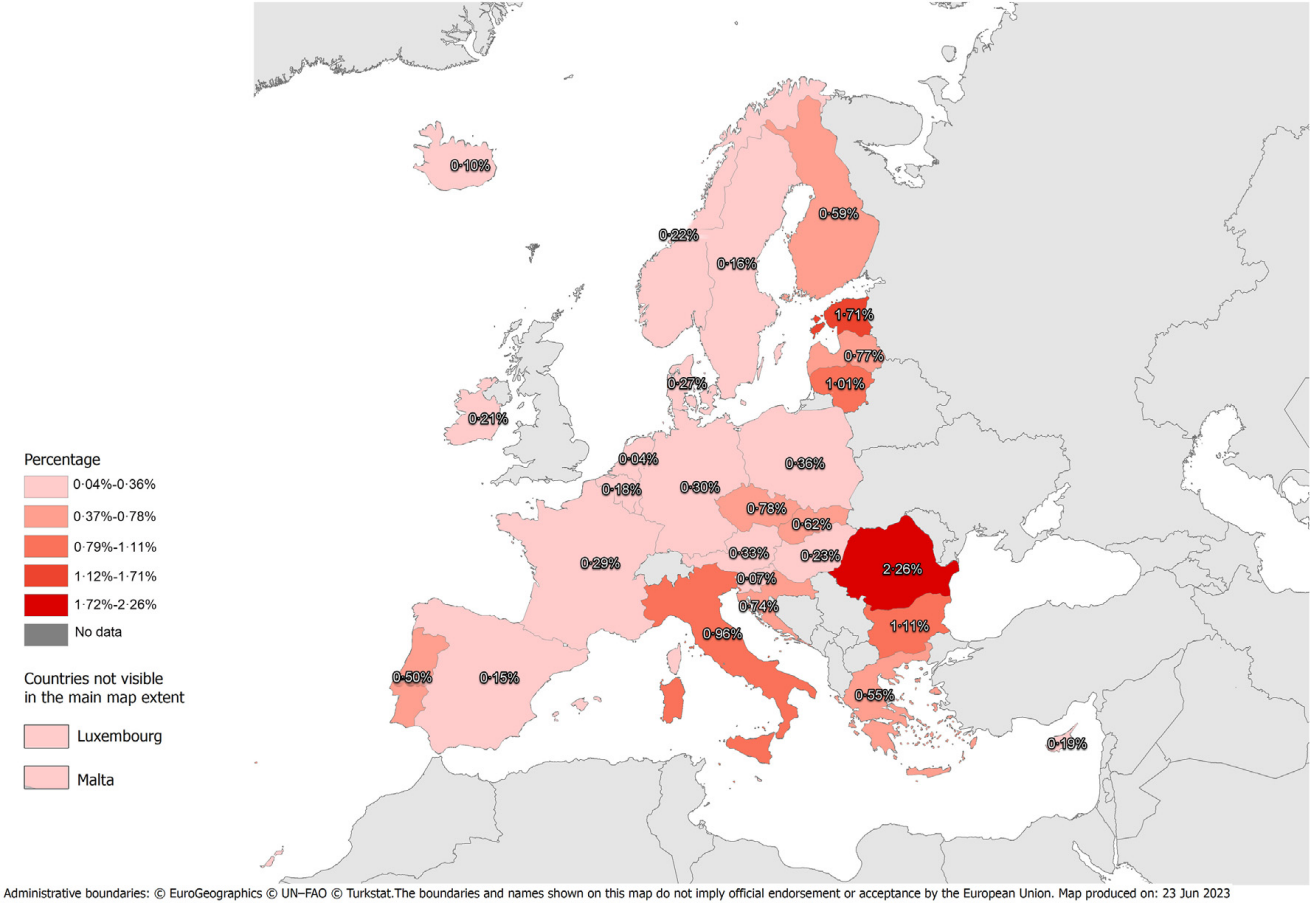
Prevalence (%) of chronic hepatitis C virus (HCV) infection (cHCV) in European Union—European Economic Area (EU/EEA) countries. Notes: HCV-RNA prevalence is used as a proxy for cHCV; Population: 15–79 years old.

The estimated cHCV prevalence in 29 of 30 EU/EEA countries (except Lichtenstein) at the end of 2019 was 0.50% [95% Credible Interval (CrI): 0.46%, 0.55%] (Table 1 and Fig. 1). The EU/EEA regions with the highest and lowest cHCV prevalence were Eastern Europe (0.88%; 95% CrI: 0.81%, 0.94%) and Western Europe (0.27%; 95% CrI: 0.20%, 0.34%). The cHCV prevalence in Northern and Southern Europe was estimated to be 0.41% (95% CrI: 0.37%, 0.45%) and 0.59% (95% CrI: 0.50%, 0.69%), respectively.

In 2019, 25 EU/EEA countries had a cHCV prevalence of less than 1%. The Netherlands (0.04%; 95% CrI: 0%, 0.16%), Slovenia (0.07%; 95% CrI: 0.02%, 0.14%), and Iceland (0.10%; 95% CrI: 0.05%, 0.20%) were estimated to have the lowest cHCV prevalence. In contrast, the highest cHCV prevalence was estimated in Romania (2.26%; 95% CrI: 2.11%, 2.41%), Estonia (1.71%; 95% CrI: 1.49%, 2.06%), and Bulgaria (1.11%; 95% CrI: 0.83%, 1.60%).

### Number of people living with cHCV (absolute numbers)

Overall, the number of people with cHCV at the end of 2019 was estimated at 1,782,923 (95% CrI: 1,638,132, 1,941,583) (Table 1). The estimated population with cHCV in Eastern (six countries), Northern (nine countries), Southern (eight countries), and Western Europe (six countries) was 634,273 (95% CrI: 587,854, 684,696), 120,493 (95% CrI: 109,815, 134,393), 633,109 (95% CrI: 540,571, 737,905), and 391,737 (95% CrI: 297,454, 504,226), respectively.

The three countries with the largest population with cHCV were Italy (459,000; 95% CrI: 379,172, 549,698), Romania (348,939; 95% CrI: 326,554, 372,034), and Germany (196,671; 95% CrI: 137,554, 279,639). The smallest populations with cHCV were estimated for Iceland (279; 95% CrI: 151, 547), Slovenia (1078; 95% CrI: 317, 2319), and Malta (1083; 95% CrI: 812, 1398).

### Proportion of cHCV attributable to IDU and to other/unknown routes

**Fig. 2:**
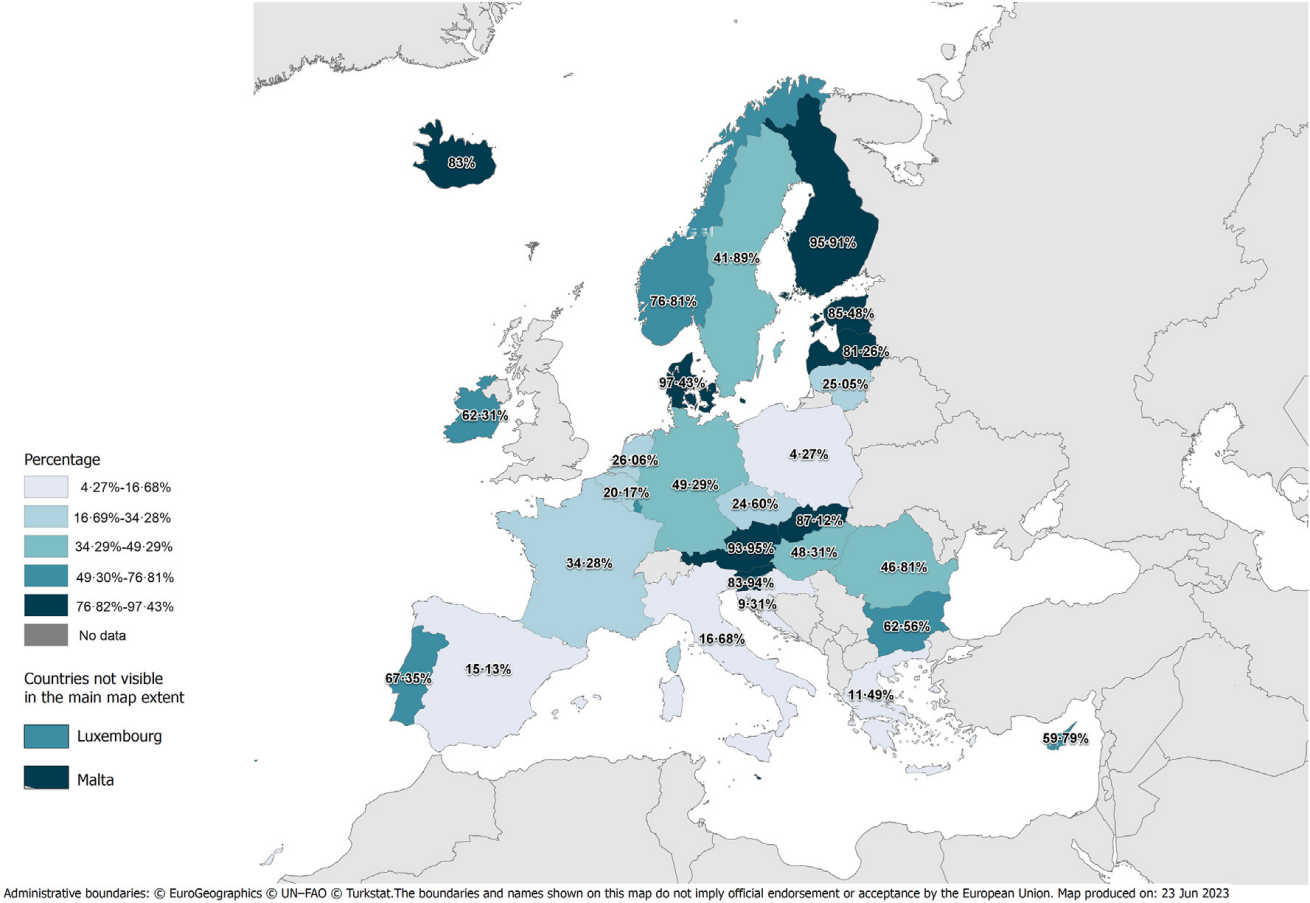
Least proportion (%) of prevalence of chronic hepatitis C virus (HCV) infection (cHCV) that is attributed to injection drug use in the European Union—European Economic Area (EU/EEA). Notes: HCV-RNA prevalence is used as a proxy for cHCV; Population: 15–79 years old.

The model estimated that at least 35.76% (95% CrI: 33.07%, 38.60%) of the 2019 cHCV population was associated with IDU (Table 1 and Fig. 2). The highest and lowest contribution of IDU to the 2019 cHCV prevalence was estimated in Northern (70.03%; 95% CrI: 65.03%, 73.98%) and Southern Europe (19.38%; 95% CrI: 16.66%, 22.67%), respectively. The least contribution of IDU to the 2019 cHCV prevalence in Eastern and Western Europe was 40.34% (95% CrI: 37.14%, 43.58%) and 44.63% (95% CrI: 36.45%, 53.68%), respectively.

The top three countries, where the majority of the 2019 cHCV infections were attributed to IDU, included Denmark (97.43%; 95% CrI: 96.05%, 98.45%), Finland (95.91%; 95% CrI: 94.66%, 96.94%), and Austria (93.95%; 95% CrI: 91.72%, 95.72%). In contrast, the top three countries, where the majority of the 2019 cHCV infections were attributed to other/unknown routes, included Poland (95.73%; 95% CrI: 94.17%, 96.84%), Croatia (90.69%; 95% CrI: 82.43%, 95.08%), and Greece (88.51%; 95% CrI: 82.28%, 92.25%).

## Discussion

Despite considerable progress over the last years, mostly because of effective primary prevention and treatment with DAAs, there are still barriers and gaps to overcome, and it is paramount to find appropriate solutions for the realisation of nationwide HCV elimination programs. In order to meet the 2030 elimination targets, it is essential to accurately monitor the epidemiological trends of cHCV.^25^ Based on a collaborative effort of local experts and a MPES approach, the estimated overall cHCV prevalence in 2019 among people 15–79 years of age in 29 out of 30 EU/EEA countries was 0.50%. The highest estimates were observed in Eastern European countries (0.88%) and the lowest in Western European countries (0.27%). This prevalence estimate corresponded to approximately 1,800,000 viremic infections, with at least one third of them attributed to IDU. The contribution of IDU varied across regions with the highest proportion observed in Northern European countries (70%) and the lowest in Southern European countries (19%) indicating the differing epidemics and testing patterns of hepatitis C in these regions and the need for prevention and control activities targeted to the local epidemiology.

Based on the MPES approach, at the country level, there were examples of very low estimated HCV prevalence (<0.1%), such as in Iceland,^26^ Slovenia, and the Netherlands. In contrast, countries with a higher prevalence (>1.5%), such as Romania or Estonia, will likely require additional resources to scale up services in order to meet the targets for HCV elimination. Moreover, some countries with low estimated cHCV prevalence (for instance, Germany and France), still contribute substantially to the hepatitis C burden in Europe due to their large population size and need to maintain prevention and control strategies.

Other research teams have also tried to estimate the burden of cHCV. The recent study from Polaris Observatory HCV Collaborators estimated, using mathematical modelling, the global prevalence of viremic HCV infection across all age groups in early 2020 to be 0.7%, which was lower than the previous estimate of 0.9% in 2015.^1^ The Global Burden of Disease Study 2019 estimated that the prevalence of HCV-related cirrhosis and other chronic liver diseases in Europe declined by approximately 6% between 2010 and 2019.^27^ This decrease in prevalence of cHCV and its complications could be attributed to the widespread rollout of the highly effective DAAs, but may also be due to reductions in HCV incidence as well as due to deaths and outward migration of cases over time.^27^, ^28^, ^29^, ^30^, ^31^, ^32^, ^33^, ^34^ However, the success in reducing cHCV prevalence has been variable across countries and regions depending on the availability of testing services and treatment, as well as the uptake of primary prevention, especially among PWID.^35^^,^^36^

The Polaris group^1^ also generated country-level estimates of cHCV and, in comparison with our figures, there were several EU/EEA countries with similar estimates and overlapping 95% confidence/credible intervals from the two approaches, which suggests that the true estimate for these countries probably lies close to the estimates provided by these two methods (for example, Spain, Slovenia, and Iceland). Nevertheless, there were also country-level differences between the two methods, which could be partially explained by the different methodology (mathematical modelling vs. MPES) or different country-specific inputs.

Our approach has a number of strengths including: i) high coverage involving 29 of 30 EU/EEA countries, ii) engagement of panels of local country experts including epidemiologists from the corresponding public health agencies and clinicians who, through an iterative and interactive process, gave valuable feedback, validated the data used for inputs, and provided updated local information, iii) use of a common and straightforward approach for all EU/EEA countries that allows reliable country-level or regional comparisons; it should be noted that this method has been used successfully in other settings such as to calculate the prevalence of antibodies to HCV infection in England and Wales^15^ and in New York City,^37^ and to estimate the number of ever injectors with undiagnosed HCV infection in Scotland,^38^ iv) simultaneous modelling of all data from multiple sources in a Bayesian framework, v) consideration of the administration and impact of DAAs on prevalence, and finally vi) the possibility of easy and quick update of the results from our approach when new information is identified or becomes available allowing thus also the evaluation of trends and changes over time. To allow for reproducible and transparent research, computing codes of both the Markov model and the MPES method are available in the country-specific reports included as a supplementary material (Appendix B).

There are also a few limitations to our approach: Firstly, the quality of the input data affects the accuracy of the final estimations. Quite often, prevalence studies conducted in PWID or in non-drug-using groups were of weak methodological design. For example, many studies did not recruit participants using a random sampling scheme and were thus less likely to be representative of the target population. For the non-PWID population, some data came from studies relating to pregnant women or from the testing of blood donors, which may underestimate the true prevalence or the attribution of the overall cHCV burden to the non-PWID population. Furthermore, in most cases, the participation rates, even in well-designed studies, were unknown and thus assessment of non-response bias was not possible. To minimise bias, an algorithm was introduced for the selection of studies conducted in the general population prioritising evidence that had received a lower risk of bias score in the ECDC review. The selection process regarding the studies on cHCV prevalence among PWID and the number of recent PWID (ideally those using capture-recapture sampling) was not arbitrary either; relevant studies were initially obtained from EMCDDA with the final decision on which studies to include reached through extensive consultation and collaboration with the NCPs. However, a formal algorithm assessing study quality, such as the one used in the general population, was not applied. Thus, cHCV prevalence data relating to PWID were often retrieved from the testing of PWID at drug treatment facilities and this population may not be representative of all PWID. Moreover, drug use is associated with stigma and so self-reported information might be less reliable.

Although there were many countries with well-designed national surveys for cHCV in the general population, within-country heterogeneity by region, age, or gender could also be an issue. However, this stratification was not possible to perform for most countries due to lack of data. There is also significant between-country heterogeneity, even among neighboring countries. Thus, the total and regional estimates at the European level should be interpreted as summary statistics.

Given that the MPES is based on defining non-overlapping groups, to the extent the relevant information was available, we excluded PWID-related data from studies conducted in the general population. A further major limitation, however, was that some vulnerable populations have probably not been considered or modelled appropriately (MSM or chemsex users, people with migratory background from high endemicity countries). For example, people with migratory background who did not use drugs recently or in the past are likely to be underrepresented in surveys among the general population due to language issues or because they are more challenging to reach. Sensitivity analyses were conducted where people with migratory background were considered as a separate group using estimates of their population size and of cHCV among them from a ECDC review^23^; however, the MPES estimates from these sensitivity analyses were probably biased because the degree of overlapping between people with migratory background and other groups is unknown. On most occasions, NCPs considered the MPES figures from these sensitivity analyses as overestimates and did not endorse them. Nevertheless, incorporating other risk groups, such as MSM, could be relevant in some circumstances, depending upon the local epidemiological situation and the availability of data. In the Netherlands, for example, we explored including MSM with HIV as a separate risk group, following discussions with local experts. However, extending this approach to all countries presents challenges. Firstly, it necessitates the availability of studies on cHCV prevalence among MSM, which is not the case for most countries.^8^^,^^12^ Secondly, there is again potential overlapping between groups: non-PWID MSM are likely already included in general population studies, while MSM who inject drugs are probably included in PWID studies. This suggests a probable two-way overlapping, potentially resulting in an overestimation of the overall cHCV prevalence. The exception is perhaps MSM who inject drugs for chemsex, as they are not likely to participate in either general population or PWID studies.

Another limitation was the lack of the necessary data on population group size or on HCV prevalence for a very small number of countries. The problem was partially addressed by using information from neighboring countries unless the national experts considered that these estimates could not or did not reflect the reality in their own countries. Reinfection was also a further limitation that was not specifically taken into consideration in the MPES estimates.^39^ Moreover, on a few occasions, there were differences in the MPES estimates between some countries whose HCV prevalence estimates were much closer in magnitude based on other modelling approaches.^40^ A further issue is that cHCV refers primarily to HCV-RNA positive people, which may slightly overestimate the number of chronically infected because it might include a small number of acute infections that cleared spontaneously. Another limitation is that the number of ex-PWID is in general difficult to estimate directly (e.g., through surveys). In this work, we estimated it through a mathematical modelling approach, requiring further assumptions which can impact the results. A final limitation was that we had to adjust results based on the prevalence of antibodies to HCV using a common factor for spontaneous clearance, which may not be correct in some countries. However, this could normally be addressed with direct information on the prevalence of viremic HCV infection or on DAAs.

Our MPES is a simple version of that proposed by Sweeting et al.^15^ that could be significantly improved if: i) more complete data, through mandatory and comprehensive reporting systems, on HCV cases and DAAs uptake, overall but especially by injection status, were available for all EU/EEA countries, ii) definitions in studies among PWID clearly differentiated recent from ex-PWID, iii) higher quality and more representative studies in the general population, in PWID but also in other key populations (e.g., MSM, people with a migratory background) were conducted measuring the prevalence of viremic infection; for instance, Romania, a country with high cHCV prevalence in 2019, released results from a well-conducted study in the general population in 2022–2023, which will help update the national estimate,^41^ and iv) the methodology was expanded to estimate not only the prevalence of cHCV but also what proportion of people with cHCV are diagnosed, linked to care, and started on DAAs.

### Conclusion

The results of our MPES study to derive national estimates of cHCV indicate that the overall prevalence of cHCV is low across the EU/EEA, but remains high in some countries and among PWID, although our findings should be interpreted with caution due to limitations of this methodological approach. The total number of cHCV cases in the region is substantial and EU/EEA countries need to continue their efforts to eliminate HCV by scaling up testing alongside harm reduction measures and timely linkage to treatment.^42^ The estimates presented in this analysis alongside the collection of data on the undiagnosed fraction of cHCV infections from other analyses are core elements of information needed by EU/EEA countries to review and adapt their local HCV strategies, including their screening and harm reduction policies (i.e., needles and syringes programmes, opioid agonist therapy, and take-home naloxone), and enable the monitoring of progress towards the 2030 elimination targets.

## Contributors

ED, IG, CT, and GN conceptualized the study. ED and GN supervised the study and were responsible for the administration of the project. All authors contributed to investigation and data collection. IG, KG, CT, BB, TS, ED, and GN designed the methodology. IG, KG, CT, and GN conducted the data analysis. All authors contributed to data validation and interpretation. IG, CT, and GN directly assessed and verified the underlying data reported in the manuscript. IG, KG, and CT were responsible for data visualisation. IG, KG, CT, and GN wrote the original draft. All authors participated in writing, reviewed, and edited the manuscript. All authors had full access to all the data in the study, agreed on the content of the manuscript, and approved the final version.

## Data sharing statement

All data collected for this manuscript, all individual country reports including data sources, methodology, results, and analysis codes are available at http://hcveurope.eu/ and in Appendix B.

## Declaration of interests

IG: He is currently an employee of MSD Greece. He joined MSD after his post-doctoral work at the University of Cyprus. TV: He has received grants from Gilead Sciences and Bristol Myers Squibb; he has served as a consultant for Janssen Pharmaceuticals, Gilead Sciences, AbbVie, Bristol Myers Squibb; and he has served as a sponsored lecturer for Gilead Sciences and Abbvie. PBC: He has received unrestricted research grants for other studies from Abbvie, Gilead, and MSD. MG: He has received consultancy and speaker’s fees from Gilead Sciences. LAK: She has received personal lecturer fee from Abbvie and Gilead Sciences and an institutional grant from Gilead Italy Fellowship 2022. LJ: She has received honorarium for lectures from AbbVie and MSD; offered consultancy to AbbVie, MSD, Tamro; and received conference attending fee from AbbVie, MSD, Pfizer, Swixx Biopharma. CSD: She has received educational and research grants for other studies from Abbvie and Gilead Sciences. MV: He participated in advisory boards (ViiV, Gilead, and MSD–fees paid to his institution); he has received independent research grants from ViiV and Gilead (paid to his institution). CV: She has received honorarium for lectures and consultancy from AbbVie, Gilead, MSD, and ViiV Healthcare. AD: She has received a grant for another study and speaker fee at a conference about HIV from Gilead Sciences. AB: He has received speaker's fees from Gilead Sciences. GN: He has received an ASKLEPIOS grant (HIV-related competitive grant) from Gilead Sciences (Greece). All other authors declare no conflict of interest.

## Appendix A Supplementary data

Appendix B

Protocol FINAL
