# Supplementary material for: Prevalence of chronic HCV infection in EU/EEA countries in 2019 using multiparameter evidence synthesis
Source: Lancet Reg Health Eur. 2023 Dec 13;36:100792. doi: 10.1016/j.lanepe.2023.100792 (PMC10769889; doi:10.1016/j.lanepe.2023.100792)
Supplement: Appendix A [file mmc1.pdf]

Prevalence of chronic HCV infection in EU/EEA countries in 2019 using multiparameter evidence synthesis

Appendix

Contents

Markov model......2

Schematic outline of the mathematical model......2

Goodness-of-fit metric (GoF)......3

Type of model......3

Programming language......3

Fit of the MPES model......5

Proportion of recent PWID, ex-PWID, and non-PWID in the population (ρrec, ρex, ρnon)......5

Prevalence of chronic HCV (cHCV) among recent PWID (πrec)......5

Prevalence of cHCV among ex-PWID (πex)......5

Prevalence of cHCV among non-PWID (πnon)......6

Adjustment for treatment with direct-acting antivirals (DAAs)......6

Sensitivity analyses......7

Geographical divisions......8

References......9

Markov model

Schematic outline of the mathematical model

Figure S1: Schematic outline of the mathematical model. Parameters: β: flow rate of new individuals in the model, Pα: annual transition probability of non-PWID (people who inject drugs) to recent PWID, Pk: annual transition probability of relapse of ex-PWID to recent PWID, Pγ: annual transition probability of recent PWID to ex-PWID, Pμ: annual mortality probability (Pμ is higher in the population of PWID).

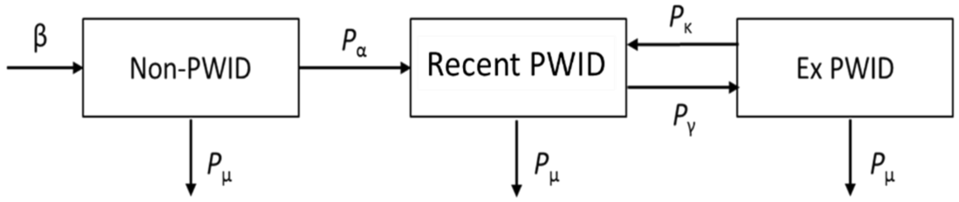

The starting year of the simulation is set arbitrarily to the year 1950. Initially, the 15–79-year-old population in that year is seeded in the model. Every simulation year a group of 15-year-old individuals enters the non-PWID compartment while those aged older than 80 years old leave the model. Population data for each country were retrieved from the United Nations (UN) Population Division.[1] Individuals from the non-PWID compartment, aged between 15 and 39 years old, can transit to the recent PWID compartment through a fixed annual transition probability (Pα). After the age of 39 years, it is assumed that initiation of drug injection is unlikely. When countries had local data on the age of injecting cessation, the model was updated considering the country-specific data (e.g., Belgium-see Appendix B). PWID can move from their compartment to that of ex-PWID through a fixed annual probability of ceasing injecting (Pγ).[2] Ex-PWID can relapse (move back to the recent PWID compartment) with a fixed annual probability (Pκ).[3] Ageing is taken into consideration so that the total size of both recent and ex-PWID sub-populations for a given age range at a given time can be estimated. Individuals can leave the model through country-specific background mortality by applying the national age-specific all-cause mortality rates (World Health Organization - Life tables).[4] PWID-related excess mortality was also considered in the model.[5] The interpretation of the parameters used in the model is also presented in Table S1.

Table S1: Interpretation of each parameter in the Markov model and source of its estimate.

Symbol	Parameter	Value	Source
β	Annual number of 15-year-old individuals entering the population	Country-specific	United Nations Population Division[1]
P_γ	Annual transition probability of active people who inject drugs (PWID) becoming ex-PWID	Rate: 1 / country-specific duration of injecting drugs	Hines et al 2020[2]
P_κ	Annual transition probability of relapse – ex-PWID to active PWID	Rate: 0.004/month	De Vos et al 2013[3]
P_μ	Annual mortality probability	Rate: μ (country and age-specific)	World Health Organization national mortality rates[4]
Ω	Excess mortality	13-fold increase for active PWID	Mothers et al 2013[5]

P_α	Annual transition probability – non-PWID to active PWID	Model fit	Estimated parameter

The model is calibrated on the number of recent PWID reported either in the review of Grebely et al[6] or in the European Monitoring Centre for Drugs and Drug Addiction (EMCDDA) statistical bulletin (https://www.emcdda.europa.eu/data/stats2021/pdu_en; last time accessed: October 7th, 2023) / Barometer (https://www.emcdda.europa.eu/publications/html/viral-hepatitis-elimination-barometer_en; last time accessed: October 7th, 2023) or by the European Centre for Disease Prevention and Control (ECDC) national operational contact points (NCPs) and other national experts (Figures S2a and S2b). By fitting the model to the observed calibration point, the annual transition probability Pα of becoming recent PWID was adjusted so as the simulated recent PWID population size fitted the targeted data point. After the identification of the most appropriate annual transition probability Pα of becoming recent PWID, the model was used to estimate the number of recent and ex-PWID in the population in 2019. The size of non-PWID was estimated by subtracting the number of recent and ex-PWID from the total size of the population of each country derived from Eurostat. Finally, $\rho_{rec}, \rho_{ex}\ and\ \rho_{non}$ were estimated by dividing the above-mentioned numbers by the total population (15-79 years old) of each country.

Goodness-of-fit metric (GoF)

A GoF metric assists, as the objective function in an optimization procedure, in measuring the accuracy of the predictions of the model against the targets. In our model, the least squares method was used to measure the accuracy. Smaller values of the GoF metric indicate better fit to the observed data.

Type of model

In our analysis, a discrete-time, stochastic, individual-based model (IBM) was used. IBM simulates people's trajectories at the individual level. It is important to note that these models possess inherent randomness due to their methodology. The way that the model examines if a pseudo individual would change state is through the draw of random numbers. More specifically, the model estimates the probability of moving from one stage to the next. Then, for each pseudo individual, a random number is drawn. If the resulting random number (e.g., 0·3) is smaller than the estimated probability of changing stage (e.g., 0·4), this pseudo individual changes stage and vice versa. For example, regarding the transition from recent PWID to ex-PWID, if the risk of stopping injection of drugs is 20%, then all recent PWID with drawn random numbers lying in the range of (0-0·2) are assumed to become ex-PWID. As the outcome of each run depends on chance, every simulation leads to slightly different results. Uncertainty comes from a single set of parameters but across multiple simulations with randomness included. For that, results over all simulations are pooled and the median along a range is normally presented (stochastic variability). In these models, in order for the results to be reliable, several runs should be conducted since if the number of runs is limited, extreme results from simulations would affect significantly the pooled estimates. The model for each country included 1000 runs. To represent uncertainty in model projections (stochastic variability), the median and 2.5th / 97.5th percentiles (95% Credible Interval - CrI) are shown. The value of 1000 was chosen so that the distribution (median and CrIs) is stabilized (i.e., it remains unchanged after further increasing the number of runs).

For more details regarding the IBM models one could look at the following reference book.[7]

Programming language

The simulations were performed in the low-level programming language C++ (Dev-C++ v.5.11) and the graphs were produced in Stata 16.1.

Figure S2a: Results of the Markov model in selected countries (Greece and Croatia).

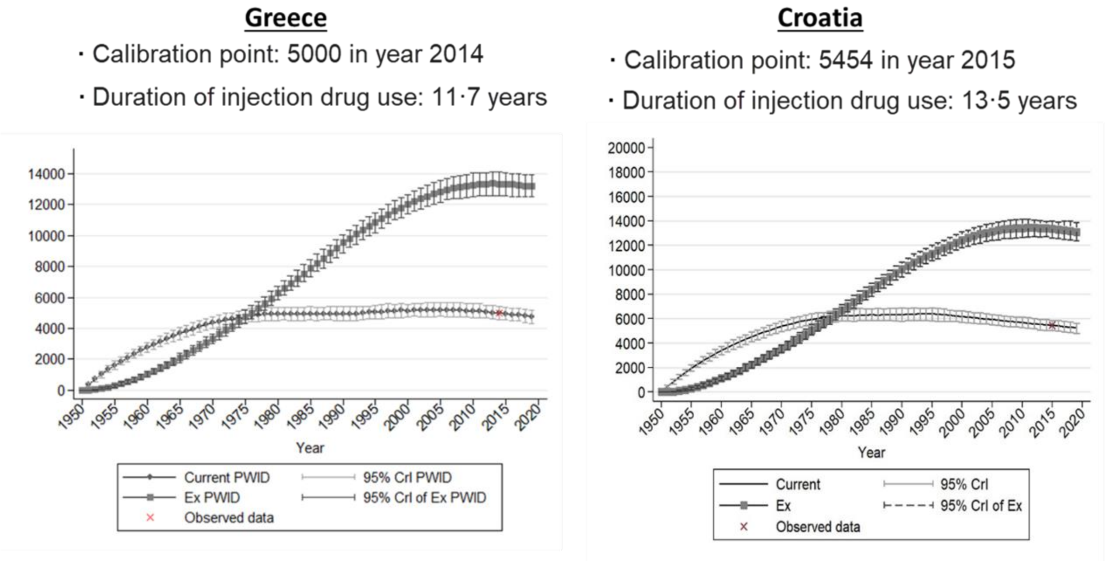

Figure S2b: Results of the Markov model in selected countries (Belgium and Bulgaria).

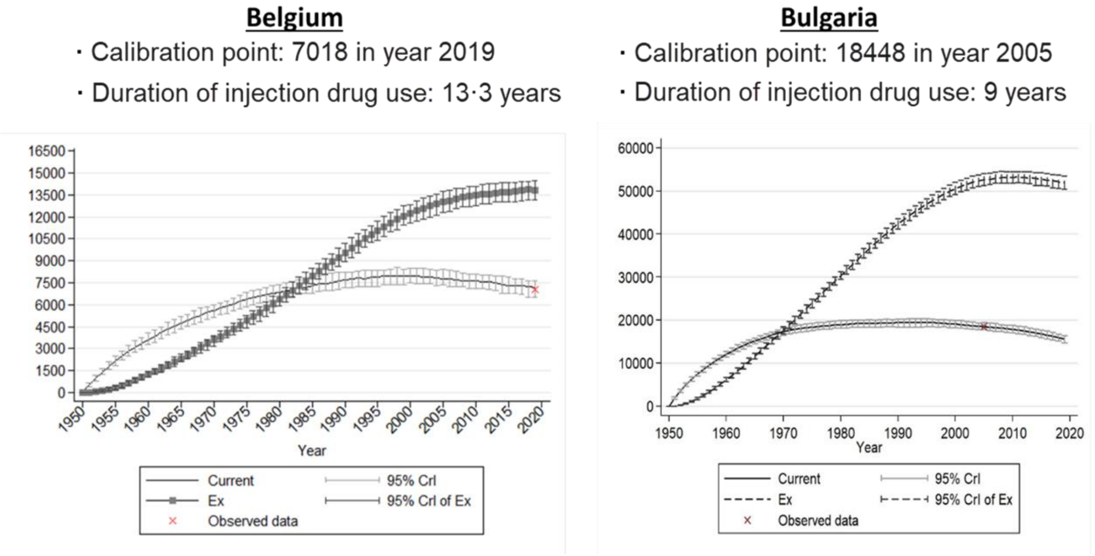

Notes: Details about the sources of the calibration points can be found in the reports for each country (http://hcveurope.eu/ and in Appendix B).

PWID: People who inject drugs

CrI: Credible Interval

Fit of the MPES model

The unified Multi-Parameter Evidence Synthesis (MPES) model was fitted under a Bayesian approach using Hamiltonian Monte Carlo (HMC) through the STAN software. HMC is a Markov chain Monte Carlo (MCMC) method that uses the derivatives of the density function being sampled to generate efficient transitions spanning the space of the posterior, thereby avoiding the random-walk behavior that arises in standard MCMC samplers when there is correlation in the posterior distribution. Vague, uniform from 0 to 1, prior distributions were specified for the hepatitis C virus (HCV) prevalence (chronic HCV – cHCV) probabilities. Since most data were available in the form of numerator and denominator, independent Binomial distributions were used. In total, 30,000 iterations were used, with the first 10,000 discarded as a burn-in (warm-up) period. We also ran parallel chains of the algorithm and, based on visual inspection of trace plots and relevant statistical tests, there was no indication of convergence issues. In the following paragraphs, we describe in more detail the procedure used for each parameter of the MPES approach. Most HCV prevalence sources were after 2010. Country-specific HCV data were used in all but two countries (Croatia and Estonia), i.e., in ~93·1% (27/29) of the countries.

Proportion of recent PWID, ex-PWID, and non-PWID in the population (ρ_{rec}, ρ_{ex}, ρ_{non})

The numbers/proportions of recent and ex-PWID were estimated based on the stochastic, individual-based multi-state Markov model described above. After applying the Markov model for each country, we computed the number (and the corresponding CrI) of recent and ex-PWID in 2019. The numbers of recent and ex-PWID were then divided by the population size (15-79 years) in the same year, and independent Normal distributions, constrained such that $\rho_{rec} \in (0,1)$ and $\rho_{ex} \in (0,1)$, were assumed for ρ_{rec} and ρ_{ex}. The standard deviations (in the absence of constraints) of these distributions were specified to approximately correspond to the respective CrIs: e.g., for ρ_{ex}, the standard deviation is assumed to be equal to $\left(UB_{\rho_{ex}} - LB_{\rho_{ex}}\right)/(2 \times 1 \cdot 96)$, where $UB_{\rho_{ex}}$ and $LB_{\rho_{ex}}$ denote the upper and lower limit of the CrI. The standard deviation for ρ_{rec} was specified accordingly. These distributions were then incorporated into the MPES methodology as priors.

Prevalence of chronic HCV (cHCV) among recent PWID (π_{rec})

The prevalence (and the 95% CrI) of cHCV among recent PWID was informed ideally by respondent-driven sampling (RDS) studies or other studies published in the literature, as suggested by the ECDC NCPs. If no such studies were available, estimates from the EMCDDA 2021 or 2022 statistical bulletin were used. Finally, if EMCDDA data were also unavailable, estimates from the paper of Grebely et al 2019[6] were used. However, data from some countries referred to the prevalence of antibodies to HCV (anti-HCV) and not to the prevalence of cHCV (viremic population, i.e., positive for HCV-RNA or the HCV core antigen) and, consequently, the estimates could not be used directly. This issue was addressed by noting that spontaneous viral clearance occurs in approximately one in four people with acute hepatitis C. To account for the variability of the HCV clearance probabilities, we used the result reported in Micallef et al,[8] i.e., the proportion of HCV clearance (denoted by ρ_{clear}) is equal to 0·26 [95% Confidence Interval (CI): 0·22–0·29]. Thus, estimates of the cHCV prevalence among recent PWID (π_{rec}) were obtained using the formula

$$\pi_{rec} = \pi(\text{anti-HCV})_{rec}(1 - \rho_{clear}),$$

where $\pi(\text{anti-HCV})_{rec}$ denotes the anti-HCV prevalence among recent PWID.

Prevalence of cHCV among ex-PWID (π_{ex})

Direct information on cHCV prevalence among ex-PWID was not available for most countries. When there were available data for π_{ex}, though, we used them directly (for example, in Spain). To overcome this problem, data on cHCV prevalence among ever PWID (indirect information) obtained through the EMCDDA database or other sources suggested by NCPs were used. However, cHCV prevalence among ever PWID (including both active PWID as well as those who injected in the past) provides information on a mixture of the parameters of interest, i.e., π_{rec} and π_{ex}. Note also that if the data on ever PWID corresponded to anti-HCV prevalence, they were firstly adjusted as described above, i.e.,

$$\pi_{ever} = \pi(\text{anti-HCV})_{ever}(1 - \rho_{clear}).$$

Then, unless additional information was provided, we used the estimated population risk-group proportions by the Markov model as the mixture proportions, i.e.,

$$\pi_{ever} = \frac{\rho_{rec}}{\rho_{rec} + \rho_{ex}}\pi_{rec} + \frac{\rho_{ex}}{\rho_{rec} + \rho_{ex}}\pi_{ex}$$

Therefore, π_{ex} was indirectly computed by applying the formula below:

$$\pi_{ex} = \left(\pi_{ever} - \frac{\rho_{rec}}{\rho_{rec} + \rho_{ex}}\pi_{rec}\right) \times \frac{\rho_{rec} + \rho_{ex}}{\rho_{ex}}$$

In some cases, the mixture proportion of recent PWID among ever PWID, say ω, was known or reliably estimated (for example, in France). If so, we adopted ω and $1 - \omega$ as the mixture proportions of recent and ex-PWID, respectively, i.e.,

$$\pi_{ever} = \omega\pi_{rec} + (1 - \omega)\pi_{ex},$$

and estimated π_{ex} similarly.

Prevalence of cHCV among non-PWID (π_{non})

We directly used cHCV prevalence data from studies in the general population, if available, or other studies as suggested by NCPs. If the data in the general population referred to anti-HCV prevalence (without any data on the viremic population), we adjusted the estimates to get the cHCV prevalence based on the spontaneous HCV clearance estimate of 26%,[8] as previously described.

Adjustment for treatment with direct-acting antivirals (DAAs)

After performing the above-mentioned procedures, we accounted for the fact that some people have been treated with DAAs, with the sustained virologic response (SVR_{PWID}) among PWID estimated to be 88% (95% CI: 80% to 93%)[9] and among non-PWID 96.7% (95% CI: 95.4% to 98.1%).[10] The total number of individuals treated with DAAs up to 2019, N_{DAA}, was provided by NCPs. We adjusted the cHCV prevalence estimates among recent, ex-, and non-PWID by stochastically subtracting the corresponding number of individuals cured by DAAs from the respective cHCV population using the formulas:

$$\pi_{rec} = \frac{N_{15,79}\rho_{rec}\tilde{\pi}_{rec} - N_{DAA}\rho_{rec|DAA}SVR_{PWID}}{N_{15,79}\rho_{rec}}$$

$$\pi_{ex} = \frac{N_{15,79}\rho_{ex}\tilde{\pi}_{ex} - N_{DAA}\rho_{ex|DAA}SVR_{PWID}}{N_{15,79}\rho_{ex}}$$

$$\pi_{non} = \frac{N_{15,79}\rho_{non}\tilde{\pi}_{non} - N_{DAA}\rho_{non|DAA}SVR}{N_{15,79}\rho_{non}}$$

where $\tilde{\pi}_{rec}$, $\tilde{\pi}_{ex}$, $\tilde{\pi}_{non}$ denote the cHCV prevalence estimates ignoring the effect of DAAs among recent, ex-, and non-PWID respectively, $\rho_{rec|DAA}$, $\rho_{ex|DAA}$, and $\rho_{non|DAA}$ denote the proportions of recent, ex-, and non-PWID among those who were treated with DAAs, and SVR and SVR_{PWID} the estimated SVR rates among non-PWID and PWID, respectively. The uncertainty of SVR rates was considered by assuming normal distributions matching the 95% CIs reported in Lampertico et al[10] and Graf et al.[9] If the estimates $\tilde{\pi}_{rec}$, $\tilde{\pi}_{ex}$, $\tilde{\pi}_{non}$ were obtained by adjusting the corresponding anti-HCV prevalence for spontaneous clearance, the total number of DAAs up to 2019 was used (N_{DAA}). However, if $\tilde{\pi}_{rec}$, $\tilde{\pi}_{ex}$, $\tilde{\pi}_{non}$ were based on cHCV estimates before 2019, the number of DAAs after the year of the respective study was used instead. It should be also noted that when cHCV data were available in 2019 for some of the three groups, no DAA adjustment was made for that group.

The proportions of the three groups among those treated were unknown for most of the countries. If so, we assumed that $\rho_{rec|DAA}$ is equal to the proportion of recent PWID among cHCV-positive individuals, i.e., $\Pr(\text{Recent PWID}|\text{cHCV})$, as estimated by our model (using the Bayes rule) when information on DAAs is ignored. Similarly, we assumed that $\rho_{ex|DAA} = \Pr(\text{Ex-PWID}|\text{cHCV})$ and $\rho_{non|DAA} = \Pr(\text{Non-PWID}|\text{cHCV})$. Moreover, in some countries, the proportion of recent PWID among those treated was set equal to zero ($\rho_{rec|DAA} = 0$) upon suggestion from the NCP or other national expert, and thus, treatment adjustment for the cHCV prevalence among recent PWID was not performed. In these cases, the proportions of ex- and non-PWID among those treated with DAAs were assumed to be proportional to the corresponding proportions of ex- and non-PWID among cHCV-positive individuals, i.e.,

$$\rho_{ex|DAA} = \frac{\Pr(\text{Ex-PWID}|\text{cHCV})}{\Pr(\text{Ex-PWID}|\text{cHCV}) + \Pr(\text{Non-PWID}|\text{cHCV})}$$

and

$$\rho_{non|DAA} = \frac{\Pr(\text{Non-PWID}|\text{cHCV})}{\Pr(\text{Ex-PWID}|\text{cHCV}) + \Pr(\text{Non-PWID}|\text{cHCV})}$$

as estimated by our model when the DAA uptake is ignored.

In other countries, the proportion of ever PWID among those treated with DAAs ($\rho_{ever|DAA}$) was known or adequately estimated (equivalently, the proportion of non-PWID among those treated was equal to $\rho_{non|DAA} = 1 - \rho_{ever|DAA}$). In these cases, among treated ever PWID, we assumed that treatment was distributed proportionally to the proportion of recent and ex-PWID among cHCV-positive individuals, Pr(Recent PWID|cHCV) and Pr(Ex-PWID|cHCV), as estimated by our model when the DAA uptake is ignored. Formally, we assumed that

$$\rho_{rec|DAA} = \rho_{ever|DAA} \frac{\Pr(\text{Recent PWID}|\text{cHCV})}{\Pr(\text{Recent PWID}|\text{cHCV})+\Pr(\text{Ex-PWID}|\text{cHCV})},$$

$$\rho_{ex|DAA} = \rho_{ever|DAA} \frac{\Pr(\text{Ex-PWID}|\text{cHCV})}{\Pr(\text{Recent PWID}|\text{cHCV})+\Pr(\text{Ex-PWID}|\text{cHCV})},$$

$$\rho_{non|DAA} = 1 - \rho_{ever|DAA}$$

Data on the number of people treated with DAAs were available and taken into account in 20 out of 29 countries (~69%). The countries for which the number of DAAs was not considered were Portugal, Czechia, Austria, Estonia, Romania, Hungary, Cyprus, Bulgaria, and Belgium. For Belgium, though, treatment was at least partly considered; more information can be found in the country reports (Appendix B and at http://hcveurope.eu/).

Two countries (Iceland and Latvia) provided complete information about the proportions of the three risk groups among those treated with DAAs, whereas, in seven countries (Poland, Slovakia, Ireland, Slovenia, Denmark, Finland, and Italy), some information was available (e.g., $\rho_{rec|DAA} = 0$ or $\rho_{ever|DAA}$ being known) and taken into consideration.

Sensitivity analyses

An additional sensitivity analysis including people with migratory background from high endemicity countries as a separate group was performed for each country. Estimates about the respective population size and cHCV prevalence (π_{mig}) were based on a ECDC technical report (https://www.ecdc.europa.eu/sites/default/files/media/en/publications/Publications/epidemiological-assessment-hepatitis-B-and-C-among-migrants-EU-EEA.pdf; last time accessed October 7th, 2023).

In this analysis, the overall cHCV prevalence was estimated by

$$\pi = \pi_{rec}\rho_{rec} + \pi_{ex}\rho_{ex} + \pi_{mig}\rho_{mig} + \pi_{non}\rho_{non},$$

where $\rho_{rec} + \rho_{ex} + \rho_{mig} + \rho_{non} = 1$. A treatment adjustment similar to that previously described was also performed. However, these analyses come with possible limitations; that is, including individuals with migratory background as a separate group is valid only if they do not overlap with the remaining groups (recent PWID, ex-PWID, and non-PWID). Thus, if people with migratory background do participate proportionally in the study(ies) in the general population, these analyses may result in biased overall cHCV estimates (most probably in higher cHCV prevalence estimates).

In some countries, sensitivity analyses were also performed for the risk-group distribution among individuals treated with DAAs. A detailed description of all analyses performed is available in the individual country reports (Appendix B and at http://hcveurope.eu/).

Geographical divisions

Countries were grouped into regions using the definition of the UN Statistics Division (https://unstats.un.org/unsd/methodology/m49/; last time accessed: October 7th, 2023). Sub-regions names denote the European Union (EU)/European Economic Area (EEA) countries within the UN sub-regions. Cyprus was grouped into Southern Europe for this analysis.

Table S2: Sub-regions in the European Union (EU)/European Economic Area (EEA) based on the United Nations (UN) Statistics Division.

**Sub-regions**	**Countries**
Eastern Europe	1. Bulgaria<br>2. Czechia<br>3. Hungary<br>4. Poland<br>5. Romania<br>6. Slovakia
Northern Europe	1. Denmark<br>2. Estonia<br>3. Finland<br>4. Iceland<br>5. Ireland<br>6. Latvia<br>7. Lithuania<br>8. Norway<br>9. Sweden
Southern Europe	1. Croatia<br>2. Cyprus<br>3. Greece<br>4. Italy<br>5. Malta<br>6. Portugal<br>7. Slovenia<br>8. Spain
Western Europe	1. Austria<br>2. Belgium<br>3. France<br>4. Germany<br>5. Luxembourg<br>6. Netherlands

References

1 United Nations (UN). Total Population by country. https://population.un.org/wpp/Download/Standard/MostUsed/. Accessed: October 7th, 2023.

2 Hines LA, Trickey A, Leung J, et al. Associations between national development indicators and the age profile of people who inject drugs: results from a global systematic review and meta-analysis. *Lancet Glob Health.* 2020; **8**: e76–e91.

3 de Vos AS, van der Helm JJ, Matser A, Prins M, Kretzschmar ME. Decline in incidence of HIV and hepatitis C virus infection among injecting drug users in Amsterdam; evidence for harm reduction? *Addiction.* 2013; **108**: 1070–81.

4 World Health Organization (WHO). Global Health Observatory data repository. 2020. https://apps.who.int/gho/data/view.main.60420?lang=en. Accessed: October 7th, 2023.

5 Mathers BM, Degenhardt L, Bucello C, Lemon J, Wiessing L, Hickman M. Mortality among people who inject drugs: a systematic review and meta-analysis. *Bull World Health Organ.* 2013; **91**: 102–23.

6 Grebely J, Larney S, Peacock A, et al. Global, regional, and country-level estimates of hepatitis C infection among people who have recently injected drugs. *Addiction.* 2019; **114**: 150–66.

7 Vynnycky E, White R. An Introduction to Infectious Disease Modelling. Oxford: *Oxford University Press,* 2010.

8 Micallef JM, Kaldor JM, Dore GJ. Spontaneous viral clearance following acute hepatitis C infection: a systematic review of longitudinal studies. *J Viral Hepat.* 2006; **13**: 34–41.

9 Graf C, Mucke MM, Dultz G, et al. Efficacy of Direct-acting Antivirals for Chronic Hepatitis C Virus Infection in People Who Inject Drugs or Receive Opioid Substitution Therapy: A Systematic Review and Meta-analysis. *Clin Infect Dis* 2020; **70**: 2355–65.

10 Lampertico P, Carrion JA, Curry M, et al. Real-world effectiveness and safety of glecaprevir/pibrentasvir for the treatment of patients with chronic HCV infection: A meta-analysis. *J Hepatol* 2020; **72**: 1112–21.
