# Supplementary material for: Prevalence of chronic HCV infection in EU/EEA countries in 2019 using multiparameter evidence synthesis
Source: Lancet Reg Health Eur. 2023 Dec 13;36:100792. doi: 10.1016/j.lanepe.2023.100792 (PMC10769889; doi:10.1016/j.lanepe.2023.100792)
Supplement: Appendix B [file mmc2.pdf]

# Prevalence of chronic HCV infection in EU/EEA countries in 2019 using multiparameter evidence synthesis

## Appendix B

**Detailed reports for each country**

**Austria** ........ **2-29**
**Belgium** ........ **30-56**
**Bulgaria** ........ **57-82**
**Croatia** ........ **83-113**
**Cyprus** ........ **114-140**
**Czechia** ........ **141-167**
**Denmark** ........ **168-200**
**Estonia** ........ **201-227**
**Finland** ........ **228-261**
**France** ........ **262-292**
**Germany** ........ **293-320**
**Greece** ........ **321-346**
**Hungary** ........ **347-375**
**Iceland** ........ **376-401**
**Ireland** ........ **402-433**
**Italy** ........ **434-466**
**Latvia** ........ **467-497**
**Lithuania** ........ **498-530**
**Luxembourg** ........ **531-564**
**Malta** ........ **565-597**
**Netherlands** ........ **598-631**
**Norway** ........ **632-659**
**Poland** ........ **660-690**
**Portugal** ........ **691-718**
**Romania** ........ **719-746**
**Slovakia** ........ **747-776**
**Slovenia** ........ **777-805**
**Spain** ........ **806-834**
**Sweden** ........ **835-865**

# Estimating the prevalence of chronic hepatitis C infection (CHC) in Austria using Bayesian multiparameter evidence synthesis

14/06/2023

## Table of Contents

Introduction ..... 2
Methods ..... 2
- Prevalence of ex-PWID ..... 3
- Prevalence of recent PWID ..... 3
- Prevalence of CHC among recent PWID ..... 4
- Prevalence of CHC among ex-PWID ..... 4
- Prevalence of CHC among non-PWID ..... 5
- Sensitivity analysis including migrants from endemic countries ..... 6

Results ..... 6
Tables and Figures ..... 8
Appendix ..... 13
- Fit of the multi-state Markov model ..... 13
- Stan code for Bayesian multiparameter evidence synthesis ..... 14
- Multi-state Markov model ..... 18

References ..... 26

# INTRODUCTION

Patients with chronic hepatitis C (HCV) infection often remain asymptomatic for decades and even for life, but a proportion of them develop active chronic hepatitis, which is a progressive disease (Thein et al. 2008). Because of the largely asymptomatic nature of HCV infection, notification data reflect national screening and testing practices and do not give accurate insights into the prevalence of HCV infection. Although there are studies that have estimated the prevalence of HCV antibodies (anti-HCV) in specific groups such as the general population, first-time blood donors, or people who inject drugs (PWID) at drug treatment centers, those alone cannot be combined to produce national estimates unless some additional information regarding the composition of each HCV risk group and its prevalence in the population are known (Sweeting et al. 2008; McDonald et al. 2014; Tan et al. 2018).

# METHODS

Bayesian multiparameter evidence synthesis (MPES) has been a popular approach to formally estimate anti-HCV or HIV prevalence (Sweeting et al. 2008; McDonald et al. 2014; Tan et al. 2018; Presanis et al. 2010; Veen et al. 2011). In this project, we extend this method to estimating the CHC prevalence, with the population (15-79 years) split into three main non-overlapping risk groups, i.e., recent PWID (those who have injected in the last year), ex-PWID, and non-PWID. A unified model is assumed including parameters associated with the CHC prevalence of recent, ex-PWID, and non-PWID (denoted by $\pi_{rec}$, $\pi_{ex}$, and $\pi_{non}$, respectively) and the prevalence of these risk groups in the population (denoted by $\rho_{rec}$, $\rho_{ex}$, and $\rho_{non}$, respectively; $\rho_{rec} + \rho_{ex} + \rho_{non} = 1$). To estimate the CHC prevalence in the whole population, $\pi$, we used the formula:

$$\pi = \pi_{rec}\rho_{rec} + \pi_{ex}\rho_{ex} + \pi_{non}\rho_{non} \quad (1)$$

Bayesian synthesis considers all available data, allowing for an evidence-based estimation of CHC prevalence in the population, with inherent uncertainty properly accounted for. Our aim is to estimate the CHC prevalence for the total (15-79) population of Austria in 2019 using sources of information over the last decade (2010-2019).

## Prevalence of ex-PWID

Data on the prevalence of ex-PWID are sparse and generally often unreliable. To overcome this limitation, we apply the method proposed by McDonald et al. (2014), which requires simulating a multi-state Markov model representing the non-PWID, recent PWID, and ex-PWID. In the multi-state Markov model, the simulation starts in 1950 and examines the population aged 15-79 years old. A certain number of 15-year-old individuals enter the simulation process each year. These numbers of 15-year-old individuals are obtained from the United Nations Statistic Division UNSD, being different for each country. The ageing of the population is also taken into account. Country-specific overall mortality data are retrieved from the Life Tables of the World Health Organization WHO. The duration of injecting career is also country-specific and obtained from the paper of Hines et al. (2020).

To estimate the number of ex-PWID, the model can be calibrated on the number of recent PWID provided in the EMCDDA barometer or the EMCDDA statistical bulletin (the code of the Markov model can be found in the Appendix). If there are no available data in the barometer, estimates from the systematic review of Grebely et al. (2019) could be used. Alternatively, if the national focal points suggest or provide different and updated or more accurate data for calibration purposes, we will consider their advice and adjust the model accordingly.

After applying the model for Austria, we compute the number (and the corresponding Confidence Interval - CI) of ex-PWID in 2019. The number of ex-PWID is then divided by the population size (15-79 years) in the same year, and a Normal distribution, constrained such that $\rho_{ex} \in (0,1)$, is assumed for $\rho_{ex}$. The standard deviation of this distribution is specified to approximately correspond to the respective CI, i.e. $\left(UB_{\rho_{ex}} - LB_{\rho_{ex}}\right)/(2 \times 1.96)$, where $UB_{\rho_{ex}}$ and $LB_{\rho_{ex}}$ denote the upper and lower limit of the CI.

## Prevalence of recent PWID

The number of recent PWID in Austria in 2019 is also projected by the multi-state Markov model described in the previous subsection. To estimate $\rho_{rec}$, the number of recent PWID is divided by the population size (15-79 years). Similar to the previous subsection, a Normal distribution, constrained such that $\rho_{rec} \in (0,1)$, is assumed for $\rho_{rec}$, with the standard deviation specified to correspond to the CI obtained from the multi-state Markov model.

## Prevalence of CHC among recent PWID

The prevalence (and the 95% CI) of CHC among recent PWID can be informed from studies in the EMCDDA statistical bulletin. If information on the CHC prevalence is available, the Binomial distribution in the model to inform $\pi_{rec}$ was used. However, as these EMCDDA data typically refer to the anti-HCV prevalence and not to the CHC prevalence, they could not be used directly. This issue is addressed by noting that spontaneous viral clearance occurs in approximately one in four people with acute hepatitis C (Micallef, Kaldor, and Dore 2006). To account for the variability of the HCV clearance probabilities, we used the result reported in Micallef, Kaldor, and Dore (2006), i.e., the proportion of HCV clearance (denoted by $\rho_{clear}$) is equal to 0.26 (95% CI 0.22–0.29). Thus, estimates of the CHC prevalence among recent PWID ($\pi_{rec}$) can be obtained using the formula $\pi_{rec} = \pi(\text{anti-HCV})_{rec}(1-\rho_{clear})$, where $\pi(\text{anti-HCV})_{rec}$ denotes the anti-HCV prevalence among recent PWID.

Apart from spontaneous clearance, some people may have been treated with direct acting antivirals (DAAs). Since treatment data may be weak and unstandardised or unavailable for most EU/EEA countries, we have not formally taken treatment into account.

If information on the HCV prevalence (CHC or anti-HCV) among recent PWID in the EMCDDA database is not available, the required information is obtained from the paper of Grebely et al. (2019). Currently, nationwide anti-HCV prevalence data on current PWID in 2019, reported in the EMCDDA statistical bulletin, were used. If the national focal point recommends updated formal estimates, the model input could be adjusted accordingly.

## Prevalence of CHC among ex-PWID

Information on CHC prevalence among ex-PWID (i.e., $\pi_{ex}$) is difficult to obtain directly. To overcome this, nationwide data on the HCV prevalence among ever users through the EMCDDA database was utilized. However, if these data refer to the anti-HCV prevalence, they are adjusted according to the procedure described in the previous subsection, i.e. $\pi_{ever} = \pi(\text{anti-HCV})_{ever}(1-\rho_{clear})$, where $\pi(\text{anti-HCV})_{ever}$ denotes the anti-HCV prevalence among ever PWID. In any case, if an estimate of $\pi_{ever}$ is available, $\pi_{ex}$ can be indirectly estimated since $\pi_{ever}$ is equal to a weighted average of $\pi_{rec}$ and $\pi_{ex}$, as shown by the following formula

$$\pi_{ever} = \frac{\rho_{rec}}{\rho_{rec} + \rho_{ex}} \pi_{rec} + \frac{\rho_{ex}}{\rho_{rec} + \rho_{ex}} \pi_{ex}, \quad (2)$$

or, equivalently,

$$\pi_{ex} = \left( \pi_{ever} - \frac{\rho_{rec}}{\rho_{rec} + \rho_{ex}} \pi_{rec} \right) \times \frac{\rho_{rec} + \rho_{ex}}{\rho_{ex}}, \quad (3)$$

Currently, CHC prevalence data from ever PWID in Vienna in 2020, obtained through personal communication from EMCDDA, were used. In any case, the model could be updated with any other relevant study/information suggested by the national focal point.

## Prevalence of CHC among non-PWID

To estimate the CHC prevalence among non-PWID in a country (after 2010), the ECDC database (European Centre for Disease Prevention and Control (ECDC) 2021) was used. The ECDC group has carefully and critically collected information from relevant studies across the EU/EEA region in cooperation with the focal points of each country. On the basis of the representativeness of each study and other factors (European Centre for Disease Prevention and Control (ECDC) 2016), a risk of bias score was assigned to each study, with higher values indicating higher-quality studies. When higher-quality studies are available in a country (≥ 4), they are used to estimate the overall CHC prevalence in that country among non-PWID. If there are no higher-quality studies, general population estimates with a lower quality (<4) are pooled. If no general population prevalence estimates are available, data on the prevalence of HCV in pregnant women could be used as a prevalence measure. Finally, if data on pregnant women are lacking too, first-time blood donor studies could be an option. However, if the national focal point agrees or recommends, estimates from a neighboring country could be used instead, or we could use any other relevant information provided/suggested by the national focal point.

We directly use CHC prevalence data from studies in the general population, if available. If the data in the general population refer to anti-HCV prevalence (without any data on the viremic population), we adjust the estimates to get CHC prevalence based on the spontaneous HCV clearance estimate of 26%, described in the previous subsection.

As $\rho_{rec}$ and $\rho_{ex}$ are likely to be low, $\pi_{non}$ will be the most critical factor for the estimation of the overall CHC prevalence in the population. To adjust for potential heterogeneity between the CHC prevalence estimates from different studies in the non-PWID population in a country, as a sensitivity analysis, we also apply a random-effect meta-analytic approach to pool the CHC prevalence across studies on non-PWID (Lin and Chu 2020). The random-effect approach is carried out only when at least 3 studies are available in the general population of a certain country, and the estimates are compared with the corresponding estimates from the approach assuming no heterogeneity (similar to a fixed-effect meta-analysis). If the estimates are inconsistent, possible explanations are investigated and discussed. If there is substantial heterogeneity between studies, it would help more to explore the causes; for example, following national focal point suggestions, some studies could be excluded to reduce heterogeneity.

### Sensitivity analysis including migrants from endemic countries

A sensitivity analysis including data for the viremic population among migrants based on the report of the ECDC group (European Centre for Disease Prevention and Control 2016) was carried out. Migrants from endemic countries represent 12.2% of the adult population in Austria (Table 8 in European Centre for Disease Prevention and Control (2016)), with the respective CHC prevalence being equal to $\pi_{mig} = 1.3\%$ (Table 9 in European Centre for Disease Prevention and Control (2016)). The overall CHC prevalence is now estimated by

$$\pi = \pi_{rec}\rho_{rec} + \pi_{ex}\rho_{ex} + \pi_{mig}\rho_{mig} + \pi_{non}\rho_{non},$$

where $\rho_{rec} + \rho_{ex} + \rho_{mig} + \rho_{non} = 1$.

## RESULTS

The above-mentioned approach was used to estimate the prevalence of CHC in 2019 (considering ages between 15-79 years: 7,137,566).

The aggregated data used by our approach are briefly presented in Table 1. The multi-state Markov model was calibrated based on information on the number of recent PWID in 2019 provided by the national contact points. In Austria, there were 4 studies on first-time blood donors, which included only anti-HCV data. Actually, there was also a low-quality study including pregnant women (Diab-Elschahawi et al. 2013), which was rather old recruiting

individuals before 2010. Therefore, it was not considered in this analysis. To estimate the CHC prevalence in the general population (primarily non-PWID), we used a fixed-effect meta-analytic approach, with the corresponding results presented in Table 2. The prevalence of recent and ex-PWID was low in Austria (about 0.17% and 0.47%, respectively) corresponding to 12,250 (95% CI: 11,325-12,965) recent PWID and 33,330 (95% CI: 32,085-34,510) ex-PWID in the population. However, the CHC prevalence in these groups was substantial (Table 2) being 19.73% and 59.9%, respectively. This translates to 2,415 (95% CI: 1,529-3,496) and 19,967 (95% CI: 16,898-23,066) recent and ex-PWID aged 15-79 living with CHC infection in Austria in 2019. The CHC prevalence in the general population was 0.02% (95% CI: 0.01%-0.03%), much lower than that of the high-risk groups. Taking all pieces of information into account, the overall CHC prevalence in Austria in 2019 was equal to 0.33% (95% CI: 0.29%-0.38%), which corresponds to 23,860 (95% CI: 20,931-26,854) individuals aged 15-79 years with CHC infection.

The corresponding results under a random-effect meta-analysis for the studies in the general population were very similar and are provided in Table 3.

The results from our model including migrants from endemic countries as a separate group are presented in Table 4. However, this analysis comes with possible limitations; that is, including migrants as a separate group is valid only if migrants do not overlap with the remaining groups (recent PWID, ex-PWID, and non-PWID). Thus, if migrants do participate proportionally in the study(ies) in the general population, this analysis may result in biased overall CHC estimates (most probably in higher CHC prevalence estimates). However, if the national focal points consider that including migrants as a separate group is valid, we could consider results in Table 4 as the main analysis.

The proportion of CHC cases attributed to injection drug use was estimated to be around 93.95%, which was considered to be relatively high by the national contact points. However, since there was no recent study in the general population in Austria, we relied on data from first-time blood donors. Therefore, the CHC prevalence in the general population may be underestimated, which in turn can lead to an overestimation of the proportion of CHC prevalence that is attributed to injection drug use.

## TABLES AND FIGURES

*Table 1. Data (real) contributing to the estimation of CHC prevalence in Austria in 2019.*

| Parameter | Number (95%CI) | Numerator | Denominator | Notes | Year of study |
|---|---|---|---|---|---|
| $\rho_{rec}$ | 12,250 (11,325-12,965) | | | Method based on McDonald et al. | 2019 |
| $\rho_{ex}$ | 33,330 (32,085-34,510) | | | Method based on McDonald et al. | 2019 |
| $\pi(\text{anti-HCV})_{rec}$ | | 17 | 65 | EMCDDA database | 2019 |
| $\pi_{ever}$ | | 121 | 246 | EMCDDA email | 2020 |
| $\pi(\text{anti-HCV})_{non}$ | | 18 | 46,603 | ECDC database (NA et al.); Risk of bias=NA | 2010 |
| $\pi(\text{anti-HCV})_{non}$ | | 2 | 17,899 | ECDC database (NA et al.); Risk of bias=NA | 2014 |
| $\pi(\text{anti-HCV})_{non}$ | | 7 | 38,369 | ECDC database (NA et al.); Risk of bias=NA | 2015 |
| $\pi(\text{anti-HCV})_{non}$ | | 10 | 34,420 | ECDC database (NA et al.); Risk of bias=NA | 2016 |

*Notes*: Higher risk of bias score denotes a higher-quality study (range from 0 to 6); † After excluding PWID from the study.

*Table 2. Results from the method assuming no heterogeneity in the estimation of the CHC prevalence in the general population from different studies (i.e., similar to a fixed-effect meta-analysis).*

| Parameter | Posterior median | Posterior sd | LB (2.5%) | UB (97.5%) |
|---|---|---|---|---|
| $\rho_{rec}$ (%) | 0.17 | 0.01 | 0.16 | 0.18 |
| $\rho_{ex}$ (%) | 0.47 | 0.01 | 0.45 | 0.48 |
| $\pi_{rec}$ (%) | 19.73 | 4.05 | 12.54 | 28.4 |
| $\pi_{ex}$ (%) | 59.9 | 4.64 | 50.89 | 69.05 |
| $\pi_{non}$ (%) | 0.02 | 0 | 0.01 | 0.03 |
| $\pi$ (%) | 0.33 | 0.02 | 0.29 | 0.38 |
| Number with CHC | 23,860 | 1,524 | 20,931 | 26,854 |
| Pr(Ever PWID\|CHC) (%) | 93.95 | 1.02 | 91.72 | 95.72 |
| Pr(Non-PWID\|CHC) (%) | 6.05 | 1.02 | 4.28 | 8.28 |

*Notes*: $\rho_{rec}$, prevalence of recent PWID; $\rho_{ex}$, prevalence of ex-PWID; $\pi_{rec}$, CHC prevalence among recent PWID; $\pi_{ex}$, CHC prevalence among ex-PWID; $\pi_{non}$, CHC prevalence among non-PWID; $\pi$ overall CHC prevalence in Austria; LB, Lower Boundary; UP, Upper Boundary

*Table 3. Results from the method assuming heterogeneity in the estimation of the CHC prevalence in the general population from different studies (i.e., similar to a random-effect meta-analysis).*

| Parameter | Posterior median | Posterior sd | LB (2.5%) | UB (97.5%) |
|---|---|---|---|---|
| $\rho_{rec}$ (%) | 0.17 | 0.01 | 0.16 | 0.18 |
| $\rho_{ex}$ (%) | 0.47 | 0.01 | 0.45 | 0.48 |
| $\pi_{rec}$ (%) | 19.67 | 4.03 | 12.52 | 28.28 |
| $\pi_{ex}$ (%) | 59.91 | 4.67 | 50.66 | 69.02 |
| $\pi_{non}$ (%) | 0.02 | 0.02 | 0 | 0.05 |
| $\pi$ (%) | 0.33 | 0.03 | 0.29 | 0.38 |
| Number with CHC | 23,704 | 2,314 | 20,531 | 27,448 |
| Pr(Ever PWID\|CHC) (%) | 94.94 | 3.82 | 85.46 | 98.61 |
| Pr(Non-PWID\|CHC) (%) | 5.06 | 3.82 | 1.39 | 14.54 |
| Between-study variance | 0.68 | 1.74 | 0.04 | 5.87 |

*Notes*: $\rho_{rec}$, prevalence of recent PWID; $\rho_{ex}$, prevalence of ex-PWID; $\pi_{rec}$, CHC prevalence among recent PWID; $\pi_{ex}$, CHC prevalence among ex-PWID; $\pi_{non}$, CHC prevalence among non-PWID; $\pi$ overall CHC prevalence in Austria; LB, Lower Boundary; UP, Upper Boundary

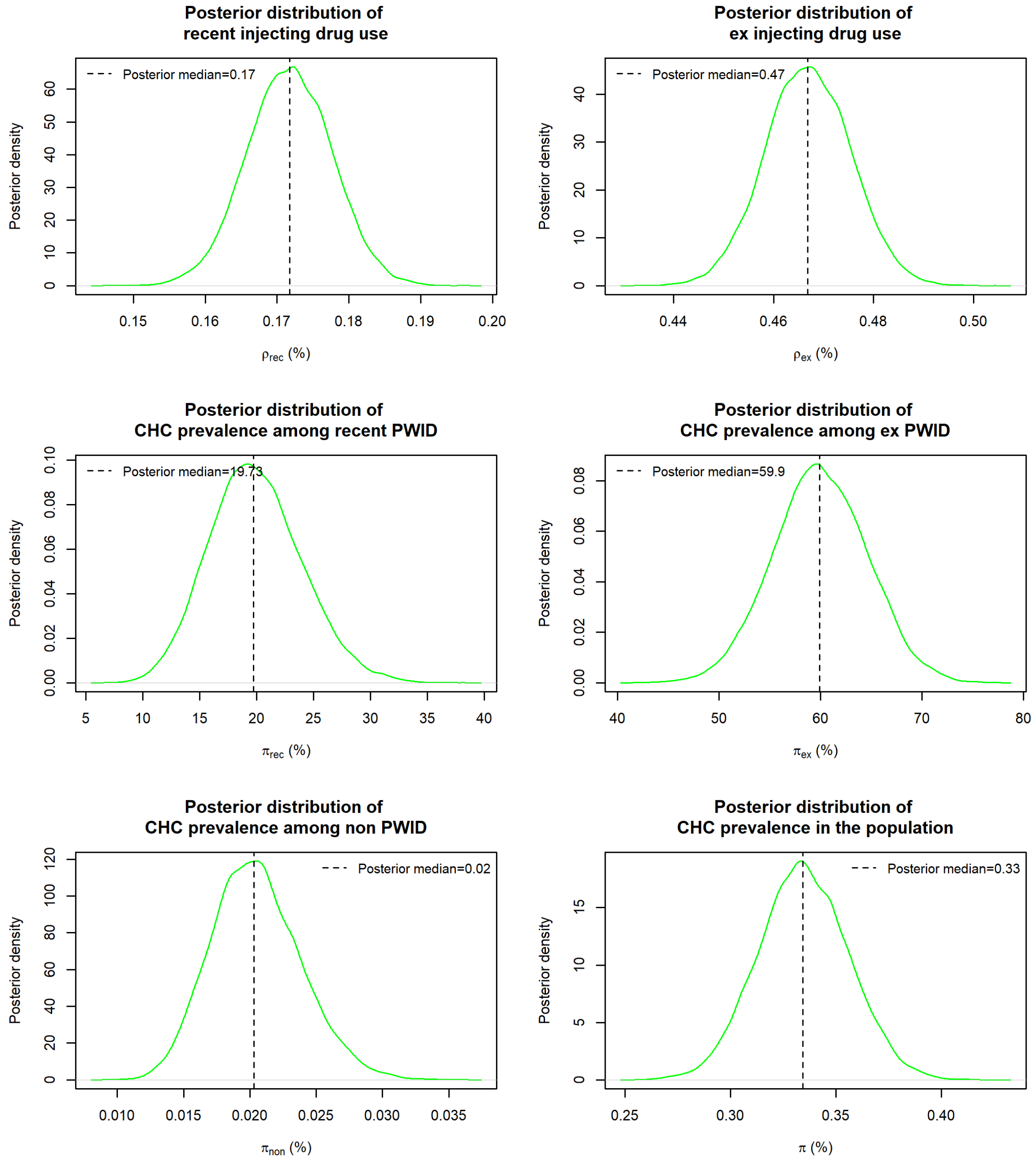


*Figure 1. Posterior distributions of the key model parameters using the approach that assumes no heterogeneity in the estimation of the CHC prevalence in the general population (primarily non-PWID) from different studies (i.e., similar to a fixed-effect meta-analysis).*

*Table 4. Results from the method assuming no heterogeneity in the estimation of the CHC prevalence in the general population (primarily non-PWID) from different studies (i.e., similar to a fixed-effect meta-analysis) including migrants from endemic countries as a separate group.*

| Parameter | Posterior median | Posterior sd | LB (2.5%) | UB (97.5%) |
|---|---|---|---|---|
| $\rho_{rec}$ (%) | 0.17 | 0.01 | 0.16 | 0.18 |
| $\rho_{ex}$ (%) | 0.47 | 0.01 | 0.45 | 0.48 |
| $\rho_{mig}$ (%) | 12.2 | 0 | 12.2 | 12.2 |
| $\pi_{rec}$ (%) | 19.76 | 4 | 12.6 | 28.19 |
| $\pi_{ex}$ (%) | 59.93 | 4.74 | 50.75 | 69.3 |
| $\pi_{mig}$ (%) | 1.3 | 0.2 | 0.91 | 1.69 |
| $\pi_{non}$ (%) | 0.02 | 0 | 0.01 | 0.03 |
| $\pi$ (%) | 0.49 | 0.03 | 0.43 | 0.56 |
| Number with CHC | 35,037 | 2,342 | 30,443 | 39,661 |
| Pr(Ever PWID\|CHC) (%) | 63.99 | 3.59 | 57.32 | 71.35 |
| Pr(Mig\|CHC) (%) | 32.39 | 3.68 | 24.73 | 39.15 |
| Pr(Non-PWID\|CHC) (%) | 3.62 | 0.62 | 2.55 | 4.97 |

*Notes*: $\rho_{rec}$, prevalence of recent PWID; $\rho_{ex}$, prevalence of ex-PWID; $\rho_{mig}$, prevalence of migrants from endemic countries; $\pi_{rec}$, CHC prevalence among recent PWID; $\pi_{ex}$, CHC prevalence among ex-PWID; $\pi_{mig}$, CHC prevalence among migrants from endemic countries; $\pi_{non}$, CHC prevalence among non-PWID; $\pi$ overall CHC prevalence in Austria; LB, Lower Boundary; UP, Upper Boundary

# APPENDIX

## Fit of the multi-state Markov model

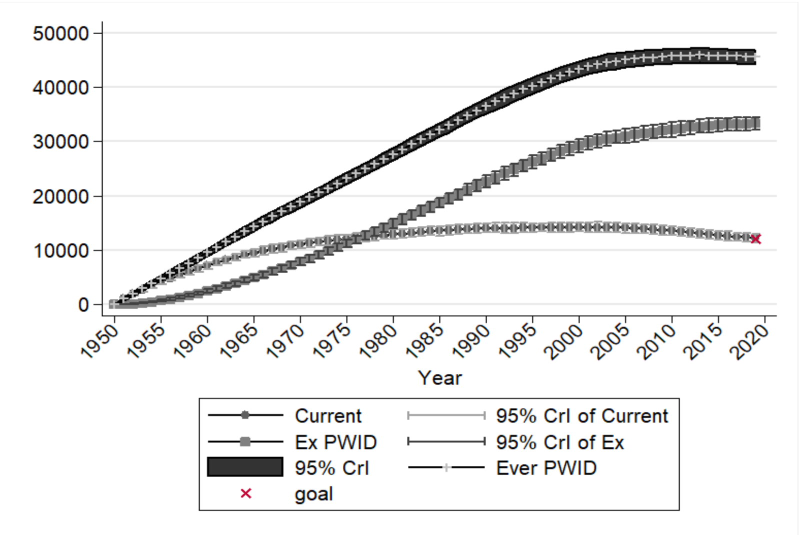


*Figure 2: Model predictions for the numbers of current and ex-PWID (people who inject drugs). The solid black line and shaded grey error bars show the median and 95% credible intervals (95% CrI) for the model projections. For comparison, asterisks indicate the observed country's data.*

## Stan code for Bayesian multiparameter evidence synthesis

```
data {
  int<lower=0> N1579; // Population of 15-79 in `Country`
  int<lower=1> Knon; // Number of studies in the ECDC data for `Country`
  int<lower=1> Kever; // Number of studies in the EMCDDA data for ever users in `Country`
  int<lower=1> Kcur; // Number of studies in the EMCDDA barometer (for HCV prevalence of PWID) in `Country`

  real p_cur_mean; // Prior mean for the prevalence of current use in `Country`
  real<lower=0> p_cur_sd; // Prior sd for the prevalence of current use in `Country`

  real p_ex_mean; // Prior mean for the prevalence of ex-use in `Country`
  real<lower=0> p_ex_sd; // Prior sd for the prevalence of ex-use in `Country`

  int<lower=0> Nst_hcv_cur[Kcur]; // Number of individuals in the study estimating anti-HCV among recent PWID
  int<lower=0> Yst_hcv_cur[Kcur]; // Number of HCV+ in the study estimating anti-HCV among recent PWID

  int<lower=0> Nst_CHC_ever[Kever]; // Number of individuals in the study estimating CHC among ever IDU
  int<lower=0> Yst_CHC_ever[Kever]; // Number of HCV+ in the study estimating CHC among ever PWID

  int<lower=0> Nst_hcv_non[Knon]; // Number of individuals in the study estimating anti-HCV among non PWID
  int<lower=0> Yst_hcv_non[Knon]; // Number of HCV+ in the study estimating anti-HCV among non PWID
```

```
  vector<lower=0>[3] alpha; // parameter of the Diriclet prior

  real HCVclear_mean; // Prior mean for the HCV clearance probability
  real<lower=0> HCVclear_sd; // Prior sd for the HCV clearance probabilit
y
}

// Block defining the original parameters
parameters {
  // The parameters to be sampled
  simplex[3] rho; // Prevalence of the three risk groups
  real<lower=0,upper=1> CHCpi_cur; // P(CHC+|Cur)
  real<lower=0,upper=1> CHCpi_ex; // P(CHC+|Ex)
  real<lower=0,upper=1> CHCpi_non; // P(CHC+|Non)

  real<lower=0,upper=1-CHCpi_cur> HCVclear; // Probability of HCV clearan
ce; upper bound = 1-prevalence of chronic HCV
}



transformed parameters {
  // Change scales
  real<lower=0,upper=1> rho_ever;
  real<lower=0,upper=1> CHCpi_ever;
  real<lower=0,upper=1> pi_non;
  real<lower=0,upper=1> pi_cur;

  rho_ever = rho[1] + rho[2];
  CHCpi_ever = CHCpi_cur*rho[1]/rho_ever + CHCpi_ex*rho[2]/rho_ever;
  pi_non = CHCpi_non/(1-HCVclear);
  pi_cur = CHCpi_cur/(1-HCVclear);
```

```
}



// Binomial regression model
model {
  // Priors
  rho ~ dirichlet(alpha);

  ////////////////////////////////
  // Likelihood contributions //
  ////////////////////////////////

  // Probability of HCV clearance
  HCVclear ~ normal(HCVclear_mean,HCVclear_sd);

  // Prevalence of current use
  rho[1] ~ normal(p_cur_mean,p_cur_sd);

  // Prevalence of ex-use
  rho[2] ~ normal(p_ex_mean,p_ex_sd);

  // Prevalence of chronic HCV among current users
  Yst_hcv_cur ~ binomial(Nst_hcv_cur,pi_cur);

  // Prevalence of HCV among ever users
  Yst_CHC_ever ~ binomial(Nst_CHC_ever,CHCpi_ever);

  // HCV+ among non
  Yst_hcv_non ~ binomial(Nst_hcv_non,pi_non);
}
```

```
generated quantities {
  // Functions of parameters
  real<lower=0,upper=100> overalCHC;
  real logit_CHCpi_cur;
  real logit_CHCpi_ex;
  real logit_CHCpi_non;
  real logit_rho_cur;
  real logit_rho_ex;
  real logit_rho_non;
  real<lower=0,upper=1> pEverGivenCHC;
  real<lower=0,upper=1> pNonGivenCHC;
  real logit_HCVclear;
  real<lower=0> NumberCHC;

  // Overall HCV prevalence
  overalCHC = 100*(rho[1]*CHCpi_cur + rho[2]*CHCpi_ex +  rho[3]*CHCpi_non
);
  pEverGivenCHC = CHCpi_ever*rho_ever/(overalCHC/100);
  pNonGivenCHC = CHCpi_non*(1-rho_ever)/(overalCHC/100);
  NumberCHC = round(overalCHC*N1579/100);

  logit_rho_cur = logit(rho[1]);
  logit_rho_ex = logit(rho[2]);
  logit_rho_non = logit(rho[3]);
  logit_CHCpi_cur = logit(CHCpi_cur);
  logit_CHCpi_ex = logit(CHCpi_ex);
  logit_CHCpi_non = logit(CHCpi_non);
  logit_HCVclear = logit(HCVclear);
}
```

## Multi-state Markov model

```
#include <stdio.h>
#include <stdlib.h>
#include <string.h>
#include <math.h>
#include <time.h>

#define loops 100
int populationPerYearAndAge[2021][66];
double deathRatePerAge[66];
int totalPopulationPerYear[2021];

FILE *F_DeathRate=fopen("`Country`-deathRates.txt","r");
FILE *F_Population=fopen("`Country`-population.txt","r");
FILE *out;

double pa_start = 0.00012;                    // Setting Variable
double pa_step  = 0.000002;                   // Setting Variable
double pa_stop  = 0.000123;                   // Setting Variable

double pa;
double pg = 1.0/11.7;   // Assumed average active injecting career of  11
.7 years
double pk = 0.004*12.0;     // Relapse rate of 0.004/month

struct people{
    int age;
    int state;
};

char filename[100];
struct people person[50000000];
```

```
long int totalPersons=0;

void setPopulationAge()
{
    for( int j=0; j<71; j++){
        for( int i=0; i<51; i++){
            fscanf(F_Population,"%d",&populationPerYearAndAge[1950+j][15+
i]);
        }
    }
}

void setDeathRate()
{
    for( int i=0; i<51; i++){
        fscanf(F_DeathRate,"%lf",&deathRatePerAge[15+i*5]);
        for( int j=0; j<5; j++){
            deathRatePerAge[15+i*5+j] = deathRatePerAge[15+i*5];
        }
    }
}

void getTotalPopulationPerAge()
{
    for( int j=0; j<71; j++){
        for( int i=0; i<51; i++){
            totalPopulationPerYear[1950+j] += populationPerYearAndAge[195
0+j][15+i];
        }
    }
}
```

```
void printTotalPopulationPerAge()
{
    printf("---- - ----------\n");
    printf("Year - Population\n");
    printf("---- - ----------\n");
    for( int i=1950; i<2021; i++){
        printf("%d - %d\n",i,totalPopulationPerYear[i]);
    }
}

void printDeathRatePerAge()
{
    printf("--- - -----\n");
    printf("Age - Rate\n");
    printf("--- - -----\n");
    for( int i=15; i<65; i++){
        printf("%d - %lf\n",i,deathRatePerAge[i]);
    }
}

void printTotalPersonPerState( int year)
{
    int count[4]= {0,0,0,0};
    for( int i=0; i<totalPersons; i++){
        count[person[i].state]++;
    }
    //printf("%d\t%ld\t%ld\t%ld\t%ld\n",year,count[0],count[1],count[2],c
ount[3]);
    if(year==2014)
        printf("%d\t%ld\t%ld\t%ld\t%ld\t%lf\n",year,count[0],count[1],cou
nt[2],count[3],pa);
    if( year>2009){
```

```
        fprintf(out, "%d\t%ld\t%ld\t%ld\t%ld\n",year,count[0],count[1],co
unt[2],count[3]);
    }
}

void initializePopulation()
{
    int cnt=0;
    for( int j=15; j<65; j++){
        for( int i=0; i<populationPerYearAndAge[1950][j]*100; i++){
            person[cnt].age=j;
            person[cnt].state=0;
            cnt++;
        }
    }
    totalPersons = cnt;
}

bool checkRate(double rate)
{
    if (rate <0.001){
        double k = rand()/(1.00+RAND_MAX);
        if(k<0.001){
            rate = rate*1000;
        }
        else{
            return false;
        }
    }
    double k = rand()/(1.00+RAND_MAX);
    if ( k < rate )
        return true;
```

```
    else
        return false;
}

void changeStatusAndAge()
{
    for( int i=0; i<totalPersons; i++){
        // change state
        double k = rand()/(1.00+RAND_MAX);
        if( person[i].state==0){
            if ( checkRate(pa) &&  person[i].age<40){
                person[i].state=1;
            }
        }
        else if( person[i].state==1){
            if ( k < pg ){
                person[i].state=2;
            }
        }
        else if( person[i].state==2){
            if ( k < pk ){
                person[i].state=1;
            }
        }
        // After their 49th year, all active PWID are assumed to cease in
jecting
        if(person[i].age>49 && person[i].state==1){
            person[i].state=2;
        }
        // After their 64th year, remove
        if(person[i].age>64){
            person[i].state=3;
```

```
        }
        // death rate depending on age and state
        k = rand()/(1.00+RAND_MAX);
        if( person[i].state==0){
            if ( k < deathRatePerAge[person[i].age] ){
                person[i].state=3;
            }
        }
        else if( person[i].state==1){
            if ( k < deathRatePerAge[person[i].age] * 13.0 ){
                person[i].state=3;
            }
        }
        else if( person[i].state==2){
            if ( k < deathRatePerAge[person[i].age] ){
                person[i].state=3;
            }
        }
        // increase age one year
        person[i].age++;
    }
}

void addNewPersons(int year)
{
    // add new person [15 years old and non PWID]
    int cnt=totalPersons;
    for( int i=0; i<populationPerYearAndAge[year+1][15]*100; i++){
// 100 instead of 1000 for the population to reduce execution time
        person[cnt].age=15;
        person[cnt].state=0;
        cnt++;
```

```
    }
    // increase the total number of population
    totalPersons = cnt;
}
int main()
{
    srand(time(NULL));

    setPopulationAge();
    setDeathRate();

    getTotalPopulationPerAge();
    pa = pa_start;
    while ( pa < pa_stop){

        snprintf(filename, 100, "result_%lf.txt",pa);
        out=fopen(filename,"w");

        for( int iter=0; iter<loops ;iter++){
            initializePopulation();
            for( int year=1950; year<2020; year++){
                printTotalPersonPerState(year);
                changeStatusAndAge();
                addNewPersons(year);
            }
        }

        fclose(out);
        pa = pa + pa_step;
    }
```

```
}
```

## REFERENCES

Diab-Elschahawi, Magda, Verena Dosch, Claudia Honsig, Birgit Jatzko, Luigi Segagni, Ojan Assadian, and Elisabeth Presterl. 2013. "Evaluation of a Universal Vs a Targeted Hepatitis c Virus Screening Strategy Among Pregnant Women at the Vienna University Hospital." *American Journal of Infection Control* 41 (5): 459–60. https://doi.org/10.1016/j.ajic.2012.06.003.

European Centre for Disease Prevention and Control. 2016. "Epidemiological Assessment of Hepatitis b and c Among Migrants in the EU/EEA." https://www.ecdc.europa.eu/sites/default/files/media/en/publications/Publications/epidemiological-assessment-hepatitis-B-and-C-among-migrants-EU-EEA.pdf.

European Centre for Disease Prevention and Control (ECDC). 2016. "Systematic Review on Hepatitis b and c Prevalence in the EU/EEA." https://www.ecdc.europa.eu/sites/default/files/media/en/publications/Publications/systematic-review-hepatitis-B-C-prevalence.pdf.

———. 2021. "Hepatitis c Prevalence Database." https://www.ecdc.europa.eu/en/all-topics-z/hepatitis-c/tools/hepatitis-c-prevalence-database.

Grebely, Jason, Sarah Larney, Amy Peacock, Samantha Colledge, Janni Leung, Matthew Hickman, Peter Vickerman, et al. 2019. "Global, Regional, and Country-Level Estimates of Hepatitis c Infection Among People Who Have Recently Injected Drugs." *Addiction* 114 (1): 150–66. https://doi.org/https://doi.org/10.1111/add.14393.

Hines, Lindsey A., Adam Trickey, Janni Leung, Sarah Larney, Amy Peacock, Louisa Degenhardt, Samantha Colledge, et al. 2020. "Associations Between National Development Indicators and the Age Profile of People Who Inject Drugs: Results from a Global Systematic Review and Meta-Analysis." *The Lancet Global Health* 8 (1): e76–91. https://doi.org/10.1016/S2214-109X(19)30462-0.

Lin, Lifeng, and Haitao Chu. 2020. "Meta-Analysis of Proportions Using Generalized Linear Mixed Models." *Epidemiology* 31 (5). https://journals.lww.com/epidem/Fulltext/2020/09000/Meta_analysis_of_Proportions_Using_Generalized.16.aspx.

McDonald, Scott A., Rosmawati Mohamed, Maznah Dahlui, Herlianna Naning, and Adeeba Kamarulzaman. 2014. “Bridging the Data Gaps in the Epidemiology of Hepatitis c Virus Infection in Malaysia Using Multi-Parameter Evidence Synthesis.” *BMC Infectious Diseases* 14 (1): 564. https://doi.org/10.1186/s12879-014-0564-6.

Micallef, J. M., J. M. Kaldor, and G. J. Dore. 2006. “Spontaneous Viral Clearance Following Acute Hepatitis c Infection: A Systematic Review of Longitudinal Studies.” *Journal of Viral Hepatitis* 13 (1): 34–41. https://doi.org/https://doi.org/10.1111/j.1365-2893.2005.00651.x.

Presanis, Anne M., O. Noel Gill, Timothy R. Chadborn, Caterina Hill, Vivian Hope, Louise Logan, Brian D. Rice, Valerie C. Delpech, A. E. Ades, and Daniela De Angelis. 2010. “Insights into the Rise in HIV Infections, 2001 to 2008: A Bayesian Synthesis of Prevalence Evidence.” *AIDS* 24 (18). https://journals.lww.com/aidsonline/Fulltext/2010/11270/Insights_into_the_rise_in_HIV_infections,_2001_to.12.aspx.

Sweeting, M. J., D. De Angelis, M. Hickman, and A. E. Ades. 2008. “Estimating hepatitis C prevalence in England and Wales by synthesizing evidence from multiple data sources. Assessing data conflict and model fit.” *Biostatistics* 9 (4): 715–34. https://doi.org/10.1093/biostatistics/kxn004.

Tan, Sarah, Susanna Makela, Daliah Heller, Kevin Konty, Sharon Balter, Tian Zheng, and James H. Stark. 2018. “A Bayesian Evidence Synthesis Approach to Estimate Disease Prevalence in Hard-to-Reach Populations: Hepatitis c in New York City.” *Epidemics* 23: 96–109. https://doi.org/https://doi.org/10.1016/j.epidem.2018.01.002.

Thein, Hla-Hla, Qilong Yi, Gregory J. Dore, and Murray D. Krahn. 2008. “Estimation of Stage-Specific Fibrosis Progression Rates in Chronic Hepatitis c Virus Infection: A Meta-Analysis and Meta-Regression.” *Hepatology* 48 (2): 418–31. https://doi.org/https://doi.org/10.1002/hep.22375.

Veen, Maaike G. van, Anne M. Presanis, Stefano Conti, Maria Xiridou, Annemarie R. Stengaard, Martin C. Donoghoe, Ard I. van Sighem, Marianne A. van der Sande, and Daniela De Angelis. 2011. “National Estimate of HIV Prevalence in the Netherlands: Comparison and Applicability of Different Estimation Tools.” *AIDS* 25 (2).

https://journals.lww.com/aidsonline/Fulltext/2011/01140/National_estimate_of_HIV_prevalence_in_the.14.aspx.

# Estimating the prevalence of chronic hepatitis C infection (CHC) in Belgium using Bayesian multiparameter evidence synthesis

14/04/2022

## Table of Contents


Introduction ........ 2
Methods ........ 2
- Prevalence of ex-PWID ........ 3
- Prevalence of recent PWID ........ 3
- Prevalence of CHC among recent PWID ........ 4
- Prevalence of CHC among ex-PWID ........ 5
- Prevalence of CHC among non-PWID ........ 5

Results ........ 6
Tables and Figures ........ 8
Appendix ........ 11
- Fit of the multi-state Markov model ........ 11
- Stan code for Bayesian multiparameter evidence synthesis ........ 12
- Multi-state Markov model ........ 17

References ........ 25

# INTRODUCTION

Patients with chronic hepatitis C (HCV) infection often remain asymptomatic for decades and even for life, but a proportion of them develop active chronic hepatitis, which is a progressive disease (Thein et al. 2008). Because of the largely asymptomatic nature of HCV infection, notification data reflect national screening and testing practices and do not give accurate insights into the prevalence of HCV infection. Although there are studies that have estimated the prevalence of HCV antibodies (anti-HCV) in specific groups such as the general population, first-time blood donors, or people who inject drugs (PWID) at drug treatment centers, those alone cannot be combined to produce national estimates unless some additional information regarding the composition of each HCV risk group and its prevalence in the population are known (Sweeting et al. 2008; McDonald et al. 2014; Tan et al. 2018).

# METHODS

Bayesian multiparameter evidence synthesis (MPES) has been a popular approach to formally estimate anti-HCV or HIV prevalence (Sweeting et al. 2008; McDonald et al. 2014; Tan et al. 2018; Presanis et al. 2010; Veen et al. 2011). In this project, we extend this method to estimating the CHC prevalence, with the population (15-79 years) split into three main non-overlapping risk groups, i.e., recent PWID (those who have injected in the last year), ex-PWID, and non-PWID. A unified model is assumed including parameters associated with the CHC prevalence of recent, ex-PWID, and non-PWID (denoted by $\pi_{rec}$, $\pi_{ex}$, and $\pi_{non}$, respectively) and the prevalence of these risk groups in the population (denoted by $\rho_{rec}$, $\rho_{ex}$, and $\rho_{non}$, respectively; $\rho_{rec} + \rho_{ex} + \rho_{non} = 1$). To estimate the CHC prevalence in the whole population, $\pi$, we used the formula:

$$\pi = \pi_{rec}\rho_{rec} + \pi_{ex}\rho_{ex} + \pi_{non}\rho_{non} \quad (1)$$

Bayesian synthesis considers all available data, allowing for an evidence-based estimation of CHC prevalence in the population, with inherent uncertainty properly accounted for. Our aim is to estimate the CHC prevalence for the total (15-79) population of Belgium in 2019 using sources of information over the last decade (2010-2019).

## Prevalence of ex-PWID

Data on the prevalence of ex-PWID are sparse and generally often unreliable. To overcome this limitation, we apply the method proposed by McDonald et al. (2014), which requires simulating a multi-state Markov model representing the non-PWID, recent PWID, and ex-PWID. In the multi-state Markov model, the simulation starts in 1950 and examines the population aged 15-79 years old. A certain number of 15-year-old individuals enter the simulation process each year. These numbers of 15-year-old individuals are obtained from the United Nations Statistic Division UNSD, being different for each country. The ageing of the population is also taken into account. Country-specific overall mortality data are retrieved from the Life Tables of the World Health Organization WHO. The duration of injecting career is also country-specific and obtained from the paper of Hines et al. (2020). The multi-state Markov model currently assumes no relapses to drug injection after the age of 55 years.

To estimate the number of ex-PWID, the model can be calibrated on the number of recent PWID provided in the EMCDDA barometer or the EMCDDA statistical bulletin (the code of the Markov model can be found in the Appendix). If there are no available data in the barometer, estimates from the systematic review of Grebely et al. (2019) could be used. Currently, the model was calibrated on the results reported in the study of Plettinckx et al. (2021), also available in the EMCDDA barometer.

After applying the model for Belgium, we compute the number (and the corresponding Confidence Interval - CI) of ex-PWID in 2019. The number of ex-PWID is then divided by the population size (15-79 years) in the same year, and a Normal distribution, constrained such that $\rho_{ex} \in (0,1)$, is assumed for $\rho_{ex}$. The standard deviation of this distribution is specified to approximately correspond to the respective CI, i.e. $\left(UB_{\rho_{ex}} - LB_{\rho_{ex}}\right)/(2 \times 1.96)$, where $UB_{\rho_{ex}}$ and $LB_{\rho_{ex}}$ denote the upper and lower limit of the CI.

## Prevalence of recent PWID

The number of recent PWID in Belgium in 2019 is also projected by the multi-state Markov model described in the previous subsection. To estimate $\rho_{rec}$, the number of recent PWID is divided by the population size (15-79 years). Similar to the previous subsection, a Normal

distribution, constrained such that $\rho_{rec} \in (0,1)$, is assumed for $\rho_{rec}$, with the standard deviation specified to correspond to the CI obtained from the multi-state Markov model.

## Prevalence of CHC among recent PWID

The prevalence (and the 95% CI) of CHC among recent PWID can be informed from studies in the EMCDDA statistical bulletin. If information on the CHC prevalence is available, the Binomial distribution in the model to inform $\pi_{rec}$ was used. However, as these EMCDDA data typically refer to the anti-HCV prevalence and not to the CHC prevalence, they could not be used directly. This issue is addressed by noting that spontaneous viral clearance occurs in approximately one in four people with acute hepatitis C (Micallef, Kaldor, and Dore 2006). To account for the variability of the HCV clearance probabilities, we used the result reported in Micallef, Kaldor, and Dore (2006), i.e., the proportion of HCV clearance (denoted by $\rho_{clear}$) is equal to 0.26 (95% CI: 0.22–0.29).

Apart from spontaneous clearance, some people may have been treated with direct acting antivirals (DAAs). Based on a recent respondent-driven sampling (RDS) study in Brussels (Van Baelen et al. 2020), the proportion of PWID reporting having received treatment after that they were tested for HCV and seemed infected is equal to 38.2% (21/55). Moreover, the sustained virologic response (SVR) among PWID is estimated to be 88% (95% CI: 80% to 93%) (Graf et al. 2019). Taking the variability of the above estimates into account, the CHC prevalence among recent PWID ($\pi_{rec}$) can be obtained using the formula

$$\pi_{rec} = \pi(\text{anti-HCV})_{rec}(1 - \rho_{clear})(1 - \rho_{trt} \times SVR)$$

where $\pi(\text{anti-HCV})_{rec}$ denotes the anti-HCV prevalence among recent PWID and $\rho_{trt}$ denotes the proportion of anti-HCV positive PWID having initiated treatment.

If information on the HCV prevalence (CHC or anti-HCV) among recent PWID in the EMCDDA database is not available, the required information is obtained from the paper of Grebely et al. (2019). Currently, anti-HCV prevalence data from the study of Van Baelen et al. (2020), available also in the EMCDDA statistical bulletin, were used.

## Prevalence of CHC among ex-PWID

Information on CHC prevalence among ex-PWID (i.e., $\pi_{ex}$) is difficult to obtain directly. To overcome this, nationwide data on HCV prevalence among ever users through the EMCDDA database was utilized. Once an estimate of $\pi_{ever}$ is available, $\pi_{ex}$ can be indirectly estimated since $\pi_{ever}$ is equal to a weighted average of $\pi_{rec}$ and $\pi_{ex}$, as shown by the following formula

$$\pi_{ever} = \frac{\rho_{rec}}{\rho_{rec} + \rho_{ex}} \pi_{rec} + \frac{\rho_{ex}}{\rho_{rec} + \rho_{ex}} \pi_{ex}, \quad (2)$$

or, equivalently,

$$\pi_{ex} = \left( \pi_{ever} - \frac{\rho_{rec}}{\rho_{rec} + \rho_{ex}} \pi_{rec} \right) \times \frac{\rho_{rec} + \rho_{ex}}{\rho_{ex}}, \quad (3)$$

Currently, CHC prevalence data have been provided from EMCDDA through personal communication.

## Prevalence of CHC among non-PWID

To estimate the CHC prevalence among non-PWID in a country (after 2010), the ECDC database (European Centre for Disease Prevention and Control (ECDC) 2021) was used. The ECDC group has carefully and critically collected information from relevant studies across the EU/EEA region in cooperation with the focal points of each country. On the basis of the representativeness of each study and other factors (European Centre for Disease Prevention and Control (ECDC) 2016), a risk of bias score was assigned to each study, with higher values indicating higher-quality studies. When higher-quality studies are available in a country (≥ 4), they are used to estimate the overall CHC prevalence in that country among non-PWID. If there are no higher-quality studies, general population estimates with a lower quality (<4) are pooled. If no general population prevalence estimates are available, data on the prevalence of HCV in pregnant women could be used as a prevalence measure. Finally, if data on pregnant women are lacking too, first-time blood donor studies could be an option. However, if the national focal point agrees or recommends, estimates from a neighboring country could be used instead, or we could use any other relevant information provided/suggested by the national focal point.

We directly use CHC prevalence data from studies in the general population, if available. If the data in the general population refer to anti-HCV prevalence (without any data on the viremic population), we adjust the estimates to get CHC prevalence based on the spontaneous HCV clearance estimate of 26%, described in the previous subsection.

As $\rho_{rec}$ and $\rho_{ex}$ are likely to be low, $\pi_{non}$ will be the most critical factor for the estimation of the overall CHC prevalence in the population. To adjust for potential heterogeneity between the CHC prevalence estimates from different studies in the non-PWID population in a country, as a sensitivity analysis, we also apply a random-effect meta-analytic approach to pool the CHC prevalence across studies on non-PWID (Lin and Chu 2020). The random-effect approach is carried out only when at least 3 studies are available in the general population of a certain country, and the estimates are compared with the corresponding estimates from the approach assuming no heterogeneity (similar to a fixed-effect meta-analysis). If the estimates are inconsistent, possible explanations are investigated and discussed. If there is substantial heterogeneity between studies, it would help more to explore the causes; for example, following national focal point suggestions, some studies could be excluded to reduce heterogeneity.

# RESULTS

The above-mentioned approach was used to estimate the prevalence of CHC in 2019 (considering ages between 15-79 years: 8,868,984).

The aggregated data used by our approach are briefly presented in Table 1. In Belgium, there was 1 study on non-PWID of high quality, which included CHC data (Litzroth et al. 2019). To estimate the CHC prevalence in the general population (primarily non-PWID), we used a fixed-effect meta-analytic approach, with the corresponding results presented in Table 2. The prevalence of recent and ex-PWID was low in Belgium (about 0.08% and 0.16%, respectively) corresponding to 7,160 (95% CI: 6,510-7,660) recent PWID and 14,050 (95% CI: 13,170-14,650) ex-PWID in the population. However, the CHC prevalence in these groups was substantial (Table 2) being 20.24% and 12.93%, respectively. This translates to 1,448 (95% CI: 1,089-1,857) and 1,815 (95% CI: 808-2,997) recent and ex-PWID aged 15-79 living with CHC infection in Belgium in 2019. The CHC prevalence in the general population was 0.15% (95% CI: 0.05%-0.32%), much lower than that of the high-risk groups. Taking all pieces of information into account, the overall CHC prevalence in Belgium in 2019 was equal to 0.18%

(95% CI: 0.09%-0.36%), which corresponds to 16,178 (95% CI: 7,737-31,562) individuals aged 15-79 years with CHC infection.

# TABLES AND FIGURES

*Table 1. Data (real) contributing to the estimation of CHC prevalence in Belgium in 2019.*

| Parameter | Estimate (95%CI) | Number (95%CI) | Numerator | Denominator | Notes | Year of study |
|---|---|---|---|---|---|---|
| $\rho_{rec}$ | | 7,160 (6,510-7,660) | | | Method based on McDonald et al. | 2019 |
| $\rho_{ex}$ | | 14,050 (13,170-14,650) | | | Method based on McDonald et al. | 2019 |
| $\pi(\text{anti-HCV})_{rec}$ | | | 69 | 168 | EMCDDA database | 2019 |
| $\pi_{ever}$ | | | 32 | 211 | EMCDDA email | 2019 |
| $\pi_{non}$ | | | 4 | 3,209 | ECDC database (Litzroth et al.); Risk of bias=4 | 2013 |

*Notes*: Higher risk of bias score denotes a higher-quality study (range from 0 to 6).

*Table 2. Results from the method assuming no heterogeneity in the estimation of the CHC prevalence in the general population from different studies (i.e., similar to a fixed-effect meta-analysis).*

| Parameter | Posterior median | Posterior sd | LB (2.5%) | UB (97.5%) |
|---|---|---|---|---|
| $\rho_{rec}$ (%) | 0.08 | 0 | 0.07 | 0.09 |
| $\rho_{ex}$ (%) | 0.16 | 0 | 0.15 | 0.17 |
| $\pi_{rec}$ (%) | 20.24 | 2.58 | 15.42 | 25.6 |
| $\pi_{ex}$ (%) | 12.93 | 3.98 | 5.75 | 21.25 |
| $\pi_{non}$ (%) | 0.15 | 0.07 | 0.05 | 0.32 |
| $\pi$ (%) | 0.18 | 0.07 | 0.09 | 0.36 |
| Number with CHC | 16,178 | 6,170 | 7,737 | 31,562 |
| Pr(Ever PWID\|CHC) (%) | 20.17 | 8.59 | 9.76 | 42.62 |
| Pr(Non-PWID\|CHC) (%) | 79.83 | 8.59 | 57.38 | 90.24 |

*Notes*: $\rho_{rec}$, prevalence of recent PWID; $\rho_{ex}$, prevalence of ex-PWID; $\pi_{rec}$, CHC prevalence among recent PWID; $\pi_{ex}$, CHC prevalence among ex-PWID; $\pi_{non}$, CHC prevalence among non-PWID; $\pi$ overall CHC prevalence in Belgium; LB, Lower Boundary; UP, Upper Boundary

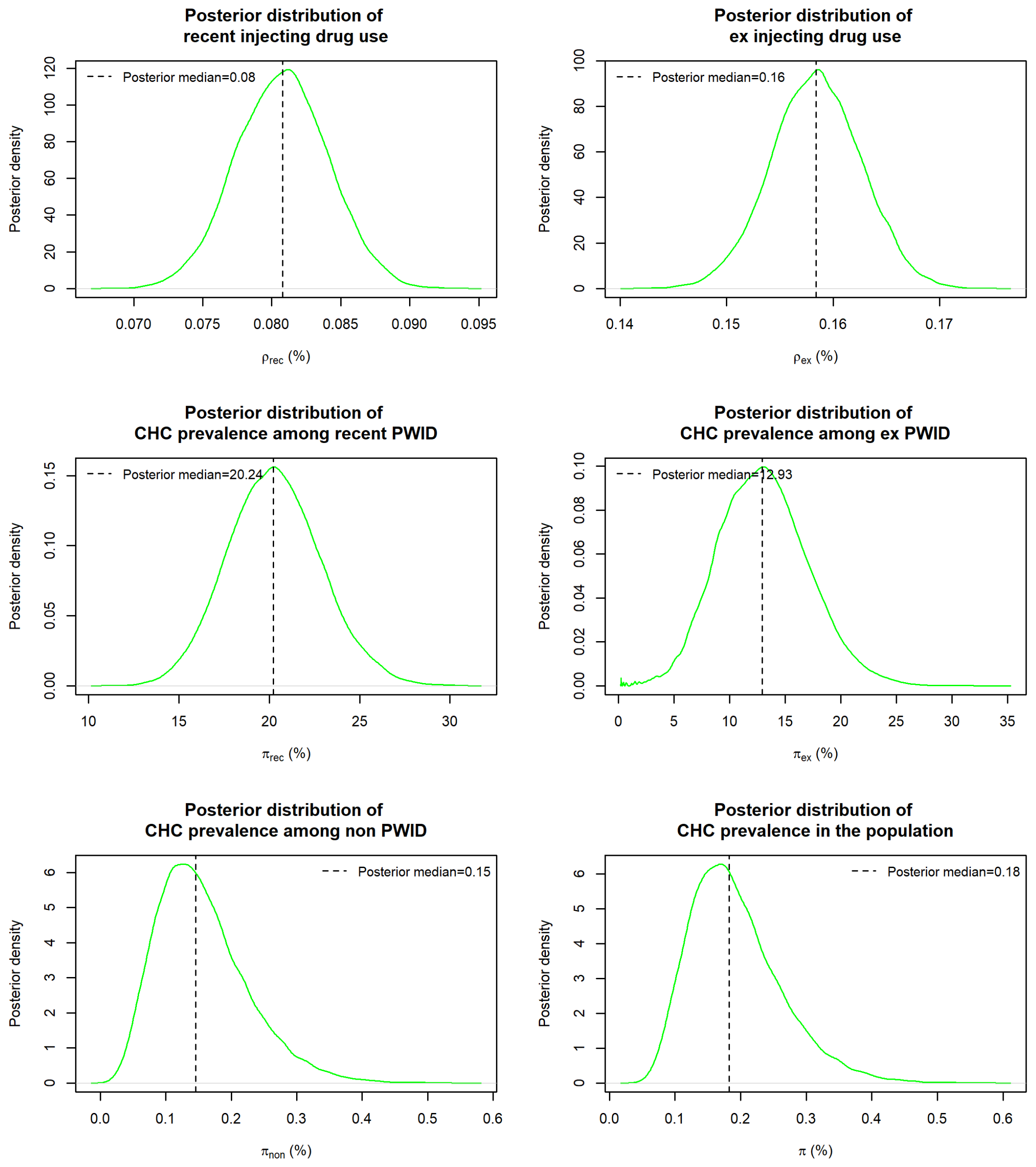


*Figure 1. Posterior distributions of the key model parameters using the approach that assumes no heterogeneity in the estimation of the CHC prevalence in the general population (primarily non-PWID) from different studies (i.e., similar to a fixed-effect meta-analysis).*

# APPENDIX

## Fit of the multi-state Markov model

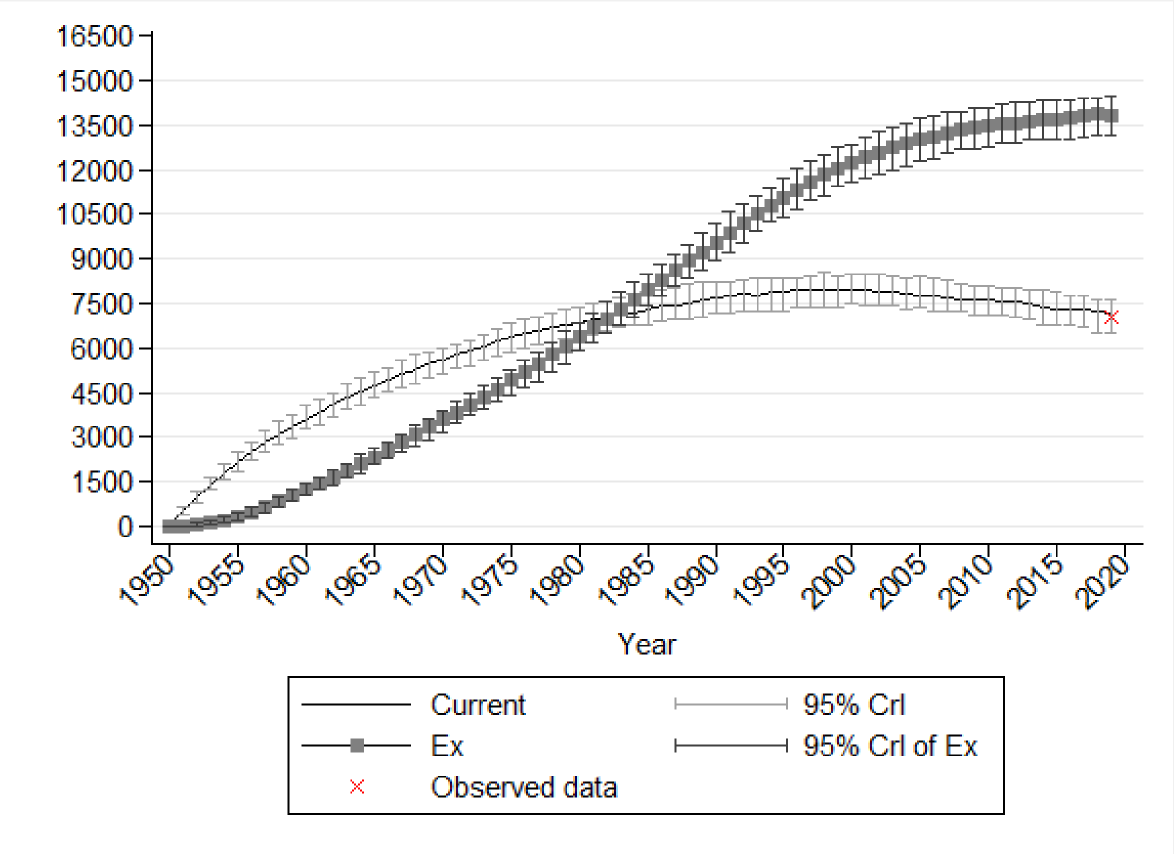


*Figure 2: Model predictions for the numbers of current and ex-PWID (people who inject drugs). The solid black line and shaded grey error bars show the median and 95% credible intervals (95% CrI) for the model projections. For comparison, asterisks indicate the observed country's data.*

## Stan code for Bayesian multiparameter evidence synthesis

```
data {
  int<lower=0> N1579; // Population of 15-79 in `Country`
  int<lower=1> Knon; // Number of studies in the ECDC data for `Country`
  int<lower=1> Kever; // Number of studies in the EMCDDA data for ever users in `Country`
  int<lower=1> Kcur; // Number of studies in the EMCDDA barometer (for HCV prevalence of PWID) in `Country`

  real p_cur_mean; // Prior mean for the prevalence of current use in `Country`
  real<lower=0> p_cur_sd; // Prior sd for the prevalence of current use in `Country`

  real p_ex_mean; // Prior mean for the prevalence of ex-use in `Country`
  real<lower=0> p_ex_sd; // Prior sd for the prevalence of ex-use in `Country`

  int<lower=0> Nst_TRT_cur; // Number of individuals in the study for TRT among current PWID
  int<lower=0> Yst_TRT_cur; // Number of individuals having received TRT among current PWID

  int<lower=0> Nst_hcv_cur[Kcur]; // Number of individuals in the study estimating anti-HCV among recent PWID
  int<lower=0> Yst_hcv_cur[Kcur]; // Number of HCV+ in the study estimating anti-HCV among recent PWID

  int<lower=0> Nst_CHC_ever[Kever]; // Number of individuals in the study estimating chronic HCV among ever IDU
  int<lower=0> Yst_CHC_ever[Kever]; // Number of HCV+ in the study estimating chronic HCV among ever PWID
```

```
  int<lower=0> Nst_CHC_non[Knon]; // Number of individuals in the study e
stimating chronic HCV among non PWID
  int<lower=0> Yst_CHC_non[Knon]; // Number of HCV+ in the study estimati
ng chronic HCV among non PWID

  vector<lower=0>[3] alpha; // parameter of the Diriclet prior

  real HCVclear_mean; // Prior mean for the HCV clearance probability
  real<lower=0> HCVclear_sd; // Prior sd for the HCV clearance probabilit
y

  real SVR_PWID_mean; // Prior mean for the SVR among PWID
  real<lower=0> SVR_PWID_sd; // Prior sd for the SVR among PWID
}

// Block defining the original parameters
parameters {
  // The parameters to be sampled
  simplex[3] rho; // Prevalence of the three risk groups
  real<lower=0,upper=1> CHCpi_cur; // P(CHC+|Cur)
  real<lower=0,upper=1> CHCpi_ex; // P(CHC+|Ex)
  real<lower=0,upper=1> CHCpi_non; // P(CHC+|Non)

  real<lower=0,upper=1> SVR_PWID;
  real<lower=0,upper=1> TRT_cur;
  real<lower=0,upper=1-CHCpi_cur/(1-SVR_PWID*TRT_cur)> HCVclear; // Proba
bility of HCV clearance; upper bound = 1-prevalence of chronic HCV
}


transformed parameters {
```

```
  // Change scales
  real<lower=0,upper=1> rho_ever;
  real<lower=0,upper=1> CHCpi_ever;
  real<lower=0,upper=1> pi_ever;
  real<lower=0,upper=1> pi_cur;

  rho_ever = rho[1] + rho[2];
  CHCpi_ever = CHCpi_cur*rho[1]/rho_ever + CHCpi_ex*rho[2]/rho_ever;
  pi_ever = CHCpi_ever/(1-HCVclear);
  pi_cur = CHCpi_cur/((1-HCVclear)*(1-TRT_cur*SVR_PWID));
}


// Binomial regression model
model {
  // Priors
  rho ~ dirichlet(alpha);

  ///////////////////////////////
  // Likelihood contributions //
  ///////////////////////////////

  // Probability of HCV clearance
  HCVclear ~ normal(HCVclear_mean,HCVclear_sd);

  // SVR among PWID
  SVR_PWID ~ normal(SVR_PWID_mean,SVR_PWID_sd);

  // TRT among current
  Yst_TRT_cur ~ binomial(Nst_TRT_cur,TRT_cur);

  // Prevalence of current use
```

```
  rho[1] ~ normal(p_cur_mean,p_cur_sd);

  // Prevalence of ex-use
  rho[2] ~ normal(p_ex_mean,p_ex_sd);

  // Prevalence of chronic HCV among current users
  Yst_hcv_cur ~ binomial(Nst_hcv_cur,pi_cur);

  // Prevalence of HCV among ever users
  Yst_CHC_ever ~ binomial(Nst_CHC_ever,CHCpi_ever);

  // HCV+ among non
  Yst_CHC_non ~ binomial(Nst_CHC_non,CHCpi_non);
}


generated quantities {
  // Functions of parameters
  real<lower=0,upper=100> overalCHC;
  real logit_CHCpi_cur;
  real logit_CHCpi_ex;
  real logit_CHCpi_non;
  real logit_rho_cur;
  real logit_rho_ex;
  real logit_rho_non;
  real<lower=0,upper=1> pEverGivenCHC;
  real<lower=0,upper=1> pNonGivenCHC;
  real logit_HCVclear;
  real<lower=0> NumberCHC;
  real logit_SVR_PWID;
  real logit_TRT_cur;
```

```
  // Overall HCV prevalence
  overalCHC = 100*(rho[1]*CHCpi_cur + rho[2]*CHCpi_ex +  rho[3]*CHCpi_non
);
  pEverGivenCHC = CHCpi_ever*rho_ever/(overalCHC/100);
  pNonGivenCHC = CHCpi_non*(1-rho_ever)/(overalCHC/100);
  NumberCHC = round(overalCHC*N1579/100);

  logit_rho_cur = logit(rho[1]);
  logit_rho_ex = logit(rho[2]);
  logit_rho_non = logit(rho[3]);
  logit_CHCpi_cur = logit(CHCpi_cur);
  logit_CHCpi_ex = logit(CHCpi_ex);
  logit_CHCpi_non = logit(CHCpi_non);
  logit_HCVclear = logit(HCVclear);
  logit_SVR_PWID = logit(SVR_PWID);
  logit_TRT_cur = logit(TRT_cur);
}
```

## Multi-state Markov model

```
#include <stdio.h>
#include <stdlib.h>
#include <string.h>
#include <math.h>
#include <time.h>

#define loops 100
int populationPerYearAndAge[2021][66];
double deathRatePerAge[66];
int totalPopulationPerYear[2021];

FILE *F_DeathRate=fopen("‘country’-deathRates.txt","r");
FILE *F_Population=fopen("‘country’-population.txt","r");
FILE *out;

double pa_start = 0.00012;                    // Setting Variable
double pa_step  = 0.000002;                   // Setting Variable
double pa_stop  = 0.000123;                   // Setting Variable

double pa;
double pg = 1.0/11.7;    // Assumed average active injecting career of  11
.7 years
double pk = 0.004*12.0;      // Relapse rate of 0.004/month

struct people{
    int age;
    int state;
};

char filename[100];
struct people person[50000000];
```

```
long int totalPersons=0;

void setPopulationAge()
{
    for( int j=0; j<71; j++){
        for( int i=0; i<51; i++){
            fscanf(F_Population,"%d",&populationPerYearAndAge[1950+j][15+
i]);
        }
    }
}

void setDeathRate()
{
    for( int i=0; i<51; i++){
        fscanf(F_DeathRate,"%lf",&deathRatePerAge[15+i*5]);
        for( int j=0; j<5; j++){
            deathRatePerAge[15+i*5+j] = deathRatePerAge[15+i*5];
        }
    }
}

void getTotalPopulationPerAge()
{
    for( int j=0; j<71; j++){
        for( int i=0; i<51; i++){
            totalPopulationPerYear[1950+j] += populationPerYearAndAge[195
0+j][15+i];
        }
    }
}
```

```
void printTotalPopulationPerAge()
{
    printf("---- - ----------\n");
    printf("Year - Population\n");
    printf("---- - ----------\n");
    for( int i=1950; i<2021; i++){
        printf("%d - %d\n",i,totalPopulationPerYear[i]);
    }
}

void printDeathRatePerAge()
{
    printf("--- - -----\n");
    printf("Age - Rate\n");
    printf("--- - -----\n");
    for( int i=15; i<65; i++){
        printf("%d - %lf\n",i,deathRatePerAge[i]);
    }
}

void printTotalPersonPerState( int year)
{
    int count[4]= {0,0,0,0};
    for( int i=0; i<totalPersons; i++){
        count[person[i].state]++;
    }
    //printf("%d\t%ld\t%ld\t%ld\t%ld\n",year,count[0],count[1],count[2],c
ount[3]);
    if(year==2014)
        printf("%d\t%ld\t%ld\t%ld\t%ld\t%lf\n",year,count[0],count[1],cou
nt[2],count[3],pa);
    if( year>2009){
```

```
        fprintf(out, "%d\t%ld\t%ld\t%ld\t%ld\n",year,count[0],count[1],co
unt[2],count[3]);
    }
}

void initializePopulation()
{
    int cnt=0;
    for( int j=15; j<65; j++){
        for( int i=0; i<populationPerYearAndAge[1950][j]*100; i++){
            person[cnt].age=j;
            person[cnt].state=0;
            cnt++;
        }
    }
    totalPersons = cnt;
}

bool checkRate(double rate)
{
    if (rate <0.001){
        double k = rand()/(1.00+RAND_MAX);
        if(k<0.001){
            rate = rate*1000;
        }
        else{
            return false;
        }
    }
    double k = rand()/(1.00+RAND_MAX);
    if ( k < rate )
        return true;
```

```
    else
        return false;
}

void changeStatusAndAge()
{
    for( int i=0; i<totalPersons; i++){
        // change state
        double k = rand()/(1.00+RAND_MAX);
        if( person[i].state==0){
            if ( checkRate(pa) &&  person[i].age<40){
                person[i].state=1;
            }
        }
        else if( person[i].state==1){
            if ( k < pg ){
                person[i].state=2;
            }
        }
        else if( person[i].state==2){
            if ( k < pk ){
                person[i].state=1;
            }
        }
        // After their 49th year, all active PWID are assumed to cease in
jecting
        if(person[i].age>49 && person[i].state==1){
            person[i].state=2;
        }
        // After their 64th year, remove
        if(person[i].age>64){
            person[i].state=3;
```

```
        }
        // death rate depending on age and state
        k = rand()/(1.00+RAND_MAX);
        if( person[i].state==0){
            if ( k < deathRatePerAge[person[i].age] ){
                person[i].state=3;
            }
        }
        else if( person[i].state==1){
            if ( k < deathRatePerAge[person[i].age] * 13.0 ){
                person[i].state=3;
            }
        }
        else if( person[i].state==2){
            if ( k < deathRatePerAge[person[i].age] ){
                person[i].state=3;
            }
        }
        // increase age one year
        person[i].age++;
    }
}

void addNewPersons(int year)
{
    // add new person [15 years old and non PWID]
    int cnt=totalPersons;
    for( int i=0; i<populationPerYearAndAge[year+1][15]*100; i++){
// 100 instead of 1000 for the population to reduce execution time
        person[cnt].age=15;
        person[cnt].state=0;
        cnt++;
```

```
    }
    // increase the total number of population
    totalPersons = cnt;
}
int main()
{
    srand(time(NULL));

    setPopulationAge();
    setDeathRate();

    getTotalPopulationPerAge();
    pa = pa_start;
    while ( pa < pa_stop){

        snprintf(filename, 100, "result_%lf.txt",pa);
        out=fopen(filename,"w");

        for( int iter=0; iter<loops ;iter++){
            initializePopulation();
            for( int year=1950; year<2020; year++){
                printTotalPersonPerState(year);
                changeStatusAndAge();
                addNewPersons(year);
            }
        }

        fclose(out);
        pa = pa + pa_step;
    }
```

```
}
```

## REFERENCES

European Centre for Disease Prevention and Control (ECDC). 2016. "Systematic Review on Hepatitis b and c Prevalence in the EU/EEA." https://www.ecdc.europa.eu/sites/default/files/media/en/publications/Publications/systematic-review-hepatitis-B-C-prevalence.pdf.

———. 2021. "Hepatitis c Prevalence Database." https://www.ecdc.europa.eu/en/all-topics-z/hepatitis-c/tools/hepatitis-c-prevalence-database.

Graf, Christiana, Marcus M Mücke, Georg Dultz, Kai-Henrik Peiffer, Alica Kubesch, Patrick Ingiliz, Stefan Zeuzem, Eva Herrmann, and Johannes Vermehren. 2019. "Efficacy of Direct-acting Antivirals for Chronic Hepatitis C Virus Infection in People Who Inject Drugs or Receive Opioid Substitution Therapy: A Systematic Review and Meta-analysis." *Clinical Infectious Diseases* 70 (11): 2355–65. https://doi.org/10.1093/cid/ciz696.

Grebely, Jason, Sarah Larney, Amy Peacock, Samantha Colledge, Janni Leung, Matthew Hickman, Peter Vickerman, et al. 2019. "Global, Regional, and Country-Level Estimates of Hepatitis c Infection Among People Who Have Recently Injected Drugs." *Addiction* 114 (1): 150–66. https://doi.org/https://doi.org/10.1111/add.14393.

Hines, Lindsey A., Adam Trickey, Janni Leung, Sarah Larney, Amy Peacock, Louisa Degenhardt, Samantha Colledge, et al. 2020. "Associations Between National Development Indicators and the Age Profile of People Who Inject Drugs: Results from a Global Systematic Review and Meta-Analysis." *The Lancet Global Health* 8 (1): e76–91. https://doi.org/10.1016/S2214-109X(19)30462-0.

Lin, Lifeng, and Haitao Chu. 2020. "Meta-Analysis of Proportions Using Generalized Linear Mixed Models." *Epidemiology* 31 (5). https://journals.lww.com/epidem/Fulltext/2020/09000/Meta_analysis_of_Proportions_Using_Generalized.16.aspx.

Litzroth, Amber, Vanessa Suin, Chloé Wyndham-Thomas, Sophie Quoilin, Gaëtan Muyldermans, Thomas Vanwolleghem, Benoît Kabamba-Mukadi, et al. 2019. "Low Hepatitis c Prevalence in

Belgium: Implications for Treatment Reimbursement and Scale Up." *BMC Public Health* 19 (1): 39. https://doi.org/10.1186/s12889-018-6347-z.

McDonald, Scott A., Rosmawati Mohamed, Maznah Dahlui, Herlianna Naning, and Adeeba Kamarulzaman. 2014. "Bridging the Data Gaps in the Epidemiology of Hepatitis c Virus Infection in Malaysia Using Multi-Parameter Evidence Synthesis." *BMC Infectious Diseases* 14 (1): 564. https://doi.org/10.1186/s12879-014-0564-6.

Micallef, J. M., J. M. Kaldor, and G. J. Dore. 2006. "Spontaneous Viral Clearance Following Acute Hepatitis c Infection: A Systematic Review of Longitudinal Studies." *Journal of Viral Hepatitis* 13 (1): 34–41. https://doi.org/https://doi.org/10.1111/j.1365-2893.2005.00651.x.

Plettinckx, Els, Forrest W. Crawford, Jerome Antoine, Lies Gremeaux, and Luk Van Baelen. 2021. "Estimates of People Who Injected Drugs Within the Last 12 Months in Belgium Based on a Capture-Recapture and Multiplier Method." *Drug and Alcohol Dependence* 219: 108436. https://doi.org/https://doi.org/10.1016/j.drugalcdep.2020.108436.

Presanis, Anne M., O. Noel Gill, Timothy R. Chadborn, Caterina Hill, Vivian Hope, Louise Logan, Brian D. Rice, Valerie C. Delpech, A. E. Ades, and Daniela De Angelis. 2010. "Insights into the Rise in HIV Infections, 2001 to 2008: A Bayesian Synthesis of Prevalence Evidence." *AIDS* 24 (18). https://journals.lww.com/aidsonline/Fulltext/2010/11270/Insights_into_the_rise_in_HIV_infections,_2001_to.12.aspx.

Sweeting, M. J., D. De Angelis, M. Hickman, and A. E. Ades. 2008. "Estimating hepatitis C prevalence in England and Wales by synthesizing evidence from multiple data sources. Assessing data conflict and model fit." *Biostatistics* 9 (4): 715–34. https://doi.org/10.1093/biostatistics/kxn004.

Tan, Sarah, Susanna Makela, Daliah Heller, Kevin Konty, Sharon Balter, Tian Zheng, and James H. Stark. 2018. "A Bayesian Evidence Synthesis Approach to Estimate Disease Prevalence in Hard-to-Reach Populations: Hepatitis c in New York City." *Epidemics* 23: 96–109. https://doi.org/https://doi.org/10.1016/j.epidem.2018.01.002.

Thein, Hla-Hla, Qilong Yi, Gregory J. Dore, and Murray D. Krahn. 2008. "Estimation of Stage-Specific Fibrosis Progression Rates in Chronic Hepatitis c Virus Infection: A Meta-Analysis and

Meta-Regression." *Hepatology* 48 (2): 418–31. https://doi.org/https://doi.org/10.1002/hep.22375.

Van Baelen, Luk, Els Plettinckx, Jérôme Antoine, and Lies Gremeaux. 2020. "Prevalence of HCV Among People Who Inject Drugs in Brussels—a Respondent-Driven Sampling Survey." *Harm Reduction Journal* 17 (1): 11. https://doi.org/10.1186/s12954-020-00358-3.

Veen, Maaike G. van, Anne M. Presanis, Stefano Conti, Maria Xiridou, Annemarie R. Stengaard, Martin C. Donoghoe, Ard I. van Sighem, Marianne A. van der Sande, and Daniela De Angelis. 2011. "National Estimate of HIV Prevalence in the Netherlands: Comparison and Applicability of Different Estimation Tools." *AIDS* 25 (2). https://journals.lww.com/aidsonline/Fulltext/2011/01140/National_estimate_of_HIV_prevalence_in_the.14.aspx.

# Estimating the prevalence of chronic hepatitis C infection (CHC) in Bulgaria using Bayesian multiparameter evidence synthesis

06/03/2022

## Table of Contents


Introduction ..... 2

Methods ..... 2

Prevalence of ex-PWID ..... 3

Prevalence of recent PWID ..... 3

Prevalence of CHC among recent PWID ..... 4

Prevalence of CHC among ex-PWID ..... 4

Prevalence of CHC among non-PWID ..... 5

Sensitivity analysis including migrants from endemic countries ..... 6

Results ..... 6

Tables and Figures ..... 8

Appendix ..... 12

Fit of the multi-state Markov model ..... 12

Stan code for Bayesian multiparameter evidence synthesis ..... 13

Multi-state Markov model ..... 17

References ..... 25

# INTRODUCTION

Patients with chronic hepatitis C (HCV) infection often remain asymptomatic for decades and even for life, but a proportion of them develop active chronic hepatitis, which is a progressive disease (Thein et al. 2008). Because of the largely asymptomatic nature of HCV infection, notification data reflect national screening and testing practices and do not give accurate insights into the prevalence of HCV infection. Although there are studies that have estimated the prevalence of HCV antibodies (anti-HCV) in specific groups such as the general population, first-time blood donors, or people who inject drugs (PWID) at drug treatment centers, those alone cannot be combined to produce national estimates unless some additional information regarding the composition of each HCV risk group and its prevalence in the population are known (Sweeting et al. 2008; McDonald et al. 2014; Tan et al. 2018).

# METHODS

Bayesian multiparameter evidence synthesis (BMES) has been a popular approach to formally estimate anti-HCV or HIV prevalence (Sweeting et al. 2008; McDonald et al. 2014; Tan et al. 2018; Presanis et al. 2010; Veen et al. 2011). In this project, we extend this method to estimating the CHC prevalence, with the population (15-79 years) split into three main non-overlapping risk groups, i.e., recent PWID (those who have injected in the last year), ex-PWID, and non-PWID. A unified model is assumed including parameters associated with the CHC prevalence of recent, ex-PWID, and non-PWID (denoted by $\pi_{rec}$, $\pi_{ex}$, and $\pi_{non}$, respectively) and the prevalence of these risk groups in the population (denoted by $\rho_{rec}$, $\rho_{ex}$, and $\rho_{non}$, respectively; $\rho_{rec} + \rho_{ex} + \rho_{non} = 1$). To estimate the CHC prevalence in the whole population, $\pi$, we used the formula:

$$\pi = \rho_{rec}\pi_{rec} + \pi_{ex}\rho_{ex} + \pi_{non}\rho_{non} \quad (1)$$

Bayesian synthesis considers all available data, allowing for an evidence-based estimation of CHC prevalence in the population, with inherent uncertainty properly accounted for. Our aim is to estimate the CHC prevalence for the total (15-79) population of Bulgaria in 2019 using sources of information over the last decade (2010-2019).

## Prevalence of ex-PWID

Data on the prevalence of ex-PWID are sparse and generally often unreliable. To overcome this limitation, we apply the method proposed by McDonald et al. (2014), which requires simulating a multi-state Markov model representing the non-PWID, recent PWID, and ex-PWID. In the multi-state Markov model, the simulation starts in 1950 and examines the population aged 15-79 years old. A certain number of 15-year-old individuals enter the simulation process each year. These numbers of 15-year-old individuals are obtained from the United Nations Statistic Division UNSD, being different for each country. The ageing of the population is also taken into account. Country-specific overall mortality data are retrieved from the Life Tables of the World Health Organization WHO. The duration of injecting career is also country-specific and obtained from the paper of Hines et al. (2020).

To estimate the number of ex-PWID, the model can be calibrated on the number of recent PWID provided in the EMCDDA barometer or the EMCDDA statistical bulletin (the code of the Markov model can be found in the Appendix). If there are no available data in the barometer, estimates from the systematic review of Grebely et al. (2019) could be used. Alternatively, if the national focal points suggest or provide different and updated or more accurate data for calibration purposes, we will consider their advice and adjust the model accordingly.

After applying the model for Bulgaria, we compute the number (and the corresponding Confidence Interval - CI) of ex-PWID in 2019. The number of ex-PWID is then divided by the population size (15-79 years) in the same year, and a Normal distribution, constrained such that $\rho_{ex} \in (0,1)$, is assumed for $\rho_{ex}$. The standard deviation of this distribution is specified to approximately correspond to the respective CI, i.e. $(UB_{\rho_{ex}} - LB_{\rho_{ex}})/(2 \times 1.96)$, where $UB_{\rho_{ex}}$ and $LB_{\rho_{ex}}$ denote the upper and lower limit of the CI.

## Prevalence of recent PWID

The number of recent PWID in Bulgaria in 2019 is also projected by the multi-state Markov model described in the previous subsection. To estimate $\rho_{rec}$, the number of recent PWID is divided by the population size (15-79 years). Similar to the previous subsection, a Normal distribution, constrained such that $\rho_{rec} \in (0,1)$, is assumed for $\rho_{rec}$, with the standard deviation specified to correspond to the CI obtained from the multi-state Markov model.

## Prevalence of CHC among recent PWID

The prevalence (and the 95% CI) of CHC among recent PWID can be informed from studies in the EMCDDA statistical bulletin. If information on the CHC prevalence is available, the Binomial distribution in the model to inform $\pi_{rec}$ was used. However, as these data EMCDDA typically refer to the anti-HCV prevalence and not to the CHC prevalence, they could not be used directly. This issue is addressed by noting that spontaneous viral clearance occurs in approximately one in four people with acute hepatitis C (Micallef, Kaldor, and Dore 2006). To account for the variability of the HCV clearance probabilities, we used the result reported in Micallef, Kaldor, and Dore (2006), i.e., the proportion of HCV clearance (denoted by $\rho_{clear}$) is equal to 0.26 (95% CI 0.22–0.29). Thus, estimates of the CHC prevalence among recent PWID ($\pi_{rec}$) can be obtained using the formula $\pi_{rec} = \pi(\text{anti-HCV})_{rec}(1-\rho_{clear})$, where $\pi(\text{anti-HCV})_{rec}$ denotes the anti-HCV prevalence among recent PWID.

Apart from spontaneous clearance, some people may have been treated with direct acting antivirals (DAAs). Since treatment data may be weak and unstandardised or unavailable for most EU/EEA countries, we have not formally taken treatment into account.

If information on the HCV prevalence (CHC or anti-HCV) among recent PWID in the EMCDDA database is not available, the required information is obtained from the paper of Grebely et al. (2019). Currently, anti-HCV prevalence data from a multi-regional study on current PWID in 2016, available in the EMCDDA statistical bulletin, was used. If the national focal point recommends updated formal estimates, the model input could be adjusted accordingly.

## Prevalence of CHC among ex-PWID

Information on CHC prevalence among ex-PWID (i.e., $\pi_{ex}$) is difficult to obtain directly. To overcome this, nationwide data on HCV prevalence among ever users through the EMCDDA database was utilized. However, if these data refer to the anti-HCV prevalence, they are adjusted according to the procedure described in the previous subsection, i.e. $\pi_{ever} = \pi(\text{anti-HCV})_{ever}(1-\rho_{clear})$, where $\pi(\text{anti-HCV})_{ever}$ denotes the anti-HCV prevalence among ever PWID. In any case, if an estimate of $\pi_{ever}$ is available, $\pi_{ex}$ can be indirectly estimated since $\pi_{ever}$ is equal to a weighted average of $\pi_{rec}$ and $\pi_{ex}$, as shown by the following formula

$$\pi_{ever} = \frac{\rho_{rec}}{\rho_{rec} + \rho_{ex}} \pi_{rec} + \frac{\rho_{ex}}{\rho_{rec} + \rho_{ex}} \pi_{ex}, \quad (2)$$

or, equivalently,

$$\pi_{ex} = \left(\pi_{ever} - \frac{\rho_{rec}}{\rho_{rec} + \rho_{ex}} \pi_{rec}\right) \times \frac{\rho_{rec} + \rho_{ex}}{\rho_{ex}}, \quad (3)$$

Currently, anti-HCV prevalence data from ever PWID going to drug treatment in Sofia in 2019, available in the EMCDDA statistical bulletin, was used. In any case, the model could be updated with any other relevant study/information suggested by the national focal point.

## Prevalence of CHC among non-PWID

To estimate the CHC prevalence among non-PWID in a country (after 2010), the ECDC database (European Centre for Disease Prevention and Control (ECDC) 2021) was used. The ECDC group has carefully and critically collected information from relevant studies across the EU/EEA region in cooperation with the focal points of each country. On the basis of the representativeness of each study and other factors (European Centre for Disease Prevention and Control (ECDC) 2016), a risk of bias score was assigned to each study, with higher values indicating higher-quality studies. When higher-quality studies are available in a country (≥ 4), they are used to estimate the overall CHC prevalence in that country among non-PWID. If there are no higher-quality studies, general population estimates with a lower quality (<4) are pooled. If no general population prevalence estimates are available, data on the prevalence of HCV in pregnant women could be used as a prevalence measure. Finally, if data on pregnant women are lacking too, first-time blood donor studies could be an option. However, if the national focal point agrees or recommends, estimates from a neighboring country could be used instead, or we could use any other relevant information provided/suggested by the national focal point.

We directly use CHC prevalence data from studies in the general population, if available. If the data in the general population refer to anti-HCV prevalence (without any data on the viremic population), we adjust the estimates to get CHC prevalence based on the spontaneous HCV clearance estimate of 26%, described in the previous subsection.

As $\rho_{rec}$ and $\rho_{ex}$ are likely to be low, $\pi_{non}$ will be the most critical factor for the estimation of the overall CHC prevalence in the population. To adjust for potential heterogeneity between the CHC prevalence estimates from different studies in the non-PWID population in a country, as a sensitivity analysis, we also apply a random-effect meta-analytic approach to pool the CHC prevalence across studies on non-PWID (Lin and Chu 2020). The random-effect approach is carried out only when at least 3 studies are available in the general population of a certain country, and the estimates are compared with the corresponding estimates from the approach assuming no heterogeneity (similar to a fixed-effect meta-analysis). If the estimates are inconsistent, possible explanations are investigated and discussed. If there is substantial heterogeneity between studies, it would help more to explore the causes; for example, following national focal point suggestions, some studies could be excluded to reduce heterogeneity.

## Sensitivity analysis including migrants from endemic countries

A sensitivity analysis including data for the viremic population among migrants based on the report of the ECDC group (European Centre for Disease Prevention and Control 2016) was carried out. Migrants from endemic countries represent 1% of the adult population in Bulgaria (Table 8 in European Centre for Disease Prevention and Control (2016)), with the respective CHC prevalence being equal to $\pi_{mig} = 2.2\%$ (Table 9 in European Centre for Disease Prevention and Control (2016)). The overall CHC prevalence is now estimated by

$$\pi = \rho_{rec}\pi_{rec} + \pi_{ex}\rho_{ex} + \pi_{mig}\rho_{mig} + \pi_{non}\rho_{non},$$

where $\rho_{rec} + \rho_{ex} + \rho_{mig} + \rho_{non} = 1$.

# RESULTS

The above-mentioned approach was used to estimate the prevalence of CHC in 2019 (considering ages between 15-79 years: 5,656,584).

The aggregated data used by our approach are briefly presented in Table 1. Since there were no available data to estimate the prevalence of recent and ex-PWID for Bulgaria in the EMCDDA barometer or database, the multi-state Markov model was calibrated on the estimates reported in the systematic review of Grebely et al. (2019). In Bulgaria, there were 2 studies on non-PWID of medium quality, which included CHC data. To estimate the CHC prevalence in the general

population (primarily non-PWID), we used a fixed-effect meta-analytic approach, with the corresponding results presented in Table 2. The prevalence of recent and ex-PWID was low in Bulgaria (about 0.28% and 0.92%, respectively) corresponding to 15,600 (95% CI: 14,760-16,370) recent PWID and 51,920 (95% CI: 50,430-53,520) ex-PWID in the population. However, the CHC prevalence in these groups was substantial (Table 2) being 50.67% and 60.05%, respectively. This translates to 7,903 (95% CI: 7,145-8,709) and 31,174 (95% CI: 28,477-33,958) recent and ex-PWID aged 15-79 living with CHC infection in Bulgaria in 2019. The CHC prevalence in the general population was 0.42% (95% CI: 0.15%-0.92%), much lower than that of the high-risk groups. Taking all pieces of information into account, the overall CHC prevalence in Bulgaria in 2019 was equal to 1.11% (95% CI: 0.83%-1.6%), which corresponds to 62,610 (95% CI: 47,033-90,766) individuals aged 15-79 years with CHC infection.

The results from our model including migrants from endemic countries as a separate group are presented in Table 3. However, this analysis comes with possible limitations; that is, including migrants as a separate group is valid only if migrants do not overlap with the remaining groups (recent PWID, ex-PWID, and non-PWID). Thus, if migrants do participate proportionally in the study(ies) in the general population, this analysis may result in biased overall CHC estimates (most probably in higher CHC prevalence estimates). However, if the national focal points consider that including migrants as a separate group is valid, we could consider results in Table 3 as the main analysis.

# TABLES AND FIGURES

*Table 1. Data (real) contributing to the estimation of CHC prevalence in Bulgaria in 2019.*

| Parameter | Number (95%CI) | Numerator | Denominator | Notes | Year of study |
|---|---|---|---|---|---|
| $\rho_{rec}$ | 15,600 (14,760-16,370) | | | Method based on McDonald et al. | 2019 |
| $\rho_{ex}$ | 51,920 (50,430-53,520) | | | Method based on McDonald et al. | 2019 |
| $\pi(\text{anti-HCV})_{rec}$ | | 246 | 359 | EMCDDA database | 2016 |
| $\pi(\text{anti-HCV})_{ever}$ | | 300 | 383 | EMCDDA database | 2019 |
| $\pi_{non}$ | | 3 | 865 | ECDC database (Kevorykan et al.); Risk of bias=3† | 2011 |
| $\pi_{non}$ | | 1 | 250 | ECDC database (Sperle et al.); Risk of bias=1†† | 2018 |

*Notes*: Higher risk of bias score denotes a higher-quality study (range from 0 to 6); † 3 CHC positive out of 6 anti-HCV positive; †† 2 both CHC and anti-HCV positive. Two participants reported injecting drug use, and one of them tested HCV positive.

*Table 2. Results from the method assuming no heterogeneity in the estimation of the CHC prevalence in the general population from different studies (i.e., similar to a fixed-effect meta-analysis).*

| Parameter | Posterior median | Posterior sd | LB (2.5%) | UB (97.5%) |
|---|---|---|---|---|
| $\rho_{rec}$ (%) | 0.28 | 0.01 | 0.26 | 0.29 |
| $\rho_{ex}$ (%) | 0.92 | 0.01 | 0.89 | 0.95 |
| $\pi_{rec}$ (%) | 50.67 | 2.18 | 46.42 | 54.98 |
| $\pi_{ex}$ (%) | 60.05 | 2.53 | 55.1 | 65.11 |
| $\pi_{non}$ (%) | 0.42 | 0.2 | 0.15 | 0.92 |
| $\pi$ (%) | 1.11 | 0.2 | 0.83 | 1.6 |
| Number with CHC | 62,610 | 11,263 | 47,033 | 90,766 |
| Pr(Ever PWID\|CHC) (%) | 62.56 | 10.28 | 43.14 | 82.76 |
| Pr(Non-PWID\|CHC) (%) | 37.44 | 10.28 | 17.24 | 56.86 |

*Notes*: $\rho_{rec}$, prevalence of recent PWID; $\rho_{ex}$, prevalence of ex-PWID; $\pi_{rec}$, CHC prevalence among recent PWID; $\pi_{ex}$, CHC prevalence among ex-PWID; $\pi_{non}$, CHC prevalence among non-PWID; $\pi$ overall CHC prevalence in Bulgaria; LB, Lower Boundary; UP, Upper Boundary

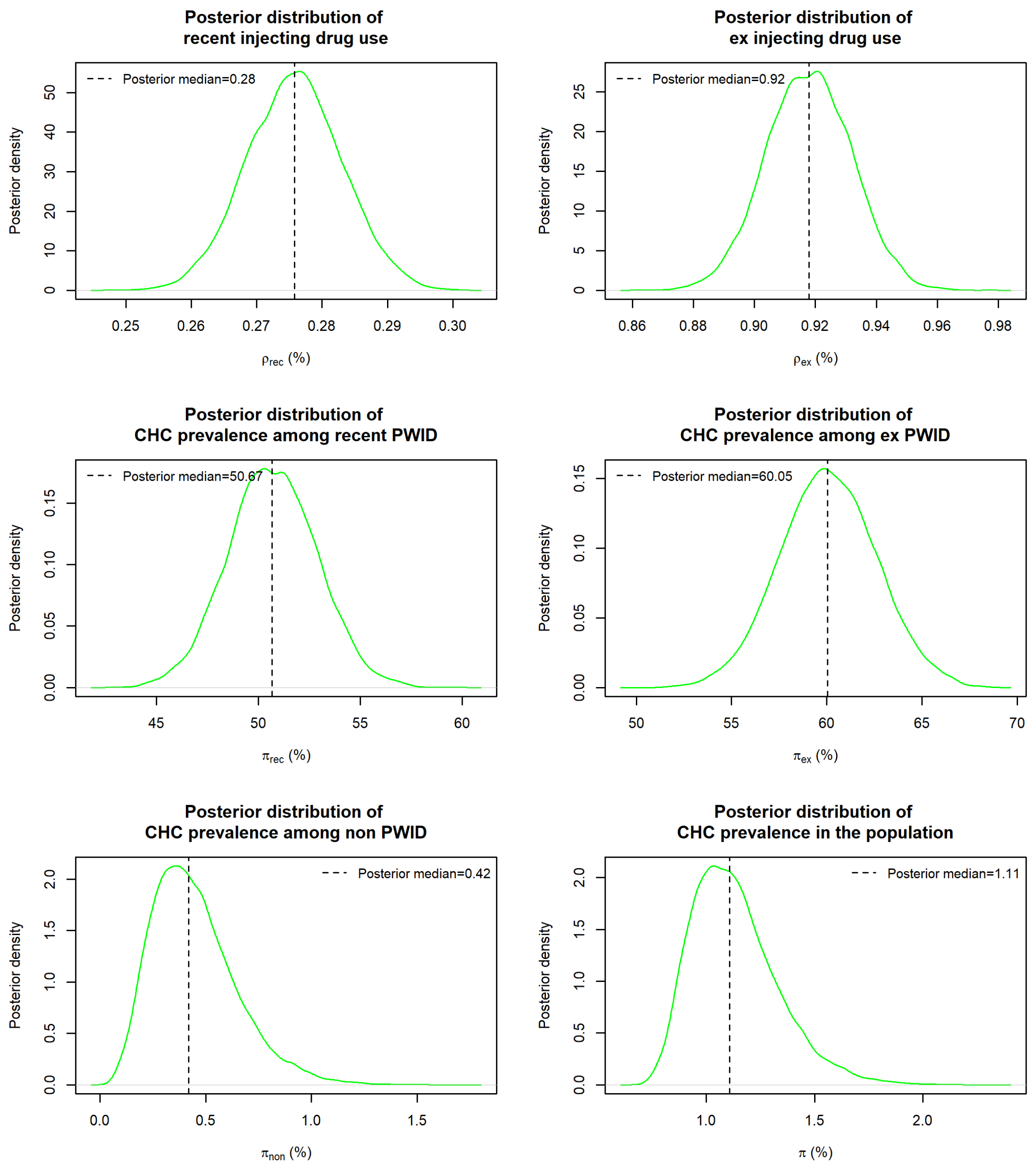


*Figure 1. Posterior distributions of the key model parameters using the approach that assumes no heterogeneity in the estimation of the CHC prevalence in the general population (primarily non-PWID) from different studies (i.e., similar to a fixed-effect meta-analysis).*

*Table 3. Results from the method assuming no heterogeneity in the estimation of the CHC prevalence in the general population (primarily non-PWID) from different studies (i.e., similar to a fixed-effect meta-analysis) including migrants from endemic countries as a separate group.*

| Parameter | Posterior median | Posterior sd | LB (2.5%) | UB (97.5%) |
|---|---|---|---|---|
| $\rho_{rec}$ (%) | 0.28 | 0.01 | 0.26 | 0.29 |
| $\rho_{ex}$ (%) | 0.92 | 0.01 | 0.89 | 0.94 |
| $\rho_{mig}$ (%) | 1 | 0 | 1 | 1 |
| $\pi_{rec}$ (%) | 50.71 | 2.18 | 46.39 | 54.93 |
| $\pi_{ex}$ (%) | 60.09 | 2.55 | 55.1 | 65.11 |
| $\pi_{mig}$ (%) | 2.2 | 0.5 | 1.21 | 3.18 |
| $\pi_{non}$ (%) | 0.42 | 0.2 | 0.15 | 0.91 |
| $\pi$ (%) | 1.12 | 0.2 | 0.85 | 1.61 |
| Number with CHC | 63,620 | 11,066 | 48,154 | 90,845 |
| Pr(Ever PWID\|CHC) (%) | 61.51 | 9.79 | 43 | 80.88 |
| Pr(Mig\|CHC) (%) | 1.92 | 0.55 | 0.99 | 3.14 |
| Pr(Non-PWID\|CHC) (%) | 36.52 | 10.1 | 16.6 | 55.63 |

*Notes*: $\rho_{rec}$, prevalence of recent PWID; $\rho_{ex}$, prevalence of ex-PWID; $\rho_{mig}$, prevalence of migrants from endemic countries; $\pi_{rec}$, CHC prevalence among recent PWID; $\pi_{ex}$, CHC prevalence among ex-PWID; $\pi_{mig}$, CHC prevalence among migrants from endemic countries; $\pi_{non}$, CHC prevalence among non-PWID; $\pi$ overall CHC prevalence in Bulgaria; LB, Lower Boundary; UP, Upper Boundary

# APPENDIX

## Fit of the multi-state Markov model

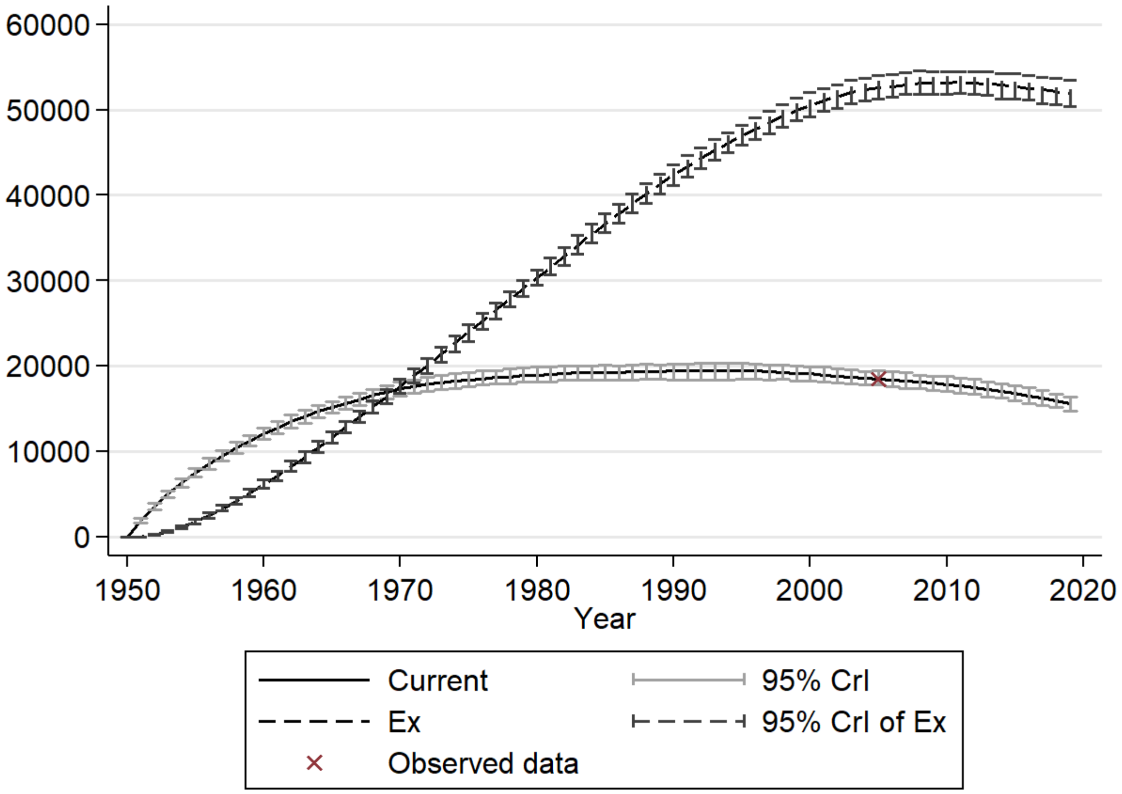


*Figure 2: Model predictions for the numbers of current and ex-PWID (people who inject drugs). The solid black line and shaded grey error bars show the median and 95% credible intervals (95% CrI) for the model projections. For comparison, asterisks indicate the observed country's data.*

## Stan code for Bayesian multiparameter evidence synthesis

```
data {
  int<lower=0> N1580; // Population of 15-64 in `Country`
  int<lower=1> Knon; // Number of studies in the ECDC data for `Country`
  int<lower=1> Kever; // Number of studies in the EMCDDA data for ever users in `Country`
  int<lower=1> Kcur; // Number of studies in the EMCDDA barometer (for HCV prevalence of PWID) in `Country`

  real p_cur_mean; // Prior mean for the prevalence of current use in `Country`
  real<lower=0> p_cur_sd; // Prior sd for the prevalence of current use in `Country`

  real p_ex_mean; // Prior mean for the prevalence of ex-use in `Country`
  real<lower=0> p_ex_sd; // Prior sd for the prevalence of ex-use in `Country`

  int<lower=0> Nst_hcv_cur[Kcur]; // Number of individuals in the study estimating anti-HCV among recent PWID
  int<lower=0> Yst_hcv_cur[Kcur]; // Number of HCV+ in the study estimating anti-HCV among recent PWID

  int<lower=0> Nst_CHC_ever[Kever]; // Number of individuals in the study estimating chronic HCV among ever IDU
  int<lower=0> Yst_CHC_ever[Kever]; // Number of HCV+ in the study estimating chronic HCV among ever PWID

  int<lower=0> Nst_CHC_non[Knon]; // Number of individuals in the study estimating chronic HCV among non PWID
  int<lower=0> Yst_CHC_non[Knon]; // Number of HCV+ in the study estimating chronic HCV among non PWID
```

```
  vector<lower=0>[3] alpha; // parameter of the Diriclet prior

  real HCVclear_mean; // Prior mean for the HCV clearance probability
  real<lower=0> HCVclear_sd; // Prior sd for the HCV clearance probabilit
y
}

// Block defining the original parameters
parameters {
  // The parameters to be sampled
  simplex[3] rho; // Prevalence of the three risk groups
  real<lower=0,upper=1> CHCpi_cur; // P(CHC+|Cur)
  real<lower=0,upper=1> CHCpi_ex; // P(CHC+|Ex)
  real<lower=0,upper=1> CHCpi_non; // P(CHC+|Non)

  real<lower=0,upper=1-CHCpi_cur> HCVclear; // Probability of HCV clearan
ce; upper bound = 1-prevalence of chronic HCV
}



transformed parameters {
  // Change scales
  real<lower=0,upper=1> rho_ever;
  real<lower=0,upper=1> CHCpi_ever;
  real<lower=0,upper=1> pi_ever;
  real<lower=0,upper=1> pi_cur;

  rho_ever = rho[1] + rho[2];
  CHCpi_ever = CHCpi_cur*rho[1]/rho_ever + CHCpi_ex*rho[2]/rho_ever;
  pi_ever = CHCpi_ever/(1-HCVclear);
  pi_cur = CHCpi_cur/(1-HCVclear);
```

```
}



// Binomial regression model
model {
  // Priors
  rho ~ dirichlet(alpha);

  //////////////////////////////
  // Likelihood contributions //
  //////////////////////////////

  // Probability of HCV clearance
  HCVclear ~ normal(HCVclear_mean,HCVclear_sd);

  // Prevalence of current use
  rho[1] ~ normal(p_cur_mean,p_cur_sd);

  // Prevalence of ex-use
  rho[2] ~ normal(p_ex_mean,p_ex_sd);

  // Prevalence of chronic HCV among current users
  Yst_hcv_cur ~ binomial(Nst_hcv_cur,pi_cur);

  // Prevalence of HCV among ever users
  Yst_CHC_ever ~ binomial(Nst_CHC_ever,CHCpi_ever);

  // HCV+ among non
  Yst_CHC_non ~ binomial(Nst_CHC_non,CHCpi_non);
}
```

```
generated quantities {
  // Functions of parameters
  real<lower=0,upper=100> overalCHC;
  real logit_CHCpi_cur;
  real logit_CHCpi_ex;
  real logit_CHCpi_non;
  real logit_rho_cur;
  real logit_rho_ex;
  real logit_rho_non;
  real<lower=0,upper=1> pEverGivenCHC;
  real<lower=0,upper=1> pNonGivenCHC;
  real logit_HCVclear;
  real<lower=0> NumberCHC;

  // Overall HCV prevalence
  overalCHC = 100*(rho[1]*CHCpi_cur + rho[2]*CHCpi_ex +  rho[3]*CHCpi_non
);
  pEverGivenCHC = CHCpi_ever*rho_ever/(overalCHC/100);
  pNonGivenCHC = CHCpi_non*(1-rho_ever)/(overalCHC/100);
  NumberCHC = round(overalCHC*N1580/100);

  logit_rho_cur = logit(rho[1]);
  logit_rho_ex = logit(rho[2]);
  logit_rho_non = logit(rho[3]);
  logit_CHCpi_cur = logit(CHCpi_cur);
  logit_CHCpi_ex = logit(CHCpi_ex);
  logit_CHCpi_non = logit(CHCpi_non);
  logit_HCVclear = logit(HCVclear);
}
```

## Multi-state Markov model

```
#include <stdio.h>
#include <stdlib.h>
#include <string.h>
#include <math.h>
#include <time.h>

#define loops 100
int populationPerYearAndAge[2021][66];
double deathRatePerAge[66];
int totalPopulationPerYear[2021];

FILE *F_DeathRate=fopen("greece-deathRates.txt","r");
FILE *F_Population=fopen("greece-population.txt","r");
FILE *out;

double pa_start = 0.00012;                    // Setting Variable
double pa_step  = 0.000002;                   // Setting Variable
double pa_stop  = 0.000123;                   // Setting Variable

double pa;
double pg = 1.0/11.7;   // Assumed average active injecting career of  11
.7 years
double pk = 0.004*12.0;     // Relapse rate of 0.004/month

struct people{
    int age;
    int state;
};

char filename[100];
struct people person[50000000];
```

```
long int totalPersons=0;

void setPopulationAge()
{
    for( int j=0; j<71; j++){
        for( int i=0; i<51; i++){
            fscanf(F_Population,"%d",&populationPerYearAndAge[1950+j][15+
i]);
        }
    }
}

void setDeathRate()
{
    for( int i=0; i<51; i++){
        fscanf(F_DeathRate,"%lf",&deathRatePerAge[15+i*5]);
        for( int j=0; j<5; j++){
            deathRatePerAge[15+i*5+j] = deathRatePerAge[15+i*5];
        }
    }
}

void getTotalPopulationPerAge()
{
    for( int j=0; j<71; j++){
        for( int i=0; i<51; i++){
            totalPopulationPerYear[1950+j] += populationPerYearAndAge[195
0+j][15+i];
        }
    }
}
```

```
void printTotalPopulationPerAge()
{
    printf("---- - ----------\n");
    printf("Year - Population\n");
    printf("---- - ----------\n");
    for( int i=1950; i<2021; i++){
        printf("%d - %d\n",i,totalPopulationPerYear[i]);
    }
}

void printDeathRatePerAge()
{
    printf("--- - -----\n");
    printf("Age - Rate\n");
    printf("--- - -----\n");
    for( int i=15; i<65; i++){
        printf("%d - %lf\n",i,deathRatePerAge[i]);
    }
}

void printTotalPersonPerState( int year)
{
    int count[4]= {0,0,0,0};
    for( int i=0; i<totalPersons; i++){
        count[person[i].state]++;
    }
    //printf("%d\t%ld\t%ld\t%ld\t%ld\n",year,count[0],count[1],count[2],c
ount[3]);
    if(year==2014)
        printf("%d\t%ld\t%ld\t%ld\t%ld\t%lf\n",year,count[0],count[1],cou
nt[2],count[3],pa);
    if( year>2009){
```

```
        fprintf(out, "%d\t%ld\t%ld\t%ld\t%ld\n",year,count[0],count[1],co
unt[2],count[3]);
    }
}

void initializePopulation()
{
    int cnt=0;
    for( int j=15; j<65; j++){
        for( int i=0; i<populationPerYearAndAge[1950][j]*100; i++){
            person[cnt].age=j;
            person[cnt].state=0;
            cnt++;
        }
    }
    totalPersons = cnt;
}

bool checkRate(double rate)
{
    if (rate <0.001){
        double k = rand()/(1.00+RAND_MAX);
        if(k<0.001){
            rate = rate*1000;
        }
        else{
            return false;
        }
    }
    double k = rand()/(1.00+RAND_MAX);
    if ( k < rate )
        return true;
```

```
    else
        return false;
}

void changeStatusAndAge()
{
    for( int i=0; i<totalPersons; i++){
        // change state
        double k = rand()/(1.00+RAND_MAX);
        if( person[i].state==0){
            if ( checkRate(pa) &&  person[i].age<40){
                person[i].state=1;
            }
        }
        else if( person[i].state==1){
            if ( k < pg ){
                person[i].state=2;
            }
        }
        else if( person[i].state==2){
            if ( k < pk ){
                person[i].state=1;
            }
        }
        // After their 49th year, all active PWID are assumed to cease in
jecting
        if(person[i].age>49 && person[i].state==1){
            person[i].state=2;
        }
        // After their 64th year, remove
        if(person[i].age>64){
            person[i].state=3;
```

```
        }
        // death rate depending on age and state
        k = rand()/(1.00+RAND_MAX);
        if( person[i].state==0){
            if ( k < deathRatePerAge[person[i].age] ){
                person[i].state=3;
            }
        }
        else if( person[i].state==1){
            if ( k < deathRatePerAge[person[i].age] * 13.0 ){
                person[i].state=3;
            }
        }
        else if( person[i].state==2){
            if ( k < deathRatePerAge[person[i].age] ){
                person[i].state=3;
            }
        }
        // increase age one year
        person[i].age++;
    }
}

void addNewPersons(int year)
{
    // add new person [15 years old and non PWID]
    int cnt=totalPersons;
    for( int i=0; i<populationPerYearAndAge[year+1][15]*100; i++){
// 100 instead of 1000 for the population to reduce execution time
        person[cnt].age=15;
        person[cnt].state=0;
        cnt++;
```

```
    }
    // increase the total number of population
    totalPersons = cnt;
}
int main()
{
    srand(time(NULL));

    setPopulationAge();
    setDeathRate();

    getTotalPopulationPerAge();
    pa = pa_start;
    while ( pa < pa_stop){

        snprintf(filename, 100, "result_%lf.txt",pa);
        out=fopen(filename,"w");

        for( int iter=0; iter<loops ;iter++){
            initializePopulation();
            for( int year=1950; year<2020; year++){
                printTotalPersonPerState(year);
                changeStatusAndAge();
                addNewPersons(year);
            }
        }

        fclose(out);
        pa = pa + pa_step;
    }
```

```
}
```

# REFERENCES

European Centre for Disease Prevention and Control. 2016. "Epidemiological Assessment of Hepatitis b and c Among Migrants in the EU/EEA." https://www.ecdc.europa.eu/sites/default/files/media/en/publications/Publications/epidemiological-assessment-hepatitis-B-and-C-among-migrants-EU-EEA.pdf.

European Centre for Disease Prevention and Control (ECDC). 2016. "Systematic Review on Hepatitis b and c Prevalence in the EU/EEA." https://www.ecdc.europa.eu/sites/default/files/media/en/publications/Publications/systematic-review-hepatitis-B-C-prevalence.pdf.

———. 2021. "Hepatitis c Prevalence Database." https://www.ecdc.europa.eu/en/all-topics-z/hepatitis-c/tools/hepatitis-c-prevalence-database.

Grebely, Jason, Sarah Larney, Amy Peacock, Samantha Colledge, Janni Leung, Matthew Hickman, Peter Vickerman, et al. 2019. "Global, Regional, and Country-Level Estimates of Hepatitis c Infection Among People Who Have Recently Injected Drugs." *Addiction* 114 (1): 150–66. https://doi.org/https://doi.org/10.1111/add.14393.

Hines, Lindsey A., Adam Trickey, Janni Leung, Sarah Larney, Amy Peacock, Louisa Degenhardt, Samantha Colledge, et al. 2020. "Associations Between National Development Indicators and the Age Profile of People Who Inject Drugs: Results from a Global Systematic Review and Meta-Analysis." *The Lancet Global Health* 8 (1): e76–91. https://doi.org/10.1016/S2214-109X(19)30462-0.

Lin, Lifeng, and Haitao Chu. 2020. "Meta-Analysis of Proportions Using Generalized Linear Mixed Models." *Epidemiology* 31 (5). https://journals.lww.com/epidem/Fulltext/2020/09000/Meta_analysis_of_Proportions_Using_Generalized.16.aspx.

McDonald, Scott A., Rosmawati Mohamed, Maznah Dahlui, Herlianna Naning, and Adeeba Kamarulzaman. 2014. "Bridging the Data Gaps in the Epidemiology of Hepatitis c Virus Infection in Malaysia Using Multi-Parameter Evidence Synthesis." *BMC Infectious Diseases* 14 (1): 564. https://doi.org/10.1186/s12879-014-0564-6.

Micallef, J. M., J. M. Kaldor, and G. J. Dore. 2006. "Spontaneous Viral Clearance Following Acute Hepatitis c Infection: A Systematic Review of Longitudinal Studies." *Journal of Viral Hepatitis* 13 (1): 34–41. https://doi.org/https://doi.org/10.1111/j.1365-2893.2005.00651.x.

Presanis, Anne M., O. Noel Gill, Timothy R. Chadborn, Caterina Hill, Vivian Hope, Louise Logan, Brian D. Rice, Valerie C. Delpech, A. E. Ades, and Daniela De Angelis. 2010. "Insights into the Rise in HIV Infections, 2001 to 2008: A Bayesian Synthesis of Prevalence Evidence." *AIDS* 24 (18). https://journals.lww.com/aidsonline/Fulltext/2010/11270/Insights_into_the_rise_in_HIV_infections,_2001_to.12.aspx.

Sweeting, M. J., D. De Angelis, M. Hickman, and A. E. Ades. 2008. "Estimating hepatitis C prevalence in England and Wales by synthesizing evidence from multiple data sources. Assessing data conflict and model fit." *Biostatistics* 9 (4): 715–34. https://doi.org/10.1093/biostatistics/kxn004.

Tan, Sarah, Susanna Makela, Daliah Heller, Kevin Konty, Sharon Balter, Tian Zheng, and James H. Stark. 2018. "A Bayesian Evidence Synthesis Approach to Estimate Disease Prevalence in Hard-to-Reach Populations: Hepatitis c in New York City." *Epidemics* 23: 96–109. https://doi.org/https://doi.org/10.1016/j.epidem.2018.01.002.

Thein, Hla-Hla, Qilong Yi, Gregory J. Dore, and Murray D. Krahn. 2008. "Estimation of Stage-Specific Fibrosis Progression Rates in Chronic Hepatitis c Virus Infection: A Meta-Analysis and Meta-Regression." *Hepatology* 48 (2): 418–31. https://doi.org/https://doi.org/10.1002/hep.22375.

Veen, Maaike G. van, Anne M. Presanis, Stefano Conti, Maria Xiridou, Annemarie R. Stengaard, Martin C. Donoghoe, Ard I. van Sighem, Marianne A. van der Sande, and Daniela De Angelis. 2011. "National Estimate of HIV Prevalence in the Netherlands: Comparison and Applicability of Different Estimation Tools." *AIDS* 25 (2). https://journals.lww.com/aidsonline/Fulltext/2011/01140/National_estimate_of_HIV_prevalence_in_the.14.aspx.

# Estimating the prevalence of chronic hepatitis C infection (CHC) in Croatia using Bayesian multiparameter evidence synthesis

16/06/2022

## Table of Contents


Introduction ..... 2

Methods ..... 2

- Prevalence of ex-PWID ..... 3
- Prevalence of recent PWID ..... 3
- Prevalence of CHC among recent PWID ..... 4
- Prevalence of CHC among ex-PWID ..... 4
- Prevalence of CHC among non-PWID ..... 5
- Sensitivity analysis including migrants from endemic countries ..... 6

Results ..... 7

Tables and Figures ..... 9

Appendix ..... 14

- Fit of the multi-state Markov model ..... 14
- Stan code for Bayesian multiparameter evidence synthesis ..... 15
- Multi-state Markov model ..... 21

References ..... 29

# INTRODUCTION

Patients with chronic hepatitis C (HCV) infection often remain asymptomatic for decades and even for life, but a proportion of them develop active chronic hepatitis, which is a progressive disease (Thein et al. 2008). Because of the largely asymptomatic nature of HCV infection, notification data reflect national screening and testing practices and do not give accurate insights into the prevalence of HCV infection. Although there are studies that have estimated the prevalence of HCV antibodies (anti-HCV) in specific groups such as the general population, first-time blood donors, or people who inject drugs (PWID) at drug treatment centers, those alone cannot be combined to produce national estimates unless some additional information regarding the composition of each HCV risk group and its prevalence in the population are known (Sweeting et al. 2008; McDonald et al. 2014; Tan et al. 2018).

# METHODS

Bayesian multiparameter evidence synthesis (MPES) has been a popular approach to formally estimate anti-HCV or HIV prevalence (Sweeting et al. 2008; McDonald et al. 2014; Tan et al. 2018; Presanis et al. 2010; Veen et al. 2011). In this project, we extend this method to estimating the CHC prevalence, with the population (15-79 years) split into three main non-overlapping risk groups, i.e., recent PWID (those who have injected in the last year), ex-PWID, and non-PWID. A unified model is assumed including parameters associated with the CHC prevalence of recent, ex-PWID, and non-PWID (denoted by $\pi_{rec}$, $\pi_{ex}$, and $\pi_{non}$, respectively) and the prevalence of these risk groups in the population (denoted by $\rho_{rec}$, $\rho_{ex}$, and $\rho_{non}$, respectively; $\rho_{rec} + \rho_{ex} + \rho_{non} = 1$). To estimate the CHC prevalence in the whole population, $\pi$, we used the formula:

$$\pi = \pi_{rec}\rho_{rec} + \pi_{ex}\rho_{ex} + \pi_{non}\rho_{non} \quad (1)$$

Bayesian synthesis considers all available data, allowing for an evidence-based estimation of CHC prevalence in the population, with inherent uncertainty properly accounted for. Our aim is to estimate the CHC prevalence for the total (15-79) population of Croatia in 2019 using sources of information over the last decade (2010-2019).

## Prevalence of ex-PWID

Data on the prevalence of ex-PWID are sparse and generally often unreliable. To overcome this limitation, we apply the method proposed by McDonald et al. (2014), which requires simulating a multi-state Markov model representing the non-PWID, recent PWID, and ex-PWID. In the multi-state Markov model, the simulation starts in 1950 and examines the population aged 15-79 years old. A certain number of 15-year-old individuals enter the simulation process each year. These numbers of 15-year-old individuals are obtained from the United Nations Statistic Division UNSD, being different for each country. The ageing of the population is also taken into account. Country-specific overall mortality data are retrieved from the Life Tables of the World Health Organization WHO. The duration of injecting career is also country-specific and obtained from the paper of Hines et al. (2020).

To estimate the number of ex-PWID, the model can be calibrated on the number of recent PWID provided in the EMCDDA barometer or the EMCDDA statistical bulletin (the code of the Markov model can be found in the Appendix). If there are no available data in the barometer, estimates from the systematic review of Grebely et al. (2019) could be used. Alternatively, if the national focal points suggest or provide different and updated or more accurate data for calibration purposes, we will consider their advice and adjust the model accordingly.

After applying the model for Croatia, we compute the number (and the corresponding Confidence Interval - CI) of ex-PWID in 2019. The number of ex-PWID is then divided by the population size (15-79 years) in the same year, and a Normal distribution, constrained such that $\rho_{ex} \in (0,1)$, is assumed for $\rho_{ex}$. The standard deviation of this distribution is specified to approximately correspond to the respective CI, i.e. $\left(UB_{\rho_{ex}} - LB_{\rho_{ex}}\right)/(2 \times 1.96)$, where $UB_{\rho_{ex}}$ and $LB_{\rho_{ex}}$ denote the upper and lower limit of the CI.

## Prevalence of recent PWID

The number of recent PWID in Croatia in 2019 is also projected by the multi-state Markov model described in the previous subsection. To estimate $\rho_{rec}$, the number of recent PWID is divided by the population size (15-79 years). Similar to the previous subsection, a Normal distribution, constrained such that $\rho_{rec} \in (0,1)$, is assumed for $\rho_{rec}$, with the standard deviation specified to correspond to the CI obtained from the multi-state Markov model.

## Prevalence of CHC among recent PWID

The prevalence (and the 95% CI) of CHC among recent PWID was informed by anti-HCV prevalence data from a respondent-driven study in Zagreb, Split, and Rijeka (Handanagic et al. 2016) in 2,014, reported also in the EMCDDA statistical bulletin. The Binomial distribution in the model to inform $\pi_{rec}$ was used. However, as the data reported in Handanagic et al. (2016) refer to the anti-HCV prevalence and not to the CHC prevalence, they could not be used directly. This issue can be partly addressed by noting that spontaneous viral clearance occurs in approximately one in four people with acute hepatitis C (Micallef, Kaldor, and Dore 2006). To account for variability in the HCV clearance probabilities, we used the result reported in Micallef, Kaldor, and Dore (2006), i.e., the proportion of HCV clearance (denoted by $\rho_{clear}$) is equal to 0.26 (95% CI 0.22–0.29). However, some people may have been treated with direct acting antivirals (DAAs), with the sustained virologic response ($SVR_{PWID}$) among PWID estimated to be 88% (95% CI: 80% to 93%) (Graf et al. 2019). Based on information provided by the national focal point, the number of individuals treated with DAAs up to 2019 is equal to 1,569. However, the proportions of each risk group among treated with DAAs, i.e. $\rho_{rec|DAA}$, $\rho_{ex|DAA}$, and $\rho_{non|DAA}$ ($\rho_{rec|DAA} + \rho_{ex|DAA} + \rho_{non|DAA} = 1$), are not currently available in Croatia. In this report, we make the assumption that $\rho_{rec|DAA}$ is equal to the proportion of recent PWID among CHC-positive individuals, i.e. Pr(Recent PWID|CHC), as estimated by our model when the DAA uptake is ignored (Table 2). Similarly, we assume that $\rho_{ex|DAA} =$ Pr(Ex-PWID|CHC) and $\rho_{non|DAA} =$ Pr(Non-PWID|CHC). Thus, the CHC prevalence among recent PWID, adjusted for DAAs, can be estimated by

$$\pi_{rec} = \frac{N_{15,79}\rho_{rec}\pi(\text{anti-HCV})_{rec}(1-\rho_{clear}) - N_{DAA}\rho_{rec|DAA}SVR_{PWID}}{N_{15,79}\rho_{rec}}, \quad (2)$$

where $\pi(\text{anti-HCV})_{rec}$ denotes the anti-HCV estimate derived solely by the data reported in Handanagic et al. (2016).

## Prevalence of CHC among ex-PWID

Information on CHC prevalence among ex-PWID (i.e., $\pi_{ex}$) is difficult to obtain directly. To overcome this, national anti-HCV prevalence data from ever PWID from Slovenia in 2019, available in the EMCDDA statistical bulletin, were used. However, as these data refer to the anti-

HCV prevalence, they are adjusted according to the procedure described in the previous subsection, i.e.

$$\pi_{ever} = \frac{N_{15,79}\rho_{ever}\pi(\text{anti-HCV})_{ever}(1-\rho_{clear}) - N_{DAA}\rho_{ever|DAA}SVR_{PWID}}{N_{15,79}\rho_{ever}}. \quad (3)$$

where $\pi(\text{anti-HCV})_{ever}$ denotes the anti-HCV prevalence among ever PWID, $\rho_{ever} = \rho_{rec} + \rho_{ex}$, $\rho_{clear}$ denotes the spontaneous viral clearance [assumed to be equal to 0.26 (95% CI 0.22–0.29); (Micallef, Kaldor, and Dore 2006)] , and $\rho_{ever|DAA}$ the proportion of ever PWID among individuals treated with DAAs. Recall that it is assumed that $\rho_{ever|DAA}$ is assumed to be equal to Pr(Ever PWID|CHC), as estimated by our model ignoring the effect of DAAs.

Then, since an estimate of $\pi_{ever}$ is available, $\pi_{ex}$ can be indirectly estimated since $\pi_{ever}$ is equal to a weighted average of $\pi_{rec}$ and $\pi_{ex}$, as shown by the following formula

$$\pi_{ever} = \frac{\rho_{rec}}{\rho_{ever}}\pi_{rec} + \frac{\rho_{ex}}{\rho_{ever}}\pi_{ex}, \quad (4)$$

or, equivalently,

$$\pi_{ex} = \left(\pi_{ever} - \frac{\rho_{rec}}{\rho_{ever}}\pi_{rec}\right) \times \frac{\rho_{ever}}{\rho_{ex}}. \quad (5)$$

## Prevalence of CHC among non-PWID

To estimate the CHC prevalence among non-PWID in a country (after 2010), the ECDC database (European Centre for Disease Prevention and Control (ECDC) 2021) was used. The ECDC group has carefully and critically collected information from relevant studies across the EU/EEA region in cooperation with the focal points of each country. On the basis of the representativeness of each study and other factors (European Centre for Disease Prevention and Control (ECDC) 2016), a risk of bias score was assigned to each study, with higher values indicating higher-quality studies. When higher-quality studies are available in a country ($\geq 4$), they are used to estimate the overall CHC prevalence in that country among non-PWID. If there are no higher-quality studies, general population estimates with a lower quality (<4) are pooled. If no general population prevalence estimates are available, data on the prevalence of HCV in pregnant women could be used as a prevalence measure. Finally, if data on pregnant women are lacking too, first-time blood donor studies could be an option. However, if the

national focal point agrees or recommends, estimates from a neighboring country could be used instead, or we could use any other relevant information provided/suggested by the national focal point.

We directly use CHC prevalence data from studies in the general population, if available. If the data in the general population refer to anti-HCV prevalence (without any data on the viremic population), we adjust the estimates to get the CHC prevalence based on the spontaneous HCV clearance estimate of 26% and the number of individuals treated with DAAs in the general population, as previously described, i.e.

$$\pi_{non} = \frac{N_{15,79}\rho_{non}\pi(\text{anti-HCV})_{non}(1-\rho_{clear}) - N_{DAA}\rho_{non|DAA}SVR}{N_{15,79}\rho_{non}}. \quad (6)$$

where $\pi(\text{anti-HCV})_{non}$ denotes the anti-HCV prevalence among non-PWID and $SVR$ is the sustained virologic response of DAAs in the general population estimated to be 96.7% (95% CI: 95.4% to 98.1%)(Lampertico et al. 2020).

As $\rho_{rec}$ and $\rho_{ex}$ are likely to be low, $\pi_{non}$ will be the most critical factor for the estimation of the overall CHC prevalence in the population. To adjust for potential heterogeneity between the CHC prevalence estimates from different studies in the non-PWID population in a country, as a sensitivity analysis, we also apply a random-effect meta-analytic approach to pool the CHC prevalence across studies on non-PWID (Lin and Chu 2020). The random-effect approach is carried out only when at least 3 studies are available in the general population of a certain country, and the estimates are compared with the corresponding estimates from the approach assuming no heterogeneity (similar to a fixed-effect meta-analysis). If the estimates are inconsistent, possible explanations are investigated and discussed. If there is substantial heterogeneity between studies, it would help more to explore the causes; for example, following national focal point suggestions, some studies could be excluded to reduce heterogeneity.

## Sensitivity analysis including migrants from endemic countries

A sensitivity analysis including data for the viremic population among migrants based on the report of the ECDC group (European Centre for Disease Prevention and Control 2016) was carried out. Migrants from endemic countries represent 14.4% of the adult population in Croatia (Table 8 in European Centre for Disease Prevention and Control (2016)), with the

respective CHC prevalence being equal to $\pi_{mig} = 0.9\%$ (Table 9 in European Centre for Disease Prevention and Control (2016)). The overall CHC prevalence is now estimated by

$$\pi = \pi_{rec}\rho_{rec} + \pi_{ex}\rho_{ex} + \pi_{mig}\rho_{mig} + \pi_{non}\rho_{non},$$

where $\rho_{rec} + \rho_{ex} + \rho_{mig} + \rho_{non} = 1$. A treatment adjustment similar to that described in the previous subsections was also performed.

# RESULTS

The above-mentioned approach was used to estimate the prevalence of CHC in 2019 (considering ages between 15-79 years: 3,270,827).

The aggregated data used by our approach are briefly presented in Table 1. The multi-state Markov model was calibrated on the estimated prevalence of recent PWID reported in the systematic review of Grebely et al. (2019). In Croatia, there was 1 study on non-PWID of medium quality, which included only anti-HCV data (Vilibic-Cavlek et al. 2014). To estimate the CHC prevalence in the general population (primarily non-PWID), we used a fixed-effect meta-analytic approach, with the corresponding results, when information on DAAs is ignored, presented in Table 2. However, based on data provided by the focal point, approximately 1,569 individuals were treated with DAAs from 2015 to 2019 in Croatia, with the proportion of recent PWID, ex-PWID and non-PWID among the 1,569 treated individuals assumed to be equal to Pr(Recent PWID|CHC) ≈ 5.19% Pr(Ex-PWID|CHC) ≈ 3.89% and Pr(Non-PWID|CHC) ≈ 90.82%, respectively (Table 2).

The corresponding results accounting for the DAA uptake are presented in Table 3. The prevalence of recent and ex-PWID was low in Croatia (about 0.16% and 0.4%, respectively) corresponding to 5,275 (95% CI: 4,780-5,640) recent PWID and 13,085 (95% CI: 12,380-13,880) ex-PWID in the population. However, the CHC prevalence in these groups was substantial (Table 3) being 23.98% and 7.27%, respectively. This translates to 1,264 (95% CI: 1,100-1,442) and 951 (95% CI: 78-2,668) recent and ex-PWID aged 15-79 living with CHC infection in Croatia in 2019. The CHC prevalence in the general population was 0.67% (95% CI: 0.4%-1.04%), much lower than that of the high-risk groups. Taking all pieces of information into account, the overall CHC prevalence in Croatia in 2019 was equal to 0.74% (95% CI: 0.46%-

1.11%), which corresponds to 24,274 (95% CI: 15,060-36,404) individuals aged 15-79 years with CHC infection.

The results from our model including migrants from endemic countries as a separate group are presented in Table 4. However, this analysis comes with possible limitations; that is, including migrants as a separate group is valid only if migrants do not overlap with the remaining groups (recent PWID, ex-PWID, and non-PWID). Thus, if migrants do participate proportionally in the study(ies) in the general population, this analysis may result in biased overall CHC estimates (most probably in higher CHC prevalence estimates).

# TABLES AND FIGURES

*Table 1. Data (real) contributing to the estimation of CHC prevalence in Croatia in 2019.*

| Parameter | Number (95%CI) | Numerator | Denominator | Notes | Year of study |
|---|---|---|---|---|---|
| $\rho_{rec}$ | 5,275 (4,780-5,640) | | | Method based on McDonald et al. | 2019 |
| $\rho_{ex}$ | 13,085 (12,380-13,880) | | | Method based on McDonald et al. | 2019 |
| $\pi(\text{anti-HCV})_{rec}$ | | 280 | 817 | (Handanagic et al. 2016) | 2014 |
| $\pi(\text{anti-HCV})_{ever}$ | | 6 | 39 | EMCDDA database (Slovenia) | 2019 |
| $\pi(\text{anti-HCV})_{non}$ | | 18 | 1,930 | ECDC database (Vilibic-Cavlek et al.); Risk of bias=3 | 2011 |

*Notes*: Higher risk of bias score denotes a higher-quality study (range from 0 to 6);

*Table 2. Results from the method assuming no heterogeneity in the estimation of the CHC prevalence in the general population from different studies (i.e., similar to a fixed-effect meta-analysis).*

| Parameter | Posterior median | Posterior sd | LB (2.5%) | UB (97.5%) |
|---|---|---|---|---|
| $\rho_{rec}$ (%) | 0.16 | 0.01 | 0.15 | 0.17 |
| $\rho_{ex}$ (%) | 0.4 | 0.01 | 0.38 | 0.42 |
| $\pi_{rec}$ (%) | 25.39 | 1.35 | 22.8 | 28.12 |
| $\pi_{ex}$ (%) | 7.68 | 5.51 | 0.53 | 21.36 |
| $\pi_{non}$ (%) | 0.72 | 0.17 | 0.44 | 1.09 |
| $\pi$ (%) | 0.79 | 0.17 | 0.51 | 1.16 |
| Number with CHC | 25,789 | 5,445 | 16,771 | 38,028 |
| Pr(Recent PWID\|CHC) (%) | 5.19 | 1.19 | 3.45 | 8.1 |
| Pr(Ex-PWID\|CHC) (%) | 3.89 | 2.87 | 0.27 | 11.07 |
| Pr(Non-PWID\|CHC) (%) | 90.82 | 3.27 | 82.71 | 95.15 |

*Notes*: Information on DAAs is not taken into account.

*Abbreviations*: $\rho_{rec}$, prevalence of recent PWID; $\rho_{ex}$, prevalence of ex-PWID; $\pi_{rec}$, CHC prevalence among recent PWID; $\pi_{ex}$, CHC prevalence among ex-PWID; $\pi_{non}$, CHC prevalence among non-PWID; $\pi$ overall CHC prevalence in Croatia; LB, Lower Boundary; UP, Upper Boundary

*Table 3. Results from the method assuming no heterogeneity in the estimation of the CHC prevalence in the general population from different studies (i.e., similar to a fixed-effect meta-analysis).*

| Parameter | Posterior median | Posterior sd | LB (2.5%) | UB (97.5%) |
|---|---|---|---|---|
| $\rho_{rec}$ (%) | 0.16 | 0.01 | 0.15 | 0.17 |
| $\rho_{ex}$ (%) | 0.4 | 0.01 | 0.38 | 0.42 |
| $\pi_{rec}$ (%) | 23.98 | 1.34 | 21.42 | 26.66 |
| $\pi_{ex}$ (%) | 7.27 | 5.23 | 0.6 | 20.3 |
| $\pi_{non}$ (%) | 0.67 | 0.17 | 0.4 | 1.04 |
| $\pi$ (%) | 0.74 | 0.17 | 0.46 | 1.11 |
| Number with CHC | 24,274 | 5,526 | 15,060 | 36,404 |
| Pr(Recent PWID\|CHC) (%) | 5.22 | 1.24 | 3.45 | 8.27 |
| Pr(Ex-PWID\|CHC) (%) | 3.94 | 2.89 | 0.33 | 11.24 |
| Pr(Non-PWID\|CHC) (%) | 90.69 | 3.32 | 82.43 | 95.08 |

*Notes*: The number of individuals treated with DAAs is taken into account.

*Abbreviations*: $\rho_{rec}$, prevalence of recent PWID; $\rho_{ex}$, prevalence of ex-PWID; $\pi_{rec}$, CHC prevalence among recent PWID; $\pi_{ex}$, CHC prevalence among ex-PWID; $\pi_{non}$, CHC prevalence among non-PWID; $\pi$ overall CHC prevalence in Croatia; LB, Lower Boundary; UP, Upper Boundary

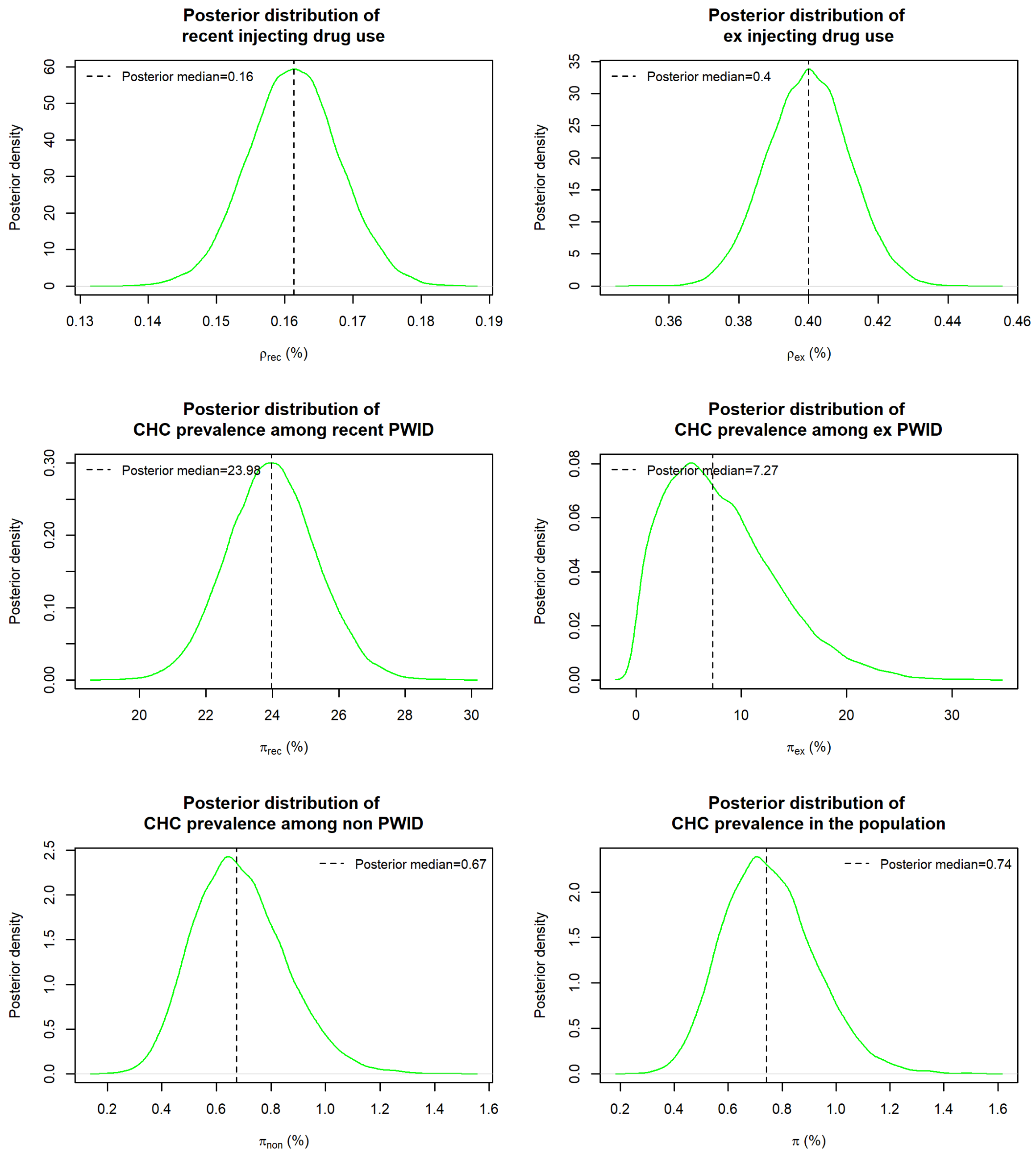


*Figure 1. Posterior distributions of the key model parameters using the approach that assumes no heterogeneity in the estimation of the CHC prevalence in the general population (primarily non-PWID) from different studies (i.e., similar to a fixed-effect meta-analysis), with the number of individuals treated with DAA taken into account.*

*Table 4. Results from the method assuming no heterogeneity in the estimation of the CHC prevalence in the general population (primarily non-PWID) from different studies (i.e., similar to a fixed-effect meta-analysis) including migrants from endemic countries as a separate group.*

| Parameter | Posterior median | Posterior sd | LB (2.5%) | UB (97.5%) |
|---|---|---|---|---|
| $\rho_{rec}$ (%) | 0.16 | 0.01 | 0.15 | 0.17 |
| $\rho_{ex}$ (%) | 0.4 | 0.01 | 0.38 | 0.42 |
| $\rho_{mig}$ (%) | 14.4 | 0 | 14.4 | 14.4 |
| $\pi_{rec}$ (%) | 24.07 | 1.34 | 21.55 | 26.79 |
| $\pi_{ex}$ (%) | 7.28 | 5.22 | 0.58 | 20.1 |
| $\pi_{mig}$ (%) | 0.85 | 0.09 | 0.67 | 1.03 |
| $\pi_{non}$ (%) | 0.68 | 0.17 | 0.41 | 1.05 |
| $\pi$ (%) | 0.77 | 0.15 | 0.53 | 1.09 |
| Number with CHC | 25,239 | 4,759 | 17,271 | 35,799 |
| Pr(Recent PWID\|CHC) (%) | 5.04 | 0.97 | 3.52 | 7.29 |
| Pr(Ex-PWID\|CHC) (%) | 3.75 | 2.7 | 0.31 | 10.46 |
| Pr(Mig\|CHC) (%) | 15.85 | 3.25 | 10.65 | 23.29 |
| Pr(Non-PWID\|CHC) (%) | 74.94 | 5.03 | 63.48 | 83.12 |

*Notes*: The number of individuals treated with DAAs is taken into account.

*Abbreviations*: $\rho_{rec}$, prevalence of recent PWID; $\rho_{ex}$, prevalence of ex-PWID; $\rho_{mig}$, prevalence of migrants from endemic countries; $\pi_{rec}$, CHC prevalence among recent PWID; $\pi_{ex}$, CHC prevalence among ex-PWID; $\pi_{mig}$, CHC prevalence among migrants from endemic countries; $\pi_{non}$, CHC prevalence among non-PWID; $\pi$ overall CHC prevalence in Croatia; LB, Lower Boundary; UP, Upper Boundary

# APPENDIX

## Fit of the multi-state Markov model

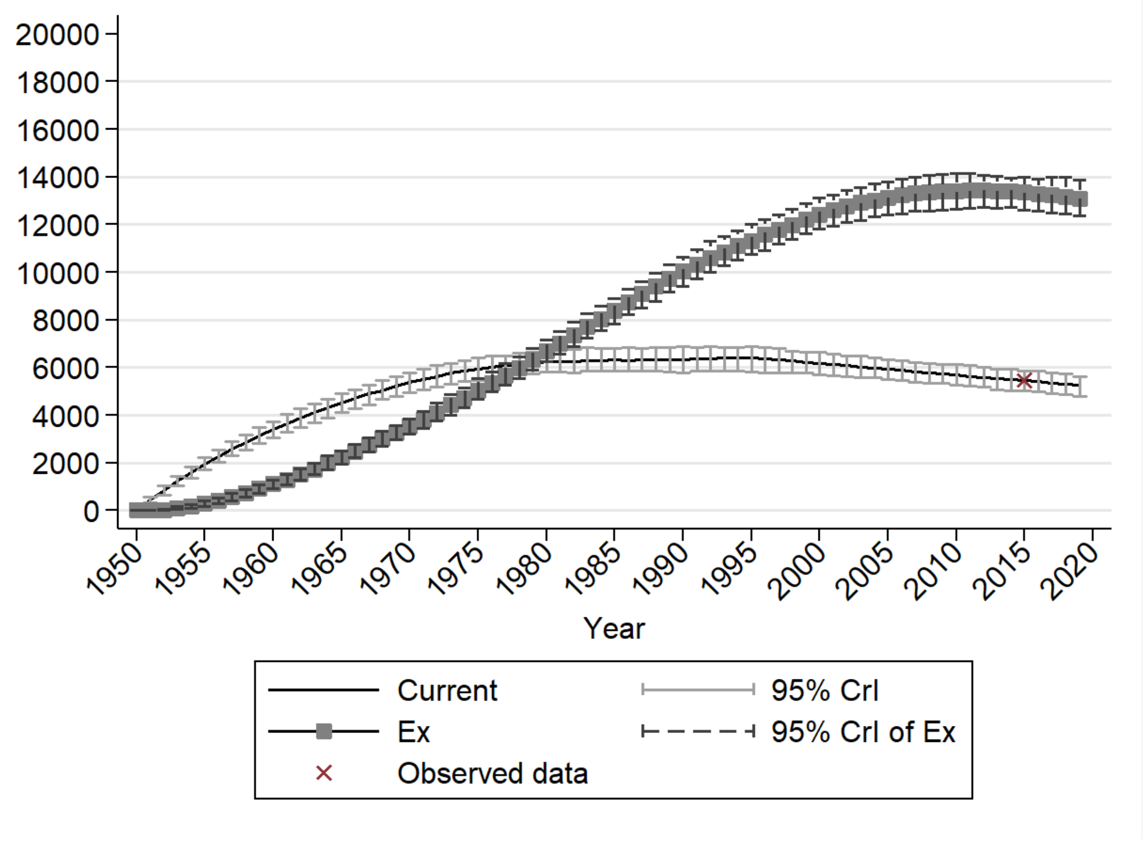


*Figure 2: Model predictions for the numbers of current and ex-PWID (people who inject drugs). The solid black line and shaded grey error bars show the median and 95% credible intervals (95% CrI) for the model projections. For comparison, asterisks indicate the observed country's data.*

## Stan code for Bayesian multiparameter evidence synthesis

```
data {
  int<lower=0> N1579; // Population of 15-79 in `Country`
  int<lower=1> Knon; // Number of studies in the ECDC data for `Country`
  int<lower=1> Kever; // Number of studies in the EMCDDA data for ever us
ers in `Country`
  int<lower=1> Kcur; // Number of studies in the EMCDDA barometer (for HC
V prevalence of PWID) in `Country`
  int<lower=1> NDAA; // Total number of DAAs from 2015 to 2019

  real p_cur_mean; // Prior mean for the prevalence of current use in `Co
untry`
  real<lower=0> p_cur_sd; // Prior sd for the prevalence of current use i
n `Country`

  real p_ex_mean; // Prior mean for the prevalence of ex-use in `Country`
  real<lower=0> p_ex_sd; // Prior sd for the prevalence of ex-use in `Cou
ntry`

  int<lower=0> Nst_hcv_cur[Kcur]; // Number of individuals in the study e
stimating anti-HCV among recent PWID
  int<lower=0> Yst_hcv_cur[Kcur]; // Number of HCV+ in the study estimati
ng anti-HCV among recent PWID

  int<lower=0> Nst_hcv_ever[Kever]; // Number of individuals in the study
estimating anti-HCV among ever IDU
  int<lower=0> Yst_hcv_ever[Kever]; // Number of HCV+ in the study estima
ting anti-HCV among ever PWID

  int<lower=0> Nst_hcv_non[Knon]; // Number of individuals in the study e
stimating anti-HCV among non PWID
  int<lower=0> Yst_hcv_non[Knon]; // Number of HCV+ in the study estimati
```

```
ng anti-HCV among non PWID

  vector<lower=0>[3] alpha; // parameter of the Diriclet prior

  real HCVclear_mean; // Prior mean for the HCV clearance probability
  real<lower=0> HCVclear_sd; // Prior sd for the HCV clearance probabilit
y

  real SVR_mean; // Prior mean for the SVR among non-PWID
  real<lower=0> SVR_sd; // Prior sd for the SVR among non-PWID

  real SVR_PWID_mean; // Prior mean for the SVR among PWID
  real<lower=0> SVR_PWID_sd; // Prior sd for the SVR among PWID
}

// Block defining the original parameters
parameters {
  // The parameters to be sampled
  simplex[3] rho; // Prevalence of the three risk groups
  real<lower=0,upper=1> CHCpi_cur; // P(CHC+|Cur)
  real<lower=0,upper=1> CHCpi_ex; // P(CHC+|Ex)
  real<lower=0,upper=1> CHCpi_non; // P(CHC+|Non)
  real<lower=0,upper=1> SVR; // SVR of DAAs among non-PWID
  real<lower=0,upper=1> SVR_PWID; // SVR of DAAs among PWID

  real<lower=0,upper=1-CHCpi_cur> HCVclear; // Probability of HCV clearan
ce; upper bound = 1-prevalence of chronic HCV
}


transformed parameters {
  // Change scales
```

```
  real<lower=0,upper=1> rho_ever;
  real<lower=0,upper=1> CHCpi_ever;
  real<lower=0,upper=1> pi_ever;
  real<lower=0,upper=1> pi_non;
  real<lower=0,upper=1> pi_cur;

  rho_ever = rho[1] + rho[2];
  CHCpi_ever = CHCpi_cur*rho[1]/rho_ever + CHCpi_ex*rho[2]/rho_ever;
  pi_ever = CHCpi_ever/(1-HCVclear);
  pi_non = CHCpi_non/(1-HCVclear);
  pi_cur = CHCpi_cur/(1-HCVclear);
}


// Binomial regression model
model {
  // Priors
  rho ~ dirichlet(alpha);

  ///////////////////////////////
  // Likelihood contributions //
  //////////////////////////////

  // Probability of HCV clearance
  HCVclear ~ normal(HCVclear_mean,HCVclear_sd);

  // Prevalence of current use
  rho[1] ~ normal(p_cur_mean,p_cur_sd);

  // Prevalence of ex-use
  rho[2] ~ normal(p_ex_mean,p_ex_sd);
```

```
  // Prevalence of chronic HCV among current users
  Yst_hcv_cur ~ binomial(Nst_hcv_cur,pi_cur);

  // Prevalence of HCV among ever users
  Yst_hcv_ever ~ binomial(Nst_hcv_ever,pi_ever);

  // HCV+ among non
  Yst_hcv_non ~ binomial(Nst_hcv_non,pi_non);

  // SVR of DAAs among non-PWID
  SVR ~ normal(SVR_mean,SVR_sd);

  // SVR of DAAs among PWID
  SVR_PWID ~ normal(SVR_PWID_mean,SVR_PWID_sd);
}


generated quantities {
  // Functions of parameters
  real<lower=0,upper=100> overalCHC;
  real logit_CHCpi_cur;
  real logit_CHCpi_ex;
  real logit_CHCpi_non;
  real logit_rho_cur;
  real logit_rho_ex;
  real logit_rho_non;
  real<lower=0,upper=1> pEverGivenCHC;
  real<lower=0,upper=1> pCurGivenCHC;
  real<lower=0,upper=1> pExGivenCHC;
  real<lower=0,upper=1> pNonGivenCHC;
  real logit_HCVclear;
  real<lower=0> NumberCHC;
```

```
  real<lower=0,upper=1> CHCDAApi_non; // P(CHC+|Non) adjusted for DAA
  real<lower=0,upper=1> CHCDAApi_cur; // P(CHC+|Cur) adjusted for DAA
  real<lower=0,upper=1> CHCDAApi_ex; // P(CHC+|Ex) adjusted for DAA
  real<lower=0,upper=1> CHCDAApi_ever; // P(CHC+|Ever) adjusted for DAA

  real<lower=0,upper=100> overalCHC_DAA;
  real pEverGivenCHC_DAA;
  real pCurGivenCHC_DAA;
  real pExGivenCHC_DAA;
  real pNonGivenCHC_DAA;
  real<lower=0> NumberCHC_DAA;

  // Overall HCV prevalence
  overalCHC = 100*(rho[1]*CHCpi_cur + rho[2]*CHCpi_ex +  rho[3]*CHCpi_non
);
  pEverGivenCHC = CHCpi_ever*rho_ever/(overalCHC/100);
  pCurGivenCHC = CHCpi_cur*rho[1]/(overalCHC/100);
  pExGivenCHC = CHCpi_ex*rho[2]/(overalCHC/100);
  pNonGivenCHC = CHCpi_non*(1-rho_ever)/(overalCHC/100);
  NumberCHC = round(overalCHC*N1579/100);

  CHCDAApi_non = ( N1579*rho[3]*CHCpi_non - NDAA*pNonGivenCHC*SVR)/(N1579
*rho[3]);
  CHCDAApi_cur = ( N1579*rho[1]*CHCpi_cur - NDAA*pCurGivenCHC*SVR_PWID_me
an)/(N1579*rho[1]);
  CHCDAApi_ex = ( N1579*rho[2]*CHCpi_ex - NDAA*pExGivenCHC*SVR_PWID_mean)
/(N1579*rho[2]);
  CHCDAApi_ever = CHCDAApi_cur*rho[1]/rho_ever + CHCDAApi_ex*rho[2]/rho_e
ver;

  overalCHC_DAA = 100*(rho[1]*CHCDAApi_cur + rho[2]*CHCDAApi_ex +  rho[3]
```

```
*CHCDAApi_non);
  pEverGivenCHC_DAA = CHCDAApi_ever*rho_ever/(overalCHC_DAA/100);
  pCurGivenCHC_DAA = CHCDAApi_cur*rho[1]/(overalCHC_DAA/100);
  pExGivenCHC_DAA = CHCDAApi_ex*rho[2]/(overalCHC_DAA/100);
  pNonGivenCHC_DAA = CHCDAApi_non*(1-rho_ever)/(overalCHC_DAA/100);
  NumberCHC_DAA = round(overalCHC_DAA*N1579/100);



  logit_rho_cur = logit(rho[1]);
  logit_rho_ex = logit(rho[2]);
  logit_rho_non = logit(rho[3]);
  logit_CHCpi_cur = logit(CHCpi_cur);
  logit_CHCpi_ex = logit(CHCpi_ex);
  logit_CHCpi_non = logit(CHCpi_non);
  logit_HCVclear = logit(HCVclear);
}
```

## Multi-state Markov model

```
#include <stdio.h>
#include <stdlib.h>
#include <string.h>
#include <math.h>
#include <time.h>

#define loops 100
int populationPerYearAndAge[2021][66];
double deathRatePerAge[66];
int totalPopulationPerYear[2021];

FILE *F_DeathRate=fopen("`Country`-deathRates.txt","r");
FILE *F_Population=fopen("`Country`-population.txt","r");
FILE *out;

double pa_start = 0.00012;                     // Setting Variable
double pa_step  = 0.000002;                    // Setting Variable
double pa_stop  = 0.000123;                    // Setting Variable

double pa;
double pg = 1.0/11.7;   // Assumed average active injecting career of  11
.7 years
double pk = 0.004*12.0;     // Relapse rate of 0.004/month

struct people{
    int age;
    int state;
};

char filename[100];
struct people person[50000000];
```

```
long int totalPersons=0;

void setPopulationAge()
{
    for( int j=0; j<71; j++){
        for( int i=0; i<51; i++){
            fscanf(F_Population,"%d",&populationPerYearAndAge[1950+j][15+
i]);
        }
    }
}

void setDeathRate()
{
    for( int i=0; i<51; i++){
        fscanf(F_DeathRate,"%lf",&deathRatePerAge[15+i*5]);
        for( int j=0; j<5; j++){
            deathRatePerAge[15+i*5+j] = deathRatePerAge[15+i*5];
        }
    }
}

void getTotalPopulationPerAge()
{
    for( int j=0; j<71; j++){
        for( int i=0; i<51; i++){
            totalPopulationPerYear[1950+j] += populationPerYearAndAge[195
0+j][15+i];
        }
    }
}
```

```
void printTotalPopulationPerAge()
{
    printf("---- - ----------\n");
    printf("Year - Population\n");
    printf("---- - ----------\n");
    for( int i=1950; i<2021; i++){
        printf("%d - %d\n",i,totalPopulationPerYear[i]);
    }
}

void printDeathRatePerAge()
{
    printf("--- - -----\n");
    printf("Age - Rate\n");
    printf("--- - -----\n");
    for( int i=15; i<65; i++){
        printf("%d - %lf\n",i,deathRatePerAge[i]);
    }
}

void printTotalPersonPerState( int year)
{
    int count[4]= {0,0,0,0};
    for( int i=0; i<totalPersons; i++){
        count[person[i].state]++;
    }
    //printf("%d\t%ld\t%ld\t%ld\t%ld\n",year,count[0],count[1],count[2],c
ount[3]);
    if(year==2014)
        printf("%d\t%ld\t%ld\t%ld\t%ld\t%lf\n",year,count[0],count[1],cou
nt[2],count[3],pa);
    if( year>2009){
```

```
        fprintf(out, "%d\t%ld\t%ld\t%ld\t%ld\n",year,count[0],count[1],co
unt[2],count[3]);
    }
}

void initializePopulation()
{
    int cnt=0;
    for( int j=15; j<65; j++){
        for( int i=0; i<populationPerYearAndAge[1950][j]*100; i++){
            person[cnt].age=j;
            person[cnt].state=0;
            cnt++;
        }
    }
    totalPersons = cnt;
}

bool checkRate(double rate)
{
    if (rate <0.001){
        double k = rand()/(1.00+RAND_MAX);
        if(k<0.001){
            rate = rate*1000;
        }
        else{
            return false;
        }
    }
    double k = rand()/(1.00+RAND_MAX);
    if ( k < rate )
        return true;
```

```
    else
        return false;
}

void changeStatusAndAge()
{
    for( int i=0; i<totalPersons; i++){
        // change state
        double k = rand()/(1.00+RAND_MAX);
        if( person[i].state==0){
            if ( checkRate(pa) &&  person[i].age<40){
                person[i].state=1;
            }
        }
        else if( person[i].state==1){
            if ( k < pg ){
                person[i].state=2;
            }
        }
        else if( person[i].state==2){
            if ( k < pk ){
                person[i].state=1;
            }
        }
        // After their 49th year, all active PWID are assumed to cease in
jecting
        if(person[i].age>49 && person[i].state==1){
            person[i].state=2;
        }
        // After their 64th year, remove
        if(person[i].age>64){
            person[i].state=3;
```

```
        }
        // death rate depending on age and state
        k = rand()/(1.00+RAND_MAX);
        if( person[i].state==0){
            if ( k < deathRatePerAge[person[i].age] ){
                person[i].state=3;
            }
        }
        else if( person[i].state==1){
            if ( k < deathRatePerAge[person[i].age] * 13.0 ){
                person[i].state=3;
            }
        }
        else if( person[i].state==2){
            if ( k < deathRatePerAge[person[i].age] ){
                person[i].state=3;
            }
        }
        // increase age one year
        person[i].age++;
    }
}

void addNewPersons(int year)
{
    // add new person [15 years old and non PWID]
    int cnt=totalPersons;
    for( int i=0; i<populationPerYearAndAge[year+1][15]*100; i++){
// 100 instead of 1000 for the population to reduce execution time
        person[cnt].age=15;
        person[cnt].state=0;
        cnt++;
```

```
    }
    // increase the total number of population
    totalPersons = cnt;
}
int main()
{
    srand(time(NULL));

    setPopulationAge();
    setDeathRate();

    getTotalPopulationPerAge();
    pa = pa_start;
    while ( pa < pa_stop){

        snprintf(filename, 100, "result_%lf.txt",pa);
        out=fopen(filename,"w");

        for( int iter=0; iter<loops ;iter++){
            initializePopulation();
            for( int year=1950; year<2020; year++){
                printTotalPersonPerState(year);
                changeStatusAndAge();
                addNewPersons(year);
            }
        }

        fclose(out);
        pa = pa + pa_step;
    }
```

```
}
```

# REFERENCES

European Centre for Disease Prevention and Control. 2016. "Epidemiological Assessment of Hepatitis b and c Among Migrants in the EU/EEA." https://www.ecdc.europa.eu/sites/default/files/media/en/publications/Publications/epidemiological-assessment-hepatitis-B-and-C-among-migrants-EU-EEA.pdf.

European Centre for Disease Prevention and Control (ECDC). 2016. "Systematic Review on Hepatitis b and c Prevalence in the EU/EEA." https://www.ecdc.europa.eu/sites/default/files/media/en/publications/Publications/systematic-review-hepatitis-B-C-prevalence.pdf.

———. 2021. "Hepatitis c Prevalence Database." https://www.ecdc.europa.eu/en/all-topics-z/hepatitis-c/tools/hepatitis-c-prevalence-database.

Graf, Christiana, Marcus M Mücke, Georg Dultz, Kai-Henrik Peiffer, Alica Kubesch, Patrick Ingiliz, Stefan Zeuzem, Eva Herrmann, and Johannes Vermehren. 2019. "Efficacy of Direct-acting Antivirals for Chronic Hepatitis C Virus Infection in People Who Inject Drugs or Receive Opioid Substitution Therapy: A Systematic Review and Meta-analysis." *Clinical Infectious Diseases* 70 (11): 2355–65. https://doi.org/10.1093/cid/ciz696.

Grebely, Jason, Sarah Larney, Amy Peacock, Samantha Colledge, Janni Leung, Matthew Hickman, Peter Vickerman, et al. 2019. "Global, Regional, and Country-Level Estimates of Hepatitis c Infection Among People Who Have Recently Injected Drugs." *Addiction* 114 (1): 150–66. https://doi.org/https://doi.org/10.1111/add.14393.

Handanagic, Senad, Ivana Bozicevic, Marta Civljak, Zoran Dominkovic, Sandra Sevic, Jelena Barbaric, Tatjana Nemeth Blazic, Oktavija Dakovic Rode, and Josip Begovac. 2016. "HIV and Hepatitis c Prevalence, and Related Risk Behaviours Among People Who Inject Drugs in Three Cities in Croatia: Findings from Respondent-Driven Sampling Surveys." *International Journal of Drug Policy* 32: 57–63. https://doi.org/https://doi.org/10.1016/j.drugpo.2016.04.007.

Hines, Lindsey A., Adam Trickey, Janni Leung, Sarah Larney, Amy Peacock, Louisa Degenhardt, Samantha Colledge, et al. 2020. "Associations Between National Development Indicators and the Age Profile of People Who Inject Drugs: Results from a Global Systematic Review and Meta-

Analysis." *The Lancet Global Health* 8 (1): e76–91. https://doi.org/10.1016/S2214-109X(19)30462-0.

Lampertico, Pietro, Jose A. Carrión, Michael Curry, Juan Turnes, Markus Cornberg, Francesco Negro, Ashley Brown, et al. 2020. "Real-World Effectiveness and Safety of Glecaprevir/Pibrentasvir for the Treatment of Patients with Chronic HCV Infection: A meta-Analysis." *Journal of Hepatology* 72 (6): 1112–21. https://doi.org/10.1016/j.jhep.2020.01.025.

Lin, Lifeng, and Haitao Chu. 2020. "Meta-Analysis of Proportions Using Generalized Linear Mixed Models." *Epidemiology* 31 (5). https://journals.lww.com/epidem/Fulltext/2020/09000/Meta_analysis_of_Proportions_Using_Generalized.16.aspx.

McDonald, Scott A., Rosmawati Mohamed, Maznah Dahlui, Herlianna Naning, and Adeeba Kamarulzaman. 2014. "Bridging the Data Gaps in the Epidemiology of Hepatitis c Virus Infection in Malaysia Using Multi-Parameter Evidence Synthesis." *BMC Infectious Diseases* 14 (1): 564. https://doi.org/10.1186/s12879-014-0564-6.

Micallef, J. M., J. M. Kaldor, and G. J. Dore. 2006. "Spontaneous Viral Clearance Following Acute Hepatitis c Infection: A Systematic Review of Longitudinal Studies." *Journal of Viral Hepatitis* 13 (1): 34–41. https://doi.org/https://doi.org/10.1111/j.1365-2893.2005.00651.x.

Presanis, Anne M., O. Noel Gill, Timothy R. Chadborn, Caterina Hill, Vivian Hope, Louise Logan, Brian D. Rice, Valerie C. Delpech, A. E. Ades, and Daniela De Angelis. 2010. "Insights into the Rise in HIV Infections, 2001 to 2008: A Bayesian Synthesis of Prevalence Evidence." *AIDS* 24 (18). https://journals.lww.com/aidsonline/Fulltext/2010/11270/Insights_into_the_rise_in_HIV_infections,_2001_to.12.aspx.

Sweeting, M. J., D. De Angelis, M. Hickman, and A. E. Ades. 2008. "Estimating hepatitis C prevalence in England and Wales by synthesizing evidence from multiple data sources. Assessing data conflict and model fit." *Biostatistics* 9 (4): 715–34. https://doi.org/10.1093/biostatistics/kxn004.

Tan, Sarah, Susanna Makela, Daliah Heller, Kevin Konty, Sharon Balter, Tian Zheng, and James H. Stark. 2018. "A Bayesian Evidence Synthesis Approach to Estimate Disease Prevalence in Hard-to-Reach Populations: Hepatitis c in New York City." *Epidemics* 23: 96–109. https://doi.org/https://doi.org/10.1016/j.epidem.2018.01.002.

Thein, Hla-Hla, Qilong Yi, Gregory J. Dore, and Murray D. Krahn. 2008. "Estimation of Stage-Specific Fibrosis Progression Rates in Chronic Hepatitis c Virus Infection: A Meta-Analysis and Meta-Regression." *Hepatology* 48 (2): 418–31. https://doi.org/https://doi.org/10.1002/hep.22375.

Veen, Maaike G. van, Anne M. Presanis, Stefano Conti, Maria Xiridou, Annemarie R. Stengaard, Martin C. Donoghoe, Ard I. van Sighem, Marianne A. van der Sande, and Daniela De Angelis. 2011. "National Estimate of HIV Prevalence in the Netherlands: Comparison and Applicability of Different Estimation Tools." *AIDS* 25 (2). https://journals.lww.com/aidsonline/Fulltext/2011/01140/National_estimate_of_HIV_prevalence_in_the.14.aspx.

Vilibic-Cavlek, Tatjana, Jasmina Kucinar, Suncanica Ljubin-Sternak, Bernard Kaic, Lorena Lazaric-Stefanovic, and Branko Kolaric. 2014. "Prevalence of Viral Hepatitis in Croatian Adult Population Undergoing Routine Check-up, 2010-2011." *Central European Journal of Public Health* 22 (1): 29–33. https://doi.org/10.21101/cejph.a3844.

# Estimating the prevalence of chronic hepatitis C infection (CHC) in Cyprus using Bayesian multiparameter evidence synthesis

11/03/2022

## Table of Contents

Introduction ........ 2

Methods ........ 2

Prevalence of ex-PWID ........ 3

Prevalence of recent PWID ........ 3

Prevalence of CHC among recent PWID ........ 4

Prevalence of CHC among ex-PWID ........ 4

Prevalence of CHC among non-PWID ........ 5

Sensitivity analysis including migrants from endemic countries ........ 6

Results ........ 6

Tables and Figures ........ 8

Appendix ........ 12

Fit of the multi-state Markov model ........ 12

Stan code for Bayesian multiparameter evidence synthesis ........ 13

Multi-state Markov model ........ 18

References ........ 26

# INTRODUCTION

Patients with chronic hepatitis C (HCV) infection often remain asymptomatic for decades and even for life, but a proportion of them develop active chronic hepatitis, which is a progressive disease (Thein et al. 2008). Because of the largely asymptomatic nature of HCV infection, notification data reflect national screening and testing practices and do not give accurate insights into the prevalence of HCV infection. Although there are studies that have estimated the prevalence of HCV antibodies (anti-HCV) in specific groups such as the general population, first-time blood donors, or people who inject drugs (PWID) at drug treatment centers, those alone cannot be combined to produce national estimates unless some additional information regarding the composition of each HCV risk group and its prevalence in the population are known (Sweeting et al. 2008; McDonald et al. 2014; Tan et al. 2018).

# METHODS

Bayesian multiparameter evidence synthesis (BMES) has been a popular approach to formally estimate anti-HCV or HIV prevalence (Sweeting et al. 2008; McDonald et al. 2014; Tan et al. 2018; Presanis et al. 2010; Veen et al. 2011). In this project, we extend this method to estimating the CHC prevalence, with the population (15-79 years) split into three main non-overlapping risk groups, i.e., recent PWID (those who have injected in the last year), ex-PWID, and non-PWID. A unified model is assumed including parameters associated with the CHC prevalence of recent, ex-PWID, and non-PWID (denoted by $\pi_{rec}$, $\pi_{ex}$, and $\pi_{non}$, respectively) and the prevalence of these risk groups in the population (denoted by $\rho_{rec}$, $\rho_{ex}$, and $\rho_{non}$, respectively; $\rho_{rec} + \rho_{ex} + \rho_{non} = 1$). To estimate the CHC prevalence in the whole population, $\pi$, we used the formula:

$$\pi = \pi_{rec}\rho_{rec} + \pi_{ex}\rho_{ex} + \pi_{non}\rho_{non} \quad (1)$$

Bayesian synthesis considers all available data, allowing for an evidence-based estimation of CHC prevalence in the population, with inherent uncertainty properly accounted for. Our aim is to estimate the CHC prevalence for the total (15-79) population of Cyprus in 2019 using sources of information over the last decade (2010-2019).

## Prevalence of ex-PWID

Data on the prevalence of ex-PWID are sparse and generally often unreliable. To overcome this limitation, we apply the method proposed by McDonald et al. (2014), which requires simulating a multi-state Markov model representing the non-PWID, recent PWID, and ex-PWID. In the multi-state Markov model, the simulation starts in 1950 and examines the population aged 15-79 years old. A certain number of 15-year-old individuals enter the simulation process each year. These numbers of 15-year-old individuals are obtained from the United Nations Statistic Division UNSD, being different for each country. The ageing of the population is also taken into account. Country-specific overall mortality data are retrieved from the Life Tables of the World Health Organization WHO. The duration of injecting career is also country-specific and obtained from the paper of Hines et al. (2020).

To estimate the number of ex-PWID, the model can be calibrated on the number of recent PWID provided in the EMCDDA barometer or the EMCDDA statistical bulletin (the code of the Markov model can be found in the Appendix). If there are no available data in the barometer, estimates from the systematic review of Grebely et al. (2019) could be used. Alternatively, if the national focal points suggest or provide different and updated or more accurate data for calibration purposes, we will consider their advice and adjust the model accordingly.

After applying the model for Cyprus, we compute the number (and the corresponding Confidence Interval - CI) of ex-PWID in 2019. The number of ex-PWID is then divided by the population size (15-79 years) in the same year, and a Normal distribution, constrained such that $\rho_{ex} \in (0,1)$, is assumed for $\rho_{ex}$. The standard deviation of this distribution is specified to approximately correspond to the respective CI, i.e. $\left(UB_{\rho_{ex}} - LB_{\rho_{ex}}\right)/(2 \times 1.96)$, where $UB_{\rho_{ex}}$ and $LB_{\rho_{ex}}$ denote the upper and lower limit of the CI.

## Prevalence of recent PWID

The number of recent PWID in Cyprus in 2019 is also projected by the multi-state Markov model described in the previous subsection. To estimate $\rho_{rec}$, the number of recent PWID is divided by the population size (15-79 years). Similar to the previous subsection, a Normal distribution, constrained such that $\rho_{rec} \in (0,1)$, is assumed for $\rho_{rec}$, with the standard deviation specified to correspond to the CI obtained from the multi-state Markov model.

## Prevalence of CHC among recent PWID

The prevalence (and the 95% CI) of CHC among recent PWID can be informed from studies in the EMCDDA statistical bulletin. If information on the CHC prevalence is available, the Binomial distribution in the model to inform $\pi_{rec}$ was used. However, as these EMCDDA data typically refer to the anti-HCV prevalence and not to the CHC prevalence, they could not be used directly. This issue is addressed by noting that spontaneous viral clearance occurs in approximately one in four people with acute hepatitis C (Micallef, Kaldor, and Dore 2006). To account for the variability of the HCV clearance probabilities, we used the result reported in Micallef, Kaldor, and Dore (2006), i.e., the proportion of HCV clearance (denoted by $\rho_{clear}$) is equal to 0.26 (95% CI 0.22–0.29). Thus, estimates of the CHC prevalence among recent PWID ($\pi_{rec}$) can be obtained using the formula $\pi_{rec} = \pi(\text{anti-HCV})_{rec}(1 - \rho_{clear})$, where $\pi(\text{anti-HCV})_{rec}$ denotes the anti-HCV prevalence among recent PWID.

Apart from spontaneous clearance, some people may have been treated with direct acting antivirals (DAAs). Since treatment data may be weak and unstandardised or unavailable for most EU/EEA countries, we have not formally taken treatment into account.

If information on the HCV prevalence (CHC or anti-HCV) among recent PWID in the EMCDDA database is not available, the required information is obtained from the paper of Grebely et al. (2019). Currently, CHC prevalence estimates from the paper of Grebely et al. (2019) were used. If the national focal point recommends updated formal estimates, the model input could be adjusted accordingly.

## Prevalence of CHC among ex-PWID

Information on CHC prevalence among ex-PWID (i.e., $\pi_{ex}$) is difficult to obtain directly. To overcome this, nationwide data on HCV prevalence among ever users through the EMCDDA database was utilized. However, if these data refer to the anti-HCV prevalence, they are adjusted according to the procedure described in the previous subsection, i.e. $\pi_{ever} = \pi(\text{anti-HCV})_{ever}(1 - \rho_{clear})$, where $\pi(\text{anti-HCV})_{ever}$ denotes the anti-HCV prevalence among ever PWID. In any case, if an estimate of $\pi_{ever}$ is available, $\pi_{ex}$ can be indirectly estimated since $\pi_{ever}$ is equal to a weighted average of $\pi_{rec}$ and $\pi_{ex}$, as shown by the following formula

$$\pi_{ever} = \frac{\rho_{rec}}{\rho_{rec} + \rho_{ex}}\pi_{rec} + \frac{\rho_{ex}}{\rho_{rec} + \rho_{ex}}\pi_{ex}, \quad (2)$$

or, equivalently,

$$\pi_{ex} = \left(\pi_{ever} - \frac{\rho_{rec}}{\rho_{rec} + \rho_{ex}}\pi_{rec}\right) \times \frac{\rho_{rec} + \rho_{ex}}{\rho_{ex}}, \quad (3)$$

Currently, national anti-HCV prevalence data from ever PWID in 2019, available in the EMCDDA statistical bulletin, were used. In any case, the model could be updated with any other relevant study/information suggested by the national focal point.

## Prevalence of CHC among non-PWID

To estimate the CHC prevalence among non-PWID in a country (after 2010), the ECDC database (European Centre for Disease Prevention and Control (ECDC) 2021) was used. The ECDC group has carefully and critically collected information from relevant studies across the EU/EEA region in cooperation with the focal points of each country. On the basis of the representativeness of each study and other factors (European Centre for Disease Prevention and Control (ECDC) 2016), a risk of bias score was assigned to each study, with higher values indicating higher-quality studies. When higher-quality studies are available in a country (≥ 4), they are used to estimate the overall CHC prevalence in that country among non-PWID. If there are no higher-quality studies, general population estimates with a lower quality (<4) are pooled. If no general population prevalence estimates are available, data on the prevalence of HCV in pregnant women could be used as a prevalence measure. Finally, if data on pregnant women are lacking too, first-time blood donor studies could be an option. However, if the national focal point agrees or recommends, estimates from a neighboring country could be used instead, or we could use any other relevant information provided/suggested by the national focal point.

We directly use CHC prevalence data from studies in the general population, if available. If the data in the general population refer to anti-HCV prevalence (without any data on the viremic population), we adjust the estimates to get CHC prevalence based on the spontaneous HCV clearance estimate of 26%, described in the previous subsection.

As $\rho_{rec}$ and $\rho_{ex}$ are likely to be low, $\pi_{non}$ will be the most critical factor for the estimation of the overall CHC prevalence in the population. To adjust for potential heterogeneity between the CHC prevalence estimates from different studies in the non-PWID population in a country, as a sensitivity analysis, we also apply a random-effect meta-analytic approach to pool the CHC prevalence across studies on non-PWID (Lin and Chu 2020). The random-effect approach is carried out only when at least 3 studies are available in the general population of a certain country, and the estimates are compared with the corresponding estimates from the approach assuming no heterogeneity (similar to a fixed-effect meta-analysis). If the estimates are inconsistent, possible explanations are investigated and discussed. If there is substantial heterogeneity between studies, it would help more to explore the causes; for example, following national focal point suggestions, some studies could be excluded to reduce heterogeneity.

## Sensitivity analysis including migrants from endemic countries

A sensitivity analysis including data for the viremic population among migrants based on the report of the ECDC group (European Centre for Disease Prevention and Control 2016) was carried out. Migrants from endemic countries represent 18.2% of the adult population in Cyprus (Table 8 in European Centre for Disease Prevention and Control (2016)), with the respective CHC prevalence being equal to $\pi_{mig} = 2.1\%$ (Table 9 in European Centre for Disease Prevention and Control (2016)). The overall CHC prevalence is now estimated by

$$\pi = \rho_{rec}\pi_{rec} + \pi_{ex}\rho_{ex} + \pi_{mig}\rho_{mig} + \pi_{non}\rho_{non},$$

where $\rho_{rec} + \rho_{ex} + \rho_{mig} + \rho_{non} = 1$.

# RESULTS

The above-mentioned approach was used to estimate the prevalence of CHC in 2019 (considering ages between 15-79 years: 702,682).

The aggregated data used by our approach are briefly presented in Table 1. The multi-state Markov model to estimate the prevalence of recent and ex-PWID was calibrated on the estimates reported in in the EMCDDA barometer. In Cyprus, there were 2 studies on first time blood donors, which included only anti-HCV data. To estimate the CHC prevalence in the general population (primarily non-PWID), we used a fixed-effect meta-analytic approach, with the

corresponding results presented in Table 2. The prevalence of recent and ex-PWID was low in Cyprus (about 0.09% and 0.2%, respectively) corresponding to 630 (95% CI: 500-800) recent PWID and 1,400 (95% CI: 1,150-1,650) ex-PWID in the population. However, the CHC prevalence in these groups was substantial (Table 2) being 37.28% and 41.09%, respectively. This translates to 235 (95% CI: 175-300) and 572 (95% CI: 400-763) recent and ex-PWID aged 15-79 living with CHC infection in Cyprus in 2019. The CHC prevalence in the general population was 0.08% (95% CI: 0.04%-0.13%), much lower than that of the high-risk groups. Taking all pieces of information into account, the overall CHC prevalence in Cyprus in 2019 was equal to 0.19% (95% CI: 0.15%-0.25%), which corresponds to 1,353 (95% CI: 1,035-1,756) individuals aged 15-79 years with CHC infection.

The results from our model including migrants from endemic countries as a separate group are presented in Table 3. However, this analysis comes with possible limitations; that is, including migrants as a separate group is valid only if migrants do not overlap with the remaining groups (recent PWID, ex-PWID, and non-PWID). Thus, if migrants do participate proportionally in the study(ies) in the general population, this analysis may result in biased overall CHC estimates (most probably in higher CHC prevalence estimates). However, if the national focal points consider that including migrants as a separate group is valid, we could consider results in Table 3 as the main analysis.

# TABLES AND FIGURES

*Table 1. Data (real) contributing to the estimation of CHC prevalence in Cyprus in 2019.*

| Parameter | Estimate (95%CI) | Number (95%CI) | Numerator | Denominator | Notes | Year of study |
|---|---|---|---|---|---|---|
| $\rho_{rec}$ | | 630 (500-800) | | | Method based on McDonald et al. | 2019 |
| $\rho_{ex}$ | | 1,400 (1,150-1,650) | | | Method based on McDonald et al. | 2019 |
| $\pi_{rec}$ | 37.3% (32.9%-41.8%) | | | | Grebely et al. | 2011-2014 |
| $\pi(\text{anti-HCV})_{ever}$ | | | 55 | 102 | EMCDDA database | 2019 |
| $\pi(\text{anti-HCV})_{non}$ | | | 11 | 10,959 | ECDC database (first-time blood donors) | 2013 |
| $\pi(\text{anti-HCV})_{non}$ | | | 1 | 1,185 | ECDC database (first-time blood donors) | 2016 |

*Notes*: Higher risk of bias score denotes a higher-quality study (range from 0 to 6);

*Table 2. Results from the method assuming no heterogeneity in the estimation of the CHC prevalence in the general population from different studies (i.e., similar to a fixed-effect meta-analysis).*

| Parameter | Posterior median | Posterior sd | LB (2.5%) | UB (97.5%) |
|---|---|---|---|---|
| $\rho_{rec}$ (%) | 0.09 | 0.01 | 0.07 | 0.11 |
| $\rho_{ex}$ (%) | 0.2 | 0.02 | 0.16 | 0.23 |
| $\pi_{rec}$ (%) | 37.28 | 2.26 | 32.9 | 41.71 |
| $\pi_{ex}$ (%) | 41.09 | 5.48 | 30.38 | 51.76 |
| $\pi_{non}$ (%) | 0.08 | 0.02 | 0.04 | 0.13 |
| $\pi$ (%) | 0.19 | 0.03 | 0.15 | 0.25 |
| Number with CHC | 1,353 | 184 | 1,035 | 1,756 |
| Pr(Ever PWID\|CHC) (%) | 59.79 | 7.12 | 46.03 | 73.76 |
| Pr(Non-PWID\|CHC) (%) | 40.21 | 7.12 | 26.24 | 53.97 |

*Notes*: $\rho_{rec}$, prevalence of recent PWID; $\rho_{ex}$, prevalence of ex-PWID; $\pi_{rec}$, CHC prevalence among recent PWID; $\pi_{ex}$, CHC prevalence among ex-PWID; $\pi_{non}$, CHC prevalence among non-PWID; $\pi$ overall CHC prevalence in Cyprus; LB, Lower Boundary; UP, Upper Boundary

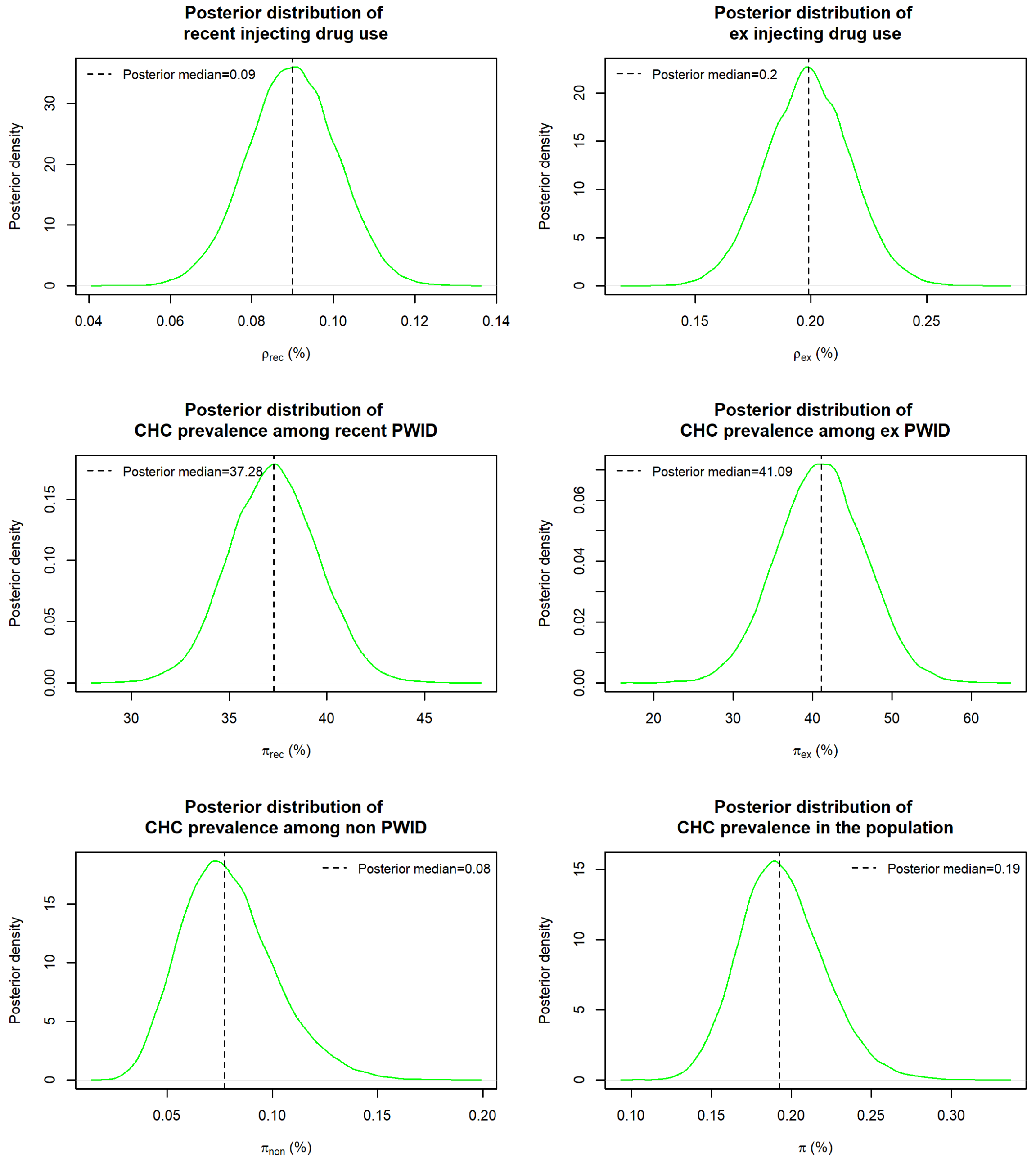


*Figure 1. Posterior distributions of the key model parameters using the approach that assumes no heterogeneity in the estimation of the CHC prevalence in the general population (primarily non-PWID) from different studies (i.e., similar to a fixed-effect meta-analysis).*

*Table 3. Results from the method assuming no heterogeneity in the estimation of the CHC prevalence in the general population (primarily non-PWID) from different studies (i.e., similar to a fixed-effect meta-analysis) including migrants from endemic countries as a separate group.*

| Parameter | Posterior median | Posterior sd | LB (2.5%) | UB (97.5%) |
|---|---|---|---|---|
| $\rho_{rec}$ (%) | 0.09 | 0.01 | 0.07 | 0.11 |
| $\rho_{ex}$ (%) | 0.2 | 0.02 | 0.16 | 0.23 |
| $\rho_{mig}$ (%) | 18.2 | 0 | 18.2 | 18.2 |
| $\pi_{rec}$ (%) | 37.3 | 2.28 | 32.86 | 41.78 |
| $\pi_{ex}$ (%) | 41.09 | 5.56 | 30.22 | 51.83 |
| $\pi_{mig}$ (%) | 2.1 | 0.34 | 1.44 | 2.77 |
| $\pi_{non}$ (%) | 0.08 | 0.02 | 0.04 | 0.13 |
| $\pi$ (%) | 0.56 | 0.07 | 0.43 | 0.69 |
| Number with CHC | 3,952 | 463 | 3,050 | 4,866 |
| Pr(Ever PWID\|CHC) (%) | 20.43 | 3.16 | 15.22 | 27.61 |
| Pr(Mig\|CHC) (%) | 68.11 | 4.57 | 57.65 | 75.82 |
| Pr(Non-PWID\|CHC) (%) | 11.27 | 3.09 | 6.32 | 18.28 |

*Notes*: $\rho_{rec}$, prevalence of recent PWID; $\rho_{ex}$, prevalence of ex-PWID; $\rho_{mig}$, prevalence of migrants from endemic countries; $\pi_{rec}$, CHC prevalence among recent PWID; $\pi_{ex}$, CHC prevalence among ex-PWID; $\pi_{mig}$, CHC prevalence among migrants from endemic countries; $\pi_{non}$, CHC prevalence among non-PWID; $\pi$ overall CHC prevalence in Cyprus; LB, Lower Boundary; UP, Upper Boundary

# APPENDIX

## Fit of the multi-state Markov model

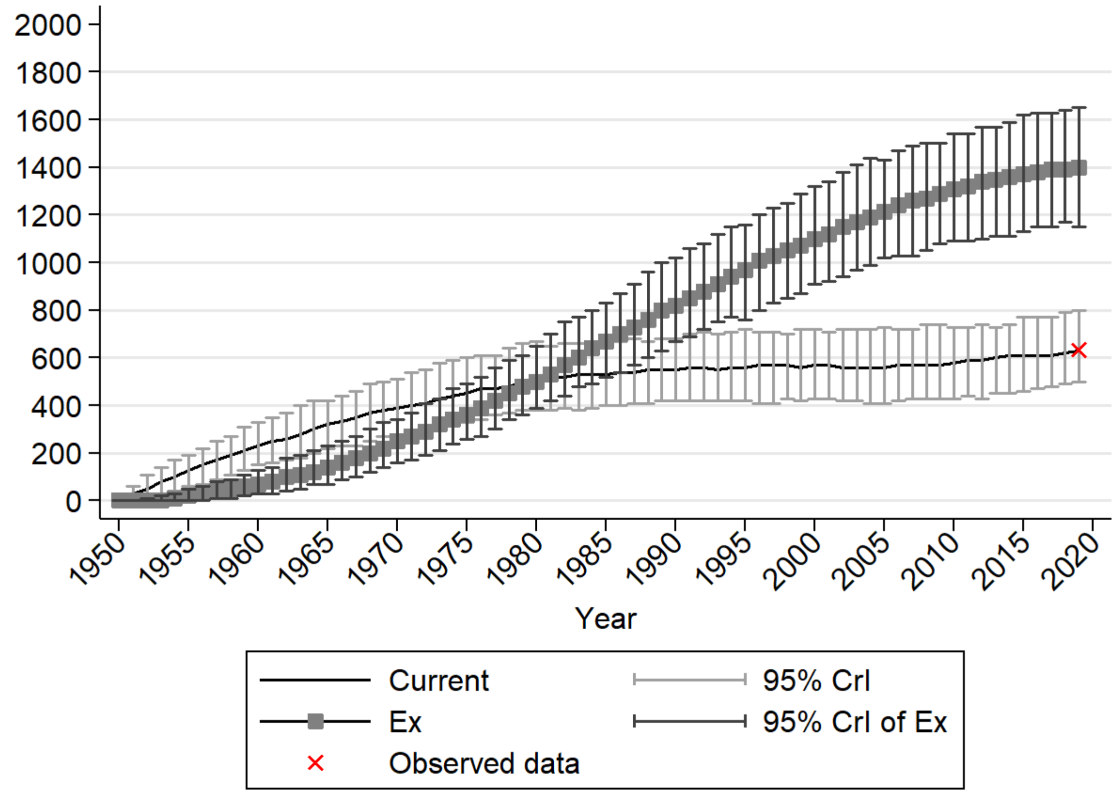


*Figure 2: Model predictions for the numbers of current and ex-PWID (people who inject drugs). The solid black line and shaded grey error bars show the median and 95% credible intervals (95% CrI) for the model projections. For comparison, asterisks indicate the observed country's data.*

## Stan code for Bayesian multiparameter evidence synthesis

```
data {
  int<lower=0> N1580; // Population of 15-64 in `Country`
  int<lower=1> Knon; // Number of studies in the ECDC data for `Country`
  int<lower=1> Kever; // Number of studies in the EMCDDA data for ever us
ers in `Country`
  int<lower=1> Kcur; // Number of studies in the EMCDDA barometer (for HC
V prevalence of PWID) in `Country`

  real p_cur_mean; // Prior mean for the prevalence of current use in `Co
untry`
  real<lower=0> p_cur_sd; // Prior sd for the prevalence of current use i
n `Country`

  real p_ex_mean; // Prior mean for the prevalence of ex-use in `Country`
  real<lower=0> p_ex_sd; // Prior sd for the prevalence of ex-use in `Cou
ntry`

  //int<lower=0> Nst_CHC_cur[Kcur]; // Number of individuals in the study
estimating chronic HCV among recent PWID
  //int<lower=0> Yst_CHC_cur[Kcur]; // Number of HCV+ in the study estima
ting chronic HCV among recent PWID
  real p_CHC_cur_mean; // Prior mean for the CHC prevalence among recent
PWID in `Country`
  real<lower=0> p_CHC_cur_sd; // Prior sd for the CHC prevalence among re
cent PWID in `Country`

  int<lower=0> Nst_hcv_ever[Kever]; // Number of individuals in the study
estimating HCV among ever IDU
  int<lower=0> Yst_hcv_ever[Kever]; // Number of HCV+ in the study estima
ting HCV among ever PWID
```

```
  int<lower=0> Nst_hcv_non[Knon]; // Number of individuals in the study e
stimating anti-HCV among non PWID
  int<lower=0> Yst_hcv_non[Knon]; // Number of HCV+ in the study estimati
ng anti-HCV among non PWID

  vector<lower=0>[3] alpha; // parameter of the Diriclet prior

  real HCVclear_mean; // Prior mean for the HCV clearance probability
  real<lower=0> HCVclear_sd; // Prior sd for the HCV clearance probabilit
y
}

// Block defining the original parameters
parameters {
  // The parameters to be sampled
  simplex[3] rho; // Prevalence of the three risk groups
  real<lower=0,upper=1> CHCpi_cur; // P(CHC+|Cur)
  real<lower=0,upper=1> CHCpi_ex; // P(CHC+|Ex)
  real<lower=0,upper=1> CHCpi_non; // P(CHC+|Non)

  real<lower=0,upper=1-CHCpi_cur> HCVclear; // Probability of HCV clearan
ce; upper bound = 1-prevalence of chronic HCV
}


transformed parameters {
  // Change scales
  real<lower=0,upper=1> rho_ever;
  real<lower=0,upper=1> CHCpi_ever;
  real<lower=0,upper=1> pi_ever;
  real<lower=0,upper=1> pi_non;
```

```
  rho_ever = rho[1] + rho[2];
  CHCpi_ever = CHCpi_cur*rho[1]/rho_ever + CHCpi_ex*rho[2]/rho_ever;
  pi_ever = CHCpi_ever/(1-HCVclear);
  pi_non = CHCpi_non/(1-HCVclear);
}


// Binomial regression model
model {
  // Priors
  rho ~ dirichlet(alpha);

  //////////////////////////////
  // Likelihood contributions //
  //////////////////////////////

  // Probability of HCV clearance
  HCVclear ~ normal(HCVclear_mean,HCVclear_sd);

  // Prevalence of current use
  rho[1] ~ normal(p_cur_mean,p_cur_sd);

  // Prevalence of ex-use
  rho[2] ~ normal(p_ex_mean,p_ex_sd);

  // Prevalence of chronic HCV among current users
  //Yst_CHC_cur ~ binomial(Nst_CHC_cur,CHCpi_cur);
  CHCpi_cur ~ normal(p_CHC_cur_mean,p_CHC_cur_sd);

  // Prevalence of HCV among ever users
  Yst_hcv_ever ~ binomial(Nst_hcv_ever,pi_ever);
```

```
  // HCV+ among non
  Yst_hcv_non ~ binomial(Nst_hcv_non,pi_non);
}


generated quantities {
  // Functions of parameters
  real<lower=0,upper=100> overalCHC;
  real logit_CHCpi_cur;
  real logit_CHCpi_ex;
  real logit_CHCpi_non;
  real logit_rho_cur;
  real logit_rho_ex;
  real logit_rho_non;
  real<lower=0,upper=1> pEverGivenCHC;
  real<lower=0,upper=1> pNonGivenCHC;
  real logit_HCVclear;
  real<lower=0> NumberCHC;

  // Overall HCV prevalence
  overalCHC = 100*(rho[1]*CHCpi_cur + rho[2]*CHCpi_ex +  rho[3]*CHCpi_non
);
  pEverGivenCHC = CHCpi_ever*rho_ever/(overalCHC/100);
  pNonGivenCHC = CHCpi_non*(1-rho_ever)/(overalCHC/100);
  NumberCHC = round(overalCHC*N1580/100);

  logit_rho_cur = logit(rho[1]);
  logit_rho_ex = logit(rho[2]);
  logit_rho_non = logit(rho[3]);
  logit_CHCpi_cur = logit(CHCpi_cur);
  logit_CHCpi_ex = logit(CHCpi_ex);
  logit_CHCpi_non = logit(CHCpi_non);
```

```
  logit_HCVclear = logit(HCVclear);
}
```

## Multi-state Markov model

```
#include <stdio.h>
#include <stdlib.h>
#include <string.h>
#include <math.h>
#include <time.h>

#define loops 100
int populationPerYearAndAge[2021][66];
double deathRatePerAge[66];
int totalPopulationPerYear[2021];

FILE *F_DeathRate=fopen("greece-deathRates.txt","r");
FILE *F_Population=fopen("greece-population.txt","r");
FILE *out;

double pa_start = 0.00012;                          // Setting Variable
double pa_step  = 0.000002;                         // Setting Variable
double pa_stop  = 0.000123;                         // Setting Variable

double pa;
double pg = 1.0/11.7;    // Assumed average active injecting career of  11
.7 years
double pk = 0.004*12.0;      // Relapse rate of 0.004/month

struct people{
    int age;
    int state;
};

char filename[100];
struct people person[50000000];
```

```
long int totalPersons=0;

void setPopulationAge()
{
    for( int j=0; j<71; j++){
        for( int i=0; i<51; i++){
            fscanf(F_Population,"%d",&populationPerYearAndAge[1950+j][15+
i]);
        }
    }
}

void setDeathRate()
{
    for( int i=0; i<51; i++){
        fscanf(F_DeathRate,"%lf",&deathRatePerAge[15+i*5]);
        for( int j=0; j<5; j++){
            deathRatePerAge[15+i*5+j] = deathRatePerAge[15+i*5];
        }
    }
}

void getTotalPopulationPerAge()
{
    for( int j=0; j<71; j++){
        for( int i=0; i<51; i++){
            totalPopulationPerYear[1950+j] += populationPerYearAndAge[195
0+j][15+i];
        }
    }
}
```

```
void printTotalPopulationPerAge()
{
    printf("---- - ----------\n");
    printf("Year - Population\n");
    printf("---- - ----------\n");
    for( int i=1950; i<2021; i++){
        printf("%d - %d\n",i,totalPopulationPerYear[i]);
    }
}

void printDeathRatePerAge()
{
    printf("--- - -----\n");
    printf("Age - Rate\n");
    printf("--- - -----\n");
    for( int i=15; i<65; i++){
        printf("%d - %lf\n",i,deathRatePerAge[i]);
    }
}

void printTotalPersonPerState( int year)
{
    int count[4]= {0,0,0,0};
    for( int i=0; i<totalPersons; i++){
        count[person[i].state]++;
    }
    //printf("%d\t%ld\t%ld\t%ld\t%ld\n",year,count[0],count[1],count[2],c
ount[3]);
    if(year==2014)
        printf("%d\t%ld\t%ld\t%ld\t%ld\t%lf\n",year,count[0],count[1],cou
nt[2],count[3],pa);
    if( year>2009){
```

```
        fprintf(out, "%d\t%ld\t%ld\t%ld\t%ld\n",year,count[0],count[1],co
unt[2],count[3]);
    }
}

void initializePopulation()
{
    int cnt=0;
    for( int j=15; j<65; j++){
        for( int i=0; i<populationPerYearAndAge[1950][j]*100; i++){
            person[cnt].age=j;
            person[cnt].state=0;
            cnt++;
        }
    }
    totalPersons = cnt;
}

bool checkRate(double rate)
{
    if (rate <0.001){
        double k = rand()/(1.00+RAND_MAX);
        if(k<0.001){
            rate = rate*1000;
        }
        else{
            return false;
        }
    }
    double k = rand()/(1.00+RAND_MAX);
    if ( k < rate )
        return true;
```

```
    else
        return false;
}

void changeStatusAndAge()
{
    for( int i=0; i<totalPersons; i++){
        // change state
        double k = rand()/(1.00+RAND_MAX);
        if( person[i].state==0){
            if ( checkRate(pa) &&  person[i].age<40){
                person[i].state=1;
            }
        }
        else if( person[i].state==1){
            if ( k < pg ){
                person[i].state=2;
            }
        }
        else if( person[i].state==2){
            if ( k < pk ){
                person[i].state=1;
            }
        }
        // After their 49th year, all active PWID are assumed to cease in
jecting
        if(person[i].age>49 && person[i].state==1){
            person[i].state=2;
        }
        // After their 64th year, remove
        if(person[i].age>64){
            person[i].state=3;
```

```
        }
        // death rate depending on age and state
        k = rand()/(1.00+RAND_MAX);
        if( person[i].state==0){
            if ( k < deathRatePerAge[person[i].age] ){
                person[i].state=3;
            }
        }
        else if( person[i].state==1){
            if ( k < deathRatePerAge[person[i].age] * 13.0 ){
                person[i].state=3;
            }
        }
        else if( person[i].state==2){
            if ( k < deathRatePerAge[person[i].age] ){
                person[i].state=3;
            }
        }
        // increase age one year
        person[i].age++;
    }
}

void addNewPersons(int year)
{
    // add new person [15 years old and non PWID]
    int cnt=totalPersons;
    for( int i=0; i<populationPerYearAndAge[year+1][15]*100; i++){
// 100 instead of 1000 for the population to reduce execution time
        person[cnt].age=15;
        person[cnt].state=0;
        cnt++;
```

```
    }
    // increase the total number of population
    totalPersons = cnt;
}
int main()
{
    srand(time(NULL));

    setPopulationAge();
    setDeathRate();

    getTotalPopulationPerAge();
    pa = pa_start;
    while ( pa < pa_stop){

        snprintf(filename, 100, "result_%lf.txt",pa);
        out=fopen(filename,"w");

        for( int iter=0; iter<loops ;iter++){
            initializePopulation();
            for( int year=1950; year<2020; year++){
                printTotalPersonPerState(year);
                changeStatusAndAge();
                addNewPersons(year);
            }
        }

        fclose(out);
        pa = pa + pa_step;
    }
```

```
}
```

# REFERENCES

European Centre for Disease Prevention and Control. 2016. "Epidemiological Assessment of Hepatitis b and c Among Migrants in the EU/EEA." https://www.ecdc.europa.eu/sites/default/files/media/en/publications/Publications/epidemiological-assessment-hepatitis-B-and-C-among-migrants-EU-EEA.pdf.

European Centre for Disease Prevention and Control (ECDC). 2016. "Systematic Review on Hepatitis b and c Prevalence in the EU/EEA." https://www.ecdc.europa.eu/sites/default/files/media/en/publications/Publications/systematic-review-hepatitis-B-C-prevalence.pdf.

———. 2021. "Hepatitis c Prevalence Database." https://www.ecdc.europa.eu/en/all-topics-z/hepatitis-c/tools/hepatitis-c-prevalence-database.

Grebely, Jason, Sarah Larney, Amy Peacock, Samantha Colledge, Janni Leung, Matthew Hickman, Peter Vickerman, et al. 2019. "Global, Regional, and Country-Level Estimates of Hepatitis c Infection Among People Who Have Recently Injected Drugs." *Addiction* 114 (1): 150–66. https://doi.org/https://doi.org/10.1111/add.14393.

Hines, Lindsey A., Adam Trickey, Janni Leung, Sarah Larney, Amy Peacock, Louisa Degenhardt, Samantha Colledge, et al. 2020. "Associations Between National Development Indicators and the Age Profile of People Who Inject Drugs: Results from a Global Systematic Review and Meta-Analysis." *The Lancet Global Health* 8 (1): e76–91. https://doi.org/10.1016/S2214-109X(19)30462-0.

Lin, Lifeng, and Haitao Chu. 2020. "Meta-Analysis of Proportions Using Generalized Linear Mixed Models." *Epidemiology* 31 (5). https://journals.lww.com/epidem/Fulltext/2020/09000/Meta_analysis_of_Proportions_Using_Generalized.16.aspx.

McDonald, Scott A., Rosmawati Mohamed, Maznah Dahlui, Herlianna Naning, and Adeeba Kamarulzaman. 2014. "Bridging the Data Gaps in the Epidemiology of Hepatitis c Virus Infection in Malaysia Using Multi-Parameter Evidence Synthesis." *BMC Infectious Diseases* 14 (1): 564. https://doi.org/10.1186/s12879-014-0564-6.

Micallef, J. M., J. M. Kaldor, and G. J. Dore. 2006. "Spontaneous Viral Clearance Following Acute Hepatitis c Infection: A Systematic Review of Longitudinal Studies." *Journal of Viral Hepatitis* 13 (1): 34–41. https://doi.org/https://doi.org/10.1111/j.1365-2893.2005.00651.x.

Presanis, Anne M., O. Noel Gill, Timothy R. Chadborn, Caterina Hill, Vivian Hope, Louise Logan, Brian D. Rice, Valerie C. Delpech, A. E. Ades, and Daniela De Angelis. 2010. "Insights into the Rise in HIV Infections, 2001 to 2008: A Bayesian Synthesis of Prevalence Evidence." *AIDS* 24 (18). https://journals.lww.com/aidsonline/Fulltext/2010/11270/Insights_into_the_rise_in_HIV_infections,_2001_to.12.aspx.

Sweeting, M. J., D. De Angelis, M. Hickman, and A. E. Ades. 2008. "Estimating hepatitis C prevalence in England and Wales by synthesizing evidence from multiple data sources. Assessing data conflict and model fit." *Biostatistics* 9 (4): 715–34. https://doi.org/10.1093/biostatistics/kxn004.

Tan, Sarah, Susanna Makela, Daliah Heller, Kevin Konty, Sharon Balter, Tian Zheng, and James H. Stark. 2018. "A Bayesian Evidence Synthesis Approach to Estimate Disease Prevalence in Hard-to-Reach Populations: Hepatitis c in New York City." *Epidemics* 23: 96–109. https://doi.org/https://doi.org/10.1016/j.epidem.2018.01.002.

Thein, Hla-Hla, Qilong Yi, Gregory J. Dore, and Murray D. Krahn. 2008. "Estimation of Stage-Specific Fibrosis Progression Rates in Chronic Hepatitis c Virus Infection: A Meta-Analysis and Meta-Regression." *Hepatology* 48 (2): 418–31. https://doi.org/https://doi.org/10.1002/hep.22375.

Veen, Maaike G. van, Anne M. Presanis, Stefano Conti, Maria Xiridou, Annemarie R. Stengaard, Martin C. Donoghoe, Ard I. van Sighem, Marianne A. van der Sande, and Daniela De Angelis. 2011. "National Estimate of HIV Prevalence in the Netherlands: Comparison and Applicability of Different Estimation Tools." *AIDS* 25 (2). https://journals.lww.com/aidsonline/Fulltext/2011/01140/National_estimate_of_HIV_prevalence_in_the.14.aspx.

# Estimating the prevalence of chronic hepatitis C infection (CHC) in Czechia using Bayesian multiparameter evidence synthesis

19/05/2023

## Table of Contents


Introduction ..... 2

Methods ..... 2

- Prevalence of ex-PWID ..... 3
- Prevalence of recent PWID ..... 4
- Prevalence of CHC among recent PWID ..... 4
- Prevalence of CHC among ex-PWID ..... 4
- Prevalence of CHC among non-PWID ..... 5
- Sensitivity analysis including migrants from endemic countries ..... 6

Results ..... 6

Tables and Figures ..... 8

Appendix ..... 12

- Fit of the multi-state Markov model ..... 12
- Stan code for Bayesian multiparameter evidence synthesis ..... 13
- Multi-state Markov model ..... 17

References ..... 25

# INTRODUCTION

Patients with chronic hepatitis C (HCV) infection often remain asymptomatic for decades and even for life, but a proportion of them develop active chronic hepatitis, which is a progressive disease (Thein et al. 2008). Because of the largely asymptomatic nature of HCV infection, notification data reflect national screening and testing practices and do not give accurate insights into the prevalence of HCV infection. Although there are studies that have estimated the prevalence of HCV antibodies (anti-HCV) in specific groups such as the general population, first-time blood donors, or people who inject drugs (PWID) at drug treatment centers, those alone cannot be combined to produce national estimates unless some additional information regarding the composition of each HCV risk group and its prevalence in the population are known (Sweeting et al. 2008; McDonald et al. 2014; Tan et al. 2018).

# METHODS

Bayesian multiparameter evidence synthesis (MPES) has been a popular approach to formally estimate anti-HCV or HIV prevalence (Sweeting et al. 2008; McDonald et al. 2014; Tan et al. 2018; Presanis et al. 2010; Veen et al. 2011). In this project, we extend this method to estimating the CHC prevalence, with the population (15-79 years) split into three main non-overlapping risk groups, i.e., recent PWID (those who have injected in the last year), ex-PWID, and non-PWID. A unified model is assumed including parameters associated with the CHC prevalence of recent, ex-PWID, and non-PWID (denoted by $\pi_{rec}$, $\pi_{ex}$, and $\pi_{non}$, respectively) and the prevalence of these risk groups in the population (denoted by $\rho_{rec}$, $\rho_{ex}$, and $\rho_{non}$, respectively; $\rho_{rec} + \rho_{ex} + \rho_{non} = 1$). To estimate the CHC prevalence in the whole population, $\pi$, we used the formula:

$$\pi = \pi_{rec}\rho_{rec} + \pi_{ex}\rho_{ex} + \pi_{non}\rho_{non} \quad (1)$$

Bayesian synthesis considers all available data, allowing for an evidence-based estimation of CHC prevalence in the population, with inherent uncertainty properly accounted for. Our aim is to estimate the CHC prevalence for the total (15-79) population of Czechia in 2019 using primarily sources of information over the last decade (2010-2019).

## Prevalence of ex-PWID

Data on the prevalence of ex-PWID are sparse and generally often unreliable. To overcome this limitation, we apply the method proposed by McDonald et al. (2014), which requires simulating a multi-state Markov model representing the non-PWID, recent PWID, and ex-PWID. In the multi-state Markov model, the simulation typically starts in 1950 and examines the population aged 15-79 years old. However, based on information provided by the national focal points, the number of PWID before 1989 was particularly low in Czechia. Thus, the simulation started in 1990. A certain number of 15-year-old individuals enter the simulation process each year. These numbers of 15-year-old individuals are obtained from the United Nations Statistic Division UNSD, being different for each country. The ageing of the population is also taken into account. Country-specific overall mortality data are retrieved from the Life Tables of the World Health Organization WHO. The duration of injecting career is also country-specific and obtained from the paper of Hines et al. (2020). However, since no data for Czechia are reported in Hines et al. (2020), we used the average duration of injecting career in Eastern Europe (Hines et al. 2020).

To estimate the number of ex-PWID, the model can be calibrated on the number of recent PWID provided in the EMCDDA barometer or the EMCDDA statistical bulletin (the code of the Markov model can be found in the Appendix). If there are no available data in the barometer, estimates from the systematic review of Grebely et al. (2019) could be used.

After applying the model for Czechia, we compute the number (and the corresponding Confidence Interval - CI) of ex-PWID in 2019. The number of ex-PWID is then divided by the population size (15-79 years) in the same year, and a Normal distribution, constrained such that $\rho_{ex} \in (0,1)$, is assumed for $\rho_{ex}$. The standard deviation of this distribution is specified to approximately correspond to the respective CI, i.e. $\left(UB_{\rho_{ex}} - LB_{\rho_{ex}}\right)/(2 \times 1.96)$, where $UB_{\rho_{ex}}$ and $LB_{\rho_{ex}}$ denote the upper and lower limit of the CI.

## Prevalence of recent PWID

The number of recent PWID in Czechia in 2019 is also projected by the multi-state Markov model described in the previous subsection. To estimate $\rho_{rec}$, the number of recent PWID is divided by the population size (15-79 years). Similar to the previous subsection, a Normal distribution, constrained such that $\rho_{rec} \in (0,1)$, is assumed for $\rho_{rec}$, with the standard deviation specified to correspond to the CI obtained from the multi-state Markov model.

## Prevalence of CHC among recent PWID

The prevalence (and the 95% CI) of CHC among recent PWID can be informed from studies in the EMCDDA statistical bulletin. If information on the CHC prevalence is available, the Binomial distribution in the model to inform $\pi_{rec}$ was used. However, as these EMCDDA data typically refer to the anti-HCV prevalence and not to the CHC prevalence, they could not be used directly. This issue is addressed by noting that spontaneous viral clearance occurs in approximately one in four people with acute hepatitis C (Micallef, Kaldor, and Dore 2006). To account for the variability of the HCV clearance probabilities, we used the result reported in Micallef, Kaldor, and Dore (2006), i.e., the proportion of HCV clearance (denoted by $\rho_{clear}$) is equal to 0.26 (95% CI 0.22–0.29). Thus, estimates of the CHC prevalence among recent PWID ($\pi_{rec}$) can be obtained using the formula $\pi_{rec} = \pi(\text{anti-HCV})_{rec}(1 - \rho_{clear})$, where $\pi(\text{anti-HCV})_{rec}$ denotes the anti-HCV prevalence among recent PWID.

Apart from spontaneous clearance, some people may have been treated with direct acting antivirals (DAAs). Since treatment data may be weak and unstandardised or unavailable for most EU/EEA countries, we have not formally taken treatment into account.

If information on the HCV prevalence (CHC or anti-HCV) among recent PWID in the EMCDDA database is not available, the required information is obtained from the paper of Grebely et al. (2019). Currently, national anti-HCV prevalence data from recent PWID in 2003, available in the EMCDDA statistical bulletin, were used.

## Prevalence of CHC among ex-PWID

Information on CHC prevalence among ex-PWID (i.e., $\pi_{ex}$) is difficult to obtain directly. To overcome this, nationwide data on the HCV prevalence among ever users through the

EMCDDA database was utilized. However, if these data refer to the anti-HCV prevalence, they are adjusted according to the procedure described in the previous subsection, i.e. $\pi_{ever} = \pi(\text{anti-HCV})_{ever}(1-\rho_{clear})$, where $\pi(\text{anti-HCV})_{ever}$ denotes the anti-HCV prevalence among ever PWID. In any case, if an estimate of $\pi_{ever}$ is available, $\pi_{ex}$ can be indirectly estimated since $\pi_{ever}$ is equal to a weighted average of $\pi_{rec}$ and $\pi_{ex}$, as shown by the following formula

$$\pi_{ever} = \frac{\rho_{rec}}{\rho_{rec}+\rho_{ex}}\pi_{rec} + \frac{\rho_{ex}}{\rho_{rec}+\rho_{ex}}\pi_{ex}, \quad (2)$$

or, equivalently,

$$\pi_{ex} = \left(\pi_{ever} - \frac{\rho_{rec}}{\rho_{rec}+\rho_{ex}}\pi_{rec}\right) \times \frac{\rho_{rec}+\rho_{ex}}{\rho_{ex}}, \quad (3)$$

Currently, national data on the anti-HCV prevalence of ever PWID, available in the EMCDDA statistical bulletin, were used.

## Prevalence of CHC among non-PWID

To estimate the CHC prevalence among non-PWID in a country (after 2010), the ECDC database (European Centre for Disease Prevention and Control (ECDC) 2021) was used. The ECDC group has carefully and critically collected information from relevant studies across the EU/EEA region in cooperation with the focal points of each country. On the basis of the representativeness of each study and other factors (European Centre for Disease Prevention and Control (ECDC) 2016), a risk of bias score was assigned to each study, with higher values indicating higher-quality studies. When higher-quality studies are available in a country ($\geq 4$), they are used to estimate the overall CHC prevalence in that country among non-PWID. If there are no higher-quality studies, general population estimates with a lower quality ($<4$) are pooled. If no general population prevalence estimates are available, data on the prevalence of HCV in pregnant women could be used as a prevalence measure. Finally, if data on pregnant women are lacking too, first-time blood donor studies could be an option. However, if the national focal point agrees or recommends, estimates from a neighboring country could be used instead, or we could use any other relevant information provided/suggested by the national focal point.

We directly use CHC prevalence data from studies in the general population, if available. If the data in the general population refer to anti-HCV prevalence (without any data on the viremic population), we adjust the estimates to get CHC prevalence based on the spontaneous HCV clearance estimate of 26%, described in the previous subsection.

As $\rho_{rec}$ and $\rho_{ex}$ are likely to be low, $\pi_{non}$ will be the most critical factor for the estimation of the overall CHC prevalence in the population. To adjust for potential heterogeneity between the CHC prevalence estimates from different studies in the non-PWID population in a country, as a sensitivity analysis, we also apply a random-effect meta-analytic approach to pool the CHC prevalence across studies on non-PWID (Lin and Chu 2020). The random-effect approach is carried out only when at least 3 studies are available in the general population of a certain country, and the estimates are compared with the corresponding estimates from the approach assuming no heterogeneity (similar to a fixed-effect meta-analysis). If the estimates are inconsistent, possible explanations are investigated and discussed. If there is substantial heterogeneity between studies, it would help more to explore the causes; for example, following national focal point suggestions, some studies could be excluded to reduce heterogeneity.

## Sensitivity analysis including migrants from endemic countries

A sensitivity analysis including data for the viremic population among migrants based on the report of the ECDC group (European Centre for Disease Prevention and Control 2016) was carried out. Migrants from endemic countries represent 3.7% of the adult population in Czechia (Table 8 in European Centre for Disease Prevention and Control (2016)), with the respective CHC prevalence being equal to $\pi_{mig} = 1.8\%$ (Table 9 in European Centre for Disease Prevention and Control (2016)). The overall CHC prevalence is now estimated by

$$\pi = \pi_{rec}\rho_{rec} + \pi_{ex}\rho_{ex} + \pi_{mig}\rho_{mig} + \pi_{non}\rho_{non},$$

where $\rho_{rec} + \rho_{ex} + \rho_{mig} + \rho_{non} = 1$.

# RESULTS

The above-mentioned approach was used to estimate the prevalence of CHC in 2019 (considering ages between 15-79 years: 8,523,833).

The aggregated data used by our approach are briefly presented in Table 1. The multi-state Markov model was calibrated on the estimated prevalence of recent PWID reported in the EMCDDA barometer. In Czechia, there was 1 study on non-PWID of high quality, which included CHC data (Chlibek 2017). To estimate the CHC prevalence in the general population (primarily non-PWID), we used a fixed-effect meta-analytic approach, with the corresponding results presented in Table 2. The prevalence of recent and ex-PWID was low in Czechia (about 0.47% and 0.81%, respectively) corresponding to 40,260 (95% CI: 38,680-41,620) recent PWID and 69,050 (95% CI: 67,040-71,320) ex-PWID in the population. However, the CHC prevalence in these groups was substantial (Table 2) being 22.02% and 10.9%, respectively. This translates to 8,864 (95% CI: 7,822-9,988) and 7,528 (95% CI: 5,644-9,561) recent and ex-PWID aged 15-79 living with CHC infection in Czechia in 2019. The CHC prevalence in the general population was 0.6% (95% CI: 0.36%-0.92%), much lower than that of the high-risk groups. Taking all pieces of information into account, the overall CHC prevalence in Czechia in 2019 was equal to 0.78% (95% CI: 0.55%-1.11%), which corresponds to 66,794 (95% CI: 46,853-94,196) individuals aged 15-79 years with CHC infection.

The results from our model including migrants from endemic countries as a separate group are presented in Table 3. However, this analysis comes with possible limitations; that is, including migrants as a separate group is valid only if migrants do not overlap with the remaining groups (recent PWID, ex-PWID, and non-PWID). Thus, if migrants do participate proportionally in the study(ies) in the general population, this analysis may result in biased overall CHC estimates (most probably in higher CHC prevalence estimates).

# TABLES AND FIGURES

*Table 1. Data (real) contributing to the estimation of CHC prevalence in Czechia in 2019.*

| Parameter | Number (95%CI) | Numerator | Denominator | Notes | Year of study |
|---|---|---|---|---|---|
| $\rho_{rec}$ | 40,260 (38,680-41,620) | | | Method based on McDonald et al. | 2019 |
| $\rho_{ex}$ | 69,050 (67,040-71,320) | | | Method based on McDonald et al. | 2019 |
| $\pi(\text{anti-HCV})_{rec}$ | | 226 | 760 | EMCDDA database | 2003 |
| $\pi(\text{anti-HCV})_{ever}$ | | 315 | 1,557 | EMCDDA database | 2019 |
| $\pi_{non}$ | | 17 | 2,953 | ECDC database (Chlibeck et al.); Risk of bias=4† | 2015 |

*Notes*: Higher risk of bias score denotes a higher-quality study (range from 0 to 6); † After excluding PWID from the study; 47 individuals reported injecting drug use, of whom, 11 were CHC positive.

*Table 2. Results from the method assuming no heterogeneity in the estimation of the CHC prevalence in the general population from different studies (i.e., similar to a fixed-effect meta-analysis).*

| Parameter | Posterior median | Posterior sd | LB (2.5%) | UB (97.5%) |
|---|---|---|---|---|
| $\rho_{rec}$ (%) | 0.47 | 0.01 | 0.45 | 0.49 |
| $\rho_{ex}$ (%) | 0.81 | 0.01 | 0.78 | 0.84 |
| $\pi_{rec}$ (%) | 22.02 | 1.33 | 19.52 | 24.72 |
| $\pi_{ex}$ (%) | 10.9 | 1.43 | 8.19 | 13.79 |
| $\pi_{non}$ (%) | 0.6 | 0.14 | 0.36 | 0.92 |
| $\pi$ (%) | 0.78 | 0.14 | 0.55 | 1.11 |
| Number with CHC | 66,794 | 12,123 | 46,853 | 94,196 |
| Pr(Ever PWID\|CHC) (%) | 24.6 | 4.62 | 17.22 | 35.1 |
| Pr(Non-PWID\|CHC) (%) | 75.4 | 4.62 | 64.9 | 82.78 |

*Notes*: $\rho_{rec}$, prevalence of recent PWID; $\rho_{ex}$, prevalence of ex-PWID; $\pi_{rec}$, CHC prevalence among recent PWID; $\pi_{ex}$, CHC prevalence among ex-PWID; $\pi_{non}$, CHC prevalence among non-PWID; $\pi$ overall CHC prevalence in Czechia; LB, Lower Boundary; UP, Upper Boundary

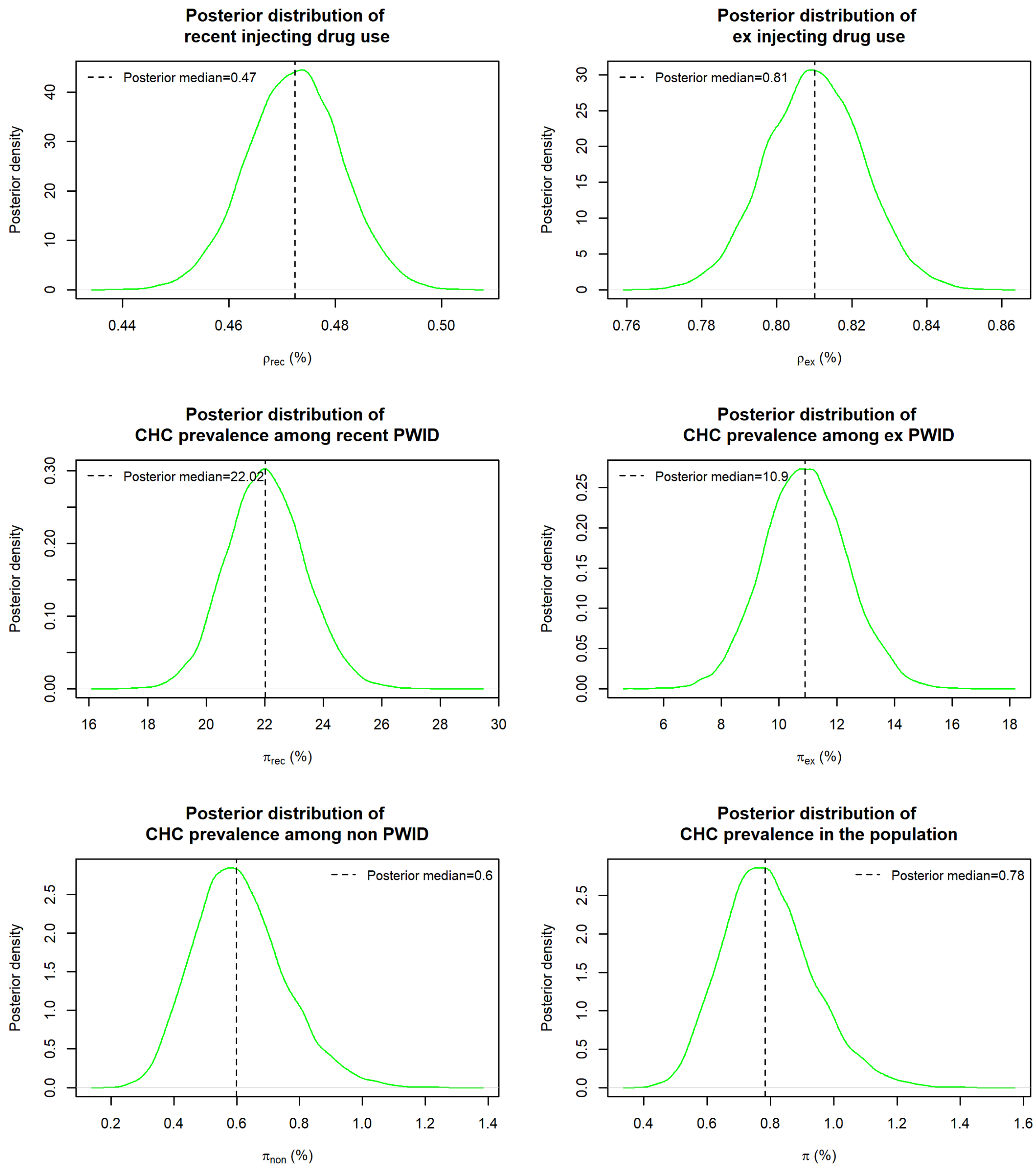


*Figure 1. Posterior distributions of the key model parameters using the approach that assumes no heterogeneity in the estimation of the CHC prevalence in the general population (primarily non-PWID) from different studies (i.e., similar to a fixed-effect meta-analysis).*

*Table 3. Results from the method assuming no heterogeneity in the estimation of the CHC prevalence in the general population (primarily non-PWID) from different studies (i.e., similar to a fixed-effect meta-analysis) including migrants from endemic countries as a separate group.*

| Parameter | Posterior median | Posterior sd | LB (2.5%) | UB (97.5%) |
|---|---|---|---|---|
| $\rho_{rec}$ (%) | 0.47 | 0.01 | 0.45 | 0.49 |
| $\rho_{ex}$ (%) | 0.81 | 0.01 | 0.78 | 0.84 |
| $\rho_{mig}$ (%) | 3.7 | 0 | 3.7 | 3.7 |
| $\pi_{rec}$ (%) | 22.05 | 1.36 | 19.48 | 24.81 |
| $\pi_{ex}$ (%) | 10.88 | 1.44 | 8.12 | 13.73 |
| $\pi_{mig}$ (%) | 1.8 | 0.43 | 0.96 | 2.64 |
| $\pi_{non}$ (%) | 0.6 | 0.14 | 0.36 | 0.92 |
| $\pi$ (%) | 0.83 | 0.14 | 0.6 | 1.13 |
| Number with CHC | 70,478 | 11,747 | 50,720 | 96,713 |
| Pr(Ever PWID\|CHC) (%) | 23.26 | 4 | 16.76 | 32.41 |
| Pr(Mig\|CHC) (%) | 7.98 | 2.23 | 4.15 | 12.85 |
| Pr(Non-PWID\|CHC) (%) | 68.69 | 5.38 | 56.68 | 77.55 |

*Notes*: $\rho_{rec}$, prevalence of recent PWID; $\rho_{ex}$, prevalence of ex-PWID; $\rho_{mig}$, prevalence of migrants from endemic countries; $\pi_{rec}$, CHC prevalence among recent PWID; $\pi_{ex}$, CHC prevalence among ex-PWID; $\pi_{mig}$, CHC prevalence among migrants from endemic countries; $\pi_{non}$, CHC prevalence among non-PWID; $\pi$ overall CHC prevalence in Czechia; LB, Lower Boundary; UP, Upper Boundary

# APPENDIX

## Fit of the multi-state Markov model

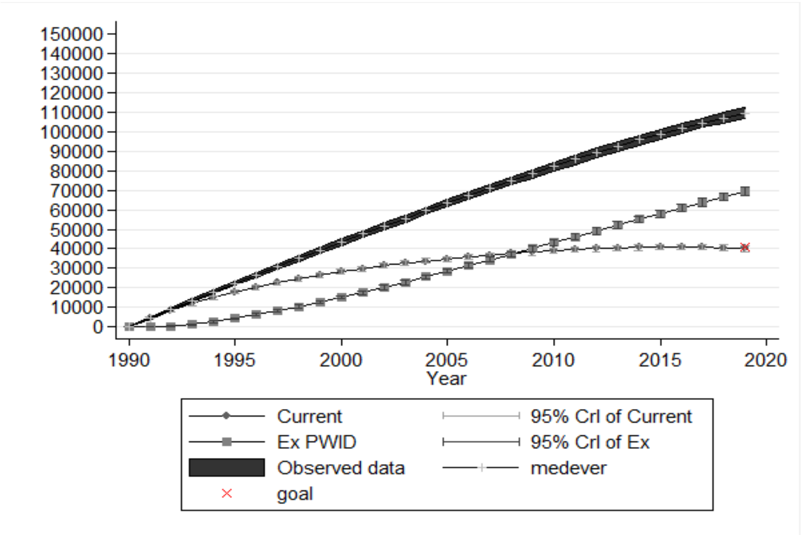


*Figure 2: Model predictions for the numbers of current and ex-PWID (people who inject drugs). The solid black line and shaded grey error bars show the median and 95% credible intervals (95% CrI) for the model projections. For comparison, asterisks indicate the observed country's data.*

## Stan code for Bayesian multiparameter evidence synthesis

```
data {
  int<lower=0> N1579; // Population of 15-79 in `Country`
  int<lower=1> Knon; // Number of studies in the ECDC data for `Country`
  int<lower=1> Kever; // Number of studies in the EMCDDA data for ever users in `Country`
  int<lower=1> Kcur; // Number of studies in the EMCDDA barometer (for HCV prevalence of PWID) in `Country`

  real p_cur_mean; // Prior mean for the prevalence of current use in `Country`
  real<lower=0> p_cur_sd; // Prior sd for the prevalence of current use in `Country`

  real p_ex_mean; // Prior mean for the prevalence of ex-use in `Country`
  real<lower=0> p_ex_sd; // Prior sd for the prevalence of ex-use in `Country`

  int<lower=0> Nst_hcv_cur[Kcur]; // Number of individuals in the study estimating anti-HCV among recent PWID
  int<lower=0> Yst_hcv_cur[Kcur]; // Number of HCV+ in the study estimating anti-HCV among recent PWID

  int<lower=0> Nst_hcv_ever[Kever]; // Number of individuals in the study estimating anti-HCV among ever IDU
  int<lower=0> Yst_hcv_ever[Kever]; // Number of HCV+ in the study estimating anti-HCV among ever PWID

  int<lower=0> Nst_CHC_non[Knon]; // Number of individuals in the study estimating CHC among non PWID
  int<lower=0> Yst_CHC_non[Knon]; // Number of HCV+ in the study estimating CHC among non PWID
```

```
  vector<lower=0>[3] alpha; // parameter of the Diriclet prior

  real HCVclear_mean; // Prior mean for the HCV clearance probability
  real<lower=0> HCVclear_sd; // Prior sd for the HCV clearance
probability
}

// Block defining the original parameters
parameters {
  // The parameters to be sampled
  simplex[3] rho; // Prevalence of the three risk groups
  real<lower=0,upper=1> CHCpi_cur; // P(CHC+|Cur)
  real<lower=0,upper=1> CHCpi_ex; // P(CHC+|Ex)
  real<lower=0,upper=1> CHCpi_non; // P(CHC+|Non)

  real<lower=0,upper=1-CHCpi_cur> HCVclear; // Probability of HCV
clearance; upper bound = 1-prevalence of chronic HCV
}


transformed parameters {
  // Change scales
  real<lower=0,upper=1> rho_ever;
  real<lower=0,upper=1> CHCpi_ever;
  real<lower=0,upper=1> pi_ever;
  real<lower=0,upper=1> pi_cur;

  rho_ever = rho[1] + rho[2];
  CHCpi_ever = CHCpi_cur*rho[1]/rho_ever + CHCpi_ex*rho[2]/rho_ever;
  pi_ever = CHCpi_ever/(1-HCVclear);
  pi_cur = CHCpi_cur/(1-HCVclear);
```

```
}


// Binomial regression model
model {
  // Priors
  rho ~ dirichlet(alpha);

  ///////////////////////////////
  // Likelihood contributions //
  ///////////////////////////////

  // Probability of HCV clearance
  HCVclear ~ normal(HCVclear_mean,HCVclear_sd);

  // Prevalence of current use
  rho[1] ~ normal(p_cur_mean,p_cur_sd);

  // Prevalence of ex-use
  rho[2] ~ normal(p_ex_mean,p_ex_sd);

  // Prevalence of chronic HCV among current users
  Yst_hcv_cur ~ binomial(Nst_hcv_cur,pi_cur);

  // Prevalence of HCV among ever users
  Yst_hcv_ever ~ binomial(Nst_hcv_ever,pi_ever);

  // HCV+ among non
  Yst_CHC_non ~ binomial(Nst_CHC_non,CHCpi_non);
}
```

```
generated quantities {
  // Functions of parameters
  real<lower=0,upper=100> overalCHC;
  real logit_CHCpi_cur;
  real logit_CHCpi_ex;
  real logit_CHCpi_non;
  real logit_rho_cur;
  real logit_rho_ex;
  real logit_rho_non;
  real<lower=0,upper=1> pEverGivenCHC;
  real<lower=0,upper=1> pNonGivenCHC;
  real logit_HCVclear;
  real<lower=0> NumberCHC;

  // Overall HCV prevalence
  overalCHC = 100*(rho[1]*CHCpi_cur + rho[2]*CHCpi_ex +
rho[3]*CHCpi_non);
  pEverGivenCHC = CHCpi_ever*rho_ever/(overalCHC/100);
  pNonGivenCHC = CHCpi_non*(1-rho_ever)/(overalCHC/100);
  NumberCHC = round(overalCHC*N1579/100);

  logit_rho_cur = logit(rho[1]);
  logit_rho_ex = logit(rho[2]);
  logit_rho_non = logit(rho[3]);
  logit_CHCpi_cur = logit(CHCpi_cur);
  logit_CHCpi_ex = logit(CHCpi_ex);
  logit_CHCpi_non = logit(CHCpi_non);
  logit_HCVclear = logit(HCVclear);
}
```

## Multi-state Markov model

```
#include <stdio.h>
#include <stdlib.h>
#include <string.h>
#include <math.h>
#include <time.h>

#define loops 100
int populationPerYearAndAge[2021][66];
double deathRatePerAge[66];
int totalPopulationPerYear[2021];

FILE *F_DeathRate=fopen("`Country`-deathRates.txt","r");
FILE *F_Population=fopen("`Country`-population.txt","r");
FILE *out;

double pa_start = 0.00012;                          // Setting Variable
double pa_step  = 0.000002;                         // Setting Variable
double pa_stop  = 0.000123;                         // Setting Variable

double pa;
double pg = 1.0/11.7;    // Assumed average active injecting career of 
11.7 years
double pk = 0.004*12.0;      // Relapse rate of 0.004/month

struct people{
    int age;
    int state;
};

char filename[100];
struct people person[50000000];
```

```
long int totalPersons=0;

void setPopulationAge()
{
    for( int j=0; j<71; j++){
        for( int i=0; i<51; i++){

fscanf(F_Population,"%d",&populationPerYearAndAge[1950+j][15+i]);
        }
    }
}

void setDeathRate()
{
    for( int i=0; i<51; i++){
        fscanf(F_DeathRate,"%lf",&deathRatePerAge[15+i*5]);
        for( int j=0; j<5; j++){
            deathRatePerAge[15+i*5+j] = deathRatePerAge[15+i*5];
        }
    }
}

void getTotalPopulationPerAge()
{
    for( int j=0; j<71; j++){
        for( int i=0; i<51; i++){
            totalPopulationPerYear[1950+j] +=
populationPerYearAndAge[1950+j][15+i];
        }
    }
}
```

```
void printTotalPopulationPerAge()
{
    printf("---- - ----------\n");
    printf("Year - Population\n");
    printf("---- - ----------\n");
    for( int i=1950; i<2021; i++){
        printf("%d - %d\n",i,totalPopulationPerYear[i]);
    }
}

void printDeathRatePerAge()
{
    printf("--- - -----\n");
    printf("Age - Rate\n");
    printf("--- - -----\n");
    for( int i=15; i<65; i++){
        printf("%d - %lf\n",i,deathRatePerAge[i]);
    }
}

void printTotalPersonPerState( int year)
{
    int count[4]= {0,0,0,0};
    for( int i=0; i<totalPersons; i++){
        count[person[i].state]++;
    }

//printf("%d\t%ld\t%ld\t%ld\t%ld\n",year,count[0],count[1],count[2],count
[3]);
    if(year==2014)

printf("%d\t%ld\t%ld\t%ld\t%ld\t%lf\n",year,count[0],count[1],count[2],co
```

```
unt[3],pa);
    if( year>2009){
        fprintf(out,
"%d\t%ld\t%ld\t%ld\t%ld\n",year,count[0],count[1],count[2],count[3]);
    }
}

void initializePopulation()
{
    int cnt=0;
    for( int j=15; j<65; j++){
        for( int i=0; i<populationPerYearAndAge[1950][j]*100; i++){
            person[cnt].age=j;
            person[cnt].state=0;
            cnt++;
        }
    }
    totalPersons = cnt;
}

bool checkRate(double rate)
{
    if (rate <0.001){
        double k = rand()/(1.00+RAND_MAX);
        if(k<0.001){
            rate = rate*1000;
        }
        else{
            return false;
        }
    }
    double k = rand()/(1.00+RAND_MAX);
```

```
    if ( k < rate )
        return true;
    else
        return false;
}

void changeStatusAndAge()
{
    for( int i=0; i<totalPersons; i++){
        // change state
        double k = rand()/(1.00+RAND_MAX);
        if( person[i].state==0){
            if ( checkRate(pa) &&  person[i].age<40){
                person[i].state=1;
            }
        }
        else if( person[i].state==1){
            if ( k < pg ){
                person[i].state=2;
            }
        }
        else if( person[i].state==2){
            if ( k < pk ){
                person[i].state=1;
            }
        }
        // After their 49th year, all active PWID are assumed to cease
injecting
        if(person[i].age>49 && person[i].state==1){
            person[i].state=2;
        }
        // After their 64th year, remove
```

```
        if(person[i].age>64){
            person[i].state=3;
        }
        // death rate depending on age and state
        k = rand()/(1.00+RAND_MAX);
        if( person[i].state==0){
            if ( k < deathRatePerAge[person[i].age] ){
                person[i].state=3;
            }
        }
        else if( person[i].state==1){
            if ( k < deathRatePerAge[person[i].age] * 13.0 ){
                person[i].state=3;
            }
        }
        else if( person[i].state==2){
            if ( k < deathRatePerAge[person[i].age] ){
                person[i].state=3;
            }
        }
        // increase age one year
        person[i].age++;
    }
}

void addNewPersons(int year)
{
    // add new person [15 years old and non PWID]
    int cnt=totalPersons;
    for( int i=0; i<populationPerYearAndAge[year+1][15]*100; i++){
// 100 instead of 1000 for the population to reduce execution time
        person[cnt].age=15;
```

```
        person[cnt].state=0;
        cnt++;
    }
    // increase the total number of population
    totalPersons = cnt;
}
int main()
{
    srand(time(NULL));

    setPopulationAge();
    setDeathRate();

    getTotalPopulationPerAge();
    pa = pa_start;
    while ( pa < pa_stop){

        snprintf(filename, 100, "result_%lf.txt",pa);
        out=fopen(filename,"w");

        for( int iter=0; iter<loops ;iter++){
            initializePopulation();
            for( int year=1950; year<2020; year++){
                printTotalPersonPerState(year);
                changeStatusAndAge();
                addNewPersons(year);
            }
        }

        fclose(out);
        pa = pa + pa_step;
    }
```

```
}
```

# REFERENCES

Chlibek, Jan AND Sosovickova, Roman AND Smetana. 2017. “Prevalence of Hepatitis c Virus in Adult Population in the Czech Republic - Time for Birth Cohort Screening.” *PLOS ONE* 12 (4): 1–9. https://doi.org/10.1371/journal.pone.0175525.

European Centre for Disease Prevention and Control. 2016. “Epidemiological Assessment of Hepatitis b and c Among Migrants in the EU/EEA.” https://www.ecdc.europa.eu/sites/default/files/media/en/publications/Publications/epidemiological-assessment-hepatitis-B-and-C-among-migrants-EU-EEA.pdf.

European Centre for Disease Prevention and Control (ECDC). 2016. “Systematic Review on Hepatitis b and c Prevalence in the EU/EEA.” https://www.ecdc.europa.eu/sites/default/files/media/en/publications/Publications/systematic-review-hepatitis-B-C-prevalence.pdf.

———. 2021. “Hepatitis c Prevalence Database.” https://www.ecdc.europa.eu/en/all-topics-z/hepatitis-c/tools/hepatitis-c-prevalence-database.

Grebely, Jason, Sarah Larney, Amy Peacock, Samantha Colledge, Janni Leung, Matthew Hickman, Peter Vickerman, et al. 2019. “Global, Regional, and Country-Level Estimates of Hepatitis c Infection Among People Who Have Recently Injected Drugs.” *Addiction* 114 (1): 150–66. https://doi.org/https://doi.org/10.1111/add.14393.

Hines, Lindsey A., Adam Trickey, Janni Leung, Sarah Larney, Amy Peacock, Louisa Degenhardt, Samantha Colledge, et al. 2020. “Associations Between National Development Indicators and the Age Profile of People Who Inject Drugs: Results from a Global Systematic Review and Meta-Analysis.” *The Lancet Global Health* 8 (1): e76–91. https://doi.org/10.1016/S2214-109X(19)30462-0.

Lin, Lifeng, and Haitao Chu. 2020. “Meta-Analysis of Proportions Using Generalized Linear Mixed Models.” *Epidemiology* 31 (5). https://journals.lww.com/epidem/Fulltext/2020/09000/Meta_analysis_of_Proportions_Using_Generalized.16.aspx.

McDonald, Scott A., Rosmawati Mohamed, Maznah Dahlui, Herlianna Naning, and Adeeba Kamarulzaman. 2014. "Bridging the Data Gaps in the Epidemiology of Hepatitis c Virus Infection in Malaysia Using Multi-Parameter Evidence Synthesis." *BMC Infectious Diseases* 14 (1): 564. https://doi.org/10.1186/s12879-014-0564-6.

Micallef, J. M., J. M. Kaldor, and G. J. Dore. 2006. "Spontaneous Viral Clearance Following Acute Hepatitis c Infection: A Systematic Review of Longitudinal Studies." *Journal of Viral Hepatitis* 13 (1): 34–41. https://doi.org/https://doi.org/10.1111/j.1365-2893.2005.00651.x.

Presanis, Anne M., O. Noel Gill, Timothy R. Chadborn, Caterina Hill, Vivian Hope, Louise Logan, Brian D. Rice, Valerie C. Delpech, A. E. Ades, and Daniela De Angelis. 2010. "Insights into the Rise in HIV Infections, 2001 to 2008: A Bayesian Synthesis of Prevalence Evidence." *AIDS* 24 (18). https://journals.lww.com/aidsonline/Fulltext/2010/11270/Insights_into_the_rise_in_HIV_infections,_2001_to.12.aspx.

Sweeting, M. J., D. De Angelis, M. Hickman, and A. E. Ades. 2008. "Estimating hepatitis C prevalence in England and Wales by synthesizing evidence from multiple data sources. Assessing data conflict and model fit." *Biostatistics* 9 (4): 715–34. https://doi.org/10.1093/biostatistics/kxn004.

Tan, Sarah, Susanna Makela, Daliah Heller, Kevin Konty, Sharon Balter, Tian Zheng, and James H. Stark. 2018. "A Bayesian Evidence Synthesis Approach to Estimate Disease Prevalence in Hard-to-Reach Populations: Hepatitis c in New York City." *Epidemics* 23: 96–109. https://doi.org/https://doi.org/10.1016/j.epidem.2018.01.002.

Thein, Hla-Hla, Qilong Yi, Gregory J. Dore, and Murray D. Krahn. 2008. "Estimation of Stage-Specific Fibrosis Progression Rates in Chronic Hepatitis c Virus Infection: A Meta-Analysis and Meta-Regression." *Hepatology* 48 (2): 418–31. https://doi.org/https://doi.org/10.1002/hep.22375.

Veen, Maaike G. van, Anne M. Presanis, Stefano Conti, Maria Xiridou, Annemarie R. Stengaard, Martin C. Donoghoe, Ard I. van Sighem, Marianne A. van der Sande, and Daniela De Angelis. 2011. "National Estimate of HIV Prevalence in the Netherlands: Comparison and Applicability of Different Estimation Tools." *AIDS* 25 (2).

https://journals.lww.com/aidsonline/Fulltext/2011/01140/National_estimate_of_HIV_prevalence_in_the.14.aspx.

# Estimating the prevalence of chronic hepatitis C infection (CHC) in Denmark using Bayesian multiparameter evidence synthesis

08/07/2022

## Table of Contents


Introduction .....2

Methods .....2

Prevalence of ex-PWID .....3

Prevalence of recent PWID .....3

Prevalence of CHC among recent PWID .....4

Prevalence of CHC among ex-PWID .....4

Prevalence of CHC among non-PWID .....5

Sensitivity analysis including migrants from endemic countries .....6

Results .....6

Comparison with other studies .....8

Tables and Figures .....9

Appendix .....16

Fit of the multi-state Markov model .....16

Stan code for Bayesian multiparameter evidence synthesis .....17

Multi-state Markov model .....23

References .....31

# INTRODUCTION

Patients with chronic hepatitis C (HCV) infection often remain asymptomatic for decades and even for life, but a proportion of them develop active chronic hepatitis, which is a progressive disease (Thein et al. 2008). Because of the largely asymptomatic nature of HCV infection, notification data reflect national screening and testing practices and do not give accurate insights into the prevalence of HCV infection. Although there are studies that have estimated the prevalence of HCV antibodies (anti-HCV) in specific groups such as the general population, first-time blood donors, or people who inject drugs (PWID) at drug treatment centers, those alone cannot be combined to produce national estimates unless some additional information regarding the composition of each HCV risk group and its prevalence in the population are known (Sweeting et al. 2008; McDonald et al. 2014; Tan et al. 2018).

# METHODS

Bayesian multiparameter evidence synthesis (BMES) has been a popular approach to formally estimate anti-HCV or HIV prevalence (Sweeting et al. 2008; McDonald et al. 2014; Tan et al. 2018; Presanis et al. 2010; Veen et al. 2011). In this project, we extend this method to estimating the CHC prevalence, with the population (15-79 years) split into three main non-overlapping risk groups, i.e., recent PWID (those who have injected in the last year), ex-PWID, and non-PWID. A unified model is assumed including parameters associated with the CHC prevalence of recent, ex-PWID, and non-PWID (denoted by $\pi_{rec}$, $\pi_{ex}$, and $\pi_{non}$, respectively) and the prevalence of these risk groups in the population (denoted by $\rho_{rec}$, $\rho_{ex}$, and $\rho_{non}$, respectively; $\rho_{rec} + \rho_{ex} + \rho_{non} = 1$). To estimate the CHC prevalence in the whole population, $\pi$, we used the formula:

$$\pi = \pi_{rec}\rho_{rec} + \pi_{ex}\rho_{ex} + \pi_{non}\rho_{non} \quad (1)$$

Bayesian synthesis considers all available data, allowing for an evidence-based estimation of CHC prevalence in the population, with inherent uncertainty properly accounted for. Our aim is to estimate the CHC prevalence for the total (15-79) population of Denmark in 2019 using sources of information over the last decade (2010-2019).

## Prevalence of ex-PWID

Data on the prevalence of ex-PWID are sparse and generally often unreliable. To overcome this limitation, we apply the method proposed by McDonald et al. (2014), which requires simulating a multi-state Markov model representing the non-PWID, recent PWID, and ex-PWID. In the multi-state Markov model, the simulation starts in 1950 and examines the population aged 15-79 years old. A certain number of 15-year-old individuals enter the simulation process each year. These numbers of 15-year-old individuals are obtained from the United Nations Statistic Division UNSD, being different for each country. The ageing of the population is also taken into account. Country-specific overall mortality data are retrieved from the Life Tables of the World Health Organization WHO, assuming excess mortality in recent PWID. The duration of injecting career is also country-specific and obtained from the paper of Hines et al. (2020).

To estimate the number of ex-PWID, the model can be calibrated on the number of recent PWID provided in the EMCDDA barometer or the EMCDDA statistical bulletin (the code of the Markov model can be found in the Appendix). If there are no available data in the barometer, estimates from the systematic review of Grebely et al. (2019) could be used. Currently, the multi-state Markov model was calibrated on the estimated prevalence of recent PWID reported in the systematic review of Grebely et al. (2019).

After applying the model for Denmark, we compute the number (and the corresponding Confidence Interval - CI) of ex-PWID in 2019. The number of ex-PWID is then divided by the population size (15-79 years) in the same year, and a Normal distribution, constrained such that $\rho_{ex} \in (0,1)$, is assumed for $\rho_{ex}$. The standard deviation of this distribution is specified to approximately correspond to the respective CI, i.e. $\left(UB_{\rho_{ex}} - LB_{\rho_{ex}}\right)/(2 \times 1.96)$, where $UB_{\rho_{ex}}$ and $LB_{\rho_{ex}}$ denote the upper and lower limit of the CI.

## Prevalence of recent PWID

The number of recent PWID in Denmark in 2019 is also projected by the multi-state Markov model described in the previous subsection. To estimate $\rho_{rec}$, the number of recent PWID is divided by the population size (15-79 years). Similar to the previous subsection, a Normal distribution, constrained such that $\rho_{rec} \in (0,1)$, is assumed for $\rho_{rec}$, with the standard deviation specified to correspond to the CI obtained from the multi-state Markov model.

## Prevalence of CHC among recent PWID

The prevalence of CHC among recent PWID was informed by CHC prevalence data from the paper of Grebely et al. (2019) in 2011. However, some people may have been treated with direct-acting antivirals (DAAs), with the sustained virologic response ($SVR_{PWID}$) among PWID estimated to be 88% (95% CI: 80% to 93%) (Graf et al. 2019). Based on the paper of Christensen, Søholm, and Øvrehus (2021), which was provided by the national focal points, the number of individuals treated with DAAs from 2015 to 2019 is equal to 4,276, with approximately 85% of them having been infected by injecting drug use. Thus, $N_{PWID|DAA} \approx$ 3,635 PWID (recent or ex) are assumed to have been treated with DAAs. However, the proportions of recent and ex-PWID among treated ever PWID individuals are unknown. In this report, we make the assumption that these are proportional to the proportions of recent and ex-PWID among CHC-positive individuals, i.e. Pr(Recent PWID|CHC) and Pr(Ex-PWID|CHC), as estimated by our model when the DAA uptake is ignored (Table 2). Thus, the CHC prevalence among recent PWID, adjusted for DAAs, can be estimated by

$$\pi_{rec} = \frac{N_{15,79}\rho_{rec}\tilde{\pi}_{rec} - N_{PWID|DAA}\dfrac{\text{Pr(Recent PWID|CHC)}}{\text{Pr(Recent PWID|CHC)} + \text{Pr(Ex-PWID|CHC)}}SVR_{PWID}}{N_{15,79}\rho_{rec}}, \quad (2)$$

where $\tilde{\pi}_{rec}$ denotes the CHC estimate derived solely from the paper of Grebely et al. (2019) in 2011.

## Prevalence of CHC among ex-PWID

Information on CHC prevalence among ex-PWID (i.e., $\pi_{ex}$) is difficult to obtain directly. To overcome this, CHC prevalence data on ever PWID from a registry-based cohort study (Ovrehus et al. 2019) in 2014 were used. However, some ever PWID may have been treated with DAAs after 2,014. Similarly to the procedure described in the previous subsection, the CHC prevalence among ever PWID, adjusted for DAAs, can be estimated by

$$\pi_{ever} = \frac{N_{15,79}\rho_{ever}\tilde{\pi}_{ever} - N_{PWID|DAA}SVR_{PWID}}{N_{15,79}\rho_{ever}}. \quad (3)$$

where $\tilde{\pi}_{ever}$ denotes the CHC prevalence among ever PWID based on Ovrehus et al. (2019) and $\rho_{ever} = \rho_{rec} + \rho_{ex}$.

Then, since an estimate of $\pi_{ever}$ is available, $\pi_{ex}$ can be indirectly estimated since $\pi_{ever}$ is equal to a weighted average of $\pi_{rec}$ and $\pi_{ex}$, as shown by the following formula

$$\pi_{ever} = \frac{\rho_{rec}}{\rho_{ever}}\pi_{rec} + \frac{\rho_{ex}}{\rho_{ever}}\pi_{ex}, \quad (4)$$

or, equivalently,

$$\pi_{ex} = \left(\pi_{ever} - \frac{\rho_{rec}}{\rho_{ever}}\pi_{rec}\right) \times \frac{\rho_{ever}}{\rho_{ex}}. \quad (5)$$

## Prevalence of CHC among non-PWID

To estimate the CHC prevalence among non-PWID in a country (after 2010), the ECDC database (European Centre for Disease Prevention and Control (ECDC) 2021) was used. The ECDC group has carefully and critically collected information from relevant studies across the EU/EEA region in cooperation with the focal points of each country. On the basis of the representativeness of each study and other factors (European Centre for Disease Prevention and Control (ECDC) 2016), a risk of bias score was assigned to each study, with higher values indicating higher-quality studies. When higher-quality studies are available in a country (≥ 4), they are used to estimate the overall CHC prevalence in that country among non-PWID. If there are no higher-quality studies, general population estimates with a lower quality (<4) are pooled. If no general population prevalence estimates are available, data on the prevalence of HCV in pregnant women could be used as a prevalence measure. Finally, if data on pregnant women are lacking too, first-time blood donor studies could be an option. However, if the national focal point agrees or recommends, estimates from a neighboring country could be used instead, or we could use any other relevant information provided/suggested by the national focal point.

We directly use CHC prevalence data from studies in the general population, if available. However, anti-HCV data in the general population in Denmark were based on first-time blood donors, with a recent seroprevalence study in the general population lacking. Since the CHC prevalence based on first-time blood donor studies may be underestimated, we did not perform a treatment adjustment in the general population as this would lead to CHC prevalence close to zero, which would be unreliable. In principle, we intend to remove individuals cured with DAAs from the general population only if a recent seroprevalence study is available.

As $\rho_{rec}$ and $\rho_{ex}$ are likely to be low, $\pi_{non}$ will be the most critical factor for the estimation of the overall CHC prevalence in the population. To adjust for potential heterogeneity between the CHC prevalence estimates from different studies in the non-PWID population in a country, as a sensitivity analysis, we also apply a random-effect meta-analytic approach to pool the CHC prevalence across studies on non-PWID (Lin and Chu 2020). The random-effect approach is carried out only when at least 3 studies are available in the general population of a certain country, and the estimates are compared with the corresponding estimates from the approach assuming no heterogeneity (similar to a fixed-effect meta-analysis). If the estimates are inconsistent, possible explanations are investigated and discussed. If there is substantial heterogeneity between studies, it would help more to explore the causes; for example, following national focal point suggestions, some studies could be excluded to reduce heterogeneity.

### Sensitivity analysis including migrants from endemic countries

A sensitivity analysis including data for the viremic population among migrants based on the report of the ECDC group (European Centre for Disease Prevention and Control 2016) was carried out. Migrants from endemic countries represent 6% of the adult population in Denmark (Table 8 in European Centre for Disease Prevention and Control (2016)), with the respective CHC prevalence being equal to $\pi_{mig} = 1.4\%$ (Table 9 in European Centre for Disease Prevention and Control (2016)). The overall CHC prevalence is now estimated by

$$\pi = \pi_{rec}\rho_{rec} + \pi_{ex}\rho_{ex} + \pi_{mig}\rho_{mig} + \pi_{non}\rho_{non},$$

where $\rho_{rec} + \rho_{ex} + \rho_{mig} + \rho_{non} = 1$. Due to lack of data on the proportion of migrants among individuals treated with DAAs, we did not perform a treatment adjustment in this sensitivity analysis.

## RESULTS

The above-mentioned approach was used to estimate the prevalence of CHC in 2019 (considering ages between 15-79 years: 4,584,865).

The aggregated data used by our approach are briefly presented in Table 1. The multi-state Markov model was calibrated on the estimated prevalence of recent PWID reported in the systematic review of Grebely et al. (2019). In Denmark, there were 7 studies on first-time blood

donors, which included only anti-HCV data. To estimate the CHC prevalence in the general population (primarily non-PWID), we used a fixed-effect meta-analytic approach, with the corresponding results, when information on DAAs is ignored, presented in Table 2. However, approximately 4,276 individuals were treated with DAAs from 2015 to 2019 in Denmark, of whom, 3,635 were recent or ex-PWID. In this report, among the 3,635 ever PWID treated individuals, it is assumed that the proportions of recent PWID and ex-PWID are equal to $\frac{\Pr(\text{Recent PWID}|\text{CHC})}{\Pr(\text{Recent PWID}|\text{CHC})+\Pr(\text{Ex-PWID}|\text{CHC})} \approx 33.36\%$ (95% CI:27.53%-39.69%) and $\frac{\Pr(\text{Ex-PWID}|\text{CHC})}{\Pr(\text{Recent PWID}|\text{CHC})+\Pr(\text{Ex-PWID}|\text{CHC})} \approx 66.64\%$ (95% CI:60.31%-72.47%), respectively, as estimated by our model when information on DAAs is ignored (Table 2).

The corresponding results accounting for the DAA uptake are presented in Table 3. The prevalence of recent and ex-PWID was low in Denmark (about 0.35% and 0.75%, respectively) corresponding to 16,000 (95% CI: 15,250-16,800) recent PWID and 34,330 (95% CI: 33,320-35,500) ex-PWID in the population. However, the CHC prevalence in these groups was substantial (Table 3) being 25.23% and 23.5%, respectively. This translates to 4,031 (95% CI: 3,351-4,740) and 8,066 (95% CI: 6,832-9,327) recent and ex-PWID aged 15-79 living with CHC infection in Denmark in 2019. The CHC prevalence in the general population was 0.01% (95% CI: 0%-0.01%), much lower than that of the high-risk groups. Taking all pieces of information into account, the overall CHC prevalence in Denmark in 2019 was equal to 0.27% (95% CI: 0.25%-0.3%), which corresponds to 12,423 (95% CI: 11,262-13,621) individuals aged 15-79 years with CHC infection. The corresponding results under a random-effect meta-analysis for the studies in the general population were very similar and are provided in Table 4. Moreover, the heterogeneity between the estimates of the studies in the general population is estimated to be low (Table 4).

The results from our model including migrants from endemic countries as a separate group are presented in Table 5. However, this analysis comes with possible limitations; that is, including migrants as a separate group is valid only if migrants do not overlap with the remaining groups (recent PWID, ex-PWID, and non-PWID). Thus, if migrants do participate proportionally in the study(ies) in the general population, this analysis may result in biased overall CHC estimates (most probably in higher CHC prevalence estimates).

## Comparison with other studies/Comments

In Denmark, there are prior studies aiming to estimate the prevalence of CHC. Using capture-recapture methods with data from all the different registers of CHC in Denmark and covering cases up to 2007, Christensen et al. (2012) estimated that the total population with CHC in Denmark was 16,888 (95% CI: 16,474-18,287), corresponding to 0.38% (95% CI: 0.37-0.42) of the population over 15 years of age. It is interesting that these results are very similar to our estimate when DAA information is ignored, i.e. the total CHC prevalence of 0.34% (95% CI: 0.32%-0.37%), which corresponds to 15,617 (95% CI: 14,468-16,806) individuals aged 15-79 years (Table 2).

Using the same methodology and databases as Christensen et al. (2012), Nielsen et al. (2020) estimated that the total CHC-infected population was 9,975 corresponding to 0.21% of the adult population (95%CI: 9,758–16,659; 0.21%-0.36%), after taking data up to 2016 into account. This is quite similar to our estimate when information on treatment is considered (Table 3), i.e. 12,423 (95% CI: 11,262-13,621) individuals aged 15-79 years living with CHC infection in 2019.

Finally, according to local experts, the estimate of current drug injectors is probably higher than the actual one as drug injection has been decreasing over the last decade.

# TABLES AND FIGURES

*Table 1. Data (real) contributing to the estimation of CHC prevalence in Denmark in 2019.*

| Parameter | Estimate (95%CI) | Number (95%CI) | Numerator | Denominator | Notes | Year of study |
|---|---|---|---|---|---|---|
| $\rho_{rec}$ | | 16,000 (15,250-16,800) | | | Method based on McDonald et al. | 2019 |
| $\rho_{ex}$ | | 34,330 (33,320-35,500) | | | Method based on McDonald et al. | 2019 |
| $\pi_{rec}$ | 31.9% (26.8%-37.2%) | | | | Grebely et al. | 2011 |
| $\pi_{ever}$ | | | 521 | 1,715 | (Ovrehus et al. 2019) | 2014 |
| $\pi(\text{anti-HCV})_{non}$ | | | 1 | 27,282 | ECDC database (NA et al.); Risk of bias=NA | 2010 |
| $\pi(\text{anti-HCV})_{non}$ | | | 4 | 25,647 | ECDC database (NA et al.); Risk of bias=NA | 2011 |

| Parameter | Estimate (95%CI) | Number (95%CI) | Numerator | Denominator | Notes | Year of study |
|---|---|---|---|---|---|---|
| $\pi(\text{anti-HCV})_{non}$ | | | 4 | 35,522 | ECDC database (NA et al.); Risk of bias=NA | 2012 |
| $\pi(\text{anti-HCV})_{non}$ | | | 2 | 21,576 | ECDC database (NA et al.); Risk of bias=NA | 2013 |
| $\pi(\text{anti-HCV})_{non}$ | | | 2 | 21,794 | ECDC database (NA et al.); Risk of bias=NA | 2014 |
| $\pi(\text{anti-HCV})_{non}$ | | | 3 | 25,745 | ECDC database (NA et al.); Risk of bias=NA | 2015 |
| $\pi(\text{anti-HCV})_{non}$ | | | 1 | 28,294 | ECDC database (NA et al.); Risk of bias=NA | 2016 |

*Notes*: Although it looks counter-intuitive, **a higher risk of bias score denotes a higher-quality study** (range from 0 to 6);

Table 2. Results from the method assuming no heterogeneity in the estimation of the CHC prevalence in the general population from different studies (i.e., similar to a fixed-effect meta-analysis).

| Parameter | Posterior median | Posterior sd | LB (2.5%) | UB (97.5%) |
|---|---|---|---|---|
| $\rho_{rec}$ (%) | 0.35 | 0.01 | 0.33 | 0.37 |
| $\rho_{ex}$ (%) | 0.75 | 0.01 | 0.72 | 0.77 |
| $\pi_{rec}$ (%) | 31.93 | 2.65 | 26.82 | 37.11 |
| $\pi_{ex}$ (%) | 29.69 | 2.03 | 25.72 | 33.67 |
| $\pi_{non}$ (%) | 0.01 | 0 | 0 | 0.01 |
| $\pi$ (%) | 0.34 | 0.01 | 0.32 | 0.37 |
| Number with CHC | 15,617 | 596 | 14,468 | 16,806 |
| Pr(Recent PWID\|CHC) (%) | 32.66 | 3.03 | 26.96 | 38.82 |
| Pr(Ex-PWID\|CHC) (%) | 65.26 | 3.08 | 58.96 | 71.04 |
| Pr(Non-PWID\|CHC) (%) | 2.04 | 0.5 | 1.23 | 3.15 |

*Notes*: Information on DAAs is not taken into account.

*Abbreviations*: $\rho_{rec}$, prevalence of recent PWID (proportion of the population that is recent PWID); $\rho_{ex}$, prevalence of ex-PWID (proportion of the population that is ex-PWID); $\pi_{rec}$, CHC prevalence among recent PWID; $\pi_{ex}$, CHC prevalence among ex-PWID; $\pi_{non}$, CHC prevalence among non-PWID; $\pi$ overall CHC prevalence in Denmark; LB, Lower Boundary; UP, Upper Boundary

*Table 3. Results from the method assuming no heterogeneity in the estimation of the CHC prevalence in the general population from different studies (i.e., similar to a fixed-effect meta-analysis).*

| Parameter | Posterior median | Posterior sd | LB (2.5%) | UB (97.5%) |
|---|---|---|---|---|
| $\rho_{rec}$ (%) | 0.35 | 0.01 | 0.33 | 0.37 |
| $\rho_{ex}$ (%) | 0.75 | 0.01 | 0.72 | 0.77 |
| $\pi_{rec}$ (%) | 25.23 | 2.12 | 21.1 | 29.41 |
| $\pi_{ex}$ (%) | 23.5 | 1.79 | 20.01 | 27.01 |
| $\pi_{non}$ (%) | 0.01 | 0 | 0 | 0.01 |
| $\pi$ (%) | 0.27 | 0.01 | 0.25 | 0.3 |
| Number with CHC | 12,423 | 600 | 11,262 | 13,621 |
| Pr(Recent PWID\|CHC) (%) | 32.49 | 3.02 | 26.79 | 38.66 |
| Pr(Ex-PWID\|CHC) (%) | 64.91 | 3.07 | 58.62 | 70.71 |
| Pr(Non-PWID\|CHC) (%) | 2.57 | 0.62 | 1.55 | 3.95 |

*Notes*: The number of individuals treated with DAAs is taken into account.

*Abbreviations*: $\rho_{rec}$, prevalence of recent PWID (proportion of the population that is recent PWID); $\rho_{ex}$, prevalence of ex-PWID (proportion of the population that is ex-PWID); $\pi_{rec}$, CHC prevalence among recent PWID; $\pi_{ex}$, CHC prevalence among ex-PWID; $\pi_{non}$, CHC prevalence among non-PWID; $\pi$ overall CHC prevalence in Denmark; LB, Lower Boundary; UP, Upper Boundary

*Table 4. Results from the method assuming heterogeneity in the estimation of the CHC prevalence in the general population from different studies (i.e., similar to a random-effect meta-analysis).*

| Parameter | Posterior median | Posterior sd | LB (2.5%) | UB (97.5%) |
|---|---|---|---|---|
| $\rho_{rec}$ (%) | 0.35 | 0.01 | 0.33 | 0.37 |
| $\rho_{ex}$ (%) | 0.75 | 0.01 | 0.72 | 0.77 |
| $\pi_{rec}$ (%) | 25.17 | 2.1 | 21.17 | 29.32 |
| $\pi_{ex}$ (%) | 23.51 | 1.8 | 19.99 | 27.08 |
| $\pi_{non}$ (%) | 0.01 | 0 | 0 | 0.01 |
| $\pi$ (%) | 0.27 | 0.01 | 0.24 | 0.3 |
| Number with CHC | 12,374 | 610 | 11,212 | 13,595 |
| Pr(Recent PWID\|CHC) (%) | 32.52 | 3.02 | 26.9 | 38.68 |
| Pr(Ex-PWID\|CHC) (%) | 65.17 | 3.1 | 58.85 | 70.93 |
| Pr(Non-PWID\|CHC) (%) | 2.21 | 0.95 | 0.92 | 4.41 |
| Between-study variance | 0.34 | 0.78 | 0.02 | 2.78 |

*Notes*: The number of individuals treated with DAAs is taken into account.

*Abbreviations*: $\rho_{rec}$, prevalence of recent PWID (proportion of the population that is recent PWID); $\rho_{ex}$, prevalence of ex-PWID (proportion of the population that is ex-PWID); $\pi_{rec}$, CHC prevalence among recent PWID; $\pi_{ex}$, CHC prevalence among ex-PWID; $\pi_{non}$, CHC prevalence among non-PWID; $\pi$ overall CHC prevalence in Denmark; LB, Lower Boundary; UP, Upper Boundary

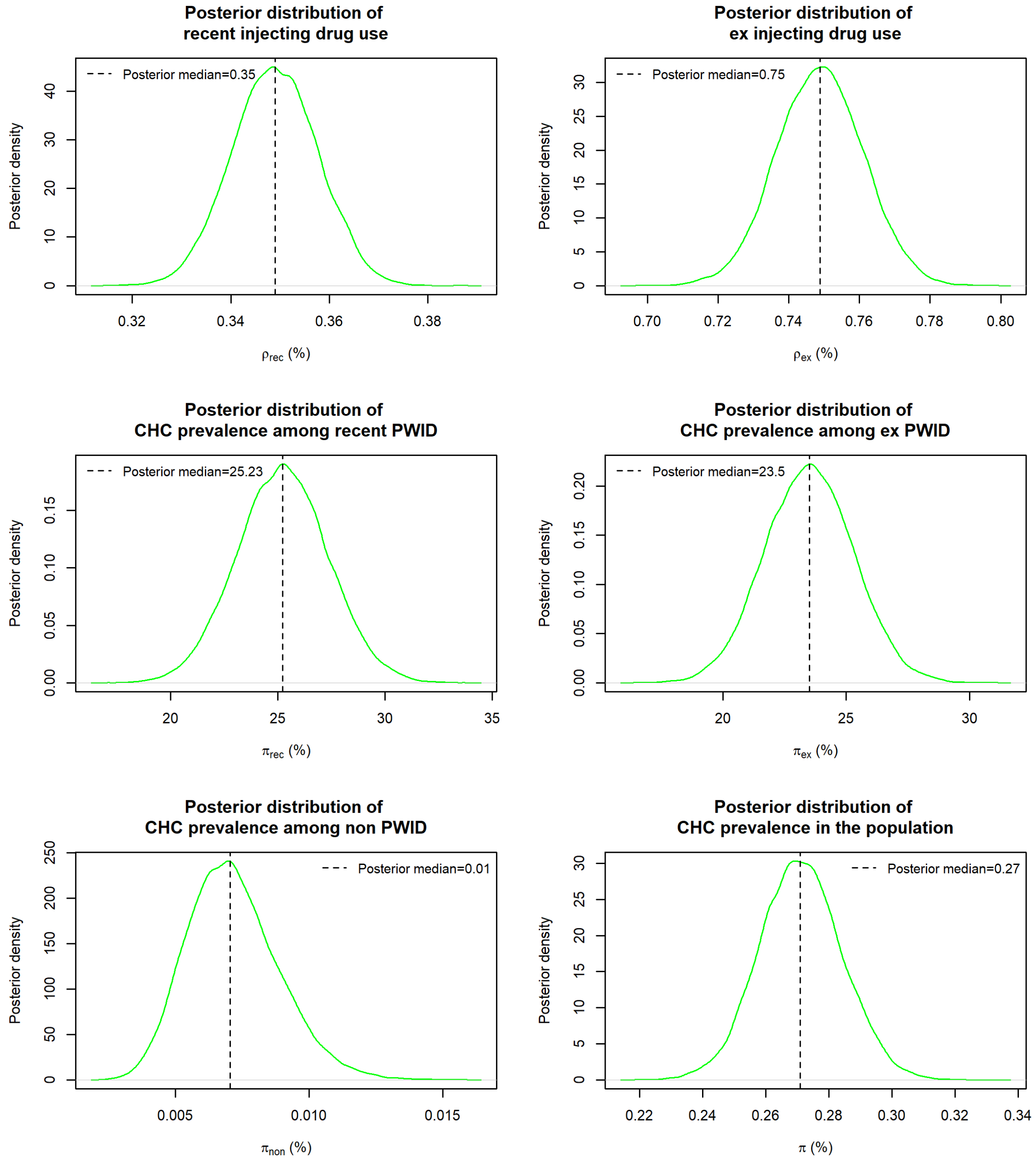


*Figure 1. Posterior distributions of the key model parameters using the approach that assumes no heterogeneity in the estimation of the CHC prevalence in the general population (primarily non-PWID) from different studies (i.e., similar to a fixed-effect meta-analysis), with the number of individuals treated with DAA taken into account.*

*Table 5. Results from the method assuming no heterogeneity in the estimation of the CHC prevalence in the general population (primarily non-PWID) from different studies (i.e., similar to a fixed-effect meta-analysis) including migrants from endemic countries as a separate group.*

| Parameter | Posterior median | Posterior sd | LB (2.5%) | UB (97.5%) |
|---|---|---|---|---|
| $\rho_{rec}$ (%) | 0.35 | 0.01 | 0.33 | 0.37 |
| $\rho_{ex}$ (%) | 0.75 | 0.01 | 0.72 | 0.77 |
| $\rho_{mig}$ (%) | 6 | 0 | 6 | 6 |
| $\pi_{rec}$ (%) | 31.92 | 2.67 | 26.58 | 37.16 |
| $\pi_{ex}$ (%) | 29.7 | 2.06 | 25.68 | 33.76 |
| $\pi_{mig}$ (%) | 1.4 | 0.27 | 0.88 | 1.93 |
| $\pi_{non}$ (%) | 0.01 | 0 | 0 | 0.01 |
| $\pi$ (%) | 0.42 | 0.02 | 0.38 | 0.47 |
| Number with CHC | 19,447 | 957 | 17,578 | 21,337 |
| Pr(Recent PWID\|CHC) (%) | 26.24 | 2.61 | 21.4 | 31.59 |
| Pr(Ex-PWID\|CHC) (%) | 52.38 | 3.31 | 46.08 | 59.09 |
| Pr(Mig\|CHC) (%) | 19.78 | 3.11 | 13.39 | 25.49 |
| Pr(Non-PWID\|CHC) (%) | 1.54 | 0.38 | 0.93 | 2.39 |

*Notes*: The number of individuals treated with DAAs is not taken into account.

*Abbreviations*: $\rho_{rec}$, prevalence of recent PWID (proportion of the population that is recent PWID); $\rho_{ex}$, prevalence of ex-PWID (proportion of the population that is ex-PWID); $\rho_{mig}$, prevalence of migrants from endemic countries (proportion of the population that belongs to this group); $\pi_{rec}$, CHC prevalence among recent PWID; $\pi_{ex}$, CHC prevalence among ex-PWID; $\pi_{mig}$, CHC prevalence among migrants from endemic countries; $\pi_{non}$, CHC prevalence among non-PWID; $\pi$ overall CHC prevalence in Denmark; LB, Lower Boundary; UP, Upper Boundary

# APPENDIX

## Fit of the multi-state Markov model

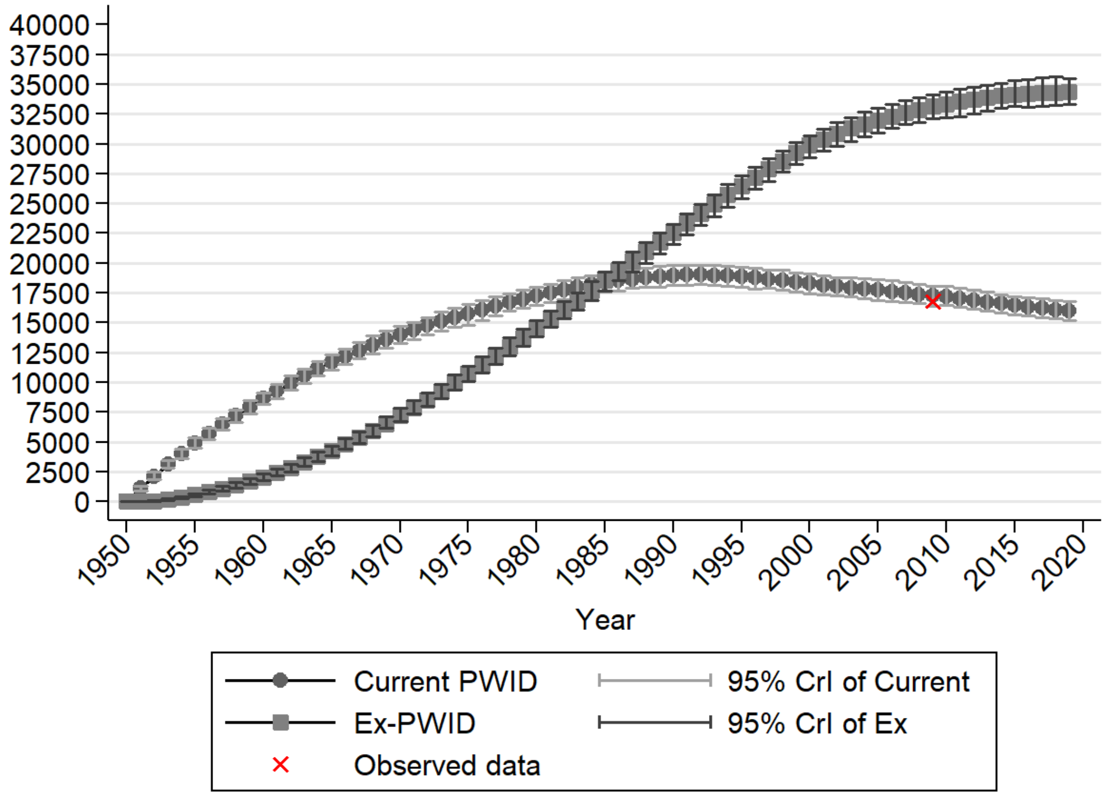


*Figure 2: Model predictions for the numbers of current and ex-PWID (people who inject drugs). The solid black line and shaded grey error bars show the median and 95% credible intervals (95% CrI) for the model projections. For comparison, asterisks indicate the observed country's data.*

## Stan code for Bayesian multiparameter evidence synthesis

```
data {
  int<lower=0> N1579; // Population of 15-64 in `Country`
  int<lower=1> Knon; // Number of studies in the ECDC data for `Country`
  int<lower=1> Kever; // Number of studies in the EMCDDA data for ever users in `Country`
  int<lower=1> NDAA; // Total number of DAAs from 2015 to 2019
  int<lower=1> NDAA_PWID; // Total number of DAAs among PWIDs from 2015 to 2019

  real p_cur_mean; // Prior mean for the prevalence of current use in `Country`
  real<lower=0> p_cur_sd; // Prior sd for the prevalence of current use in `Country`

  real p_ex_mean; // Prior mean for the prevalence of ex-use in `Country`
  real<lower=0> p_ex_sd; // Prior sd for the prevalence of ex-use in `Country`

  real p_CHC_cur_mean; // Prior mean for the CHC prevalence among recent PWID in `Country`
  real<lower=0> p_CHC_cur_sd; // Prior sd for the CHC prevalence among recent PWID in `Country`

  int<lower=0> Nst_CHC_ever[Kever]; // Number of individuals in the study estimating CHC among ever IDU
  int<lower=0> Yst_CHC_ever[Kever]; // Number of HCV+ in the study estimating CHC among ever PWID

  int<lower=0> Nst_hcv_non[Knon]; // Number of individuals in the study estimating anti-HCV among non PWID
  int<lower=0> Yst_hcv_non[Knon]; // Number of HCV+ in the study estimati
```

```
ng anti-HCV among non PWID

  vector<lower=0>[3] alpha; // parameter of the Diriclet prior

  real HCVclear_mean; // Prior mean for the HCV clearance probability
  real<lower=0> HCVclear_sd; // Prior sd for the HCV clearance probabilit
y

  real SVR_mean; // Prior mean for the SVR among non-PWID
  real<lower=0> SVR_sd; // Prior sd for the SVR among non-PWID

  real SVR_PWID_mean; // Prior mean for the SVR among PWID
  real<lower=0> SVR_PWID_sd; // Prior sd for the SVR among PWID
}

// Block defining the original parameters
parameters {
  // The parameters to be sampled
  simplex[3] rho; // Prevalence of the three risk groups
  real<lower=0,upper=1> CHCpi_cur; // P(CHC+|Cur)
  real<lower=0,upper=1> CHCpi_ex; // P(CHC+|Ex)
  real<lower=0,upper=1> CHCpi_non; // P(CHC+|Non)
  real<lower=0,upper=1> SVR; // SVR of DAAs among non-PWID
  real<lower=0,upper=1> SVR_PWID; // SVR of DAAs among PWID

  real<lower=0,upper=1-CHCpi_cur> HCVclear; // Probability of HCV clearan
ce; upper bound = 1-prevalence of chronic HCV
}

transformed parameters {
  // Change scales
  real<lower=0,upper=1> rho_ever;
```

```
  real<lower=0,upper=1> CHCpi_ever;
  real<lower=0,upper=1> pi_non;

  rho_ever = rho[1] + rho[2];
  CHCpi_ever = CHCpi_cur*rho[1]/rho_ever + CHCpi_ex*rho[2]/rho_ever;
  pi_non = CHCpi_non/(1-HCVclear);
}


// Binomial regression model
model {
  // Priors
  rho ~ dirichlet(alpha);

  //////////////////////////////
  // Likelihood contributions //
  //////////////////////////////

  // Probability of HCV clearance
  HCVclear ~ normal(HCVclear_mean,HCVclear_sd);

  // Prevalence of current use
  rho[1] ~ normal(p_cur_mean,p_cur_sd);

  // Prevalence of ex-use
  rho[2] ~ normal(p_ex_mean,p_ex_sd);

  // Prevalence of chronic HCV among current users
  CHCpi_cur ~ normal(p_CHC_cur_mean,p_CHC_cur_sd);

  // Prevalence of HCV among ex users
  Yst_CHC_ever ~ binomial(Nst_CHC_ever,CHCpi_ever);
```

```
  // HCV+ among non
  Yst_hcv_non ~ binomial(Nst_hcv_non,pi_non);

  // SVR of DAAs among non-PWID
  SVR ~ normal(SVR_mean,SVR_sd);

  // SVR of DAAs among PWID
  SVR_PWID ~ normal(SVR_PWID_mean,SVR_PWID_sd);
}


generated quantities {
  // Functions of parameters
  real<lower=0,upper=100> overalCHC;
  real logit_CHCpi_cur;
  real logit_CHCpi_ex;
  real logit_CHCpi_non;
  real logit_rho_cur;
  real logit_rho_ex;
  real logit_rho_non;
  real<lower=0,upper=1> pEverGivenCHC;
  real<lower=0,upper=1> pCurGivenCHC;
  real<lower=0,upper=1> pExGivenCHC;
  real<lower=0,upper=1> pNonGivenCHC;
  real logit_HCVclear;
  real<lower=0> NumberCHC;

  real<lower=0,upper=1> CHCDAApi_non; // P(CHC+|Non) adjusted for DAA
  real<lower=0,upper=1> CHCDAApi_cur; // P(CHC+|Cur) adjusted for DAA
  real<lower=0,upper=1> CHCDAApi_ex; // P(CHC+|Ex) adjusted for DAA
  real<lower=0,upper=1> CHCDAApi_ever; // P(CHC+|Ever) adjusted for DAA
```

```
  real<lower=0,upper=100> overalCHC_DAA;
  real pEverGivenCHC_DAA;
  real pCurGivenCHC_DAA;
  real pExGivenCHC_DAA;
  real pNonGivenCHC_DAA;
  real<lower=0> NumberCHC_DAA;

  // Overall HCV prevalence
  overalCHC = 100*(rho[1]*CHCpi_cur + rho[2]*CHCpi_ex +  rho[3]*CHCpi_non
);
  pEverGivenCHC = CHCpi_ever*rho_ever/(overalCHC/100);
  pCurGivenCHC = CHCpi_cur*rho[1]/(overalCHC/100);
  pExGivenCHC = CHCpi_ex*rho[2]/(overalCHC/100);
  pNonGivenCHC = CHCpi_non*(1-rho_ever)/(overalCHC/100);
  NumberCHC = round(overalCHC*N1579/100);

  CHCDAApi_non = ( N1579*rho[3]*CHCpi_non - 0)/(N1579*rho[3]);
  CHCDAApi_cur = ( N1579*rho[1]*CHCpi_cur - NDAA_PWID*(pCurGivenCHC/(pCur
GivenCHC+pExGivenCHC))*SVR_PWID_mean)/(N1579*rho[1]);
  CHCDAApi_ex = ( N1579*rho[2]*CHCpi_ex - NDAA_PWID*(pExGivenCHC/(pCurGiv
enCHC+pExGivenCHC))*SVR_PWID_mean)/(N1579*rho[2]);
  CHCDAApi_ever = CHCDAApi_cur*rho[1]/rho_ever + CHCDAApi_ex*rho[2]/rho_e
ver;

  overalCHC_DAA = 100*(rho[1]*CHCDAApi_cur + rho[2]*CHCDAApi_ex + rho[3]*
CHCDAApi_non);
  pEverGivenCHC_DAA = CHCDAApi_ever*rho_ever/(overalCHC_DAA/100);
  pCurGivenCHC_DAA = CHCDAApi_cur*rho[1]/(overalCHC_DAA/100);
  pExGivenCHC_DAA = CHCDAApi_ex*rho[2]/(overalCHC_DAA/100);
  pNonGivenCHC_DAA = CHCDAApi_non*(1-rho_ever)/(overalCHC_DAA/100);
  NumberCHC_DAA = round(overalCHC_DAA*N1579/100);
```

```
  logit_rho_cur = logit(rho[1]);
  logit_rho_ex = logit(rho[2]);
  logit_rho_non = logit(rho[3]);
  logit_CHCpi_cur = logit(CHCpi_cur);
  logit_CHCpi_ex = logit(CHCpi_ex);
  logit_CHCpi_non = logit(CHCpi_non);
  logit_HCVclear = logit(HCVclear);
}
```

## Multi-state Markov model

```
#include <stdio.h>
#include <stdlib.h>
#include <string.h>
#include <math.h>
#include <time.h>

#define loops 100
int populationPerYearAndAge[2021][66];
double deathRatePerAge[66];
int totalPopulationPerYear[2021];

FILE *F_DeathRate=fopen("`Country`-deathRates.txt","r");
FILE *F_Population=fopen("`Country`-population.txt","r");
FILE *out;

double pa_start = 0.00012;                    // Setting Variable
double pa_step  = 0.000002;                   // Setting Variable
double pa_stop  = 0.000123;                   // Setting Variable

double pa;
double pg = 1.0/11.7;    // Assumed average active injecting career of  11
.7 years
double pk = 0.004*12.0;     // Relapse rate of 0.004/month

struct people{
    int age;
    int state;
};

char filename[100];
struct people person[50000000];
```

```
long int totalPersons=0;

void setPopulationAge()
{
    for( int j=0; j<71; j++){
        for( int i=0; i<51; i++){
            fscanf(F_Population,"%d",&populationPerYearAndAge[1950+j][15+
i]);
        }
    }
}

void setDeathRate()
{
    for( int i=0; i<51; i++){
        fscanf(F_DeathRate,"%lf",&deathRatePerAge[15+i*5]);
        for( int j=0; j<5; j++){
            deathRatePerAge[15+i*5+j] = deathRatePerAge[15+i*5];
        }
    }
}

void getTotalPopulationPerAge()
{
    for( int j=0; j<71; j++){
        for( int i=0; i<51; i++){
            totalPopulationPerYear[1950+j] += populationPerYearAndAge[195
0+j][15+i];
        }
    }
}
```

```
void printTotalPopulationPerAge()
{
    printf("---- - ----------\n");
    printf("Year - Population\n");
    printf("---- - ----------\n");
    for( int i=1950; i<2021; i++){
        printf("%d - %d\n",i,totalPopulationPerYear[i]);
    }
}

void printDeathRatePerAge()
{
    printf("--- - -----\n");
    printf("Age - Rate\n");
    printf("--- - -----\n");
    for( int i=15; i<65; i++){
        printf("%d - %lf\n",i,deathRatePerAge[i]);
    }
}

void printTotalPersonPerState( int year)
{
    int count[4]= {0,0,0,0};
    for( int i=0; i<totalPersons; i++){
        count[person[i].state]++;
    }
    //printf("%d\t%ld\t%ld\t%ld\t%ld\n",year,count[0],count[1],count[2],c
ount[3]);
    if(year==2014)
        printf("%d\t%ld\t%ld\t%ld\t%ld\t%lf\n",year,count[0],count[1],cou
nt[2],count[3],pa);
    if( year>2009){
```

```
        fprintf(out, "%d\t%ld\t%ld\t%ld\t%ld\n",year,count[0],count[1],co
unt[2],count[3]);
    }
}

void initializePopulation()
{
    int cnt=0;
    for( int j=15; j<65; j++){
        for( int i=0; i<populationPerYearAndAge[1950][j]*100; i++){
            person[cnt].age=j;
            person[cnt].state=0;
            cnt++;
        }
    }
    totalPersons = cnt;
}

bool checkRate(double rate)
{
    if (rate <0.001){
        double k = rand()/(1.00+RAND_MAX);
        if(k<0.001){
            rate = rate*1000;
        }
        else{
            return false;
        }
    }
    double k = rand()/(1.00+RAND_MAX);
    if ( k < rate )
        return true;
```

```
    else
        return false;
}

void changeStatusAndAge()
{
    for( int i=0; i<totalPersons; i++){
        // change state
        double k = rand()/(1.00+RAND_MAX);
        if( person[i].state==0){
            if ( checkRate(pa) &&  person[i].age<40){
                person[i].state=1;
            }
        }
        else if( person[i].state==1){
            if ( k < pg ){
                person[i].state=2;
            }
        }
        else if( person[i].state==2){
            if ( k < pk ){
                person[i].state=1;
            }
        }
        // After their 49th year, all active PWID are assumed to cease in
jecting
        if(person[i].age>49 && person[i].state==1){
            person[i].state=2;
        }
        // After their 64th year, remove
        if(person[i].age>64){
            person[i].state=3;
```

```
        }
        // death rate depending on age and state
        k = rand()/(1.00+RAND_MAX);
        if( person[i].state==0){
            if ( k < deathRatePerAge[person[i].age] ){
                person[i].state=3;
            }
        }
        else if( person[i].state==1){
            if ( k < deathRatePerAge[person[i].age] * 13.0 ){
                person[i].state=3;
            }
        }
        else if( person[i].state==2){
            if ( k < deathRatePerAge[person[i].age] ){
                person[i].state=3;
            }
        }
        // increase age one year
        person[i].age++;
    }
}

void addNewPersons(int year)
{
    // add new person [15 years old and non PWID]
    int cnt=totalPersons;
    for( int i=0; i<populationPerYearAndAge[year+1][15]*100; i++){
// 100 instead of 1000 for the population to reduce execution time
        person[cnt].age=15;
        person[cnt].state=0;
        cnt++;
```

```
    }
    // increase the total number of population
    totalPersons = cnt;
}
int main()
{
    srand(time(NULL));

    setPopulationAge();
    setDeathRate();

    getTotalPopulationPerAge();
    pa = pa_start;
    while ( pa < pa_stop){

        snprintf(filename, 100, "result_%lf.txt",pa);
        out=fopen(filename,"w");

        for( int iter=0; iter<loops ;iter++){
            initializePopulation();
            for( int year=1950; year<2020; year++){
                printTotalPersonPerState(year);
                changeStatusAndAge();
                addNewPersons(year);
            }
        }

        fclose(out);
        pa = pa + pa_step;
    }
```

```
}
```

## REFERENCES

Christensen, Peer Brehm, Gordon Hay, Peter Jepsen, Lars Haukali Omland, Søren Andreas Just, Henrik Bygum Krarup, Nina Weis, Niels Obel, and Susan Cowan. 2012. "Hepatitis c Prevalence in Denmark -an Estimate Based on Multiple National Registers." *BMC Infectious Diseases* 12 (1): 178. https://doi.org/10.1186/1471-2334-12-178.

Christensen, Peer Brehm, Jacob Søholm, and Anne Øvrehus. 2021. "Fra Behandling i Blinde Mod Elimination Af Hepatitis c." *Ugeskr Læger* 183: V02210145.

European Centre for Disease Prevention and Control. 2016. "Epidemiological Assessment of Hepatitis b and c Among Migrants in the EU/EEA." https://www.ecdc.europa.eu/sites/default/files/media/en/publications/Publications/epidemiological-assessment-hepatitis-B-and-C-among-migrants-EU-EEA.pdf.

European Centre for Disease Prevention and Control (ECDC). 2016. "Systematic Review on Hepatitis b and c Prevalence in the EU/EEA." https://www.ecdc.europa.eu/sites/default/files/media/en/publications/Publications/systematic-review-hepatitis-B-C-prevalence.pdf.

———. 2021. "Hepatitis c Prevalence Database." https://www.ecdc.europa.eu/en/all-topics-z/hepatitis-c/tools/hepatitis-c-prevalence-database.

Graf, Christiana, Marcus M Mücke, Georg Dultz, Kai-Henrik Peiffer, Alica Kubesch, Patrick Ingiliz, Stefan Zeuzem, Eva Herrmann, and Johannes Vermehren. 2019. "Efficacy of Direct-acting Antivirals for Chronic Hepatitis C Virus Infection in People Who Inject Drugs or Receive Opioid Substitution Therapy: A Systematic Review and Meta-analysis." *Clinical Infectious Diseases* 70 (11): 2355–65. https://doi.org/10.1093/cid/ciz696.

Grebely, Jason, Sarah Larney, Amy Peacock, Samantha Colledge, Janni Leung, Matthew Hickman, Peter Vickerman, et al. 2019. "Global, Regional, and Country-Level Estimates of Hepatitis c Infection Among People Who Have Recently Injected Drugs." *Addiction* 114 (1): 150–66. https://doi.org/https://doi.org/10.1111/add.14393.

Hines, Lindsey A., Adam Trickey, Janni Leung, Sarah Larney, Amy Peacock, Louisa Degenhardt, Samantha Colledge, et al. 2020. "Associations Between National Development Indicators and

the Age Profile of People Who Inject Drugs: Results from a Global Systematic Review and Meta-Analysis." *The Lancet Global Health* 8 (1): e76–91. https://doi.org/10.1016/S2214-109X(19)30462-0.

Lin, Lifeng, and Haitao Chu. 2020. "Meta-Analysis of Proportions Using Generalized Linear Mixed Models." *Epidemiology* 31 (5). https://journals.lww.com/epidem/Fulltext/2020/09000/Meta_analysis_of_Proportions_Using_Generalized.16.aspx.

McDonald, Scott A., Rosmawati Mohamed, Maznah Dahlui, Herlianna Naning, and Adeeba Kamarulzaman. 2014. "Bridging the Data Gaps in the Epidemiology of Hepatitis c Virus Infection in Malaysia Using Multi-Parameter Evidence Synthesis." *BMC Infectious Diseases* 14 (1): 564. https://doi.org/10.1186/s12879-014-0564-6.

Nielsen, Stine, Janne Fuglsang Hansen, Gordon Hay, Susan Cowan, Peter Jepsen, Lars Haukali Omland, Henrik Bygum Krarup, et al. 2020. "Hepatitis c Prevalence in Denmark in 2016—an Updated Estimate Using Multiple National Registers." *PLOS ONE* 15 (9): e0238203. https://doi.org/10.1371/journal.pone.0238203.

Ovrehus, Anne, Stine Nielsen, Janne Fuglsang Hansen, Dorte Kinggaard Holm, and Peer Christensen. 2019. "Test Uptake and Hepatitis c Prevalence in 5483 Danish People in Drug Use Treatment from 1996 to 2015: A Registry-Based Cohort Study." *Addiction* 114 (3): 494–503. https://doi.org/https://doi.org/10.1111/add.14479.

Presanis, Anne M., O. Noel Gill, Timothy R. Chadborn, Caterina Hill, Vivian Hope, Louise Logan, Brian D. Rice, Valerie C. Delpech, A. E. Ades, and Daniela De Angelis. 2010. "Insights into the Rise in HIV Infections, 2001 to 2008: A Bayesian Synthesis of Prevalence Evidence." *AIDS* 24 (18). https://journals.lww.com/aidsonline/Fulltext/2010/11270/Insights_into_the_rise_in_HIV_infections,_2001_to.12.aspx.

Sweeting, M. J., D. De Angelis, M. Hickman, and A. E. Ades. 2008. "Estimating hepatitis C prevalence in England and Wales by synthesizing evidence from multiple data sources. Assessing data conflict and model fit." *Biostatistics* 9 (4): 715–34. https://doi.org/10.1093/biostatistics/kxn004.

Tan, Sarah, Susanna Makela, Daliah Heller, Kevin Konty, Sharon Balter, Tian Zheng, and James H. Stark. 2018. “A Bayesian Evidence Synthesis Approach to Estimate Disease Prevalence in Hard-to-Reach Populations: Hepatitis c in New York City.” *Epidemics* 23: 96–109. https://doi.org/https://doi.org/10.1016/j.epidem.2018.01.002.

Thein, Hla-Hla, Qilong Yi, Gregory J. Dore, and Murray D. Krahn. 2008. “Estimation of Stage-Specific Fibrosis Progression Rates in Chronic Hepatitis c Virus Infection: A Meta-Analysis and Meta-Regression.” *Hepatology* 48 (2): 418–31. https://doi.org/https://doi.org/10.1002/hep.22375.

Veen, Maaike G. van, Anne M. Presanis, Stefano Conti, Maria Xiridou, Annemarie R. Stengaard, Martin C. Donoghoe, Ard I. van Sighem, Marianne A. van der Sande, and Daniela De Angelis. 2011. “National Estimate of HIV Prevalence in the Netherlands: Comparison and Applicability of Different Estimation Tools.” *AIDS* 25 (2). https://journals.lww.com/aidsonline/Fulltext/2011/01140/National_estimate_of_HIV_prevalence_in_the.14.aspx.

# Estimating the prevalence of chronic hepatitis C infection (CHC) in Estonia using Bayesian multiparameter evidence synthesis

19/05/2022

## Table of Contents


Introduction ........ 2
Methods ........ 2
Prevalence of ex-PWID ........ 3
Prevalence of recent PWID ........ 3
Prevalence of CHC among recent PWID ........ 4
Prevalence of CHC among ex-PWID ........ 4
Prevalence of CHC among non-PWID ........ 5
Results ........ 6
Tables and Figures ........ 7
Appendix ........ 12
Fit of the multi-state Markov model ........ 12
Stan code for Bayesian multiparameter evidence synthesis ........ 13
Multi-state Markov model ........ 17
References ........ 25

# INTRODUCTION

Patients with chronic hepatitis C (HCV) infection often remain asymptomatic for decades and even for life, but a proportion of them develop active chronic hepatitis, which is a progressive disease (Thein et al. 2008). Because of the largely asymptomatic nature of HCV infection, notification data reflect national screening and testing practices and do not give accurate insights into the prevalence of HCV infection. Although there are studies that have estimated the prevalence of HCV antibodies (anti-HCV) in specific groups such as the general population, first-time blood donors, or people who inject drugs (PWID) at drug treatment centers, those alone cannot be combined to produce national estimates unless some additional information regarding the composition of each HCV risk group and its prevalence in the population are known (Sweeting et al. 2008; McDonald et al. 2014; Tan et al. 2018).

# METHODS

Bayesian multiparameter evidence synthesis (MPES) has been a popular approach to formally estimate anti-HCV or HIV prevalence (Sweeting et al. 2008; McDonald et al. 2014; Tan et al. 2018; Presanis et al. 2010; Veen et al. 2011). In this project, we extend this method to estimating the CHC prevalence, with the population (15-79 years) split into three main non-overlapping risk groups, i.e., recent PWID (those who have injected in the last year), ex-PWID, and non-PWID. A unified model is assumed including parameters associated with the CHC prevalence of recent, ex-PWID, and non-PWID (denoted by $\pi_{rec}$, $\pi_{ex}$, and $\pi_{non}$, respectively) and the prevalence of these risk groups in the population (denoted by $\rho_{rec}$, $\rho_{ex}$, and $\rho_{non}$, respectively; $\rho_{rec} + \rho_{ex} + \rho_{non} = 1$). To estimate the CHC prevalence in the whole population, $\pi$, we used the formula:

$$\pi = \pi_{rec}\rho_{rec} + \pi_{ex}\rho_{ex} + \pi_{non}\rho_{non} \quad (1)$$

Bayesian synthesis considers all available data, allowing for an evidence-based estimation of CHC prevalence in the population, with inherent uncertainty properly accounted for. Our aim is to estimate the CHC prevalence for the total (15-79) population of Estonia in 2019 using sources of information over the last decade (2010-2019).

## Prevalence of ex-PWID

Data on the prevalence of ex-PWID are sparse and generally often unreliable. To overcome this limitation, we apply the method proposed by McDonald et al. (2014), which requires simulating a multi-state Markov model representing the non-PWID, recent PWID, and ex-PWID. In the multi-state Markov model, the simulation starts in 1950 and examines the population aged 15-79 years old. A certain number of 15-year-old individuals enter the simulation process each year. These numbers of 15-year-old individuals are obtained from the United Nations Statistic Division UNSD, being different for each country. The ageing of the population is also taken into account. Country-specific overall mortality data are retrieved from the Life Tables of the World Health Organization WHO. The duration of injecting career is also country-specific and obtained from the paper of Hines et al. (2020).

To estimate the number of ex-PWID, the model can be calibrated on the number of recent PWID provided in the EMCDDA barometer or the EMCDDA statistical bulletin (the code of the Markov model can be found in the Appendix). If there are no available data in the barometer, estimates from the systematic review of Grebely et al. (2019) could be used. Alternatively, if the national focal points suggest or provide different and updated or more accurate data for calibration purposes, we will consider their advice and adjust the model accordingly.

After applying the model for Estonia, we compute the number (and the corresponding Confidence Interval - CI) of ex-PWID in 2019. The number of ex-PWID is then divided by the population size (15-79 years) in the same year, and a Normal distribution, constrained such that $\rho_{ex} \in (0,1)$, is assumed for $\rho_{ex}$. The standard deviation of this distribution is specified to approximately correspond to the respective CI, i.e. $(UB_{\rho_{ex}} - LB_{\rho_{ex}})/(2 \times 1.96)$, where $UB_{\rho_{ex}}$ and $LB_{\rho_{ex}}$ denote the upper and lower limit of the CI.

## Prevalence of recent PWID

The number of recent PWID in Estonia in 2019 is also projected by the multi-state Markov model described in the previous subsection. To estimate $\rho_{rec}$, the number of recent PWID is divided by the population size (15-79 years). Similar to the previous subsection, a Normal distribution, constrained such that $\rho_{rec} \in (0,1)$, is assumed for $\rho_{rec}$, with the standard deviation specified to correspond to the CI obtained from the multi-state Markov model.

## Prevalence of CHC among recent PWID

The prevalence (and the 95% CI) of CHC among recent PWID can be informed from studies in the EMCDDA statistical bulletin. If information on the CHC prevalence is available, the Binomial distribution in the model to inform $\pi_{rec}$ was used. However, as these EMCDDA data typically refer to the anti-HCV prevalence and not to the CHC prevalence, they could not be used directly. This issue is addressed by noting that spontaneous viral clearance occurs in approximately one in four people with acute hepatitis C (Micallef, Kaldor, and Dore 2006). To account for the variability of the HCV clearance probabilities, we used the result reported in Micallef, Kaldor, and Dore (2006), i.e., the proportion of HCV clearance (denoted by $\rho_{clear}$) is equal to 0.26 (95% CI 0.22–0.29). Thus, estimates of the CHC prevalence among recent PWID ($\pi_{rec}$) can be obtained using the formula $\pi_{rec} = \pi(\text{anti-HCV})_{rec}(1 - \rho_{clear})$, where $\pi(\text{anti-HCV})_{rec}$ denotes the anti-HCV prevalence among recent PWID.

Apart from spontaneous clearance, some people may have been treated with direct acting antivirals (DAAs). Since treatment data may be weak and unstandardised or unavailable for most EU/EEA countries, we have not formally taken treatment into account.

If information on the HCV prevalence (CHC or anti-HCV) among recent PWID in the EMCDDA database is not available, the required information is obtained from the paper of Grebely et al. (2019). Currently, CHC prevalence data from three respondent-driven studies conducted in harm reduction centers in Tallinn in 2017 , Narva in 2018, and Kohtla-Järve in 2020.

## Prevalence of CHC among ex-PWID

Information on CHC prevalence among ex-PWID (i.e., $\pi_{ex}$) is difficult to obtain directly. To overcome this, nationwide data on the HCV prevalence among ever users through the EMCDDA database was utilized. However, if these data refer to the anti-HCV prevalence, they are adjusted according to the procedure described in the previous subsection, i.e. $\pi_{ever} = \pi(\text{anti-HCV})_{ever}(1 - \rho_{clear})$, where $\pi(\text{anti-HCV})_{ever}$ denotes the anti-HCV prevalence among ever PWID. In any case, if an estimate of $\pi_{ever}$ is available, $\pi_{ex}$ can be indirectly estimated since $\pi_{ever}$ is equal to a weighted average of $\pi_{rec}$ and $\pi_{ex}$, as shown by the following formula

$$\pi_{ever} = \frac{\rho_{rec}}{\rho_{rec} + \rho_{ex}}\pi_{rec} + \frac{\rho_{ex}}{\rho_{rec} + \rho_{ex}}\pi_{ex}, \quad (2)$$

or, equivalently,

$$\pi_{ex} = \left(\pi_{ever} - \frac{\rho_{rec}}{\rho_{rec} + \rho_{ex}}\pi_{rec}\right) \times \frac{\rho_{rec} + \rho_{ex}}{\rho_{ex}}, \quad (3)$$

Currently, as Estonia may lack data on the anti-HCV prevalence of ever PWID, anti-HCV prevalence data from ever PWID from Latvia and Lithuania, available in the EMCDDA statistical bulletin, were used.

## Prevalence of CHC among non-PWID

To estimate the CHC prevalence among non-PWID in a country (after 2010), the ECDC database (European Centre for Disease Prevention and Control (ECDC) 2021) was used. The ECDC group has carefully and critically collected information from relevant studies across the EU/EEA region in cooperation with the focal points of each country. On the basis of the representativeness of each study and other factors (European Centre for Disease Prevention and Control (ECDC) 2016), a risk of bias score was assigned to each study, with higher values indicating higher-quality studies. When higher-quality studies are available in a country (≥ 4), they are used to estimate the overall CHC prevalence in that country among non-PWID. If there are no higher-quality studies, general population estimates with a lower quality (<4) are pooled. If no general population prevalence estimates are available, data on the prevalence of HCV in pregnant women or military forces could be used as a prevalence measure. Finally, if data on pregnant women or military forces are lacking too, first-time blood donor studies could be an option. However, if the national focal point agrees or recommends, estimates from a neighboring country could be used instead, or we could use any other relevant information provided/suggested by the national focal point.

We directly use CHC prevalence data from studies in the general population, if available. If the data in the general population refer to anti-HCV prevalence (without any data on the viremic population), we adjust the estimates to get CHC prevalence based on the spontaneous HCV clearance estimate of 26%, described in the previous subsection.

As $\rho_{rec}$ and $\rho_{ex}$ are likely to be low, $\pi_{non}$ will be the most critical factor for the estimation of the overall CHC prevalence in the population. To adjust for potential heterogeneity between the CHC prevalence estimates from different studies in the non-PWID population in a country, as a

sensitivity analysis, we also apply a random-effect meta-analytic approach to pool the CHC prevalence across studies on non-PWID (Lin and Chu 2020). The random-effect approach is carried out only when at least 3 studies are available in the general population of a certain country, and the estimates are compared with the corresponding estimates from the approach assuming no heterogeneity (similar to a fixed-effect meta-analysis). If the estimates are inconsistent, possible explanations are investigated and discussed. If there is substantial heterogeneity between studies, it would help more to explore the causes; for example, following national focal point suggestions, some studies could be excluded to reduce heterogeneity.

# RESULTS

The above-mentioned approach was used to estimate the prevalence of CHC in 2019 (considering ages between 15-79 years: 1,032,680).

The aggregated data used by our approach are briefly presented in Table 1. The multi-state Markov model was calibrated on the estimated prevalence of recent PWID reported in the EMCDDA barometer. In Estonia, there were 3 studies on non-PWID of medium quality, which included only anti-HCV data (D. Parker et al. 2015; David. Parker and Ruutel 2017). To estimate the CHC prevalence in the general population (primarily non-PWID), we used a fixed-effect meta-analytic approach, with the corresponding results presented in Table 2. The prevalence of recent and ex-PWID was low in Estonia (about 0.79% and 2.45%, respectively) corresponding to 8,185 (95% CI: 7,590-8,710) recent PWID and 25,260 (95% CI: 24,320-26,210) ex-PWID in the population. However, the CHC prevalence in these groups was substantial (Table 2) being 53.97% and 42.08%, respectively. This translates to 4,416 (95% CI: 4,012-4,831) and 10,623 (95% CI: 9,507-11,781) recent and ex-PWID aged 15-79 living with CHC infection in Estonia in 2019. The CHC prevalence in the general population was 0.26% (95% CI: 0.08%-0.61%), much lower than that of the high-risk groups. Taking all pieces of information into account, the overall CHC prevalence in Estonia in 2019 was equal to 1.71% (95% CI: 1.49%-2.06%), which corresponds to 17,634 (95% CI: 15,413-21,306) individuals aged 15-79 years with CHC infection. The corresponding results under a random-effect meta-analysis for the studies in the general population were similar and are provided in Table 3.

# TABLES AND FIGURES

*Table 1. Data (real) contributing to the estimation of CHC prevalence in Estonia in 2019.*

| Parameter | Number (95%CI) | Numerator | Denominator | Notes | Year of study |
|---|---|---|---|---|---|
| $\rho_{rec}$ | 8,185 (7,590-8,710) | | | Method based on McDonald et al. | 2019 |
| $\rho_{ex}$ | 25,260 (24,320-26,210) | | | Method based on McDonald et al. | 2019 |
| $\pi_{rec}$ | | 59 | 112 | Country feedback document (RDS in Tallinn) | 2017 |
| $\pi_{rec}$ | | 145 | 350 | Country feedback document (RDS in Narva) | 2018 |
| $\pi_{rec}$ | | 234 | 350 | Country feedback document (RDS in Kohtla-Jarve) | 2020 |
| $\pi(\text{anti-HCV})_{ever}$ | | 629 | 1,035 | EMCDDA database (Latvia/Lithuania) | 2019 |
| $\pi(\text{anti-HCV})_{non}$ | | 0 | 185 | ECDC database (David. Parker and Ruutel 2017); Risk of bias=2 | 2013 |
| $\pi(\text{anti-HCV})_{non}$ | | 1 | 584 | ECDC database (D. Parker et al. 2015); Risk of bias=NA | 2012 |

| Parameter | Number (95%CI) | Numerator | Denominator | Notes | Year of study |
|---|---|---|---|---|---|
| $\pi(\text{anti-HCV})_{non}$ | | 2 | 298 | ECDC database (Country feedback document); Risk of bias=NA | 2018 |

*Notes*: Higher risk of bias score denotes a higher-quality study (range from 0 to 6);

*Table 2. Results from the method assuming no heterogeneity in the estimation of the CHC prevalence in the general population from different studies (i.e., similar to a fixed-effect meta-analysis).*

| Parameter | Posterior median | Posterior sd | LB (2.5%) | UB (97.5%) |
|---|---|---|---|---|
| $\rho_{rec}$ (%) | 0.79 | 0.03 | 0.74 | 0.85 |
| $\rho_{ex}$ (%) | 2.45 | 0.05 | 2.35 | 2.54 |
| $\pi_{rec}$ (%) | 53.97 | 1.74 | 50.52 | 57.38 |
| $\pi_{ex}$ (%) | 42.08 | 2.15 | 37.89 | 46.29 |
| $\pi_{non}$ (%) | 0.26 | 0.14 | 0.08 | 0.61 |
| $\pi$ (%) | 1.71 | 0.15 | 1.49 | 2.06 |
| Number with CHC | 17,634 | 1,512 | 15,413 | 21,306 |
| Pr(Ever PWID\|CHC) (%) | 85.48 | 6.24 | 71.19 | 95.16 |
| Pr(Non-PWID\|CHC) (%) | 14.52 | 6.24 | 4.84 | 28.81 |

*Notes*: $\rho_{rec}$, prevalence of recent PWID; $\rho_{ex}$, prevalence of ex-PWID; $\pi_{rec}$, CHC prevalence among recent PWID; $\pi_{ex}$, CHC prevalence among ex-PWID; $\pi_{non}$, CHC prevalence among non-PWID; $\pi$ overall CHC prevalence in Estonia; LB, Lower Boundary; UP, Upper Boundary

*Table 3. Results from the method assuming heterogeneity in the estimation of the CHC prevalence in the general population from different studies (i.e., similar to a random-effect meta-analysis).*

| Parameter | Posterior median | Posterior sd | LB (2.5%) | UB (97.5%) |
|---|---|---|---|---|
| $\rho_{rec}$ (%) | 0.79 | 0.03 | 0.74 | 0.85 |
| $\rho_{ex}$ (%) | 2.45 | 0.05 | 2.36 | 2.54 |
| $\pi_{rec}$ (%) | 53.93 | 1.74 | 50.55 | 57.31 |
| $\pi_{ex}$ (%) | 42.05 | 2.14 | 37.89 | 46.33 |
| $\pi_{non}$ (%) | 0.13 | 0.82 | 0.01 | 1.19 |
| $\pi$ (%) | 1.59 | 0.8 | 1.41 | 2.61 |
| Number with CHC | 16,466 | 8,223 | 14,519 | 26,956 |
| Pr(Ever PWID\|CHC) (%) | 92 | 11.65 | 55.71 | 99.63 |
| Pr(Non-PWID\|CHC) (%) | 8 | 11.65 | 0.37 | 44.29 |
| Between-study variance | 1.98 | 3.45 | 0.12 | 13.68 |

*Notes*: $\rho_{rec}$, prevalence of recent PWID; $\rho_{ex}$, prevalence of ex-PWID; $\pi_{rec}$, CHC prevalence among recent PWID; $\pi_{ex}$, CHC prevalence among ex-PWID; $\pi_{non}$, CHC prevalence among non-PWID; $\pi$ overall CHC prevalence in Estonia; LB, Lower Boundary; UP, Upper Boundary

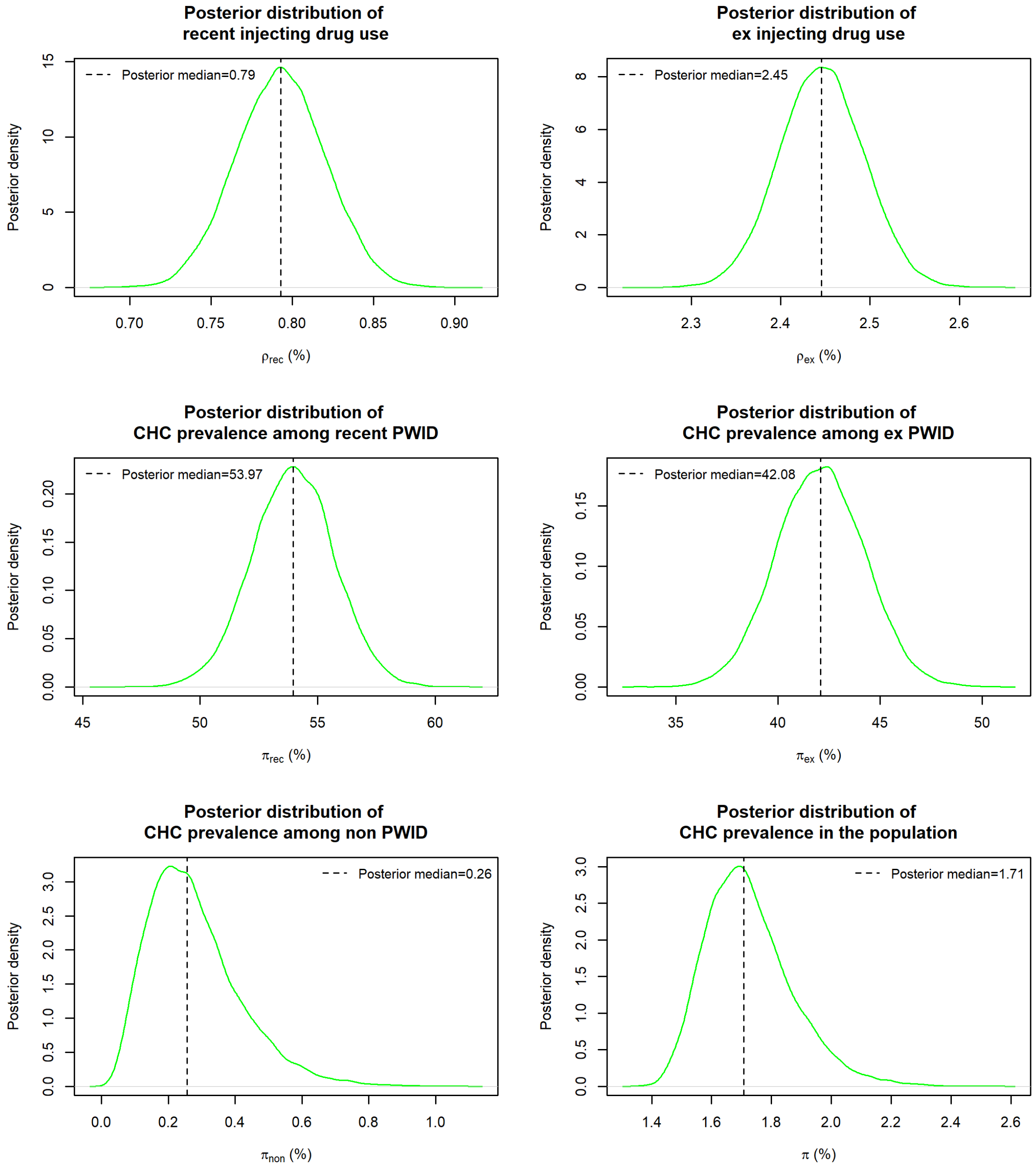


*Figure 1. Posterior distributions of the key model parameters using the approach that assumes no heterogeneity in the estimation of the CHC prevalence in the general population (primarily non-PWID) from different studies (i.e., similar to a fixed-effect meta-analysis).*

# APPENDIX

## Fit of the multi-state Markov model

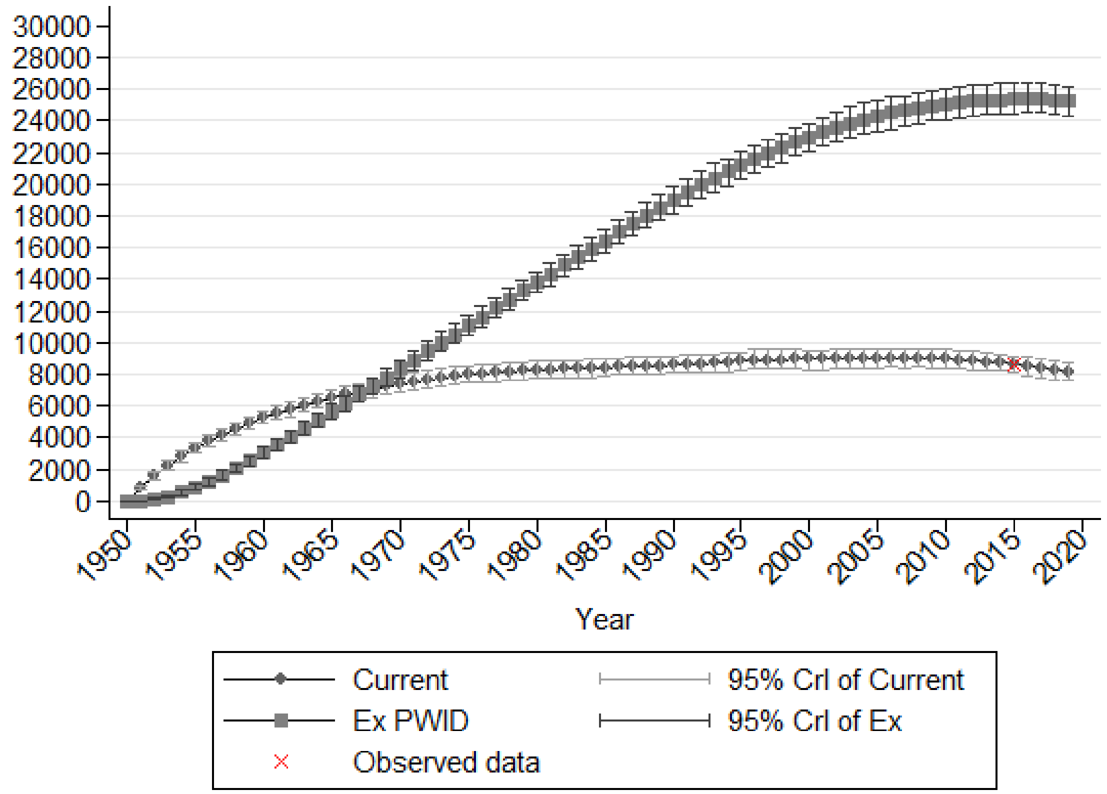


*Figure 2: Model predictions for the numbers of current and ex-PWID (people who inject drugs). The solid black line and shaded grey error bars show the median and 95% credible intervals (95% CrI) for the model projections. For comparison, asterisks indicate the observed country's data.*

## Stan code for Bayesian multiparameter evidence synthesis

```
data {
  int<lower=0> N1579; // Population of 15-79 in `Country`
  int<lower=1> Knon; // Number of studies in the ECDC data for `Country`
  int<lower=1> Kever; // Number of studies in the EMCDDA data for ever users in `Country`
  int<lower=1> Kcur; // Number of studies in the EMCDDA barometer (for HCV prevalence of PWID) in `Country`

  real p_cur_mean; // Prior mean for the prevalence of current use in `Country`
  real<lower=0> p_cur_sd; // Prior sd for the prevalence of current use in `Country`

  real p_ex_mean; // Prior mean for the prevalence of ex-use in `Country`
  real<lower=0> p_ex_sd; // Prior sd for the prevalence of ex-use in `Country`

  int<lower=0> Nst_CHC_cur[Kcur]; // Number of individuals in the study estimating CHC among recent PWID
  int<lower=0> Yst_CHC_cur[Kcur]; // Number of HCV+ in the study estimating CHC among recent PWID

  int<lower=0> Nst_hcv_ever[Kever]; // Number of individuals in the study estimating anti-HCV among ever IDU
  int<lower=0> Yst_hcv_ever[Kever]; // Number of HCV+ in the study estimating anti-HCV among ever PWID

  int<lower=0> Nst_hcv_non[Knon]; // Number of individuals in the study estimating anti-HCV among non PWID
  int<lower=0> Yst_hcv_non[Knon]; // Number of HCV+ in the study estimating anti-HCV among non PWID
```

```
  vector<lower=0>[3] alpha; // parameter of the Diriclet prior

  real HCVclear_mean; // Prior mean for the HCV clearance probability
  real<lower=0> HCVclear_sd; // Prior sd for the HCV clearance probabilit
y
}

// Block defining the original parameters
parameters {
  // The parameters to be sampled
  simplex[3] rho; // Prevalence of the three risk groups
  real<lower=0,upper=1> CHCpi_cur; // P(CHC+|Cur)
  real<lower=0,upper=1> CHCpi_ex; // P(CHC+|Ex)
  real<lower=0,upper=1> CHCpi_non; // P(CHC+|Non)

  real<lower=0,upper=1-CHCpi_cur> HCVclear; // Probability of HCV clearan
ce; upper bound = 1-prevalence of chronic HCV
}



transformed parameters {
  // Change scales
  real<lower=0,upper=1> rho_ever;
  real<lower=0,upper=1> CHCpi_ever;
  real<lower=0,upper=1> pi_ever;
  real<lower=0,upper=1> pi_non;
  real<lower=0,upper=1> pi_cur;

  rho_ever = rho[1] + rho[2];
  CHCpi_ever = CHCpi_cur*rho[1]/rho_ever + CHCpi_ex*rho[2]/rho_ever;
  pi_ever = CHCpi_ever/(1-HCVclear);
```

```
  pi_non = CHCpi_non/(1-HCVclear);
  pi_cur = CHCpi_cur/(1-HCVclear);
}


// Binomial regression model
model {
  // Priors
  rho ~ dirichlet(alpha);

  //////////////////////////////
  // Likelihood contributions //
  //////////////////////////////

  // Probability of HCV clearance
  HCVclear ~ normal(HCVclear_mean,HCVclear_sd);

  // Prevalence of current use
  rho[1] ~ normal(p_cur_mean,p_cur_sd);

  // Prevalence of ex-use
  rho[2] ~ normal(p_ex_mean,p_ex_sd);

  // Prevalence of chronic HCV among current users
  Yst_CHC_cur ~ binomial(Nst_CHC_cur,CHCpi_cur);

  // Prevalence of HCV among ever users
  Yst_hcv_ever ~ binomial(Nst_hcv_ever,pi_ever);

  // HCV+ among non
  Yst_hcv_non ~ binomial(Nst_hcv_non,pi_non);
}
```

```
generated quantities {
  // Functions of parameters
  real<lower=0,upper=100> overalCHC;
  real logit_CHCpi_cur;
  real logit_CHCpi_ex;
  real logit_CHCpi_non;
  real logit_rho_cur;
  real logit_rho_ex;
  real logit_rho_non;
  real<lower=0,upper=1> pEverGivenCHC;
  real<lower=0,upper=1> pNonGivenCHC;
  real logit_HCVclear;
  real<lower=0> NumberCHC;

  // Overall HCV prevalence
  overalCHC = 100*(rho[1]*CHCpi_cur + rho[2]*CHCpi_ex +  rho[3]*CHCpi_non
);
  pEverGivenCHC = CHCpi_ever*rho_ever/(overalCHC/100);
  pNonGivenCHC = CHCpi_non*(1-rho_ever)/(overalCHC/100);
  NumberCHC = round(overalCHC*N1579/100);

  logit_rho_cur = logit(rho[1]);
  logit_rho_ex = logit(rho[2]);
  logit_rho_non = logit(rho[3]);
  logit_CHCpi_cur = logit(CHCpi_cur);
  logit_CHCpi_ex = logit(CHCpi_ex);
  logit_CHCpi_non = logit(CHCpi_non);
  logit_HCVclear = logit(HCVclear);
}
```

## Multi-state Markov model

```
#include <stdio.h>
#include <stdlib.h>
#include <string.h>
#include <math.h>
#include <time.h>

#define loops 100
int populationPerYearAndAge[2021][66];
double deathRatePerAge[66];
int totalPopulationPerYear[2021];

FILE *F_DeathRate=fopen("`Country`-deathRates.txt","r");
FILE *F_Population=fopen("`Country`-population.txt","r");
FILE *out;

double pa_start = 0.00012;                    // Setting Variable
double pa_step  = 0.000002;                   // Setting Variable
double pa_stop  = 0.000123;                   // Setting Variable

double pa;
double pg = 1.0/11.7;    // Assumed average active injecting career of  11
.7 years
double pk = 0.004*12.0;     // Relapse rate of 0.004/month

struct people{
    int age;
    int state;
};

char filename[100];
struct people person[50000000];
```

```
long int totalPersons=0;

void setPopulationAge()
{
    for( int j=0; j<71; j++){
        for( int i=0; i<51; i++){
            fscanf(F_Population,"%d",&populationPerYearAndAge[1950+j][15+
i]);
        }
    }
}

void setDeathRate()
{
    for( int i=0; i<51; i++){
        fscanf(F_DeathRate,"%lf",&deathRatePerAge[15+i*5]);
        for( int j=0; j<5; j++){
            deathRatePerAge[15+i*5+j] = deathRatePerAge[15+i*5];
        }
    }
}

void getTotalPopulationPerAge()
{
    for( int j=0; j<71; j++){
        for( int i=0; i<51; i++){
            totalPopulationPerYear[1950+j] += populationPerYearAndAge[195
0+j][15+i];
        }
    }
}
```

```
void printTotalPopulationPerAge()
{
    printf("---- - ----------\n");
    printf("Year - Population\n");
    printf("---- - ----------\n");
    for( int i=1950; i<2021; i++){
        printf("%d - %d\n",i,totalPopulationPerYear[i]);
    }
}

void printDeathRatePerAge()
{
    printf("--- - -----\n");
    printf("Age - Rate\n");
    printf("--- - -----\n");
    for( int i=15; i<65; i++){
        printf("%d - %lf\n",i,deathRatePerAge[i]);
    }
}

void printTotalPersonPerState( int year)
{
    int count[4]= {0,0,0,0};
    for( int i=0; i<totalPersons; i++){
        count[person[i].state]++;
    }
    //printf("%d\t%ld\t%ld\t%ld\t%ld\n",year,count[0],count[1],count[2],c
ount[3]);
    if(year==2014)
        printf("%d\t%ld\t%ld\t%ld\t%ld\t%lf\n",year,count[0],count[1],cou
nt[2],count[3],pa);
    if( year>2009){
```

```
        fprintf(out, "%d\t%ld\t%ld\t%ld\t%ld\n",year,count[0],count[1],co
unt[2],count[3]);
    }
}

void initializePopulation()
{
    int cnt=0;
    for( int j=15; j<65; j++){
        for( int i=0; i<populationPerYearAndAge[1950][j]*100; i++){
            person[cnt].age=j;
            person[cnt].state=0;
            cnt++;
        }
    }
    totalPersons = cnt;
}

bool checkRate(double rate)
{
    if (rate <0.001){
        double k = rand()/(1.00+RAND_MAX);
        if(k<0.001){
            rate = rate*1000;
        }
        else{
            return false;
        }
    }
    double k = rand()/(1.00+RAND_MAX);
    if ( k < rate )
        return true;
```

```
    else
        return false;
}

void changeStatusAndAge()
{
    for( int i=0; i<totalPersons; i++){
        // change state
        double k = rand()/(1.00+RAND_MAX);
        if( person[i].state==0){
            if ( checkRate(pa) &&  person[i].age<40){
                person[i].state=1;
            }
        }
        else if( person[i].state==1){
            if ( k < pg ){
                person[i].state=2;
            }
        }
        else if( person[i].state==2){
            if ( k < pk ){
                person[i].state=1;
            }
        }
        // After their 49th year, all active PWID are assumed to cease in
jecting
        if(person[i].age>49 && person[i].state==1){
            person[i].state=2;
        }
        // After their 64th year, remove
        if(person[i].age>64){
            person[i].state=3;
```

```
        }
        // death rate depending on age and state
        k = rand()/(1.00+RAND_MAX);
        if( person[i].state==0){
            if ( k < deathRatePerAge[person[i].age] ){
                person[i].state=3;
            }
        }
        else if( person[i].state==1){
            if ( k < deathRatePerAge[person[i].age] * 13.0 ){
                person[i].state=3;
            }
        }
        else if( person[i].state==2){
            if ( k < deathRatePerAge[person[i].age] ){
                person[i].state=3;
            }
        }
        // increase age one year
        person[i].age++;
    }
}

void addNewPersons(int year)
{
    // add new person [15 years old and non PWID]
    int cnt=totalPersons;
    for( int i=0; i<populationPerYearAndAge[year+1][15]*100; i++){
// 100 instead of 1000 for the population to reduce execution time
        person[cnt].age=15;
        person[cnt].state=0;
        cnt++;
```

```
    }
    // increase the total number of population
    totalPersons = cnt;
}
int main()
{
    srand(time(NULL));

    setPopulationAge();
    setDeathRate();

    getTotalPopulationPerAge();
    pa = pa_start;
    while ( pa < pa_stop){

        snprintf(filename, 100, "result_%lf.txt",pa);
        out=fopen(filename,"w");

        for( int iter=0; iter<loops ;iter++){
            initializePopulation();
            for( int year=1950; year<2020; year++){
                printTotalPersonPerState(year);
                changeStatusAndAge();
                addNewPersons(year);
            }
        }

        fclose(out);
        pa = pa + pa_step;
    }
```

```
}
```

## REFERENCES

European Centre for Disease Prevention and Control (ECDC). 2016. “Systematic Review on Hepatitis b and c Prevalence in the EU/EEA.” https://www.ecdc.europa.eu/sites/default/files/media/en/publications/Publications/systematic-review-hepatitis-B-C-prevalence.pdf.

———. 2021. “Hepatitis c Prevalence Database.” https://www.ecdc.europa.eu/en/all-topics-z/hepatitis-c/tools/hepatitis-c-prevalence-database.

Grebely, Jason, Sarah Larney, Amy Peacock, Samantha Colledge, Janni Leung, Matthew Hickman, Peter Vickerman, et al. 2019. “Global, Regional, and Country-Level Estimates of Hepatitis c Infection Among People Who Have Recently Injected Drugs.” *Addiction* 114 (1): 150–66. https://doi.org/https://doi.org/10.1111/add.14393.

Hines, Lindsey A., Adam Trickey, Janni Leung, Sarah Larney, Amy Peacock, Louisa Degenhardt, Samantha Colledge, et al. 2020. “Associations Between National Development Indicators and the Age Profile of People Who Inject Drugs: Results from a Global Systematic Review and Meta-Analysis.” *The Lancet Global Health* 8 (1): e76–91. https://doi.org/10.1016/S2214-109X(19)30462-0.

Lin, Lifeng, and Haitao Chu. 2020. “Meta-Analysis of Proportions Using Generalized Linear Mixed Models.” *Epidemiology* 31 (5). https://journals.lww.com/epidem/Fulltext/2020/09000/Meta_analysis_of_Proportions_Using_Generalized.16.aspx.

McDonald, Scott A., Rosmawati Mohamed, Maznah Dahlui, Herlianna Naning, and Adeeba Kamarulzaman. 2014. “Bridging the Data Gaps in the Epidemiology of Hepatitis c Virus Infection in Malaysia Using Multi-Parameter Evidence Synthesis.” *BMC Infectious Diseases* 14 (1): 564. https://doi.org/10.1186/s12879-014-0564-6.

Micallef, J. M., J. M. Kaldor, and G. J. Dore. 2006. “Spontaneous Viral Clearance Following Acute Hepatitis c Infection: A Systematic Review of Longitudinal Studies.” *Journal of Viral Hepatitis* 13 (1): 34–41. https://doi.org/https://doi.org/10.1111/j.1365-2893.2005.00651.x.

Parker, David, Michael Regier, Joseph Widmeyer, and Kristi Ruutel. 2015. "HIV/STI Prevalence Study Among Military Conscripts in Estonia." *Journal of Community Health* 40 (2): 271–75. https://doi.org/10.1007/s10900-014-9926-7.

Parker, David., and Kristi Ruutel. 2017. "Sexually Transmitted Infections - Prevalence, Knowledge and Behaviours Among Professional Defence Forces in Estonia: A Pilot Study", Journal="central European Journal of Public Health" 25 (1): 11–14. https://doi.org/10.21101/cejph.a4498.

Presanis, Anne M., O. Noel Gill, Timothy R. Chadborn, Caterina Hill, Vivian Hope, Louise Logan, Brian D. Rice, Valerie C. Delpech, A. E. Ades, and Daniela De Angelis. 2010. "Insights into the Rise in HIV Infections, 2001 to 2008: A Bayesian Synthesis of Prevalence Evidence." *AIDS* 24 (18). https://journals.lww.com/aidsonline/Fulltext/2010/11270/Insights_into_the_rise_in_HIV_infections,_2001_to.12.aspx.

Sweeting, M. J., D. De Angelis, M. Hickman, and A. E. Ades. 2008. "Estimating hepatitis C prevalence in England and Wales by synthesizing evidence from multiple data sources. Assessing data conflict and model fit." *Biostatistics* 9 (4): 715–34. https://doi.org/10.1093/biostatistics/kxn004.

Tan, Sarah, Susanna Makela, Daliah Heller, Kevin Konty, Sharon Balter, Tian Zheng, and James H. Stark. 2018. "A Bayesian Evidence Synthesis Approach to Estimate Disease Prevalence in Hard-to-Reach Populations: Hepatitis c in New York City." *Epidemics* 23: 96–109. https://doi.org/https://doi.org/10.1016/j.epidem.2018.01.002.

Thein, Hla-Hla, Qilong Yi, Gregory J. Dore, and Murray D. Krahn. 2008. "Estimation of Stage-Specific Fibrosis Progression Rates in Chronic Hepatitis c Virus Infection: A Meta-Analysis and Meta-Regression." *Hepatology* 48 (2): 418–31. https://doi.org/https://doi.org/10.1002/hep.22375.

Veen, Maaike G. van, Anne M. Presanis, Stefano Conti, Maria Xiridou, Annemarie R. Stengaard, Martin C. Donoghoe, Ard I. van Sighem, Marianne A. van der Sande, and Daniela De Angelis. 2011. "National Estimate of HIV Prevalence in the Netherlands: Comparison and Applicability of Different Estimation Tools." *AIDS* 25 (2).

https://journals.lww.com/aidsonline/Fulltext/2011/01140/National_estimate_of_HIV_prevalence_in_the.14.aspx.

# Estimating the prevalence of chronic hepatitis C infection (CHC) in Finland using Bayesian multiparameter evidence synthesis

14/07/2022

## Table of Contents


Introduction ........ 2

Methods ........ 2

Prevalence of ex-PWID ........ 3

Prevalence of recent PWID ........ 3

Prevalence of CHC among recent PWID ........ 4

Prevalence of CHC among ex-PWID ........ 5

Prevalence of CHC among non-PWID ........ 5

Sensitivity analysis including migrants from endemic countries ........ 7

Results ........ 7

Tables and Figures ........ 9

Appendix ........ 17

Fit of the multi-state Markov model ........ 17

Stan code for Bayesian multiparameter evidence synthesis ........ 18

Multi-state Markov model ........ 24

References ........ 32

# INTRODUCTION

Patients with chronic hepatitis C (HCV) infection often remain asymptomatic for decades and even for life, but a proportion of them develop active chronic hepatitis, which is a progressive disease (Thein et al. 2008). Because of the largely asymptomatic nature of HCV infection, notification data reflect national screening and testing practices and do not give accurate insights into the prevalence of HCV infection. Although there are studies that have estimated the prevalence of HCV antibodies (anti-HCV) in specific groups such as the general population, first-time blood donors, or people who inject drugs (PWID) at drug treatment centers, those alone cannot be combined to produce national estimates unless some additional information regarding the composition of each HCV risk group and its prevalence in the population are known (Sweeting et al. 2008; McDonald et al. 2014; Tan et al. 2018).

# METHODS

Bayesian multiparameter evidence synthesis (BMES) has been a popular approach to formally estimate anti-HCV or HIV prevalence (Sweeting et al. 2008; McDonald et al. 2014; Tan et al. 2018; Presanis et al. 2010; Veen et al. 2011). In this project, we extend this method to estimating the CHC prevalence, with the population (15-79 years) split into three main non-overlapping risk groups, i.e., recent PWID (those who have injected in the last year), ex-PWID, and non-PWID. A unified model is assumed including parameters associated with the CHC prevalence of recent, ex-PWID, and non-PWID (denoted by $\pi_{rec}$, $\pi_{ex}$, and $\pi_{non}$, respectively) and the prevalence of these risk groups in the population (denoted by $\rho_{rec}$, $\rho_{ex}$, and $\rho_{non}$, respectively; $\rho_{rec} + \rho_{ex} + \rho_{non} = 1$). To estimate the CHC prevalence in the whole population, $\pi$, we used the formula:

$$\pi = \pi_{rec}\rho_{rec} + \pi_{ex}\rho_{ex} + \pi_{non}\rho_{non} \quad (1)$$

Bayesian synthesis considers all available data, allowing for an evidence-based estimation of CHC prevalence in the population, with inherent uncertainty properly accounted for. Our aim is to estimate the CHC prevalence for the total (15-79) population of Finland in 2019 using sources of information over the last decade (2010-2019).

## Prevalence of ex-PWID

Data on the prevalence of ex-PWID are sparse and generally often unreliable. To overcome this limitation, we apply the method proposed by McDonald et al. (2014), which requires simulating a multi-state Markov model representing the non-PWID, recent PWID, and ex-PWID. In the multi-state Markov model, the simulation starts in 1950 and examines the population aged 15-79 years old. A certain number of 15-year-old individuals enter the simulation process each year. These numbers of 15-year-old individuals are obtained from the United Nations Statistic Division UNSD, being different for each country. The ageing of the population is also taken into account. Country-specific overall mortality data are retrieved from the Life Tables of the World Health Organization WHO. The duration of injecting career is also country-specific and obtained from the paper of Hines et al. (2020). However, since no data for Finland are reported in Hines et al. (2020), we used the average duration of injecting career from Sweden, Norway, and Denmark.

To estimate the number of ex-PWID, the model can be calibrated on the number of recent PWID provided in the EMCDDA barometer or the EMCDDA statistical bulletin (the code of the Markov model can be found in the Appendix). If there are no available data in the barometer, estimates from the systematic review of Grebely et al. (2019) could be used. Currently, the multi-state Markov model was calibrated on the estimated prevalence of recent PWID reported in the EMCDDA barometer.

After applying the model for Finland, we compute the number (and the corresponding Confidence Interval - CI) of ex-PWID in 2019. The number of ex-PWID is then divided by the population size (15-79 years) in the same year, and a Normal distribution, constrained such that $\rho_{ex} \in (0,1)$, is assumed for $\rho_{ex}$. The standard deviation of this distribution is specified to approximately correspond to the respective CI, i.e. $\left(UB_{\rho_{ex}} - LB_{\rho_{ex}}\right)/(2 \times 1.96)$, where $UB_{\rho_{ex}}$ and $LB_{\rho_{ex}}$ denote the upper and lower limit of the CI.

## Prevalence of recent PWID

The number of recent PWID in Finland in 2019 is also projected by the multi-state Markov model described in the previous subsection. To estimate $\rho_{rec}$, the number of recent PWID is divided by the population size (15-79 years). Similar to the previous subsection, a Normal

distribution, constrained such that $\rho_{rec} \in (0,1)$, is assumed for $\rho_{rec}$, with the standard deviation specified to correspond to the CI obtained from the multi-state Markov model.

## Prevalence of CHC among recent PWID

The prevalence (and the 95% CI) of CHC among recent PWID was informed by national anti-HCV prevalence data from recent PWID in 2014 (Fraser et al. 2018), available also in the EMCDDA statistical bulletin. The Binomial distribution in the model to inform $\pi_{rec}$ was used. However, as the data reported in Fraser et al. (2018) refer to the anti-HCV prevalence and not to the CHC prevalence, they could not be used directly. This issue can be partly addressed by noting that spontaneous viral clearance occurs in approximately one in four people with acute hepatitis C (Micallef, Kaldor, and Dore 2006). To account for variability in the HCV clearance probabilities, we used the result reported in Micallef, Kaldor, and Dore (2006), i.e., the proportion of HCV clearance (denoted by $\rho_{clear}$) is equal to 0.26 (95% CI 0.22–0.29). However, some people may have been treated with direct-acting antivirals (DAAs), with the sustained virologic response ($SVR_{PWID}$) among PWID estimated to be 88% (95% CI: 80% to 93%) (Graf et al. 2019). Based on information provided by the national focal point, the number of individuals treated with DAAs up to 2019 is equal to 3,905. However, the proportions of each risk group among individuals treated with DAAs, i.e. $\rho_{rec|DAA}$, $\rho_{ex|DAA}$, and $\rho_{non|DAA}$ ($\rho_{rec|DAA} + \rho_{ex|DAA} + \rho_{non|DAA} = 1$), are not currently available in Finland. In this report, based on information provided by the focal point, we assume that the prior distribution of $\left(\rho_{rec|DAA}, \rho_{ex|DAA}, \rho_{non|DAA}\right)$ is the Dirichlet distribution with corresponding means 3%, 35%, and 62%, respectively. The Dirichlet distribution ensures that $\rho_{rec|DAA} + \rho_{ex|DAA} + \rho_{non|DAA} =$ 100%, whereas the associated 95% probability intervals for $\rho_{rec|DAA}$, $\rho_{ex|DAA}$, and $\rho_{non|DAA}$ are equal to (0.06%-11.23%), (19.19%-52.92%), and (44.28%-78.35%), respectively. Thus, the CHC prevalence among recent PWID, adjusted for DAAs, can be estimated by

$$\pi_{rec} = \frac{N_{15,79}\rho_{rec}\pi(\text{anti-HCV})_{rec}(1-\rho_{clear}) - N_{DAA}\rho_{rec|DAA}SVR_{PWID}}{N_{15,79}\rho_{rec}}, \quad (2)$$

where $\pi(\text{anti-HCV})_{rec}$ denotes the anti-HCV estimate derived solely from the data reported in Fraser et al. (2018).

## Prevalence of CHC among ex-PWID

Information on CHC prevalence among ex-PWID (i.e., $\pi_{ex}$) is difficult to obtain directly. To overcome this, national anti-HCV prevalence data from ever PWID in 2019, available in the EMCDDA statistical bulletin, were used. However, as these data refer to the anti-HCV prevalence, they are adjusted according to the procedure described in the previous subsection, i.e.

$$\pi_{ever} = \frac{N_{15,79}\rho_{ever}\pi(\text{anti-HCV})_{ever}(1-\rho_{clear}) - N_{DAA}\rho_{ever|DAA}SVR_{PWID}}{N_{15,79}\rho_{ever}}. \quad (3)$$

where $\pi(\text{anti-HCV})_{ever}$ denotes the anti-HCV prevalence among ever PWID, $\rho_{ever} = \rho_{rec} + \rho_{ex}$, $\rho_{clear}$ denotes the spontaneous viral clearance [assumed to be equal to 0.26 (95% CI 0.22–0.29); (Micallef, Kaldor, and Dore 2006)], and $\rho_{ever|DAA}$ the proportion of ever PWID among individuals treated with DAAs. Note that $\rho_{ever|DAA}$ is equal to $\rho_{rec|DAA} + \rho_{ex|DAA}$, with $(\rho_{rec|DAA}, \rho_{ex|DAA}, \rho_{non|DAA})$ assumed to follow the Dirichlet distribution, as described in detail in the previous subsection.

Then, since an estimate of $\pi_{ever}$ is available, $\pi_{ex}$ can be indirectly estimated since $\pi_{ever}$ is equal to a weighted average of $\pi_{rec}$ and $\pi_{ex}$, as shown by the following formula

$$\pi_{ever} = \frac{\rho_{rec}}{\rho_{ever}}\pi_{rec} + \frac{\rho_{ex}}{\rho_{ever}}\pi_{ex}, \quad (4)$$

or, equivalently,

$$\pi_{ex} = \left(\pi_{ever} - \frac{\rho_{rec}}{\rho_{ever}}\pi_{rec}\right) \times \frac{\rho_{ever}}{\rho_{ex}}. \quad (5)$$

## Prevalence of CHC among non-PWID

To estimate the CHC prevalence among non-PWID in a country (after 2010), the ECDC database (European Centre for Disease Prevention and Control (ECDC) 2021) was used. The ECDC group has carefully and critically collected information from relevant studies across the EU/EEA region in cooperation with the focal points of each country. On the basis of the representativeness of each study and other factors (European Centre for Disease Prevention and Control (ECDC) 2016), a risk of bias score was assigned to each study, with higher values

indicating higher-quality studies. When higher-quality studies are available in a country ($\geq 4$), they are used to estimate the overall CHC prevalence in that country among non-PWID. If there are no higher-quality studies, general population estimates with a lower quality (<4) are pooled. If no general population prevalence estimates are available, data on the prevalence of HCV in pregnant women could be used as a prevalence measure. Finally, if data on pregnant women are lacking too, first-time blood donor studies could be an option. However, if the national focal point agrees or recommends, estimates from a neighboring country could be used instead, or we could use any other relevant information provided/suggested by the national focal point.

We directly use CHC prevalence data from studies in the general population, if available. If the data in the general population refer to anti-HCV prevalence (without any data on the viremic population), we could adjust the estimates to get the CHC prevalence based on the spontaneous HCV clearance estimate of 26% and the number of individuals treated with DAAs in the general population, as previously described. However, since data in the general population were based on first-time blood donors, the CHC prevalence is likely to be underestimated. Thus, we did not perform a treatment adjustment in the general population as this would lead to CHC prevalence close to zero, which would be unreliable. In principle, we intend to remove individuals cured with DAAs from the general population only if a recent seroprevalence study is available.

As $\rho_{rec}$ and $\rho_{ex}$ are likely to be low, $\pi_{non}$ will be the most critical factor for the estimation of the overall CHC prevalence in the population. To adjust for potential heterogeneity between the CHC prevalence estimates from different studies in the non-PWID population in a country, as a sensitivity analysis, we also apply a random-effect meta-analytic approach to pool the CHC prevalence across studies on non-PWID (Lin and Chu 2020). The random-effect approach is carried out only when at least 3 studies are available in the general population of a certain country, and the estimates are compared with the corresponding estimates from the approach assuming no heterogeneity (similar to a fixed-effect meta-analysis). If the estimates are inconsistent, possible explanations are investigated and discussed. If there is substantial heterogeneity between studies, it would help more to explore the causes; for example, following national focal point suggestions, some studies could be excluded to reduce heterogeneity.

## Sensitivity analysis including migrants from endemic countries

A sensitivity analysis including data for the viremic population among migrants based on the report of the ECDC group (European Centre for Disease Prevention and Control 2016) was carried out. Migrants from endemic countries represent 3.9% of the adult population in Finland (Table 8 in European Centre for Disease Prevention and Control (2016)), with the respective CHC prevalence being equal to $\pi_{mig} = 1.9\%$ (Table 9 in European Centre for Disease Prevention and Control (2016)). The overall CHC prevalence is now estimated by

$$\pi = \pi_{rec}\rho_{rec} + \pi_{ex}\rho_{ex} + \pi_{mig}\rho_{mig} + \pi_{non}\rho_{non},$$

where $\rho_{rec} + \rho_{ex} + \rho_{mig} + \rho_{non} = 1$. Due to lack of data on the proportion of migrants among those treated with DAAs, a treatment adjustment was not made in this analysis.

# RESULTS

The above-mentioned approach was used to estimate the prevalence of CHC in 2019 (considering ages between 15-79 years: 4,332,975).

The aggregated data used by our approach are briefly presented in Table 1. The multi-state Markov model was calibrated on the estimated prevalence of recent PWID reported in the EMCDDA barometer. In Finland, there were 10 studies on first-time blood donors, which included only anti-HCV data.
To estimate the CHC prevalence in the general population (primarily non-PWID), we used a fixed-effect meta-analytic approach, with the corresponding results, when information on DAAs is ignored, presented in Table 2. However, based on data provided by the focal point, approximately 3,905 individuals were treated with DAAs from 2016 to 2019 in Finland.

The corresponding results accounting for the DAA uptake are presented in Table 3. The prevalence of recent and ex-PWID was low in Finland (about 0.35% and 0.8%, respectively) corresponding to 15,110 (95% CI: 14,300-15,850) recent PWID and 34,600 (95% CI: 33,320-35,730) ex-PWID in the population. However, the CHC prevalence in these groups was substantial (Table 3) being 54.13% and 47.47%, respectively. This translates to 8,178 (95% CI: 7,440-8,924) and 16,417 (95% CI: 13,742-19,019) recent and ex-PWID aged 15-79 living with CHC infection in Finland in 2019. The CHC prevalence in the general population was 0.02%

(95% CI: 0.02%-0.03%), much lower than that of the high-risk groups. Taking all pieces of information into account, the overall CHC prevalence in Finland in 2019 was equal to 0.59% (95% CI: 0.53%-0.66%), which corresponds to 25,650 (95% CI: 22,801-28,477) individuals aged 15-79 years with CHC infection. The corresponding results under a random-effect meta-analysis for the studies in the general population were very similar and are provided in Table 4.

The results from our model including migrants from endemic countries as a separate group are presented in Table 5. However, this analysis comes with possible limitations; that is, including migrants as a separate group is valid only if migrants do not overlap with the remaining groups (recent PWID, ex-PWID, and non-PWID). Thus, if migrants do participate proportionally in the study(ies) in the general population, this analysis may result in biased overall CHC estimates (most probably in higher CHC prevalence estimates).

## TABLES AND FIGURES

*Table 1. Data (real) contributing to the estimation of CHC prevalence in Finland in 2019.*

| Parameter | Estimate (95%CI) | Number (95%CI) | Numerator | Denominator | Notes | Year of study |
|---|---|---|---|---|---|---|
| $\rho_{rec}$ | | 15,110 (14,300-15,850) | | | Method based on McDonald et al. | 2019 |
| $\rho_{ex}$ | | 34,600 (33,320-35,730) | | | Method based on McDonald et al. | 2019 |
| $\pi(\text{anti-HCV})_{rec}$ | | | 436 | 589 | EMCDDA; (Fraser et al. 2018) | 2014 |
| $\pi(\text{anti-HCV})_{ever}$ | | | 143 | 203 | EMCDDA | 2019 |
| $\pi(\text{anti-HCV})_{non}$ | | | 2 | 19,427 | ECDC database (NA et al.); Risk of bias=NA | 2010 |
| $\pi(\text{anti-HCV})_{non}$ | | | 5 | 19,775 | ECDC database (NA et al.); Risk of bias=NA | 2011 |
| $\pi(\text{anti-HCV})_{non}$ | | | 10 | 15,759 | ECDC database | 2012 |

| Parameter | Estimate (95%CI) | Number (95%CI) | Numerator | Denominator | Notes | Year of study |
|---|---|---|---|---|---|---|
| | | | | | (NA et al.); Risk of bias=NA | |
| $\pi(\text{anti-HCV})_{non}$ | | | 7 | 14,221 | ECDC database (NA et al.); Risk of bias=NA | 2013 |
| $\pi(\text{anti-HCV})_{non}$ | | | 3 | 14,324 | ECDC database (NA et al.); Risk of bias=NA | 2014 |
| $\pi(\text{anti-HCV})_{non}$ | | | 5 | 13,622 | ECDC database (NA et al.); Risk of bias=NA | 2015 |
| $\pi(\text{anti-HCV})_{non}$ | | | 5 | 13,361 | ECDC database (FRC BS, Annual statistics et al.); Risk of bias=NA | 2016 |
| $\pi(\text{anti-HCV})_{non}$ | | | 4 | 14,354 | ECDC database (FRC BS, | 2017 |

| Parameter | Estimate (95%CI) | Number (95%CI) | Numerator | Denominator | Notes | Year of study |
|---|---|---|---|---|---|---|
| | | | | | Annual statistics et al.); Risk of bias=NA | |
| $\pi(\text{anti-HCV})_{non}$ | | | 3 | 14,746 | ECDC database (FRC BS, Annual statistics et al.); Risk of bias=NA | 2018 |
| $\pi(\text{anti-HCV})_{non}$ | | | 6 | 13,724 | ECDC database (FRC BS, Annual statistics et al.); Risk of bias=NA | 2019 |

*Notes*: Although it looks counter-intuitive, **a higher risk of bias score denotes a higher-quality study** (range from 0 to 6).

*Table 2. Results from the method assuming no heterogeneity in the estimation of the CHC prevalence in the general population from different studies (i.e., similar to a fixed-effect meta-analysis).*

| Parameter | Posterior median | Posterior sd | LB (2.5%) | UB (97.5%) |
|---|---|---|---|---|
| $\rho_{rec}$ (%) | 0.35 | 0.01 | 0.33 | 0.37 |
| $\rho_{ex}$ (%) | 0.8 | 0.01 | 0.77 | 0.83 |
| $\pi_{rec}$ (%) | 54.83 | 1.87 | 51.18 | 58.51 |
| $\pi_{ex}$ (%) | 50.87 | 3.64 | 43.64 | 58 |
| $\pi_{non}$ (%) | 0.02 | 0 | 0.02 | 0.03 |
| $\pi$ (%) | 0.62 | 0.03 | 0.56 | 0.69 |
| Number with CHC | 26,931 | 1,394 | 24,225 | 29,706 |
| Pr(Recent PWID\|CHC) (%) | 30.75 | 1.7 | 27.69 | 34.41 |
| Pr(Ex-PWID\|CHC) (%) | 65.35 | 1.87 | 61.35 | 68.72 |
| Pr(Non-PWID\|CHC) (%) | 3.89 | 0.55 | 2.92 | 5.08 |

*Notes*: Information on DAAs is not taken into account.

*Abbreviations*: $\rho_{rec}$, prevalence of recent PWID; $\rho_{ex}$, prevalence of ex-PWID; $\pi_{rec}$, CHC prevalence among recent PWID; $\pi_{ex}$, CHC prevalence among ex-PWID; $\pi_{non}$, CHC prevalence among non-PWID; $\pi$ overall CHC prevalence in Finland; LB, Lower Boundary; UP, Upper Boundary

*Table 3. Results from the method assuming no heterogeneity in the estimation of the CHC prevalence in the general population from different studies (i.e., similar to a fixed-effect meta-analysis).*

| Parameter | Posterior median | Posterior sd | LB (2.5%) | UB (97.5%) |
|---|---|---|---|---|
| $\rho_{rec}$ (%) | 0.35 | 0.01 | 0.33 | 0.37 |
| $\rho_{ex}$ (%) | 0.8 | 0.01 | 0.77 | 0.83 |
| $\pi_{rec}$ (%) | 54.13 | 2 | 50.17 | 58.01 |
| $\pi_{ex}$ (%) | 47.47 | 3.77 | 39.89 | 54.72 |
| $\pi_{non}$ (%) | 0.02 | 0 | 0.02 | 0.03 |
| $\pi$ (%) | 0.59 | 0.03 | 0.53 | 0.66 |
| Number with CHC | 25,650 | 1,436 | 22,801 | 28,477 |
| Pr(Recent PWID\|CHC) (%) | 31.88 | 1.9 | 28.48 | 36.01 |
| Pr(Ex-PWID\|CHC) (%) | 64.01 | 2.09 | 59.5 | 67.73 |
| Pr(Non-PWID\|CHC) (%) | 4.09 | 0.59 | 3.06 | 5.34 |

*Notes*: The number of individuals treated with DAAs is taken into account.

*Abbreviations*: $\rho_{rec}$, prevalence of recent PWID; $\rho_{ex}$, prevalence of ex-PWID; $\pi_{rec}$, CHC prevalence among recent PWID; $\pi_{ex}$, CHC prevalence among ex-PWID; $\pi_{non}$, CHC prevalence among non-PWID; $\pi$ overall CHC prevalence in Finland; LB, Lower Boundary; UP, Upper Boundary

*Table 4. Results from the method assuming heterogeneity in the estimation of the CHC prevalence in the general population from different studies (i.e., similar to a random-effect meta-analysis).*

| Parameter | Posterior median | Posterior sd | LB (2.5%) | UB (97.5%) |
|---|---|---|---|---|
| $\rho_{rec}$ (%) | 0.35 | 0.01 | 0.33 | 0.37 |
| $\rho_{ex}$ (%) | 0.8 | 0.01 | 0.77 | 0.83 |
| $\pi_{rec}$ (%) | 54.04 | 2.02 | 50.16 | 58.03 |
| $\pi_{ex}$ (%) | 47.45 | 3.75 | 39.84 | 54.57 |
| $\pi_{non}$ (%) | 0.02 | 0.01 | 0.01 | 0.03 |
| $\pi$ (%) | 0.59 | 0.03 | 0.52 | 0.65 |
| Number with CHC | 25,564 | 1,441 | 22,681 | 28,362 |
| Pr(Recent PWID\|CHC) (%) | 31.96 | 1.93 | 28.62 | 36.17 |
| Pr(Ex-PWID\|CHC) (%) | 64.16 | 2.13 | 59.52 | 67.93 |
| Pr(Non-PWID\|CHC) (%) | 3.84 | 0.86 | 2.33 | 5.73 |
| Between-study variance | 0.2 | 0.32 | 0.01 | 1.18 |

*Notes*: The number of individuals treated with DAAs is taken into account.

*Abbreviations*: $\rho_{rec}$, prevalence of recent PWID; $\rho_{ex}$, prevalence of ex-PWID; $\pi_{rec}$, CHC prevalence among recent PWID; $\pi_{ex}$, CHC prevalence among ex-PWID; $\pi_{non}$, CHC prevalence among non-PWID; $\pi$ overall CHC prevalence in Finland; LB, Lower Boundary; UP, Upper Boundary

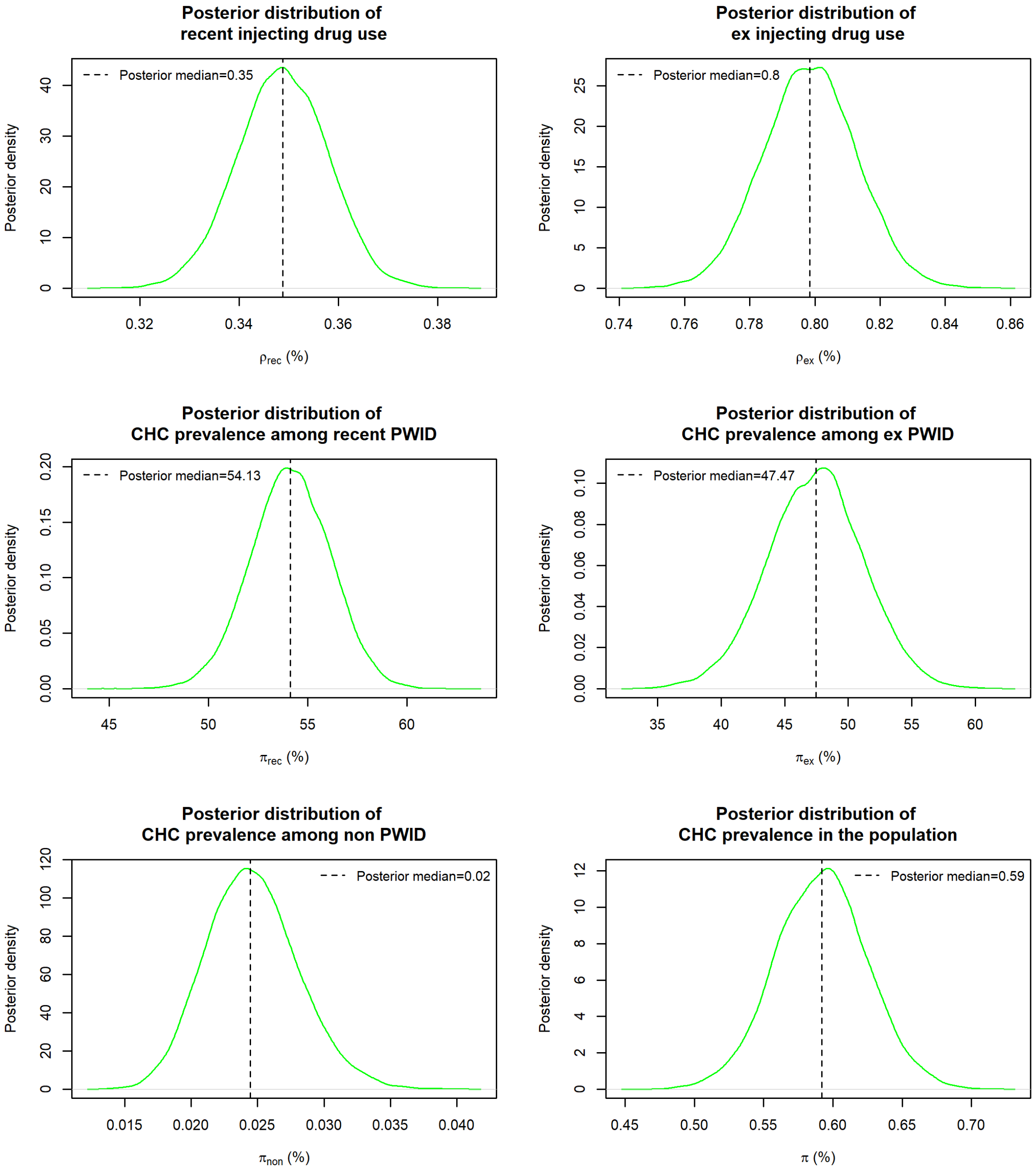


*Figure 1. Posterior distributions of the key model parameters using the approach that assumes no heterogeneity in the estimation of the CHC prevalence in the general population (primarily non-PWID) from different studies (i.e., similar to a fixed-effect meta-analysis), with the number of individuals treated with DAA taken into account.*

*Table 5. Results from the method assuming no heterogeneity in the estimation of the CHC prevalence in the general population (primarily non-PWID) from different studies (i.e., similar to a fixed-effect meta-analysis) including migrants from endemic countries as a separate group.*

| Parameter | Posterior median | Posterior sd | LB (2.5%) | UB (97.5%) |
|---|---|---|---|---|
| $\rho_{rec}$ (%) | 0.35 | 0.01 | 0.33 | 0.37 |
| $\rho_{ex}$ (%) | 0.8 | 0.01 | 0.77 | 0.83 |
| $\rho_{mig}$ (%) | 3.9 | 0 | 3.9 | 3.9 |
| $\pi_{rec}$ (%) | 54.82 | 1.89 | 51.13 | 58.56 |
| $\pi_{ex}$ (%) | 50.91 | 3.63 | 43.68 | 57.89 |
| $\pi_{mig}$ (%) | 1.9 | 0.45 | 1.01 | 2.77 |
| $\pi_{non}$ (%) | 0.02 | 0 | 0.02 | 0.03 |
| $\pi$ (%) | 0.7 | 0.04 | 0.62 | 0.77 |
| Number with CHC | 30,126 | 1,593 | 26,968 | 33,215 |
| Pr(Recent PWID\|CHC) (%) | 27.5 | 1.59 | 24.66 | 30.94 |
| Pr(Ex-PWID\|CHC) (%) | 58.46 | 2.41 | 53.52 | 63.04 |
| Pr(Mig\|CHC) (%) | 10.67 | 2.32 | 5.97 | 15.04 |
| Pr(Non-PWID\|CHC) (%) | 3.35 | 0.49 | 2.5 | 4.4 |

*Notes*: The number of individuals treated with DAAs is not taken into account.

*Abbreviations*: $\rho_{rec}$, prevalence of recent PWID; $\rho_{ex}$, prevalence of ex-PWID; $\rho_{mig}$, prevalence of migrants from endemic countries; $\pi_{rec}$, CHC prevalence among recent PWID; $\pi_{ex}$, CHC prevalence among ex-PWID; $\pi_{mig}$, CHC prevalence among migrants from endemic countries; $\pi_{non}$, CHC prevalence among non-PWID; $\pi$ overall CHC prevalence in Finland; LB, Lower Boundary; UP, Upper Boundary

# APPENDIX

## Fit of the multi-state Markov model

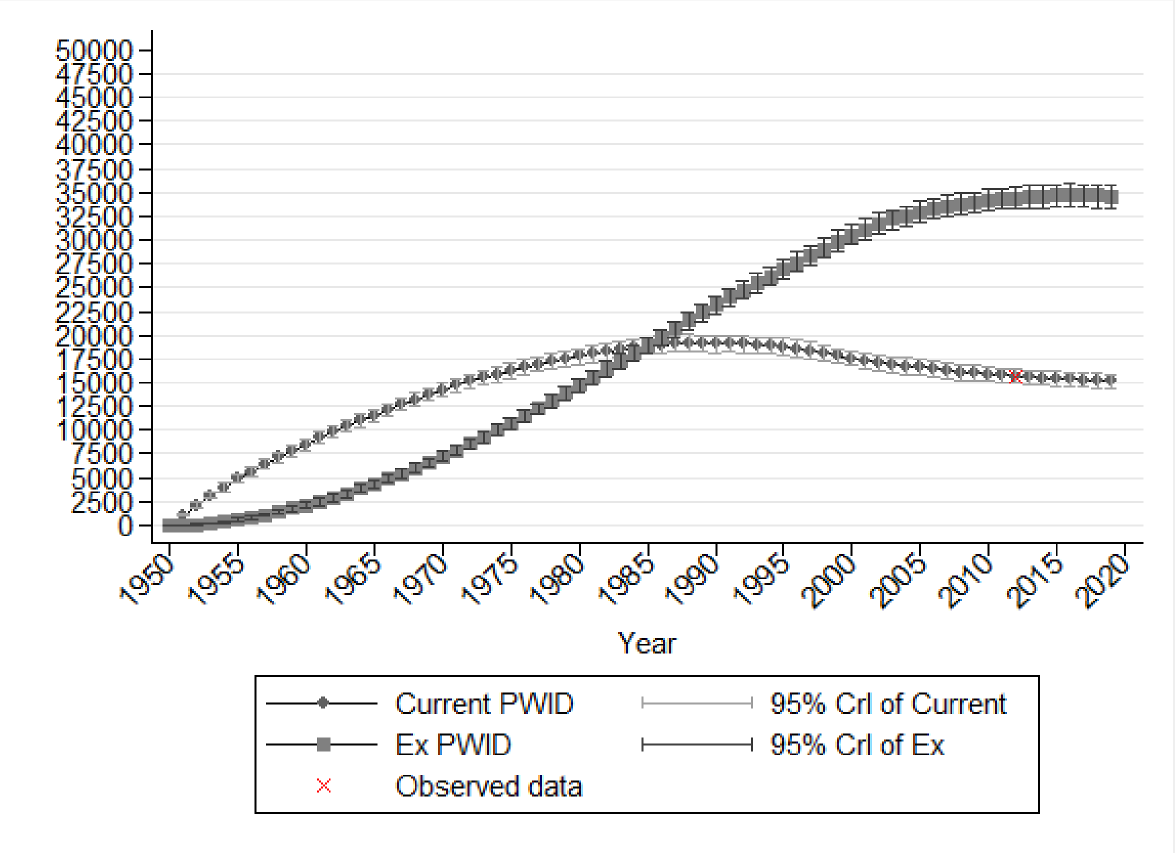


*Figure 2: Model predictions for the numbers of current and ex-PWID (people who inject drugs). The solid black line and shaded grey error bars show the median and 95% credible intervals (95% CrI) for the model projections. For comparison, asterisks indicate the observed country's data.*

## Stan code for Bayesian multiparameter evidence synthesis

```
data {
  int<lower=0> N1579; // Population of 15-79 in `Country`
  int<lower=1> Knon; // Number of studies in the ECDC data for `Country`
  int<lower=1> Kever; // Number of studies in the EMCDDA data for ever us
ers in `Country`
  int<lower=1> Kcur; // Number of studies in the EMCDDA barometer (for HC
V prevalence of PWID) in `Country`
  int<lower=1> NDAA; // Total number of DAAs from 2015 to 2019

  real p_cur_mean; // Prior mean for the prevalence of current use in `Co
untry`
  real<lower=0> p_cur_sd; // Prior sd for the prevalence of current use i
n `Country`

  real p_ex_mean; // Prior mean for the prevalence of ex-use in `Country`
  real<lower=0> p_ex_sd; // Prior sd for the prevalence of ex-use in `Cou
ntry`

  int<lower=0> Nst_hcv_cur[Kcur]; // Number of individuals in the study e
stimating anti-HCV among recent PWID
  int<lower=0> Yst_hcv_cur[Kcur]; // Number of HCV+ in the study estimati
ng anti-HCV among recent PWID

  int<lower=0> Nst_hcv_ever[Kever]; // Number of individuals in the study
estimating anti-HCV among ever IDU
  int<lower=0> Yst_hcv_ever[Kever]; // Number of HCV+ in the study estima
ting anti-HCV among ever PWID

  int<lower=0> Nst_hcv_non[Knon]; // Number of individuals in the study e
stimating anti-HCV among non PWID
  int<lower=0> Yst_hcv_non[Knon]; // Number of HCV+ in the study estimati
```

```
ng anti-HCV among non PWID

  vector<lower=0>[3] alpha; // parameter of the Diriclet prior
  vector<lower=0>[3] alphaDAA; // parameter of the Diriclet prior

  real HCVclear_mean; // Prior mean for the HCV clearance probability
  real<lower=0> HCVclear_sd; // Prior sd for the HCV clearance probabilit
y

  real SVR_mean; // Prior mean for the SVR among non-PWID
  real<lower=0> SVR_sd; // Prior sd for the SVR among non-PWID

  real SVR_PWID_mean; // Prior mean for the SVR among PWID
  real<lower=0> SVR_PWID_sd; // Prior sd for the SVR among PWID
}

// Block defining the original parameters
parameters {
  // The parameters to be sampled
  simplex[3] rho; // Prevalence of the three risk groups
  real<lower=0,upper=1> CHCpi_cur; // P(CHC+|Cur)
  real<lower=0,upper=1> CHCpi_ex; // P(CHC+|Ex)
  real<lower=0,upper=1> CHCpi_non; // P(CHC+|Non)
  real<lower=0,upper=1> SVR; // SVR of DAAs among non-PWID
  real<lower=0,upper=1> SVR_PWID; // SVR of DAAs among PWID
  simplex[3] rho_DAA; // Prevalence of the three risk groups among those
treated with DAAs

  real<lower=0,upper=1-CHCpi_cur> HCVclear; // Probability of HCV clearan
ce; upper bound = 1-prevalence of chronic HCV
}
```

```
transformed parameters {
  // Change scales
  real<lower=0,upper=1> rho_ever;
  real<lower=0,upper=1> CHCpi_ever;
  real<lower=0,upper=1> pi_ever;
  real<lower=0,upper=1> pi_non;
  real<lower=0,upper=1> pi_cur;

  rho_ever = rho[1] + rho[2];
  CHCpi_ever = CHCpi_cur*rho[1]/rho_ever + CHCpi_ex*rho[2]/rho_ever;
  pi_ever = CHCpi_ever/(1-HCVclear);
  pi_non = CHCpi_non/(1-HCVclear);
  pi_cur = CHCpi_cur/(1-HCVclear);
}


// Binomial regression model
model {
  // Priors
  rho ~ dirichlet(alpha);
  rho_DAA ~ dirichlet(alphaDAA);

  //////////////////////////////
  // Likelihood contributions //
  //////////////////////////////

  // Probability of HCV clearance
  HCVclear ~ normal(HCVclear_mean,HCVclear_sd);

  // Prevalence of current use
  rho[1] ~ normal(p_cur_mean,p_cur_sd);
```

```
  // Prevalence of ex-use
  rho[2] ~ normal(p_ex_mean,p_ex_sd);

  // Prevalence of chronic HCV among current users
  Yst_hcv_cur ~ binomial(Nst_hcv_cur,pi_cur);

  // Prevalence of HCV among ever users
  Yst_hcv_ever ~ binomial(Nst_hcv_ever,pi_ever);

  // HCV+ among non
  Yst_hcv_non ~ binomial(Nst_hcv_non,pi_non);

  // SVR of DAAs among non-PWID
  SVR ~ normal(SVR_mean,SVR_sd);

  // SVR of DAAs among PWID
  SVR_PWID ~ normal(SVR_PWID_mean,SVR_PWID_sd);
}


generated quantities {
  // Functions of parameters
  real<lower=0,upper=100> overalCHC;
  real logit_CHCpi_cur;
  real logit_CHCpi_ex;
  real logit_CHCpi_non;
  real logit_rho_cur;
  real logit_rho_ex;
  real logit_rho_non;
  real<lower=0,upper=1> pEverGivenCHC;
  real<lower=0,upper=1> pCurGivenCHC;
```

```
  real<lower=0,upper=1> pExGivenCHC;
  real<lower=0,upper=1> pNonGivenCHC;

  real logit_HCVclear;
  real<lower=0> NumberCHC;

  real<lower=0,upper=1> CHCDAApi_non; // P(CHC+|Non) adjusted for DAA
  real<lower=0,upper=1> CHCDAApi_cur; // P(CHC+|Cur) adjusted for DAA
  real<lower=0,upper=1> CHCDAApi_ex; // P(CHC+|Ex) adjusted for DAA
  real<lower=0,upper=1> CHCDAApi_ever; // P(CHC+|Ever) adjusted for DAA

  real<lower=0,upper=100> overalCHC_DAA;
  real pEverGivenCHC_DAA;
  real pCurGivenCHC_DAA;
  real pExGivenCHC_DAA;
  real pNonGivenCHC_DAA;
  real<lower=0> NumberCHC_DAA;

  // Overall HCV prevalence
  overalCHC = 100*(rho[1]*CHCpi_cur + rho[2]*CHCpi_ex + rho[3]*CHCpi_non)
;
  pEverGivenCHC = CHCpi_ever*rho_ever/(overalCHC/100);
  pCurGivenCHC = CHCpi_cur*rho[1]/(overalCHC/100);
  pExGivenCHC = CHCpi_ex*rho[2]/(overalCHC/100);
  pNonGivenCHC = CHCpi_non*(1-rho_ever)/(overalCHC/100);
  NumberCHC = round(overalCHC*N1579/100);

  CHCDAApi_non = ( N1579*rho[3]*CHCpi_non - NDAA*0*SVR)/(N1579*rho[3]);
  CHCDAApi_cur = ( N1579*rho[1]*CHCpi_cur - NDAA*rho_DAA[1]*SVR_PWID_mean
)/(N1579*rho[1]);
  CHCDAApi_ex = ( N1579*rho[2]*CHCpi_ex - NDAA*rho_DAA[2]*SVR_PWID_mean)/
(N1579*rho[2]);
```

```
  CHCDAApi_ever = CHCDAApi_cur*rho[1]/rho_ever + CHCDAApi_ex*rho[2]/rho_e
ver;

  overalCHC_DAA = 100*(rho[1]*CHCDAApi_cur + rho[2]*CHCDAApi_ex +  rho[3]
*CHCDAApi_non);
  pEverGivenCHC_DAA = CHCDAApi_ever*rho_ever/(overalCHC_DAA/100);
  pCurGivenCHC_DAA = CHCDAApi_cur*rho[1]/(overalCHC_DAA/100);
  pExGivenCHC_DAA = CHCDAApi_ex*rho[2]/(overalCHC_DAA/100);
  pNonGivenCHC_DAA = CHCDAApi_non*(1-rho_ever)/(overalCHC_DAA/100);
  NumberCHC_DAA = round(overalCHC_DAA*N1579/100);


  logit_rho_cur = logit(rho[1]);
  logit_rho_ex = logit(rho[2]);
  logit_rho_non = logit(rho[3]);
  logit_CHCpi_cur = logit(CHCpi_cur);
  logit_CHCpi_ex = logit(CHCpi_ex);
  logit_CHCpi_non = logit(CHCpi_non);
  logit_HCVclear = logit(HCVclear);
}
```

### Multi-state Markov model

```
#include <stdio.h>
#include <stdlib.h>
#include <string.h>
#include <math.h>
#include <time.h>

#define loops 100
int populationPerYearAndAge[2021][66];
double deathRatePerAge[66];
int totalPopulationPerYear[2021];

FILE *F_DeathRate=fopen("`Country`-deathRates.txt","r");
FILE *F_Population=fopen("`Country`-population.txt","r");
FILE *out;

double pa_start = 0.00012;                    // Setting Variable
double pa_step  = 0.000002;                   // Setting Variable
double pa_stop  = 0.000123;                   // Setting Variable

double pa;
double pg = 1.0/11.7;   // Assumed average active injecting career of  11
.7 years
double pk = 0.004*12.0;     // Relapse rate of 0.004/month

struct people{
    int age;
    int state;
};

char filename[100];
struct people person[50000000];
```

```
long int totalPersons=0;

void setPopulationAge()
{
    for( int j=0; j<71; j++){
        for( int i=0; i<51; i++){
            fscanf(F_Population,"%d",&populationPerYearAndAge[1950+j][15+
i]);
        }
    }
}

void setDeathRate()
{
    for( int i=0; i<51; i++){
        fscanf(F_DeathRate,"%lf",&deathRatePerAge[15+i*5]);
        for( int j=0; j<5; j++){
            deathRatePerAge[15+i*5+j] = deathRatePerAge[15+i*5];
        }
    }
}

void getTotalPopulationPerAge()
{
    for( int j=0; j<71; j++){
        for( int i=0; i<51; i++){
            totalPopulationPerYear[1950+j] += populationPerYearAndAge[195
0+j][15+i];
        }
    }
}
```

```
void printTotalPopulationPerAge()
{
    printf("---- - ----------\n");
    printf("Year - Population\n");
    printf("---- - ----------\n");
    for( int i=1950; i<2021; i++){
        printf("%d - %d\n",i,totalPopulationPerYear[i]);
    }
}

void printDeathRatePerAge()
{
    printf("--- - -----\n");
    printf("Age - Rate\n");
    printf("--- - -----\n");
    for( int i=15; i<65; i++){
        printf("%d - %lf\n",i,deathRatePerAge[i]);
    }
}

void printTotalPersonPerState( int year)
{
    int count[4]= {0,0,0,0};
    for( int i=0; i<totalPersons; i++){
        count[person[i].state]++;
    }
    //printf("%d\t%ld\t%ld\t%ld\t%ld\n",year,count[0],count[1],count[2],c
ount[3]);
    if(year==2014)
        printf("%d\t%ld\t%ld\t%ld\t%ld\t%lf\n",year,count[0],count[1],cou
nt[2],count[3],pa);
    if( year>2009){
```

```
        fprintf(out, "%d\t%ld\t%ld\t%ld\t%ld\n",year,count[0],count[1],co
unt[2],count[3]);
    }
}

void initializePopulation()
{
    int cnt=0;
    for( int j=15; j<65; j++){
        for( int i=0; i<populationPerYearAndAge[1950][j]*100; i++){
            person[cnt].age=j;
            person[cnt].state=0;
            cnt++;
        }
    }
    totalPersons = cnt;
}

bool checkRate(double rate)
{
    if (rate <0.001){
        double k = rand()/(1.00+RAND_MAX);
        if(k<0.001){
            rate = rate*1000;
        }
        else{
            return false;
        }
    }
    double k = rand()/(1.00+RAND_MAX);
    if ( k < rate )
        return true;
```

```
    else
        return false;
}

void changeStatusAndAge()
{
    for( int i=0; i<totalPersons; i++){
        // change state
        double k = rand()/(1.00+RAND_MAX);
        if( person[i].state==0){
            if ( checkRate(pa) &&  person[i].age<40){
                person[i].state=1;
            }
        }
        else if( person[i].state==1){
            if ( k < pg ){
                person[i].state=2;
            }
        }
        else if( person[i].state==2){
            if ( k < pk ){
                person[i].state=1;
            }
        }
        // After their 49th year, all active PWID are assumed to cease in
jecting
        if(person[i].age>49 && person[i].state==1){
            person[i].state=2;
        }
        // After their 64th year, remove
        if(person[i].age>64){
            person[i].state=3;
```

```
        }
        // death rate depending on age and state
        k = rand()/(1.00+RAND_MAX);
        if( person[i].state==0){
            if ( k < deathRatePerAge[person[i].age] ){
                person[i].state=3;
            }
        }
        else if( person[i].state==1){
            if ( k < deathRatePerAge[person[i].age] * 13.0 ){
                person[i].state=3;
            }
        }
        else if( person[i].state==2){
            if ( k < deathRatePerAge[person[i].age] ){
                person[i].state=3;
            }
        }
        // increase age one year
        person[i].age++;
    }
}

void addNewPersons(int year)
{
    // add new person [15 years old and non PWID]
    int cnt=totalPersons;
    for( int i=0; i<populationPerYearAndAge[year+1][15]*100; i++){
// 100 instead of 1000 for the population to reduce execution time
        person[cnt].age=15;
        person[cnt].state=0;
        cnt++;
```

```
    }
    // increase the total number of population
    totalPersons = cnt;
}
int main()
{
    srand(time(NULL));

    setPopulationAge();
    setDeathRate();

    getTotalPopulationPerAge();
    pa = pa_start;
    while ( pa < pa_stop){

        snprintf(filename, 100, "result_%lf.txt",pa);
        out=fopen(filename,"w");

        for( int iter=0; iter<loops ;iter++){
            initializePopulation();
            for( int year=1950; year<2020; year++){
                printTotalPersonPerState(year);
                changeStatusAndAge();
                addNewPersons(year);
            }
        }

        fclose(out);
        pa = pa + pa_step;
    }
```

```
}
```

# REFERENCES

European Centre for Disease Prevention and Control. 2016. "Epidemiological Assessment of Hepatitis b and c Among Migrants in the EU/EEA." https://www.ecdc.europa.eu/sites/default/files/media/en/publications/Publications/epidemiological-assessment-hepatitis-B-and-C-among-migrants-EU-EEA.pdf.

European Centre for Disease Prevention and Control (ECDC). 2016. "Systematic Review on Hepatitis b and c Prevalence in the EU/EEA." https://www.ecdc.europa.eu/sites/default/files/media/en/publications/Publications/systematic-review-hepatitis-B-C-prevalence.pdf.

———. 2021. "Hepatitis c Prevalence Database." https://www.ecdc.europa.eu/en/all-topics-z/hepatitis-c/tools/hepatitis-c-prevalence-database.

Fraser, Hannah, Natasha K. Martin, Henrikki Brummer-Korvenkontio, Patrizia Carrieri, Olav Dalgard, John Dillon, David Goldberg, et al. 2018. "Model Projections on the Impact of HCV Treatment in the Prevention of HCV Transmission Among People Who Inject Drugs in Europe." *Journal of Hepatology* 68 (3): 402–11. https://doi.org/10.1016/j.jhep.2017.10.010.

Graf, Christiana, Marcus M Mücke, Georg Dultz, Kai-Henrik Peiffer, Alica Kubesch, Patrick Ingiliz, Stefan Zeuzem, Eva Herrmann, and Johannes Vermehren. 2019. "Efficacy of Direct-acting Antivirals for Chronic Hepatitis C Virus Infection in People Who Inject Drugs or Receive Opioid Substitution Therapy: A Systematic Review and Meta-analysis." *Clinical Infectious Diseases* 70 (11): 2355–65. https://doi.org/10.1093/cid/ciz696.

Grebely, Jason, Sarah Larney, Amy Peacock, Samantha Colledge, Janni Leung, Matthew Hickman, Peter Vickerman, et al. 2019. "Global, Regional, and Country-Level Estimates of Hepatitis c Infection Among People Who Have Recently Injected Drugs." *Addiction* 114 (1): 150–66. https://doi.org/https://doi.org/10.1111/add.14393.

Hines, Lindsey A., Adam Trickey, Janni Leung, Sarah Larney, Amy Peacock, Louisa Degenhardt, Samantha Colledge, et al. 2020. "Associations Between National Development Indicators and the Age Profile of People Who Inject Drugs: Results from a Global Systematic Review and Meta-

Analysis." *The Lancet Global Health* 8 (1): e76–91. https://doi.org/10.1016/S2214-109X(19)30462-0.

Lin, Lifeng, and Haitao Chu. 2020. "Meta-Analysis of Proportions Using Generalized Linear Mixed Models." *Epidemiology* 31 (5). https://journals.lww.com/epidem/Fulltext/2020/09000/Meta_analysis_of_Proportions_Using_Generalized.16.aspx.

McDonald, Scott A., Rosmawati Mohamed, Maznah Dahlui, Herlianna Naning, and Adeeba Kamarulzaman. 2014. "Bridging the Data Gaps in the Epidemiology of Hepatitis c Virus Infection in Malaysia Using Multi-Parameter Evidence Synthesis." *BMC Infectious Diseases* 14 (1): 564. https://doi.org/10.1186/s12879-014-0564-6.

Micallef, J. M., J. M. Kaldor, and G. J. Dore. 2006. "Spontaneous Viral Clearance Following Acute Hepatitis c Infection: A Systematic Review of Longitudinal Studies." *Journal of Viral Hepatitis* 13 (1): 34–41. https://doi.org/https://doi.org/10.1111/j.1365-2893.2005.00651.x.

Presanis, Anne M., O. Noel Gill, Timothy R. Chadborn, Caterina Hill, Vivian Hope, Louise Logan, Brian D. Rice, Valerie C. Delpech, A. E. Ades, and Daniela De Angelis. 2010. "Insights into the Rise in HIV Infections, 2001 to 2008: A Bayesian Synthesis of Prevalence Evidence." *AIDS* 24 (18). https://journals.lww.com/aidsonline/Fulltext/2010/11270/Insights_into_the_rise_in_HIV_infections,_2001_to.12.aspx.

Sweeting, M. J., D. De Angelis, M. Hickman, and A. E. Ades. 2008. "Estimating hepatitis C prevalence in England and Wales by synthesizing evidence from multiple data sources. Assessing data conflict and model fit." *Biostatistics* 9 (4): 715–34. https://doi.org/10.1093/biostatistics/kxn004.

Tan, Sarah, Susanna Makela, Daliah Heller, Kevin Konty, Sharon Balter, Tian Zheng, and James H. Stark. 2018. "A Bayesian Evidence Synthesis Approach to Estimate Disease Prevalence in Hard-to-Reach Populations: Hepatitis c in New York City." *Epidemics* 23: 96–109. https://doi.org/https://doi.org/10.1016/j.epidem.2018.01.002.

Thein, Hla-Hla, Qilong Yi, Gregory J. Dore, and Murray D. Krahn. 2008. "Estimation of Stage-Specific Fibrosis Progression Rates in Chronic Hepatitis c Virus Infection: A Meta-Analysis and

Meta-Regression." *Hepatology* 48 (2): 418–31. https://doi.org/https://doi.org/10.1002/hep.22375.

Veen, Maaike G. van, Anne M. Presanis, Stefano Conti, Maria Xiridou, Annemarie R. Stengaard, Martin C. Donoghoe, Ard I. van Sighem, Marianne A. van der Sande, and Daniela De Angelis. 2011. "National Estimate of HIV Prevalence in the Netherlands: Comparison and Applicability of Different Estimation Tools." *AIDS* 25 (2). https://journals.lww.com/aidsonline/Fulltext/2011/01140/National_estimate_of_HIV_prevalence_in_the.14.aspx.

# Estimating the prevalence of chronic hepatitis C infection (CHC) in France using Bayesian multiparameter evidence synthesis

10/05/2023

## Table of Contents

Introduction ........ 2

Methods ........ 2

Prevalence of ex-PWID ........ 3

Prevalence of recent PWID ........ 3

Prevalence of CHC among recent PWID ........ 4

Prevalence of CHC among ex-PWID ........ 4

Prevalence of CHC among non-PWID ........ 5

Sensitivity analysis for the injection status among individuals treated with DAAs ........ 6

Results ........ 7

Limitations ........ 8

Tables and Figures ........ 9

Appendix ........ 14

Fit of the multi-state Markov model ........ 14

Stan code for Bayesian multiparameter evidence synthesis ........ 15

Multi-state Markov model ........ 21

References ........ 29

## INTRODUCTION

Patients with chronic hepatitis C (HCV) infection often remain asymptomatic for decades and even for life, but a proportion of them develop active chronic hepatitis, which is a progressive disease (Thein et al. 2008). Because of the largely asymptomatic nature of HCV infection, notification data reflect national screening and testing practices and do not give accurate insights into the prevalence of HCV infection. Although there are studies that have estimated the prevalence of HCV antibodies (anti-HCV) in specific groups such as the general population, first-time blood donors, or people who inject drugs (PWID) at drug treatment centers, those alone cannot be combined to produce national estimates unless some additional information regarding the composition of each HCV risk group and its prevalence in the population are known (Sweeting et al. 2008; McDonald et al. 2014; Tan et al. 2018).

## METHODS

Bayesian multiparameter evidence synthesis (MPES) has been a popular approach to formally estimate anti-HCV or HIV prevalence (Sweeting et al. 2008; McDonald et al. 2014; Tan et al. 2018; Presanis et al. 2010; Veen et al. 2011). In this project, we extend this method to estimating the CHC prevalence, with the population (15-79 years) split into three main non-overlapping risk groups, i.e., recent PWID (those who have injected in the last year), ex-PWID, and non-PWID. A unified model is assumed including parameters associated with the CHC prevalence of recent, ex-PWID, and non-PWID (denoted by $\pi_{rec}$, $\pi_{ex}$, and $\pi_{non}$, respectively) and the prevalence of these risk groups in the population (denoted by $\rho_{rec}$, $\rho_{ex}$, and $\rho_{non}$, respectively; $\rho_{rec} + \rho_{ex} + \rho_{non} = 1$). To estimate the CHC prevalence in the whole population, $\pi$, we used the formula:

$$\pi = \pi_{rec}\rho_{rec} + \pi_{ex}\rho_{ex} + \pi_{non}\rho_{non} \quad (1)$$

Bayesian synthesis considers all available data, allowing for an evidence-based estimation of CHC prevalence in the population, with inherent uncertainty properly accounted for. Our aim is to estimate the CHC prevalence for the total (15-79) population of mainland France in 2019 using sources of information over the last decade (2010-2019). The population size of mainland France was obtained from National Institute of statistics and economic studies (INSEE).

## Prevalence of ex-PWID

Data on the prevalence of ex-PWID are sparse and generally often unreliable. To overcome this limitation, we apply the method proposed by McDonald et al. (2014), which requires simulating a multi-state Markov model representing the non-PWID, recent PWID, and ex-PWID. In the multi-state Markov model, the simulation starts in 1950 and examines the population aged 15-79 years old. A certain number of 15-year-old individuals enter the simulation process each year. These numbers of 15-year-old individuals are obtained from the United Nations Statistic Division UNSD, being different for each country. The ageing of the population is also taken into account. Country-specific overall mortality data are retrieved from the Life Tables of the World Health Organization WHO. The duration of injecting career is also country-specific and obtained from the paper of Hines et al. (2020). However, since no data for mainland France are reported in Hines et al. (2020), we used the duration of injecting career from the study of Cousien et al. (2016).

To estimate the number of ex-PWID, the model can be calibrated on the number of recent PWID provided in the EMCDDA barometer or the EMCDDA statistical bulletin (the code of the Markov model can be found in the Appendix). If there are no available data in the barometer, estimates from the systematic review of Grebely et al. (2019) could be used. Currently, as suggested by the national focal point, the multi-state Markov model was calibrated on the number of recent PWID reported in the report by the French Observatory for Drugs and Drug Addiction (OFDT).

After applying the model for mainland France, we compute the number (and the corresponding Confidence Interval - CI) of ex-PWID in 2019. The number of ex-PWID is then divided by the population size (15-79 years) in the same year, and a Normal distribution, constrained such that $\rho_{ex} \in (0,1)$, is assumed for $\rho_{ex}$. The standard deviation of this distribution is specified to approximately correspond to the respective CI, i.e. $\left(UB_{\rho_{ex}} - LB_{\rho_{ex}}\right)/(2 \times 1.96)$, where $UB_{\rho_{ex}}$ and $LB_{\rho_{ex}}$ denote the upper and lower limit of the CI.

## Prevalence of recent PWID

The number of recent PWID in mainland France in 2019 is also projected by the multi-state Markov model described in the previous subsection. To estimate $\rho_{rec}$, the number of recent

PWID is divided by the population size (15-79 years). Similar to the previous subsection, a Normal distribution, constrained such that $\rho_{rec} \in (0,1)$, is assumed for $\rho_{rec}$, with the standard deviation specified to correspond to the CI obtained from the multi-state Markov model.

## Prevalence of CHC among recent PWID

The prevalence of CHC among recent PWID was informed by CHC prevalence data from the cross-sectional ANRS-Coquelicot survey (Weill-Barillet et al. 2016) conducted for the second time in 2011, with the CHC point estimate obtained from the country feedback document returned to ECDC by the focal point. The Binomial distribution was used in the model. However, some people may have been treated with direct-acting antivirals (DAAs), with the sustained virologic response ($SVR_{PWID}$) among PWID estimated to be 88% (95% CI: 80% to 93%) (Graf et al. 2019). Based on the information provided in the paper of Pol et al. (2022) and Dessauce et al. (2019), as suggested by the national focal point, the number of individuals treated with DAAs from 2014 to 2019 is equal to 82,966. However, the proportions of each risk group among those treated with DAAs, i.e. $\rho_{rec|DAA}$, $\rho_{ex|DAA}$, and $\rho_{non|DAA}$ ($\rho_{rec|DAA} + \rho_{ex|DAA} + \rho_{non|DAA} = 1$), are not currently available in France. In this report, we make the assumption that $\rho_{rec|DAA}$ is equal to the proportion of recent PWID among CHC-positive individuals, i.e. $\Pr(\text{Recent PWID}|\text{CHC})$, as estimated by our model when the DAA uptake is ignored (Table 2). Similarly, we assume that $\rho_{ex|DAA} = \Pr(\text{Ex-PWID}|\text{CHC})$ and $\rho_{non|DAA} = \Pr(\text{Non-PWID}|\text{CHC})$. Thus, the CHC prevalence among recent PWID, adjusted for DAAs, can be estimated by

$$\pi_{rec} = \frac{N_{15,79}\rho_{rec}\tilde{\pi}_{rec} - N_{DAA}\rho_{rec|DAA}SVR_{PWID}}{N_{15,79}\rho_{rec}}, \quad (2)$$

where $\tilde{\pi}_{rec}$ denotes the CHC estimate derived solely by CHC data on recent PWID (injection in last month; Weill-Barillet et al. (2016) and country feedback document returned to ECDC by the focal point).

## Prevalence of CHC among ex-PWID

Information on CHC prevalence among ex-PWID (i.e., $\pi_{ex}$) is difficult to obtain directly. To overcome this, we used CHC prevalence data among ever PWID from the cross-sectional

ANRS-Coquelicot survey (Weill-Barillet et al. 2016) conducted for the second time in 2011, with the CHC point estimate obtained from the country feedback document returned to ECDC by the focal point. Thus, as an estimate of $\pi_{ever}$ is available, $\pi_{ex}$ can be indirectly estimated since $\pi_{ever}$ is equal to a weighted average of $\pi_{rec}$ and $\pi_{ex}$, with the mixing proportions informed by Weill-Barillet et al. (2016),

$$\pi_{ever} = 0.76\pi_{rec} + (1 - 0.76)\pi_{ex}, \quad (3)$$

or, equivalently,

$$\pi_{ex} = (\pi_{ever} - 0.76\pi_{rec}) \times \frac{1}{1 - 0.76}, \quad (4)$$

However, some ex-PWID may have been treated with DAAs. Similarly to the procedure described in the previous subsection, the CHC prevalence among ex-PWID, adjusted for DAAs, can be estimated by

$$\pi_{ex} = \frac{N_{15,79}\rho_{ex}\tilde{\pi}_{ex} - N_{DAA}\rho_{ex|DAA}SVR_{PWID}}{N_{15,79}\rho_{ex}}. \quad (5)$$

where $\tilde{\pi}_{ex}$ denotes the CHC estimate among ex-PWID ignoring information on DAAs. Recall that it is assumed that $\rho_{ex|DAA}$ is assumed to be equal to Pr(Ex-PWID|CHC), as estimated by our model ignoring the effect of DAAs (Table 2).

## Prevalence of CHC among non-PWID

To estimate the CHC prevalence among non-PWID in a country (after 2010), the ECDC database (European Centre for Disease Prevention and Control (ECDC) 2021) was used. The ECDC group has carefully and critically collected information from relevant studies across the EU/EEA region in cooperation with the focal points of each country. On the basis of the representativeness of each study and other factors (European Centre for Disease Prevention and Control (ECDC) 2016), a risk of bias score was assigned to each study, with higher values indicating higher-quality studies. When higher-quality studies are available in a country ($\geq 4$), they are used to estimate the overall CHC prevalence in that country among non-PWID. If there are no higher-quality studies, general population estimates with a lower quality (<4) are pooled. If no general population prevalence estimates are available, data on the prevalence of

HCV in pregnant women could be used as a prevalence measure. Finally, if data on pregnant women are lacking too, first-time blood donor studies could be an option. However, if the national focal point agrees or recommends, estimates from a neighboring country could be used instead, or we could use any other relevant information provided/suggested by the national focal point.

In France, there was 1 study on non-PWID of high quality in 2016, which included CHC data (Brouard et al. 2019). Thus, individuals treated with DAAs should be removed, with the sustained virologic response ($SVR$) in the general population estimated to be 96.7% (95% CI: 95.4% to 98.1%) (Lampertico et al. 2020). Similarly to the procedure described in the previous subsections, the CHC prevalence among non-PWID, adjusted for DAAs, can be estimated by

$$\pi_{non} = \frac{N_{15,79}\rho_{non}\tilde{\pi}_{non} - \frac{4}{6}N_{DAA}\rho_{non|DAA}SVR}{N_{15,79}\rho_{non}}. \quad (6)$$

where $\tilde{\pi}_{non}$ denotes the CHC prevalence estimate based solely on Brouard et al. (2019), after excluding individuals reporting an injection history. Note also that we have multiplied the number of DAAs by $\frac{4}{6}$ as the study of Brouard et al. (2019) was performed in the first semester of 2016 and DAAs were available from 2014.

## Sensitivity analysis for the injection status among individuals treated with DAAs

Since the injection status among those treated with DAAs is unknown, we carried out a sensitivity analysis. Specifically, using information from Pol et al. (2022), the proportion of ever PWID among those treated with DAAs is assumed to be 21%, although, as mentioned by the focal point, the proportion of recent or ex-PWID having received treatment may be higher. Among ever PWID, we also assume that treatment is distributed according to the estimated number of recent and ex-PWID in the population. That is, we assume that the proportion of each risk group among those treated with DAAs are $\rho_{rec|DAA} = 0.21\frac{\rho_{rec}}{\rho_{rec}+\rho_{ex}}$, $\rho_{ex|DAA} = 0.21\frac{\rho_{ex}}{\rho_{rec}+\rho_{ex}}$, and $\rho_{non|DAA} = (1-0.21)$ $(\rho_{rec|DAA} + \rho_{ex|DAA} + \rho_{non|DAA} = 1)$. The corresponding results are provided in Table 4.

# RESULTS

The above-mentioned approach was used to estimate the prevalence of CHC in 2019 (considering ages between 15-79 years: 49,606,968).

The aggregated data used by our approach are briefly presented in Table 1. The multi-state Markov model was calibrated on the number of recent PWID reported in the report by the French Observatory for Drugs and Drug Addiction (OFDT). In France, there was 1 study of high quality, which included CHC data (Brouard et al. 2019). To estimate the CHC prevalence in the general population (primarily non-PWID), we used a fixed-effect meta-analytic approach, with the corresponding results, when information on DAAs is ignored, presented in Table 2. However, approximately 82,966 individuals were treated with DAAs from 2014 to 2019 in France, with the proportion of recent PWID, ex-PWID, and non-PWID among the 82,966 treated individuals assumed to be equal to Pr(Recent PWID|CHC) ≈ 18.83% Pr(Ex-PWID|CHC) ≈ 18.72% and Pr(Non-PWID|CHC) ≈ 62.17%, respectively, with the uncertainty in these estimates taken into account (Table 2).

The corresponding results, accounting for the DAA uptake, are presented in Table 3. The prevalence of recent and ex-PWID was low in France (about 0.22% and 0.51%, respectively) corresponding to 109,950 (95% CI: 107,435-112,300) recent PWID and 254,510 (95% CI: 250,615-257,695) ex-PWID in the population. However, the CHC prevalence in these groups was substantial (Table 3) being 22.12% and 9.33%, respectively. This translates to 24,324 (95% CI: 18,660-29,049) and 23,730 (95% CI: 1,645-63,252) recent and ex-PWID aged 15-79 living with CHC infection in France in 2019. The CHC prevalence in the general population was 0.19% (95% CI: 0.1%-0.32%), much lower than that of the high-risk groups. Taking all pieces of information into account, the overall CHC prevalence in France in 2019 was equal to 0.29% (95% CI: 0.16%-0.46%), which corresponds to 142,921 (95% CI: 77,226-227,201) individuals aged 15-79 years with CHC infection.

The results when the injection status among those treated with DAAs is based on Pol et al. (2022) are presented in Table 4. The total CHC-positive population is similar to that reported in Table 3. However, the CHC prevalence estimate among recent PWID is substantially higher than the corresponding estimate in Table 3.

## Limitations

The analyses reported in this document have certain limitations. First, although the total number of individuals treated with DAAs was adequately estimated, the proportion of the three risk groups (recent, ex, and non-PWID) among those treated was not available. In this report, we assume that these proportions are equal to the corresponding proportions of the three risk groups among CHC-positive individuals, as estimated by our model when information on DAAs is ignored. Although this assumption may not be entirely correct, our resulting CHC estimate among ever PWID was similar to the estimate reported in this publication in 2019, a study which was mentioned by the national focal point. As a sensitivity analysis, based on the information available in Pol et al. (2022), we assumed that the proportion of ever PWID among individuals treated with DAAs is equal to 21%. However, this has to be interpreted as a minimum estimate because people who received OST from hospital pharmacies or harm reduction centers cannot be identified in the French administrative healthcare databases, on which data in Pol et al. (2022) were based. Thus, the CHC estimates among PWID from this sensitivity analysis (Table 4) have to be interpreted as a maximum.

Moreover, the estimates for the prevalence of CHC in recent and ex-PWID are based on the Coquelicot survey conducted in 2011. Between 2011 and 2014, though, some of these individuals may also have been cured thanks to IFN/Peg-RBV treatment. Another potential limitation is that the reinfection risk (mostly among PWIDs) was not considered.

# TABLES AND FIGURES

*Table 1. Data (real) contributing to the estimation of CHC prevalence in France in 2019.*

| Parameter | Number (95%CI) | Numerator | Denominator | Notes | Year of study |
|---|---|---|---|---|---|
| $\rho_{rec}$ | 109,950 (107,435-112,300) | | | Method based on McDonald et al. | 2019 |
| $\rho_{ex}$ | 254,510 (250,615-257,695) | | | Method based on McDonald et al. | 2019 |
| $\pi_{rec}$ | | 105 | 298 | (Weill-Barillet et al. 2016)† | 2011 |
| $\pi_{ever}$ | | 271 | 903 | (Weill-Barillet et al. 2016)† | 2011 |
| $\pi_{non}$ | | 16 | 6,536 | ECDC database (Brouard et al.); Risk of bias=6†† | 2016 |

*Notes*: Although it looks counter-intuitive, **a higher risk of bias score denotes a higher-quality study** (range from 0 to 6); † Denominator based on information available in Weill-Barillet et al. (2016) and numerator based also on the CHC estimates reported in the country feedback document; †† After excluding PWID from the study.

*Table 2. Results from the method assuming no heterogeneity in the estimation of the CHC prevalence in the general population from different studies (i.e., similar to a fixed-effect meta-analysis).*

| Parameter | Posterior median | Posterior sd | LB (2.5%) | UB (97.5%) |
|---|---|---|---|---|
| $\rho_{rec}$ (%) | 0.22 | 0 | 0.22 | 0.23 |
| $\rho_{ex}$ (%) | 0.51 | 0 | 0.51 | 0.52 |
| $\pi_{rec}$ (%) | 34.91 | 2.44 | 29.89 | 39.38 |
| $\pi_{ex}$ (%) | 14.95 | 9.01 | 1.19 | 34.84 |
| $\pi_{ever}$ (%) | 21.01 | 5.76 | 12.17 | 33.77 |
| $\pi_{non}$ (%) | 0.26 | 0.06 | 0.15 | 0.39 |
| $\pi$ (%) | 0.41 | 0.08 | 0.28 | 0.58 |
| Number with CHC | 203,556 | 37,487 | 139,554 | 285,418 |
| Pr(Recent PWID\|CHC) (%) | 18.83 | 4.34 | 12.34 | 29.1 |
| Pr(Ex-PWID\|CHC) (%) | 18.72 | 9.57 | 1.74 | 37.7 |
| Pr(Non-PWID\|CHC) (%) | 62.17 | 8.34 | 44.68 | 76.98 |

*Notes*: Information on DAAs is not taken into account.

*Abbreviations*: $\rho_{rec}$, prevalence of recent PWID (proportion of the population that is recent PWID); $\rho_{ex}$, prevalence of ex-PWID (proportion of the population that is ex-PWID); $\pi_{rec}$, CHC prevalence among recent PWID; $\pi_{ex}$, CHC prevalence among ex-PWID; $\pi_{non}$, CHC prevalence among non-PWID; $\pi$ overall CHC prevalence in France; LB, Lower Boundary; UP, Upper Boundary

*Table 3. Results from the method assuming no heterogeneity in the estimation of the CHC prevalence in the general population from different studies (i.e., similar to a fixed-effect meta-analysis).*

| Parameter | Posterior median | Posterior sd | LB (2.5%) | UB (97.5%) |
|---|---|---|---|---|
| $\rho_{rec}$ (%) | 0.22 | 0 | 0.22 | 0.23 |
| $\rho_{ex}$ (%) | 0.51 | 0 | 0.51 | 0.52 |
| $\pi_{rec}$ (%) | 22.12 | 2.37 | 16.99 | 26.37 |
| $\pi_{ex}$ (%) | 9.33 | 6.5 | 0.65 | 24.82 |
| $\pi_{ever}$ (%) | 13.24 | 4.7 | 6.46 | 24.08 |
| $\pi_{non}$ (%) | 0.19 | 0.06 | 0.1 | 0.32 |
| $\pi$ (%) | 0.29 | 0.08 | 0.16 | 0.46 |
| Number with CHC | 142,921 | 38,173 | 77,226 | 227,201 |
| Pr(Recent PWID\|CHC) (%) | 17.23 | 3.43 | 1.56 | 35.39 |
| Pr(Ex-PWID\|CHC) (%) | 16.84 | 9.07 | 1.56 | 35.39 |
| Pr(Non-PWID\|CHC) (%) | 65.72 | 8.01 | 48.68 | 79.16 |

*Notes*: The number of individuals treated with DAAs is taken into account. The proportion of the three risk groups (recent, ex, and non-PWID) among those treated with DAAs was assumed to be equal to the corresponding one among CHC-positive.

*Abbreviations*: $\rho_{rec}$, prevalence of recent PWID (proportion of the population that is recent PWID); $\rho_{ex}$, prevalence of ex-PWID (proportion of the population that is ex-PWID); $\pi_{rec}$, CHC prevalence among recent PWID; $\pi_{ex}$, CHC prevalence among ex-PWID; $\pi_{non}$, CHC prevalence among non-PWID; $\pi$ overall CHC prevalence in France; LB, Lower Boundary; UP, Upper Boundary

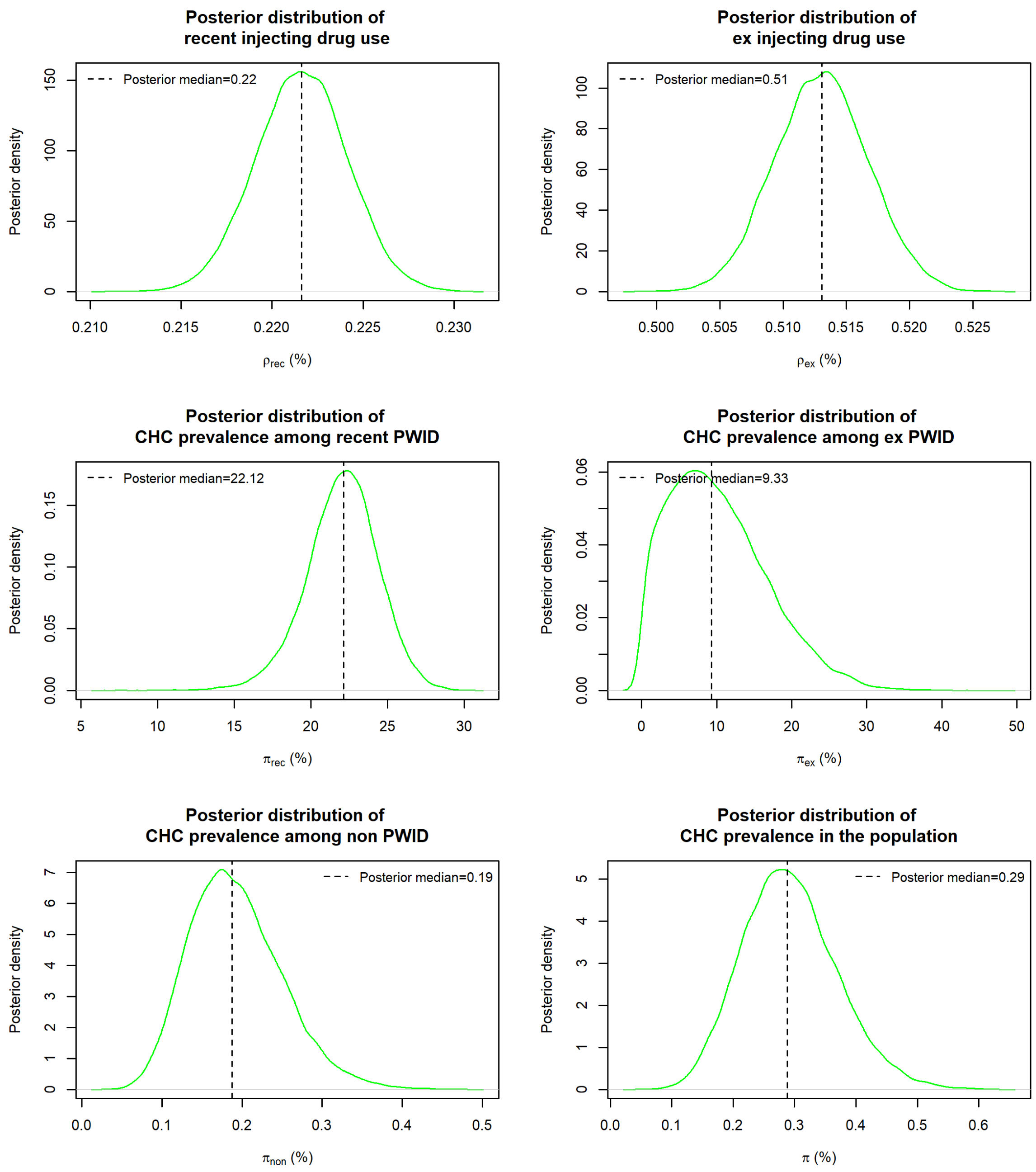


*Figure 1. Posterior distributions of the key model parameters using the approach that assumes no heterogeneity in the estimation of the CHC prevalence in the general population (primarily non-PWID) from different studies (i.e., similar to a fixed-effect meta-analysis), with the number of individuals treated with DAA taken into account.*

*Table 4. Results from the method assuming no heterogeneity in the estimation of the CHC prevalence in the general population from different studies (i.e., similar to a fixed-effect meta-analysis).*

| Parameter | Posterior median | Posterior sd | LB (2.5%) | UB (97.5%) |
|---|---|---|---|---|
| $\rho_{rec}$ (%) | 0.22 | 0 | 0.22 | 0.23 |
| $\rho_{ex}$ (%) | 0.51 | 0 | 0.51 | 0.52 |
| $\pi_{rec}$ (%) | 30.74 | 2.44 | 25.72 | 35.2 |
| $\pi_{ex}$ (%) | 10.51 | 9.03 | 0 | 30.67 |
| $\pi_{ever}$ (%) | 16.65 | 5.78 | 8 | 29.56 |
| $\pi_{non}$ (%) | 0.17 | 0.06 | 0.07 | 0.31 |
| $\pi$ (%) | 0.29 | 0.08 | 0.16 | 0.46 |
| Number with CHC | 146,252 | 37,920 | 81,146 | 229,976 |
| Pr(Recent PWID\|CHC) (%) | 23.07 | 8.1 | 0 | 44.82 |
| Pr(Ex-PWID\|CHC) (%) | 18.38 | 13.72 | -6.85 | 44.82 |
| Pr(Non-PWID\|CHC) (%) | 57.79 | 12.17 | 31.67 | 78.16 |

*Notes*: The number of individuals treated with DAAs is taken into account. The proportion of the three risk groups (recent, ex, and non-PWID) among those treated with DAAs was based on information reported in Pol et al. (2022).

*Abbreviations*: $\rho_{rec}$, prevalence of recent PWID (proportion of the population that is recent PWID); $\rho_{ex}$, prevalence of ex-PWID (proportion of the population that is ex-PWID); $\pi_{rec}$, CHC prevalence among recent PWID; $\pi_{ex}$, CHC prevalence among ex-PWID; $\pi_{non}$, CHC prevalence among non-PWID; $\pi$ overall CHC prevalence in France; LB, Lower Boundary; UP, Upper Boundary

# APPENDIX

## Fit of the multi-state Markov model

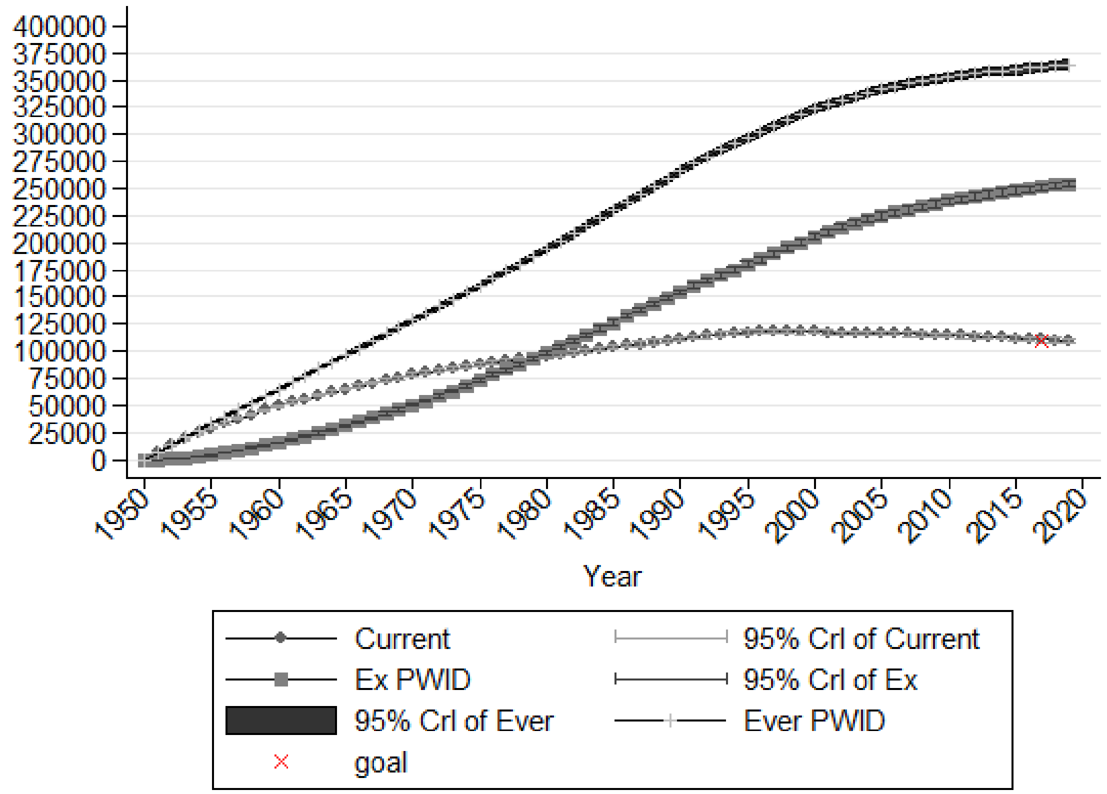


*Figure 2: Model predictions for the numbers of current and ex-PWID (people who inject drugs). The solid black line and shaded grey error bars show the median and 95% credible intervals (95% CrI) for the model projections. For comparison, asterisks indicate the observed country's data.*

## Stan code for Bayesian multiparameter evidence synthesis

```
data {
  int<lower=0> N1579; // Population of 15-79 in `Country`
  int<lower=1> Knon; // Number of studies in the ECDC data for `Country`
  int<lower=1> Kever; // Number of studies in the EMCDDA data for ever
users in `Country`
  int<lower=1> Kcur; // Number of studies in the EMCDDA barometer (for
HCV prevalence of PWID) in `Country`
  int<lower=1> NDAA; // Total number of DAAs from 2015 to 2019
  int<lower=1> NDAA_non; // Total number of DAAs from 2016 to 2019

  real p_cur_mean; // Prior mean for the prevalence of current use in
`Country`
  real<lower=0> p_cur_sd; // Prior sd for the prevalence of current use
in `Country`

  real p_ex_mean; // Prior mean for the prevalence of ex-use in `Country`
  real<lower=0> p_ex_sd; // Prior sd for the prevalence of ex-use in
`Country`

  int<lower=0> Nst_CHC_cur[Kcur]; // Number of individuals in the study
estimating CHC among recent PWID
  int<lower=0> Yst_CHC_cur[Kcur]; // Number of HCV+ in the study
estimating CHC among recent PWID

  int<lower=0> Nst_CHC_ever[Kever]; // Number of individuals in the study
estimating CHC among ever IDU
  int<lower=0> Yst_CHC_ever[Kever]; // Number of HCV+ in the study
estimating CHC among ever PWID

  int<lower=0> Nst_CHC_non[Knon]; // Number of individuals in the study
estimating CHC among non PWID
```

```
  int<lower=0> Yst_CHC_non[Knon]; // Number of HCV+ in the study
estimating CHC among non PWID

  vector<lower=0>[3] alpha; // parameter of the Diriclet prior

  real HCVclear_mean; // Prior mean for the HCV clearance probability
  real<lower=0> HCVclear_sd; // Prior sd for the HCV clearance
probability

  real SVR_mean; // Prior mean for the SVR among non-PWID
  real<lower=0> SVR_sd; // Prior sd for the SVR among non-PWID

  real SVR_PWID_mean; // Prior mean for the SVR among PWID
  real<lower=0> SVR_PWID_sd; // Prior sd for the SVR among PWID

  real<lower=0,upper=1> omega_cur; // Proportion of recent PWID among
ever
}

// Block defining the original parameters
parameters {
  // The parameters to be sampled
  simplex[3] rho; // Prevalence of the three risk groups
  real<lower=0,upper=1> CHCpi_cur; // P(CHC+|Cur)
  real<lower=0,upper=1> CHCpi_ex; // P(CHC+|Ex)
  real<lower=0,upper=1> CHCpi_non; // P(CHC+|Non)
  real<lower=0,upper=1> SVR; // SVR of DAAs among non-PWID
  real<lower=0,upper=1> SVR_PWID; // SVR of DAAs among PWID

  real<lower=0,upper=1-CHCpi_cur> HCVclear; // Probability of HCV
clearance; upper bound = 1-prevalence of chronic HCV
}
```

```
transformed parameters {
  // Change scales
  real<lower=0,upper=1> rho_ever;
  real<lower=0,upper=1> CHCpi_ever;
  real<lower=0,upper=1> CHCpi_ever_adj;

  rho_ever = rho[1] + rho[2];
  CHCpi_ever = CHCpi_cur*rho[1]/rho_ever + CHCpi_ex*rho[2]/rho_ever;
  CHCpi_ever_adj = CHCpi_cur*omega_cur + CHCpi_ex*(1-omega_cur);
}


// Binomial regression model
model {
  // Priors
  rho ~ dirichlet(alpha);

  ///////////////////////////////
  // Likelihood contributions //
  ///////////////////////////////

  // Probability of HCV clearance
  HCVclear ~ normal(HCVclear_mean,HCVclear_sd);

  // Prevalence of current use
  rho[1] ~ normal(p_cur_mean,p_cur_sd);

  // Prevalence of ex-use
  rho[2] ~ normal(p_ex_mean,p_ex_sd);
```

```
  // Prevalence of CHC among current users
  Yst_CHC_cur ~ binomial(Nst_CHC_cur,CHCpi_cur);

  // Prevalence of CHC among ever users
  Yst_CHC_ever ~ binomial(Nst_CHC_ever,CHCpi_ever_adj);

  // Prevalence of CHC among non-PWID
  Yst_CHC_non ~ binomial(Nst_CHC_non,CHCpi_non);

  // SVR of DAAs among non-PWID
  SVR ~ normal(SVR_mean,SVR_sd);

  // SVR of DAAs among PWID
  SVR_PWID ~ normal(SVR_PWID_mean,SVR_PWID_sd);
}


generated quantities {
  // Functions of parameters
  real<lower=0,upper=100> overalCHC;
  real logit_CHCpi_cur;
  real logit_CHCpi_ex;
  real logit_CHCpi_non;
  real logit_rho_cur;
  real logit_rho_ex;
  real logit_rho_non;
  real<lower=0,upper=1> pEverGivenCHC;
  real<lower=0,upper=1> pCurGivenCHC;
  real<lower=0,upper=1> pExGivenCHC;
  real<lower=0,upper=1> pNonGivenCHC;
  real logit_HCVclear;
  real<lower=0> NumberCHC;
```

```
  real CHCDAApi_non; // P(CHC+|Non) adjusted for DAA
  real CHCDAApi_cur; // P(CHC+|Cur) adjusted for DAA
  real CHCDAApi_ex; // P(CHC+|Ex) adjusted for DAA
  real CHCDAApi_ever; // P(CHC+|Ever) adjusted for DAA

  real<lower=0,upper=100> overalCHC_DAA;
  real pEverGivenCHC_DAA;
  real pCurGivenCHC_DAA;
  real pExGivenCHC_DAA;
  real pNonGivenCHC_DAA;
  real<lower=0> NumberCHC_DAA;

  // Overall HCV prevalence
  overalCHC = 100*(rho[1]*CHCpi_cur + rho[2]*CHCpi_ex +
rho[3]*CHCpi_non);
  pEverGivenCHC = CHCpi_ever*rho_ever/(overalCHC/100);
  pCurGivenCHC = CHCpi_cur*rho[1]/(overalCHC/100);
  pExGivenCHC = CHCpi_ex*rho[2]/(overalCHC/100);
  pNonGivenCHC = CHCpi_non*(1-rho_ever)/(overalCHC/100);
  NumberCHC = round(overalCHC*N1579/100);

  CHCDAApi_non = ( N1579*rho[3]*CHCpi_non -
NDAA_non*pNonGivenCHC*SVR)/(N1579*rho[3]);
  CHCDAApi_cur = ( N1579*rho[1]*CHCpi_cur -
NDAA*pCurGivenCHC*SVR_PWID_mean)/(N1579*rho[1]);
  CHCDAApi_ex = ( N1579*rho[2]*CHCpi_ex -
NDAA*pExGivenCHC*SVR_PWID_mean)/(N1579*rho[2]);
  CHCDAApi_ever = CHCDAApi_cur*rho[1]/rho_ever +
CHCDAApi_ex*rho[2]/rho_ever;

  overalCHC_DAA = 100*(rho[1]*CHCDAApi_cur + rho[2]*CHCDAApi_ex +
```

```
rho[3]*CHCDAApi_non);
  pEverGivenCHC_DAA = CHCDAApi_ever*rho_ever/(overalCHC_DAA/100);
  pCurGivenCHC_DAA = CHCDAApi_cur*rho[1]/(overalCHC_DAA/100);
  pExGivenCHC_DAA = CHCDAApi_ex*rho[2]/(overalCHC_DAA/100);
  pNonGivenCHC_DAA = CHCDAApi_non*(1-rho_ever)/(overalCHC_DAA/100);
  NumberCHC_DAA = round(overalCHC_DAA*N1579/100);


  logit_rho_cur = logit(rho[1]);
  logit_rho_ex = logit(rho[2]);
  logit_rho_non = logit(rho[3]);
  logit_CHCpi_cur = logit(CHCpi_cur);
  logit_CHCpi_ex = logit(CHCpi_ex);
  logit_CHCpi_non = logit(CHCpi_non);
  logit_HCVclear = logit(HCVclear);
}
```

## Multi-state Markov model

```
#include <stdio.h>
#include <stdlib.h>
#include <string.h>
#include <math.h>
#include <time.h>

#define loops 100
int populationPerYearAndAge[2021][66];
double deathRatePerAge[66];
int totalPopulationPerYear[2021];

FILE *F_DeathRate=fopen("`Country`-deathRates.txt","r");
FILE *F_Population=fopen("`Country`-population.txt","r");
FILE *out;

double pa_start = 0.00012;                  // Setting Variable
double pa_step  = 0.000002;                 // Setting Variable
double pa_stop  = 0.000123;                 // Setting Variable

double pa;
double pg = 1.0/11.7;    // Assumed average active injecting career of
11.7 years
double pk = 0.004*12.0;      // Relapse rate of 0.004/month

struct people{
    int age;
    int state;
};

char filename[100];
struct people person[50000000];
```

```
long int totalPersons=0;

void setPopulationAge()
{
    for( int j=0; j<71; j++){
        for( int i=0; i<51; i++){

fscanf(F_Population,"%d",&populationPerYearAndAge[1950+j][15+i]);
        }
    }
}

void setDeathRate()
{
    for( int i=0; i<51; i++){
        fscanf(F_DeathRate,"%lf",&deathRatePerAge[15+i*5]);
        for( int j=0; j<5; j++){
            deathRatePerAge[15+i*5+j] = deathRatePerAge[15+i*5];
        }
    }
}

void getTotalPopulationPerAge()
{
    for( int j=0; j<71; j++){
        for( int i=0; i<51; i++){
            totalPopulationPerYear[1950+j] +=
populationPerYearAndAge[1950+j][15+i];
        }
    }
}
```

```
void printTotalPopulationPerAge()
{
    printf("---- - ----------\n");
    printf("Year - Population\n");
    printf("---- - ----------\n");
    for( int i=1950; i<2021; i++){
        printf("%d - %d\n",i,totalPopulationPerYear[i]);
    }
}

void printDeathRatePerAge()
{
    printf("--- - -----\n");
    printf("Age - Rate\n");
    printf("--- - -----\n");
    for( int i=15; i<65; i++){
        printf("%d - %lf\n",i,deathRatePerAge[i]);
    }
}

void printTotalPersonPerState( int year)
{
    int count[4]= {0,0,0,0};
    for( int i=0; i<totalPersons; i++){
        count[person[i].state]++;
    }

//printf("%d\t%ld\t%ld\t%ld\t%ld\n",year,count[0],count[1],count[2],count
[3]);
    if(year==2014)

printf("%d\t%ld\t%ld\t%ld\t%ld\t%lf\n",year,count[0],count[1],count[2],co
```

```
unt[3],pa);
    if( year>2009){
        fprintf(out,
"%d\t%ld\t%ld\t%ld\t%ld\n",year,count[0],count[1],count[2],count[3]);
    }
}

void initializePopulation()
{
    int cnt=0;
    for( int j=15; j<65; j++){
        for( int i=0; i<populationPerYearAndAge[1950][j]*100; i++){
            person[cnt].age=j;
            person[cnt].state=0;
            cnt++;
        }
    }
    totalPersons = cnt;
}

bool checkRate(double rate)
{
    if (rate <0.001){
        double k = rand()/(1.00+RAND_MAX);
        if(k<0.001){
            rate = rate*1000;
        }
        else{
            return false;
        }
    }
    double k = rand()/(1.00+RAND_MAX);
```

```
    if ( k < rate )
        return true;
    else
        return false;
}

void changeStatusAndAge()
{
    for( int i=0; i<totalPersons; i++){
        // change state
        double k = rand()/(1.00+RAND_MAX);
        if( person[i].state==0){
            if ( checkRate(pa) &&  person[i].age<40){
                person[i].state=1;
            }
        }
        else if( person[i].state==1){
            if ( k < pg ){
                person[i].state=2;
            }
        }
        else if( person[i].state==2){
            if ( k < pk ){
                person[i].state=1;
            }
        }
        // After their 49th year, all active PWID are assumed to cease
injecting
        if(person[i].age>49 && person[i].state==1){
            person[i].state=2;
        }
        // After their 64th year, remove
```

```
        if(person[i].age>64){
            person[i].state=3;
        }
        // death rate depending on age and state
        k = rand()/(1.00+RAND_MAX);
        if( person[i].state==0){
            if ( k < deathRatePerAge[person[i].age] ){
                person[i].state=3;
            }
        }
        else if( person[i].state==1){
            if ( k < deathRatePerAge[person[i].age] * 13.0 ){
                person[i].state=3;
            }
        }
        else if( person[i].state==2){
            if ( k < deathRatePerAge[person[i].age] ){
                person[i].state=3;
            }
        }
        // increase age one year
        person[i].age++;
    }
}

void addNewPersons(int year)
{
    // add new person [15 years old and non PWID]
    int cnt=totalPersons;
    for( int i=0; i<populationPerYearAndAge[year+1][15]*100; i++){
// 100 instead of 1000 for the population to reduce execution time
        person[cnt].age=15;
```

```
        person[cnt].state=0;
        cnt++;
    }
    // increase the total number of population
    totalPersons = cnt;
}
int main()
{
    srand(time(NULL));

    setPopulationAge();
    setDeathRate();

    getTotalPopulationPerAge();
    pa = pa_start;
    while ( pa < pa_stop){

        snprintf(filename, 100, "result_%lf.txt",pa);
        out=fopen(filename,"w");

        for( int iter=0; iter<loops ;iter++){
            initializePopulation();
            for( int year=1950; year<2020; year++){
                printTotalPersonPerState(year);
                changeStatusAndAge();
                addNewPersons(year);
            }
        }

        fclose(out);
        pa = pa + pa_step;
    }
```

```
}
```

# REFERENCES

Brouard, Cécile, Leïla Saboni, Arnaud Gautier, Stéphane Chevaliez, Delphine Rahib, Jean-Baptiste Richard, Francis Barin, et al. 2019. "HCV and HBV Prevalence Based on Home Blood Self-Sampling and Screening History in the General Population in 2016: Contribution to the New French Screening Strategy." *BMC Infectious Diseases* 19 (1): 896. https://doi.org/10.1186/s12879-019-4493-2.

Cousien, Anthony, Viet Chi Tran, Sylvie Deuffic-Burban, Marie Jauffret-Roustide, Jean-Stéphane Dhersin, and Yazdan Yazdanpanah. 2016. "Hepatitis c Treatment as Prevention of Viral Transmission and Liver-Related Morbidity in Persons Who Inject Drugs." *Hepatology* 63 (4): 1090–1101. https://doi.org/https://doi.org/10.1002/hep.28227.

European Centre for Disease Prevention and Control (ECDC). 2016. "Systematic Review on Hepatitis b and c Prevalence in the EU/EEA." https://www.ecdc.europa.eu/sites/default/files/media/en/publications/Publications/systematic-review-hepatitis-B-C-prevalence.pdf.

———. 2021. "Hepatitis c Prevalence Database." https://www.ecdc.europa.eu/en/all-topics-z/hepatitis-c/tools/hepatitis-c-prevalence-database.

Graf, Christiana, Marcus M Mücke, Georg Dultz, Kai-Henrik Peiffer, Alica Kubesch, Patrick Ingiliz, Stefan Zeuzem, Eva Herrmann, and Johannes Vermehren. 2019. "Efficacy of Direct-acting Antivirals for Chronic Hepatitis C Virus Infection in People Who Inject Drugs or Receive Opioid Substitution Therapy: A Systematic Review and Meta-analysis." *Clinical Infectious Diseases* 70 (11): 2355–65. https://doi.org/10.1093/cid/ciz696.

Grebely, Jason, Sarah Larney, Amy Peacock, Samantha Colledge, Janni Leung, Matthew Hickman, Peter Vickerman, et al. 2019. "Global, Regional, and Country-Level Estimates of Hepatitis c Infection Among People Who Have Recently Injected Drugs." *Addiction* 114 (1): 150–66. https://doi.org/https://doi.org/10.1111/add.14393.

Hines, Lindsey A., Adam Trickey, Janni Leung, Sarah Larney, Amy Peacock, Louisa Degenhardt, Samantha Colledge, et al. 2020. "Associations Between National Development Indicators and the Age Profile of People Who Inject Drugs: Results from a Global Systematic Review and

Meta-Analysis." *The Lancet Global Health* 8 (1): e76–91. https://doi.org/10.1016/S2214-109X(19)30462-0.

Lampertico, Pietro, Jose A. Carrión, Michael Curry, Juan Turnes, Markus Cornberg, Francesco Negro, Ashley Brown, et al. 2020. "Real-World Effectiveness and Safety of Glecaprevir/Pibrentasvir for the Treatment of Patients with Chronic HCV Infection: A meta-Analysis." *Journal of Hepatology* 72 (6): 1112–21. https://doi.org/10.1016/j.jhep.2020.01.025.

McDonald, Scott A., Rosmawati Mohamed, Maznah Dahlui, Herlianna Naning, and Adeeba Kamarulzaman. 2014. "Bridging the Data Gaps in the Epidemiology of Hepatitis c Virus Infection in Malaysia Using Multi-Parameter Evidence Synthesis." *BMC Infectious Diseases* 14 (1): 564. https://doi.org/10.1186/s12879-014-0564-6.

Pol, Stanislas, Fayssoil Fouad, Magali Lemaitre, Ingrid Rodriguez, Olivier Lada, Pascaline Rabiega, Elias Benabadji, and Françoise Roudot-Thoraval. 2022. "Impact of Extending Direct Antiviral Agents (DAA) Availability in France: An Observational Cohort Study (2015-2019) of Data from French Administrative Healthcare Databases (SNDS)." *The Lancet Regional Health – Europe* 13 (February). https://doi.org/10.1016/j.lanepe.2021.100281.

Presanis, Anne M., O. Noel Gill, Timothy R. Chadborn, Caterina Hill, Vivian Hope, Louise Logan, Brian D. Rice, Valerie C. Delpech, A. E. Ades, and Daniela De Angelis. 2010. "Insights into the Rise in HIV Infections, 2001 to 2008: A Bayesian Synthesis of Prevalence Evidence." *AIDS* 24 (18). https://journals.lww.com/aidsonline/Fulltext/2010/11270/Insights_into_the_rise_in_HIV_infections,_2001_to.12.aspx.

Sweeting, M. J., D. De Angelis, M. Hickman, and A. E. Ades. 2008. "Estimating hepatitis C prevalence in England and Wales by synthesizing evidence from multiple data sources. Assessing data conflict and model fit." *Biostatistics* 9 (4): 715–34. https://doi.org/10.1093/biostatistics/kxn004.

Tan, Sarah, Susanna Makela, Daliah Heller, Kevin Konty, Sharon Balter, Tian Zheng, and James H. Stark. 2018. "A Bayesian Evidence Synthesis Approach to Estimate Disease Prevalence in

Hard-to-Reach Populations: Hepatitis c in New York City." *Epidemics* 23: 96–109. https://doi.org/https://doi.org/10.1016/j.epidem.2018.01.002.

Thein, Hla-Hla, Qilong Yi, Gregory J. Dore, and Murray D. Krahn. 2008. "Estimation of Stage-Specific Fibrosis Progression Rates in Chronic Hepatitis c Virus Infection: A Meta-Analysis and Meta-Regression." *Hepatology* 48 (2): 418–31. https://doi.org/https://doi.org/10.1002/hep.22375.

Veen, Maaike G. van, Anne M. Presanis, Stefano Conti, Maria Xiridou, Annemarie R. Stengaard, Martin C. Donoghoe, Ard I. van Sighem, Marianne A. van der Sande, and Daniela De Angelis. 2011. "National Estimate of HIV Prevalence in the Netherlands: Comparison and Applicability of Different Estimation Tools." *AIDS* 25 (2). https://journals.lww.com/aidsonline/Fulltext/2011/01140/National_estimate_of_HIV_prevalence_in_the.14.aspx.

Weill-Barillet, L., J. Pillonel, C. Semaille, L. L?on, Y. Le Strat, X. Pascal, F. Barin, and M. Jauffret-Roustide. 2016. "Hepatitis c Virus and HIV Seroprevalences, Sociodemographic Characteristics, Behaviors and Access to Syringes Among Drug Users, a Comparison of Geographical Areas in France, ANRS-Coquelicot 2011 Survey." *Revue d'?pid?miologie Et de Sant? Publique* 64 (4): 301–12. https://doi.org/https://doi.org/10.1016/j.respe.2015.10.003.

# Estimating the prevalence of chronic hepatitis C infection (CHC) in Germany using Bayesian multiparameter evidence synthesis

11/07/2022

## Table of Contents

Introduction ..... 2

Methods ..... 2

Prevalence of ex-PWID ..... 3

Prevalence of recent PWID ..... 3

Prevalence of CHC among recent PWID ..... 4

Prevalence of CHC among ex-PWID ..... 4

Prevalence of CHC among non-PWID ..... 4

Sensitivity analysis including migrants from endemic countries ..... 6

Treatment adjustment ..... 6

Results ..... 6

Tables and Figures ..... 8

Appendix ..... 12

Fit of the multi-state Markov model ..... 12

Stan code for Bayesian multiparameter evidence synthesis ..... 13

Multi-state Markov model ..... 18

References ..... 26

# INTRODUCTION

Patients with chronic hepatitis C (HCV) infection often remain asymptomatic for decades and even for life, but a proportion of them develop active chronic hepatitis, which is a progressive disease (Thein et al. 2008). Because of the largely asymptomatic nature of HCV infection, notification data reflect national screening and testing practices and do not give accurate insights into the prevalence of HCV infection. Although there are studies that have estimated the prevalence of HCV antibodies (anti-HCV) in specific groups such as the general population, first-time blood donors, or people who inject drugs (PWID) at drug treatment centers, those alone cannot be combined to produce national estimates unless some additional information regarding the composition of each HCV risk group and its prevalence in the population are known (Sweeting et al. 2008; McDonald et al. 2014; Tan et al. 2018).

# METHODS

Bayesian multiparameter evidence synthesis (MPES) has been a popular approach to formally estimate anti-HCV or HIV prevalence (Sweeting et al. 2008; McDonald et al. 2014; Tan et al. 2018; Presanis et al. 2010; Veen et al. 2011). In this project, we extend this method to estimating the CHC prevalence, with the population (15-79 years) split into three main non-overlapping risk groups, i.e., recent PWID (those who have injected in the last year), ex-PWID, and non-PWID. A unified model is assumed including parameters associated with the CHC prevalence of recent, ex-PWID, and non-PWID (denoted by $\pi_{rec}$, $\pi_{ex}$, and $\pi_{non}$, respectively) and the prevalence of these risk groups in the population (proportion of the population that belongs to each group), denoted by $\rho_{rec}$, $\rho_{ex}$, and $\rho_{non}$, respectively; $\rho_{rec} + \rho_{ex} + \rho_{non} = 1$. To estimate the CHC prevalence in the whole population, $\pi$, we used the formula:

$$\pi = \pi_{rec}\rho_{rec} + \pi_{ex}\rho_{ex} + \pi_{non}\rho_{non} \quad (1)$$

Bayesian synthesis considers all available data, allowing for an evidence-based estimation of CHC prevalence in the population, with inherent uncertainty properly accounted for. Our aim is to estimate the CHC prevalence for the total (15-79) population of Germany in 2019 using sources of information over the last decade (2010-2019).

## Prevalence of ex-PWID

Data on the prevalence of ex-PWID (proportion of the population that is ex-PWID) are sparse and generally often unreliable. To overcome this limitation, we apply the method proposed by McDonald et al. (2014), which requires simulating a multi-state Markov model representing the non-PWID, recent PWID, and ex-PWID. In the multi-state Markov model, the simulation starts in 1950 and examines the population aged 15-79 years old. A certain number of 15-year-old individuals enter the simulation process each year. These numbers of 15-year-old individuals are obtained from the United Nations Statistic Division UNSD, being different for each country. The ageing of the population is also taken into account. Country-specific overall mortality data are retrieved from the Life Tables of the World Health Organization WHO. The duration of injecting career is also country-specific and obtained from the paper of Hines et al. (2020).

To estimate the number of ex-PWID, the model can be calibrated on the number of recent PWID provided in the EMCDDA barometer or the EMCDDA statistical bulletin (the code of the Markov model can be found in the Appendix). If there are no available data in the barometer, estimates from the systematic review of Grebely et al. (2019) could be used. Currently, the multi-state Markov model was calibrated on the estimated prevalence of recent PWID in 2000 reported in the systematic review of Grebely et al. (2019).

After applying the model for Germany, we compute the number (and the corresponding Confidence Interval - CI) of ex-PWID in 2019. The number of ex-PWID is then divided by the population size (15-79 years) in the same year, and a Normal distribution, constrained such that $\rho_{ex} \in (0,1)$, is assumed for $\rho_{ex}$. The standard deviation of this distribution is specified to approximately correspond to the respective CI, i.e. $\left(UB_{\rho_{ex}} - LB_{\rho_{ex}}\right)/(2 \times 1.96)$, where $UB_{\rho_{ex}}$ and $LB_{\rho_{ex}}$ denote the upper and lower limit of the CI.

## Prevalence of recent PWID

The number of recent PWID in Germany in 2019 is also projected by the multi-state Markov model described in the previous subsection. To estimate $\rho_{rec}$, the number of recent PWID is divided by the population size (15-79 years). Similar to the previous subsection, a Normal

distribution, constrained such that $\rho_{rec} \in (0,1)$, is assumed for $\rho_{rec}$, with the standard deviation specified to correspond to the CI obtained from the multi-state Markov model.

## Prevalence of CHC among recent PWID

The prevalence of CHC among recent PWID was informed by CHC prevalence data from a cross-sectional study using respondent-driven sampling in eight German cities in 2011-14 (Wenz et al. 2016). The Binomial distribution was used in the model. However, some people may have been treated with direct-acting antivirals (DAAs). Based on the information provided by the national focal point, the number of individuals treated with DAAs up to 2019 is equal to 76,400. However, the proportions of each risk group among those treated with DAAs are not currently available in Germany. Thus, in this report, we only subtract individuals cured with DAAs from the whole population, i.e. we adjust $\pi$ for DAA uptake.

## Prevalence of CHC among ex-PWID

Information on CHC prevalence among ex-PWID (i.e., $\pi_{ex}$) is difficult to obtain directly. To overcome this, nationwide data on the HCV prevalence among ever users could be used. Thus, if an estimate of $\pi_{ever}$ is available, $\pi_{ex}$ can be indirectly estimated since $\pi_{ever}$ is equal to a weighted average of $\pi_{rec}$ and $\pi_{ex}$, as shown by the following formula

$$\pi_{ever} = \frac{\rho_{rec}}{\rho_{rec} + \rho_{ex}} \pi_{rec} + \frac{\rho_{ex}}{\rho_{rec} + \rho_{ex}} \pi_{ex}, \quad (2)$$

or, equivalently,

$$\pi_{ex} = \left(\pi_{ever} - \frac{\rho_{rec}}{\rho_{rec} + \rho_{ex}} \pi_{rec}\right) \times \frac{\rho_{rec} + \rho_{ex}}{\rho_{ex}}, \quad (3)$$

Currently, as a proxy for the population having ever injected drugs, CHC prevalence data from a nationwide sample of patients in opioid substitution treatment (OST) (Schulte et al. 2019) in 2016 were used.

## Prevalence of CHC among non-PWID

To estimate the CHC prevalence among non-PWID in a country (after 2010), the ECDC database (European Centre for Disease Prevention and Control (ECDC) 2021) was used. The

ECDC group has carefully and critically collected information from relevant studies across the EU/EEA region in cooperation with the focal points of each country. On the basis of the representativeness of each study and other factors (European Centre for Disease Prevention and Control (ECDC) 2016), a risk of bias score was assigned to each study, with higher values indicating higher-quality studies. When higher-quality studies are available in a country ($\geq 4$), they are used to estimate the overall CHC prevalence in that country among non-PWID. If there are no higher-quality studies, general population estimates with a lower quality (<4) are pooled. If no general population prevalence estimates are available, data on the prevalence of HCV in pregnant women could be used as a prevalence measure. Finally, if data on pregnant women are lacking too, first-time blood donor studies could be an option. However, if the national focal point agrees or recommends, estimates from a neighboring country could be used instead, or we could use any other relevant information provided/suggested by the national focal point.

We directly use CHC prevalence data from studies in the general population, if available. If the data in the general population refer to anti-HCV prevalence (without any data on the viremic population), we adjust the estimates to get CHC prevalence based on the spontaneous HCV clearance estimate of 26% (Micallef, Kaldor, and Dore 2006).

As $\rho_{rec}$ and $\rho_{ex}$ are likely to be low, $\pi_{non}$ will be the most critical factor for the estimation of the overall CHC prevalence in the population. To adjust for potential heterogeneity between the CHC prevalence estimates from different studies in the non-PWID population in a country, as a sensitivity analysis, we also apply a random-effect meta-analytic approach to pool the CHC prevalence across studies on non-PWID (Lin and Chu 2020). The random-effect approach is carried out only when at least 3 studies are available in the general population of a certain country, and the estimates are compared with the corresponding estimates from the approach assuming no heterogeneity (similar to a fixed-effect meta-analysis). If the estimates are inconsistent, possible explanations are investigated and discussed. If there is substantial heterogeneity between studies, it would help more to explore the causes; for example, following national focal point suggestions, some studies could be excluded to reduce heterogeneity.

## Sensitivity analysis including migrants from endemic countries

A sensitivity analysis including data for the viremic population among migrants based on the report of the ECDC group (European Centre for Disease Prevention and Control 2016) was carried out. Migrants from endemic countries represent 12.8% of the adult population in Germany (Table 8 in European Centre for Disease Prevention and Control (2016)), with the respective CHC prevalence being equal to $\pi_{mig} = 1.4\%$ (Table 9 in European Centre for Disease Prevention and Control (2016)). The overall CHC prevalence is now estimated by

$$\pi = \pi_{rec}\rho_{rec} + \pi_{ex}\rho_{ex} + \pi_{mig}\rho_{mig} + \pi_{non}\rho_{non},$$

where $\rho_{rec} + \rho_{ex} + \rho_{mig} + \rho_{non} = 1$.

## Treatment adjustment

In Germany, based on the information provided by the national focal point, the number of individuals treated with DAAs up to 2019 is equal to 76,400. Thus, to take DAAs into account, the overall CHC prevalence is adjusted by stochastically subtracting the individuals cured with DAAs from the infected population, i.e.

$$\pi = \frac{N_{15,79}\tilde{\pi} - N_{DAA}SVR}{N_{15,79}}, \quad (4)$$

where $\tilde{\pi}$ denotes the overall CHC prevalence when information on DAAs is ignored and $SVR$ is the sustained virologic response of DAAs, estimated to be 96.7% (95% CI: 95.4% to 98.1%) (Lampertico et al. 2020).

# RESULTS

The above-mentioned approach was used to estimate the prevalence of CHC in 2019 (considering ages between 15-79 years: 66,339,292).

The aggregated data used by our approach are briefly presented in Table 1. The multi-state Markov model was calibrated on the estimated prevalence of recent PWID in 2000 reported in the systematic review of Grebely et al. (2019). In Germany, there was 1 study on non-PWID of high quality, which included CHC data (Poethko-Müller et al. 2013). To estimate the CHC

prevalence in the general population (primarily non-PWID), we used a fixed-effect meta-analytic approach, with the corresponding results presented in Table 2. The prevalence of recent and ex-PWID (proportion of the population that belongs to these groups) was low in Germany (about 0.24% and 0.49%, respectively) corresponding to 159,840 (95% CI: 156,885-162,355) recent PWID and 327,645 (95% CI: 322,910-331,385) ex-PWID in the population. However, the CHC prevalence in these groups was substantial (Table 2) being 44.01% and 19.17%, respectively. This translates to 70,340 (95% CI: 66,826-73,989) and 62,796 (95% CI: 53,325-72,524) recent and ex-PWID aged 15-79 living with CHC infection in Germany in 2019. The CHC prevalence in the general population was 0.21% (95% CI: 0.12%-0.33%), much lower than that of the high-risk groups. Taking all pieces of information into account, the overall CHC prevalence in Germany in 2019 was equal to 0.41% (95% CI: 0.32%-0.53%), which corresponds to 270,486 (95% CI: 211,243-353,615) individuals aged 15-79 years with CHC infection. However, when information on DAAs is considered, the overall CHC prevalence in Germany in 2019 was equal to 0.3% (95% CI: 0.21%-0.42%), which corresponds to 196,671 (95% CI: 137,554.8-279,639.3) individuals aged 15-79 years with CHC infection.

The results from our model including migrants from endemic countries as a separate group are presented in Table 3. However, this analysis comes with possible limitations; that is, including migrants as a separate group is valid only if migrants do not overlap with the remaining groups (recent PWID, ex-PWID, and non-PWID). According to the national focal point, though, the study on the general population (Poethko-Müller et al. 2013) does not representatively include migrants. Thus, after subtracting the total number of DAA-treated persons, the results including migrants as a separate risk group (Table 3) may be considered as an upper bound for the total estimate of the infected population.

# TABLES AND FIGURES

*Table 1. Data (real) contributing to the estimation of CHC prevalence in Germany in 2019.*

| Parameter | Number (95%CI) | Numerator | Denominator | Notes | Year of study |
|---|---|---|---|---|---|
| $\rho_{rec}$ | 159,840 (156,885-162,355) | | | Method based on McDonald et al. | 2019 |
| $\rho_{ex}$ | 327,645 (322,910-331,385) | | | Method based on McDonald et al. | 2019 |
| $\pi_{rec}$ | | 914 | 2,077 | (Wenz et al. 2016) | 2011-14 |
| $\pi_{ever}$ | | 617 | 2,260 | (Schulte et al. 2019) | 2016 |
| $\pi_{non}$ | | 14 | 7,047 | ECDC database (Poethko-Muller et al.); Risk of bias=5 | 2011 |

*Notes*: Although it looks counter-intuitive, **higher risk of bias score denotes a higher-quality study** (range from 0 to 6).

*Table 2. Results from the method assuming no heterogeneity in the estimation of the CHC prevalence in the general population from different studies (i.e., similar to a fixed-effect meta-analysis).*

| Parameter | Posterior median | Posterior sd | LB (2.5%) | UB (97.5%) |
|---|---|---|---|---|
| $\rho_{rec}$ (%) | 0.24 | 0 | 0.24 | 0.25 |
| $\rho_{ex}$ (%) | 0.49 | 0 | 0.49 | 0.5 |
| $\pi_{rec}$ (%) | 44.01 | 1.07 | 41.93 | 46.12 |
| $\pi_{ex}$ (%) | 19.17 | 1.48 | 16.3 | 22.09 |
| $\pi_{non}$ (%) | 0.21 | 0.05 | 0.12 | 0.33 |
| $\pi$ (%) | 0.41 | 0.05 | 0.32 | 0.53 |
| Number with CHC | 270,486 | 36,345 | 211,243 | 353,615 |
| $\pi$ † (%) | 0.3 | 0.05 | 0.21 | 0.42 |
| Number with CHC† | 196,671 | 36,346 | 137,554 | 279,639 |
| Pr(Recent PWID\|CHC) (%) | 26 | 3.5 | 19.78 | 33.51 |
| Pr(Ex-PWID\|CHC) (%) | 23.21 | 3.36 | 17.3 | 30.26 |
| Pr(Non-PWID\|CHC) (%) | 50.71 | 6.49 | 37.01 | 62.34 |

*Notes*: $\rho_{rec}$, prevalence of recent PWID (proportion of the population that is recent PWID); $\rho_{ex}$, prevalence of ex-PWID (proportion of the population that is ex-PWID); $\pi_{rec}$, CHC prevalence among recent PWID; $\pi_{ex}$, CHC prevalence among ex-PWID; $\pi_{non}$, CHC prevalence among non-PWID; $\pi$ overall CHC prevalence in Germany; $\pi$ † overall CHC prevalence in Germany adjusted for DAAs; LB, Lower Boundary; UP, Upper Boundary

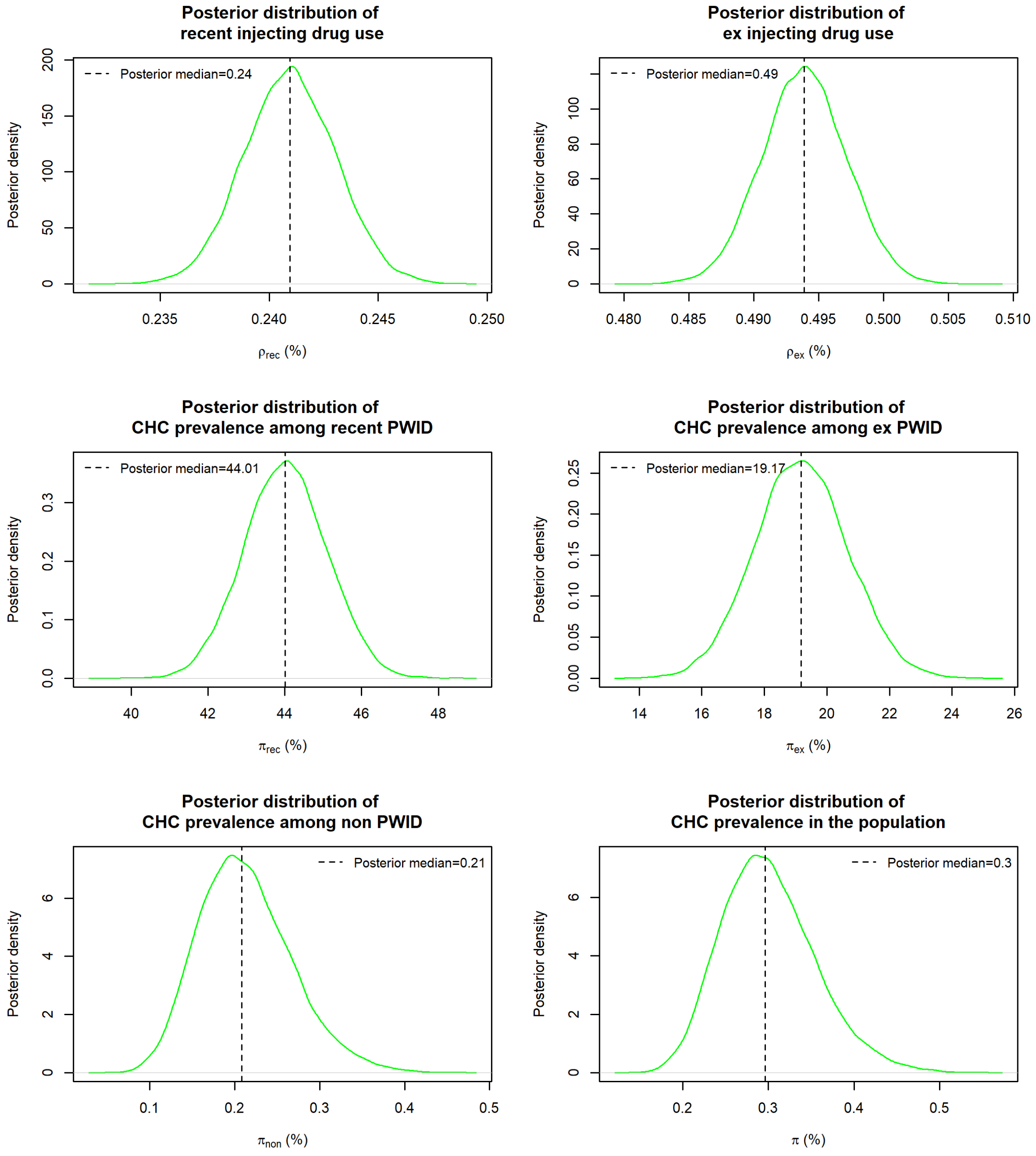


*Figure 1. Posterior distributions of the key model parameters using the approach that assumes no heterogeneity in the estimation of the CHC prevalence in the general population (primarily non-PWID) from different studies (i.e., similar to a fixed-effect meta-analysis).*

*Table 3. Results from the method assuming no heterogeneity in the estimation of the CHC prevalence in the general population (primarily non-PWID) from different studies (i.e., similar to a fixed-effect meta-analysis) including migrants from endemic countries as a separate group.*

| Parameter | Posterior median | Posterior sd | LB (2.5%) | UB (97.5%) |
|---|---|---|---|---|
| $\rho_{rec}$ (%) | 0.24 | 0 | 0.24 | 0.25 |
| $\rho_{ex}$ (%) | 0.49 | 0 | 0.49 | 0.5 |
| $\rho_{mig}$ (%) | 12.8 | 0 | 12.8 | 12.8 |
| $\pi_{rec}$ (%) | 44.02 | 1.07 | 41.93 | 46.11 |
| $\pi_{ex}$ (%) | 19.16 | 1.49 | 16.27 | 22.13 |
| $\pi_{mig}$ (%) | 1.4 | 0.39 | 0.63 | 2.14 |
| $\pi_{non}$ (%) | 0.21 | 0.05 | 0.12 | 0.33 |
| $\pi$ (%) | 0.56 | 0.07 | 0.43 | 0.7 |
| Number with CHC | 372,682 | 45,468 | 286,193 | 465,219 |
| $\pi$ † (%) | 0.45 | 0.07 | 0.32 | 0.59 |
| Number with CHC† | 298,776 | 45,460 | 212,394 | 391,392 |
| Pr(Recent PWID\|CHC) (%) | 18.86 | 2.46 | 15.04 | 24.71 |
| Pr(Ex-PWID\|CHC) (%) | 16.85 | 2.4 | 12.95 | 22.34 |
| Pr(Mig\|CHC) (%) | 31.79 | 6.72 | 16.96 | 43.55 |
| Pr(Non-PWID\|CHC) (%) | 32.36 | 6.26 | 20.8 | 45.28 |

*Notes*: $\rho_{rec}$, prevalence of recent PWID (proportion of the population that is recent PWID); $\rho_{ex}$, prevalence of ex-PWID (proportion of the population that is ex-PWID); $\rho_{mig}$, prevalence of migrants from endemic countries (proportion of the population that belongs to this group); $\pi_{rec}$, CHC prevalence among recent PWID; $\pi_{ex}$, CHC prevalence among ex-PWID; $\pi_{mig}$, CHC prevalence among migrants from endemic countries; $\pi_{non}$, CHC prevalence among non-PWID; $\pi$ overall CHC prevalence in Germany; $\pi$ † overall CHC prevalence in Germany adjusted for DAAs; LB, Lower Boundary; UP, Upper Boundary

# APPENDIX

## Fit of the multi-state Markov model

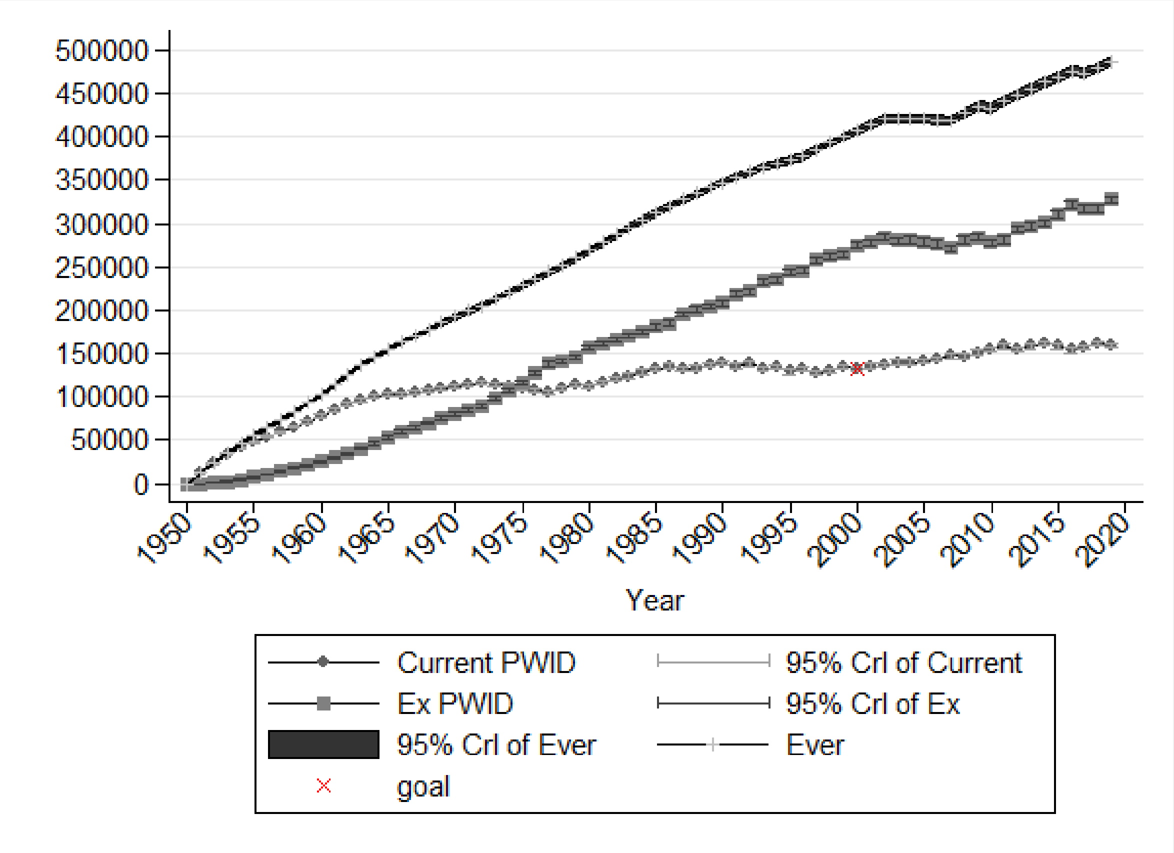


*Figure 2: Model predictions for the numbers of current and ex-PWID (people who inject drugs). The solid black line and shaded grey error bars show the median and 95% credible intervals (95% CrI) for the model projections. For comparison, asterisks indicate the observed country's data.*

## Stan code for Bayesian multiparameter evidence synthesis

```
data {
  int<lower=0> N1579; // Population of 15-79 in `Country`
  int<lower=1> Knon; // Number of studies in the ECDC data for `Country`
  int<lower=1> Kever; // Number of studies in the EMCDDA data for ever
users in `Country`
  int<lower=1> Kcur; // Number of studies in the EMCDDA barometer (for
HCV prevalence of PWID) in `Country`
  int<lower=1> NDAA; // Total number of DAAs from 2015 to 2019

  real p_cur_mean; // Prior mean for the prevalence of current use in
`Country`
  real<lower=0> p_cur_sd; // Prior sd for the prevalence of current use
in `Country`

  real p_ex_mean; // Prior mean for the prevalence of ex-use in `Country`
  real<lower=0> p_ex_sd; // Prior sd for the prevalence of ex-use in
`Country`

  int<lower=0> Nst_CHC_cur[Kcur]; // Number of individuals in the study
estimating CHC among recent PWID
  int<lower=0> Yst_CHC_cur[Kcur]; // Number of HCV+ in the study
estimating CHC among recent PWID

  int<lower=0> Nst_CHC_ever[Kever]; // Number of individuals in the study
estimating CHC among ever IDU
  int<lower=0> Yst_CHC_ever[Kever]; // Number of HCV+ in the study
estimating CHC among ever PWID

  int<lower=0> Nst_CHC_non[Knon]; // Number of individuals in the study
estimating CHC among non PWID
  int<lower=0> Yst_CHC_non[Knon]; // Number of HCV+ in the study
```

```
estimating CHC among non PWID

  vector<lower=0>[3] alpha; // parameter of the Diriclet prior

  real HCVclear_mean; // Prior mean for the HCV clearance probability
  real<lower=0> HCVclear_sd; // Prior sd for the HCV clearance
probability

  real SVR_mean; // Prior mean for the SVR among non-PWID
  real<lower=0> SVR_sd; // Prior sd for the SVR among non-PWID

  real SVR_PWID_mean; // Prior mean for the SVR among PWID
  real<lower=0> SVR_PWID_sd; // Prior sd for the SVR among PWID
}

// Block defining the original parameters
parameters {
  // The parameters to be sampled
  simplex[3] rho; // Prevalence of the three risk groups
  real<lower=0,upper=1> CHCpi_cur; // P(CHC+|Cur)
  real<lower=0,upper=1> CHCpi_ex; // P(CHC+|Ex)
  real<lower=0,upper=1> CHCpi_non; // P(CHC+|Non)
  real<lower=0,upper=1> SVR; // SVR of DAAs among non-PWID
  real<lower=0,upper=1> SVR_PWID; // SVR of DAAs among PWID

  real<lower=0,upper=1-CHCpi_cur> HCVclear; // Probability of HCV
clearance; upper bound = 1-prevalence of chronic HCV
}


transformed parameters {
  // Change scales
```

```
  real<lower=0,upper=1> rho_ever;
  real<lower=0,upper=1> CHCpi_ever;

  rho_ever = rho[1] + rho[2];
  CHCpi_ever = CHCpi_cur*rho[1]/rho_ever + CHCpi_ex*rho[2]/rho_ever;
}


// Binomial regression model
model {
  // Priors
  rho ~ dirichlet(alpha);

  //////////////////////////////
  // Likelihood contributions //
  //////////////////////////////

  // Probability of HCV clearance
  HCVclear ~ normal(HCVclear_mean,HCVclear_sd);

  // Prevalence of current use
  rho[1] ~ normal(p_cur_mean,p_cur_sd);

  // Prevalence of ex-use
  rho[2] ~ normal(p_ex_mean,p_ex_sd);

  // Prevalence of chronic HCV among current users
  Yst_CHC_cur ~ binomial(Nst_CHC_cur,CHCpi_cur);

  // Prevalence of HCV among ever users
  Yst_CHC_ever ~ binomial(Nst_CHC_ever,CHCpi_ever);
```

```
  // HCV+ among non
  Yst_CHC_non ~ binomial(Nst_CHC_non,CHCpi_non);

  // SVR of DAAs among non-PWID
  SVR ~ normal(SVR_mean,SVR_sd);

  // SVR of DAAs among PWID
  SVR_PWID ~ normal(SVR_PWID_mean,SVR_PWID_sd);
}


generated quantities {
  // Functions of parameters
  real<lower=0,upper=100> overalCHC;
  real logit_CHCpi_cur;
  real logit_CHCpi_ex;
  real logit_CHCpi_non;
  real logit_rho_cur;
  real logit_rho_ex;
  real logit_rho_non;
  real<lower=0,upper=1> pEverGivenCHC;
  real<lower=0,upper=1> pCurGivenCHC;
  real<lower=0,upper=1> pExGivenCHC;
  real<lower=0,upper=1> pNonGivenCHC;
  real logit_HCVclear;
  real<lower=0> NumberCHC;
  real<lower=0,upper=100> overalCHC_DAA;
  real<lower=0> NumberCHC_DAA;

  // Overall HCV prevalence
  overalCHC = 100*(rho[1]*CHCpi_cur + rho[2]*CHCpi_ex +
rho[3]*CHCpi_non);
```

```
  pEverGivenCHC = CHCpi_ever*rho_ever/(overalCHC/100);
  pCurGivenCHC = CHCpi_cur*rho[1]/(overalCHC/100);
  pExGivenCHC = CHCpi_ex*rho[2]/(overalCHC/100);
  pNonGivenCHC = CHCpi_non*(1-rho_ever)/(overalCHC/100);
  NumberCHC = round(overalCHC*N1579/100);

  NumberCHC_DAA = NumberCHC - NDAA*SVR;
  overalCHC_DAA = round(100*NumberCHC_DAA/N1579);

  logit_rho_cur = logit(rho[1]);
  logit_rho_ex = logit(rho[2]);
  logit_rho_non = logit(rho[3]);
  logit_CHCpi_cur = logit(CHCpi_cur);
  logit_CHCpi_ex = logit(CHCpi_ex);
  logit_CHCpi_non = logit(CHCpi_non);
  logit_HCVclear = logit(HCVclear);
}
```

## Multi-state Markov model

```
#include <stdio.h>
#include <stdlib.h>
#include <string.h>
#include <math.h>
#include <time.h>

#define loops 100
int populationPerYearAndAge[2021][66];
double deathRatePerAge[66];
int totalPopulationPerYear[2021];

FILE *F_DeathRate=fopen("`Country`-deathRates.txt","r");
FILE *F_Population=fopen("`Country`-population.txt","r");
FILE *out;

double pa_start = 0.00012;                    // Setting Variable
double pa_step  = 0.000002;                   // Setting Variable
double pa_stop  = 0.000123;                   // Setting Variable

double pa;
double pg = 1.0/11.7;    // Assumed average active injecting career of 
11.7 years
double pk = 0.004*12.0;     // Relapse rate of 0.004/month

struct people{
    int age;
    int state;
};

char filename[100];
struct people person[50000000];
```

```
long int totalPersons=0;

void setPopulationAge()
{
    for( int j=0; j<71; j++){
        for( int i=0; i<51; i++){

fscanf(F_Population,"%d",&populationPerYearAndAge[1950+j][15+i]);
        }
    }
}

void setDeathRate()
{
    for( int i=0; i<51; i++){
        fscanf(F_DeathRate,"%lf",&deathRatePerAge[15+i*5]);
        for( int j=0; j<5; j++){
            deathRatePerAge[15+i*5+j] = deathRatePerAge[15+i*5];
        }
    }
}

void getTotalPopulationPerAge()
{
    for( int j=0; j<71; j++){
        for( int i=0; i<51; i++){
            totalPopulationPerYear[1950+j] +=
populationPerYearAndAge[1950+j][15+i];
        }
    }
}
```

```
void printTotalPopulationPerAge()
{
    printf("---- - ----------\n");
    printf("Year - Population\n");
    printf("---- - ----------\n");
    for( int i=1950; i<2021; i++){
        printf("%d - %d\n",i,totalPopulationPerYear[i]);
    }
}

void printDeathRatePerAge()
{
    printf("--- - -----\n");
    printf("Age - Rate\n");
    printf("--- - -----\n");
    for( int i=15; i<65; i++){
        printf("%d - %lf\n",i,deathRatePerAge[i]);
    }
}

void printTotalPersonPerState( int year)
{
    int count[4]= {0,0,0,0};
    for( int i=0; i<totalPersons; i++){
        count[person[i].state]++;
    }

//printf("%d\t%ld\t%ld\t%ld\t%ld\n",year,count[0],count[1],count[2],count
[3]);
    if(year==2014)

printf("%d\t%ld\t%ld\t%ld\t%ld\t%lf\n",year,count[0],count[1],count[2],co
```

```
unt[3],pa);
    if( year>2009){
        fprintf(out,
"%d\t%ld\t%ld\t%ld\t%ld\n",year,count[0],count[1],count[2],count[3]);
    }
}

void initializePopulation()
{
    int cnt=0;
    for( int j=15; j<65; j++){
        for( int i=0; i<populationPerYearAndAge[1950][j]*100; i++){
            person[cnt].age=j;
            person[cnt].state=0;
            cnt++;
        }
    }
    totalPersons = cnt;
}

bool checkRate(double rate)
{
    if (rate <0.001){
        double k = rand()/(1.00+RAND_MAX);
        if(k<0.001){
            rate = rate*1000;
        }
        else{
            return false;
        }
    }
    double k = rand()/(1.00+RAND_MAX);
```

```
    if ( k < rate )
        return true;
    else
        return false;
}

void changeStatusAndAge()
{
    for( int i=0; i<totalPersons; i++){
        // change state
        double k = rand()/(1.00+RAND_MAX);
        if( person[i].state==0){
            if ( checkRate(pa) &&  person[i].age<40){
                person[i].state=1;
            }
        }
        else if( person[i].state==1){
            if ( k < pg ){
                person[i].state=2;
            }
        }
        else if( person[i].state==2){
            if ( k < pk ){
                person[i].state=1;
            }
        }
        // After their 49th year, all active PWID are assumed to cease 
injecting
        if(person[i].age>49 && person[i].state==1){
            person[i].state=2;
        }
        // After their 64th year, remove
```

```
        if(person[i].age>64){
            person[i].state=3;
        }
        // death rate depending on age and state
        k = rand()/(1.00+RAND_MAX);
        if( person[i].state==0){
            if ( k < deathRatePerAge[person[i].age] ){
                person[i].state=3;
            }
        }
        else if( person[i].state==1){
            if ( k < deathRatePerAge[person[i].age] * 13.0 ){
                person[i].state=3;
            }
        }
        else if( person[i].state==2){
            if ( k < deathRatePerAge[person[i].age] ){
                person[i].state=3;
            }
        }
        // increase age one year
        person[i].age++;
    }
}

void addNewPersons(int year)
{
    // add new person [15 years old and non PWID]
    int cnt=totalPersons;
    for( int i=0; i<populationPerYearAndAge[year+1][15]*100; i++){
// 100 instead of 1000 for the population to reduce execution time
        person[cnt].age=15;
```

```
        person[cnt].state=0;
        cnt++;
    }
    // increase the total number of population
    totalPersons = cnt;
}
int main()
{
    srand(time(NULL));

    setPopulationAge();
    setDeathRate();

    getTotalPopulationPerAge();
    pa = pa_start;
    while ( pa < pa_stop){

        snprintf(filename, 100, "result_%lf.txt",pa);
        out=fopen(filename,"w");

        for( int iter=0; iter<loops ;iter++){
            initializePopulation();
            for( int year=1950; year<2020; year++){
                printTotalPersonPerState(year);
                changeStatusAndAge();
                addNewPersons(year);
            }
        }

        fclose(out);
        pa = pa + pa_step;
    }
```

```
}
```

# REFERENCES

European Centre for Disease Prevention and Control. 2016. "Epidemiological Assessment of Hepatitis b and c Among Migrants in the EU/EEA." https://www.ecdc.europa.eu/sites/default/files/media/en/publications/Publications/epidemiological-assessment-hepatitis-B-and-C-among-migrants-EU-EEA.pdf.

European Centre for Disease Prevention and Control (ECDC). 2016. "Systematic Review on Hepatitis b and c Prevalence in the EU/EEA." https://www.ecdc.europa.eu/sites/default/files/media/en/publications/Publications/systematic-review-hepatitis-B-C-prevalence.pdf.

———. 2021. "Hepatitis c Prevalence Database." https://www.ecdc.europa.eu/en/all-topics-z/hepatitis-c/tools/hepatitis-c-prevalence-database.

Grebely, Jason, Sarah Larney, Amy Peacock, Samantha Colledge, Janni Leung, Matthew Hickman, Peter Vickerman, et al. 2019. "Global, Regional, and Country-Level Estimates of Hepatitis c Infection Among People Who Have Recently Injected Drugs." *Addiction* 114 (1): 150–66. https://doi.org/https://doi.org/10.1111/add.14393.

Hines, Lindsey A., Adam Trickey, Janni Leung, Sarah Larney, Amy Peacock, Louisa Degenhardt, Samantha Colledge, et al. 2020. "Associations Between National Development Indicators and the Age Profile of People Who Inject Drugs: Results from a Global Systematic Review and Meta-Analysis." *The Lancet Global Health* 8 (1): e76–91. https://doi.org/10.1016/S2214-109X(19)30462-0.

Lampertico, Pietro, Jose A. Carrión, Michael Curry, Juan Turnes, Markus Cornberg, Francesco Negro, Ashley Brown, et al. 2020. "Real-World Effectiveness and Safety of Glecaprevir/Pibrentasvir for the Treatment of Patients with Chronic HCV Infection: A meta-Analysis." *Journal of Hepatology* 72 (6): 1112–21. https://doi.org/10.1016/j.jhep.2020.01.025.

Lin, Lifeng, and Haitao Chu. 2020. "Meta-Analysis of Proportions Using Generalized Linear Mixed Models." *Epidemiology* 31 (5).

https://journals.lww.com/epidem/Fulltext/2020/09000/Meta_analysis_of_Proportions_Using_Generalized.16.aspx.

McDonald, Scott A., Rosmawati Mohamed, Maznah Dahlui, Herlianna Naning, and Adeeba Kamarulzaman. 2014. "Bridging the Data Gaps in the Epidemiology of Hepatitis c Virus Infection in Malaysia Using Multi-Parameter Evidence Synthesis." *BMC Infectious Diseases* 14 (1): 564. https://doi.org/10.1186/s12879-014-0564-6.

Micallef, J. M., J. M. Kaldor, and G. J. Dore. 2006. "Spontaneous Viral Clearance Following Acute Hepatitis c Infection: A Systematic Review of Longitudinal Studies." *Journal of Viral Hepatitis* 13 (1): 34–41. https://doi.org/https://doi.org/10.1111/j.1365-2893.2005.00651.x.

Poethko-Müller, C., R. Zimmermann, O. Hamouda, M. Faber, K. Stark, R. S. Ross, and M. Thamm. 2013. "Die Seroepidemiologie Der Hepatitis?a, b Und c in Deutschland." *Bundesgesundheitsblatt - Gesundheitsforschung - Gesundheitsschutz* 56 (5): 707–15. https://doi.org/10.1007/s00103-013-1673-x.

Presanis, Anne M., O. Noel Gill, Timothy R. Chadborn, Caterina Hill, Vivian Hope, Louise Logan, Brian D. Rice, Valerie C. Delpech, A. E. Ades, and Daniela De Angelis. 2010. "Insights into the Rise in HIV Infections, 2001 to 2008: A Bayesian Synthesis of Prevalence Evidence." *AIDS* 24 (18). https://journals.lww.com/aidsonline/Fulltext/2010/11270/Insights_into_the_rise_in_HIV_infections,_2001_to.12.aspx.

Schulte, Bernd, Christiane Sybille Schmidt, Lisa Strada, Moritz Rosenkranz, Ingo Sch?fer, Uwe Verthein, and Jens Reimer. 2019. "Hepatitis C Virus Prevalence and Incidence in a Large Nationwide Sample of Patients in Opioid Substitution Treatment in Germany: A Prospective Cohort Study." *Clinical Infectious Diseases* 70 (10): 2199–2205. https://doi.org/10.1093/cid/ciz661.

Sweeting, M. J., D. De Angelis, M. Hickman, and A. E. Ades. 2008. "Estimating hepatitis C prevalence in England and Wales by synthesizing evidence from multiple data sources. Assessing data conflict and model fit." *Biostatistics* 9 (4): 715–34. https://doi.org/10.1093/biostatistics/kxn004.

Tan, Sarah, Susanna Makela, Daliah Heller, Kevin Konty, Sharon Balter, Tian Zheng, and James H. Stark. 2018. “A Bayesian Evidence Synthesis Approach to Estimate Disease Prevalence in Hard-to-Reach Populations: Hepatitis c in New York City.” *Epidemics* 23: 96–109. https://doi.org/https://doi.org/10.1016/j.epidem.2018.01.002.

Thein, Hla-Hla, Qilong Yi, Gregory J. Dore, and Murray D. Krahn. 2008. “Estimation of Stage-Specific Fibrosis Progression Rates in Chronic Hepatitis c Virus Infection: A Meta-Analysis and Meta-Regression.” *Hepatology* 48 (2): 418–31. https://doi.org/https://doi.org/10.1002/hep.22375.

Veen, Maaike G. van, Anne M. Presanis, Stefano Conti, Maria Xiridou, Annemarie R. Stengaard, Martin C. Donoghoe, Ard I. van Sighem, Marianne A. van der Sande, and Daniela De Angelis. 2011. “National Estimate of HIV Prevalence in the Netherlands: Comparison and Applicability of Different Estimation Tools.” *AIDS* 25 (2). https://journals.lww.com/aidsonline/Fulltext/2011/01140/National_estimate_of_HIV_prevalence_in_the.14.aspx.

Wenz, Benjamin, Stine Nielsen, Martyna Gassowski, Claudia Santos-Hövener, Wei Cai, R. Stefan Ross, Claus-Thomas Bock, et al. 2016. “High Variability of HIV and HCV Seroprevalence and Risk Behaviours Among People Who Inject Drugs: Results from a Cross-Sectional Study Using Respondent-Driven Sampling in Eight German Cities (2011–14).” *BMC Public Health* 16 (1): 927. https://doi.org/10.1186/s12889-016-3545-4.

# Estimating the prevalence of chronic hepatitis C infection (CHC) in Greece using Bayesian multiparameter evidence synthesis

22/05/2022

## Table of Contents

Introduction ..... 2
Methods ..... 2
Prevalence of ex-PWID ..... 3
Prevalence of recent PWID ..... 3
Prevalence of CHC among recent PWID ..... 4
Prevalence of CHC among ex-PWID ..... 4
Prevalence of CHC among non-PWID ..... 4
Results ..... 6
Tables and Figures ..... 7
Appendix ..... 11
Fit of the multi-state Markov model ..... 11
Stan code for Bayesian multiparameter evidence synthesis ..... 12
Multi-state Markov model ..... 17
References ..... 25

# INTRODUCTION

Patients with chronic hepatitis C (HCV) infection often remain asymptomatic for decades and even for life, but a proportion of them develop active chronic hepatitis, which is a progressive disease (Thein et al. 2008). Because of the largely asymptomatic nature of HCV infection, notification data reflect national screening and testing practices and do not give accurate insights into the prevalence of HCV infection. Although there are studies that have estimated the prevalence of HCV antibodies (anti-HCV) in specific groups such as the general population, first-time blood donors, or people who inject drugs (PWID) at drug treatment centers, those alone cannot be combined to produce national estimates unless some additional information regarding the composition of each HCV risk group and its prevalence in the population are known (Sweeting et al. 2008; McDonald et al. 2014; Tan et al. 2018).

# METHODS

Bayesian multiparameter evidence synthesis (MPES) has been a popular approach to formally estimate anti-HCV or HIV prevalence (Sweeting et al. 2008; McDonald et al. 2014; Tan et al. 2018; Presanis et al. 2010; Veen et al. 2011). In this project, we extend this method to estimating the CHC prevalence, with the population (15-79 years) split into three main non-overlapping risk groups, i.e., recent PWID (those who have injected in the last year), ex-PWID, and non-PWID. A unified model is assumed including parameters associated with the CHC prevalence of recent, ex-PWID, and non-PWID (denoted by $\pi_{rec}$, $\pi_{ex}$, and $\pi_{non}$, respectively) and the prevalence of these risk groups in the population (denoted by $\rho_{rec}$, $\rho_{ex}$, and $\rho_{non}$, respectively; $\rho_{rec} + \rho_{ex} + \rho_{non} = 1$). To estimate the CHC prevalence in the whole population, $\pi$, we used the formula:

$$\pi = \pi_{rec}\rho_{rec} + \pi_{ex}\rho_{ex} + \pi_{non}\rho_{non} \quad (1)$$

Bayesian synthesis considers all available data, allowing for an evidence-based estimation of CHC prevalence in the population, with inherent uncertainty properly accounted for. Our aim is to estimate the CHC prevalence for the total (15-79) population of Greece in 2019 using sources of information over the last decade (2010-2019).

## Prevalence of ex-PWID

Data on the prevalence of ex-PWID are sparse and generally often unreliable. To overcome this limitation, we apply the method proposed by McDonald et al. (2014), which requires simulating a multi-state Markov model representing the non-PWID, recent PWID, and ex-PWID. In the multi-state Markov model, the simulation starts in 1950 and examines the population aged 15-79 years old. A certain number of 15-year-old individuals enter the simulation process each year. These numbers of 15-year-old individuals are obtained from the United Nations Statistic Division UNSD, being different for each country. The ageing of the population is also taken into account. Country-specific overall mortality data are retrieved from the Life Tables of the World Health Organization WHO. The duration of injecting career is also country-specific and obtained from the paper of Hines et al. (2020).

To estimate the number of ex-PWID, the model can be calibrated on the number of recent PWID provided in the EMCDDA barometer or the EMCDDA statistical bulletin (the code of the Markov model can be found in the Appendix). If there are no available data in the barometer, estimates from the systematic review of Grebely et al. (2019) could be used. Alternatively, if the national focal points suggest or provide different and updated or more accurate data for calibration purposes, we will consider their advice and adjust the model accordingly.

After applying the model for Greece, we compute the number (and the corresponding Confidence Interval - CI) of ex-PWID in 2019. The number of ex-PWID is then divided by the population size (15-79 years) in the same year, and a Normal distribution, constrained such that $\rho_{ex} \in (0,1)$, is assumed for $\rho_{ex}$. The standard deviation of this distribution is specified to approximately correspond to the respective CI, i.e. $\left(UB_{\rho_{ex}} - LB_{\rho_{ex}}\right)/(2 \times 1.96)$, where $UB_{\rho_{ex}}$ and $LB_{\rho_{ex}}$ denote the upper and lower limit of the CI.

## Prevalence of recent PWID

The number of recent PWID in Greece in 2019 is also projected by the multi-state Markov model described in the previous subsection. To estimate $\rho_{rec}$, the number of recent PWID is divided by the population size (15-79 years). Similar to the previous subsection, a Normal distribution, constrained such that $\rho_{rec} \in (0,1)$, is assumed for $\rho_{rec}$, with the standard deviation specified to correspond to the CI obtained from the multi-state Markov model.

## Prevalence of CHC among recent PWID

The prevalence (and the 95% CI) of CHC among recent PWID can be informed from studies in the EMCDDA statistical bulletin. If information on the CHC prevalence is available, the Binomial distribution in the model to inform $\pi_{rec}$ is used. If information on the HCV prevalence among recent PWID in the EMCDDA database is not available, the required information is obtained from the paper of Grebely et al. (2019). Currently, CHC prevalence data from the Alexandros program in Thessaloniki in 2019-2020 were used.

## Prevalence of CHC among ex-PWID

Information on CHC prevalence among ex-PWID (proportion of the population that is ex-PWID) (i.e., $\pi_{ex}$) is difficult to obtain directly. To overcome this, nationwide data on the HCV prevalence among ever users through the EMCDDA database can be utilized. If an estimate of $\pi_{ever}$ is available, $\pi_{ex}$ can be indirectly estimated since $\pi_{ever}$ is equal to a weighted average of $\pi_{rec}$ and $\pi_{ex}$, as shown by the following formula

$$\pi_{ever} = \frac{\rho_{rec}}{\rho_{rec} + \rho_{ex}}\pi_{rec} + \frac{\rho_{ex}}{\rho_{rec} + \rho_{ex}}\pi_{ex}, \quad (2)$$

or, equivalently,

$$\pi_{ex} = \left(\pi_{ever} - \frac{\rho_{rec}}{\rho_{rec} + \rho_{ex}}\pi_{rec}\right) \times \frac{\rho_{rec} + \rho_{ex}}{\rho_{ex}}, \quad (3)$$

Currently, CHC prevalence data from ever PWID in 2020, obtained through personal communication from EMCDDA, were used.

## Prevalence of CHC among non-PWID

To estimate the CHC prevalence among non-PWID in a country (after 2010), the ECDC database (European Centre for Disease Prevention and Control (ECDC) 2021) was used. The ECDC group has carefully and critically collected information from relevant studies across the EU/EEA region in cooperation with the focal points of each country. On the basis of the representativeness of each study and other factors (European Centre for Disease Prevention and Control (ECDC) 2016), a risk of bias score was assigned to each study, with higher values

indicating higher-quality studies. When higher-quality studies are available in a country ($\geq 4$), they are used to estimate the overall CHC prevalence in that country among non-PWID. If there are no higher-quality studies, general population estimates with a lower quality (<4) are pooled. If no general population prevalence estimates are available, data on the prevalence of HCV in pregnant women could be used as a prevalence measure. Finally, if data on pregnant women are lacking too, first-time blood donor studies could be an option. However, if the national focal point agrees or recommends, estimates from a neighboring country could be used instead, or we could use any other relevant information provided/suggested by the national focal point.

We directly use CHC prevalence data from studies in the general population, if available. However, as data often refer to the anti-HCV prevalence and not to the CHC prevalence, they could not be used directly. This issue can be partly addressed by noting that spontaneous viral clearance occurs in approximately one in four people with acute hepatitis C (Micallef, Kaldor, and Dore 2006). To account for the variability of the HCV clearance probabilities, we can use the result reported in Micallef, Kaldor, and Dore (2006), i.e., the proportion of HCV clearance (denoted by $\rho_{clear}$) is equal to 0.26 (95% CI 0.22–0.29). Thus, in the absence of treatment, estimates of the CHC prevalence among non-PWID ($\pi_{non}$) could be obtained using the formula $\pi_{non} = \pi(\text{anti-HCV})_{non}(1 - \rho_{clear})$, where $\pi(\text{anti-HCV})_{non}$ denotes the anti-HCV prevalence among non-PWID. However, apart from spontaneous clearance, some people may have been treated with direct acting antivirals (DAAs), with the sustained virologic response ($SVR$) estimated to be 96.7% (95% CI: 95.4% to 98.1%)(Lampertico et al. 2020). According to information provided by the national focal point, the total number of individuals having initiated treatment with DAAs up to 2019 in Greece is equal to 10,081. Denoting the proportion of non-PWID among individuals treated with DAAs by $\rho_{non|DAA}$, the CHC prevalence in non-PWID can be estimated by

$$\pi_{non} = \frac{N_{15,79}\rho_{non}\pi(\text{anti-HCV})_{non}(1 - \rho_{clear}) - N_{DAA}\rho_{non|DAA}SVR}{N_{15,79}\rho_{non}}. \quad (4)$$

However, the proportion of non-PWID among treated with DAAs, $\rho_{non|DAA}$, is not available in Greece. In this report, we make the assumption that $\rho_{non|DAA}$ is equal to the proportion of non-PWID among CHC-positive individuals, Pr(Non-PWID|CHC), as estimated by our model when

information on DAAs is completely ignored. That is, $\rho_{non|DAA} = \Pr(\text{Non-PWID}|\text{CHC})$ is estimated using recent anti-HCV data among ever PWID (Table 1), where the effect of DAAs is not present.

# RESULTS

The above-mentioned approach was used to estimate the prevalence of CHC in 2019 (considering ages between 15-79 years: 8,427,090).

The aggregated data used by our approach are briefly presented in Table 1. The multi-state Markov model was calibrated on the estimated prevalence of recent PWID reported in the systematic review of Grebely et al. (2019). In Greece, there was 1 study on non-PWID of high quality, which included only anti-HCV data. To estimate the CHC prevalence in the general population (primarily non-PWID), we used a fixed-effect meta-analytic approach, with the corresponding results presented in Table 2. The prevalence of recent and ex-PWID (proportion of the population that belongs to these groups) was low in Greece (about 0.06% and 0.16%, respectively) corresponding to 4,850 (95% CI: 4,470-5,280) recent PWID and 13,350 (95% CI: 12,700-14,090) ex-PWID in the population. However, the CHC prevalence in these groups was substantial (Table 2) being 39.82% and 25.35%, respectively. This translates to 1,931 (95% CI: 1,724-2,152) and 3,380 (95% CI: 2,689-4,110) recent and ex-PWID aged 15-79 living with CHC infection in Greece in 2019. Ignoring information on DAA uptake, the CHC prevalence in the general population was 0.59% (95% CI: 0.39%-0.84%), much lower than that of the high-risk groups (Table 2).

When DAAs are considered using Equation (4) assuming that $\rho_{non|DAA}$ is equal to $\Pr(\text{Non-PWID}|\text{CHC}) \approx 85.6\%$, the CHC prevalence in the general population reduces to 0.49% (95% CI: 0.3%-0.73%) (Table 3). Taking all pieces of information into account, the overall CHC prevalence in Greece in 2019 was equal to 0.55% (95% CI: 0.36%-0.8%), which corresponds to 46,260 (95% CI: 30,310-67,042) individuals aged 15-79 years with CHC infection (Table 3).

# TABLES AND FIGURES

*Table 1. Data (real) contributing to the estimation of CHC prevalence in Greece in 2019.*

| Parameter | Number (95%CI) | Numerator | Denominator | Notes | Year of study |
|---|---|---|---|---|---|
| $\rho_{rec}$ | 4,850 (4,470-5,280) | | | Method based on McDonald et al. | 2019 |
| $\rho_{ex}$ | 13,350 (12,700-14,090) | | | Method based on McDonald et al. | 2019 |
| $\pi_{rec}$ | | 438 | 1,100 | Alexandros program† | 2019-2020 |
| $\pi_{ever}$ | | 181 | 621 | EMCDDA database | 2020 |
| $\pi(\text{anti-HCV})_{ever}$ | | 413 | 671 | EMCDDA database | 2019 |
| $\pi(\text{anti-HCV})_{non}$ | | 26 | 3,367 | ECDC database (Touloumi et al.); Risk of bias=5†† | 2016 |

*Notes*: Although it looks counter-intuitive, **higher risk of bias score denotes a higher-quality study** (range from 0 to 6); † Βάνα Σύψα (Πανελλήνια Συνάντηση «AIDS, Ηπατίτιδες & Αναδυόμενα Νοσήματα) †† After excluding PWID from the study (data obtained through personal communication).

*Table 2. Results from the method assuming no heterogeneity in the estimation of the CHC prevalence in the general population from different studies (i.e., similar to a fixed-effect meta-analysis), ignoring information on DAAs.*

| Parameter | Posterior median | Posterior sd | LB (2.5%) | UB (97.5%) |
|---|---|---|---|---|
| $\rho_{rec}$ (%) | 0.06 | 0 | 0.05 | 0.06 |
| $\rho_{ex}$ (%) | 0.16 | 0 | 0.15 | 0.17 |
| $\pi_{rec}$ (%) | 39.82 | 1.47 | 36.99 | 42.75 |
| $\pi_{ex}$ (%) | 25.35 | 2.57 | 20.42 | 30.45 |
| $\pi_{non}$ (%) | 0.59 | 0.11 | 0.39 | 0.84 |
| $\pi$ (%) | 0.65 | 0.11 | 0.45 | 0.9 |
| Number with CHC | 54,704 | 9,676 | 38,206 | 75,889 |
| Pr(Ever PWID\|CHC) (%) | 9.73 | 1.86 | 6.84 | 14.09 |
| Pr(Non-PWID\|CHC) (%) | 90.27 | 1.86 | 85.91 | 93.16 |

*Notes*: $\rho_{rec}$, prevalence of recent PWID; $\rho_{ex}$, prevalence of ex-PWID; $\pi_{rec}$, CHC prevalence among recent PWID; $\pi_{ex}$, CHC prevalence among ex-PWID; $\pi_{non}$, CHC prevalence among non-PWID; $\pi$ overall CHC prevalence in Greece; LB, Lower Boundary; UP, Upper Boundary

*Table 3. Results from the method assuming no heterogeneity in the estimation of the CHC prevalence in the general population from different studies (i.e., similar to a fixed-effect meta-analysis), with the number of individuals treated with DAA taken into account.*

| Parameter | Posterior median | Posterior sd | LB (2.5%) | UB (97.5%) |
|---|---|---|---|---|
| $\rho_{rec}$ (%) | 0.06 | 0 | 0.05 | 0.06 |
| $\rho_{ex}$ (%) | 0.16 | 0 | 0.15 | 0.17 |
| $\pi_{rec}$ (%) | 39.85 | 1.47 | 36.94 | 42.74 |
| $\pi_{ex}$ (%) | 25.33 | 2.59 | 20.36 | 30.5 |
| $\pi_{non}$ (%) | 0.49 | 0.11 | 0.3 | 0.73 |
| $\pi$ (%) | 0.55 | 0.11 | 0.36 | 0.8 |
| Number with CHC | 46,260 | 9,513 | 30,310 | 67,042 |
| Pr(Ever PWID\|CHC) (%) | 11.49 | 2.57 | 7.75 | 17.72 |
| Pr(Non-PWID\|CHC) (%) | 88.51 | 2.57 | 82.28 | 92.25 |

*Notes*: $\rho_{rec}$, prevalence of recent PWID; $\rho_{ex}$, prevalence of ex-PWID; $\pi_{rec}$, CHC prevalence among recent PWID; $\pi_{ex}$, CHC prevalence among ex-PWID; $\pi_{non}$, CHC prevalence among non-PWID; $\pi$ overall CHC prevalence in Greece; LB, Lower Boundary; UP, Upper Boundary

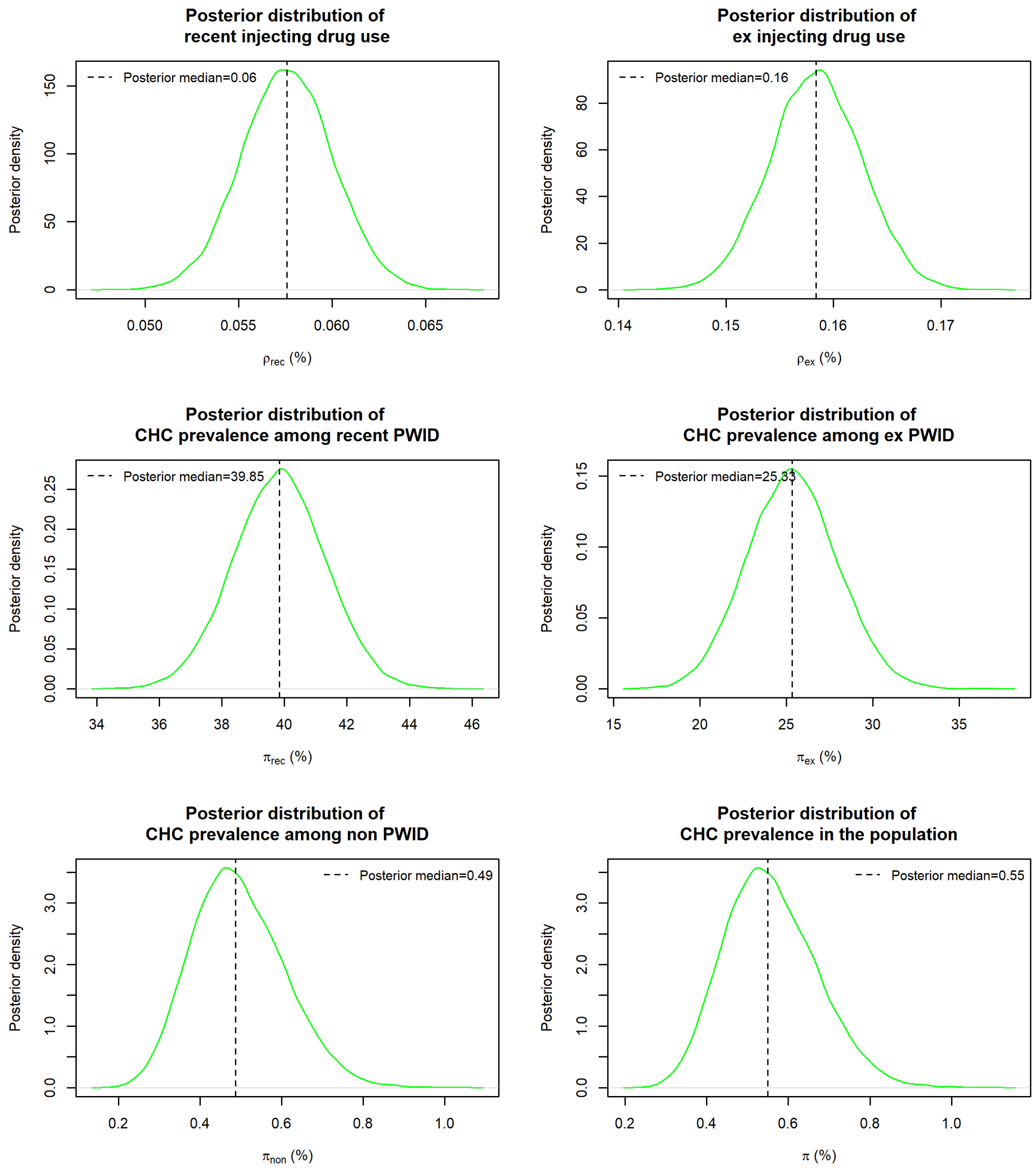


*Figure 1. Posterior distributions of the key model parameters using the approach that assumes no heterogeneity in the estimation of the CHC prevalence in the general population (primarily non-PWID) from different studies (i.e., similar to a fixed-effect meta-analysis), with the number of individuals treated with DAA taken into account.*

# APPENDIX

## Fit of the multi-state Markov model

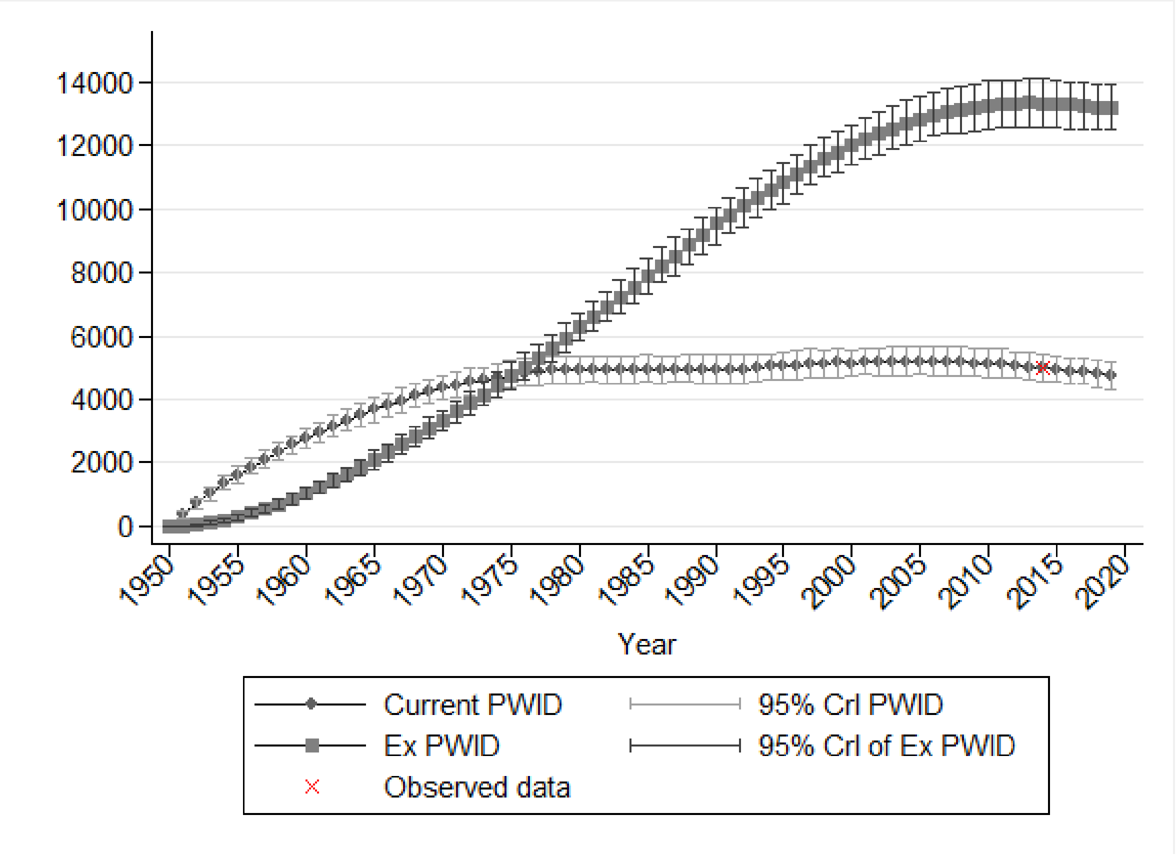


*Figure 2: Model predictions for the numbers of current and ex-PWID (people who inject drugs). The solid black line and shaded grey error bars show the median and 95% credible intervals (95% CrI) for the model projections. For comparison, asterisks indicate the observed country's data.*

## Stan code for Bayesian multiparameter evidence synthesis

```
data {
  int<lower=0> N1579; // Population of 15-79 in `Country`
  int<lower=1> Knon; // Number of studies in the ECDC data for `Country`
  int<lower=1> Kever; // Number of studies in the EMCDDA data for ever users in `Country`
  int<lower=1> Kcur; // Number of studies in the EMCDDA barometer (for HCV prevalence of PWID) in `Country`
  int<lower=1> NTotalDAA; // Number of individuals having received DAA up to 2019

  real p_cur_mean; // Prior mean for the prevalence of current use in `Country`
  real<lower=0> p_cur_sd; // Prior sd for the prevalence of current use in `Country`

  real p_ex_mean; // Prior mean for the prevalence of ex-use in `Country`
  real<lower=0> p_ex_sd; // Prior sd for the prevalence of ex-use in `Country`

  int<lower=0> Nst_CHC_cur[Kcur]; // Number of individuals in the study estimating CHC among recent PWID
  int<lower=0> Yst_CHC_cur[Kcur]; // Number of HCV+ in the study estimating CHC among recent PWID

  int<lower=0> Nst_CHC_ever[Kever]; // Number of individuals in the study estimating CHC among ever IDU
  int<lower=0> Yst_CHC_ever[Kever]; // Number of HCV+ in the study estimating CHC among ever PWID

  int<lower=0> Nst_hcv_ever[Kever]; // Number of individuals in the study estimating anti-HCV among ever IDU
```

```
  int<lower=0> Yst_hcv_ever[Kever]; // Number of HCV+ in the study estima
ting anti-HCV among ever PWID

  int<lower=0> Nst_hcv_non[Knon]; // Number of individuals in the study e
stimating anti-HCV among non PWID
  int<lower=0> Yst_hcv_non[Knon]; // Number of HCV+ in the study estimati
ng anti-HCV among non PWID

  vector<lower=0>[3] alpha; // parameter of the Diriclet prior

  real HCVclear_mean; // Prior mean for the HCV clearance probability
  real<lower=0> HCVclear_sd; // Prior sd for the HCV clearance probabilit
y

  real SVR_mean; // Prior mean for the SVR among non-PWID
  real<lower=0> SVR_sd; // Prior sd for the SVR among non-PWID
}

// Block defining the original parameters
parameters {
  // The parameters to be sampled
  simplex[3] rho; // Prevalence of the three risk groups
  real<lower=0,upper=1> CHCpi_cur; // P(CHC+|Cur)
  real<lower=0,upper=1> CHCpi_ex; // P(CHC+|Ex)
  real<lower=0,upper=1> CHCpi_non; // P(CHC+|Non)
  real<lower=0,upper=1> SVR; // SVR of DAAs among non-PWID
  real<lower=0,upper=1> pi_everPost; // P(anti-HCV|Ever)

  real<lower=0,upper=1-CHCpi_cur> HCVclear; // Probability of HCV clearan
ce; upper bound = 1-prevalence of chronic HCV
}
```

```
transformed parameters {
  // Change scales
  real<lower=0,upper=1> rho_ever;
  real<lower=0,upper=1> CHCpi_ever;
  real<lower=0,upper=1> pi_non;

  rho_ever = rho[1] + rho[2];
  CHCpi_ever = CHCpi_cur*rho[1]/rho_ever + CHCpi_ex*rho[2]/rho_ever;
  pi_non = CHCpi_non/(1-HCVclear);
}


// Binomial regression model
model {
  // Priors
  rho ~ dirichlet(alpha);

  //////////////////////////////
  // Likelihood contributions //
  //////////////////////////////

  // Probability of HCV clearance
  HCVclear ~ normal(HCVclear_mean,HCVclear_sd);

  // Prevalence of current use
  rho[1] ~ normal(p_cur_mean,p_cur_sd);

  // Prevalence of ex-use
  rho[2] ~ normal(p_ex_mean,p_ex_sd);

  // Prevalence of chronic HCV among current users
```

```
  Yst_CHC_cur ~ binomial(Nst_CHC_cur,CHCpi_cur);

  // Prevalence of HCV among ever users
  Yst_CHC_ever ~ binomial(Nst_CHC_ever,CHCpi_ever);
  Yst_hcv_ever ~ binomial(Nst_hcv_ever,pi_everPost);

  // HCV+ among non
  Yst_hcv_non ~ binomial(Nst_hcv_non,pi_non);

  // SVR of DAAs among non-PWID
  SVR ~ normal(SVR_mean,SVR_sd);
}


generated quantities {
  // Functions of parameters
  real<lower=0,upper=100> overalCHC;
  real logit_CHCpi_cur;
  real logit_CHCpi_ex;
  real logit_CHCpi_non;
  real logit_rho_cur;
  real logit_rho_ex;
  real logit_rho_non;
  real<lower=0,upper=1> pEverGivenCHC; // Ignoring DAA uptake
  real<lower=0,upper=1> pNonGivenCHC; // Ignoring DAA uptake
  real logit_HCVclear;
  real<lower=0> NumberCHC;// Ignoring DAA uptake
  real<lower=0,upper=1> CHCDAApi_non; // P(CHC+|Non) adjusted for DAA

  real<lower=0,upper=100> overalCHCPost;
  real<lower=0,upper=1> pEverGivenCHCPost;
  real<lower=0,upper=1> pNonGivenCHCPost;
```

```
  real<lower=0> NumberCHCPost;
  real<lower=0,upper=1> CHCpi_everPost;
  CHCpi_everPost = pi_everPost*(1-HCVclear);

  // Overall HCV prevalence, ignoring DAA uptake
  overalCHC = 100*(rho_ever*CHCpi_everPost + rho[3]*CHCpi_non);
  pEverGivenCHC = rho_ever*CHCpi_everPost/(overalCHC/100);
  pNonGivenCHC = CHCpi_non*(1-rho_ever)/(overalCHC/100);
  NumberCHC = round(overalCHC*N1579/100);

  // Taking DAAs into account
  CHCDAApi_non = ( N1579*rho[3]*(1-HCVclear)*pi_non - NTotalDAA*pNonGiven
CHC*SVR)/(N1579*rho[3]);
  overalCHCPost = 100*(rho[1]*CHCpi_cur + rho[2]*CHCpi_ex +  rho[3]*CHCDA
Api_non);
  pEverGivenCHCPost = CHCpi_ever*rho_ever/(overalCHCPost/100);
  pNonGivenCHCPost = CHCDAApi_non*(1-rho_ever)/(overalCHCPost/100);
  NumberCHCPost = round(overalCHCPost*N1579/100);

  logit_rho_cur = logit(rho[1]);
  logit_rho_ex = logit(rho[2]);
  logit_rho_non = logit(rho[3]);
  logit_CHCpi_cur = logit(CHCpi_cur);
  logit_CHCpi_ex = logit(CHCpi_ex);
  logit_CHCpi_non = logit(CHCDAApi_non);
  logit_HCVclear = logit(HCVclear);
}
```

## Multi-state Markov model

```
#include <stdio.h>
#include <stdlib.h>
#include <string.h>
#include <math.h>
#include <time.h>

#define loops 100
int populationPerYearAndAge[2021][66];
double deathRatePerAge[66];
int totalPopulationPerYear[2021];

FILE *F_DeathRate=fopen("`Country`-deathRates.txt","r");
FILE *F_Population=fopen("`Country`-population.txt","r");
FILE *out;

double pa_start = 0.00012;                  // Setting Variable
double pa_step  = 0.000002;                 // Setting Variable
double pa_stop  = 0.000123;                 // Setting Variable

double pa;
double pg = 1.0/11.7;   // Assumed average active injecting career of  11
.7 years
double pk = 0.004*12.0;     // Relapse rate of 0.004/month

struct people{
    int age;
    int state;
};

char filename[100];
struct people person[50000000];
```

```
long int totalPersons=0;

void setPopulationAge()
{
    for( int j=0; j<71; j++){
        for( int i=0; i<51; i++){
            fscanf(F_Population,"%d",&populationPerYearAndAge[1950+j][15+
i]);
        }
    }
}

void setDeathRate()
{
    for( int i=0; i<51; i++){
        fscanf(F_DeathRate,"%lf",&deathRatePerAge[15+i*5]);
        for( int j=0; j<5; j++){
            deathRatePerAge[15+i*5+j] = deathRatePerAge[15+i*5];
        }
    }
}

void getTotalPopulationPerAge()
{
    for( int j=0; j<71; j++){
        for( int i=0; i<51; i++){
            totalPopulationPerYear[1950+j] += populationPerYearAndAge[195
0+j][15+i];
        }
    }
}
```

```
void printTotalPopulationPerAge()
{
    printf("---- - ----------\n");
    printf("Year - Population\n");
    printf("---- - ----------\n");
    for( int i=1950; i<2021; i++){
        printf("%d - %d\n",i,totalPopulationPerYear[i]);
    }
}

void printDeathRatePerAge()
{
    printf("--- - -----\n");
    printf("Age - Rate\n");
    printf("--- - -----\n");
    for( int i=15; i<65; i++){
        printf("%d - %lf\n",i,deathRatePerAge[i]);
    }
}

void printTotalPersonPerState( int year)
{
    int count[4]= {0,0,0,0};
    for( int i=0; i<totalPersons; i++){
        count[person[i].state]++;
    }
    //printf("%d\t%ld\t%ld\t%ld\t%ld\n",year,count[0],count[1],count[2],c
ount[3]);
    if(year==2014)
        printf("%d\t%ld\t%ld\t%ld\t%ld\t%lf\n",year,count[0],count[1],cou
nt[2],count[3],pa);
    if( year>2009){
```

```
        fprintf(out, "%d\t%ld\t%ld\t%ld\t%ld\n",year,count[0],count[1],co
unt[2],count[3]);
    }
}

void initializePopulation()
{
    int cnt=0;
    for( int j=15; j<65; j++){
        for( int i=0; i<populationPerYearAndAge[1950][j]*100; i++){
            person[cnt].age=j;
            person[cnt].state=0;
            cnt++;
        }
    }
    totalPersons = cnt;
}

bool checkRate(double rate)
{
    if (rate <0.001){
        double k = rand()/(1.00+RAND_MAX);
        if(k<0.001){
            rate = rate*1000;
        }
        else{
            return false;
        }
    }
    double k = rand()/(1.00+RAND_MAX);
    if ( k < rate )
        return true;
```

```
    else
        return false;
}

void changeStatusAndAge()
{
    for( int i=0; i<totalPersons; i++){
        // change state
        double k = rand()/(1.00+RAND_MAX);
        if( person[i].state==0){
            if ( checkRate(pa) &&  person[i].age<40){
                person[i].state=1;
            }
        }
        else if( person[i].state==1){
            if ( k < pg ){
                person[i].state=2;
            }
        }
        else if( person[i].state==2){
            if ( k < pk ){
                person[i].state=1;
            }
        }
        // After their 49th year, all active PWID are assumed to cease in
jecting
        if(person[i].age>49 && person[i].state==1){
            person[i].state=2;
        }
        // After their 64th year, remove
        if(person[i].age>64){
            person[i].state=3;
```

```
        }
        // death rate depending on age and state
        k = rand()/(1.00+RAND_MAX);
        if( person[i].state==0){
            if ( k < deathRatePerAge[person[i].age] ){
                person[i].state=3;
            }
        }
        else if( person[i].state==1){
            if ( k < deathRatePerAge[person[i].age] * 13.0 ){
                person[i].state=3;
            }
        }
        else if( person[i].state==2){
            if ( k < deathRatePerAge[person[i].age] ){
                person[i].state=3;
            }
        }
        // increase age one year
        person[i].age++;
    }
}

void addNewPersons(int year)
{
    // add new person [15 years old and non PWID]
    int cnt=totalPersons;
    for( int i=0; i<populationPerYearAndAge[year+1][15]*100; i++){
// 100 instead of 1000 for the population to reduce execution time
        person[cnt].age=15;
        person[cnt].state=0;
        cnt++;
```

```
    }
    // increase the total number of population
    totalPersons = cnt;
}
int main()
{
    srand(time(NULL));

    setPopulationAge();
    setDeathRate();

    getTotalPopulationPerAge();
    pa = pa_start;
    while ( pa < pa_stop){

        snprintf(filename, 100, "result_%lf.txt",pa);
        out=fopen(filename,"w");

        for( int iter=0; iter<loops ;iter++){
            initializePopulation();
            for( int year=1950; year<2020; year++){
                printTotalPersonPerState(year);
                changeStatusAndAge();
                addNewPersons(year);
            }
        }

        fclose(out);
        pa = pa + pa_step;
    }
```

```
}
```

## REFERENCES

European Centre for Disease Prevention and Control (ECDC). 2016. “Systematic Review on Hepatitis b and c Prevalence in the EU/EEA.” https://www.ecdc.europa.eu/sites/default/files/media/en/publications/Publications/systematic-review-hepatitis-B-C-prevalence.pdf.

———. 2021. “Hepatitis c Prevalence Database.” https://www.ecdc.europa.eu/en/all-topics-z/hepatitis-c/tools/hepatitis-c-prevalence-database.

Grebely, Jason, Sarah Larney, Amy Peacock, Samantha Colledge, Janni Leung, Matthew Hickman, Peter Vickerman, et al. 2019. “Global, Regional, and Country-Level Estimates of Hepatitis c Infection Among People Who Have Recently Injected Drugs.” *Addiction* 114 (1): 150–66. https://doi.org/https://doi.org/10.1111/add.14393.

Hines, Lindsey A., Adam Trickey, Janni Leung, Sarah Larney, Amy Peacock, Louisa Degenhardt, Samantha Colledge, et al. 2020. “Associations Between National Development Indicators and the Age Profile of People Who Inject Drugs: Results from a Global Systematic Review and Meta-Analysis.” *The Lancet Global Health* 8 (1): e76–91. https://doi.org/10.1016/S2214-109X(19)30462-0.

Lampertico, Pietro, Jose A. Carrión, Michael Curry, Juan Turnes, Markus Cornberg, Francesco Negro, Ashley Brown, et al. 2020. “Real-World Effectiveness and Safety of Glecaprevir/Pibrentasvir for the Treatment of Patients with Chronic HCV Infection: A meta-Analysis.” *Journal of Hepatology* 72 (6): 1112–21. https://doi.org/10.1016/j.jhep.2020.01.025.

McDonald, Scott A., Rosmawati Mohamed, Maznah Dahlui, Herlianna Naning, and Adeeba Kamarulzaman. 2014. “Bridging the Data Gaps in the Epidemiology of Hepatitis c Virus Infection in Malaysia Using Multi-Parameter Evidence Synthesis.” *BMC Infectious Diseases* 14 (1): 564. https://doi.org/10.1186/s12879-014-0564-6.

Micallef, J. M., J. M. Kaldor, and G. J. Dore. 2006. “Spontaneous Viral Clearance Following Acute Hepatitis c Infection: A Systematic Review of Longitudinal Studies.” *Journal of Viral Hepatitis* 13 (1): 34–41. https://doi.org/https://doi.org/10.1111/j.1365-2893.2005.00651.x.

Presanis, Anne M., O. Noel Gill, Timothy R. Chadborn, Caterina Hill, Vivian Hope, Louise Logan, Brian D. Rice, Valerie C. Delpech, A. E. Ades, and Daniela De Angelis. 2010. “Insights into the Rise in HIV Infections, 2001 to 2008: A Bayesian Synthesis of Prevalence Evidence.” *AIDS* 24 (18). https://journals.lww.com/aidsonline/Fulltext/2010/11270/Insights_into_the_rise_in_HIV_infections,_2001_to.12.aspx.

Sweeting, M. J., D. De Angelis, M. Hickman, and A. E. Ades. 2008. “Estimating hepatitis C prevalence in England and Wales by synthesizing evidence from multiple data sources. Assessing data conflict and model fit.” *Biostatistics* 9 (4): 715–34. https://doi.org/10.1093/biostatistics/kxn004.

Tan, Sarah, Susanna Makela, Daliah Heller, Kevin Konty, Sharon Balter, Tian Zheng, and James H. Stark. 2018. “A Bayesian Evidence Synthesis Approach to Estimate Disease Prevalence in Hard-to-Reach Populations: Hepatitis c in New York City.” *Epidemics* 23: 96–109. https://doi.org/https://doi.org/10.1016/j.epidem.2018.01.002.

Thein, Hla-Hla, Qilong Yi, Gregory J. Dore, and Murray D. Krahn. 2008. “Estimation of Stage-Specific Fibrosis Progression Rates in Chronic Hepatitis c Virus Infection: A Meta-Analysis and Meta-Regression.” *Hepatology* 48 (2): 418–31. https://doi.org/https://doi.org/10.1002/hep.22375.

Veen, Maaike G. van, Anne M. Presanis, Stefano Conti, Maria Xiridou, Annemarie R. Stengaard, Martin C. Donoghoe, Ard I. van Sighem, Marianne A. van der Sande, and Daniela De Angelis. 2011. “National Estimate of HIV Prevalence in the Netherlands: Comparison and Applicability of Different Estimation Tools.” *AIDS* 25 (2). https://journals.lww.com/aidsonline/Fulltext/2011/01140/National_estimate_of_HIV_prevalence_in_the.14.aspx.

# Estimating the prevalence of chronic hepatitis C infection (CHC) in Hungary using Bayesian multiparameter evidence synthesis

15/03/2022

## Table of Contents


Introduction ........ 2

Methods ........ 2

Prevalence of ex-PWID ........ 3

Prevalence of recent PWID ........ 3

Prevalence of CHC among recent PWID ........ 4

Prevalence of CHC among ex-PWID ........ 4

Prevalence of CHC among non-PWID ........ 5

Sensitivity analysis including migrants from endemic countries ........ 6

Results ........ 6

Tables and Figures ........ 8

Appendix ........ 14

Fit of the multi-state Markov model ........ 14

Stan code for Bayesian multiparameter evidence synthesis ........ 15

Multi-state Markov model ........ 20

References ........ 28

## INTRODUCTION

Patients with chronic hepatitis C (HCV) infection often remain asymptomatic for decades and even for life, but a proportion of them develop active chronic hepatitis, which is a progressive disease (Thein et al. 2008). Because of the largely asymptomatic nature of HCV infection, notification data reflect national screening and testing practices and do not give accurate insights into the prevalence of HCV infection. Although there are studies that have estimated the prevalence of HCV antibodies (anti-HCV) in specific groups such as the general population, first-time blood donors, or people who inject drugs (PWID) at drug treatment centers, those alone cannot be combined to produce national estimates unless some additional information regarding the composition of each HCV risk group and its prevalence in the population are known (Sweeting et al. 2008; McDonald et al. 2014; Tan et al. 2018).

## METHODS

Bayesian multiparameter evidence synthesis (MPES) has been a popular approach to formally estimate anti-HCV or HIV prevalence (Sweeting et al. 2008; McDonald et al. 2014; Tan et al. 2018; Presanis et al. 2010; Veen et al. 2011). In this project, we extend this method to estimating the CHC prevalence, with the population (15-79 years) split into three main non-overlapping risk groups, i.e., recent PWID (those who have injected in the last year), ex-PWID, and non-PWID. A unified model is assumed including parameters associated with the CHC prevalence of recent, ex-PWID, and non-PWID (denoted by $\pi_{rec}$, $\pi_{ex}$, and $\pi_{non}$, respectively) and the prevalence of these risk groups in the population (denoted by $\rho_{rec}$, $\rho_{ex}$, and $\rho_{non}$, respectively; $\rho_{rec} + \rho_{ex} + \rho_{non} = 1$). To estimate the CHC prevalence in the whole population, $\pi$, we used the formula:

$$\pi = \pi_{rec}\rho_{rec} + \pi_{ex}\rho_{ex} + \pi_{non}\rho_{non} \quad (1)$$

Bayesian synthesis considers all available data, allowing for an evidence-based estimation of CHC prevalence in the population, with inherent uncertainty properly accounted for. Our aim is to estimate the CHC prevalence for the total (15-79) population of Hungary in 2019 using sources of information over the last decade (2010-2019).

## Prevalence of ex-PWID

Data on the prevalence of ex-PWID are sparse and generally often unreliable. To overcome this limitation, we apply the method proposed by McDonald et al. (2014), which requires simulating a multi-state Markov model representing the non-PWID, recent PWID, and ex-PWID. In the multi-state Markov model, the simulation starts in 1950 and examines the population aged 15-79 years old. A certain number of 15-year-old individuals enter the simulation process each year. These numbers of 15-year-old individuals are obtained from the United Nations Statistic Division UNSD, being different for each country. The ageing of the population is also taken into account. Country-specific overall mortality data are retrieved from the Life Tables of the World Health Organization WHO. The duration of injecting career is also country-specific and obtained from the paper of Hines et al. (2020).

To estimate the number of ex-PWID, the model can be calibrated on the number of recent PWID provided in the EMCDDA barometer or the EMCDDA statistical bulletin (the code of the Markov model can be found in the Appendix). If there are no available data in the barometer, estimates from the systematic review of Grebely et al. (2019) could be used. Alternatively, if the national focal points suggest or provide different and updated or more accurate data for calibration purposes, we will consider their advice and adjust the model accordingly.

After applying the model for Hungary, we compute the number (and the corresponding Confidence Interval - CI) of ex-PWID in 2019. The number of ex-PWID is then divided by the population size (15-79 years) in the same year, and a Normal distribution, constrained such that $\rho_{ex} \in (0,1)$, is assumed for $\rho_{ex}$. The standard deviation of this distribution is specified to approximately correspond to the respective CI, i.e. $\left(UB_{\rho_{ex}} - LB_{\rho_{ex}}\right)/(2 \times 1.96)$, where $UB_{\rho_{ex}}$ and $LB_{\rho_{ex}}$ denote the upper and lower limit of the CI.

## Prevalence of recent PWID

The number of recent PWID in Hungary in 2019 is also projected by the multi-state Markov model described in the previous subsection. To estimate $\rho_{rec}$, the number of recent PWID is divided by the population size (15-79 years). Similar to the previous subsection, a Normal distribution, constrained such that $\rho_{rec} \in (0,1)$, is assumed for $\rho_{rec}$, with the standard deviation specified to correspond to the CI obtained from the multi-state Markov model.

## Prevalence of CHC among recent PWID

The prevalence (and the 95% CI) of CHC among recent PWID can be informed from studies in the EMCDDA statistical bulletin. If information on the CHC prevalence is available, the Binomial distribution in the model to inform $\pi_{rec}$ was used. However, as these EMCDDA data typically refer to the anti-HCV prevalence and not to the CHC prevalence, they could not be used directly. This issue is addressed by noting that spontaneous viral clearance occurs in approximately one in four people with acute hepatitis C (Micallef, Kaldor, and Dore 2006). To account for the variability of the HCV clearance probabilities, we used the result reported in Micallef, Kaldor, and Dore (2006), i.e., the proportion of HCV clearance (denoted by $\rho_{clear}$) is equal to 0.26 (95% CI 0.22–0.29). Thus, estimates of the CHC prevalence among recent PWID ($\pi_{rec}$) can be obtained using the formula $\pi_{rec} = \pi(\text{anti-HCV})_{rec}(1 - \rho_{clear})$, where $\pi(\text{anti-HCV})_{rec}$ denotes the anti-HCV prevalence among recent PWID.

Apart from spontaneous clearance, some people may have been treated with direct acting antivirals (DAAs). Since treatment data may be weak and unstandardised or unavailable for most EU/EEA countries, we have not formally taken treatment into account.

If information on the HCV prevalence (CHC or anti-HCV) among recent PWID in the EMCDDA database is not available, the required information is obtained from the paper of Grebely et al. (2019). Currently, CHC prevalence data from the paper of Grebely et al. (2019) were used. If the national focal point recommends updated formal estimates, the model input could be adjusted accordingly.

## Prevalence of CHC among ex-PWID

Information on CHC prevalence among ex-PWID (i.e., $\pi_{ex}$) is difficult to obtain directly. To overcome this, nationwide data on the HCV prevalence among ever users through the EMCDDA database was utilized. However, if these data refer to the anti-HCV prevalence, they are adjusted according to the procedure described in the previous subsection, i.e. $\pi_{ever} = \pi(\text{anti-HCV})_{ever}(1 - \rho_{clear})$, where $\pi(\text{anti-HCV})_{ever}$ denotes the anti-HCV prevalence among ever PWID. In any case, if an estimate of $\pi_{ever}$ is available, $\pi_{ex}$ can be indirectly estimated since $\pi_{ever}$ is equal to a weighted average of $\pi_{rec}$ and $\pi_{ex}$, as shown by the following formula

$$\pi_{ever} = \frac{\rho_{rec}}{\rho_{rec} + \rho_{ex}} \pi_{rec} + \frac{\rho_{ex}}{\rho_{rec} + \rho_{ex}} \pi_{ex}, \quad (2)$$

or, equivalently,

$$\pi_{ex} = \left( \pi_{ever} - \frac{\rho_{rec}}{\rho_{rec} + \rho_{ex}} \pi_{rec} \right) \times \frac{\rho_{rec} + \rho_{ex}}{\rho_{ex}}, \quad (3)$$

Currently, anti-HCV prevalence data from ever PWID in Budapest and Bács-Kiskun in 2019, available in the EMCDDA statistical bulletin, were used. In any case, the model could be updated with any other relevant study/information suggested by the national focal point.

## Prevalence of CHC among non-PWID

To estimate the CHC prevalence among non-PWID in a country (after 2010), the ECDC database (European Centre for Disease Prevention and Control (ECDC) 2021) was used. The ECDC group has carefully and critically collected information from relevant studies across the EU/EEA region in cooperation with the focal points of each country. On the basis of the representativeness of each study and other factors (European Centre for Disease Prevention and Control (ECDC) 2016), a risk of bias score was assigned to each study, with higher values indicating higher-quality studies. When higher-quality studies are available in a country (≥ 4), they are used to estimate the overall CHC prevalence in that country among non-PWID. If there are no higher-quality studies, general population estimates with a lower quality (<4) are pooled. If no general population prevalence estimates are available, data on the prevalence of HCV in pregnant women could be used as a prevalence measure. Finally, if data on pregnant women are lacking too, first-time blood donor studies could be an option. However, if the national focal point agrees or recommends, estimates from a neighboring country could be used instead, or we could use any other relevant information provided/suggested by the national focal point.

We directly use CHC prevalence data from studies in the general population, if available. If the data in the general population refer to anti-HCV prevalence (without any data on the viremic population), we adjust the estimates to get CHC prevalence based on the spontaneous HCV clearance estimate of 26%, described in the previous subsection.

As $\rho_{rec}$ and $\rho_{ex}$ are likely to be low, $\pi_{non}$ will be the most critical factor for the estimation of the overall CHC prevalence in the population. To adjust for potential heterogeneity between the CHC prevalence estimates from different studies in the non-PWID population in a country, as a sensitivity analysis, we also apply a random-effect meta-analytic approach to pool the CHC prevalence across studies on non-PWID (Lin and Chu 2020). The random-effect approach is carried out only when at least 3 studies are available in the general population of a certain country, and the estimates are compared with the corresponding estimates from the approach assuming no heterogeneity (similar to a fixed-effect meta-analysis). If the estimates are inconsistent, possible explanations are investigated and discussed. If there is substantial heterogeneity between studies, it would help more to explore the causes; for example, following national focal point suggestions, some studies could be excluded to reduce heterogeneity.

## Sensitivity analysis including migrants from endemic countries

A sensitivity analysis including data for the viremic population among migrants based on the report of the ECDC group (European Centre for Disease Prevention and Control 2016) was carried out. Migrants from endemic countries represent 4% of the adult population in Hungary (Table 8 in European Centre for Disease Prevention and Control (2016)), with the respective CHC prevalence being equal to $\pi_{mig} = 1.9\%$ (Table 9 in European Centre for Disease Prevention and Control (2016)). The overall CHC prevalence is now estimated by

$$\pi = \pi_{rec}\rho_{rec} + \pi_{ex}\rho_{ex} + \pi_{mig}\rho_{mig} + \pi_{non}\rho_{non},$$

where $\rho_{rec} + \rho_{ex} + \rho_{mig} + \rho_{non} = 1$.

# RESULTS

The above-mentioned approach was used to estimate the prevalence of CHC in 2019 (considering ages between 15-79 years: 7,917,984).

The aggregated data used by our approach are briefly presented in Table 1. The multi-state Markov model was calibrated on the estimated prevalence of recent PWID reported in the EMCDDA barometer. In Hungary, there were 7 studies on first-time blood donors, which included only anti-HCV data. To estimate the CHC prevalence in the general population (primarily non-PWID), we used a fixed-effect meta-analytic approach, with the corresponding

results presented in Table 2. The prevalence of recent and ex-PWID was low in Hungary (about 0.08% and 0.23%, respectively) corresponding to 6,450 (95% CI: 5,910-6,970) recent PWID and 17,930 (95% CI: 17,100-18,890) ex-PWID in the population. However, the CHC prevalence in these groups was substantial (Table 2) being 34.99% and 35.87%, respectively. This translates to 2,251 (95% CI: 1,473-3,078) and 6,437 (95% CI: 4,499-8,392) recent and ex-PWID aged 15-79 living with CHC infection in Hungary in 2019. The CHC prevalence in the general population was 0.12% (95% CI: 0.11%-0.13%), much lower than that of the high-risk groups. Taking all pieces of information into account, the overall CHC prevalence in Hungary in 2019 was equal to 0.23% (95% CI: 0.2%-0.25%), which corresponds to 17,984 (95% CI: 15,962-20,101) individuals aged 15-79 years with CHC infection. The corresponding results under a random-effect meta-analysis for the studies in the general population were very similar and are provided in Table 3. This implies that the heterogeneity between the estimates of the studies in the general population is low, which is also reflected in the small estimate of the between-study variance (Table 3).

The results from our model including migrants from endemic countries as a separate group are presented in Table 4. However, this analysis comes with possible limitations; that is, including migrants as a separate group is valid only if migrants do not overlap with the remaining groups (recent PWID, ex-PWID, and non-PWID). Thus, if migrants do participate proportionally in the study(ies) in the general population, this analysis may result in biased overall CHC estimates (most probably in higher CHC prevalence estimates). However, if the national focal points consider that including migrants as a separate group is valid, we could consider results in Table 4 as the main analysis.

# TABLES AND FIGURES

*Table 1. Data (real) contributing to the estimation of CHC prevalence in Hungary in 2019.*

| Parameter | Estimate (95%CI) | Number (95%CI) | Numerator | Denominator | Notes | Year of study |
|---|---|---|---|---|---|---|
| $\rho_{rec}$ | | 6,450 (5,910-6,970) | | | Method based on McDonald et al. | 2019 |
| $\rho_{ex}$ | | 17,930 (17,100-18,890) | | | Method based on McDonald et al. | 2019 |
| $\pi_{rec}$ | 35% (22.9%-47.2%) | | | | Grebely et al. | 2012-2015 |
| $\pi(\text{anti-HCV})_{ever}$ | | | 49 | 102 | EMCDDA database | 2019 |
| $\pi(\text{anti-HCV})_{non}$ | | | 113 | 51,154 | ECDC database (NA et al.); Risk of bias=NA | 2010 |
| $\pi(\text{anti-HCV})_{non}$ | | | 91 | 56,632 | ECDC database (NA et al.); Risk of bias=NA | 2011 |

| Parameter | Estimate (95%CI) | Number (95%CI) | Numerator | Denominator | Notes | Year of study |
|---|---|---|---|---|---|---|
| $\pi(\text{anti-HCV})_{non}$ | | | 94 | 44,785 | ECDC database (NA et al.); Risk of bias=NA | 2012 |
| $\pi(\text{anti-HCV})_{non}$ | | | 86 | 53,978 | ECDC database (NA et al.); Risk of bias=NA | 2013 |
| $\pi(\text{anti-HCV})_{non}$ | | | 70 | 53,597 | ECDC database (NA et al.); Risk of bias=NA | 2014 |
| $\pi(\text{anti-HCV})_{non}$ | | | 57 | 51,822 | ECDC database (NA et al.); Risk of bias=NA | 2015 |
| $\pi(\text{anti-HCV})_{non}$ | | | 67 | 51,863 | ECDC database (NA et al.); Risk of bias=NA | 2016 |

*Notes*: Higher risk of bias score denotes a higher-quality study (range from 0 to 6);

*Table 2. Results from the method assuming no heterogeneity in the estimation of the CHC prevalence in the general population from different studies (i.e., similar to a fixed-effect meta-analysis).*

| Parameter | Posterior median | Posterior sd | LB (2.5%) | UB (97.5%) |
|---|---|---|---|---|
| $\rho_{rec}$ (%) | 0.08 | 0 | 0.07 | 0.09 |
| $\rho_{ex}$ (%) | 0.23 | 0.01 | 0.22 | 0.24 |
| $\pi_{rec}$ (%) | 34.99 | 6.18 | 22.89 | 47.12 |
| $\pi_{ex}$ (%) | 35.87 | 5.53 | 25.15 | 46.65 |
| $\pi_{non}$ (%) | 0.12 | 0.01 | 0.11 | 0.13 |
| $\pi$ (%) | 0.23 | 0.01 | 0.2 | 0.25 |
| Number with CHC | 17,984 | 1,064 | 15,962 | 20,101 |
| Pr(Ever PWID\|CHC) (%) | 48.31 | 2.78 | 42.51 | 53.36 |
| Pr(Non-PWID\|CHC) (%) | 51.69 | 2.78 | 46.64 | 57.49 |

*Notes*: $\rho_{rec}$, prevalence of recent PWID; $\rho_{ex}$, prevalence of ex-PWID; $\pi_{rec}$, CHC prevalence among recent PWID; $\pi_{ex}$, CHC prevalence among ex-PWID; $\pi_{non}$, CHC prevalence among non-PWID; $\pi$ overall CHC prevalence in Hungary; LB, Lower Boundary; UP, Upper Boundary

*Table 3. Results from the method assuming heterogeneity in the estimation of the CHC prevalence in the general population from different studies (i.e., similar to a random-effect meta-analysis).*

| Parameter | Posterior median | Posterior sd | LB (2.5%) | UB (97.5%) |
|---|---|---|---|---|
| $\rho_{rec}$ (%) | 0.08 | 0 | 0.07 | 0.09 |
| $\rho_{ex}$ (%) | 0.23 | 0.01 | 0.22 | 0.24 |
| $\pi_{rec}$ (%) | 34.89 | 6.19 | 22.65 | 47.05 |
| $\pi_{ex}$ (%) | 35.83 | 5.55 | 25.09 | 46.92 |
| $\pi_{non}$ (%) | 0.12 | 0.02 | 0.08 | 0.16 |
| $\pi$ (%) | 0.22 | 0.02 | 0.18 | 0.27 |
| Number with CHC | 17,782 | 1,807 | 14,606 | 21,694 |
| Pr(Ever PWID\|CHC) (%) | 48.78 | 4.78 | 39.35 | 58.29 |
| Pr(Non-PWID\|CHC) (%) | 51.22 | 4.78 | 41.71 | 60.65 |
| Between-study variance | 0.11 | 0.22 | 0.02 | 0.74 |

*Notes*: $\rho_{rec}$, prevalence of recent PWID; $\rho_{ex}$, prevalence of ex-PWID; $\pi_{rec}$, CHC prevalence among recent PWID; $\pi_{ex}$, CHC prevalence among ex-PWID; $\pi_{non}$, CHC prevalence among non-PWID; $\pi$ overall CHC prevalence in Hungary; LB, Lower Boundary; UP, Upper Boundary

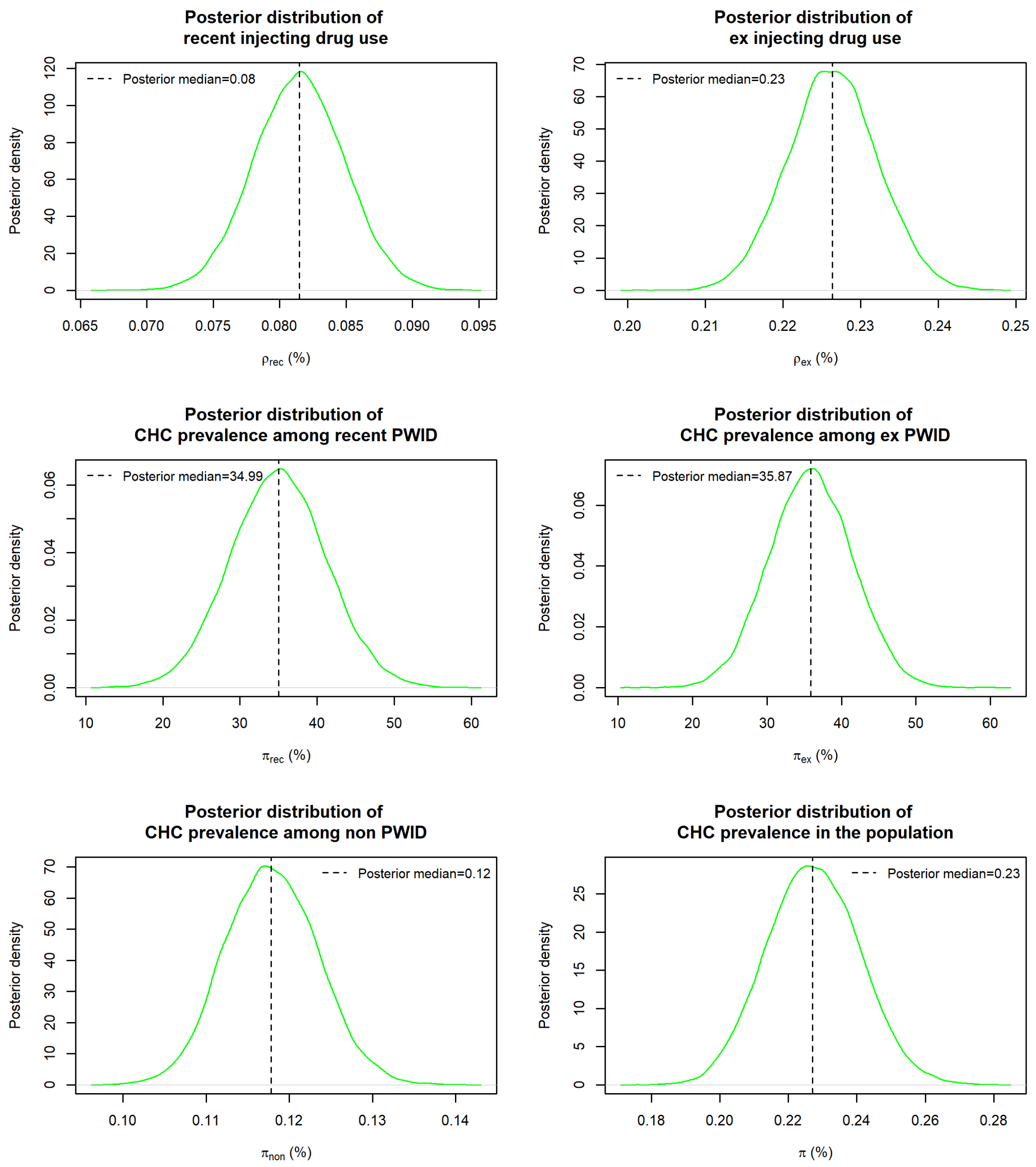


*Figure 1. Posterior distributions of the key model parameters using the approach that assumes no heterogeneity in the estimation of the CHC prevalence in the general population (primarily non-PWID) from different studies (i.e., similar to a fixed-effect meta-analysis).*

*Table 4. Results from the method assuming no heterogeneity in the estimation of the CHC prevalence in the general population (primarily non-PWID) from different studies (i.e., similar to a fixed-effect meta-analysis) including migrants from endemic countries as a separate group.*

| Parameter | Posterior median | Posterior sd | LB (2.5%) | UB (97.5%) |
|---|---|---|---|---|
| $\rho_{rec}$ (%) | 0.08 | 0 | 0.07 | 0.09 |
| $\rho_{ex}$ (%) | 0.23 | 0.01 | 0.22 | 0.24 |
| $\rho_{mig}$ (%) | 4 | 0 | 4 | 4 |
| $\pi_{rec}$ (%) | 34.98 | 6.21 | 22.78 | 47.21 |
| $\pi_{ex}$ (%) | 35.83 | 5.61 | 24.96 | 46.99 |
| $\pi_{mig}$ (%) | 1.9 | 0.22 | 1.47 | 2.34 |
| $\pi_{non}$ (%) | 0.12 | 0.01 | 0.11 | 0.13 |
| $\pi$ (%) | 0.3 | 0.02 | 0.27 | 0.33 |
| Number with CHC | 23,640 | 1,289 | 21,133 | 26,185 |
| Pr(Ever PWID\|CHC) (%) | 36.73 | 2.73 | 31.17 | 41.95 |
| Pr(Mig\|CHC) (%) | 25.48 | 2.49 | 20.59 | 30.28 |
| Pr(Non-PWID\|CHC) (%) | 37.77 | 2.1 | 33.98 | 42.19 |

*Notes*: $\rho_{rec}$, prevalence of recent PWID; $\rho_{ex}$, prevalence of ex-PWID; $\rho_{mig}$, prevalence of migrants from endemic countries; $\pi_{rec}$, CHC prevalence among recent PWID; $\pi_{ex}$, CHC prevalence among ex-PWID; $\pi_{mig}$, CHC prevalence among migrants from endemic countries; $\pi_{non}$, CHC prevalence among non-PWID; $\pi$ overall CHC prevalence in Hungary; LB, Lower Boundary; UP, Upper Boundary

# APPENDIX

## Fit of the multi-state Markov model

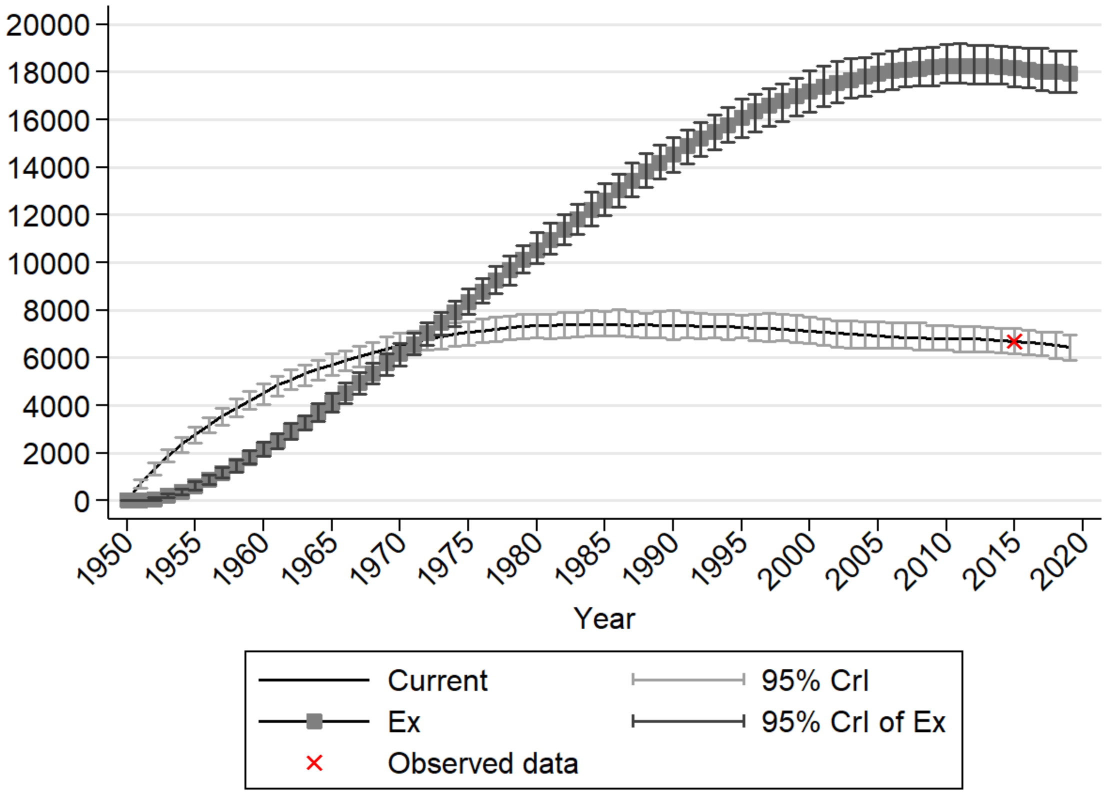


*Figure 2: Model predictions for the numbers of current and ex-PWID (people who inject drugs). The solid black line and shaded grey error bars show the median and 95% credible intervals (95% CrI) for the model projections. For comparison, asterisks indicate the observed country's data.*

## Stan code for Bayesian multiparameter evidence synthesis

```
data {
  int<lower=0> N1580; // Population of 15-64 in `Country`
  int<lower=1> Knon; // Number of studies in the ECDC data for `Country`
  int<lower=1> Kever; // Number of studies in the EMCDDA data for ever users in `Country`
  int<lower=1> Kcur; // Number of studies in the EMCDDA barometer (for HCV prevalence of PWID) in `Country`

  real p_cur_mean; // Prior mean for the prevalence of current use in `Country`
  real<lower=0> p_cur_sd; // Prior sd for the prevalence of current use in `Country`

  real p_ex_mean; // Prior mean for the prevalence of ex-use in `Country`
  real<lower=0> p_ex_sd; // Prior sd for the prevalence of ex-use in `Country`

  //int<lower=0> Nst_hcv_cur[Kcur]; // Number of individuals in the study estimating anti-HCV among recent PWID
  //int<lower=0> Yst_hcv_cur[Kcur]; // Number of HCV+ in the study estimating anti-HCV among recent PWID
  real p_CHC_cur_mean; // Prior mean for the CHC prevalence among recent PWID in `Country`
  real<lower=0> p_CHC_cur_sd; // Prior sd for the CHC prevalence among recent PWID in `Country`

  int<lower=0> Nst_hcv_ever[Kever]; // Number of individuals in the study estimating HCV among ever IDU
  int<lower=0> Yst_hcv_ever[Kever]; // Number of HCV+ in the study estimating HCV among ever PWID
```

```
  int<lower=0> Nst_hcv_non[Knon]; // Number of individuals in the study e
stimating anti-HCV among non PWID
  int<lower=0> Yst_hcv_non[Knon]; // Number of HCV+ in the study estimati
ng anti-HCV among non PWID

  vector<lower=0>[3] alpha; // parameter of the Diriclet prior

  real HCVclear_mean; // Prior mean for the HCV clearance probability
  real<lower=0> HCVclear_sd; // Prior sd for the HCV clearance probabilit
y
}

// Block defining the original parameters
parameters {
  // The parameters to be sampled
  simplex[3] rho; // Prevalence of the three risk groups
  real<lower=0,upper=1> CHCpi_cur; // P(CHC+|Cur)
  real<lower=0,upper=1> CHCpi_ex; // P(CHC+|Ex)
  real<lower=0,upper=1> CHCpi_non; // P(CHC+|Non)

  real<lower=0,upper=1-CHCpi_cur> HCVclear; // Probability of HCV clearan
ce; upper bound = 1-prevalence of chronic HCV
}


transformed parameters {
  // Change scales
  real<lower=0,upper=1> rho_ever;
  real<lower=0,upper=1> CHCpi_ever;
  real<lower=0,upper=1> pi_ever;
  real<lower=0,upper=1> pi_non;
```

```
  rho_ever = rho[1] + rho[2];
  CHCpi_ever = CHCpi_cur*rho[1]/rho_ever + CHCpi_ex*rho[2]/rho_ever;
  pi_ever = CHCpi_ever/(1-HCVclear);
  pi_non = CHCpi_non/(1-HCVclear);
}


// Binomial regression model
model {
  // Priors
  rho ~ dirichlet(alpha);

  //////////////////////////////
  // Likelihood contributions //
  //////////////////////////////

  // Probability of HCV clearance
  HCVclear ~ normal(HCVclear_mean,HCVclear_sd);

  // Prevalence of current use
  rho[1] ~ normal(p_cur_mean,p_cur_sd);

  // Prevalence of ex-use
  rho[2] ~ normal(p_ex_mean,p_ex_sd);

  // Prevalence of chronic HCV among current users
  CHCpi_cur ~ normal(p_CHC_cur_mean,p_CHC_cur_sd);
  //Yst_hcv_cur ~ binomial(Nst_hcv_cur,pi_cur);

  // Prevalence of HCV among ever users
  Yst_hcv_ever ~ binomial(Nst_hcv_ever,pi_ever);
```

```
  // HCV+ among non
  Yst_hcv_non ~ binomial(Nst_hcv_non,pi_non);
}


generated quantities {
  // Functions of parameters
  real<lower=0,upper=100> overalCHC;
  real logit_CHCpi_cur;
  real logit_CHCpi_ex;
  real logit_CHCpi_non;
  real logit_rho_cur;
  real logit_rho_ex;
  real logit_rho_non;
  real<lower=0,upper=1> pEverGivenCHC;
  real<lower=0,upper=1> pNonGivenCHC;
  real logit_HCVclear;
  real<lower=0> NumberCHC;

  // Overall HCV prevalence
  overalCHC = 100*(rho[1]*CHCpi_cur + rho[2]*CHCpi_ex +  rho[3]*CHCpi_non
);
  pEverGivenCHC = CHCpi_ever*rho_ever/(overalCHC/100);
  pNonGivenCHC = CHCpi_non*(1-rho_ever)/(overalCHC/100);
  NumberCHC = round(overalCHC*N1580/100);

  logit_rho_cur = logit(rho[1]);
  logit_rho_ex = logit(rho[2]);
  logit_rho_non = logit(rho[3]);
  logit_CHCpi_cur = logit(CHCpi_cur);
  logit_CHCpi_ex = logit(CHCpi_ex);
  logit_CHCpi_non = logit(CHCpi_non);
```

```
  logit_HCVclear = logit(HCVclear);
}
```

## Multi-state Markov model

```
#include <stdio.h>
#include <stdlib.h>
#include <string.h>
#include <math.h>
#include <time.h>

#define loops 100
int populationPerYearAndAge[2021][66];
double deathRatePerAge[66];
int totalPopulationPerYear[2021];

FILE *F_DeathRate=fopen("greece-deathRates.txt","r");
FILE *F_Population=fopen("greece-population.txt","r");
FILE *out;

double pa_start = 0.00012;                    // Setting Variable
double pa_step  = 0.000002;                   // Setting Variable
double pa_stop  = 0.000123;                   // Setting Variable

double pa;
double pg = 1.0/11.7;    // Assumed average active injecting career of  11
.7 years
double pk = 0.004*12.0;      // Relapse rate of 0.004/month

struct people{
    int age;
    int state;
};

char filename[100];
struct people person[50000000];
```

```
long int totalPersons=0;

void setPopulationAge()
{
    for( int j=0; j<71; j++){
        for( int i=0; i<51; i++){
            fscanf(F_Population,"%d",&populationPerYearAndAge[1950+j][15+
i]);
        }
    }
}

void setDeathRate()
{
    for( int i=0; i<51; i++){
        fscanf(F_DeathRate,"%lf",&deathRatePerAge[15+i*5]);
        for( int j=0; j<5; j++){
            deathRatePerAge[15+i*5+j] = deathRatePerAge[15+i*5];
        }
    }
}

void getTotalPopulationPerAge()
{
    for( int j=0; j<71; j++){
        for( int i=0; i<51; i++){
            totalPopulationPerYear[1950+j] += populationPerYearAndAge[195
0+j][15+i];
        }
    }
}
```

```
void printTotalPopulationPerAge()
{
    printf("---- - ----------\n");
    printf("Year - Population\n");
    printf("---- - ----------\n");
    for( int i=1950; i<2021; i++){
        printf("%d - %d\n",i,totalPopulationPerYear[i]);
    }
}

void printDeathRatePerAge()
{
    printf("--- - -----\n");
    printf("Age - Rate\n");
    printf("--- - -----\n");
    for( int i=15; i<65; i++){
        printf("%d - %lf\n",i,deathRatePerAge[i]);
    }
}

void printTotalPersonPerState( int year)
{
    int count[4]= {0,0,0,0};
    for( int i=0; i<totalPersons; i++){
        count[person[i].state]++;
    }
    //printf("%d\t%ld\t%ld\t%ld\t%ld\n",year,count[0],count[1],count[2],c
ount[3]);
    if(year==2014)
        printf("%d\t%ld\t%ld\t%ld\t%ld\t%lf\n",year,count[0],count[1],cou
nt[2],count[3],pa);
    if( year>2009){
```

```
        fprintf(out, "%d\t%ld\t%ld\t%ld\t%ld\n",year,count[0],count[1],co
unt[2],count[3]);
    }
}

void initializePopulation()
{
    int cnt=0;
    for( int j=15; j<65; j++){
        for( int i=0; i<populationPerYearAndAge[1950][j]*100; i++){
            person[cnt].age=j;
            person[cnt].state=0;
            cnt++;
        }
    }
    totalPersons = cnt;
}

bool checkRate(double rate)
{
    if (rate <0.001){
        double k = rand()/(1.00+RAND_MAX);
        if(k<0.001){
            rate = rate*1000;
        }
        else{
            return false;
        }
    }
    double k = rand()/(1.00+RAND_MAX);
    if ( k < rate )
        return true;
```

```
    else
        return false;
}

void changeStatusAndAge()
{
    for( int i=0; i<totalPersons; i++){
        // change state
        double k = rand()/(1.00+RAND_MAX);
        if( person[i].state==0){
            if ( checkRate(pa) &&  person[i].age<40){
                person[i].state=1;
            }
        }
        else if( person[i].state==1){
            if ( k < pg ){
                person[i].state=2;
            }
        }
        else if( person[i].state==2){
            if ( k < pk ){
                person[i].state=1;
            }
        }
        // After their 49th year, all active PWID are assumed to cease in
jecting
        if(person[i].age>49 && person[i].state==1){
            person[i].state=2;
        }
        // After their 64th year, remove
        if(person[i].age>64){
            person[i].state=3;
```

```
        }
        // death rate depending on age and state
        k = rand()/(1.00+RAND_MAX);
        if( person[i].state==0){
            if ( k < deathRatePerAge[person[i].age] ){
                person[i].state=3;
            }
        }
        else if( person[i].state==1){
            if ( k < deathRatePerAge[person[i].age] * 13.0 ){
                person[i].state=3;
            }
        }
        else if( person[i].state==2){
            if ( k < deathRatePerAge[person[i].age] ){
                person[i].state=3;
            }
        }
        // increase age one year
        person[i].age++;
    }
}

void addNewPersons(int year)
{
    // add new person [15 years old and non PWID]
    int cnt=totalPersons;
    for( int i=0; i<populationPerYearAndAge[year+1][15]*100; i++){
// 100 instead of 1000 for the population to reduce execution time
        person[cnt].age=15;
        person[cnt].state=0;
        cnt++;
```

```
    }
    // increase the total number of population
    totalPersons = cnt;
}
int main()
{
    srand(time(NULL));

    setPopulationAge();
    setDeathRate();

    getTotalPopulationPerAge();
    pa = pa_start;
    while ( pa < pa_stop){

        snprintf(filename, 100, "result_%lf.txt",pa);
        out=fopen(filename,"w");

        for( int iter=0; iter<loops ;iter++){
            initializePopulation();
            for( int year=1950; year<2020; year++){
                printTotalPersonPerState(year);
                changeStatusAndAge();
                addNewPersons(year);
            }
        }

        fclose(out);
        pa = pa + pa_step;
    }
```

```
}
```

## REFERENCES

European Centre for Disease Prevention and Control. 2016. "Epidemiological Assessment of Hepatitis b and c Among Migrants in the EU/EEA." https://www.ecdc.europa.eu/sites/default/files/media/en/publications/Publications/epidemiological-assessment-hepatitis-B-and-C-among-migrants-EU-EEA.pdf.

European Centre for Disease Prevention and Control (ECDC). 2016. "Systematic Review on Hepatitis b and c Prevalence in the EU/EEA." https://www.ecdc.europa.eu/sites/default/files/media/en/publications/Publications/systematic-review-hepatitis-B-C-prevalence.pdf.

———. 2021. "Hepatitis c Prevalence Database." https://www.ecdc.europa.eu/en/all-topics-z/hepatitis-c/tools/hepatitis-c-prevalence-database.

Grebely, Jason, Sarah Larney, Amy Peacock, Samantha Colledge, Janni Leung, Matthew Hickman, Peter Vickerman, et al. 2019. "Global, Regional, and Country-Level Estimates of Hepatitis c Infection Among People Who Have Recently Injected Drugs." *Addiction* 114 (1): 150–66. https://doi.org/https://doi.org/10.1111/add.14393.

Hines, Lindsey A., Adam Trickey, Janni Leung, Sarah Larney, Amy Peacock, Louisa Degenhardt, Samantha Colledge, et al. 2020. "Associations Between National Development Indicators and the Age Profile of People Who Inject Drugs: Results from a Global Systematic Review and Meta-Analysis." *The Lancet Global Health* 8 (1): e76–91. https://doi.org/10.1016/S2214-109X(19)30462-0.

Lin, Lifeng, and Haitao Chu. 2020. "Meta-Analysis of Proportions Using Generalized Linear Mixed Models." *Epidemiology* 31 (5). https://journals.lww.com/epidem/Fulltext/2020/09000/Meta_analysis_of_Proportions_Using_Generalized.16.aspx.

McDonald, Scott A., Rosmawati Mohamed, Maznah Dahlui, Herlianna Naning, and Adeeba Kamarulzaman. 2014. "Bridging the Data Gaps in the Epidemiology of Hepatitis c Virus Infection in Malaysia Using Multi-Parameter Evidence Synthesis." *BMC Infectious Diseases* 14 (1): 564. https://doi.org/10.1186/s12879-014-0564-6.

Micallef, J. M., J. M. Kaldor, and G. J. Dore. 2006. "Spontaneous Viral Clearance Following Acute Hepatitis c Infection: A Systematic Review of Longitudinal Studies." *Journal of Viral Hepatitis* 13 (1): 34–41. https://doi.org/https://doi.org/10.1111/j.1365-2893.2005.00651.x.

Presanis, Anne M., O. Noel Gill, Timothy R. Chadborn, Caterina Hill, Vivian Hope, Louise Logan, Brian D. Rice, Valerie C. Delpech, A. E. Ades, and Daniela De Angelis. 2010. "Insights into the Rise in HIV Infections, 2001 to 2008: A Bayesian Synthesis of Prevalence Evidence." *AIDS* 24 (18). https://journals.lww.com/aidsonline/Fulltext/2010/11270/Insights_into_the_rise_in_HIV_infections,_2001_to.12.aspx.

Sweeting, M. J., D. De Angelis, M. Hickman, and A. E. Ades. 2008. "Estimating hepatitis C prevalence in England and Wales by synthesizing evidence from multiple data sources. Assessing data conflict and model fit." *Biostatistics* 9 (4): 715–34. https://doi.org/10.1093/biostatistics/kxn004.

Tan, Sarah, Susanna Makela, Daliah Heller, Kevin Konty, Sharon Balter, Tian Zheng, and James H. Stark. 2018. "A Bayesian Evidence Synthesis Approach to Estimate Disease Prevalence in Hard-to-Reach Populations: Hepatitis c in New York City." *Epidemics* 23: 96–109. https://doi.org/https://doi.org/10.1016/j.epidem.2018.01.002.

Thein, Hla-Hla, Qilong Yi, Gregory J. Dore, and Murray D. Krahn. 2008. "Estimation of Stage-Specific Fibrosis Progression Rates in Chronic Hepatitis c Virus Infection: A Meta-Analysis and Meta-Regression." *Hepatology* 48 (2): 418–31. https://doi.org/https://doi.org/10.1002/hep.22375.

Veen, Maaike G. van, Anne M. Presanis, Stefano Conti, Maria Xiridou, Annemarie R. Stengaard, Martin C. Donoghoe, Ard I. van Sighem, Marianne A. van der Sande, and Daniela De Angelis. 2011. "National Estimate of HIV Prevalence in the Netherlands: Comparison and Applicability of Different Estimation Tools." *AIDS* 25 (2). https://journals.lww.com/aidsonline/Fulltext/2011/01140/National_estimate_of_HIV_prevalence_in_the.14.aspx.

# Estimating the prevalence of chronic hepatitis C infection (CHC) in Iceland using Bayesian multiparameter evidence synthesis

01/07/2022

## Table of Contents


Introduction .......... 2

Methods .......... 2

- Prevalence of ex-PWID .......... 3
- Prevalence of recent PWID .......... 3
- Prevalence of CHC among recent PWID .......... 4
- Prevalence of CHC among ex-PWID .......... 4
- Prevalence of CHC among non-PWID .......... 4

Results .......... 6

Tables and Figures .......... 7

Appendix .......... 11

- Fit of the multi-state Markov model .......... 11
- Stan code for Bayesian multiparameter evidence synthesis .......... 12
- Multi-state Markov model .......... 17

References .......... 25

# INTRODUCTION

Patients with chronic hepatitis C (HCV) infection often remain asymptomatic for decades and even for life, but a proportion of them develop active chronic hepatitis, which is a progressive disease (Thein et al. 2008). Because of the largely asymptomatic nature of HCV infection, notification data reflect national screening and testing practices and do not give accurate insights into the prevalence of HCV infection. Although there are studies that have estimated the prevalence of HCV antibodies (anti-HCV) in specific groups such as the general population, first-time blood donors, or people who inject drugs (PWID) at drug treatment centers, those alone cannot be combined to produce national estimates unless some additional information regarding the composition of each HCV risk group and its prevalence in the population are known (Sweeting et al. 2008; McDonald et al. 2014; Tan et al. 2018).

# METHODS

Bayesian multiparameter evidence synthesis (MPES) has been a popular approach to formally estimate anti-HCV or HIV prevalence (Sweeting et al. 2008; McDonald et al. 2014; Tan et al. 2018; Presanis et al. 2010; Veen et al. 2011). In this project, we extend this method to estimating the CHC prevalence, with the population (15-79 years) split into three main non-overlapping risk groups, i.e., recent PWID (those who have injected in the last year), ex-PWID, and non-PWID. A unified model is assumed including parameters associated with the CHC prevalence of recent, ex-PWID, and non-PWID (denoted by $\pi_{rec}$, $\pi_{ex}$, and $\pi_{non}$, respectively) and the prevalence of these risk groups in the population (proportion of the population that belongs to each group), denoted by $\rho_{rec}$, $\rho_{ex}$, and $\rho_{non}$, respectively; $\rho_{rec} + \rho_{ex} + \rho_{non} = 1$. To estimate the CHC prevalence in the whole population, $\pi$, we used the formula:

$$\pi = \pi_{rec}\rho_{rec} + \pi_{ex}\rho_{ex} + \pi_{non}\rho_{non} \quad (1)$$

Bayesian synthesis considers all available data, allowing for an evidence-based estimation of CHC prevalence in the population, with inherent uncertainty properly accounted for. Our aim is to estimate the CHC prevalence for the total (15-79) population of Iceland in 2019 using sources of information over the last decade (2010-2019).

## Prevalence of ex-PWID

Data on the prevalence of ex-PWID (proportion of the population that is ex-PWID) are sparse and generally often unreliable. To overcome this limitation, we apply the method proposed by McDonald et al. (2014), which requires simulating a multi-state Markov model representing the non-PWID, recent PWID, and ex-PWID. In the multi-state Markov model, the simulation starts in 1950 and examines the population aged 15-79 years old. A certain number of 15-year-old individuals enter the simulation process each year. These numbers of 15-year-old individuals are obtained from the United Nations Statistic Division UNSD, being different for each country. The ageing of the population is also taken into account. Country-specific overall mortality data are retrieved from the Life Tables of the World Health Organization WHO. The duration of injecting career is also country-specific and obtained from the paper of Scott et al. (2018).

To estimate the number of ex-PWID, the model can be calibrated on the number of recent PWID provided in the EMCDDA barometer or the EMCDDA statistical bulletin (the code of the Markov model can be found in the Appendix). If there are no available data in the barometer, estimates from the systematic review of Grebely et al. (2019) could be used. Alternatively, if the national focal points suggest or provide different and updated or more accurate data for calibration purposes, we will consider their advice and adjust the model accordingly.

After applying the model for Iceland, we compute the number (and the corresponding Confidence Interval - CI) of ex-PWID in 2019. The number of ex-PWID is then divided by the population size (15-79 years) in the same year, and a Normal distribution, constrained such that $\rho_{ex} \in (0,1)$, is assumed for $\rho_{ex}$. The standard deviation of this distribution is specified to approximately correspond to the respective CI, i.e. $\left(UB_{\rho_{ex}} - LB_{\rho_{ex}}\right)/(2 \times 1.96)$, where $UB_{\rho_{ex}}$ and $LB_{\rho_{ex}}$ denote the upper and lower limit of the CI.

## Prevalence of recent PWID

The number of recent PWID in Iceland in 2019 is also projected by the multi-state Markov model described in the previous subsection. To estimate $\rho_{rec}$, the number of recent PWID is divided by the population size (15-79 years). Similar to the previous subsection, a Normal distribution, constrained such that $\rho_{rec} \in (0,1)$, is assumed for $\rho_{rec}$, with the standard deviation specified to correspond to the CI obtained from the multi-state Markov model.

## Prevalence of CHC among recent PWID

The prevalence (and the 95% CI) of CHC among recent PWID was informed by CHC prevalence data from recent PWID admitted to Vogur addiction hospital in 2019, provided by the national focal points through personal communication. The Binomial distribution in the model to inform $\pi_{rec}$ was used. Since recent, i.e. in 2019, CHC data were available for recent PWID, no adjustment for treatment with direct acting antivirals (DAAs) was made.

## Prevalence of CHC among ex-PWID

Information on CHC prevalence among ex-PWID (i.e., $\pi_{ex}$) is often difficult to obtain directly. To overcome this, data on the CHC prevalence among ever PWID admitted to Vogur addiction hospital in 2019, provided by the national focal points through personal communication, were utilized. Thus, once an estimate of $\pi_{ever}$ is available, $\pi_{ex}$ can be indirectly estimated since $\pi_{ever}$ is equal to a weighted average of $\pi_{rec}$ and $\pi_{ex}$, as shown by the following formula

$$\pi_{ever} = \frac{\rho_{rec}}{\rho_{rec} + \rho_{ex}} \pi_{rec} + \frac{\rho_{ex}}{\rho_{rec} + \rho_{ex}} \pi_{ex}, \quad (2)$$

or, equivalently,

$$\pi_{ex} = \left( \pi_{ever} - \frac{\rho_{rec}}{\rho_{rec} + \rho_{ex}} \pi_{rec} \right) \times \frac{\rho_{rec} + \rho_{ex}}{\rho_{ex}}, \quad (3)$$

Since recent, i.e. in 2019, CHC data were available for both recent and ever PWID, no adjustment for treatment with DAAs was made.

## Prevalence of CHC among non-PWID

To estimate the CHC prevalence among non-PWID in a country (after 2010), the ECDC database (European Centre for Disease Prevention and Control (ECDC) 2021) was used. The ECDC group has carefully and critically collected information from relevant studies across the EU/EEA region in cooperation with the focal points of each country. On the basis of the representativeness of each study and other factors (European Centre for Disease Prevention and Control (ECDC) 2016), a risk of bias score was assigned to each study, with higher values indicating higher-quality studies. When higher-quality studies are available in a country ($\geq 4$),

they are used to estimate the overall CHC prevalence in that country among non-PWID. If there are no higher-quality studies, general population estimates with a lower quality (<4) are pooled. If no general population prevalence estimates are available, data on the prevalence of HCV in pregnant women could be used as a prevalence measure. Finally, if data on pregnant women are lacking too, first-time blood donor studies could be an option. However, if the national focal point agrees or recommends, estimates from a neighboring country could be used instead, or we could use any other relevant information provided/suggested by the national focal point.

We directly use CHC prevalence data from studies in the general population, if available. Currently, we used data among individuals without past injection history attending the Livio Reykjavik fertility clinic during 2014-18 (Olafsson et al. 2021). To adjust for the fact that some individuals in the general population may have been treated DAAs, we took into account the data from the treatment as prevention (TraP HepC) program (Olafsson et al. 2021). Based on the information provided in Olafsson et al. (2021), 717 individuals have been cured since the beginning of the program (Figure 3 in Olafsson et al. 2021), of whom, 33% were assumed to be recent PWID, 51.1% ex-PWID, and 15.9% non-PWID (Table 2 in Olafsson et al. 2021). Therefore, it is assumed that 114 CHC-positive non-PWID have been cured since the onset of the program in 2016.

Thus, to adjust the CHC prevalence among non-PWID for DAAs, we estimated the CHC prevalence in the general population by

$$\pi_{non} = \frac{N_{15,79}\rho_{non}\tilde{\pi}_{non} - \frac{3}{5}N_{non}(cured)}{N_{15,79}\rho_{non}}. \quad (4)$$

where $\tilde{\pi}_{non}$ denotes the CHC estimate in the general population when information on DAAs is ignored and $N_{non}(cured)$ denotes the number of cured non-PWID individuals. Note that we subtracted $\frac{3}{5}N_{non}(cured)$ cured individuals since the data in Olafsson et al. (2021) were collected in 2014-18.

# RESULTS

The above-mentioned approach was used to estimate the prevalence of CHC in 2019 (considering ages between 15-79 years: 276,696).

The aggregated data used by our approach are briefly presented in Table 1. The multi-state Markov model to estimate the prevalence of recent and ex-PWID was calibrated on the estimates reported in the paper of Olafsson et al. (2021). In Iceland, there was 1 study on individuals without past injection history attending the Livio Reykjavik fertility clinic during 2014-18, which included CHC data (Olafsson et al. 2021). Results, when information on DAAs is ignored, are presented in Table 2. However, 717 have been cured since the onset of the TraP HepC program in Iceland, which should be taken into consideration.

The corresponding results accounting for the DAA uptake are presented in Table 3. The prevalence of recent and ex-PWID (proportion of the population that belongs to these groups) was low in Iceland (about 0.26% and 0.6%, respectively) corresponding to 710 (95% CI: 550-870) recent PWID and 1,650 (95% CI: 1,370-1,890) ex-PWID in the population. However, the CHC prevalence in these groups was non-negligible (Table 3) being 12.15% and 8.5%, respectively, although much lower than that before the start of the TraP HepC program. This translates to 86 (95% CI: 57-125) and 140 (95% CI: 70-224) recent and ex-PWID aged 15-79 living with CHC infection in Iceland in 2019. The CHC prevalence in the general population was 0.02% (95% CI: 0%-0.11%), much lower than that of the high-risk groups. Taking all pieces of information into account, the overall CHC prevalence in Iceland in 2019 was equal to 0.1% (95% CI: 0.05%-0.2%), which corresponds to 279 (95% CI: 151-547) individuals aged 15-79 years with CHC infection.

# TABLES AND FIGURES

*Table 1. Data (real) contributing to the estimation of CHC prevalence in Iceland in 2019.*

| Parameter | Number | Num | Den | Notes | Year |
|---|---|---|---|---|---|
| $\rho_{rec}$ | 710 (550-870) | | | Method based on McDonald et al. | 2019 |
| $\rho_{ex}$ | 1,650 (1,370-1,890) | | | Method based on McDonald et al. | 2019 |
| $\pi_{rec}$ | | 32 | 267† | Vogur addiction hospital | 2019 |
| $\pi_{ever}$ | | 39 | 411 | Vogur addiction hospital | 2019 |
| $\pi_{non}$ | | 1†† | 3,998 | Livio Reykjavik fertility clinic (Olafsson et al. 2021); Risk of bias = NA | 2014-18 |

*Notes*: Although it looks counter-intuitive, **higher risk of bias score denotes a higher-quality study** (range from 0 to 6); † Adjusted to correspond to 12% CHC prevalence; †† Removing individuals with IDU history.

*Table 2. Results from the method assuming no heterogeneity in the estimation of the CHC prevalence in the general population from different studies (i.e., similar to a fixed-effect meta-analysis).*

| Parameter | Posterior median | Posterior sd | LB (2.5%) | UB (97.5%) |
|---|---|---|---|---|
| $\rho_{rec}$ (%) | 0.26 | 0.03 | 0.2 | 0.32 |
| $\rho_{ex}$ (%) | 0.6 | 0.05 | 0.5 | 0.69 |
| $\pi_{rec}$ (%) | 12.15 | 2 | 8.6 | 16.46 |
| $\pi_{ex}$ (%) | 8.5 | 2.26 | 4.38 | 13.24 |
| $\pi_{non}$ (%) | 0.04 | 0.03 | 0.01 | 0.14 |
| $\pi$ (%) | 0.13 | 0.04 | 0.08 | 0.22 |
| Number with CHC | 347 | 103 | 219 | 616 |
| Pr(Recent PWID\|CHC) (%) | 24.58 | 8.26 | 12.37 | 44.29 |
| Pr(Ex-PWID\|CHC) (%) | 40.17 | 11.88 | 18.32 | 63.58 |
| Pr(Non-PWID\|CHC) (%) | 33.58 | 15.3 | 6.6 | 63.6 |

*Notes*: Information on DAAs is not taken into account.

*Abbreviations*: $\rho_{rec}$, prevalence of recent PWID (proportion of the population that is recent PWID); $\rho_{ex}$, prevalence of ex-PWID (proportion of the population that is ex-PWID); $\pi_{rec}$, CHC prevalence among recent PWID; $\pi_{ex}$, CHC prevalence among ex-PWID; $\pi_{non}$, CHC prevalence among non-PWID; $\pi$ overall CHC prevalence in Iceland; LB, Lower Boundary; UP, Upper Boundary

*Table 3. Results from the method assuming no heterogeneity in the estimation of the CHC prevalence in the general population from different studies (i.e., similar to a fixed-effect meta-analysis).*

| Parameter | Posterior median | Posterior sd | LB (2.5%) | UB (97.5%) |
|---|---|---|---|---|
| $\rho_{rec}$ (%) | 0.26 | 0.03 | 0.2 | 0.32 |
| $\rho_{ex}$ (%) | 0.6 | 0.05 | 0.5 | 0.69 |
| $\pi_{rec}$ (%) | 12.15 | 2 | 8.6 | 16.46 |
| $\pi_{ex}$ (%) | 8.5 | 2.26 | 4.38 | 13.24 |
| $\pi_{non}$ (%) | 0.02 | 0.03 | 0 | 0.11 |
| $\pi$ (%) | 0.1 | 0.04 | 0.05 | 0.2 |
| Number with CHC | 279 | 103 | 151 | 547 |
| Pr(Recent PWID\|CHC) (%) | 30.76 | 12.63 | 14.21 | 21.58 |
| Pr(Ex-PWID\|CHC) (%) | 50.5 | 16.76 | 62.34 | 84.87 |
| Pr(Non-PWID\|CHC) (%) | 17 | 23.93 | 0 | 58.37 |

*Notes*: The number of individuals treated with DAAs is taken into account.

*Abbreviations*: $\rho_{rec}$, prevalence of recent PWID (proportion of the population that is recent PWID); $\rho_{ex}$, prevalence of ex-PWID (proportion of the population that is ex-PWID); $\pi_{rec}$, CHC prevalence among recent PWID; $\pi_{ex}$, CHC prevalence among ex-PWID; $\pi_{non}$, CHC prevalence among non-PWID; $\pi$ overall CHC prevalence in Iceland; LB, Lower Boundary; UP, Upper Boundary

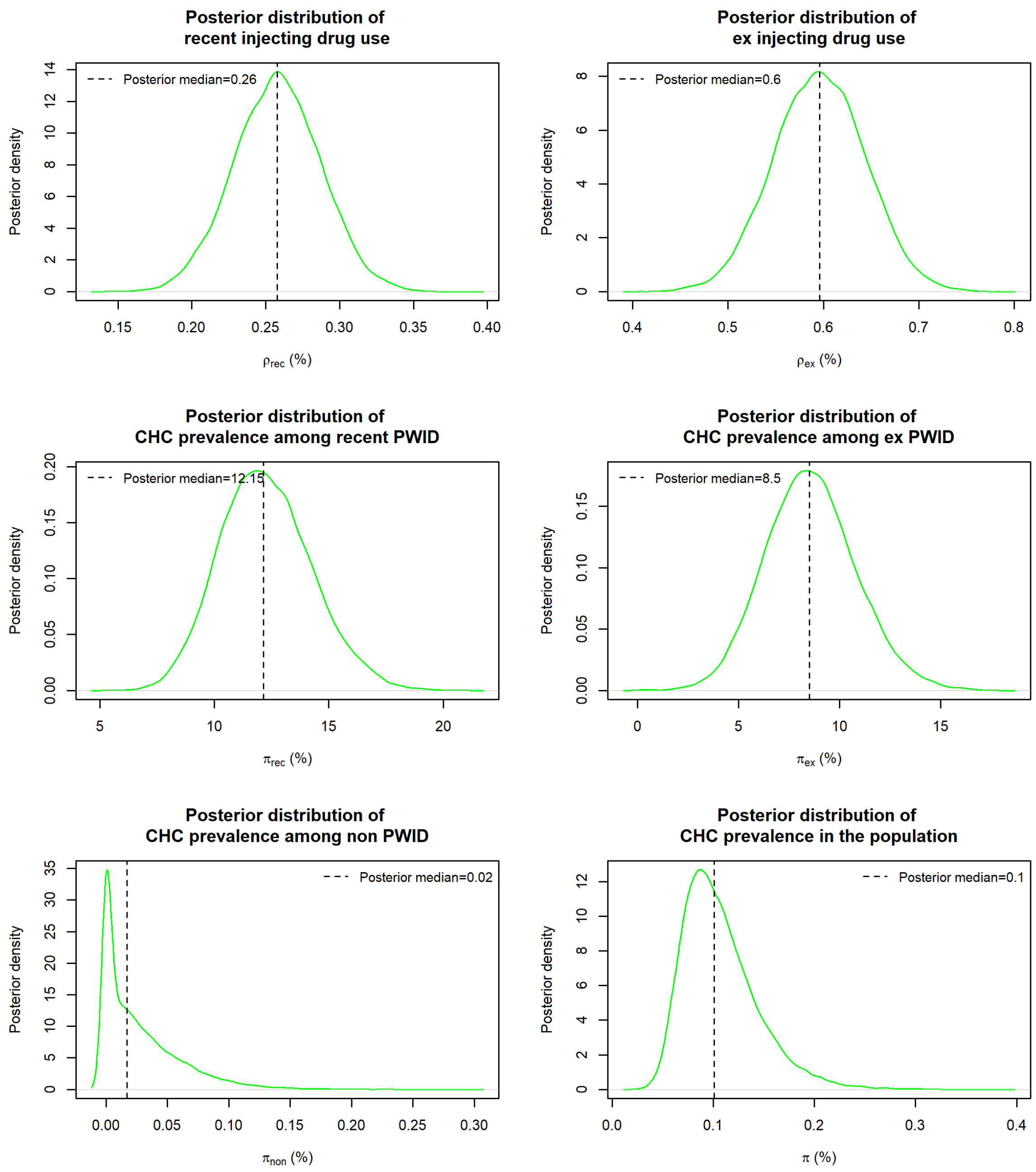


*Figure 1. Posterior distributions of the key model parameters using the approach that assumes no heterogeneity in the estimation of the CHC prevalence in the general population (primarily non-PWID) from different studies (i.e., similar to a fixed-effect meta-analysis), with the number of individuals treated with DAA taken into account.*

# APPENDIX

## Fit of the multi-state Markov model

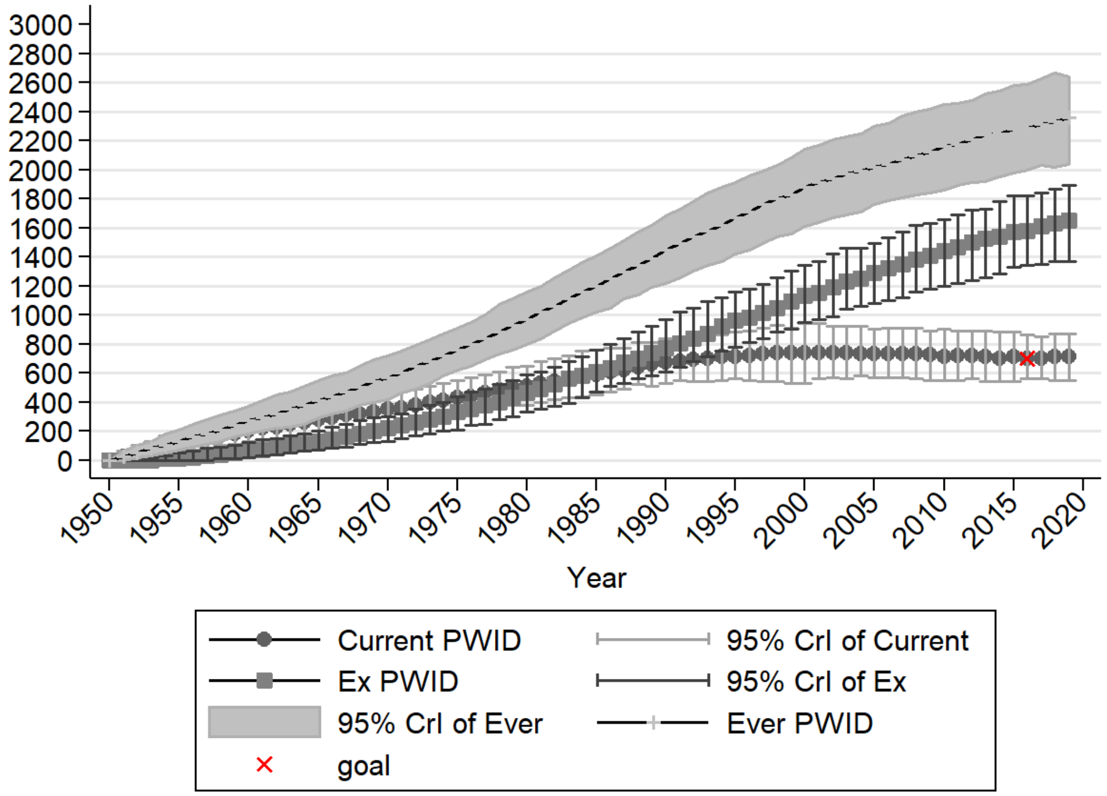


*Figure 2: Model predictions for the numbers of current and ex-PWID (people who inject drugs). The solid black line and shaded grey error bars show the median and 95% credible intervals (95% CrI) for the model projections. For comparison, asterisks indicate the observed country's data.*

## Stan code for Bayesian multiparameter evidence synthesis

```
data {
  int<lower=0> N1579; // Population of 15-79 in `Country`
  int<lower=1> Knon; // Number of studies in the ECDC data for `Country`
  int<lower=1> Kever; // Number of studies in the EMCDDA data for ever us
ers in `Country`
  int<lower=1> Kcur; // Number of studies in the EMCDDA barometer (for HC
V prevalence of PWID) in `Country`
  int<lower=1> Ncur_cured; // Number of individuals cured with DAAs from
2015 to 2019 among non-PWID
  int<lower=1> Nex_cured; // Number of individuals cured with DAAs from 2
015 to 2019 among ex-PWID
  int<lower=1> Nnon_cured; // Number of individuals cured with DAAs from
2015 to 2019 among recent PWID

  real p_cur_mean; // Prior mean for the prevalence of current use in `Co
untry`
  real<lower=0> p_cur_sd; // Prior sd for the prevalence of current use i
n `Country`

  real p_ex_mean; // Prior mean for the prevalence of ex-use in `Country`
  real<lower=0> p_ex_sd; // Prior sd for the prevalence of ex-use in `Cou
ntry`

  int<lower=0> Nst_CHC_cur[Kcur]; // Number of individuals in the study e
stimating CHC among recent PWID
  int<lower=0> Yst_CHC_cur[Kcur]; // Number of HCV+ in the study estimati
ng CHC among recent PWID

  int<lower=0> Nst_CHC_ever[Kever]; // Number of individuals in the study
estimating CHC among ever IDU
  int<lower=0> Yst_CHC_ever[Kever]; // Number of HCV+ in the study estima
```

```
ting CHC among ever PWID

  int<lower=0> Nst_CHC_non[Knon]; // Number of individuals in the study e
stimating CHC among non PWID
  int<lower=0> Yst_CHC_non[Knon]; // Number of HCV+ in the study estimati
ng CHC among non PWID

  vector<lower=0>[3] alpha; // parameter of the Diriclet prior

  real HCVclear_mean; // Prior mean for the HCV clearance probability
  real<lower=0> HCVclear_sd; // Prior sd for the HCV clearance probabilit
y
}

// Block defining the original parameters
parameters {
  // The parameters to be sampled
  simplex[3] rho; // Prevalence of the three risk groups
  real<lower=0,upper=1> CHCpi_cur; // P(CHC+|Cur)
  real<lower=0,upper=1> CHCpi_ex; // P(CHC+|Ex)
  real<lower=0,upper=1> CHCpi_non; // P(CHC+|Non)

  real<lower=0,upper=1-CHCpi_cur> HCVclear; // Probability of HCV clearan
ce; upper bound = 1-prevalence of chronic HCV
}


transformed parameters {
  // Change scales
  real<lower=0,upper=1> rho_ever;
  real<lower=0,upper=1> CHCpi_ever;
  real<lower=0,upper=1> pi_ever;
```

```
  real<lower=0,upper=1> pi_non;

  rho_ever = rho[1] + rho[2];
  CHCpi_ever = CHCpi_cur*rho[1]/rho_ever + CHCpi_ex*rho[2]/rho_ever;
  pi_ever = CHCpi_ever/(1-HCVclear);
  pi_non = CHCpi_non/(1-HCVclear);
}


// Binomial regression model
model {
  // Priors
  rho ~ dirichlet(alpha);

  //////////////////////////////
  // Likelihood contributions //
  //////////////////////////////

  // Probability of HCV clearance
  HCVclear ~ normal(HCVclear_mean,HCVclear_sd);

  // Prevalence of current use
  rho[1] ~ normal(p_cur_mean,p_cur_sd);

  // Prevalence of ex-use
  rho[2] ~ normal(p_ex_mean,p_ex_sd);

  // Prevalence of chronic HCV among current users
  Yst_CHC_cur ~ binomial(Nst_CHC_cur,CHCpi_cur);

  // Prevalence of HCV among ever users
  Yst_CHC_ever ~ binomial(Nst_CHC_ever,CHCpi_ever);
```

```
  // HCV+ among non
  Yst_CHC_non ~ binomial(Nst_CHC_non,CHCpi_non);
}


generated quantities {
  // Functions of parameters
  real overalCHC;
  real logit_CHCpi_cur;
  real logit_CHCpi_ex;
  real logit_CHCpi_non;
  real logit_rho_cur;
  real logit_rho_ex;
  real logit_rho_non;
  real pEverGivenCHC;
  real pCurGivenCHC;
  real pExGivenCHC;
  real pNonGivenCHC;
  real logit_HCVclear;
  real NumberCHC;

  real CHCDAApi_non; // P(CHC+|Non) adjusted for DAA
  real CHCDAApi_cur; // P(CHC+|Cur) adjusted for DAA
  real CHCDAApi_ex; // P(CHC+|Ex) adjusted for DAA
  real CHCDAApi_ever; // P(CHC+|Ever) adjusted for DAA

  CHCDAApi_cur = ( N1579*rho[1]*CHCpi_cur - 0)/(N1579*rho[1]);
  CHCDAApi_ex = ( N1579*rho[2]*CHCpi_ex - 0)/(N1579*rho[2]);
  CHCDAApi_non = ( N1579*rho[3]*CHCpi_non - 0.6*Nnon_cured)/(N1579*rho[3]
);
  CHCDAApi_ever = CHCDAApi_cur*rho[1]/rho_ever + CHCDAApi_ex*rho[2]/rho_e
```

```
ver;

  // Overall HCV prevalence
  overalCHC = 100*(rho[1]*CHCDAApi_cur + rho[2]*CHCDAApi_ex +  rho[3]*CHC
DAApi_non);
  pEverGivenCHC = CHCDAApi_ever*rho_ever/(overalCHC/100);
  pCurGivenCHC = CHCDAApi_cur*rho[1]/(overalCHC/100);
  pExGivenCHC = CHCDAApi_ex*rho[2]/(overalCHC/100);
  pNonGivenCHC = CHCDAApi_non*(1-rho_ever)/(overalCHC/100);
  NumberCHC = round(overalCHC*N1579/100);

  logit_rho_cur = logit(rho[1]);
  logit_rho_ex = logit(rho[2]);
  logit_rho_non = logit(rho[3]);
  logit_CHCpi_cur = logit(CHCpi_cur);
  logit_CHCpi_ex = logit(CHCpi_ex);
  logit_CHCpi_non = logit(CHCpi_non);
  logit_HCVclear = logit(HCVclear);
}
```

## Multi-state Markov model

```
#include <stdio.h>
#include <stdlib.h>
#include <string.h>
#include <math.h>
#include <time.h>

#define loops 100
int populationPerYearAndAge[2021][66];
double deathRatePerAge[66];
int totalPopulationPerYear[2021];

FILE *F_DeathRate=fopen("`Country`-deathRates.txt","r");
FILE *F_Population=fopen("`Country`-population.txt","r");
FILE *out;

double pa_start = 0.00012;                    // Setting Variable
double pa_step  = 0.000002;                   // Setting Variable
double pa_stop  = 0.000123;                   // Setting Variable

double pa;
double pg = 1.0/11.7;   // Assumed average active injecting career of  11
.7 years
double pk = 0.004*12.0;     // Relapse rate of 0.004/month

struct people{
    int age;
    int state;
};

char filename[100];
struct people person[50000000];
```

```
long int totalPersons=0;

void setPopulationAge()
{
    for( int j=0; j<71; j++){
        for( int i=0; i<51; i++){
            fscanf(F_Population,"%d",&populationPerYearAndAge[1950+j][15+
i]);
        }
    }
}

void setDeathRate()
{
    for( int i=0; i<51; i++){
        fscanf(F_DeathRate,"%lf",&deathRatePerAge[15+i*5]);
        for( int j=0; j<5; j++){
            deathRatePerAge[15+i*5+j] = deathRatePerAge[15+i*5];
        }
    }
}

void getTotalPopulationPerAge()
{
    for( int j=0; j<71; j++){
        for( int i=0; i<51; i++){
            totalPopulationPerYear[1950+j] += populationPerYearAndAge[195
0+j][15+i];
        }
    }
}
```

```
void printTotalPopulationPerAge()
{
    printf("---- - ----------\n");
    printf("Year - Population\n");
    printf("---- - ----------\n");
    for( int i=1950; i<2021; i++){
        printf("%d - %d\n",i,totalPopulationPerYear[i]);
    }
}

void printDeathRatePerAge()
{
    printf("--- - -----\n");
    printf("Age - Rate\n");
    printf("--- - -----\n");
    for( int i=15; i<65; i++){
        printf("%d - %lf\n",i,deathRatePerAge[i]);
    }
}

void printTotalPersonPerState( int year)
{
    int count[4]= {0,0,0,0};
    for( int i=0; i<totalPersons; i++){
        count[person[i].state]++;
    }
    //printf("%d\t%ld\t%ld\t%ld\t%ld\n",year,count[0],count[1],count[2],count[3]);
    if(year==2014)
        printf("%d\t%ld\t%ld\t%ld\t%ld\t%lf\n",year,count[0],count[1],count[2],count[3],pa);
    if( year>2009){
```

```
        fprintf(out, "%d\t%ld\t%ld\t%ld\t%ld\n",year,count[0],count[1],co
unt[2],count[3]);
    }
}

void initializePopulation()
{
    int cnt=0;
    for( int j=15; j<65; j++){
        for( int i=0; i<populationPerYearAndAge[1950][j]*100; i++){
            person[cnt].age=j;
            person[cnt].state=0;
            cnt++;
        }
    }
    totalPersons = cnt;
}

bool checkRate(double rate)
{
    if (rate <0.001){
        double k = rand()/(1.00+RAND_MAX);
        if(k<0.001){
            rate = rate*1000;
        }
        else{
            return false;
        }
    }
    double k = rand()/(1.00+RAND_MAX);
    if ( k < rate )
        return true;
```

```
    else
        return false;
}

void changeStatusAndAge()
{
    for( int i=0; i<totalPersons; i++){
        // change state
        double k = rand()/(1.00+RAND_MAX);
        if( person[i].state==0){
            if ( checkRate(pa) &&  person[i].age<40){
                person[i].state=1;
            }
        }
        else if( person[i].state==1){
            if ( k < pg ){
                person[i].state=2;
            }
        }
        else if( person[i].state==2){
            if ( k < pk ){
                person[i].state=1;
            }
        }
        // After their 49th year, all active PWID are assumed to cease in
jecting
        if(person[i].age>49 && person[i].state==1){
            person[i].state=2;
        }
        // After their 64th year, remove
        if(person[i].age>64){
            person[i].state=3;
```

```
        }
        // death rate depending on age and state
        k = rand()/(1.00+RAND_MAX);
        if( person[i].state==0){
            if ( k < deathRatePerAge[person[i].age] ){
                person[i].state=3;
            }
        }
        else if( person[i].state==1){
            if ( k < deathRatePerAge[person[i].age] * 13.0 ){
                person[i].state=3;
            }
        }
        else if( person[i].state==2){
            if ( k < deathRatePerAge[person[i].age] ){
                person[i].state=3;
            }
        }
        // increase age one year
        person[i].age++;
    }
}

void addNewPersons(int year)
{
    // add new person [15 years old and non PWID]
    int cnt=totalPersons;
    for( int i=0; i<populationPerYearAndAge[year+1][15]*100; i++){
// 100 instead of 1000 for the population to reduce execution time
        person[cnt].age=15;
        person[cnt].state=0;
        cnt++;
```

```
    }
    // increase the total number of population
    totalPersons = cnt;
}
int main()
{
    srand(time(NULL));

    setPopulationAge();
    setDeathRate();

    getTotalPopulationPerAge();
    pa = pa_start;
    while ( pa < pa_stop){

        snprintf(filename, 100, "result_%lf.txt",pa);
        out=fopen(filename,"w");

        for( int iter=0; iter<loops ;iter++){
            initializePopulation();
            for( int year=1950; year<2020; year++){
                printTotalPersonPerState(year);
                changeStatusAndAge();
                addNewPersons(year);
            }
        }

        fclose(out);
        pa = pa + pa_step;
    }
```

```
}
```

# REFERENCES

European Centre for Disease Prevention and Control (ECDC). 2016. "Systematic Review on Hepatitis b and c Prevalence in the EU/EEA." https://www.ecdc.europa.eu/sites/default/files/media/en/publications/Publications/systematic-review-hepatitis-B-C-prevalence.pdf.

———. 2021. "Hepatitis c Prevalence Database." https://www.ecdc.europa.eu/en/all-topics-z/hepatitis-c/tools/hepatitis-c-prevalence-database.

Grebely, Jason, Sarah Larney, Amy Peacock, Samantha Colledge, Janni Leung, Matthew Hickman, Peter Vickerman, et al. 2019. "Global, Regional, and Country-Level Estimates of Hepatitis c Infection Among People Who Have Recently Injected Drugs." *Addiction* 114 (1): 150–66. https://doi.org/https://doi.org/10.1111/add.14393.

McDonald, Scott A., Rosmawati Mohamed, Maznah Dahlui, Herlianna Naning, and Adeeba Kamarulzaman. 2014. "Bridging the Data Gaps in the Epidemiology of Hepatitis c Virus Infection in Malaysia Using Multi-Parameter Evidence Synthesis." *BMC Infectious Diseases* 14 (1): 564. https://doi.org/10.1186/s12879-014-0564-6.

Olafsson, Sigurdur, Ragnheidur H. Fridriksdottir, Thorvardur J. Love, Thorarinn Tyrfingsson, Valgerdur Runarsdottir, Ingunn Hansdottir, Ottar M. Bergmann, et al. 2021. "Cascade of Care During the First 36 Months of the Treatment as Prevention for Hepatitis c (TraP HepC) Programme in Iceland: A Population-Based Study." *The Lancet Gastroenterology & Hepatology* 6 (8): 628–37. https://doi.org/10.1016/S2468-1253(21)00137-0.

Presanis, Anne M., O. Noel Gill, Timothy R. Chadborn, Caterina Hill, Vivian Hope, Louise Logan, Brian D. Rice, Valerie C. Delpech, A. E. Ades, and Daniela De Angelis. 2010. "Insights into the Rise in HIV Infections, 2001 to 2008: A Bayesian Synthesis of Prevalence Evidence." *AIDS* 24 (18). https://journals.lww.com/aidsonline/Fulltext/2010/11270/Insights_into_the_rise_in_HIV_infections,_2001_to.12.aspx.

Scott, Nick, Sigurour Ólafsson, Magnús Gottfreosson, Thorarinn Tyrfingsson, Valgerdur Rúnarsdóttir, Ingunn Hansdottir, Ubaldo Benitez Hernandez, Guorún Sigmundsdóttir, and Margaret Hellard. 2018. "Modelling the Elimination of Hepatitis c as a Public Health Threat in

Iceland: A Goal Attainable by 2020.” *Journal of Hepatology* 68 (5): 932–39. https://doi.org/10.1016/j.jhep.2017.12.013.

Sweeting, M. J., D. De Angelis, M. Hickman, and A. E. Ades. 2008. “Estimating hepatitis C prevalence in England and Wales by synthesizing evidence from multiple data sources. Assessing data conflict and model fit.” *Biostatistics* 9 (4): 715–34. https://doi.org/10.1093/biostatistics/kxn004.

Tan, Sarah, Susanna Makela, Daliah Heller, Kevin Konty, Sharon Balter, Tian Zheng, and James H. Stark. 2018. “A Bayesian Evidence Synthesis Approach to Estimate Disease Prevalence in Hard-to-Reach Populations: Hepatitis c in New York City.” *Epidemics* 23: 96–109. https://doi.org/https://doi.org/10.1016/j.epidem.2018.01.002.

Thein, Hla-Hla, Qilong Yi, Gregory J. Dore, and Murray D. Krahn. 2008. “Estimation of Stage-Specific Fibrosis Progression Rates in Chronic Hepatitis c Virus Infection: A Meta-Analysis and Meta-Regression.” *Hepatology* 48 (2): 418–31. https://doi.org/https://doi.org/10.1002/hep.22375.

Veen, Maaike G. van, Anne M. Presanis, Stefano Conti, Maria Xiridou, Annemarie R. Stengaard, Martin C. Donoghoe, Ard I. van Sighem, Marianne A. van der Sande, and Daniela De Angelis. 2011. “National Estimate of HIV Prevalence in the Netherlands: Comparison and Applicability of Different Estimation Tools.” *AIDS* 25 (2). https://journals.lww.com/aidsonline/Fulltext/2011/01140/National_estimate_of_HIV_prevalence_in_the.14.aspx.

# Estimating the prevalence of chronic hepatitis C infection (CHC) in Ireland using Bayesian multiparameter evidence synthesis

17/07/2022

## Table of Contents


Introduction ..... 2

Methods ..... 2

- Prevalence of ex-PWID ..... 3
- Prevalence of recent PWID ..... 3
- Prevalence of CHC among recent PWID ..... 4
- Prevalence of CHC among ex-PWID ..... 4
- Prevalence of CHC among non-PWID ..... 5
- Sensitivity analysis including migrants from endemic countries ..... 6

Results ..... 7

- Limitations and Discussion ..... 8

Tables and Figures ..... 10

Appendix ..... 15

- Fit of the multi-state Markov model ..... 15
- Stan code for Bayesian multiparameter evidence synthesis ..... 16
- Multi-state Markov model ..... 22

References ..... 30

# INTRODUCTION

Patients with chronic hepatitis C (HCV) infection often remain asymptomatic for decades and even for life, but a proportion of them develop active chronic hepatitis, which is a progressive disease (Thein et al. 2008). Because of the largely asymptomatic nature of HCV infection, notification data reflect national screening and testing practices and do not give accurate insights into the prevalence of HCV infection. Although there are studies that have estimated the prevalence of HCV antibodies (anti-HCV) in specific groups such as the general population, first-time blood donors, or people who inject drugs (PWID) at drug treatment centers, those alone cannot be combined to produce national estimates unless some additional information regarding the composition of each HCV risk group and its prevalence in the population are known (Sweeting et al. 2008; McDonald et al. 2014; Tan et al. 2018).

# METHODS

Bayesian multiparameter evidence synthesis (MPES) has been a popular approach to formally estimate anti-HCV or HIV prevalence (Sweeting et al. 2008; McDonald et al. 2014; Tan et al. 2018; Presanis et al. 2010; Veen et al. 2011). In this project, we extend this method to estimating the CHC prevalence, with the population (15-79 years) split into three main non-overlapping risk groups, i.e., recent PWID (those who have injected in the last year), ex-PWID, and non-PWID. A unified model is assumed including parameters associated with the CHC prevalence of recent, ex-PWID, and non-PWID (denoted by $\pi_{rec}$, $\pi_{ex}$, and $\pi_{non}$, respectively) and the prevalence of these risk groups in the population (denoted by $\rho_{rec}$, $\rho_{ex}$, and $\rho_{non}$, respectively; $\rho_{rec} + \rho_{ex} + \rho_{non} = 1$). To estimate the CHC prevalence in the whole population, $\pi$, we used the formula:

$$\pi = \pi_{rec}\rho_{rec} + \pi_{ex}\rho_{ex} + \pi_{non}\rho_{non} \quad (1)$$

Bayesian synthesis considers all available data, allowing for an evidence-based estimation of CHC prevalence in the population, with inherent uncertainty properly accounted for. Our aim is to estimate the CHC prevalence for the total (15-79) population of Ireland in 2019 using sources of information over the last decade (2010-2019).

## Prevalence of ex-PWID

Data on the prevalence of ex-PWID are sparse and generally often unreliable. To overcome this limitation, we apply the method proposed by McDonald et al. (2014), which requires simulating a multi-state Markov model representing the non-PWID, recent PWID, and ex-PWID. In the multi-state Markov model, the simulation starts in 1950 and examines the population aged 15-79 years old. A certain number of 15-year-old individuals enter the simulation process each year. These numbers of 15-year-old individuals are obtained from the United Nations Statistic Division UNSD, being different for each country. The ageing of the population is also taken into account. Country-specific overall mortality data are retrieved from the Life Tables of the World Health Organization WHO. The duration of injecting career is also country-specific and obtained from the paper of Hines et al. (2020). However, since no data for Ireland are reported in Hines et al. (2020), we used the duration of injecting career from the United Kingdom.

To estimate the number of ex-PWID, the model can be calibrated on the number of recent PWID provided in the EMCDDA barometer or the EMCDDA statistical bulletin (the code of the Markov model can be found in the Appendix). If there are no available data in the barometer, estimates from the systematic review of Grebely et al. (2019) could be used. Currently, the multi-state Markov model was calibrated on the estimated number of ever PWID reported in the paper of Carew et al. (2017) for 2014 after removing deceased individuals.

After applying the model for Ireland, we compute the number (and the corresponding Confidence Interval - CI) of ex-PWID in 2019. The number of ex-PWID is then divided by the population size (15-79 years) in the same year, and a Normal distribution, constrained such that $\rho_{ex} \in (0,1)$, is assumed for $\rho_{ex}$. The standard deviation of this distribution is specified to approximately correspond to the respective CI, i.e. $\left(UB_{\rho_{ex}} - LB_{\rho_{ex}}\right)/(2 \times 1.96)$, where $UB_{\rho_{ex}}$ and $LB_{\rho_{ex}}$ denote the upper and lower limit of the CI.

## Prevalence of recent PWID

The number of recent PWID in Ireland in 2019 is also projected by the multi-state Markov model described in the previous subsection. To estimate $\rho_{rec}$, the number of recent PWID is divided by the population size (15-79 years). Similar to the previous subsection, a Normal

distribution, constrained such that $\rho_{rec} \in (0,1)$, is assumed for $\rho_{rec}$, with the standard deviation specified to correspond to the CI obtained from the multi-state Markov model.

## Prevalence of CHC among recent PWID

The prevalence of CHC among recent PWID was informed by CHC prevalence data from customized audit forms sent to general practitioners (GP) in 11 addiction treatment centers (Murphy, Thornton, and Bourke 2018), with available data up to 2016. However, some people may have been treated with direct-acting antivirals (DAAs), with the sustained virologic response ($SVR_{PWID}$) among PWID estimated to be 88% (95% CI: 80% to 93%) (Graf et al. 2019). Based on data from the National Hepatitis C Treatment Programme (NHCTP) provided by the national focal points, the number of individuals treated with DAAs from 2014 to 2019 is equal to 4,829, with approximately 61.29% of them having been infected by injecting drug use. Thus, $N_{PWID|DAA} \approx$ 2,960 PWID (recent or ex) are assumed to have been treated with DAAs. However, the proportions of recent and ex-PWID among treated ever PWID individuals are unknown. In this report, we make the assumption that these are proportional to the proportions of recent and ex-PWID among CHC-positive individuals, i.e. Pr(Recent PWID|CHC) and Pr(Ex-PWID|CHC), as estimated by our model when the DAA uptake is ignored (Table 2). Thus, the CHC prevalence among recent PWID, adjusted for DAAs, can be estimated by

$$\pi_{rec} = \frac{N_{15,79}\rho_{rec}\tilde{\pi}_{rec} - N_{PWID|DAA}\frac{\text{Pr(Recent PWID|CHC)}}{\text{Pr(Recent PWID|CHC)} + \text{Pr(Ex-PWID|CHC)}}SVR_{PWID}}{N_{15,79}\rho_{rec}}, \quad (2)$$

where $\tilde{\pi}_{rec}$ denotes the CHC estimate derived solely from the paper of Murphy, Thornton, and Bourke (2018).

## Prevalence of CHC among ex-PWID

Information on CHC prevalence among ex-PWID (i.e., $\pi_{ex}$) is often difficult to obtain directly. To overcome this, CHC prevalence data on ever PWID could be used. Then, once an estimate of $\pi_{ever}$ is available, $\pi_{ex}$ can be indirectly informed since $\pi_{ever}$ is equal to a weighted average of $\pi_{rec}$ and $\pi_{ex}$, as shown by the following formula

$$\pi_{ever} = \frac{\rho_{rec}}{\rho_{rec} + \rho_{ex}}\pi_{rec} + \frac{\rho_{ex}}{\rho_{rec} + \rho_{ex}}\pi_{ex}, \quad (3)$$

or, equivalently,

$$\pi_{ex} = \left(\pi_{ever} - \frac{\rho_{rec}}{\rho_{ever}}\pi_{rec}\right) \times \frac{\rho_{ever}}{\rho_{ex}}. \quad (4)$$

However, some ex-PWID may have been treated with DAAs. Similarly to the procedure described in the previous subsection, the CHC prevalence among ex-PWID, adjusted for DAAs, can be estimated by

$$\pi_{ex} = \frac{N_{15,79}\rho_{ex}\tilde{\pi}_{ex} - N_{PWID|DAA}\frac{\Pr(\text{Ex-PWID|CHC})}{\Pr(\text{Recent PWID|CHC}) + \Pr(\text{Ex-PWID|CHC})}SVR_{PWID}}{N_{15,79}\rho_{ex}}. \quad (5)$$

where $\tilde{\pi}_{ex}$ denotes the CHC prevalence among ex-PWID ignoring the contribution of DAAs. To inform $\pi_{ex}$, we considered CHC data among ex-PWID from the paper of Murphy, Thornton, and Bourke (2018) and CHC data among ever PWID receiving opioid substitution therapy in primary care practices in Ireland up to 2016 (Murtagh et al. 2018).

## Prevalence of CHC among non-PWID

To estimate the CHC prevalence among non-PWID in a country (after 2010), the ECDC database (European Centre for Disease Prevention and Control (ECDC) 2021) was used. The ECDC group has carefully and critically collected information from relevant studies across the EU/EEA region in cooperation with the focal points of each country. On the basis of the representativeness of each study and other factors (European Centre for Disease Prevention and Control (ECDC) 2016), a risk of bias score was assigned to each study, with higher values indicating higher-quality studies. When higher-quality studies are available in a country (≥ 4), they are used to estimate the overall CHC prevalence in that country among non-PWID. If there are no higher-quality studies, general population estimates with a lower quality (<4) are pooled. If no general population prevalence estimates are available, data on the prevalence of HCV in pregnant women could be used as a prevalence measure. Finally, if data on pregnant women are lacking too, first-time blood donor studies could be an option. However, if the national focal point agrees or recommends, estimates from a neighboring country could be used instead, or we could use any other relevant information provided/suggested by the national focal point.

In Ireland, there was 1 study on pregnant women in 2016-2019, which included CHC data (McCormick et al. 2022). Thus, individuals treated with DAAs should be removed, with the sustained virologic response ($SVR$) in the general population estimated to be 96.7% (95% CI: 95.4% to 98.1%) (Lampertico et al. 2020). Similarly to the procedure described in the previous subsections, the CHC prevalence among non-PWID, adjusted for DAAs, can be estimated by

$$\pi_{non} = \frac{N_{15,79}\rho_{non}\tilde{\pi}_{non} - 1082SVR}{N_{15,79}\rho_{non}}. \quad (6)$$

where $\tilde{\pi}_{non}$ denotes the CHC prevalence estimate based solely on McCormick et al. (2022) and 1,082 is the number of non-PWID individuals treated with DAAs in 2018-19. Since individuals were tested for hepatitis C virus antigen, the results were adjusted for the sensitivity of the method (Freiman et al. 2016) and its specificity.

As $\rho_{rec}$ and $\rho_{ex}$ are likely to be low, $\pi_{non}$ will be the most critical factor for the estimation of the overall CHC prevalence in the population. To adjust for potential heterogeneity between the CHC prevalence estimates from different studies in the non-PWID population in a country, as a sensitivity analysis, we also apply a random-effect meta-analytic approach to pool the CHC prevalence across studies on non-PWID (Lin and Chu 2020). The random-effect approach is carried out only when at least 3 studies are available in the general population of a certain country, and the estimates are compared with the corresponding estimates from the approach assuming no heterogeneity (similar to a fixed-effect meta-analysis). If the estimates are inconsistent, possible explanations are investigated and discussed. If there is substantial heterogeneity between studies, it would help more to explore the causes; for example, following national focal point suggestions, some studies could be excluded to reduce heterogeneity.

## Sensitivity analysis including migrants from endemic countries

A sensitivity analysis including data for the viremic population among migrants based on the report of the ECDC group (European Centre for Disease Prevention and Control 2016) was carried out. Migrants from endemic countries represent 9% of the adult population in Ireland (Table 8 in European Centre for Disease Prevention and Control (2016)), with the respective CHC prevalence being equal to $\pi_{mig} = 1.7\%$ (Table 9 in European Centre for Disease Prevention and Control (2016)). The overall CHC prevalence is now estimated by

$$\pi = \pi_{rec}\rho_{rec} + \pi_{ex}\rho_{ex} + \pi_{mig}\rho_{mig} + \pi_{non}\rho_{non},$$

where $\rho_{rec} + \rho_{ex} + \rho_{mig} + \rho_{non} = 1$. Due to lack of data on the proportion of migrants among individuals treated with DAAs, we did not perform a treatment adjustment in this sensitivity analysis.

# RESULTS

The above-mentioned approach was used to estimate the prevalence of CHC in 2019 (considering ages between 15-79 years: 3,732,658).

The aggregated data used by our approach are briefly presented in Table 1. The multi-state Markov model was calibrated on the estimated number of ever PWID reported in the paper of Carew et al. (2017) for 2014 after removing deceased individuals. In Ireland, there was 1 study on pregnant women in 2016-2019, which included CHC data (McCormick et al. 2022). To estimate the CHC prevalence in the general population (primarily non-PWID), we used a fixed-effect meta-analytic approach, with the corresponding results, when information on DAAs is ignored, presented in Table 2. However, approximately 4,829 individuals were treated with DAAs from 2014 to 2019 in Ireland, of whom, 2,960 were recent or ex-PWID. In this report, among the 2,960 ever PWID treated individuals, it is assumed that the proportions of recent PWID and ex-PWID are equal to $\frac{\text{Pr(Recent PWID|CHC)}}{\text{Pr(Recent PWID|CHC)+Pr(Ex-PWID|CHC)}} \approx$ 24.4% (95% CI:17.2%-31.79%) and $\frac{\text{Pr(Ex-PWID|CHC)}}{\text{Pr(Recent PWID|CHC)+Pr(Ex-PWID|CHC)}} \approx$ 75.6% (95% CI:68.21%-82.8%), respectively, as estimated by our model when information on DAAs is ignored (Table 2).

The corresponding results accounting for the DAA uptake are presented in Table 3. The prevalence of recent and ex-PWID was low in Ireland (about 0.13% and 0.29%, respectively) corresponding to 4,680 (95% CI: 4,175-5,150) recent PWID and 10,700 (95% CI: 9,905-11,510) ex-PWID in the population. However, the CHC prevalence in these groups was substantial (Table 3) being 25.41% and 34.35%, respectively. This translates to 1,186 (95% CI: 750-1,714) and 3,676 (95% CI: 3,008-4,376) recent and ex-PWID aged 15-79 living with CHC infection in Ireland in 2019. The CHC prevalence in the general population was 0.08% (95% CI: 0%-0.22%), much lower than that of the high-risk groups. Taking all pieces of information into account, the

overall CHC prevalence in Ireland in 2019 was equal to 0.21% (95% CI: 0.13%-0.35%), which corresponds to 7,844 (95% CI: 4,711-13,035) individuals aged 15-79 years with CHC infection.

The results from our model including migrants from endemic countries as a separate group are presented in Table 4. However, this analysis comes with possible limitations; that is, including migrants as a separate group is valid only if migrants do not overlap with the remaining groups (recent PWID, ex-PWID, and non-PWID). Thus, if migrants do participate proportionally in the study(ies) in the general population, this analysis may result in biased overall CHC estimates (most probably in higher CHC prevalence estimates).

## Limitations and Discussion

The analyses reported in this document have certain limitations. First, although a recent seroprevalence study exists (Garvey et al. 2017), as mentioned by the national focal points, the study of Garvey et al. (2017) should not be used to inform the CHC prevalence in the non-PWID population. While this study tried to exclude specimens identified as being from high-risk settings, it was not specifically a non-PWID study and there were no data on risk factors for HCV for the specimens included in the sampling frame or the specimens that tested positive. As explained by the national focal point, if this study is used for estimates, it is likely to be closer to a national estimate for CHC infection (2014-2016, 0.57%) including PWID, rather than excluding PWID. Instead, we used data from two large Dublin-based maternity hospitals that tested leftover blood samples from individuals who were not selected for HCV testing based on a risk factor assessment (McCormick et al. 2022). Thus, PWIDs (current or ex) have been most likely excluded from the population studied in McCormick et al. (2022). However, since the prevalence of HCV is expected to be higher in the Greater Dublin area compared to the rest of Ireland, results from McCormick et al. (2022) may be an overestimate of the national prevalence of CHC in females of child-bearing age. Thus, due to the lack of a high-quality representative seroprevalence study with available information on the injection status, the results of this report should be interpreted with caution as it is unclear how good the study of McCormick et al. (2022) is as a proxy for the general population.

To inform the CHC among PWID (recent and ex), we utilize data from customized audit forms sent to general practitioners (GP) in 11 addiction treatment centers (Murphy, Thornton, and Bourke 2018) and data among ever PWID receiving opioid substitution therapy in primary care

practices in Ireland (Murtagh et al. 2018), with available data up to 2016. However, it is not clear how representative the data reported in Murphy, Thornton, and Bourke (2018) and Murtagh et al. (2018) are of the PWID population.

Most studies included in this report took place before 2019, thus, some CHC-positive individuals have been treated and cured since then. To adjust for that, taking the variability in the SVR rates into account, we stochastically removed cured individuals from the CHC-positive population. Although the numbers of DAA-treated ever PWID and non-PWID were available, the exact numbers of recent and ex-PWID treated individuals were not. In this report, we assumed that these proportions are proportional to the corresponding ones among CHC-positive individuals. However, this assumption may not be so accurate. Another potential limitation is that the reinfection risk (mostly among PWIDs) was not considered.

# TABLES AND FIGURES

*Table 1. Data (real) contributing to the estimation of CHC prevalence in Ireland in 2019.*

| Parameter | Estimate (95%CI) | Number (95%CI) | Numerator | Denominator | Notes | Year of study |
|---|---|---|---|---|---|---|
| $\rho_{rec}$ | | 4,680 (4,175-5,150) | | | Method based on McDonald et al. | 2019 |
| $\rho_{ex}$ | | 10,700 (9,905-11,510) | | | Method based on McDonald et al. | 2019 |
| $\pi_{rec}$ | | | 16†† | 40 | (Murphy, Thornton, and Bourke 2018) | up to 2016 |
| $\pi_{ex}$ | | | 129†† | 242 | (Murphy, Thornton, and Bourke 2018) | up to 2016 |
| $\pi_{ever}$ | | | 60†† | 127 | (Murtagh et al. 2018) | up to 2016 |
| $\pi_{non}$ † | | | 5 | 4,655 | (McCormick et al. 2022); Risk of bias=NA | 2016-2019 |

*Notes*: Although it looks counter-intuitive, **a higher risk of bias score denotes a higher-quality study** (range from 0 to 6); † Adjusted for sensitivity and specificity of the Antigen testing (Freiman et al. 2016); †† Number of anti-HCV positive individuals multiplied by the proportion of CHC-positive among anti-HCV positive individuals with available CHC data.

*Table 2. Results from the method assuming no heterogeneity in the estimation of the CHC prevalence in the general population from different studies (i.e., similar to a fixed-effect meta-analysis).*

| Parameter | Posterior median | Posterior sd | LB (2.5%) | UB (97.5%) |
|---|---|---|---|---|
| $\rho_{rec}$ (%) | 0.13 | 0.01 | 0.11 | 0.14 |
| $\rho_{ex}$ (%) | 0.29 | 0.01 | 0.27 | 0.31 |
| $\pi_{rec}$ (%) | 39.04 | 6.71 | 26.43 | 52.73 |
| $\pi_{ex}$ (%) | 52.79 | 2.91 | 47.18 | 58.57 |
| $\pi_{non}$ (%) | 0.11 | 0.05 | 0.03 | 0.24 |
| $\pi$ (%) | 0.31 | 0.06 | 0.23 | 0.44 |
| Number with CHC | 11,543 | 2,074 | 8,521 | 16,569 |
| Pr(Recent PWID\|CHC) (%) | 15.69 | 3.67 | 9.48 | 23.66 |
| Pr(Ex-PWID\|CHC) (%) | 48.87 | 8.39 | 33.8 | 66.16 |
| Pr(Non-PWID\|CHC) (%) | 35.19 | 10.74 | 13.61 | 54.93 |

*Notes*: Information on DAAs is not taken into account.

*Abbreviations*: $\rho_{rec}$, prevalence of recent PWID (proportion of the population that is recent PWID); $\rho_{ex}$, prevalence of ex-PWID (proportion of the population that is ex-PWID); $\pi_{rec}$, CHC prevalence among recent PWID; $\pi_{ex}$, CHC prevalence among ex-PWID; $\pi_{non}$, CHC prevalence among non-PWID; $\pi$ overall CHC prevalence in Ireland; LB, Lower Boundary; UP, Upper Boundary

*Table 3. Results from the method assuming no heterogeneity in the estimation of the CHC prevalence in the general population from different studies (i.e., similar to a fixed-effect meta-analysis).*

| Parameter | Posterior median | Posterior sd | LB (2.5%) | UB (97.5%) |
|---|---|---|---|---|
| $\rho_{rec}$ (%) | 0.13 | 0.01 | 0.11 | 0.14 |
| $\rho_{ex}$ (%) | 0.29 | 0.01 | 0.27 | 0.31 |
| $\pi_{rec}$ (%) | 25.41 | 4.98 | 16.25 | 35.73 |
| $\pi_{ex}$ (%) | 34.35 | 2.76 | 28.93 | 39.82 |
| $\pi_{non}$ (%) | 0.08 | 0.06 | 0 | 0.22 |
| $\pi$ (%) | 0.21 | 0.06 | 0.13 | 0.35 |
| Number with CHC | 7,844 | 2,138 | 4,711 | 13,035 |
| Pr(Recent PWID\|CHC) (%) | 15.09 | 4.74 | 7.98 | 26.44 |
| Pr(Ex-PWID\|CHC) (%) | 47 | 12.41 | 27.89 | 76.27 |
| Pr(Non-PWID\|CHC) (%) | 37.69 | 16.2 | 0.22 | 62.99 |

*Notes*: The number of individuals treated with DAAs is taken into account.

*Abbreviations*: $\rho_{rec}$, prevalence of recent PWID (proportion of the population that is recent PWID); $\rho_{ex}$, prevalence of ex-PWID (proportion of the population that is ex-PWID); $\pi_{rec}$, CHC prevalence among recent PWID; $\pi_{ex}$, CHC prevalence among ex-PWID; $\pi_{non}$, CHC prevalence among non-PWID; $\pi$ overall CHC prevalence in Ireland; LB, Lower Boundary; UP, Upper Boundary

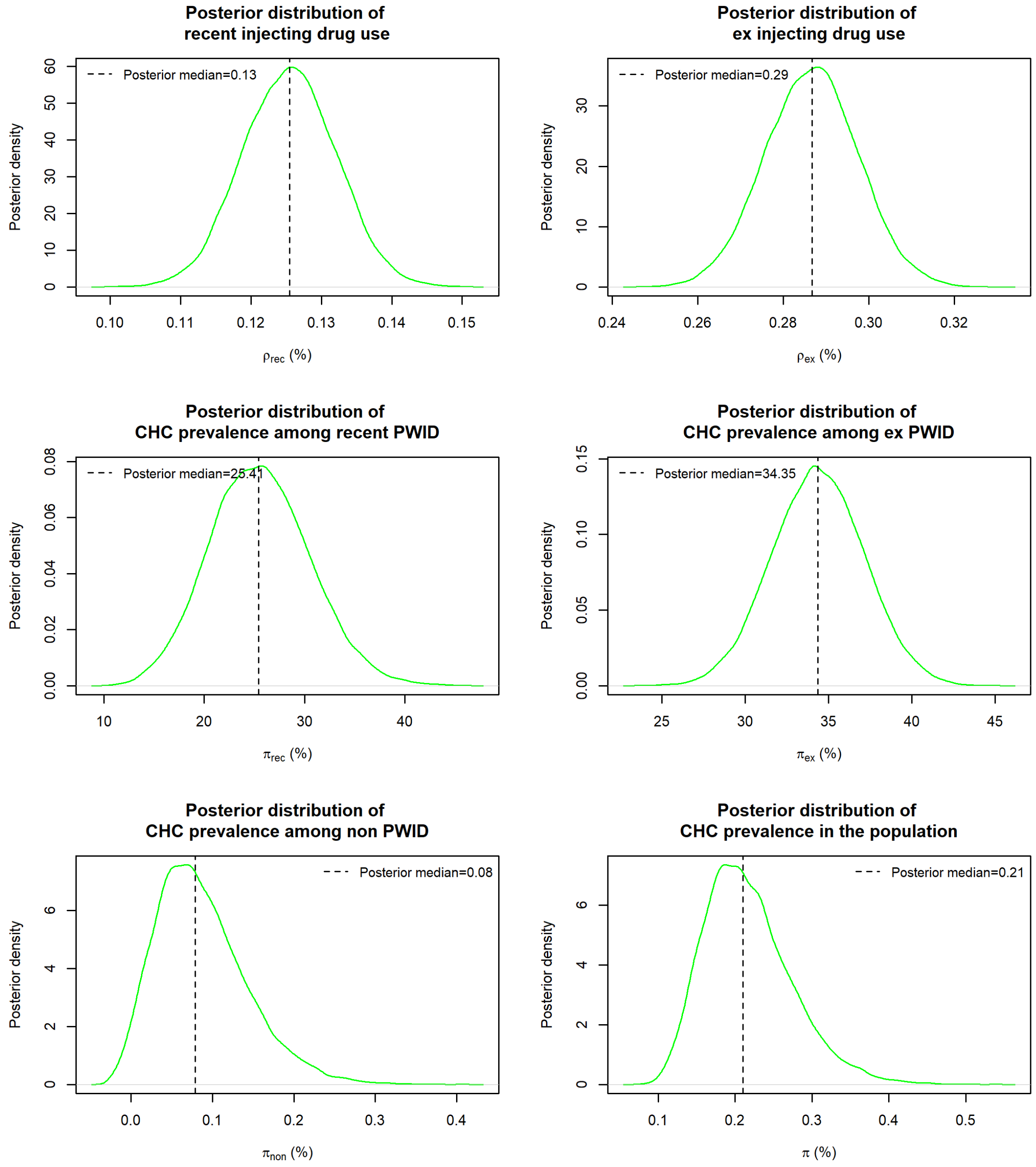


*Figure 1. Posterior distributions of the key model parameters using the approach that assumes no heterogeneity in the estimation of the CHC prevalence in the general population (primarily non-PWID) from different studies (i.e., similar to a fixed-effect meta-analysis), with the number of individuals treated with DAA taken into account.*

*Table 4. Results from the method assuming no heterogeneity in the estimation of the CHC prevalence in the general population (primarily non-PWID) from different studies (i.e., similar to a fixed-effect meta-analysis) including migrants from endemic countries as a separate group.*

| Parameter | Posterior median | Posterior sd | LB (2.5%) | UB (97.5%) |
|---|---|---|---|---|
| $\rho_{rec}$ (%) | 0.13 | 0.01 | 0.11 | 0.14 |
| $\rho_{ex}$ (%) | 0.29 | 0.01 | 0.26 | 0.31 |
| $\rho_{mig}$ (%) | 9 | 0 | 9 | 9 |
| $\pi_{rec}$ (%) | 39 | 6.7 | 26.49 | 52.7 |
| $\pi_{ex}$ (%) | 52.8 | 2.93 | 46.94 | 58.48 |
| $\pi_{mig}$ (%) | 1.7 | 0.42 | 0.87 | 2.52 |
| $\pi_{non}$ (%) | 0.11 | 0.06 | 0.03 | 0.24 |
| $\pi$ (%) | 0.46 | 0.06 | 0.34 | 0.59 |
| Number with CHC | 16,986 | 2,401 | 12,779 | 22,191 |
| Pr(Recent PWID\|CHC) (%) | 10.73 | 2.35 | 6.8 | 16.02 |
| Pr(Ex-PWID\|CHC) (%) | 33.22 | 4.95 | 25.01 | 44.35 |
| Pr(Mig\|CHC) (%) | 33.47 | 6.7 | 19.51 | 45.73 |
| Pr(Non-PWID\|CHC) (%) | 21.85 | 8.39 | 7.17 | 39.4 |

*Notes*: The number of individuals treated with DAAs is not taken into account.

*Abbreviations*: $\rho_{rec}$, prevalence of recent PWID (proportion of the population that is recent PWID); $\rho_{ex}$, prevalence of ex-PWID (proportion of the population that is ex-PWID); $\rho_{mig}$, prevalence of migrants from endemic countries (proportion of the population that belongs to this group); $\pi_{rec}$, CHC prevalence among recent PWID; $\pi_{ex}$, CHC prevalence among ex-PWID; $\pi_{mig}$, CHC prevalence among migrants from endemic countries; $\pi_{non}$, CHC prevalence among non-PWID; $\pi$ overall CHC prevalence in Ireland; LB, Lower Boundary; UP, Upper Boundary

# APPENDIX

## Fit of the multi-state Markov model

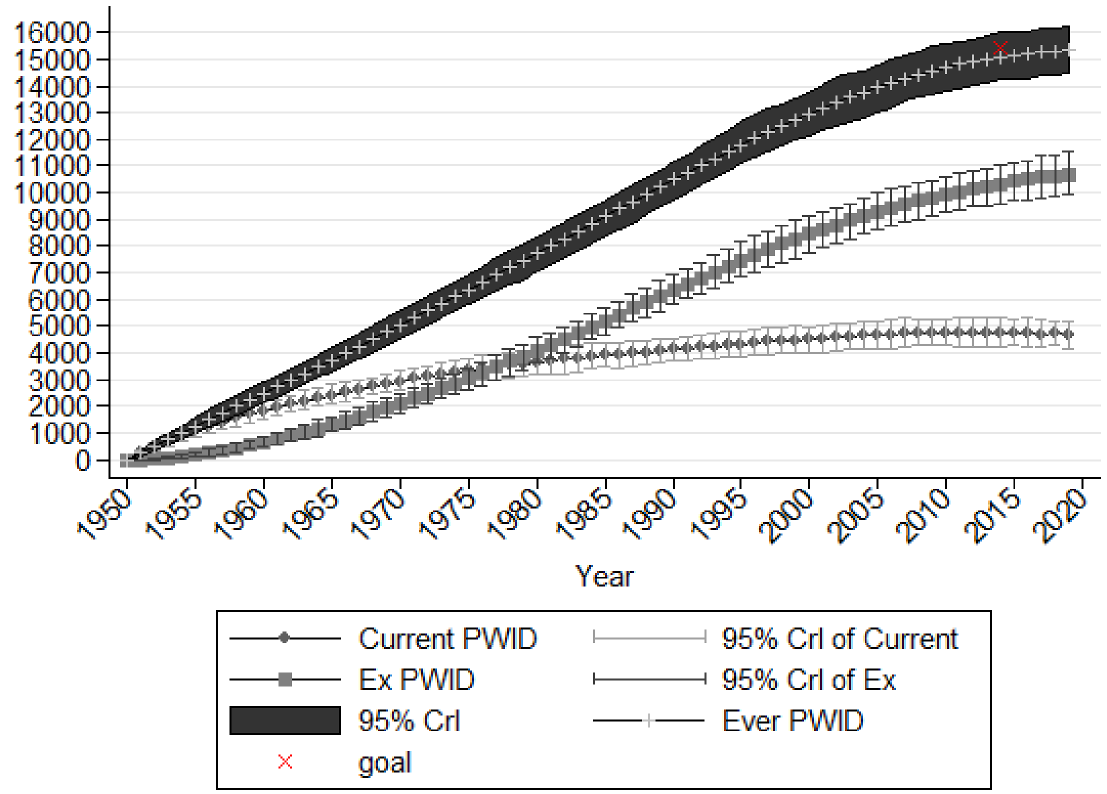


*Figure 2: Model predictions for the numbers of current and ex-PWID (people who inject drugs). The solid black line and shaded grey error bars show the median and 95% credible intervals (95% CrI) for the model projections. For comparison, asterisks indicate the observed country's data.*

## Stan code for Bayesian multiparameter evidence synthesis

```
data {
  int<lower=0> N1579; // Population of 15-79 in `Country`
  int<lower=1> Knon; // Number of studies in the ECDC data for `Country`
  int<lower=1> Kever; // Number of studies in the EMCDDA data for ever users in `Country`
  int<lower=1> Kcur; // Number of studies for recent PWID in `Country`
  int<lower=1> Kex; // Number of studies for ex-PWID in `Country`
  int<lower=1> NDAA_non; // Total number of DAAs in non-PWID from 2014 to 2019
  int<lower=1> NDAA_PWID; // Total number of DAAs among PWIDs from 2014 to 2019

  real p_cur_mean; // Prior mean for the prevalence of current use in `Country`
  real<lower=0> p_cur_sd; // Prior sd for the prevalence of current use in `Country`

  real p_ex_mean; // Prior mean for the prevalence of ex-use in `Country`
  real<lower=0> p_ex_sd; // Prior sd for the prevalence of ex-use in `Country`

  int<lower=0> Nst_CHC_cur[Kcur]; // Number of individuals in the study estimating CHC among recent PWID
  int<lower=0> Yst_CHC_cur[Kcur]; // Number of HCV+ in the study estimating CHC among recent PWID

  int<lower=0> Nst_CHC_ex[Kex]; // Number of individuals in the study estimating CHC among ex-PWID
  int<lower=0> Yst_CHC_ex[Kex]; // Number of HCV+ in the study estimating CHC among ex-PWID
```

```
  int<lower=0> Nst_CHC_ever[Kever]; // Number of individuals in the study 
estimating CHC among ever PWID
  int<lower=0> Yst_CHC_ever[Kever]; // Number of HCV+ in the study estima
ting CHC among ever PWID

  int<lower=0> Nst_CHC_non[Knon]; // Number of individuals in the study e
stimating CHC among non PWID
  int<lower=0> Yst_CHC_non[Knon]; // Number of HCV+ in the study estimati
ng CHC among non PWID

  vector<lower=0>[3] alpha; // parameter of the Diriclet prior

  real HCVclear_mean; // Prior mean for the HCV clearance probability
  real<lower=0> HCVclear_sd; // Prior sd for the HCV clearance probabilit
y

  real<lower=0,upper=1> sens; // Sensitivity of the HCV antigen test
  real<lower=0,upper=1> spec; // Specificity of the HCV antigen test

  real SVR_mean; // Prior mean for the SVR among non-PWID
  real<lower=0> SVR_sd; // Prior sd for the SVR among non-PWID

  real SVR_PWID_mean; // Prior mean for the SVR among PWID
  real<lower=0> SVR_PWID_sd; // Prior sd for the SVR among PWID
}

// Block defining the original parameters
parameters {
  // The parameters to be sampled
  simplex[3] rho; // Prevalence of the three risk groups
  real<lower=0,upper=1> CHCpi_cur; // P(CHC+|Cur)
  real<lower=0,upper=1> CHCpi_ex; // P(CHC+|Ex)
```

```
  real<lower=0,upper=1> CHCpi_non; // P(CHC+|Non)
  real<lower=0,upper=1> SVR; // SVR of DAAs among non-PWID
  real<lower=0,upper=1> SVR_PWID; // SVR of DAAs among PWID

  real<lower=0,upper=1-CHCpi_cur> HCVclear; // Probability of HCV clearan
ce; upper bound = 1-prevalence of chronic HCV
}


transformed parameters {
  // Change scales
  real<lower=0,upper=1> rho_ever;
  real<lower=0,upper=1> CHCpi_ever;
  real<lower=0,upper=1> pi_ever;
  real<lower=0,upper=1> AGpi_non;

  rho_ever = rho[1] + rho[2];
  CHCpi_ever = CHCpi_cur*rho[1]/rho_ever + CHCpi_ex*rho[2]/rho_ever;
  pi_ever = CHCpi_ever/(1-HCVclear);
  AGpi_non = sens*CHCpi_non + (1-spec)*(1-CHCpi_non);
}


// Binomial regression model
model {
  // Priors
  rho ~ dirichlet(alpha);

  ////////////////////////////////
  // Likelihood contributions //
  ////////////////////////////////
```

```
  // Probability of HCV clearance
  HCVclear ~ normal(HCVclear_mean,HCVclear_sd);

  // Prevalence of current use
  rho[1] ~ normal(p_cur_mean,p_cur_sd);

  // Prevalence of ex-use
  rho[2] ~ normal(p_ex_mean,p_ex_sd);

  // Prevalence of CHC among recent PWID
  Yst_CHC_cur ~ binomial(Nst_CHC_cur,CHCpi_cur);

  // Prevalence of CHC among ex-PWID
  Yst_CHC_ex ~ binomial(Nst_CHC_ex,CHCpi_ex);

  // Prevalence of CHC among ever PWID
  Yst_CHC_ever ~ binomial(Nst_CHC_ever,CHCpi_ever);

  // HCV+ among non
  Yst_CHC_non ~ binomial(Nst_CHC_non,AGpi_non);

  // SVR of DAAs among non-PWID
  SVR ~ normal(SVR_mean,SVR_sd);

  // SVR of DAAs among PWID
  SVR_PWID ~ normal(SVR_PWID_mean,SVR_PWID_sd);
}


generated quantities {
  // Functions of parameters
  real<lower=0,upper=100> overalCHC;
```

```
  real logit_CHCpi_cur;
  real logit_CHCpi_ex;
  real logit_CHCpi_non;
  real logit_rho_cur;
  real logit_rho_ex;
  real logit_rho_non;
  real<lower=0,upper=1> pEverGivenCHC;
  real<lower=0,upper=1> pCurGivenCHC;
  real<lower=0,upper=1> pExGivenCHC;
  real<lower=0,upper=1> pNonGivenCHC;
  real logit_HCVclear;
  real<lower=0> NumberCHC;

  real CHCDAApi_non; // P(CHC+|Non) adjusted for DAA
  real CHCDAApi_cur; // P(CHC+|Cur) adjusted for DAA
  real CHCDAApi_ex; // P(CHC+|Ex) adjusted for DAA
  real CHCDAApi_ever; // P(CHC+|Ever) adjusted for DAA

  real<lower=0,upper=100> overalCHC_DAA;
  real pEverGivenCHC_DAA;
  real pCurGivenCHC_DAA;
  real pExGivenCHC_DAA;
  real pNonGivenCHC_DAA;
  real<lower=0> NumberCHC_DAA;

  // Overall HCV prevalence
  overalCHC = 100*(rho[1]*CHCpi_cur + rho[2]*CHCpi_ex + rho[3]*CHCpi_non)
;
  pEverGivenCHC = CHCpi_ever*rho_ever/(overalCHC/100);
  pCurGivenCHC = CHCpi_cur*rho[1]/(overalCHC/100);
  pExGivenCHC = CHCpi_ex*rho[2]/(overalCHC/100);
  pNonGivenCHC = CHCpi_non*(1-rho_ever)/(overalCHC/100);
```

```
  NumberCHC = round(overalCHC*N1579/100);

  CHCDAApi_non = ( N1579*rho[3]*CHCpi_non - NDAA_non*SVR)/(N1579*rho[3]);
  CHCDAApi_cur = ( N1579*rho[1]*CHCpi_cur - NDAA_PWID*(pCurGivenCHC/(pCur
GivenCHC+pExGivenCHC))*SVR_PWID)/(N1579*rho[1]);
  CHCDAApi_ex = ( N1579*rho[2]*CHCpi_ex - NDAA_PWID*(pExGivenCHC/(pCurGiv
enCHC+pExGivenCHC))*SVR_PWID)/(N1579*rho[2]);
  CHCDAApi_ever = CHCDAApi_cur*rho[1]/rho_ever + CHCDAApi_ex*rho[2]/rho_e
ver;

  overalCHC_DAA = 100*(rho[1]*CHCDAApi_cur + rho[2]*CHCDAApi_ex + rho[3]*
CHCDAApi_non);
  pEverGivenCHC_DAA = CHCDAApi_ever*rho_ever/(overalCHC_DAA/100);
  pCurGivenCHC_DAA = CHCDAApi_cur*rho[1]/(overalCHC_DAA/100);
  pExGivenCHC_DAA = CHCDAApi_ex*rho[2]/(overalCHC_DAA/100);
  pNonGivenCHC_DAA = CHCDAApi_non*(1-rho_ever)/(overalCHC_DAA/100);
  NumberCHC_DAA = round(overalCHC_DAA*N1579/100);

  logit_rho_cur = logit(rho[1]);
  logit_rho_ex = logit(rho[2]);
  logit_rho_non = logit(rho[3]);
  logit_CHCpi_cur = logit(CHCpi_cur);
  logit_CHCpi_ex = logit(CHCpi_ex);
  logit_CHCpi_non = logit(CHCpi_non);
  logit_HCVclear = logit(HCVclear);
}
```

## Multi-state Markov model

```
#include <stdio.h>
#include <stdlib.h>
#include <string.h>
#include <math.h>
#include <time.h>

#define loops 100
int populationPerYearAndAge[2021][66];
double deathRatePerAge[66];
int totalPopulationPerYear[2021];

FILE *F_DeathRate=fopen("`Country`-deathRates.txt","r");
FILE *F_Population=fopen("`Country`-population.txt","r");
FILE *out;

double pa_start = 0.00012;                      // Setting Variable
double pa_step  = 0.000002;                     // Setting Variable
double pa_stop  = 0.000123;                     // Setting Variable

double pa;
double pg = 1.0/11.7;    // Assumed average active injecting career of  11
.7 years
double pk = 0.004*12.0;      // Relapse rate of 0.004/month

struct people{
    int age;
    int state;
};

char filename[100];
struct people person[50000000];
```

```
long int totalPersons=0;

void setPopulationAge()
{
    for( int j=0; j<71; j++){
        for( int i=0; i<51; i++){
            fscanf(F_Population,"%d",&populationPerYearAndAge[1950+j][15+
i]);
        }
    }
}

void setDeathRate()
{
    for( int i=0; i<51; i++){
        fscanf(F_DeathRate,"%lf",&deathRatePerAge[15+i*5]);
        for( int j=0; j<5; j++){
            deathRatePerAge[15+i*5+j] = deathRatePerAge[15+i*5];
        }
    }
}

void getTotalPopulationPerAge()
{
    for( int j=0; j<71; j++){
        for( int i=0; i<51; i++){
            totalPopulationPerYear[1950+j] += populationPerYearAndAge[195
0+j][15+i];
        }
    }
}
```

```
void printTotalPopulationPerAge()
{
    printf("---- - ----------\n");
    printf("Year - Population\n");
    printf("---- - ----------\n");
    for( int i=1950; i<2021; i++){
        printf("%d - %d\n",i,totalPopulationPerYear[i]);
    }
}

void printDeathRatePerAge()
{
    printf("--- - -----\n");
    printf("Age - Rate\n");
    printf("--- - -----\n");
    for( int i=15; i<65; i++){
        printf("%d - %lf\n",i,deathRatePerAge[i]);
    }
}

void printTotalPersonPerState( int year)
{
    int count[4]= {0,0,0,0};
    for( int i=0; i<totalPersons; i++){
        count[person[i].state]++;
    }
    //printf("%d\t%ld\t%ld\t%ld\t%ld\n",year,count[0],count[1],count[2],c
ount[3]);
    if(year==2014)
        printf("%d\t%ld\t%ld\t%ld\t%ld\t%lf\n",year,count[0],count[1],cou
nt[2],count[3],pa);
    if( year>2009){
```

```
        fprintf(out, "%d\t%ld\t%ld\t%ld\t%ld\n",year,count[0],count[1],co
unt[2],count[3]);
    }
}

void initializePopulation()
{
    int cnt=0;
    for( int j=15; j<65; j++){
        for( int i=0; i<populationPerYearAndAge[1950][j]*100; i++){
            person[cnt].age=j;
            person[cnt].state=0;
            cnt++;
        }
    }
    totalPersons = cnt;
}

bool checkRate(double rate)
{
    if (rate <0.001){
        double k = rand()/(1.00+RAND_MAX);
        if(k<0.001){
            rate = rate*1000;
        }
        else{
            return false;
        }
    }
    double k = rand()/(1.00+RAND_MAX);
    if ( k < rate )
        return true;
```

```
    else
        return false;
}

void changeStatusAndAge()
{
    for( int i=0; i<totalPersons; i++){
        // change state
        double k = rand()/(1.00+RAND_MAX);
        if( person[i].state==0){
            if ( checkRate(pa) &&  person[i].age<40){
                person[i].state=1;
            }
        }
        else if( person[i].state==1){
            if ( k < pg ){
                person[i].state=2;
            }
        }
        else if( person[i].state==2){
            if ( k < pk ){
                person[i].state=1;
            }
        }
        // After their 49th year, all active PWID are assumed to cease in
jecting
        if(person[i].age>49 && person[i].state==1){
            person[i].state=2;
        }
        // After their 64th year, remove
        if(person[i].age>64){
            person[i].state=3;
```

```
        }
        // death rate depending on age and state
        k = rand()/(1.00+RAND_MAX);
        if( person[i].state==0){
            if ( k < deathRatePerAge[person[i].age] ){
                person[i].state=3;
            }
        }
        else if( person[i].state==1){
            if ( k < deathRatePerAge[person[i].age] * 13.0 ){
                person[i].state=3;
            }
        }
        else if( person[i].state==2){
            if ( k < deathRatePerAge[person[i].age] ){
                person[i].state=3;
            }
        }
        // increase age one year
        person[i].age++;
    }
}

void addNewPersons(int year)
{
    // add new person [15 years old and non PWID]
    int cnt=totalPersons;
    for( int i=0; i<populationPerYearAndAge[year+1][15]*100; i++){
// 100 instead of 1000 for the population to reduce execution time
        person[cnt].age=15;
        person[cnt].state=0;
        cnt++;
```

```
    }
    // increase the total number of population
    totalPersons = cnt;
}
int main()
{
    srand(time(NULL));

    setPopulationAge();
    setDeathRate();

    getTotalPopulationPerAge();
    pa = pa_start;
    while ( pa < pa_stop){

        snprintf(filename, 100, "result_%lf.txt",pa);
        out=fopen(filename,"w");

        for( int iter=0; iter<loops ;iter++){
            initializePopulation();
            for( int year=1950; year<2020; year++){
                printTotalPersonPerState(year);
                changeStatusAndAge();
                addNewPersons(year);
            }
        }

        fclose(out);
        pa = pa + pa_step;
    }
```

```
}
```

# REFERENCES

Carew, Anne Marie, Niamh Murphy, Jean Long, Kate Hunter, Suzi Lyons, Cathal Walsh, and Lelia Thornton. 2017. "Incidence of Hepatitis c Among People Who Inject Drugs in Ireland." *Hepatology, Medicine and Policy* 2 (1): 7. https://doi.org/10.1186/s41124-017-0024-1.

European Centre for Disease Prevention and Control. 2016. "Epidemiological Assessment of Hepatitis b and c Among Migrants in the EU/EEA." https://www.ecdc.europa.eu/sites/default/files/media/en/publications/Publications/epidemiological-assessment-hepatitis-B-and-C-among-migrants-EU-EEA.pdf.

European Centre for Disease Prevention and Control (ECDC). 2016. "Systematic Review on Hepatitis b and c Prevalence in the EU/EEA." https://www.ecdc.europa.eu/sites/default/files/media/en/publications/Publications/systematic-review-hepatitis-B-C-prevalence.pdf.

———. 2021. "Hepatitis c Prevalence Database." https://www.ecdc.europa.eu/en/all-topics-z/hepatitis-c/tools/hepatitis-c-prevalence-database.

Freiman, J. Morgan, Trang M. Tran, Samuel G. Schumacher, Laura F. White, Stefano Ongarello, Jennifer Cohn, Philippa J. Easterbrook, Benjamin P. Linas, and Claudia M. Denkinger. 2016. "Hepatitis c Core Antigen Testing for Diagnosis of Hepatitis c Virus Infection." *Annals of Internal Medicine* 165 (5): 345–55. https://doi.org/10.7326/M16-0065.

Garvey, Patricia, Brian O'Grady, Geraldine Franzoni, Maeve Bolger, Katie Irwin Crosby, Jeff Connell, Deirdre Burke, Cillian De Gascun, and Lelia Thornton. 2017. "Hepatitis c Virus Seroprevalence and Prevalence of Chronic Infection in the Adult Population in Ireland: A Study of Residual Sera, April 2014 to February 2016." *Eurosurveillance* 22 (30). https://doi.org/https://doi.org/10.2807/1560-7917.ES.2017.22.30.30579.

Graf, Christiana, Marcus M Mücke, Georg Dultz, Kai-Henrik Peiffer, Alica Kubesch, Patrick Ingiliz, Stefan Zeuzem, Eva Herrmann, and Johannes Vermehren. 2019. "Efficacy of Direct-acting Antivirals for Chronic Hepatitis C Virus Infection in People Who Inject Drugs or Receive Opioid Substitution Therapy: A Systematic Review and Meta-analysis." *Clinical Infectious Diseases* 70 (11): 2355–65. https://doi.org/10.1093/cid/ciz696.

Grebely, Jason, Sarah Larney, Amy Peacock, Samantha Colledge, Janni Leung, Matthew Hickman, Peter Vickerman, et al. 2019. "Global, Regional, and Country-Level Estimates of Hepatitis c Infection Among People Who Have Recently Injected Drugs." *Addiction* 114 (1): 150–66. https://doi.org/https://doi.org/10.1111/add.14393.

Hines, Lindsey A., Adam Trickey, Janni Leung, Sarah Larney, Amy Peacock, Louisa Degenhardt, Samantha Colledge, et al. 2020. "Associations Between National Development Indicators and the Age Profile of People Who Inject Drugs: Results from a Global Systematic Review and Meta-Analysis." *The Lancet Global Health* 8 (1): e76–91. https://doi.org/10.1016/S2214-109X(19)30462-0.

Lampertico, Pietro, Jose A. Carrión, Michael Curry, Juan Turnes, Markus Cornberg, Francesco Negro, Ashley Brown, et al. 2020. "Real-World Effectiveness and Safety of Glecaprevir/Pibrentasvir for the Treatment of Patients with Chronic HCV Infection: A meta-Analysis." *Journal of Hepatology* 72 (6): 1112–21. https://doi.org/10.1016/j.jhep.2020.01.025.

Lin, Lifeng, and Haitao Chu. 2020. "Meta-Analysis of Proportions Using Generalized Linear Mixed Models." *Epidemiology* 31 (5). https://journals.lww.com/epidem/Fulltext/2020/09000/Meta_analysis_of_Proportions_Using_Generalized.16.aspx.

McCormick, C. A., L. Domegan, P. G. Carty, R. Drew, F. M. McAuliffe, O. O'Donohoe, N. White, et al. 2022. "Routine Screening for Hepatitis c in Pregnancy Is Cost-Effective in a Large Urban Population in Ireland: A Retrospective Study." *BJOG: An International Journal of Obstetrics & Gynaecology* 129 (2): 322–27. https://doi.org/10.1111/1471-0528.16984.

McDonald, Scott A., Rosmawati Mohamed, Maznah Dahlui, Herlianna Naning, and Adeeba Kamarulzaman. 2014. "Bridging the Data Gaps in the Epidemiology of Hepatitis c Virus Infection in Malaysia Using Multi-Parameter Evidence Synthesis." *BMC Infectious Diseases* 14 (1): 564. https://doi.org/10.1186/s12879-014-0564-6.

Murphy, Niamh, Lelia Thornton, and Margaret Bourke. 2018. "Audit of Hepatitis c Testing and Referral in Addiction Treatment Centres in CHO Area 7." http://hdl.handle.net/10147/623614.

Murtagh, Ross, Davina Swan, Eileen O'Connor, Geoff McCombe, John S. Lambert, Gordana Avramovic, and Walter Cullen. 2018. "Hepatitis c Prevalence and Management Among Patients Receiving Opioid Substitution Treatment in General Practice in Ireland: Baseline Data from a Feasibility Study." *Interact J Med Res* 7 (2): e10313. https://doi.org/10.2196/10313.

Presanis, Anne M., O. Noel Gill, Timothy R. Chadborn, Caterina Hill, Vivian Hope, Louise Logan, Brian D. Rice, Valerie C. Delpech, A. E. Ades, and Daniela De Angelis. 2010. "Insights into the Rise in HIV Infections, 2001 to 2008: A Bayesian Synthesis of Prevalence Evidence." *AIDS* 24 (18). https://journals.lww.com/aidsonline/Fulltext/2010/11270/Insights_into_the_rise_in_HIV_infections,_2001_to.12.aspx.

Sweeting, M. J., D. De Angelis, M. Hickman, and A. E. Ades. 2008. "Estimating hepatitis C prevalence in England and Wales by synthesizing evidence from multiple data sources. Assessing data conflict and model fit." *Biostatistics* 9 (4): 715–34. https://doi.org/10.1093/biostatistics/kxn004.

Tan, Sarah, Susanna Makela, Daliah Heller, Kevin Konty, Sharon Balter, Tian Zheng, and James H. Stark. 2018. "A Bayesian Evidence Synthesis Approach to Estimate Disease Prevalence in Hard-to-Reach Populations: Hepatitis c in New York City." *Epidemics* 23: 96–109. https://doi.org/https://doi.org/10.1016/j.epidem.2018.01.002.

Thein, Hla-Hla, Qilong Yi, Gregory J. Dore, and Murray D. Krahn. 2008. "Estimation of Stage-Specific Fibrosis Progression Rates in Chronic Hepatitis c Virus Infection: A Meta-Analysis and Meta-Regression." *Hepatology* 48 (2): 418–31. https://doi.org/https://doi.org/10.1002/hep.22375.

Veen, Maaike G. van, Anne M. Presanis, Stefano Conti, Maria Xiridou, Annemarie R. Stengaard, Martin C. Donoghoe, Ard I. van Sighem, Marianne A. van der Sande, and Daniela De Angelis. 2011. "National Estimate of HIV Prevalence in the Netherlands: Comparison and Applicability of Different Estimation Tools." *AIDS* 25 (2). https://journals.lww.com/aidsonline/Fulltext/2011/01140/National_estimate_of_HIV_prevalence_in_the.14.aspx.

# Estimating the prevalence of chronic hepatitis C infection (CHC) in Italy using Bayesian multiparameter evidence synthesis

12/03/2023

## Table of Contents

Introduction ........ 2
Methods ........ 2
Prevalence of ex-PWID ........ 3
Prevalence of recent PWID ........ 3
Prevalence of CHC among recent PWID ........ 4
Prevalence of CHC among ex-PWID ........ 4
Prevalence of CHC among non-PWID ........ 5
Results ........ 7
Limitations ........ 8
Tables and Figures ........ 9
Appendix ........ 15
Fit of the multi-state Markov model ........ 15
Stan code for Bayesian multiparameter evidence synthesis ........ 16
Multi-state Markov model ........ 23
References ........ 31

# INTRODUCTION

Patients with chronic hepatitis C (HCV) infection often remain asymptomatic for decades and even for life, but a proportion of them develop active chronic hepatitis, which is a progressive disease (Thein et al. 2008). Because of the largely asymptomatic nature of HCV infection, notification data reflect national screening and testing practices and do not give accurate insights into the prevalence of HCV infection. Although there are studies that have estimated the prevalence of HCV antibodies (anti-HCV) in specific groups such as the general population, first-time blood donors, or people who inject drugs (PWID) at drug treatment centers, those alone cannot be combined to produce national estimates unless some additional information regarding the composition of each HCV risk group and its prevalence in the population are known (Sweeting et al. 2008; McDonald et al. 2014; Tan et al. 2018).

# METHODS

Bayesian multiparameter evidence synthesis (MPES) has been a popular approach to formally estimate anti-HCV or HIV prevalence (Sweeting et al. 2008; McDonald et al. 2014; Tan et al. 2018; Presanis et al. 2010; Veen et al. 2011). In this project, we extend this method to estimating the CHC prevalence, with the population (15-79 years) split into three main non-overlapping risk groups, i.e., recent PWID (those who have injected in the last year), ex-PWID, and non-PWID. A unified model is assumed including parameters associated with the CHC prevalence of recent, ex-PWID, and non-PWID (denoted by $\pi_{rec}$, $\pi_{ex}$, and $\pi_{non}$, respectively) and the prevalence of these risk groups in the population (denoted by $\rho_{rec}$, $\rho_{ex}$, and $\rho_{non}$, respectively; $\rho_{rec} + \rho_{ex} + \rho_{non} = 1$). To estimate the CHC prevalence in the whole population, $\pi$, we used the formula:

$$\pi = \pi_{rec}\rho_{rec} + \pi_{ex}\rho_{ex} + \pi_{non}\rho_{non} \quad (1)$$

Bayesian synthesis considers all available data, allowing for an evidence-based estimation of CHC prevalence in the population, with inherent uncertainty properly accounted for. Our aim is to estimate the CHC prevalence for the total (15-79) population of Italy in 2019 using sources of information over the last decade (2010-2019).

## Prevalence of ex-PWID

Data on the prevalence of ex-PWID are sparse and generally often unreliable. To overcome this limitation, we apply the method proposed by McDonald et al. (2014), which requires simulating a multi-state Markov model representing the non-PWID, recent PWID, and ex-PWID. In the multi-state Markov model, the simulation starts in 1950 and examines the population aged 15-79 years old. A certain number of 15-year-old individuals enter the simulation process each year. These numbers of 15-year-old individuals are obtained from the United Nations Statistic Division UNSD, being different for each country. The ageing of the population is also taken into account. Country-specific overall mortality data are retrieved from the Life Tables of the World Health Organization WHO. The duration of injecting career is also country-specific and obtained from the paper of Hines et al. (2020).

To estimate the number of ex-PWID, the model can be calibrated on the number of recent PWID provided in the EMCDDA barometer or the EMCDDA statistical bulletin (the code of the Markov model can be found in the Appendix). If there are no available data in the barometer, estimates from the systematic review of Grebely et al. (2019) could be used. Currently, the multi-state Markov model was calibrated on the observed number of recent PWID in 2019 provided by the Italian public services for drug addiction through personal communication.

After applying the model for Italy, we compute the number (and the corresponding Confidence Interval - CI) of ex-PWID in 2019. The number of ex-PWID is then divided by the population size (15-79 years) in the same year, and a Normal distribution, constrained such that $\rho_{ex} \in (0,1)$, is assumed for $\rho_{ex}$. The standard deviation of this distribution is specified to approximately correspond to the respective CI, i.e. $\left(UB_{\rho_{ex}} - LB_{\rho_{ex}}\right)/(2 \times 1.96)$, where $UB_{\rho_{ex}}$ and $LB_{\rho_{ex}}$ denote the upper and lower limit of the CI.

## Prevalence of recent PWID

The number of recent PWID in Italy in 2019 is also projected by the multi-state Markov model described in the previous subsection. To estimate $\rho_{rec}$, the number of recent PWID is divided by the population size (15-79 years). Similar to the previous subsection, a Normal distribution, constrained such that $\rho_{rec} \in (0,1)$, is assumed for $\rho_{rec}$, with the standard deviation specified to correspond to the CI obtained from the multi-state Markov model.

## Prevalence of CHC among recent PWID

The prevalence of CHC among recent PWID was informed by CHC prevalence data reported in the paper of Grebely et al. (2019). However, some people may have been treated with direct-acting antivirals (DAAs), with the sustained virologic response ($SVR_{PWID}$) among PWID estimated to be 88% (95% CI: 80% to 93%) (Graf et al. 2019). Based on the information provided in Kondili et al. (2021) (Table 2 of the supplementary appendix), the number of individuals treated with DAAs up to 2019 is equal to 163,955. Even though the exact proportions of each risk group among those treated with DAAs, i.e. $\rho_{rec|DAA}$, $\rho_{ex|DAA}$, and $\rho_{non|DAA}$ ($\rho_{rec|DAA} + \rho_{ex|DAA} + \rho_{non|DAA} = 1$), are not currently available in Italy, information on the proportion of ever PWID among those treated could be inferred indirectly. Specifically, based on information provided by the national focal points, the proportions of treated patients with genotype 3 and mixed genotype were 16% and 5%, respectively. Therefore, in this report, we assume that the proportion of ever PWID among those treated with DAAs ($\rho_{ever|DAA}$) is uniformly distributed from 16% to 21% (equivalently $\rho_{non|DAA} = 1 - \rho_{ever|DAA}$). Moreover, among treated ever PWID, we assume that treatment is distributed proportionally to the proportion of recent and ex-PWID among CHC-positive individuals, Pr(Recent PWID|CHC) and Pr(Ex-PWID|CHC), as estimated by our model when the DAA uptake is ignored (Table 2). Formally, we assume that $\rho_{rec|DAA} = \rho_{ever|DAA} \frac{\text{Pr(Recent PWID|CHC)}}{\text{Pr(Recent PWID|CHC)+Pr(Ex-PWID|CHC)}}$, $\rho_{ex|DAA} = \rho_{ever|DAA} \frac{\text{Pr(Ex-PWID|CHC)}}{\text{Pr(Recent PWID|CHC)+Pr(Ex-PWID|CHC)}}$ and $\rho_{non|DAA} = 1 - \rho_{ever|DAA}$. Thus, the CHC prevalence among recent PWID, adjusted for DAAs, can be estimated by

$$\pi_{rec} = \frac{N_{15,79}\rho_{rec}\tilde{\pi}_{rec} - N_{DAA}\rho_{rec|DAA}SVR_{PWID}}{N_{15,79}\rho_{rec}}, \quad (2)$$

where $\tilde{\pi}_{rec}$ denotes the CHC estimate derived solely from the data reported in Grebely et al. (2019).

## Prevalence of CHC among ex-PWID

Information on CHC prevalence among ex-PWID (i.e., $\pi_{ex}$) is difficult to obtain directly. To overcome this, national anti-HCV prevalence data among ever PWID available in the EMCDDA

statistical bulletin were used. However, as these data refer to the anti-HCV prevalence, they should be adjusted similarly to the procedure described in the previous subsection, i.e.

$$\pi_{ever} = \frac{N_{15,79}\rho_{ever}\pi(\text{anti-HCV})_{ever}(1-\rho_{clear}) - N_{DAA}\rho_{ever|DAA}SVR_{PWID}}{N_{15,79}\rho_{ever}}. \quad (3)$$

where $\pi(\text{anti-HCV})_{ever}$ denotes the anti-HCV prevalence among ever PWID, $\rho_{ever} = \rho_{rec} + \rho_{ex}$, $\rho_{clear}$ denotes the spontaneous viral clearance [assumed to be equal to 0.26 (95% CI 0.22–0.29); (Micallef, Kaldor, and Dore 2006)], and $\rho_{ever|DAA}$ the proportion of ever PWID among individuals treated with DAAs. Recall that it is assumed that $\rho_{ever|DAA}$ is uniformly distributed from 0.16 to 0.21.

Then, since an estimate of $\pi_{ever}$ is available, $\pi_{ex}$ can be indirectly estimated since $\pi_{ever}$ is equal to a weighted average of $\pi_{rec}$ and $\pi_{ex}$, as shown by the following formula

$$\pi_{ever} = \frac{\rho_{rec}}{\rho_{ever}}\pi_{rec} + \frac{\rho_{ex}}{\rho_{ever}}\pi_{ex}, \quad (4)$$

or, equivalently,

$$\pi_{ex} = \left(\pi_{ever} - \frac{\rho_{rec}}{\rho_{ever}}\pi_{rec}\right) \times \frac{\rho_{ever}}{\rho_{ex}}. \quad (5)$$

## Prevalence of CHC among non-PWID

To estimate the CHC prevalence among non-PWID in a country (after 2010), the ECDC database (European Centre for Disease Prevention and Control (ECDC) 2021) was used. The ECDC group has carefully and critically collected information from relevant studies across the EU/EEA region in cooperation with the focal points of each country. On the basis of the representativeness of each study and other factors (European Centre for Disease Prevention and Control (ECDC) 2016), a risk of bias score was assigned to each study, with higher values indicating higher-quality studies. When higher-quality studies are available in a country (≥ 4), they are used to estimate the overall CHC prevalence in that country among non-PWID. If there are no higher-quality studies, general population estimates with a lower quality (<4) are pooled. If no general population prevalence estimates are available, data on the prevalence of HCV in pregnant women could be used as a prevalence measure. Finally, if data on pregnant women are lacking too, first-time blood donor studies could be an option. However, if the

national focal point agrees or recommends, estimates from a neighboring country could be used instead, or we could use any other relevant information provided/suggested by the national focal point.

We directly use CHC prevalence data from studies in the general population, if available. In Italy, there were 4 studies on non-PWID of high quality, which included anti-HCV (Parisi et al. 2014; Andriulli et al. 2018) and CHC data (Morisco et al. 2017; Spada et al. 2021). When data refer to anti-HCV prevalence, we first adjust the estimates to get the CHC prevalence based on the spontaneous HCV clearance estimate of 26%. However, apart from spontaneous clearance, some individuals have been treated with DAAs. Furthermore, as raised by national focal points, an issue that is probably unique in Italy is the excess HCV-related mortality among non-PWID even after the introduction of DAAs. Based on the information provided by focal points, there are approximately $N_{deaths}$ = 30,040 HCV-related deaths among non-PWID from 2015 and 2019. Thus, to take DAAs and excess mortality into account, the CHC prevalence among non-PWID is adjusted similarly to the procedure described in the previous subsections, i.e.

$$\pi_{non} = \frac{N_{15,79}\rho_{non}\tilde{\pi}_{non} - N_{DAA}\rho_{non|DAA}SVR - N_{deaths}}{N_{15,79}\rho_{non}}. \quad (6)$$

where $\tilde{\pi}_{non}$ denotes the CHC prevalence among non-PWID when information on DAAs is ignored and $SVR$ is the sustained virologic response of DAAs in the general population, estimated to be 96.7% (95% CI: 95.4% to 98.1%) (Lampertico et al. 2020).

As $\rho_{rec}$ and $\rho_{ex}$ are likely to be low, $\pi_{non}$ will be the most critical factor for the estimation of the overall CHC prevalence in the population. To adjust for potential heterogeneity between the CHC prevalence estimates from different studies in the non-PWID population in a country, as a sensitivity analysis, we also apply a random-effect meta-analytic approach to pool the CHC prevalence across studies on non-PWID (Lin and Chu 2020). The random-effect approach is carried out only when at least 3 studies are available in the general population of a certain country, and the estimates are compared with the corresponding estimates from the approach assuming no heterogeneity (similar to a fixed-effect meta-analysis). If the estimates are inconsistent, possible explanations are investigated and discussed. If there is substantial heterogeneity between studies, it would help more to explore the causes; for example, following national focal point suggestions, some studies could be excluded to reduce heterogeneity.

# RESULTS

The above-mentioned approach was used to estimate the prevalence of CHC in 2019 (considering ages between 15-79 years: 47,648,498).

The aggregated data used by our approach are briefly presented in Table 1. In Italy, there were 4 studies on non-PWID of high quality, which included anti-HCV (Parisi et al. 2014; Andriulli et al. 2018) and CHC data (Morisco et al. 2017; Spada et al. 2021). To estimate the CHC prevalence in the general population (primarily non-PWID), we used a fixed-effect meta-analytic approach, with the corresponding results, when information on DAAs is ignored, presented in Table 2. However, based on data provided by the focal point, approximately 163,955 individuals were treated with DAAs from 2015 to 2019 in Italy, with the estimated proportion (95% CI) of recent PWID, ex-PWID, and non-PWID among the 163,955 treated individuals assumed to be equal to $\rho_{rec|DAA}$ ≈ 3.54% (2.93%-4.23%), $\rho_{ex|DAA}$ ≈ 14.92% (12.98 %-16.94 %), and $\rho_{non|DAA}$ ≈ 81.52% (79.14%-83.87%), respectively (Table 2).

The corresponding results accounting for the DAA uptake are presented in Table 3. The prevalence of recent and ex-PWID was relatively low in Italy (about 0.1% and 0.38%, respectively) corresponding to 45,715 (95% CI: 44,185-47,145) recent PWID and 178,825 (95% CI: 176,325-182,130) ex-PWID in the population. However, the CHC prevalence in these groups was substantial (Table 3), being 32.21% and 34.6%, respectively. This translates to 14,722 (95% CI: 12,876-16,570) and 61,856 (95% CI: 55,621-68,183) recent and ex-PWID aged 15-79 living with CHC infection in Italy in 2019. The CHC prevalence in the general population was 0.81% (95% CI: 0.64%-0.99%), much lower than that of the high-risk groups. Taking all pieces of information into account, the overall CHC prevalence in Italy in 2019 was equal to 0.96% (95% CI: 0.8%-1.15%), which corresponds to 459,000 (95% CI: 379,172-549,698) individuals aged 15-79 years with CHC infection. The corresponding results under a random-effect meta-analysis for the studies in the general population were very similar and are provided in Table 4. However, estimates from the random-effect approach had much higher uncertainty, mainly due to the low number of studies (4) and the considerable heterogeneity in the estimates, which is also reflected in the estimate of the between-study variance (Table 4).

# LIMITATIONS

The analyses reported in this document have certain limitations. First, there is potential selection bias in the general population studies. Although 3 of the studies were scored as high-quality ones by the ECDC group (Parisi et al. 2014; Andriulli et al. 2018; Morisco et al. 2017), as mentioned in a discussion with the national focal points, potential selection issues could not be ruled out. Recall also that the study of Spada et al. (2021) was not scored by the ECDC. For example, even though the study of Andriulli et al. (2018) used random sampling and covered a large portion of the population in Italy, it has several potential biases, with the main being not totally representative of the whole population of Italy, as mentioned by the focal points. Another unique issue in Italy is the excess HCV-related mortality among non-PWID in the years under investigation (i.e. from 2015 to 2019). Unfortunately, the exact number of these deaths is not available, but a reliable estimate was obtained based on relevant information provided by the national focal points. Moreover, the numbers of DAA-treated individuals by risk group (recent PWID, ex-PWID, and non-PWID) have not been officially recorded. However, as suggested by the focal points, the number of treated ever PWID (and subsequently non-PWID) could be indirectly inferred on the basis of the number of treated individuals with genotype 3 or mixed genotype. The distribution of recent PWID and ex-PWID among treated ever PWID is unknown, thus, an assumption should be made. In this report, it is assumed that the proportions of recent PWID and ex-PWID among treated ever PWID are based on the corresponding proportions among CHC-positive individuals.

## TABLES AND FIGURES

*Table 1. Data (real) contributing to the estimation of CHC prevalence in Italy in 2019.*

| Parameter | Estimate (95%CI) | Number (95%CI) | Numerator | Denominator | Notes | Year of study |
|---|---|---|---|---|---|---|
| $\rho_{rec}$ | | 45,715 (44,185-47,145) | | | Method based on McDonald et al. | 2019 |
| $\rho_{ex}$ | | 178,825 (176,325-182,130) | | | Method based on McDonald et al. | 2019 |
| $\pi_{rec}$ | 43.4% (38.8%-48.1%) | | | | Grebely et al. | 2011-2014 |
| $\pi(\text{anti-HCV})_{ever}$ | | | 5,432 | 8,832 | EMCDDA database | 2019 |
| $\pi(\text{anti-HCV})^{1}_{non}$ | | | 27 | 4,507 | ECDC database (Parisi et al.); Risk of bias=4 | 2014 |
| $\pi(\text{anti-HCV})^{2}_{non}$ | | | 100 | 4,858 | ECDC database (Andriulli et al.); Risk of bias=5 | 2014 |

| Parameter | Estimate (95%CI) | Number (95%CI) | Numerator | Denominator | Notes | Year of study |
|---|---|---|---|---|---|---|
| $\pi_{non}^{3}$ | | | 31 | 1,312 | ECDC database (Morisco et al.); Risk of bias=5 | 2015 |
| $\pi_{non}^{4}$ | | | 11 | 1,001 | Country feedback (Spada et al.); Risk of bias=NA | 2018 |

*Notes*: Although it looks counter-intuitive, **a higher risk of bias score denotes a higher-quality study** (range from 0 to 6)

[1] The study of Parisi et al. (2014) did not provide data on injection status, so the overall prevalence of anti-HCV was actually used.

[2] In the study of Andriulli et al. (2018), anti-HCV data among non-PWID (i.e. excluding those who had a history of drug use; see Table 3 in Andriulli et al. 2018) were used because CHC data were not available by injection status.

[3] In the study of Morisco et al. (2017), 3 individuals who reported intravenous drug use (all of whom were anti-HCV negative; see Table II in Morisco et al. (2017)) were excluded from the denominator.

[4] In the study of Spada et al. (2021), 9 persons were CHC-positive: 2 were excluded from both the numerator and denominator due to history of drug use, 4 were added in the numerator because they were anti-HCV positive but CHC-negative due to treatment (recall that a DAA adjustment is performed at a next stage in our approach).

*Table 2. Results from the method assuming no heterogeneity in the estimation of the CHC prevalence in the general population from different studies (i.e., similar to a fixed-effect meta-analysis). Information on direct-acting antivirals (DAAs) is not taken into account.*

| Parameter | Posterior median | Posterior sd | LB (2.5%) | UB (97.5%) |
|---|---|---|---|---|
| $\rho_{rec}$ (%) | 0.1 | 0 | 0.09 | 0.1 |
| $\rho_{ex}$ (%) | 0.38 | 0 | 0.37 | 0.38 |
| $\pi_{rec}$ (%) | 43.44 | 2.36 | 38.78 | 48.08 |
| $\pi_{ex}$ (%) | 46.63 | 1.58 | 43.54 | 49.76 |
| $\pi_{non}$ (%) | 1.14 | 0.09 | 0.97 | 1.33 |
| $\pi$ (%) | 1.29 | 0.09 | 1.12 | 1.48 |
| Number with CHC | 615,117 | 43,705 | 533,346 | 706,040 |
| Pr(Recent PWID\|CHC) (%) | 3.23 | 0.29 | 2.7 | 3.86 |
| Pr(Ex-PWID\|CHC) (%) | 13.55 | 0.96 | 11.84 | 15.6 |
| Pr(Non-PWID\|CHC) (%) | 83.22 | 1.15 | 80.77 | 85.28 |

*Abbreviations*: $\rho_{rec}$, prevalence of recent PWID (proportion of the population that is recent PWID); $\rho_{ex}$, prevalence of ex-PWID (proportion of the population that is ex-PWID); $\pi_{rec}$, CHC prevalence among recent PWID; $\pi_{ex}$, CHC prevalence among ex-PWID; $\pi_{non}$, CHC prevalence among non-PWID; $\pi$ overall CHC prevalence in Italy; LB, Lower Boundary; UP, Upper Boundary

*Table 3. Results from the method assuming no heterogeneity in the estimation of the CHC prevalence in the general population from different studies (i.e., similar to a fixed-effect meta-analysis). The numbers of individuals treated with direct-acting antivirals (DAAs) is taken into account.*

| Parameter | Posterior median | Posterior sd | LB (2.5%) | UB (97.5%) |
|---|---|---|---|---|
| $\rho_{rec}$ (%) | 0.1 | 0 | 0.09 | 0.1 |
| $\rho_{ex}$ (%) | 0.38 | 0 | 0.37 | 0.38 |
| $\pi_{rec}$ (%) | 32.21 | 2.01 | 28.23 | 36.15 |
| $\pi_{ex}$ (%) | 34.6 | 1.76 | 31.18 | 38.02 |
| $\pi_{non}$ (%) | 0.81 | 0.09 | 0.64 | 0.99 |
| $\pi$ (%) | 0.96 | 0.09 | 0.8 | 1.15 |
| Number with CHC | 459,000 | 43,575 | 379,172 | 549,698 |
| Pr(Recent PWID\|CHC) (%) | 3.2 | 0.37 | 2.57 | 4.01 |
| Pr(Ex-PWID\|CHC) (%) | 13.48 | 1.31 | 11.2 | 16.31 |
| Pr(Non-PWID\|CHC) (%) | 83.32 | 1.61 | 79.86 | 86.13 |

*Abbreviations*: $\rho_{rec}$, prevalence of recent PWID (proportion of the population that is recent PWID); $\rho_{ex}$, prevalence of ex-PWID (proportion of the population that is ex-PWID); $\pi_{rec}$, CHC prevalence among recent PWID; $\pi_{ex}$, CHC prevalence among ex-PWID; $\pi_{non}$, CHC prevalence among non-PWID; $\pi$ overall CHC prevalence in Italy; LB, Lower Boundary; UP, Upper Boundary

*Table 4. Results from the method assuming heterogeneity in the estimation of the CHC prevalence in the general population from different studies (i.e., similar to a random-effect meta-analysis). The number of individuals treated with direct-acting antivirals (DAAs) is taken into account.*

| Parameter | Posterior median | Posterior sd | LB (2.5%) | UB (97.5%) |
|---|---|---|---|---|
| $\rho_{rec}$ (%) | 0.1 | 0 | 0.09 | 0.1 |
| $\rho_{ex}$ (%) | 0.38 | 0 | 0.37 | 0.38 |
| $\pi_{rec}$ (%) | 32.08 | 2.06 | 28.12 | 36.14 |
| $\pi_{ex}$ (%) | 34.1 | 1.8 | 30.64 | 37.6 |
| $\pi_{non}$ (%) | 0.81 | 1.14 | 0 | 3.74 |
| $\pi$ (%) | 0.97 | 1.13 | 0.12 | 3.88 |
| Number with CHC | 460,284 | 539,863 | 57,618 | 1,846,423 |
| Pr(Recent PWID\|CHC) (%) | 3.15 | 214.99 | 0.67 | 18.99 |
| Pr(Ex-PWID\|CHC) (%) | 13.07 | 850.18 | 2.78 | 79.31 |
| Pr(Non-PWID\|CHC) (%) | 83.78 | 1065.08 | 0.76 | 96.57 |
| Between-study variance | 1.08 | 1.8 | 0.21 | 6.73 |

*Abbreviations*: $\rho_{rec}$, prevalence of recent PWID (proportion of the population that is recent PWID); $\rho_{ex}$, prevalence of ex-PWID (proportion of the population that is ex-PWID); $\pi_{rec}$, CHC prevalence among recent PWID; $\pi_{ex}$, CHC prevalence among ex-PWID; $\pi_{non}$, CHC prevalence among non-PWID; $\pi$ overall CHC prevalence in Italy; LB, Lower Boundary; UP, Upper Boundary

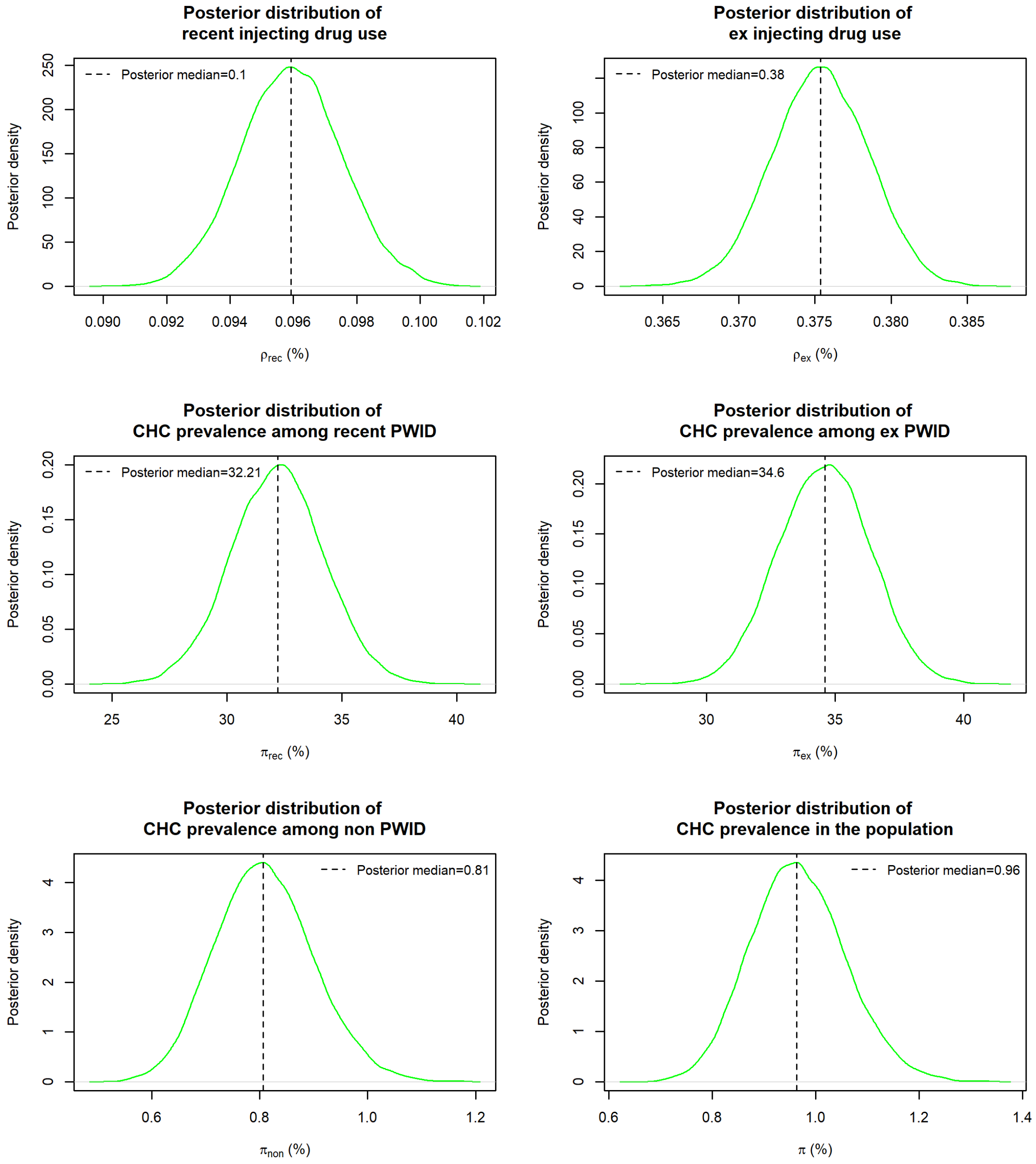


*Figure 1. Posterior distributions of the key model parameters using the approach that assumes no heterogeneity in the estimation of the CHC prevalence in the general population (primarily non-PWID) from different studies (i.e., similar to a fixed-effect meta-analysis), with the number of individuals treated with DAA taken into account.*

# APPENDIX

## Fit of the multi-state Markov model

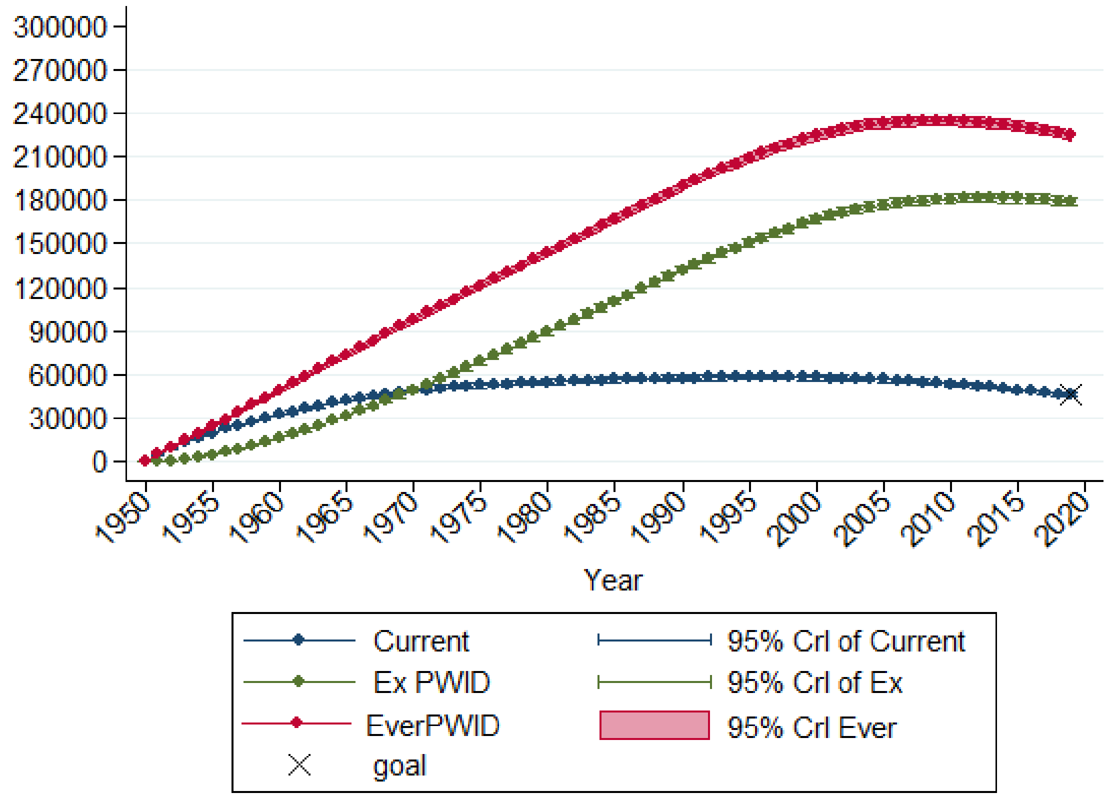


*Figure 2: Model predictions for the numbers of current and ex-PWID (people who inject drugs). The lines and the error bars show the median and 95% credible intervals (95% CrI) for the model projections. For comparison, asterisks indicate the observed country's data.*

## Stan code for Bayesian multiparameter evidence synthesis

```
data {
  int<lower=0> N1579; // Population of 15-79 in `Country`
  int<lower=1> Knon; // Number of studies in the ECDC data for `Country`
  int<lower=1> KnonCHC; // Number of studies in the ECDC data for `Country` (CHC data)
  int<lower=1> Kever; // Number of studies in the EMCDDA data for ever users in `Country`
  int<lower=1> Kcur; // Number of studies in the EMCDDA barometer (for HCV prevalence of PWID) in `Country`
  int<lower=1> NDAA; // Total number of DAAs from 2015 to 2019
  int<lower=1> sumHCVDeaths; // Number of HCV related deaths from 2015 to 2019 (non-PWID)

  real p_cur_mean; // Prior mean for the prevalence of current use in `Country`
  real<lower=0> p_cur_sd; // Prior sd for the prevalence of current use in `Country`

  real p_ex_mean; // Prior mean for the prevalence of ex-use in `Country`
  real<lower=0> p_ex_sd; // Prior sd for the prevalence of ex-use in `Country`

  //int<lower=0> Nst_hcv_cur[Kcur]; // Number of individuals in the study estimating anti-HCV among recent PWID
  //int<lower=0> Yst_hcv_cur[Kcur]; // Number of HCV+ in the study estimating anti-HCV among recent PWID
  real p_CHC_cur_mean; // Prior mean for the CHC prevalence among recent PWID in `Country`
  real<lower=0> p_CHC_cur_sd; // Prior sd for the CHC prevalence among recent PWID in `Country`
```

```
  int<lower=0> Nst_hcv_ever[Kever]; // Number of individuals in the study
estimating HCV among ever IDU
  int<lower=0> Yst_hcv_ever[Kever]; // Number of HCV+ in the study estima
ting HCV among ever PWID

  int<lower=0> Nst_hcv_non[Knon]; // Number of individuals in the study e
stimating anti-HCV among non PWID
  int<lower=0> Yst_hcv_non[Knon]; // Number of HCV+ in the study estimati
ng anti-HCV among non PWID
  int<lower=0> Nst_CHC_non[KnonCHC]; // Number of individuals in the stud
y estimating CHC among non PWID
  int<lower=0> Yst_CHC_non[KnonCHC]; // Number of HCV+ in the study estim
ating CHC among non PWID

  vector<lower=0>[3] alpha; // parameter of the Diriclet prior

  real HCVclear_mean; // Prior mean for the HCV clearance probability
  real<lower=0> HCVclear_sd; // Prior sd for the HCV clearance probabilit
y

  real SVR_mean; // Prior mean for the SVR among non-PWID
  real<lower=0> SVR_sd; // Prior sd for the SVR among non-PWID

  real SVR_PWID_mean; // Prior mean for the SVR among PWID
  real<lower=0> SVR_PWID_sd; // Prior sd for the SVR among PWID
}

// Block defining the original parameters
parameters {
  // The parameters to be sampled
  simplex[3] rho; // Prevalence of the three risk groups
  real<lower=0,upper=1> CHCpi_cur; // P(CHC+|Cur)
```

```
  real<lower=0,upper=1> CHCpi_ex; // P(CHC+|Ex)
  real<lower=0,upper=1> CHCpi_non; // P(CHC+|Non)
  real<lower=0,upper=1> SVR; // SVR of DAAs among non-PWID
  real<lower=0,upper=1> SVR_PWID; // SVR of DAAs among PWID
  real<lower=0,upper=1> rho_ever_DAA; // proportion of ever PWID among th
ose treated with DAA

  real<lower=0,upper=1-CHCpi_cur> HCVclear; // Probability of HCV clearan
ce; upper bound = 1-prevalence of chronic HCV
}


transformed parameters {
  // Change scales
  real<lower=0,upper=1> rho_ever;
  real<lower=0,upper=1> CHCpi_ever;
  real<lower=0,upper=1> pi_ever;
  real<lower=0,upper=1> pi_non;

  rho_ever = rho[1] + rho[2];
  CHCpi_ever = CHCpi_cur*rho[1]/rho_ever + CHCpi_ex*rho[2]/rho_ever;
  pi_ever = CHCpi_ever/(1-HCVclear);
  pi_non = CHCpi_non/(1-HCVclear);
}


// Binomial regression model
model {
  // Priors
  rho ~ dirichlet(alpha);

  ///////////////////////////////
```

```
  // Likelihood contributions //
  //////////////////////////////

  // Probability of HCV clearance
  HCVclear ~ normal(HCVclear_mean,HCVclear_sd);

  // Prevalence of current use
  rho[1] ~ normal(p_cur_mean,p_cur_sd);

  // Prevalence of ex-use
  rho[2] ~ normal(p_ex_mean,p_ex_sd);

  // Prevalence of chronic HCV among current users
  CHCpi_cur ~ normal(p_CHC_cur_mean,p_CHC_cur_sd);
  //Yst_hcv_cur ~ binomial(Nst_hcv_cur,pi_cur);

  // Prevalence of HCV among ever users
  Yst_hcv_ever ~ binomial(Nst_hcv_ever,pi_ever);

  // HCV+ among non
  Yst_hcv_non ~ binomial(Nst_hcv_non,pi_non);
  Yst_CHC_non ~ binomial(Nst_CHC_non,CHCpi_non);

  // SVR of DAAs among non-PWID
  SVR ~ normal(SVR_mean,SVR_sd);

  // SVR of DAAs among PWID
  SVR_PWID ~ normal(SVR_PWID_mean,SVR_PWID_sd);

  // Proportion of ever PWID among those treated with DAAs
  rho_ever_DAA ~ uniform(0.16, 0.21);
}
```

```
generated quantities {
  // Functions of parameters
  real<lower=0,upper=100> overalCHC;
  real logit_CHCpi_cur;
  real logit_CHCpi_ex;
  real logit_CHCpi_non;
  real logit_rho_cur;
  real logit_rho_ex;
  real logit_rho_non;
  real<lower=0,upper=1> pEverGivenCHC;
  real<lower=0,upper=1> pCurGivenCHC;
  real<lower=0,upper=1> pExGivenCHC;
  real<lower=0,upper=1> pNonGivenCHC;
  real logit_HCVclear;
  real<lower=0> NumberCHC;

  real<lower=0,upper=1> CHCDAApi_non; // P(CHC+|Non) adjusted for DAA
  real<lower=0,upper=1> CHCDthpi_non; // P(CHC+|Non) adjusted for HCV rel
ated mortality
  real<lower=0,upper=1> CHCDAApi_cur; // P(CHC+|Cur) adjusted for DAA
  real<lower=0,upper=1> CHCDAApi_ex; // P(CHC+|Ex) adjusted for DAA
  real<lower=0,upper=1> CHCDAApi_ever; // P(CHC+|Ever) adjusted for DAA

  real<lower=0,upper=100> overalCHC_DAA;
  real pEverGivenCHC_DAA;
  real pCurGivenCHC_DAA;
  real pExGivenCHC_DAA;
  real pNonGivenCHC_DAA;
  real<lower=0> NumberCHC_DAA;
```

```
  // Overall HCV prevalence
  CHCDthpi_non = ( N1579*rho[3]*CHCpi_non - sumHCVDeaths)/(N1579*rho[3]);
  overalCHC = 100*(rho[1]*CHCpi_cur + rho[2]*CHCpi_ex + rho[3]*CHCDthpi_n
on);
  pEverGivenCHC = CHCpi_ever*rho_ever/(overalCHC/100);
  pCurGivenCHC = CHCpi_cur*rho[1]/(overalCHC/100);
  pExGivenCHC = CHCpi_ex*rho[2]/(overalCHC/100);
  pNonGivenCHC = CHCDthpi_non*(1-rho_ever)/(overalCHC/100);
  NumberCHC = round(overalCHC*N1579/100);

  CHCDAApi_non = ( N1579*rho[3]*CHCDthpi_non - NDAA*(1-rho_ever_DAA)*SVR)
/(N1579*rho[3]);
  CHCDAApi_cur = ( N1579*rho[1]*CHCpi_cur - NDAA*rho_ever_DAA*SVR_PWID*pC
urGivenCHC/(pCurGivenCHC+pExGivenCHC))/(N1579*rho[1]);
  CHCDAApi_ex = ( N1579*rho[2]*CHCpi_ex - NDAA*rho_ever_DAA*SVR_PWID*pExG
ivenCHC/(pExGivenCHC+pCurGivenCHC))/(N1579*rho[2]);
  CHCDAApi_ever = CHCDAApi_cur*rho[1]/rho_ever + CHCDAApi_ex*rho[2]/rho_e
ver;

  overalCHC_DAA = 100*(rho[1]*CHCDAApi_cur + rho[2]*CHCDAApi_ex + rho[3]*
CHCDAApi_non);
  pEverGivenCHC_DAA = CHCDAApi_ever*rho_ever/(overalCHC_DAA/100);
  pCurGivenCHC_DAA = CHCDAApi_cur*rho[1]/(overalCHC_DAA/100);
  pExGivenCHC_DAA = CHCDAApi_ex*rho[2]/(overalCHC_DAA/100);
  pNonGivenCHC_DAA = CHCDAApi_non*(1-rho_ever)/(overalCHC_DAA/100);
  NumberCHC_DAA = round(overalCHC_DAA*N1579/100);

  logit_rho_cur = logit(rho[1]);
  logit_rho_ex = logit(rho[2]);
  logit_rho_non = logit(rho[3]);
  logit_CHCpi_cur = logit(CHCpi_cur);
  logit_CHCpi_ex = logit(CHCpi_ex);
```

```
  logit_CHCpi_non = logit(CHCpi_non);
  logit_HCVclear = logit(HCVclear);
}
```

### Multi-state Markov model

```
#include <stdio.h>
#include <stdlib.h>
#include <string.h>
#include <math.h>
#include <time.h>

#define loops 100
int populationPerYearAndAge[2021][66];
double deathRatePerAge[66];
int totalPopulationPerYear[2021];

FILE *F_DeathRate=fopen("greece-deathRates.txt","r");
FILE *F_Population=fopen("greece-population.txt","r");
FILE *out;

double pa_start = 0.00012;                    // Setting Variable
double pa_step  = 0.000002;                   // Setting Variable
double pa_stop  = 0.000123;                   // Setting Variable

double pa;
double pg = 1.0/11.7;    // Assumed average active injecting career of  11
.7 years
double pk = 0.004*12.0;     // Relapse rate of 0.004/month

struct people{
    int age;
    int state;
};

char filename[100];
struct people person[50000000];
```

```
long int totalPersons=0;

void setPopulationAge()
{
    for( int j=0; j<71; j++){
        for( int i=0; i<51; i++){
            fscanf(F_Population,"%d",&populationPerYearAndAge[1950+j][15+
i]);
        }
    }
}

void setDeathRate()
{
    for( int i=0; i<51; i++){
        fscanf(F_DeathRate,"%lf",&deathRatePerAge[15+i*5]);
        for( int j=0; j<5; j++){
            deathRatePerAge[15+i*5+j] = deathRatePerAge[15+i*5];
        }
    }
}

void getTotalPopulationPerAge()
{
    for( int j=0; j<71; j++){
        for( int i=0; i<51; i++){
            totalPopulationPerYear[1950+j] += populationPerYearAndAge[195
0+j][15+i];
        }
    }
}
```

```
void printTotalPopulationPerAge()
{
    printf("---- - ----------\n");
    printf("Year - Population\n");
    printf("---- - ----------\n");
    for( int i=1950; i<2021; i++){
        printf("%d - %d\n",i,totalPopulationPerYear[i]);
    }
}

void printDeathRatePerAge()
{
    printf("--- - -----\n");
    printf("Age - Rate\n");
    printf("--- - -----\n");
    for( int i=15; i<65; i++){
        printf("%d - %lf\n",i,deathRatePerAge[i]);
    }
}

void printTotalPersonPerState( int year)
{
    int count[4]= {0,0,0,0};
    for( int i=0; i<totalPersons; i++){
        count[person[i].state]++;
    }
    //printf("%d\t%ld\t%ld\t%ld\t%ld\n",year,count[0],count[1],count[2],c
ount[3]);
    if(year==2014)
        printf("%d\t%ld\t%ld\t%ld\t%ld\t%lf\n",year,count[0],count[1],cou
nt[2],count[3],pa);
    if( year>2009){
```

```
        fprintf(out, "%d\t%ld\t%ld\t%ld\t%ld\n",year,count[0],count[1],co
unt[2],count[3]);
    }
}

void initializePopulation()
{
    int cnt=0;
    for( int j=15; j<65; j++){
        for( int i=0; i<populationPerYearAndAge[1950][j]*100; i++){
            person[cnt].age=j;
            person[cnt].state=0;
            cnt++;
        }
    }
    totalPersons = cnt;
}

bool checkRate(double rate)
{
    if (rate <0.001){
        double k = rand()/(1.00+RAND_MAX);
        if(k<0.001){
            rate = rate*1000;
        }
        else{
            return false;
        }
    }
    double k = rand()/(1.00+RAND_MAX);
    if ( k < rate )
        return true;
```

```
    else
        return false;
}

void changeStatusAndAge()
{
    for( int i=0; i<totalPersons; i++){
        // change state
        double k = rand()/(1.00+RAND_MAX);
        if( person[i].state==0){
            if ( checkRate(pa) &&  person[i].age<40){
                person[i].state=1;
            }
        }
        else if( person[i].state==1){
            if ( k < pg ){
                person[i].state=2;
            }
        }
        else if( person[i].state==2){
            if ( k < pk ){
                person[i].state=1;
            }
        }
        // After their 49th year, all active PWID are assumed to cease in
jecting
        if(person[i].age>49 && person[i].state==1){
            person[i].state=2;
        }
        // After their 64th year, remove
        if(person[i].age>64){
            person[i].state=3;
```

```
        }
        // death rate depending on age and state
        k = rand()/(1.00+RAND_MAX);
        if( person[i].state==0){
            if ( k < deathRatePerAge[person[i].age] ){
                person[i].state=3;
            }
        }
        else if( person[i].state==1){
            if ( k < deathRatePerAge[person[i].age] * 13.0 ){
                person[i].state=3;
            }
        }
        else if( person[i].state==2){
            if ( k < deathRatePerAge[person[i].age] ){
                person[i].state=3;
            }
        }
        // increase age one year
        person[i].age++;
    }
}

void addNewPersons(int year)
{
    // add new person [15 years old and non PWID]
    int cnt=totalPersons;
    for( int i=0; i<populationPerYearAndAge[year+1][15]*100; i++){
// 100 instead of 1000 for the population to reduce execution time
        person[cnt].age=15;
        person[cnt].state=0;
        cnt++;
```

```
    }
    // increase the total number of population
    totalPersons = cnt;
}
int main()
{
    srand(time(NULL));

    setPopulationAge();
    setDeathRate();

    getTotalPopulationPerAge();
    pa = pa_start;
    while ( pa < pa_stop){

        snprintf(filename, 100, "result_%lf.txt",pa);
        out=fopen(filename,"w");

        for( int iter=0; iter<loops ;iter++){
            initializePopulation();
            for( int year=1950; year<2020; year++){
                printTotalPersonPerState(year);
                changeStatusAndAge();
                addNewPersons(year);
            }
        }

        fclose(out);
        pa = pa + pa_step;
    }
```

```
}
```

## REFERENCES


Andriulli, Angelo, Tommaso Stroffolini, Andrea Mariano, Maria Rosa Valvano, Ignazio Grattagliano, Antonio Massimo Ippolito, Adriano Grossi, et al. 2018. “Declining Prevalence and Increasing Awareness of HCV Infection in Italy: A Population-Based Survey in Five Metropolitan Areas.” *European Journal of Internal Medicine* 53 (July): 79–84. https://doi.org/10.1016/j.ejim.2018.02.015.

European Centre for Disease Prevention and Control (ECDC). 2016. “Systematic Review on Hepatitis b and c Prevalence in the EU/EEA.” https://www.ecdc.europa.eu/sites/default/files/media/en/publications/Publications/systematic-review-hepatitis-B-C-prevalence.pdf.

———. 2021. “Hepatitis c Prevalence Database.” https://www.ecdc.europa.eu/en/all-topics-z/hepatitis-c/tools/hepatitis-c-prevalence-database.

Graf, Christiana, Marcus M Mücke, Georg Dultz, Kai-Henrik Peiffer, Alica Kubesch, Patrick Ingiliz, Stefan Zeuzem, Eva Herrmann, and Johannes Vermehren. 2019. “Efficacy of Direct-acting Antivirals for Chronic Hepatitis C Virus Infection in People Who Inject Drugs or Receive Opioid Substitution Therapy: A Systematic Review and Meta-analysis.” *Clinical Infectious Diseases* 70 (11): 2355–65. https://doi.org/10.1093/cid/ciz696.

Grebely, Jason, Sarah Larney, Amy Peacock, Samantha Colledge, Janni Leung, Matthew Hickman, Peter Vickerman, et al. 2019. “Global, Regional, and Country-Level Estimates of Hepatitis c Infection Among People Who Have Recently Injected Drugs.” *Addiction* 114 (1): 150–66. https://doi.org/https://doi.org/10.1111/add.14393.

Hines, Lindsey A., Adam Trickey, Janni Leung, Sarah Larney, Amy Peacock, Louisa Degenhardt, Samantha Colledge, et al. 2020. “Associations Between National Development Indicators and the Age Profile of People Who Inject Drugs: Results from a Global Systematic Review and Meta-Analysis.” *The Lancet Global Health* 8 (1): e76–91. https://doi.org/10.1016/S2214-109X(19)30462-0.

Kondili, Loreta A., Massimo Andreoni, Alfredo Alberti, Salvatore Lobello, Sergio Babudieri, Antonio Saverio Roscini, Rocco Merolla, Walter Marrocco, and Antonio Crax. 2021. “Estimated

Prevalence of Undiagnosed HCV Infected Individuals in Italy: A Mathematical Model by Route of Transmission and Fibrosis Progression." *Epidemics* 34: 100442. https://doi.org/https://doi.org/10.1016/j.epidem.2021.100442.

Lampertico, Pietro, Jose A. Carrión, Michael Curry, Juan Turnes, Markus Cornberg, Francesco Negro, Ashley Brown, et al. 2020. "Real-World Effectiveness and Safety of Glecaprevir/Pibrentasvir for the Treatment of Patients with Chronic HCV Infection: A meta-Analysis." *Journal of Hepatology* 72 (6): 1112–21. https://doi.org/10.1016/j.jhep.2020.01.025.

Lin, Lifeng, and Haitao Chu. 2020. "Meta-Analysis of Proportions Using Generalized Linear Mixed Models." *Epidemiology* 31 (5). https://journals.lww.com/epidem/Fulltext/2020/09000/Meta_analysis_of_Proportions_Using_Generalized.16.aspx.

McDonald, Scott A., Rosmawati Mohamed, Maznah Dahlui, Herlianna Naning, and Adeeba Kamarulzaman. 2014. "Bridging the Data Gaps in the Epidemiology of Hepatitis c Virus Infection in Malaysia Using Multi-Parameter Evidence Synthesis." *BMC Infectious Diseases* 14 (1): 564. https://doi.org/10.1186/s12879-014-0564-6.

Micallef, J. M., J. M. Kaldor, and G. J. Dore. 2006. "Spontaneous Viral Clearance Following Acute Hepatitis c Infection: A Systematic Review of Longitudinal Studies." *Journal of Viral Hepatitis* 13 (1): 34–41. https://doi.org/https://doi.org/10.1111/j.1365-2893.2005.00651.x.

Morisco, Filomena, Ilaria Loperto, Tommaso Stroffolini, Flavia Lucia Lombardo, Valentina Cossiga, Maria Guarino, Alessia De Feo, and Nicola Caporaso. 2017. "Prevalence and Risk Factors of HCV Infection in a Metropolitan Area in Southern Italy: Tail of a Cohort Infected in Past Decades." *Journal of Medical Virology* 89 (2): 291–97. https://doi.org/https://doi.org/10.1002/jmv.24635.

Parisi, Maria, Laura Soldini, Gianmarino Vidoni, Chiara Mabellini, Teresa Belloni, Livia Brignolo, Silvia Negri, Karin Schlusnus, Fernanda Dorigatti, and Adriano Lazzarin. 2014. "Point-of-Care Testing for HCV Infection: Recent Advances and Implications for Alternative Screening." *The New Microbiologica* 37 (October): 449–547.

Presanis, Anne M., O. Noel Gill, Timothy R. Chadborn, Caterina Hill, Vivian Hope, Louise Logan, Brian D. Rice, Valerie C. Delpech, A. E. Ades, and Daniela De Angelis. 2010. "Insights into the Rise in HIV Infections, 2001 to 2008: A Bayesian Synthesis of Prevalence Evidence." *AIDS* 24 (18). https://journals.lww.com/aidsonline/Fulltext/2010/11270/Insights_into_the_rise_in_HIV_infections,_2001_to.12.aspx.

Spada, Enea, Cinzia Marcantonio, Maria Fenicia Vescio, Nadia Marascio, Umbertina Villano, Giulio Pisani, Elena Tritarelli, et al. 2021. "Changing Epidemiology of Hepatitis c in Italy: A Population-Based Survey in a Historically High Endemic Area." *Minerva Medica*. https://doi.org/10.23736/s0026-4806.21.07280-3.

Sweeting, M. J., D. De Angelis, M. Hickman, and A. E. Ades. 2008. "Estimating hepatitis C prevalence in England and Wales by synthesizing evidence from multiple data sources. Assessing data conflict and model fit." *Biostatistics* 9 (4): 715–34. https://doi.org/10.1093/biostatistics/kxn004.

Tan, Sarah, Susanna Makela, Daliah Heller, Kevin Konty, Sharon Balter, Tian Zheng, and James H. Stark. 2018. "A Bayesian Evidence Synthesis Approach to Estimate Disease Prevalence in Hard-to-Reach Populations: Hepatitis c in New York City." *Epidemics* 23: 96–109. https://doi.org/https://doi.org/10.1016/j.epidem.2018.01.002.

Thein, Hla-Hla, Qilong Yi, Gregory J. Dore, and Murray D. Krahn. 2008. "Estimation of Stage-Specific Fibrosis Progression Rates in Chronic Hepatitis c Virus Infection: A Meta-Analysis and Meta-Regression." *Hepatology* 48 (2): 418–31. https://doi.org/https://doi.org/10.1002/hep.22375.

Veen, Maaike G. van, Anne M. Presanis, Stefano Conti, Maria Xiridou, Annemarie R. Stengaard, Martin C. Donoghoe, Ard I. van Sighem, Marianne A. van der Sande, and Daniela De Angelis. 2011. "National Estimate of HIV Prevalence in the Netherlands: Comparison and Applicability of Different Estimation Tools." *AIDS* 25 (2). https://journals.lww.com/aidsonline/Fulltext/2011/01140/National_estimate_of_HIV_prevalence_in_the.14.aspx.

# Estimating the prevalence of chronic hepatitis C infection (CHC) in Latvia using Bayesian multiparameter evidence synthesis

23/05/2022

## Table of Contents


Introduction ........ 2

Methods ........ 2

Prevalence of ex-PWID ........ 3

Prevalence of recent PWID ........ 3

Prevalence of CHC among recent PWID ........ 4

Prevalence of CHC among ex-PWID ........ 4

Prevalence of CHC among non-PWID ........ 5

Results ........ 6

Tables and Figures ........ 8

Appendix ........ 14

Fit of the multi-state Markov model ........ 14

Stan code for Bayesian multiparameter evidence synthesis ........ 15

Multi-state Markov model ........ 21

References ........ 29

# INTRODUCTION

Patients with chronic hepatitis C (HCV) infection often remain asymptomatic for decades and even for life, but a proportion of them develop active chronic hepatitis, which is a progressive disease (Thein et al. 2008). Because of the largely asymptomatic nature of HCV infection, notification data reflect national screening and testing practices and do not give accurate insights into the prevalence of HCV infection. Although there are studies that have estimated the prevalence of HCV antibodies (anti-HCV) in specific groups such as the general population, first-time blood donors, or people who inject drugs (PWID) at drug treatment centers, those alone cannot be combined to produce national estimates unless some additional information regarding the composition of each HCV risk group and its prevalence in the population are known (Sweeting et al. 2008; McDonald et al. 2014; Tan et al. 2018).

# METHODS

Bayesian multiparameter evidence synthesis (MPES) has been a popular approach to formally estimate anti-HCV or HIV prevalence (Sweeting et al. 2008; McDonald et al. 2014; Tan et al. 2018; Presanis et al. 2010; Veen et al. 2011). In this project, we extend this method to estimating the CHC prevalence, with the population (15-79 years) split into three main non-overlapping risk groups, i.e., recent PWID (those who have injected in the last year), ex-PWID, and non-PWID. A unified model is assumed including parameters associated with the CHC prevalence of recent, ex-PWID, and non-PWID (denoted by $\pi_{rec}$, $\pi_{ex}$, and $\pi_{non}$, respectively) and the prevalence of these risk groups in the population (denoted by $\rho_{rec}$, $\rho_{ex}$, and $\rho_{non}$, respectively; $\rho_{rec} + \rho_{ex} + \rho_{non} = 1$). To estimate the CHC prevalence in the whole population, $\pi$, we used the formula:

$$\pi = \pi_{rec}\rho_{rec} + \pi_{ex}\rho_{ex} + \pi_{non}\rho_{non} \quad (1)$$

Bayesian synthesis considers all available data, allowing for an evidence-based estimation of CHC prevalence in the population, with inherent uncertainty properly accounted for. Our aim is to estimate the CHC prevalence for the total (15-79) population of Latvia in 2019 using sources of information over the last decade (2010-2019).

## Prevalence of ex-PWID

Data on the prevalence of ex-PWID are sparse and generally often unreliable. To overcome this limitation, we apply the method proposed by McDonald et al. (2014), which requires simulating a multi-state Markov model representing the non-PWID, recent PWID, and ex-PWID. In the multi-state Markov model, the simulation starts in 1950 and examines the population aged 15-79 years old. A certain number of 15-year-old individuals enter the simulation process each year. These numbers of 15-year-old individuals are obtained from the United Nations Statistic Division UNSD, being different for each country. The ageing of the population is also taken into account. Country-specific overall mortality data are retrieved from the Life Tables of the World Health Organization WHO. The duration of injecting career is also country-specific and obtained from the paper of Hines et al. (2020).

To estimate the number of ex-PWID, the model can be calibrated on the number of recent PWID provided in the EMCDDA barometer or the EMCDDA statistical bulletin (the code of the Markov model can be found in the Appendix). If there are no available data in the barometer, estimates from the systematic review of Grebely et al. (2019) could be used. Alternatively, if the national focal points suggest or provide different and updated or more accurate data for calibration purposes, we will consider their advice and adjust the model accordingly.

After applying the model for Latvia, we compute the number (and the corresponding Confidence Interval - CI) of ex-PWID in 2019. The number of ex-PWID is then divided by the population size (15-79 years) in the same year, and a Normal distribution, constrained such that $\rho_{ex} \in (0,1)$, is assumed for $\rho_{ex}$. The standard deviation of this distribution is specified to approximately correspond to the respective CI, i.e. $(UB_{\rho_{ex}} - LB_{\rho_{ex}})/(2 \times 1.96)$, where $UB_{\rho_{ex}}$ and $LB_{\rho_{ex}}$ denote the upper and lower limit of the CI.

## Prevalence of recent PWID

The number of recent PWID in Latvia in 2019 is also projected by the multi-state Markov model described in the previous subsection. To estimate $\rho_{rec}$, the number of recent PWID is divided by the population size (15-79 years). Similar to the previous subsection, a Normal distribution, constrained such that $\rho_{rec} \in (0,1)$, is assumed for $\rho_{rec}$, with the standard deviation specified to correspond to the CI obtained from the multi-state Markov model.

## Prevalence of CHC among recent PWID

The prevalence (and the 95% CI) of CHC among recent PWID can be informed from studies in the EMCDDA statistical bulletin. If information on the CHC prevalence is available, the Binomial distribution in the model to inform $\pi_{rec}$ can be used. However, as these EMCDDA data typically refer to the anti-HCV prevalence and not to the CHC prevalence, they could not be used directly. This issue can be addressed by noting that spontaneous viral clearance occurs in approximately one in four people with acute hepatitis C (Micallef, Kaldor, and Dore 2006). To account for the variability of the HCV clearance probabilities, we can use the result reported in Micallef, Kaldor, and Dore (2006), i.e., the proportion of HCV clearance (denoted by $\rho_{clear}$) is equal to 0.26 (95% CI 0.22–0.29). Thus, in the absence of effective treatment, estimates of the CHC prevalence among recent PWID ($\pi_{rec}$) could be obtained using the formula $\pi_{rec} = \pi(\text{anti-HCV})_{rec}(1 - \rho_{clear})$, where $\pi(\text{anti-HCV})_{rec}$ denotes the anti-HCV prevalence among recent PWID. However, apart from spontaneous clearance, some people may have been treated with direct acting antivirals (DAAs), with the sustained virologic response ($SVR_{PWID}$) among PWID estimated to be 88% (95% CI: 80% to 93%) (Graf et al. 2019). To adjust for that, if the total number of recent PWID treated with DAAs up to 2019, $N_{DAA|rec}$, is available in Latvia, the CHC prevalence in non-PWID can be estimated by

$$\pi_{rec} = \frac{N_{15,79}\rho_{rec}\pi(\text{anti-HCV})_{rec}(1 - \rho_{clear}) - N_{DAA|rec}SVR_{PWID}}{N_{15,79}\rho_{rec}}. \quad (2)$$

The number of recent PWID treated with DAAs up to 2019 was provided by the focal point. To estimate the anti-HCV prevalence, data from a multicenter study in 2017, available in the EMCDDA statistical bulletin, were used.

## Prevalence of CHC among ex-PWID

Information on CHC prevalence among ex-PWID (i.e., $\pi_{ex}$) is difficult to obtain directly. To overcome this, national anti-HCV prevalence data among ever PWID in 2019, available in the EMCDDA statistical bulletin, were used. However, as these data refer to the anti-HCV prevalence, they are adjusted according to the procedure described in the previous subsection, i.e.

$$\pi_{ever} = \frac{N_{15,79}\rho_{ever}\pi(\text{anti-HCV})_{ever}(1-\rho_{clear}) - N_{DAA|ever}SVR_{PWID}}{N_{15,79}\rho_{ever}}. \quad (3)$$

where $\pi(\text{anti-HCV})_{ever}$ denotes the anti-HCV prevalence among ever PWID, $\rho_{ever} = \rho_{rec} + \rho_{ex}$, and $N_{DAA|ever}$ denotes the total number of ever PWID treated with DAAs up to 2019 in Latvia, which is provided by the focal point. Then, since an estimate of $\pi_{ever}$ is available, $\pi_{ex}$ can be indirectly estimated since $\pi_{ever}$ is equal to a weighted average of $\pi_{rec}$ and $\pi_{ex}$, as shown by the following formula

$$\pi_{ever} = \frac{\rho_{rec}}{\rho_{ever}}\pi_{rec} + \frac{\rho_{ex}}{\rho_{ever}}\pi_{ex}, \quad (4)$$

or, equivalently,

$$\pi_{ex} = \left(\pi_{ever} - \frac{\rho_{rec}}{\rho_{ever}}\pi_{rec}\right) \times \frac{\rho_{ever}}{\rho_{ex}}. \quad (5)$$

## Prevalence of CHC among non-PWID

To estimate the CHC prevalence among non-PWID in a country (after 2010), the ECDC database (European Centre for Disease Prevention and Control (ECDC) 2021) was used. The ECDC group has carefully and critically collected information from relevant studies across the EU/EEA region in cooperation with the focal points of each country. On the basis of the representativeness of each study and other factors (European Centre for Disease Prevention and Control (ECDC) 2016), a risk of bias score was assigned to each study, with higher values indicating higher-quality studies. When higher-quality studies are available in a country ($\geq 4$), they are used to estimate the overall CHC prevalence in that country among non-PWID. If there are no higher-quality studies, general population estimates with a lower quality (<4) are pooled. If no general population prevalence estimates are available, data on the prevalence of HCV in pregnant women could be used as a prevalence measure. Finally, if data on pregnant women are lacking too, first-time blood donor studies could be an option. However, if the national focal point agrees or recommends, estimates from a neighboring country could be used instead, or we could use any other relevant information provided/suggested by the national focal point.

We directly use CHC prevalence data from studies in the general population, if available. If the data in the general population refer to anti-HCV prevalence (without any data on the viremic population), we adjust the estimates to get CHC prevalence based on the spontaneous HCV clearance estimate of 26% and the number of individuals treated with DAAs in the general population, as previously described, i.e.

$$\pi_{non} = \frac{N_{15,79}\rho_{non}\pi(\text{anti-HCV})_{non}(1-\rho_{clear}) - N_{DAA|non}SVR}{N_{15,79}\rho_{non}}. \quad (6)$$

where $\pi(\text{anti-HCV})_{non}$ denotes the anti-HCV prevalence among non-PWID, $SVR$ is the sustained virologic response of DAAs in the general population estimated to be 96.7% (95% CI: 95.4% to 98.1%)(Lampertico et al. 2020), and $N_{DAA|non}$ denotes the total number of non-PWID treated with DAAs up to 2019 in Latvia, provided by the focal point.

As $\rho_{rec}$ and $\rho_{ex}$ are likely to be low, $\pi_{non}$ will be the most critical factor for the estimation of the overall CHC prevalence in the population. To adjust for potential heterogeneity between the CHC prevalence estimates from different studies in the non-PWID population in a country, as a sensitivity analysis, we also apply a random-effect meta-analytic approach to pool the CHC prevalence across studies on non-PWID (Lin and Chu 2020). The random-effect approach is carried out only when at least 3 studies are available in the general population of a certain country, and the estimates are compared with the corresponding estimates from the approach assuming no heterogeneity (similar to a fixed-effect meta-analysis). If the estimates are inconsistent, possible explanations are investigated and discussed. If there is substantial heterogeneity between studies, it would help more to explore the causes; for example, following national focal point suggestions, some studies could be excluded to reduce heterogeneity.

# RESULTS

The above-mentioned approach was used to estimate the prevalence of CHC in 2019 (considering ages between 15-79 years: 1,507,375).

The aggregated data used by our approach are briefly presented in Table 1. The multi-state Markov model to estimate the prevalence of recent and ex-PWID was calibrated on the estimates reported in the EMCDDA barometer. In Latvia, there were 7 studies on first-time blood donors, which included only anti-HCV data. To estimate the CHC prevalence in the general

population (primarily non-PWID), we used a fixed-effect meta-analytic approach, with the corresponding results, when information on DAAs is ignored, presented in Table 2. However, based on data provided by the focal point, approximately 6,500 individuals were treated with DAAs up to 2019 in Latvia, of whom 13 were recent PWID, 422 were ex-PWID, and 6,065 were non-PWID (i.e. 0.2% and 6.5% of treated were recent and former PWID). The corresponding results accounting for the DAA uptake are presented in Table 3.

The prevalence of recent and ex-PWID was low in Latvia (about 0.49% and 1.39%, respectively) corresponding to 7,400 (95% CI: 6,850-8,000) recent PWID and 20,930 (95% CI: 20,140-21,750) ex-PWID in the population. However, the CHC prevalence in these groups was substantial (Table 2) being 64.59% and 22.33%, respectively. This translates to 4,779 (95% CI: 4,307-5,272) and 4,671 (95% CI: 3,782-5,597) recent and ex-PWID aged 15-79 living with CHC infection in Latvia in 2019. The CHC prevalence in the general population was 0.15% (95% CI: 0.09%-0.21%), much lower than that of the high-risk groups. Taking all pieces of information into account, the overall CHC prevalence in Latvia in 2019 was equal to 0.77% (95% CI: 0.68%-0.87%), which corresponds to 11,640 (95% CI: 10,236-13,090) individuals aged 15-79 years with CHC infection. The corresponding results under a random-effect meta-analysis for the studies in the general population were similar, but with higher uncertainty, and are provided in Table 3.

# TABLES AND FIGURES

*Table 1. Data (real) contributing to the estimation of CHC prevalence in Latvia in 2019.*

| Parameter | Number (95%CI) | Numerator | Denominator | Notes | Year of study |
|---|---|---|---|---|---|
| $\rho_{rec}$ | 7,400 (6,850-8,000) | | | Method based on McDonald et al. | 2019 |
| $\rho_{ex}$ | 20,930 (20,140-21,750) | | | Method based on McDonald et al. | 2019 |
| $\pi(\text{anti-HCV})_{rec}$ | | 240 | 274 | EMCDDA database | 2017 |
| $\pi(\text{anti-HCV})_{ever}$ | | 312 | 666 | EMCDDA database | 2019 |
| $\pi(\text{anti-HCV})_{non}$ | | 116 | 9,148 | ECDC database (NA et al.); Risk of bias=NA | 2013 |
| $\pi(\text{anti-HCV})_{non}$ | | 130 | 9,838 | ECDC database (NA et al.); Risk of bias=NA | 2014 |
| $\pi(\text{anti-HCV})_{non}$ | | 57 | 8,658 | ECDC database (NA et al.); Risk of bias=NA | 2015 |
| $\pi(\text{anti-HCV})_{non}$ | | 31 | 8,405 | Provided by the focal point | 2016 |
| $\pi(\text{anti-HCV})_{non}$ | | 22 | 6,158 | Provided by the focal point | 2017 |
| $\pi(\text{anti-HCV})_{non}$ | | 16 | 6,675 | Provided by the focal point | 2018 |
| $\pi(\text{anti-HCV})_{non}$ | | 32 | 6,280 | Provided by the focal point | 2019 |

*Notes*: Higher risk of bias score denotes a higher-quality study (range from 0 to 6);

*Table 2. Results from the method assuming no heterogeneity in the estimation of the CHC prevalence in the general population from different studies (i.e., similar to a fixed-effect meta-analysis), ignoring information on DAAs.*

| Parameter | Posterior median | Posterior sd | LB (2.5%) | UB (97.5%) |
|---|---|---|---|---|
| $\rho_{rec}$ (%) | 0.49 | 0.02 | 0.45 | 0.53 |
| $\rho_{ex}$ (%) | 1.39 | 0.03 | 1.33 | 1.44 |
| $\pi_{rec}$ (%) | 64.72 | 2.12 | 60.55 | 68.86 |
| $\pi_{ex}$ (%) | 24.12 | 2.14 | 20 | 28.43 |
| $\pi_{non}$ (%) | 0.54 | 0.03 | 0.49 | 0.6 |
| $\pi$ (%) | 1.19 | 0.05 | 1.09 | 1.28 |
| Number with CHC | 17,875 | 733 | 16,479 | 19,365 |
| Pr(Ever PWID\|CHC) (%) | 55.01 | 1.66 | 51.77 | 58.26 |
| Pr(Non-PWID\|CHC) (%) | 44.99 | 1.66 | 41.74 | 48.23 |

*Notes*: $\rho_{rec}$, prevalence of recent PWID; $\rho_{ex}$, prevalence of ex-PWID; $\pi_{rec}$, CHC prevalence among recent PWID; $\pi_{ex}$, CHC prevalence among ex-PWID; $\pi_{non}$, CHC prevalence among non-PWID; $\pi$ overall CHC prevalence in Latvia; LB, Lower Boundary; UP, Upper Boundary

*Table 3. Results from the method assuming no heterogeneity in the estimation of the CHC prevalence in the general population from different studies (i.e., similar to a fixed-effect meta-analysis), with the number of individuals treated with DAA taken into account.*

| Parameter | Posterior median | Posterior sd | LB (2.5%) | UB (97.5%) |
|---|---|---|---|---|
| $\rho_{rec}$ (%) | 0.49 | 0.02 | 0.45 | 0.53 |
| $\rho_{ex}$ (%) | 1.39 | 0.03 | 1.33 | 1.44 |
| $\pi_{rec}$ (%) | 64.59 | 2.12 | 60.36 | 68.68 |
| $\pi_{ex}$ (%) | 22.33 | 2.11 | 18.21 | 26.56 |
| $\pi_{non}$ (%) | 0.15 | 0.03 | 0.09 | 0.21 |
| $\pi$ (%) | 0.77 | 0.05 | 0.68 | 0.87 |
| Number with CHC | 11,640 | 725 | 10,236 | 13,090 |
| Pr(Ever PWID\|CHC) (%) | 81.26 | 3.07 | 75.43 | 87.61 |
| Pr(Non-PWID\|CHC) (%) | 18.74 | 3.07 | 12.39 | 24.57 |

*Notes*: $\rho_{rec}$, prevalence of recent PWID; $\rho_{ex}$, prevalence of ex-PWID; $\pi_{rec}$, CHC prevalence among recent PWID; $\pi_{ex}$, CHC prevalence among ex-PWID; $\pi_{non}$, CHC prevalence among non-PWID; $\pi$ overall CHC prevalence in Latvia; LB, Lower Boundary; UP, Upper Boundary

*Table 4. Results from the method assuming heterogeneity in the estimation of the CHC prevalence in the general population from different studies (i.e., similar to a random-effect meta-analysis), with information on DAAs taken into account.*

| Parameter | Posterior median | Posterior sd | LB (2.5%) | UB (97.5%) |
|---|---|---|---|---|
| $\rho_{rec}$ (%) | 0.49 | 0.02 | 0.45 | 0.53 |
| $\rho_{ex}$ (%) | 1.39 | 0.03 | 1.34 | 1.44 |
| $\pi_{rec}$ (%) | 64.55 | 2.15 | 60.3 | 68.7 |
| $\pi_{ex}$ (%) | 22.33 | 2.15 | 18.13 | 26.6 |
| $\pi_{non}$ (%) | 0.02 | 0.15 | 0 | 0.47 |
| $\pi$ (%) | 0.65 | 0.18 | 0.42 | 1.1 |
| Number with CHC | 9,796 | 2,720 | 6,333 | 16,592 |
| Pr(Ever PWID\|CHC) (%) | 96.78 | 12.61 | 57.84 | 100 |
| Pr(Non-PWID\|CHC) (%) | 3.22 | 12.61 | 0 | 42.16 |
| Between-study variance | 0.68 | 0.8 | 0.2 | 2.96 |

*Notes*: $\rho_{rec}$, prevalence of recent PWID; $\rho_{ex}$, prevalence of ex-PWID; $\pi_{rec}$, CHC prevalence among recent PWID; $\pi_{ex}$, CHC prevalence among ex-PWID; $\pi_{non}$, CHC prevalence among non-PWID; $\pi$ overall CHC prevalence in Latvia; LB, Lower Boundary; UP, Upper Boundary

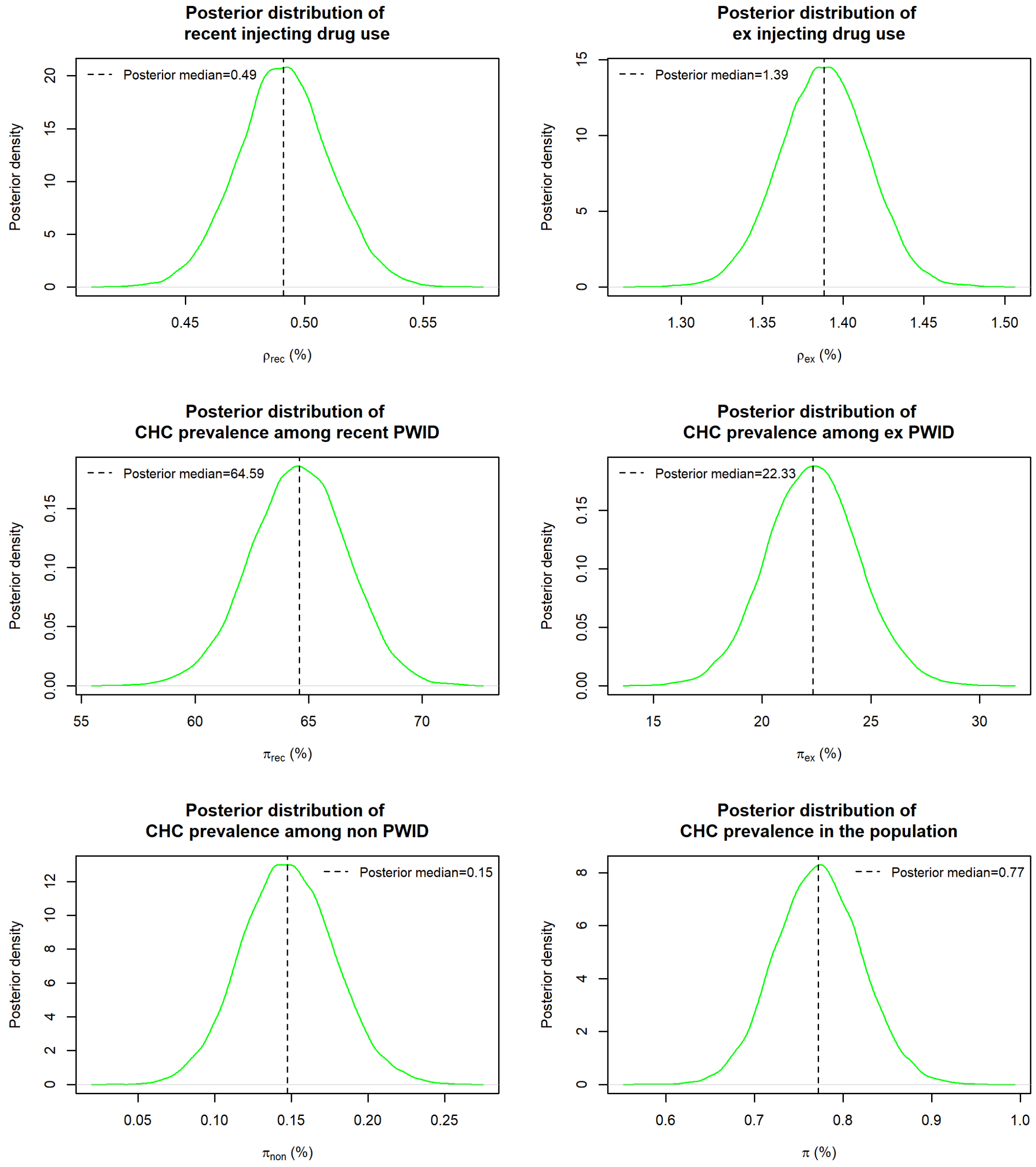


*Figure 1. Posterior distributions of the key model parameters using the approach that assumes no heterogeneity in the estimation of the CHC prevalence in the general population (primarily non-PWID) from different studies (i.e., similar to a fixed-effect meta-analysis), with information on DAA uptake accounted for.*

# APPENDIX

## Fit of the multi-state Markov model

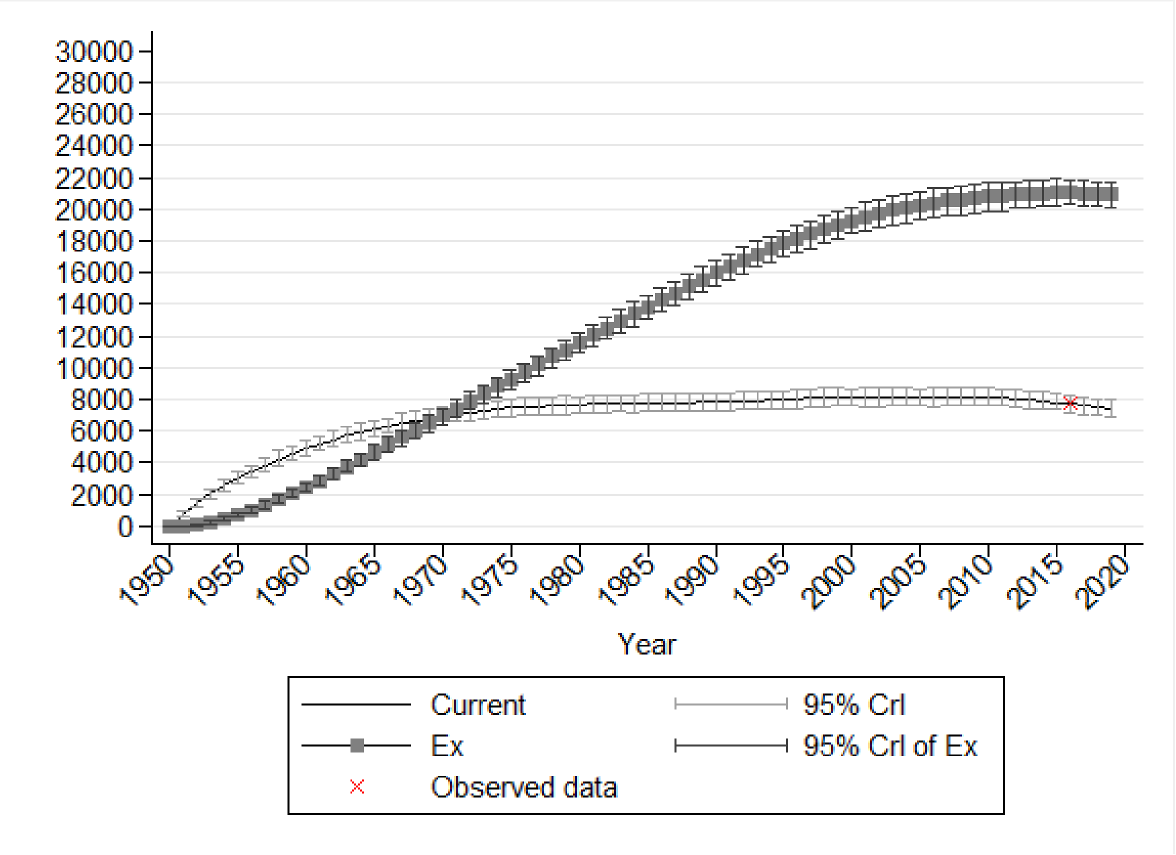


*Figure 2: Model predictions for the numbers of current and ex-PWID (people who inject drugs). The solid black line and shaded grey error bars show the median and 95% credible intervals (95% CrI) for the model projections. For comparison, asterisks indicate the observed country's data.*

## Stan code for Bayesian multiparameter evidence synthesis

```
data {
  int<lower=0> N1579; // Population of 15-79 in `Country`
  int<lower=1> Knon; // Number of studies in the ECDC data for `Country`
  int<lower=1> Kever; // Number of studies in the EMCDDA data for ever us
ers in `Country`
  int<lower=1> Kcur; // Number of studies in the EMCDDA barometer (for HC
V prevalence of PWID) in `Country`
  int<lower=1> Ntrt_non; // Number of individuals having received DAA fro
m 2015 to 2019 in non-PWID
  int<lower=1> Ntrt_ex; // Number of individuals having received DAA from
2015 to 2019 in ex-PWID
  int<lower=1> Ntrt_cur; // Number of individuals having received DAA fro
m 2015 to 2019 in recent PWID

  real p_cur_mean; // Prior mean for the prevalence of current use in `Co
untry`
  real<lower=0> p_cur_sd; // Prior sd for the prevalence of current use i
n `Country`

  real p_ex_mean; // Prior mean for the prevalence of ex-use in `Country`
  real<lower=0> p_ex_sd; // Prior sd for the prevalence of ex-use in `Cou
ntry`

  int<lower=0> Nst_hcv_cur[Kcur]; // Number of individuals in the study e
stimating anti-HCV among recent PWID
  int<lower=0> Yst_hcv_cur[Kcur]; // Number of HCV+ in the study estimati
ng anti-HCV among recent PWID
  //real p_CHC_cur_mean; // Prior mean for the CHC prevalence among recen
t PWID in `Country`
  //real<lower=0> p_CHC_cur_sd; // Prior sd for the CHC prevalence among
recent PWID in `Country`
```

```
  int<lower=0> Nst_hcv_ever[Kever]; // Number of individuals in the study
estimating HCV among ever IDU
  int<lower=0> Yst_hcv_ever[Kever]; // Number of HCV+ in the study estima
ting HCV among ever PWID

  int<lower=0> Nst_hcv_non[Knon]; // Number of individuals in the study e
stimating anti-HCV among non PWID
  int<lower=0> Yst_hcv_non[Knon]; // Number of HCV+ in the study estimati
ng anti-HCV among non PWID

  vector<lower=0>[3] alpha; // parameter of the Diriclet prior

  real HCVclear_mean; // Prior mean for the HCV clearance probability
  real<lower=0> HCVclear_sd; // Prior sd for the HCV clearance probabilit
y

  real SVR_mean; // Prior mean for the SVR among non-PWID
  real<lower=0> SVR_sd; // Prior sd for the SVR among non-PWID

  real SVR_PWID_mean; // Prior mean for the SVR among PWID
  real<lower=0> SVR_PWID_sd; // Prior sd for the SVR among PWID

}

// Block defining the original parameters
parameters {
  // The parameters to be sampled
  simplex[3] rho; // Prevalence of the three risk groups
  real<lower=0,upper=1> CHCpi_cur; // P(CHC+|Cur)
  real<lower=0,upper=1> CHCpi_ex; // P(CHC+|Ex)
  real<lower=0,upper=1> CHCpi_non; // P(CHC+|Non)
```

```
  real<lower=0,upper=1> SVR; // SVR of DAAs among non-PWID
  real<lower=0,upper=1> SVR_PWID; // SVR of DAAs among PWID

  real<lower=0,upper=1-CHCpi_cur> HCVclear; // Probability of HCV clearan
ce; upper bound = 1-prevalence of chronic HCV
}


transformed parameters {
  // Change scales
  real<lower=0,upper=1> rho_ever;
  real<lower=0,upper=1> CHCpi_ever;
  real<lower=0,upper=1> pi_ever;
  real<lower=0,upper=1> pi_non;
  real<lower=0,upper=1> pi_cur;

  rho_ever = rho[1] + rho[2];
  CHCpi_ever = CHCpi_cur*rho[1]/rho_ever + CHCpi_ex*rho[2]/rho_ever;
  pi_ever = CHCpi_ever/(1-HCVclear);
  pi_non = CHCpi_non/(1-HCVclear);
  pi_cur = CHCpi_cur/(1-HCVclear);
}


// Binomial regression model
model {
  // Priors
  rho ~ dirichlet(alpha);

  ////////////////////////////////
  // Likelihood contributions //
  ////////////////////////////////
```

```
  // Probability of HCV clearance
  HCVclear ~ normal(HCVclear_mean,HCVclear_sd);

  // Prevalence of current use
  rho[1] ~ normal(p_cur_mean,p_cur_sd);

  // Prevalence of ex-use
  rho[2] ~ normal(p_ex_mean,p_ex_sd);

  // Prevalence of chronic HCV among current users
  //CHCpi_cur ~ normal(p_CHC_cur_mean,p_CHC_cur_sd);
  Yst_hcv_cur ~ binomial(Nst_hcv_cur,pi_cur);

  // Prevalence of HCV among ever users
  Yst_hcv_ever ~ binomial(Nst_hcv_ever,pi_ever);

  // HCV+ among non
  Yst_hcv_non ~ binomial(Nst_hcv_non,pi_non);

  // SVR of DAAs among non-PWID
  SVR ~ normal(SVR_mean,SVR_sd);

  // SVR of DAAs among PWID
  SVR_PWID ~ normal(SVR_PWID_mean,SVR_PWID_sd);
}


generated quantities {
  // Functions of parameters
  real<lower=0,upper=100> overalCHC;
  real<lower=0,upper=1> pEverGivenCHC;
```

```
  real<lower=0,upper=1> pNonGivenCHC;
  real logit_HCVclear;
  real<lower=0> NumberCHC;
  real<lower=0,upper=1> CHCDAApi_non; // P(CHC+|Non) adjusted for DAA
  real<lower=0,upper=1> CHCDAApi_cur; // P(CHC+|Cur) adjusted for DAA
  real<lower=0,upper=1> CHCDAApi_ex; // P(CHC+|Ex) adjusted for DAA
  real<lower=0,upper=1> CHCDAApi_ever; // P(CHC+|Ever) adjusted for DAA
  real logit_CHCpi_cur;
  real logit_CHCpi_ex;
  real logit_CHCpi_non;
  real logit_rho_cur;
  real logit_rho_ex;
  real logit_rho_non;


  CHCDAApi_cur = ( N1579*rho[1]*(1-HCVclear)*pi_cur - Ntrt_cur*SVR_PWID_m
ean)/(N1579*rho[1]);
  CHCDAApi_ex = ( N1579*rho[2]*CHCpi_ex - Ntrt_ex*SVR_PWID_mean)/(N1579*r
ho[2]);
  CHCDAApi_non = ( N1579*rho[3]*(1-HCVclear)*pi_non - Ntrt_non*SVR)/(N157
9*rho[3]);
  CHCDAApi_ever = CHCDAApi_cur*rho[1]/rho_ever + CHCDAApi_ex*rho[2]/rho_e
ver;

  // Overall HCV prevalence
  overalCHC = 100*(rho[1]*CHCDAApi_cur + rho[2]*CHCDAApi_ex +  rho[3]*CHC
DAApi_non);
  pEverGivenCHC = CHCDAApi_ever*rho_ever/(overalCHC/100);
  pNonGivenCHC = CHCDAApi_non*(1-rho_ever)/(overalCHC/100);
  NumberCHC = round(overalCHC*N1579/100);

  logit_rho_cur = logit(rho[1]);
```

```
  logit_rho_ex = logit(rho[2]);
  logit_rho_non = logit(rho[3]);
  logit_CHCpi_cur = logit(CHCDAApi_cur);
  logit_CHCpi_ex = logit(CHCDAApi_ex);
  logit_CHCpi_non = logit(CHCDAApi_non);
  logit_HCVclear = logit(HCVclear);

}
```

## Multi-state Markov model

```
#include <stdio.h>
#include <stdlib.h>
#include <string.h>
#include <math.h>
#include <time.h>

#define loops 100
int populationPerYearAndAge[2021][66];
double deathRatePerAge[66];
int totalPopulationPerYear[2021];

FILE *F_DeathRate=fopen("greece-deathRates.txt","r");
FILE *F_Population=fopen("greece-population.txt","r");
FILE *out;

double pa_start = 0.00012;                        // Setting Variable
double pa_step  = 0.000002;                       // Setting Variable
double pa_stop  = 0.000123;                       // Setting Variable

double pa;
double pg = 1.0/11.7;    // Assumed average active injecting career of  11
.7 years
double pk = 0.004*12.0;     // Relapse rate of 0.004/month

struct people{
    int age;
    int state;
};

char filename[100];
struct people person[50000000];
```

```
long int totalPersons=0;

void setPopulationAge()
{
    for( int j=0; j<71; j++){
        for( int i=0; i<51; i++){
            fscanf(F_Population,"%d",&populationPerYearAndAge[1950+j][15+
i]);
        }
    }
}

void setDeathRate()
{
    for( int i=0; i<51; i++){
        fscanf(F_DeathRate,"%lf",&deathRatePerAge[15+i*5]);
        for( int j=0; j<5; j++){
            deathRatePerAge[15+i*5+j] = deathRatePerAge[15+i*5];
        }
    }
}

void getTotalPopulationPerAge()
{
    for( int j=0; j<71; j++){
        for( int i=0; i<51; i++){
            totalPopulationPerYear[1950+j] += populationPerYearAndAge[195
0+j][15+i];
        }
    }
}
```

```
void printTotalPopulationPerAge()
{
    printf("---- - ----------\n");
    printf("Year - Population\n");
    printf("---- - ----------\n");
    for( int i=1950; i<2021; i++){
        printf("%d - %d\n",i,totalPopulationPerYear[i]);
    }
}

void printDeathRatePerAge()
{
    printf("--- - -----\n");
    printf("Age - Rate\n");
    printf("--- - -----\n");
    for( int i=15; i<65; i++){
        printf("%d - %lf\n",i,deathRatePerAge[i]);
    }
}

void printTotalPersonPerState( int year)
{
    int count[4]= {0,0,0,0};
    for( int i=0; i<totalPersons; i++){
        count[person[i].state]++;
    }
    //printf("%d\t%ld\t%ld\t%ld\t%ld\n",year,count[0],count[1],count[2],c
ount[3]);
    if(year==2014)
        printf("%d\t%ld\t%ld\t%ld\t%ld\t%lf\n",year,count[0],count[1],cou
nt[2],count[3],pa);
    if( year>2009){
```

```
        fprintf(out, "%d\t%ld\t%ld\t%ld\t%ld\n",year,count[0],count[1],co
unt[2],count[3]);
    }
}

void initializePopulation()
{
    int cnt=0;
    for( int j=15; j<65; j++){
        for( int i=0; i<populationPerYearAndAge[1950][j]*100; i++){
            person[cnt].age=j;
            person[cnt].state=0;
            cnt++;
        }
    }
    totalPersons = cnt;
}

bool checkRate(double rate)
{
    if (rate <0.001){
        double k = rand()/(1.00+RAND_MAX);
        if(k<0.001){
            rate = rate*1000;
        }
        else{
            return false;
        }
    }
    double k = rand()/(1.00+RAND_MAX);
    if ( k < rate )
        return true;
```

```
    else
        return false;
}

void changeStatusAndAge()
{
    for( int i=0; i<totalPersons; i++){
        // change state
        double k = rand()/(1.00+RAND_MAX);
        if( person[i].state==0){
            if ( checkRate(pa) &&  person[i].age<40){
                person[i].state=1;
            }
        }
        else if( person[i].state==1){
            if ( k < pg ){
                person[i].state=2;
            }
        }
        else if( person[i].state==2){
            if ( k < pk ){
                person[i].state=1;
            }
        }
        // After their 49th year, all active PWID are assumed to cease in
jecting
        if(person[i].age>49 && person[i].state==1){
            person[i].state=2;
        }
        // After their 64th year, remove
        if(person[i].age>64){
            person[i].state=3;
```

```
        }
        // death rate depending on age and state
        k = rand()/(1.00+RAND_MAX);
        if( person[i].state==0){
            if ( k < deathRatePerAge[person[i].age] ){
                person[i].state=3;
            }
        }
        else if( person[i].state==1){
            if ( k < deathRatePerAge[person[i].age] * 13.0 ){
                person[i].state=3;
            }
        }
        else if( person[i].state==2){
            if ( k < deathRatePerAge[person[i].age] ){
                person[i].state=3;
            }
        }
        // increase age one year
        person[i].age++;
    }
}

void addNewPersons(int year)
{
    // add new person [15 years old and non PWID]
    int cnt=totalPersons;
    for( int i=0; i<populationPerYearAndAge[year+1][15]*100; i++){
// 100 instead of 1000 for the population to reduce execution time
        person[cnt].age=15;
        person[cnt].state=0;
        cnt++;
```

```
    }
    // increase the total number of population
    totalPersons = cnt;
}
int main()
{
    srand(time(NULL));

    setPopulationAge();
    setDeathRate();

    getTotalPopulationPerAge();
    pa = pa_start;
    while ( pa < pa_stop){

        snprintf(filename, 100, "result_%lf.txt",pa);
        out=fopen(filename,"w");

        for( int iter=0; iter<loops ;iter++){
            initializePopulation();
            for( int year=1950; year<2020; year++){
                printTotalPersonPerState(year);
                changeStatusAndAge();
                addNewPersons(year);
            }
        }

        fclose(out);
        pa = pa + pa_step;
    }
```

```
}
```

# REFERENCES

European Centre for Disease Prevention and Control (ECDC). 2016. "Systematic Review on Hepatitis b and c Prevalence in the EU/EEA." https://www.ecdc.europa.eu/sites/default/files/media/en/publications/Publications/systematic-review-hepatitis-B-C-prevalence.pdf.

———. 2021. "Hepatitis c Prevalence Database." https://www.ecdc.europa.eu/en/all-topics-z/hepatitis-c/tools/hepatitis-c-prevalence-database.

Graf, Christiana, Marcus M Mücke, Georg Dultz, Kai-Henrik Peiffer, Alica Kubesch, Patrick Ingiliz, Stefan Zeuzem, Eva Herrmann, and Johannes Vermehren. 2019. "Efficacy of Direct-acting Antivirals for Chronic Hepatitis C Virus Infection in People Who Inject Drugs or Receive Opioid Substitution Therapy: A Systematic Review and Meta-analysis." *Clinical Infectious Diseases* 70 (11): 2355–65. https://doi.org/10.1093/cid/ciz696.

Grebely, Jason, Sarah Larney, Amy Peacock, Samantha Colledge, Janni Leung, Matthew Hickman, Peter Vickerman, et al. 2019. "Global, Regional, and Country-Level Estimates of Hepatitis c Infection Among People Who Have Recently Injected Drugs." *Addiction* 114 (1): 150–66. https://doi.org/https://doi.org/10.1111/add.14393.

Hines, Lindsey A., Adam Trickey, Janni Leung, Sarah Larney, Amy Peacock, Louisa Degenhardt, Samantha Colledge, et al. 2020. "Associations Between National Development Indicators and the Age Profile of People Who Inject Drugs: Results from a Global Systematic Review and Meta-Analysis." *The Lancet Global Health* 8 (1): e76–91. https://doi.org/10.1016/S2214-109X(19)30462-0.

Lampertico, Pietro, Jose A. Carrión, Michael Curry, Juan Turnes, Markus Cornberg, Francesco Negro, Ashley Brown, et al. 2020. "Real-World Effectiveness and Safety of Glecaprevir/Pibrentasvir for the Treatment of Patients with Chronic HCV Infection: A meta-Analysis." *Journal of Hepatology* 72 (6): 1112–21. https://doi.org/10.1016/j.jhep.2020.01.025.

Lin, Lifeng, and Haitao Chu. 2020. "Meta-Analysis of Proportions Using Generalized Linear Mixed Models." *Epidemiology* 31 (5).

https://journals.lww.com/epidem/Fulltext/2020/09000/Meta_analysis_of_Proportions_Using_Generalized.16.aspx.

McDonald, Scott A., Rosmawati Mohamed, Maznah Dahlui, Herlianna Naning, and Adeeba Kamarulzaman. 2014. "Bridging the Data Gaps in the Epidemiology of Hepatitis c Virus Infection in Malaysia Using Multi-Parameter Evidence Synthesis." *BMC Infectious Diseases* 14 (1): 564. https://doi.org/10.1186/s12879-014-0564-6.

Micallef, J. M., J. M. Kaldor, and G. J. Dore. 2006. "Spontaneous Viral Clearance Following Acute Hepatitis c Infection: A Systematic Review of Longitudinal Studies." *Journal of Viral Hepatitis* 13 (1): 34–41. https://doi.org/https://doi.org/10.1111/j.1365-2893.2005.00651.x.

Presanis, Anne M., O. Noel Gill, Timothy R. Chadborn, Caterina Hill, Vivian Hope, Louise Logan, Brian D. Rice, Valerie C. Delpech, A. E. Ades, and Daniela De Angelis. 2010. "Insights into the Rise in HIV Infections, 2001 to 2008: A Bayesian Synthesis of Prevalence Evidence." *AIDS* 24 (18). https://journals.lww.com/aidsonline/Fulltext/2010/11270/Insights_into_the_rise_in_HIV_infections,_2001_to.12.aspx.

Sweeting, M. J., D. De Angelis, M. Hickman, and A. E. Ades. 2008. "Estimating hepatitis C prevalence in England and Wales by synthesizing evidence from multiple data sources. Assessing data conflict and model fit." *Biostatistics* 9 (4): 715–34. https://doi.org/10.1093/biostatistics/kxn004.

Tan, Sarah, Susanna Makela, Daliah Heller, Kevin Konty, Sharon Balter, Tian Zheng, and James H. Stark. 2018. "A Bayesian Evidence Synthesis Approach to Estimate Disease Prevalence in Hard-to-Reach Populations: Hepatitis c in New York City." *Epidemics* 23: 96–109. https://doi.org/https://doi.org/10.1016/j.epidem.2018.01.002.

Thein, Hla-Hla, Qilong Yi, Gregory J. Dore, and Murray D. Krahn. 2008. "Estimation of Stage-Specific Fibrosis Progression Rates in Chronic Hepatitis c Virus Infection: A Meta-Analysis and Meta-Regression." *Hepatology* 48 (2): 418–31. https://doi.org/https://doi.org/10.1002/hep.22375.

Veen, Maaike G. van, Anne M. Presanis, Stefano Conti, Maria Xiridou, Annemarie R. Stengaard, Martin C. Donoghoe, Ard I. van Sighem, Marianne A. van der Sande, and Daniela De Angelis.

2011. "National Estimate of HIV Prevalence in the Netherlands: Comparison and Applicability of Different Estimation Tools." *AIDS* 25 (2). https://journals.lww.com/aidsonline/Fulltext/2011/01140/National_estimate_of_HIV_prevalence_in_the.14.aspx.

# Estimating the prevalence of chronic hepatitis C infection (CHC) in Lithuania using Bayesian multiparameter evidence synthesis

30/06/2022

## Table of Contents

Introduction ..... 2

Methods ..... 2

Prevalence of ex-PWID ..... 3

Prevalence of recent PWID ..... 3

Prevalence of CHC among recent PWID ..... 4

Prevalence of CHC among ex-PWID ..... 4

Prevalence of CHC among non-PWID ..... 5

Sensitivity analysis including migrants from endemic countries ..... 6

Results ..... 7

Tables and Figures ..... 9

Appendix ..... 16

Fit of the multi-state Markov model ..... 16

Stan code for Bayesian multiparameter evidence synthesis ..... 17

Multi-state Markov model ..... 23

References ..... 31

# INTRODUCTION

Patients with chronic hepatitis C (HCV) infection often remain asymptomatic for decades and even for life, but a proportion of them develop active chronic hepatitis, which is a progressive disease (Thein et al. 2008). Because of the largely asymptomatic nature of HCV infection, notification data reflect national screening and testing practices and do not give accurate insights into the prevalence of HCV infection. Although there are studies that have estimated the prevalence of HCV antibodies (anti-HCV) in specific groups such as the general population, first-time blood donors, or people who inject drugs (PWID) at drug treatment centers, those alone cannot be combined to produce national estimates unless some additional information regarding the composition of each HCV risk group and its prevalence in the population are known (Sweeting et al. 2008; McDonald et al. 2014; Tan et al. 2018).

# METHODS

Bayesian multiparameter evidence synthesis (MPES) has been a popular approach to formally estimate anti-HCV or HIV prevalence (Sweeting et al. 2008; McDonald et al. 2014; Tan et al. 2018; Presanis et al. 2010; Veen et al. 2011). In this project, we extend this method to estimating the CHC prevalence, with the population (15-79 years) split into three main non-overlapping risk groups, i.e., recent PWID (those who have injected in the last year), ex-PWID, and non-PWID. A unified model is assumed including parameters associated with the CHC prevalence of recent, ex-PWID, and non-PWID (denoted by $\pi_{rec}$, $\pi_{ex}$, and $\pi_{non}$, respectively) and the prevalence of these risk groups in the population (denoted by $\rho_{rec}$, $\rho_{ex}$, and $\rho_{non}$, respectively; $\rho_{rec} + \rho_{ex} + \rho_{non} = 1$). To estimate the CHC prevalence in the whole population, $\pi$, we used the formula:

$$\pi = \pi_{rec}\rho_{rec} + \pi_{ex}\rho_{ex} + \pi_{non}\rho_{non} \quad (1)$$

Bayesian synthesis considers all available data, allowing for an evidence-based estimation of CHC prevalence in the population, with inherent uncertainty properly accounted for. Our aim is to estimate the CHC prevalence for the total (15-79) population of Lithuania in 2019 using sources of information over the last decade (2010-2019).

## Prevalence of ex-PWID

Data on the prevalence of ex-PWID are sparse and generally often unreliable. To overcome this limitation, we apply the method proposed by McDonald et al. (2014), which requires simulating a multi-state Markov model representing the non-PWID, recent PWID, and ex-PWID. In the multi-state Markov model, the simulation starts in 1950 and examines the population aged 15-79 years old. A certain number of 15-year-old individuals enter the simulation process each year. These numbers of 15-year-old individuals are obtained from the United Nations Statistic Division UNSD, being different for each country. The ageing of the population is also taken into account. Country-specific overall mortality data are retrieved from the Life Tables of the World Health Organization WHO. The duration of injecting career is also country-specific and obtained from the paper of Hines et al. (2020).

To estimate the number of ex-PWID, the model can be calibrated on the number of recent PWID provided in the EMCDDA barometer or the EMCDDA statistical bulletin (the code of the Markov model can be found in the Appendix). If there are no available data in the barometer, estimates from the systematic review of Grebely et al. (2019) could be used. Currently, The multi-state Markov model to estimate the prevalence of recent and ex-PWID was calibrated on the estimates reported in the EMCDDA barometer, which were similar to those presented in a national report.

After applying the model for Lithuania, we compute the number (and the corresponding Confidence Interval - CI) of ex-PWID in 2019. The number of ex-PWID is then divided by the population size (15-79 years) in the same year, and a Normal distribution, constrained such that $\rho_{ex} \in (0,1)$, is assumed for $\rho_{ex}$. The standard deviation of this distribution is specified to approximately correspond to the respective CI, i.e. $\left(UB_{\rho_{ex}} - LB_{\rho_{ex}}\right)/(2 \times 1.96)$, where $UB_{\rho_{ex}}$ and $LB_{\rho_{ex}}$ denote the upper and lower limit of the CI.

## Prevalence of recent PWID

The number of recent PWID in Lithuania in 2019 is also projected by the multi-state Markov model described in the previous subsection. To estimate $\rho_{rec}$, the number of recent PWID is divided by the population size (15-79 years). Similar to the previous subsection, a Normal

distribution, constrained such that $\rho_{rec} \in (0,1)$, is assumed for $\rho_{rec}$, with the standard deviation specified to correspond to the CI obtained from the multi-state Markov model.

## Prevalence of CHC among recent PWID

The prevalence (and the 95% CI) of CHC among recent PWID was informed by national anti-HCV prevalence data on current PWID in 2018, available in the EMCDDA statistical bulletin and the national report. The Binomial distribution in the model to inform $\pi_{rec}$ was used. However, as the data reported in the EMCDDA refer to the anti-HCV prevalence and not to the CHC prevalence, they could not be used directly. This issue can be partly addressed by noting that spontaneous viral clearance occurs in approximately one in four people with acute hepatitis C (Micallef, Kaldor, and Dore 2006). To account for variability in the HCV clearance probabilities, we used the result reported in Micallef, Kaldor, and Dore (2006), i.e., the proportion of HCV clearance (denoted by $\rho_{clear}$) is equal to 0.26 (95% CI 0.22–0.29). However, some people may have been treated with direct-acting antivirals (DAAs), with the sustained virologic response ($SVR_{PWID}$) among PWID estimated to be 88% (95% CI: 80% to 93%) (Graf et al. 2019). Based on information provided by the national focal point, the number of individuals treated with DAAs in 2018-2019 is equal to 3,410. However, the proportions of each risk group among those treated with DAAs, i.e. $\rho_{rec|DAA}$, $\rho_{ex|DAA}$, and $\rho_{non|DAA}$ ($\rho_{rec|DAA} + \rho_{ex|DAA} + \rho_{non|DAA} = 1$), are not currently available in Lithuania. In this report, we make the assumption that $\rho_{rec|DAA}$ is equal to the proportion of recent PWID among CHC-positive individuals, i.e. Pr(Recent PWID|CHC), as estimated by our model when the DAA uptake is ignored (Table 2). Similarly, we assume that $\rho_{ex|DAA} = \Pr(\text{Ex-PWID}|\text{CHC})$ and $\rho_{non|DAA} = \Pr(\text{Non-PWID}|\text{CHC})$. Thus, the CHC prevalence among recent PWID, adjusted for DAAs, can be estimated by

$$\pi_{rec} = \frac{N_{15,79}\rho_{rec}\pi(\text{anti-HCV})_{rec}(1-\rho_{clear}) - N_{DAA}\rho_{rec|DAA}SVR_{PWID}}{N_{15,79}\rho_{rec}}, \quad (2)$$

where $\pi(\text{anti-HCV})_{rec}$ denotes the anti-HCV among recent PWID.

## Prevalence of CHC among ex-PWID

Information on CHC prevalence among ex-PWID (i.e., $\pi_{ex}$) is difficult to obtain directly. To overcome this, anti-HCV prevalence data among ever PWID in Vilnius in 2012 available in the

EMCDDA statistical bulletin were used. However, as these data refer to the anti-HCV prevalence, they are adjusted according to the procedure described in the previous subsection, i.e.

$$\pi_{ever} = \frac{N_{15,79}\rho_{ever}\pi(\text{anti-HCV})_{ever}(1-\rho_{clear}) - N_{DAA}\rho_{ever|DAA}SVR_{PWID}}{N_{15,79}\rho_{ever}}. \quad (3)$$

where $\pi(\text{anti-HCV})_{ever}$ denotes the anti-HCV prevalence among ever PWID, $\rho_{ever} = \rho_{rec} + \rho_{ex}$, $\rho_{clear}$ denotes the spontaneous viral clearance [assumed to be equal to 0.26 (95% CI 0.22–0.29); (Micallef, Kaldor, and Dore 2006)], and $\rho_{ever|DAA}$ the proportion of ever PWID among individuals treated with DAAs. Recall that it is assumed that $\rho_{ever|DAA}$ is assumed to be equal to Pr(Ever PWID|CHC), as estimated by our model ignoring the effect of DAAs.

Then, since an estimate of $\pi_{ever}$ is available, $\pi_{ex}$ can be indirectly estimated since $\pi_{ever}$ is equal to a weighted average of $\pi_{rec}$ and $\pi_{ex}$, as shown by the following formula

$$\pi_{ever} = \frac{\rho_{rec}}{\rho_{ever}}\pi_{rec} + \frac{\rho_{ex}}{\rho_{ever}}\pi_{ex}, \quad (4)$$

or, equivalently,

$$\pi_{ex} = \left(\pi_{ever} - \frac{\rho_{rec}}{\rho_{ever}}\pi_{rec}\right) \times \frac{\rho_{ever}}{\rho_{ex}}. \quad (5)$$

## Prevalence of CHC among non-PWID

To estimate the CHC prevalence among non-PWID in a country (after 2010), the ECDC database (European Centre for Disease Prevention and Control (ECDC) 2021) was used. The ECDC group has carefully and critically collected information from relevant studies across the EU/EEA region in cooperation with the focal points of each country. On the basis of the representativeness of each study and other factors (European Centre for Disease Prevention and Control (ECDC) 2016), a risk of bias score was assigned to each study, with higher values indicating higher-quality studies. When higher-quality studies are available in a country ($\geq 4$), they are used to estimate the overall CHC prevalence in that country among non-PWID. If there are no higher-quality studies, general population estimates with a lower quality (<4) are pooled. If no general population prevalence estimates are available, data on the prevalence of HCV in pregnant women could be used as a prevalence measure. Finally, if data on pregnant women are lacking too, first-time blood donor studies could be an option. However, if the

national focal point agrees or recommends, estimates from a neighboring country could be used instead, or we could use any other relevant information provided/suggested by the national focal point.

We directly use CHC prevalence data from studies in the general population, if available. If the data in the general population refer to anti-HCV prevalence (without any data on the viremic population), we adjust the estimates to get the CHC prevalence based on the spontaneous HCV clearance estimate of 26% and the number of individuals treated with DAAs in the general population, as previously described, i.e.

$$\pi_{non} = \frac{N_{15,79}\rho_{non}\pi(\text{anti-HCV})_{non}(1-\rho_{clear}) - N_{DAA}\rho_{non|DAA}SVR}{N_{15,79}\rho_{non}}. \quad (6)$$

where $\pi(\text{anti-HCV})_{non}$ denotes the anti-HCV prevalence among non-PWID and $SVR$ is the sustained virologic response of DAAs in the general population estimated to be 96.7% (95% CI: 95.4% to 98.1%) (Lampertico et al. 2020).

As $\rho_{rec}$ and $\rho_{ex}$ are likely to be low, $\pi_{non}$ will be the most critical factor for the estimation of the overall CHC prevalence in the population. To adjust for potential heterogeneity between the CHC prevalence estimates from different studies in the non-PWID population in a country, as a sensitivity analysis, we also apply a random-effect meta-analytic approach to pool the CHC prevalence across studies on non-PWID (Lin and Chu 2020). The random-effect approach is carried out only when at least 3 studies are available in the general population of a certain country, and the estimates are compared with the corresponding estimates from the approach assuming no heterogeneity (similar to a fixed-effect meta-analysis). If the estimates are inconsistent, possible explanations are investigated and discussed. If there is substantial heterogeneity between studies, it would help more to explore the causes; for example, following national focal point suggestions, some studies could be excluded to reduce heterogeneity.

## Sensitivity analysis including migrants from endemic countries

A sensitivity analysis including data for the viremic population among migrants based on the report of the ECDC group (European Centre for Disease Prevention and Control 2016) was carried out. Migrants from endemic countries represent 5% of the adult population in Lithuania (Table 8 in European Centre for Disease Prevention and Control (2016)), with the respective

CHC prevalence being equal to $\pi_{mig} = 2.2\%$ (Table 9 in European Centre for Disease Prevention and Control (2016)). The overall CHC prevalence is now estimated by

$$\pi = \pi_{rec}\rho_{rec} + \pi_{ex}\rho_{ex} + \pi_{mig}\rho_{mig} + \pi_{non}\rho_{non},$$

where $\rho_{rec} + \rho_{ex} + \rho_{mig} + \rho_{non} = 1$. A treatment adjustment similar to that described in the previous subsections was also performed.

# RESULTS

The above-mentioned approach was used to estimate the prevalence of CHC in 2019 (considering ages between 15-79 years: 2,210,788).

The aggregated data used by our approach are briefly presented in Table 1. The multi-state Markov model to estimate the prevalence of recent and ex-PWID was calibrated on the estimates reported in the EMCDDA barometer, which were similar to those presented in the national report. In Lithuania, there were 9 studies on first-time blood donors, which included only anti-HCV data (Grubyte 2021).
To estimate the CHC prevalence in the general population (primarily non-PWID), we used a fixed-effect meta-analytic approach, with the corresponding results, when information on DAAs is ignored, presented in Table 2. However, based on data provided by the focal point, approximately 3,410 individuals were treated with DAAs in 2018-2019 in Lithuania, with the proportion of recent PWID, ex-PWID, and non-PWID among the 3,410 treated individuals assumed to be equal to Pr(Recent PWID|CHC) ≈ 20.92% Pr(Ex-PWID|CHC) ≈ 3.89% and Pr(Non-PWID|CHC) ≈ 75.18%, respectively (Table 2).

The corresponding results accounting for the DAA uptake are presented in Table 3. The prevalence of recent and ex-PWID was low in Lithuania (about 0.38% and 1.02%, respectively) corresponding to 8,440 (95% CI: 7,900-9,050) recent PWID and 22,570 (95% CI: 21,760-23,500) ex-PWID in the population. However, the CHC prevalence in these groups was high (Table 3) being 56.1% and 3.9%, respectively. This translates to 4,731 (95% CI: 4,293-5,192) and 879 (95% CI: 172-1,694) recent and ex-PWID aged 15-79 living with CHC infection in Lithuania in 2019. The CHC prevalence in the general population was 0.77% (95% CI: 0.72%-0.83%), much lower than that of the high-risk groups. Taking all pieces of information into

account, the overall CHC prevalence in Lithuania in 2019 was equal to 1.01% (95% CI: 0.94%-1.09%), which corresponds to 22,410 (95% CI: 20,761-24,139) individuals aged 15-79 years with CHC infection.
The corresponding results under a random-effect meta-analysis for the studies in the general population were similar, although suggesting a slightly lower total CHC prevalence estimate, and are provided in Table 4.

The results from our model including migrants from endemic countries as a separate group are presented in Table 5. However, this analysis comes with possible limitations; that is, including migrants as a separate group is valid only if migrants do not overlap with the remaining groups (recent PWID, ex-PWID, and non-PWID). Thus, if migrants do participate proportionally in the study(ies) in the general population, this analysis may result in biased overall CHC estimates (most probably in higher CHC prevalence estimates).

# TABLES AND FIGURES

*Table 1. Data (real) contributing to the estimation of CHC prevalence in Lithuania in 2019.*

| Parameter | Number (95%CI) | Numerator | Denominator | Notes | Year of study |
|---|---|---|---|---|---|
| $\rho_{rec}$ | 8,440 (7,900-9,050) | | | Method based on McDonald et al. | 2019 |
| $\rho_{ex}$ | 22,570 (21,760-23,500) | | | Method based on McDonald et al. | 2019 |
| $\pi(\text{anti-HCV})_{rec}$ | | 317 | 369 | EMCDDA database | 2018 |
| $\pi(\text{anti-HCV})_{ever}$ | | 165 | 598 | EMCDDA database | 2012 |
| $\pi(\text{anti-HCV})_{non}$ | | 437 | 22,539 | (Grubyte 2021); Risk of bias=NA | 2010 |
| $\pi(\text{anti-HCV})_{non}$ | | 354 | 23,034 | (Grubyte 2021); Risk of bias=NA | 2011 |
| $\pi(\text{anti-HCV})_{non}$ | | 377 | 22,922 | (Grubyte 2021); Risk of bias=NA | 2012 |
| $\pi(\text{anti-HCV})_{non}$ | | 198 | 15,606 | (Grubyte 2021); Risk of bias=NA | 2013 |
| $\pi(\text{anti-HCV})_{non}$ | | 174 | 12,690 | (Grubyte 2021); Risk of bias=NA | 2014 |
| $\pi(\text{anti-HCV})_{non}$ | | 127 | 20,377 | (Grubyte 2021); Risk of bias=NA | 2015 |
| $\pi(\text{anti-HCV})_{non}$ | | 92 | 14,190 | (Grubyte 2021); Risk of bias=NA | 2016 |

| Parameter | Number (95%CI) | Numerator | Denominator | Notes | Year of study |
|---|---|---|---|---|---|
| $\pi(\text{anti-HCV})_{non}$ | | 85 | 13,352 | (Grubyte 2021); Risk of bias=NA | 2017 |
| $\pi(\text{anti-HCV})_{non}$ | | 38 | 13,160 | (Grubyte 2021); Risk of bias=NA | 2018 |

*Notes*: Higher risk of bias score denotes a higher-quality study (range from 0 to 6); † after excluding individuals who reported injecting drug use.

*Table 2. Results from the method assuming no heterogeneity in the estimation of the CHC prevalence in the general population from different studies (i.e., similar to a fixed-effect meta-analysis).*

| Parameter | Posterior median | Posterior sd | LB (2.5%) | UB (97.5%) |
|---|---|---|---|---|
| $\rho_{rec}$ (%) | 0.38 | 0.01 | 0.36 | 0.41 |
| $\rho_{ex}$ (%) | 1.02 | 0.02 | 0.98 | 1.06 |
| $\pi_{rec}$ (%) | 63.51 | 2.03 | 59.5 | 67.38 |
| $\pi_{ex}$ (%) | 4.42 | 1.94 | 0.87 | 8.43 |
| $\pi_{non}$ (%) | 0.88 | 0.03 | 0.83 | 0.94 |
| $\pi$ (%) | 1.16 | 0.04 | 1.09 | 1.24 |
| Number with CHC | 25,619 | 864 | 23,987 | 27,357 |
| Pr(Recent PWID\|CHC) (%) | 20.92 | 0.93 | 19.1 | 22.75 |
| Pr(Ex-PWID\|CHC) (%) | 3.89 | 1.66 | 0.78 | 7.25 |
| Pr(Non-PWID\|CHC) (%) | 75.18 | 1.29 | 72.57 | 77.61 |

*Notes*: Information on DAAs is not taken into account.

*Abbreviations*: $\rho_{rec}$, prevalence of recent PWID; $\rho_{ex}$, prevalence of ex-PWID; $\pi_{rec}$, CHC prevalence among recent PWID; $\pi_{ex}$, CHC prevalence among ex-PWID; $\pi_{non}$, CHC prevalence among non-PWID; $\pi$ overall CHC prevalence in Lithuania; LB, Lower Boundary; UP, Upper Boundary

*Table 3. Results from the method assuming no heterogeneity in the estimation of the CHC prevalence in the general population from different studies (i.e., similar to a fixed-effect meta-analysis).*

| Parameter | Posterior median | Posterior sd | LB (2.5%) | UB (97.5%) |
|---|---|---|---|---|
| $\rho_{rec}$ (%) | 0.38 | 0.01 | 0.36 | 0.41 |
| $\rho_{ex}$ (%) | 1.02 | 0.02 | 0.98 | 1.06 |
| $\pi_{rec}$ (%) | 56.1 | 1.93 | 52.3 | 59.88 |
| $\pi_{ex}$ (%) | 3.9 | 1.73 | 0.76 | 7.44 |
| $\pi_{non}$ (%) | 0.77 | 0.03 | 0.72 | 0.83 |
| $\pi$ (%) | 1.01 | 0.04 | 0.94 | 1.09 |
| Number with CHC | 22,410 | 859 | 20,761 | 24,139 |
| Pr(Recent PWID\|CHC) (%) | 21.12 | 0.95 | 19.28 | 23 |
| Pr(Ex-PWID\|CHC) (%) | 3.93 | 1.68 | 0.79 | 7.32 |
| Pr(Non-PWID\|CHC) (%) | 74.95 | 1.31 | 72.32 | 77.4 |

*Notes*: The number of individuals treated with DAAs is taken into account.

*Abbreviations*: $\rho_{rec}$, prevalence of recent PWID; $\rho_{ex}$, prevalence of ex-PWID; $\pi_{rec}$, CHC prevalence among recent PWID; $\pi_{ex}$, CHC prevalence among ex-PWID; $\pi_{non}$, CHC prevalence among non-PWID; $\pi$ overall CHC prevalence in Lithuania; LB, Lower Boundary; UP, Upper Boundary

*Table 4. Results from the method assuming heterogeneity in the estimation of the CHC prevalence in the general population from different studies (i.e., similar to a random-effect meta-analysis).*

| Parameter | Posterior median | Posterior sd | LB (2.5%) | UB (97.5%) |
|---|---|---|---|---|
| $\rho_{rec}$ (%) | 0.38 | 0.01 | 0.36 | 0.41 |
| $\rho_{ex}$ (%) | 1.02 | 0.02 | 0.98 | 1.06 |
| $\pi_{rec}$ (%) | 54.72 | 2.56 | 49.42 | 59.49 |
| $\pi_{ex}$ (%) | 3.83 | 1.68 | 0.74 | 7.37 |
| $\pi_{non}$ (%) | 0.6 | 0.21 | 0.31 | 1.1 |
| $\pi$ (%) | 0.84 | 0.21 | 0.54 | 1.35 |
| Number with CHC | 18,650 | 4,712 | 11,846 | 29,898 |
| Pr(Recent PWID\|CHC) (%) | 24.76 | 5.02 | 16.23 | 36.12 |
| Pr(Ex-PWID\|CHC) (%) | 4.56 | 2.19 | 0.88 | 9.41 |
| Pr(Non-PWID\|CHC) (%) | 70.59 | 5.95 | 57.2 | 80.69 |
| Between-study variance | 0.56 | 0.53 | 0.21 | 2.02 |

*Notes*: The number of individuals treated with DAAs is taken into account.

*Abbreviations*: $\rho_{rec}$, prevalence of recent PWID; $\rho_{ex}$, prevalence of ex-PWID; $\pi_{rec}$, CHC prevalence among recent PWID; $\pi_{ex}$, CHC prevalence among ex-PWID; $\pi_{non}$, CHC prevalence among non-PWID; $\pi$ overall CHC prevalence in Lithuania; LB, Lower Boundary; UP, Upper Boundary

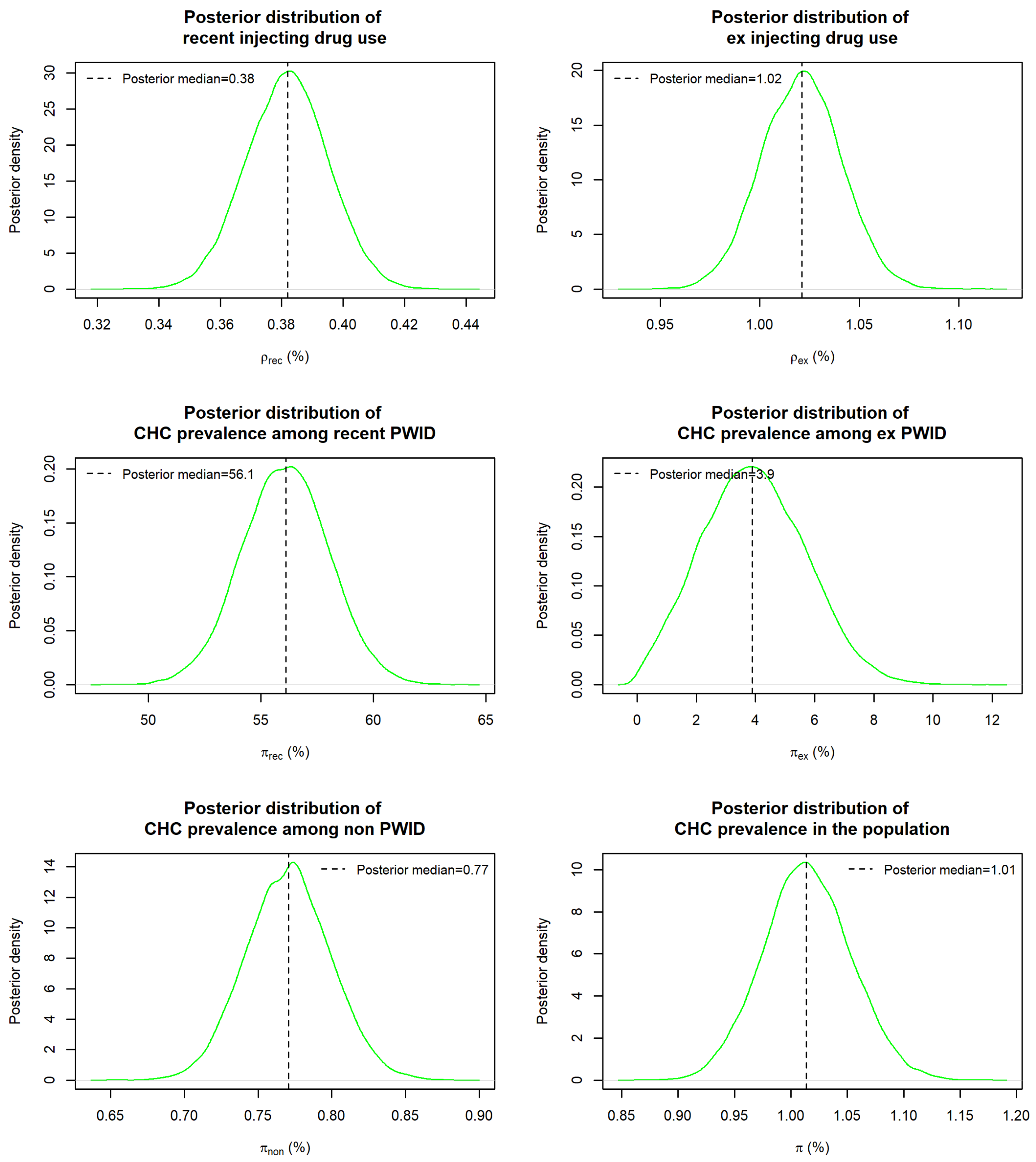


*Figure 1. Posterior distributions of the key model parameters using the approach that assumes no heterogeneity in the estimation of the CHC prevalence in the general population (primarily non-PWID) from different studies (i.e., similar to a fixed-effect meta-analysis), with the number of individuals treated with DAA taken into account.*

*Table 5. Results from the method assuming no heterogeneity in the estimation of the CHC prevalence in the general population (primarily non-PWID) from different studies (i.e., similar to a fixed-effect meta-analysis) including migrants from endemic countries as a separate group.*

| Parameter | Posterior median | Posterior sd | LB (2.5%) | UB (97.5%) |
|---|---|---|---|---|
| $\rho_{rec}$ (%) | 0.38 | 0.01 | 0.36 | 0.41 |
| $\rho_{ex}$ (%) | 1.02 | 0.02 | 0.98 | 1.06 |
| $\rho_{mig}$ (%) | 5 | 0 | 5 | 5 |
| $\pi_{rec}$ (%) | 56.49 | 1.95 | 52.73 | 60.32 |
| $\pi_{ex}$ (%) | 3.91 | 1.76 | 0.68 | 7.58 |
| $\pi_{mig}$ (%) | 1.93 | 0.54 | 0.9 | 3.02 |
| $\pi_{non}$ (%) | 0.78 | 0.03 | 0.72 | 0.83 |
| $\pi$ (%) | 1.08 | 0.05 | 0.99 | 1.18 |
| Number with CHC | 23,866 | 1,075 | 21,779 | 25,989 |
| Pr(Recent PWID\|CHC) (%) | 19.99 | 1.02 | 18.04 | 21.98 |
| Pr(Ex-PWID\|CHC) (%) | 3.71 | 1.61 | 0.65 | 6.99 |
| Pr(Mig\|CHC) (%) | 8.95 | 2.28 | 4.38 | 13.3 |
| Pr(Non-PWID\|CHC) (%) | 67.32 | 2.05 | 63.41 | 71.44 |

*Notes*: The number of individuals treated with DAAs is taken into account.

*Abbreviations*: $\rho_{rec}$, prevalence of recent PWID; $\rho_{ex}$, prevalence of ex-PWID; $\rho_{mig}$, prevalence of migrants from endemic countries; $\pi_{rec}$, CHC prevalence among recent PWID; $\pi_{ex}$, CHC prevalence among ex-PWID; $\pi_{mig}$, CHC prevalence among migrants from endemic countries; $\pi_{non}$, CHC prevalence among non-PWID; $\pi$ overall CHC prevalence in Lithuania; LB, Lower Boundary; UP, Upper Boundary

# APPENDIX

## Fit of the multi-state Markov model

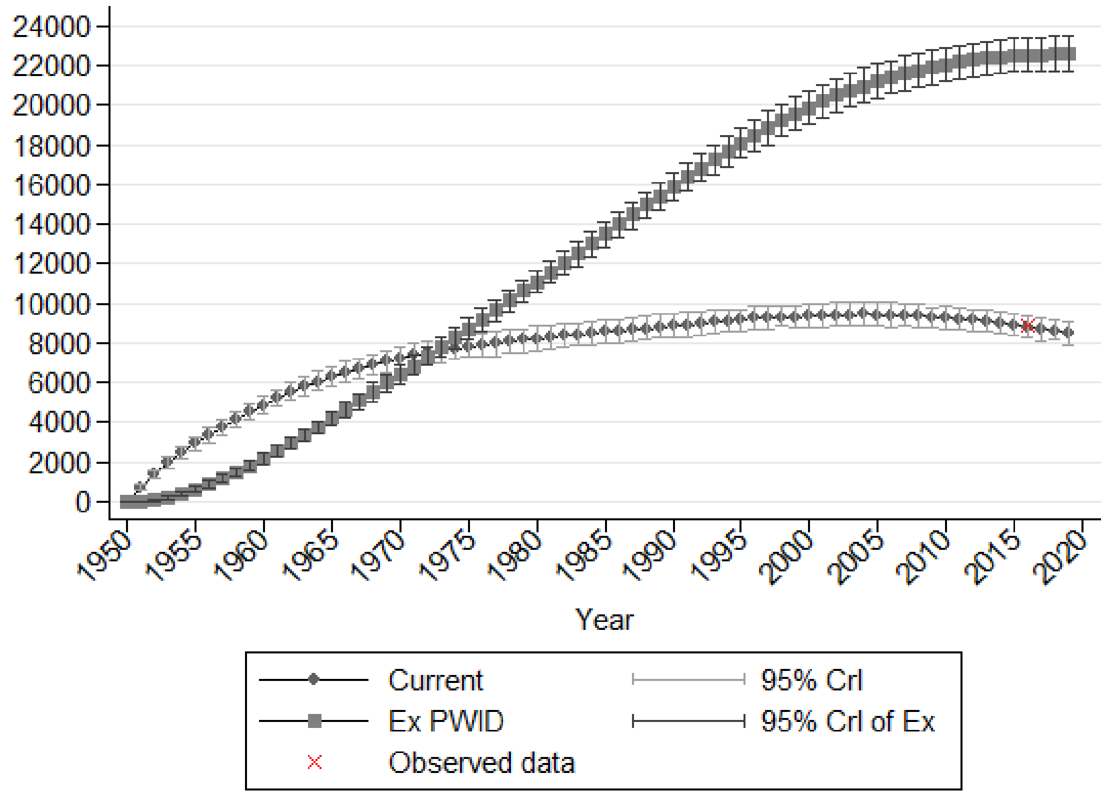


*Figure 2: Model predictions for the numbers of current and ex-PWID (people who inject drugs). The solid black line and shaded grey error bars show the median and 95% credible intervals (95% CrI) for the model projections. For comparison, asterisks indicate the observed country's data.*

## Stan code for Bayesian multiparameter evidence synthesis

```
data {
  int<lower=0> N1579; // Population of 15-79 in `Country`
  int<lower=1> Knon; // Number of studies in the ECDC data for `Country`
  int<lower=1> Kever; // Number of studies in the EMCDDA data for ever us
ers in `Country`
  int<lower=1> Kcur; // Number of studies in the EMCDDA barometer (for HC
V prevalence of PWID) in `Country`
  int<lower=1> NDAA; // Total number of DAAs from 2015 to 2019

  real p_cur_mean; // Prior mean for the prevalence of current use in `Co
untry`
  real<lower=0> p_cur_sd; // Prior sd for the prevalence of current use i
n `Country`

  real p_ex_mean; // Prior mean for the prevalence of ex-use in `Country`
  real<lower=0> p_ex_sd; // Prior sd for the prevalence of ex-use in `Cou
ntry`

  int<lower=0> Nst_hcv_cur[Kcur]; // Number of individuals in the study e
stimating anti-HCV among recent PWID
  int<lower=0> Yst_hcv_cur[Kcur]; // Number of HCV+ in the study estimati
ng anti-HCV among recent PWID
  //real p_CHC_cur_mean; // Prior mean for the CHC prevalence among recen
t PWID in `Country`
  //real<lower=0> p_CHC_cur_sd; // Prior sd for the CHC prevalence among
recent PWID in `Country`

  int<lower=0> Nst_hcv_ever[Kever]; // Number of individuals in the study
estimating anti-HCV among ever IDU
  int<lower=0> Yst_hcv_ever[Kever]; // Number of HCV+ in the study estima
ting anti-HCV among ever PWID
```

```
  int<lower=0> Nst_hcv_non[Knon]; // Number of individuals in the study e
stimating anti-HCV among non PWID
  int<lower=0> Yst_hcv_non[Knon]; // Number of HCV+ in the study estimati
ng anti-HCV among non PWID

  vector<lower=0>[3] alpha; // parameter of the Diriclet prior

  real HCVclear_mean; // Prior mean for the HCV clearance probability
  real<lower=0> HCVclear_sd; // Prior sd for the HCV clearance probabilit
y

  real SVR_mean; // Prior mean for the SVR among non-PWID
  real<lower=0> SVR_sd; // Prior sd for the SVR among non-PWID

  real SVR_PWID_mean; // Prior mean for the SVR among PWID
  real<lower=0> SVR_PWID_sd; // Prior sd for the SVR among PWID
}

// Block defining the original parameters
parameters {
  // The parameters to be sampled
  simplex[3] rho; // Prevalence of the three risk groups
  real<lower=0,upper=1> CHCpi_cur; // P(CHC+|Cur)
  real<lower=0,upper=1> CHCpi_ex; // P(CHC+|Ex)
  real<lower=0,upper=1> CHCpi_non; // P(CHC+|Non)
  real<lower=0,upper=1> SVR; // SVR of DAAs among non-PWID
  real<lower=0,upper=1> SVR_PWID; // SVR of DAAs among PWID

  real<lower=0,upper=1-CHCpi_cur> HCVclear; // Probability of HCV clearan
ce; upper bound = 1-prevalence of chronic HCV
}
```

```
transformed parameters {
  // Change scales
  real<lower=0,upper=1> rho_ever;
  real<lower=0,upper=1> CHCpi_ever;
  real<lower=0,upper=1> pi_ever;
  real<lower=0,upper=1> pi_non;
  real<lower=0,upper=1> pi_cur;

  rho_ever = rho[1] + rho[2];
  CHCpi_ever = CHCpi_cur*rho[1]/rho_ever + CHCpi_ex*rho[2]/rho_ever;
  pi_ever = CHCpi_ever/(1-HCVclear);
  pi_non = CHCpi_non/(1-HCVclear);
  pi_cur = CHCpi_cur/(1-HCVclear);
}


// Binomial regression model
model {
  // Priors
  rho ~ dirichlet(alpha);

  //////////////////////////////
  // Likelihood contributions //
  //////////////////////////////

  // Probability of HCV clearance
  HCVclear ~ normal(HCVclear_mean,HCVclear_sd);

  // Prevalence of current use
  rho[1] ~ normal(p_cur_mean,p_cur_sd);
```

```
  // Prevalence of ex-use
  rho[2] ~ normal(p_ex_mean,p_ex_sd);

  // Prevalence of chronic HCV among current users
  //CHCpi_cur ~ normal(p_CHC_cur_mean,p_CHC_cur_sd);
  Yst_hcv_cur ~ binomial(Nst_hcv_cur,pi_cur);

  // Prevalence of HCV among ever users
  Yst_hcv_ever ~ binomial(Nst_hcv_ever,pi_ever);

  // HCV+ among non
  Yst_hcv_non ~ binomial(Nst_hcv_non,pi_non);

  // SVR of DAAs among non-PWID
  SVR ~ normal(SVR_mean,SVR_sd);

  // SVR of DAAs among PWID
  SVR_PWID ~ normal(SVR_PWID_mean,SVR_PWID_sd);
}


generated quantities {
  // Functions of parameters
  real<lower=0,upper=100> overalCHC;
  real logit_CHCpi_cur;
  real logit_CHCpi_ex;
  real logit_CHCpi_non;
  real logit_rho_cur;
  real logit_rho_ex;
  real logit_rho_non;
  real<lower=0,upper=1> pEverGivenCHC;
```

```
  real<lower=0,upper=1> pCurGivenCHC;
  real<lower=0,upper=1> pExGivenCHC;
  real<lower=0,upper=1> pNonGivenCHC;
  real logit_HCVclear;
  real<lower=0> NumberCHC;

  real<lower=0,upper=1> CHCDAApi_non; // P(CHC+|Non) adjusted for DAA
  real<lower=0,upper=1> CHCDAApi_cur; // P(CHC+|Cur) adjusted for DAA
  real<lower=0,upper=1> CHCDAApi_ex; // P(CHC+|Ex) adjusted for DAA
  real<lower=0,upper=1> CHCDAApi_ever; // P(CHC+|Ever) adjusted for DAA

  real<lower=0,upper=100> overalCHC_DAA;
  real pEverGivenCHC_DAA;
  real pCurGivenCHC_DAA;
  real pExGivenCHC_DAA;
  real pNonGivenCHC_DAA;
  real<lower=0> NumberCHC_DAA;

  // Overall HCV prevalence
  overalCHC = 100*(rho[1]*CHCpi_cur + rho[2]*CHCpi_ex +  rho[3]*CHCpi_non
);
  pEverGivenCHC = CHCpi_ever*rho_ever/(overalCHC/100);
  pCurGivenCHC = CHCpi_cur*rho[1]/(overalCHC/100);
  pExGivenCHC = CHCpi_ex*rho[2]/(overalCHC/100);
  pNonGivenCHC = CHCpi_non*(1-rho_ever)/(overalCHC/100);
  NumberCHC = round(overalCHC*N1579/100);

  CHCDAApi_non = ( N1579*rho[3]*CHCpi_non - NDAA*pNonGivenCHC*SVR)/(N1579
*rho[3]);
  CHCDAApi_cur = ( N1579*rho[1]*CHCpi_cur - NDAA*pCurGivenCHC*SVR_PWID_me
an)/(N1579*rho[1]);
  CHCDAApi_ex = ( N1579*rho[2]*CHCpi_ex - NDAA*pExGivenCHC*SVR_PWID_mean)
```

```
/(N1579*rho[2]);
  CHCDAApi_ever = CHCDAApi_cur*rho[1]/rho_ever + CHCDAApi_ex*rho[2]/rho_e
ver;

  overalCHC_DAA = 100*(rho[1]*CHCDAApi_cur + rho[2]*CHCDAApi_ex +  rho[3]
*CHCDAApi_non);
  pEverGivenCHC_DAA = CHCDAApi_ever*rho_ever/(overalCHC_DAA/100);
  pCurGivenCHC_DAA = CHCDAApi_cur*rho[1]/(overalCHC_DAA/100);
  pExGivenCHC_DAA = CHCDAApi_ex*rho[2]/(overalCHC_DAA/100);
  pNonGivenCHC_DAA = CHCDAApi_non*(1-rho_ever)/(overalCHC_DAA/100);
  NumberCHC_DAA = round(overalCHC_DAA*N1579/100);

  logit_rho_cur = logit(rho[1]);
  logit_rho_ex = logit(rho[2]);
  logit_rho_non = logit(rho[3]);
  logit_CHCpi_cur = logit(CHCpi_cur);
  logit_CHCpi_ex = logit(CHCpi_ex);
  logit_CHCpi_non = logit(CHCpi_non);
  logit_HCVclear = logit(HCVclear);
}
```

## Multi-state Markov model

```
#include <stdio.h>
#include <stdlib.h>
#include <string.h>
#include <math.h>
#include <time.h>

#define loops 100
int populationPerYearAndAge[2021][66];
double deathRatePerAge[66];
int totalPopulationPerYear[2021];

FILE *F_DeathRate=fopen("`Country`-deathRates.txt","r");
FILE *F_Population=fopen("`Country`-population.txt","r");
FILE *out;

double pa_start = 0.00012;                  // Setting Variable
double pa_step  = 0.000002;                 // Setting Variable
double pa_stop  = 0.000123;                 // Setting Variable

double pa;
double pg = 1.0/11.7;   // Assumed average active injecting career of  11
.7 years
double pk = 0.004*12.0;     // Relapse rate of 0.004/month

struct people{
    int age;
    int state;
};

char filename[100];
struct people person[50000000];
```

```
long int totalPersons=0;

void setPopulationAge()
{
    for( int j=0; j<71; j++){
        for( int i=0; i<51; i++){
            fscanf(F_Population,"%d",&populationPerYearAndAge[1950+j][15+
i]);
        }
    }
}

void setDeathRate()
{
    for( int i=0; i<51; i++){
        fscanf(F_DeathRate,"%lf",&deathRatePerAge[15+i*5]);
        for( int j=0; j<5; j++){
            deathRatePerAge[15+i*5+j] = deathRatePerAge[15+i*5];
        }
    }
}

void getTotalPopulationPerAge()
{
    for( int j=0; j<71; j++){
        for( int i=0; i<51; i++){
            totalPopulationPerYear[1950+j] += populationPerYearAndAge[195
0+j][15+i];
        }
    }
}
```

```
void printTotalPopulationPerAge()
{
    printf("---- - ----------\n");
    printf("Year - Population\n");
    printf("---- - ----------\n");
    for( int i=1950; i<2021; i++){
        printf("%d - %d\n",i,totalPopulationPerYear[i]);
    }
}

void printDeathRatePerAge()
{
    printf("--- - -----\n");
    printf("Age - Rate\n");
    printf("--- - -----\n");
    for( int i=15; i<65; i++){
        printf("%d - %lf\n",i,deathRatePerAge[i]);
    }
}

void printTotalPersonPerState( int year)
{
    int count[4]= {0,0,0,0};
    for( int i=0; i<totalPersons; i++){
        count[person[i].state]++;
    }
    //printf("%d\t%ld\t%ld\t%ld\t%ld\n",year,count[0],count[1],count[2],c
ount[3]);
    if(year==2014)
        printf("%d\t%ld\t%ld\t%ld\t%ld\t%lf\n",year,count[0],count[1],cou
nt[2],count[3],pa);
    if( year>2009){
```

```
        fprintf(out, "%d\t%ld\t%ld\t%ld\t%ld\n",year,count[0],count[1],co
unt[2],count[3]);
    }
}

void initializePopulation()
{
    int cnt=0;
    for( int j=15; j<65; j++){
        for( int i=0; i<populationPerYearAndAge[1950][j]*100; i++){
            person[cnt].age=j;
            person[cnt].state=0;
            cnt++;
        }
    }
    totalPersons = cnt;
}

bool checkRate(double rate)
{
    if (rate <0.001){
        double k = rand()/(1.00+RAND_MAX);
        if(k<0.001){
            rate = rate*1000;
        }
        else{
            return false;
        }
    }
    double k = rand()/(1.00+RAND_MAX);
    if ( k < rate )
        return true;
```

```
    else
        return false;
}

void changeStatusAndAge()
{
    for( int i=0; i<totalPersons; i++){
        // change state
        double k = rand()/(1.00+RAND_MAX);
        if( person[i].state==0){
            if ( checkRate(pa) &&  person[i].age<40){
                person[i].state=1;
            }
        }
        else if( person[i].state==1){
            if ( k < pg ){
                person[i].state=2;
            }
        }
        else if( person[i].state==2){
            if ( k < pk ){
                person[i].state=1;
            }
        }
        // After their 49th year, all active PWID are assumed to cease in
jecting
        if(person[i].age>49 && person[i].state==1){
            person[i].state=2;
        }
        // After their 64th year, remove
        if(person[i].age>64){
            person[i].state=3;
```

```
        }
        // death rate depending on age and state
        k = rand()/(1.00+RAND_MAX);
        if( person[i].state==0){
            if ( k < deathRatePerAge[person[i].age] ){
                person[i].state=3;
            }
        }
        else if( person[i].state==1){
            if ( k < deathRatePerAge[person[i].age] * 13.0 ){
                person[i].state=3;
            }
        }
        else if( person[i].state==2){
            if ( k < deathRatePerAge[person[i].age] ){
                person[i].state=3;
            }
        }
        // increase age one year
        person[i].age++;
    }
}

void addNewPersons(int year)
{
    // add new person [15 years old and non PWID]
    int cnt=totalPersons;
    for( int i=0; i<populationPerYearAndAge[year+1][15]*100; i++){
// 100 instead of 1000 for the population to reduce execution time
        person[cnt].age=15;
        person[cnt].state=0;
        cnt++;
```

```
    }
    // increase the total number of population
    totalPersons = cnt;
}
int main()
{
    srand(time(NULL));

    setPopulationAge();
    setDeathRate();

    getTotalPopulationPerAge();
    pa = pa_start;
    while ( pa < pa_stop){

        snprintf(filename, 100, "result_%lf.txt",pa);
        out=fopen(filename,"w");

        for( int iter=0; iter<loops ;iter++){
            initializePopulation();
            for( int year=1950; year<2020; year++){
                printTotalPersonPerState(year);
                changeStatusAndAge();
                addNewPersons(year);
            }
        }

        fclose(out);
        pa = pa + pa_step;
    }
```

```
}
```

# REFERENCES

European Centre for Disease Prevention and Control. 2016. "Epidemiological Assessment of Hepatitis b and c Among Migrants in the EU/EEA." https://www.ecdc.europa.eu/sites/default/files/media/en/publications/Publications/epidemiological-assessment-hepatitis-B-and-C-among-migrants-EU-EEA.pdf.

European Centre for Disease Prevention and Control (ECDC). 2016. "Systematic Review on Hepatitis b and c Prevalence in the EU/EEA." https://www.ecdc.europa.eu/sites/default/files/media/en/publications/Publications/systematic-review-hepatitis-B-C-prevalence.pdf.

———. 2021. "Hepatitis c Prevalence Database." https://www.ecdc.europa.eu/en/all-topics-z/hepatitis-c/tools/hepatitis-c-prevalence-database.

Graf, Christiana, Marcus M Mücke, Georg Dultz, Kai-Henrik Peiffer, Alica Kubesch, Patrick Ingiliz, Stefan Zeuzem, Eva Herrmann, and Johannes Vermehren. 2019. "Efficacy of Direct-acting Antivirals for Chronic Hepatitis C Virus Infection in People Who Inject Drugs or Receive Opioid Substitution Therapy: A Systematic Review and Meta-analysis." *Clinical Infectious Diseases* 70 (11): 2355–65. https://doi.org/10.1093/cid/ciz696.

Grebely, Jason, Sarah Larney, Amy Peacock, Samantha Colledge, Janni Leung, Matthew Hickman, Peter Vickerman, et al. 2019. "Global, Regional, and Country-Level Estimates of Hepatitis c Infection Among People Who Have Recently Injected Drugs." *Addiction* 114 (1): 150–66. https://doi.org/https://doi.org/10.1111/add.14393.

Grubyte, Jurgita AND Nedzinskiene, Samanta AND Urboniene. 2021. "Prevalence, Incidence and Residual Risk of Transfusion Transmitted Viruses (HBV, HCV and HIV Infections) in Lithuanian Blood Donors from 2004 to 2018: The Incidence/Window-Period Model Study." *PLOS ONE* 16 (2): 1–16. https://doi.org/10.1371/journal.pone.0246704.

Hines, Lindsey A., Adam Trickey, Janni Leung, Sarah Larney, Amy Peacock, Louisa Degenhardt, Samantha Colledge, et al. 2020. "Associations Between National Development Indicators and the Age Profile of People Who Inject Drugs: Results from a Global Systematic Review and Meta-

Analysis." *The Lancet Global Health* 8 (1): e76–91. https://doi.org/10.1016/S2214-109X(19)30462-0.

Lampertico, Pietro, Jose A. Carrión, Michael Curry, Juan Turnes, Markus Cornberg, Francesco Negro, Ashley Brown, et al. 2020. "Real-World Effectiveness and Safety of Glecaprevir/Pibrentasvir for the Treatment of Patients with Chronic HCV Infection: A meta-Analysis." *Journal of Hepatology* 72 (6): 1112–21. https://doi.org/10.1016/j.jhep.2020.01.025.

Lin, Lifeng, and Haitao Chu. 2020. "Meta-Analysis of Proportions Using Generalized Linear Mixed Models." *Epidemiology* 31 (5). https://journals.lww.com/epidem/Fulltext/2020/09000/Meta_analysis_of_Proportions_Using_Generalized.16.aspx.

McDonald, Scott A., Rosmawati Mohamed, Maznah Dahlui, Herlianna Naning, and Adeeba Kamarulzaman. 2014. "Bridging the Data Gaps in the Epidemiology of Hepatitis c Virus Infection in Malaysia Using Multi-Parameter Evidence Synthesis." *BMC Infectious Diseases* 14 (1): 564. https://doi.org/10.1186/s12879-014-0564-6.

Micallef, J. M., J. M. Kaldor, and G. J. Dore. 2006. "Spontaneous Viral Clearance Following Acute Hepatitis c Infection: A Systematic Review of Longitudinal Studies." *Journal of Viral Hepatitis* 13 (1): 34–41. https://doi.org/https://doi.org/10.1111/j.1365-2893.2005.00651.x.

Presanis, Anne M., O. Noel Gill, Timothy R. Chadborn, Caterina Hill, Vivian Hope, Louise Logan, Brian D. Rice, Valerie C. Delpech, A. E. Ades, and Daniela De Angelis. 2010. "Insights into the Rise in HIV Infections, 2001 to 2008: A Bayesian Synthesis of Prevalence Evidence." *AIDS* 24 (18). https://journals.lww.com/aidsonline/Fulltext/2010/11270/Insights_into_the_rise_in_HIV_infections,_2001_to.12.aspx.

Sweeting, M. J., D. De Angelis, M. Hickman, and A. E. Ades. 2008. "Estimating hepatitis C prevalence in England and Wales by synthesizing evidence from multiple data sources. Assessing data conflict and model fit." *Biostatistics* 9 (4): 715–34. https://doi.org/10.1093/biostatistics/kxn004.

Tan, Sarah, Susanna Makela, Daliah Heller, Kevin Konty, Sharon Balter, Tian Zheng, and James H. Stark. 2018. "A Bayesian Evidence Synthesis Approach to Estimate Disease Prevalence in Hard-to-Reach Populations: Hepatitis c in New York City." *Epidemics* 23: 96–109. https://doi.org/https://doi.org/10.1016/j.epidem.2018.01.002.

Thein, Hla-Hla, Qilong Yi, Gregory J. Dore, and Murray D. Krahn. 2008. "Estimation of Stage-Specific Fibrosis Progression Rates in Chronic Hepatitis c Virus Infection: A Meta-Analysis and Meta-Regression." *Hepatology* 48 (2): 418–31. https://doi.org/https://doi.org/10.1002/hep.22375.

Veen, Maaike G. van, Anne M. Presanis, Stefano Conti, Maria Xiridou, Annemarie R. Stengaard, Martin C. Donoghoe, Ard I. van Sighem, Marianne A. van der Sande, and Daniela De Angelis. 2011. "National Estimate of HIV Prevalence in the Netherlands: Comparison and Applicability of Different Estimation Tools." *AIDS* 25 (2). https://journals.lww.com/aidsonline/Fulltext/2011/01140/National_estimate_of_HIV_prevalence_in_the.14.aspx.

# Estimating the prevalence of chronic hepatitis C infection (CHC) in Luxembourg using Bayesian multiparameter evidence synthesis

19/07/2022

## Table of Contents


Introduction ..... 2

Methods ..... 2

- Prevalence of ex-PWID ..... 3
- Prevalence of recent PWID ..... 3
- Prevalence of CHC among recent PWID ..... 4
- Prevalence of CHC among ex-PWID ..... 4
- Prevalence of CHC among non-PWID ..... 5
- Sensitivity analysis including migrants from endemic countries ..... 6

Results ..... 7

Tables and Figures ..... 9

Appendix ..... 17

- Fit of the multi-state Markov model ..... 17
- Stan code for Bayesian multiparameter evidence synthesis ..... 18
- Multi-state Markov model ..... 24

References ..... 32

# INTRODUCTION

Patients with chronic hepatitis C (HCV) infection often remain asymptomatic for decades and even for life, but a proportion of them develop active chronic hepatitis, which is a progressive disease (Thein et al. 2008). Because of the largely asymptomatic nature of HCV infection, notification data reflect national screening and testing practices and do not give accurate insights into the prevalence of HCV infection. Although there are studies that have estimated the prevalence of HCV antibodies (anti-HCV) in specific groups such as the general population, first-time blood donors, or people who inject drugs (PWID) at drug treatment centers, those alone cannot be combined to produce national estimates unless some additional information regarding the composition of each HCV risk group and its prevalence in the population are known (Sweeting et al. 2008; McDonald et al. 2014; Tan et al. 2018).

# METHODS

Bayesian multiparameter evidence synthesis (MPES) has been a popular approach to formally estimate anti-HCV or HIV prevalence (Sweeting et al. 2008; McDonald et al. 2014; Tan et al. 2018; Presanis et al. 2010; Veen et al. 2011). In this project, we extend this method to estimating the prevalence of chronic hepatitis C infection (CHC), defined as an HCV-RNA positive result [i.e., active (viremic) infection is used as a proxy of chronic disease], with the population (15-79 years) split into three main non-overlapping risk groups, i.e., recent PWID (those who have injected in the last year; without including probably those injecting for chemsex), ex-PWID, and non-PWID (those who have never injected drugs in their lifetime). A unified model is assumed including parameters associated with the CHC prevalence of recent, ex-PWID, and non-PWID (denoted by $\pi_{rec}$, $\pi_{ex}$, and $\pi_{non}$, respectively) and the prevalence of these risk groups in the population (denoted by $\rho_{rec}$, $\rho_{ex}$, and $\rho_{non}$, respectively; $\rho_{rec} + \rho_{ex} + \rho_{non} = 1$). To estimate the CHC prevalence in the whole population, $\pi$, we used the formula:

$$\pi = \pi_{rec}\rho_{rec} + \pi_{ex}\rho_{ex} + \pi_{non}\rho_{non} \quad (1)$$

Bayesian synthesis considers all available data, allowing for an evidence-based estimation of CHC prevalence in the population, with inherent uncertainty properly accounted for. Our aim is to estimate the CHC prevalence for the total (15-79) population of Luxembourg in 2019 using sources of information over the last decade (2010-2019).

## Prevalence of ex-PWID

Data on the prevalence of ex-PWID are sparse and generally often unreliable. To overcome this limitation, we apply the method proposed by McDonald et al. (2014), which requires simulating a multi-state Markov model representing the non-PWID, recent PWID, and ex-PWID. In the multi-state Markov model, the simulation starts in 1950 and examines the population aged 15-79 years old. A certain number of 15-year-old individuals enter the simulation process each year. These numbers of 15-year-old individuals are obtained from the United Nations Statistic Division UNSD, being different for each country. The ageing of the population is also taken into account. Country-specific overall mortality data are retrieved from the Life Tables of the World Health Organization WHO. The duration of injecting career is also country-specific and obtained from the paper of Trickey et al. (2019).

To estimate the number of ex-PWID, the model can be calibrated on the number of recent PWID provided in the EMCDDA barometer or the EMCDDA statistical bulletin (the code of the Markov model can be found in the Appendix). If there are no available data in the barometer, estimates from the systematic review of Grebely et al. (2019) could be used. Currently, the multi-state Markov model to estimate the prevalence of recent and ex-PWID was calibrated on the estimates reported in the EMCDDA barometer.

After applying the model for Luxembourg, we compute the number (and the corresponding Confidence Interval - CI) of ex-PWID in 2019. The number of ex-PWID is then divided by the population size (15-79 years) in the same year, and a Normal distribution, constrained such that $\rho_{ex} \in (0,1)$, is assumed for $\rho_{ex}$. The standard deviation of this distribution is specified to approximately correspond to the respective CI, i.e. $(UB_{\rho_{ex}} - LB_{\rho_{ex}})/(2 \times 1.96)$, where $UB_{\rho_{ex}}$ and $LB_{\rho_{ex}}$ denote the upper and lower limit of the CI.

## Prevalence of recent PWID

The number of recent PWID in Luxembourg in 2019 is also projected by the multi-state Markov model described in the previous subsection. To estimate $\rho_{rec}$, the number of recent PWID is divided by the population size (15-79 years). Similar to the previous subsection, a Normal distribution, constrained such that $\rho_{rec} \in (0,1)$, is assumed for $\rho_{rec}$, with the standard deviation specified to correspond to the CI obtained from the multi-state Markov model.

## Prevalence of CHC among recent PWID

The prevalence of CHC among recent PWID was informed by unpublished CHC prevalence data provided through personal communication by the national contact point in 2015-2019. However, some people may have been treated with direct-acting antivirals (DAAs), with the sustained virologic response ($SVR_{PWID}$) among PWID estimated to be 88% (95% CI: 80% to 93%) (Graf et al. 2019). Based on the information provided by the national contact point, the numbers of individuals treated with DAAs in 2016, 2017, 2018, and 2019 are equal to 301, 203, 95, and 136, respectively. However, the proportions of each risk group among those treated with DAAs, i.e. $\rho_{rec|DAA}$, $\rho_{ex|DAA}$, and $\rho_{non|DAA}$ ($\rho_{rec|DAA} + \rho_{ex|DAA} + \rho_{non|DAA} = 1$), are not currently available in Luxembourg. In this report, we make the assumption that $\rho_{rec|DAA}$ is equal to the proportion of recent PWID among CHC-positive individuals, i.e. Pr(Recent PWID|CHC), as estimated by our model when the DAA uptake is ignored (Table 2). Similarly, we assume that $\rho_{ex|DAA} = \text{Pr(Ex-PWID|CHC)}$ and $\rho_{non|DAA} = \text{Pr(Non-PWID|CHC)}$. Thus, the CHC prevalence among recent PWID, adjusted for DAAs, can be estimated by

$$\pi_{rec} = \frac{N_{15,79}\rho_{rec}\tilde{\pi}_{rec} - (95 + 136)\rho_{rec|DAA}SVR_{PWID}}{N_{15,79}\rho_{rec}}, \quad (2)$$

where $\tilde{\pi}_{rec}$ denotes the CHC estimate among recent PWID derived solely from the CHC data provided by the national contact point in 2015-2019.

## Prevalence of CHC among ex-PWID

Information on CHC prevalence among ex-PWID (i.e., $\pi_{ex}$) is difficult to obtain directly. To overcome this, national anti-HCV prevalence data from ever PWID in 2018, available in the EMCDDA statistical bulletin and the Réseau Luxembourgeois d'Information sur les Stupéfiants et les Toxicomanies (RELIS) report, were used. However, as these data refer to the anti-HCV prevalence, they should be adjusted similarly to the procedure described in the previous subsection, i.e.

$$\pi_{ever} = \frac{N_{15,79}\rho_{ever}\pi(\text{anti-HCV})_{ever}(1 - \rho_{clear}) - N_{DAA}\rho_{ever|DAA}SVR_{PWID}}{N_{15,79}\rho_{ever}}. \quad (3)$$

where $N_{DAA}$ denotes the total number of individuals treated with DAAs up to 2019, $\pi(\text{anti-HCV})_{ever}$ denotes the anti-HCV prevalence among ever PWID, $\rho_{ever} = \rho_{rec} + \rho_{ex}$, $\rho_{clear}$ denotes the spontaneous viral clearance [assumed to be equal to 0.26 (95% CI 0.22–0.29); (Micallef, Kaldor, and Dore 2006)], and $\rho_{ever|DAA}$ the proportion of ever PWID among individuals treated with DAAs. Recall that it is assumed that $\rho_{ever|DAA}$ is assumed to be equal to Pr(Ever PWID|CHC), as estimated by our model ignoring the effect of DAAs.

Then, since an estimate of $\pi_{ever}$ is available, $\pi_{ex}$ can be indirectly estimated since $\pi_{ever}$ is equal to a weighted average of $\pi_{rec}$ and $\pi_{ex}$, as shown by the following formula

$$\pi_{ever} = \frac{\rho_{rec}}{\rho_{ever}}\pi_{rec} + \frac{\rho_{ex}}{\rho_{ever}}\pi_{ex}, \quad (4)$$

or, equivalently,

$$\pi_{ex} = \left(\pi_{ever} - \frac{\rho_{rec}}{\rho_{ever}}\pi_{rec}\right) \times \frac{\rho_{ever}}{\rho_{ex}}. \quad (5)$$

## Prevalence of CHC among non-PWID

To estimate the CHC prevalence among non-PWID in a country (after 2010), the ECDC database (European Centre for Disease Prevention and Control (ECDC) 2021) was used. The ECDC group has carefully and critically collected information from relevant studies across the EU/EEA region in cooperation with the contact points of each country. On the basis of the representativeness of each study and other factors (European Centre for Disease Prevention and Control (ECDC) 2016), a risk of bias score was assigned to each study, with higher values indicating higher-quality studies. When higher-quality studies are available in a country (≥ 4), they are used to estimate the overall CHC prevalence in that country among non-PWID. If there are no higher-quality studies, general population estimates with a lower quality (<4) are pooled. If no general population prevalence estimates are available, data on the prevalence of HCV in pregnant women could be used as a prevalence measure. Finally, if data on pregnant women are lacking too, first-time blood donor studies could be an option. However, if the national contact point agrees or recommends, estimates from a neighboring country could be used instead, or we could use any other relevant information provided/suggested by the national contact point.

We directly use CHC prevalence data from studies in the general population, if available. If the data in the general population refer to anti-HCV prevalence (without any data on the viremic population), we adjust the estimates to get the CHC prevalence based on the spontaneous HCV clearance estimate of 26% and the number of individuals treated with DAAs in the general population, as previously described, i.e.

$$\pi_{non} = \frac{N_{15,79}\rho_{non}\pi(\text{anti-HCV})_{non}(1-\rho_{clear}) - N_{DAA}\rho_{non|DAA}SVR}{N_{15,79}\rho_{non}}, \quad (6)$$

where $\pi(\text{anti-HCV})_{non}$ denotes the anti-HCV prevalence among non-PWID and $SVR$ is the sustained virologic response of DAAs in the general population estimated to be 96.7% (95% CI: 95.4% to 98.1%) (Lampertico et al. 2020).

As $\rho_{rec}$ and $\rho_{ex}$ are likely to be low, $\pi_{non}$ will be the most critical factor for the estimation of the overall CHC prevalence in the population. To adjust for potential heterogeneity between the CHC prevalence estimates from different studies in the non-PWID population in a country, as a sensitivity analysis, we also apply a random-effect meta-analytic approach to pool the CHC prevalence across studies on non-PWID (Lin and Chu 2020). The random-effect approach is carried out only when at least 3 studies are available in the general population of a certain country, and the estimates are compared with the corresponding estimates from the approach assuming no heterogeneity (similar to a fixed-effect meta-analysis). If the estimates are inconsistent, possible explanations are investigated and discussed. If there is substantial heterogeneity between studies, it would help more to explore the causes; for example, following national contact point suggestions, some studies could be excluded to reduce heterogeneity.

## Sensitivity analysis including migrants from endemic countries

A sensitivity analysis including data for the viremic population among migrants based on the report of the ECDC group (European Centre for Disease Prevention and Control 2016) was carried out. Migrants from endemic countries represent 25% of the adult population in Luxembourg (Table 8 in European Centre for Disease Prevention and Control (2016)), with the respective CHC prevalence being equal to $\pi_{mig}$ = 1.6% (Table 9 in European Centre for Disease Prevention and Control (2016)). The overall CHC prevalence is now estimated by

$$\pi = \rho_{rec}\pi_{rec} + \pi_{ex}\rho_{ex} + \pi_{mig}\rho_{mig} + \pi_{non}\rho_{non},$$

where $\rho_{rec} + \rho_{ex} + \rho_{mig} + \rho_{non} = 1$. A treatment adjustment similar to that described in the previous subsections was also performed.

As a second sensitivity analysis, we used anti-HCV data among migrants applicant for international protection, provided by the national contact point, to inform $\pi_{mig}$. The corresponding number (population size) of this group in Luxembourg in 2019 was obtained by the Luxembourg refugee statistics for 2019.

# RESULTS

The above-mentioned approach was used to estimate the prevalence of CHC in 2019 (considering ages between 15-79 years: 491,005).

The aggregated data used by our approach are briefly presented in Table 1. The multi-state Markov model to estimate the prevalence of recent and ex-PWID was calibrated on the estimates reported in the EMCDDA barometer (800 recent PWID in 2018). A very similar estimation has also been reported in the (RELIS) report (822 PWID) in 2019. In Luxembourg, there were 4 studies on first-time blood donors, which included only anti-HCV. To estimate the CHC prevalence in the general population (primarily non-PWID), we used a fixed-effect meta-analytic approach, with the corresponding results, when information on DAAs is ignored, presented in Table 2. However, based on data provided by the contact point, approximately 735 individuals were treated with DAAs from 2016 to 2019 in Luxembourg, with the proportion of recent PWID, ex-PWID, and non-PWID among the 735 treated individuals assumed to be equal to Pr(Recent PWID|CHC) ≈ 19.76% Pr(Ex-PWID|CHC) ≈ 49.32% and Pr(Non-PWID|CHC) ≈ 30.5%, respectively (Table 2).

The corresponding results accounting for the DAA uptake are presented in Table 3. The prevalence of recent and ex-PWID was low in Luxembourg (about 0.17% and 0.39%, respectively) corresponding to 815 (95% CI: 650-1,000) recent PWID and 1,920 (95% CI: 1,700-2,230) ex-PWID in the population. However, the CHC prevalence in these groups was substantial (Table 3) being 39.17% and 30.22%, respectively. This translates to 320 (95% CI: 245-401) and 579 (95% CI: 297-882) recent and ex-PWID aged 15-79 living with CHC

infection in Luxembourg in 2019. The CHC prevalence in the general population was 0.07% (95% CI: 0.02%-0.16%), much lower than that of the high-risk groups. Taking all pieces of information into account, the overall CHC prevalence in Luxembourg in 2019 was equal to 0.25% (95% CI: 0.15%-0.39%), which corresponds to 1,243 (95% CI: 760-1,894) individuals aged 15-79 years with CHC infection. The corresponding results under a random-effect meta-analysis for the studies in the general population were similar and are provided in Table 4. However, estimates from the random-effect approach had much higher uncertainty, mainly due to the low number of studies (4) and the considerable heterogeneity in the estimates, which is also reflected in the estimate of the between-study variance (Table 4).

The results from our model including migrants from endemic countries as a separate group are presented in Table 5. However, this analysis comes with possible limitations; that is, including migrants as a separate group is valid only if migrants do not overlap with the remaining groups (recent PWID, ex-PWID, and non-PWID). Thus, if migrants do participate proportionally in the study(ies) in the general population, this analysis may result in biased overall CHC estimates (most probably in higher CHC prevalence estimates).

The results from the sensitivity analysis including migrants applicant for international protection as a separate risk group are presented in Table 6. As mentioned by the national contact point, this analysis has a much smaller chance of overlapping between groups since migrants who came to work in Luxembourg without legal documents are rarely present in drug treatment sites.

# TABLES AND FIGURES

*Table 1. Data (real) contributing to the estimation of CHC prevalence in Luxembourg in 2019.*

| Parameter | Estimate (95%CI) | Number (95%CI) | Numerator | Denominator | Notes | Year of study |
|---|---|---|---|---|---|---|
| $\rho_{rec}$ | | 815 (650-1,000) | | | Method based on McDonald et al. | 2019 |
| $\rho_{ex}$ | | 1,920 (1,700-2,230) | | | Method based on McDonald et al. | 2019 |
| $\pi_{rec}$ | | | 203 | 460 | Focal point personal communication | 2015-2019 |
| $\pi(\text{anti-HCV})_{ever}$ | | | 22 | 35 | EMCDDA database and (RELIS) report | 2018 |
| $\pi(\text{anti-HCV})_{mig}$ | | | 11 | 1,124 | National AIDS Committee report 2020 | 2020 |
| $\pi(\text{anti-HCV})_{non}$ | | | 2 | 907 | ECDC database (first-time blood donors) | 2011 |
| $\pi(\text{anti-HCV})_{non}$ | | | 2 | 1,106 | ECDC database (first-time blood donors) | 2012 |
| $\pi(\text{anti-HCV})_{non}$ | | | 0 | 793 | ECDC database (first-time blood donors) | 2013 |

| Parameter | Estimate (95%CI) | Number (95%CI) | Numerator | Denominator | Notes | Year of study |
|---|---|---|---|---|---|---|
| $\pi(\text{anti-HCV})_{non}$ | | | 1† | 933 | CENTRE DE TRANSFUSION SANGUINE RAPPORT D'ACTIVITE (2019) | 2019 |

*Notes*: Although it looks counter-intuitive, **a higher risk of bias score denotes a higher-quality study** (range from 0 to 6); † Assumed to be a first-time blood donor.

*Table 2. Results from the method assuming no heterogeneity in the estimation of the CHC prevalence in the general population from different studies (i.e., similar to a fixed-effect meta-analysis).*

| Parameter | Posterior median | Posterior sd | LB (2.5%) | UB (97.5%) |
|---|---|---|---|---|
| $\rho_{rec}$ (%) | 0.17 | 0.02 | 0.13 | 0.2 |
| $\rho_{ex}$ (%) | 0.39 | 0.03 | 0.34 | 0.44 |
| $\pi_{rec}$ (%) | 44.12 | 2.29 | 39.7 | 48.63 |
| $\pi_{ex}$ (%) | 47.06 | 8.5 | 29.62 | 62.9 |
| $\pi_{non}$ (%) | 0.11 | 0.05 | 0.04 | 0.23 |
| $\pi$ (%) | 0.37 | 0.06 | 0.27 | 0.5 |
| Number with CHC | 1,820 | 299 | 1,311 | 2,476 |
| Pr(Recent PWID\|CHC) (%) | 19.76 | 3.87 | 13.54 | 28.62 |
| Pr(Ex-PWID\|CHC) (%) | 49.32 | 7.84 | 33.47 | 63.88 |
| Pr(Non-PWID\|CHC) (%) | 30.5 | 8.96 | 14.07 | 48.84 |

*Notes*: Information on DAAs is not taken into account.

*Abbreviations*: $\rho_{rec}$, prevalence of recent PWID (proportion of the population that is recent PWID); $\rho_{ex}$, prevalence of ex-PWID (proportion of the population that is ex-PWID); $\pi_{rec}$, CHC prevalence among recent PWID; $\pi_{ex}$, CHC prevalence among ex-PWID; $\pi_{non}$, CHC prevalence among non-PWID; $\pi$ overall CHC prevalence in Luxembourg; LB, Lower Boundary; UP, Upper Boundary

*Table 3. Results from the method assuming no heterogeneity in the estimation of the CHC prevalence in the general population from different studies (i.e., similar to a fixed-effect meta-analysis).*

| Parameter | Posterior median | Posterior sd | LB (2.5%) | UB (97.5%) |
|---|---|---|---|---|
| $\rho_{rec}$ (%) | 0.17 | 0.02 | 0.13 | 0.2 |
| $\rho_{ex}$ (%) | 0.39 | 0.03 | 0.34 | 0.45 |
| $\pi_{rec}$ (%) | 39.17 | 2.2 | 34.81 | 43.48 |
| $\pi_{ex}$ (%) | 30.22 | 7.4 | 15.84 | 44.75 |
| $\pi_{non}$ (%) | 0.07 | 0.04 | 0.02 | 0.16 |
| $\pi$ (%) | 0.25 | 0.06 | 0.15 | 0.39 |
| Number with CHC | 1,243 | 288 | 760 | 1,894 |
| Pr(Recent PWID\|CHC) (%) | 25.79 | 6.07 | 16.81 | 40.32 |
| Pr(Ex-PWID\|CHC) (%) | 46.36 | 7.28 | 31.3 | 59.64 |
| Pr(Non-PWID\|CHC) (%) | 26.91 | 8.86 | 11.54 | 45.64 |

*Notes*: The number of individuals treated with DAAs is taken into account.

*Abbreviations*: $\rho_{rec}$, prevalence of recent PWID (proportion of the population that is recent PWID); $\rho_{ex}$, prevalence of ex-PWID (proportion of the population that is ex-PWID); $\pi_{rec}$, CHC prevalence among recent PWID; $\pi_{ex}$, CHC prevalence among ex-PWID; $\pi_{non}$, CHC prevalence among non-PWID; $\pi$ overall CHC prevalence in Luxembourg; LB, Lower Boundary; UP, Upper Boundary

*Table 4. Results from the method assuming heterogeneity in the estimation of the CHC prevalence in the general population from different studies (i.e., similar to a random-effect meta-analysis).*

| Parameter | Posterior median | Posterior sd | LB (2.5%) | UB (97.5%) |
|---|---|---|---|---|
| $\rho_{rec}$ (%) | 0.17 | 0.02 | 0.13 | 0.2 |
| $\rho_{ex}$ (%) | 0.39 | 0.03 | 0.34 | 0.45 |
| $\pi_{rec}$ (%) | 38.49 | 2.35 | 34.08 | 43.32 |
| $\pi_{ex}$ (%) | 28.26 | 7.7 | 13.92 | 43.98 |
| $\pi_{non}$ (%) | 0.04 | 0.18 | 0 | 0.24 |
| $\pi$ (%) | 0.22 | 0.19 | 0.12 | 0.46 |
| Number with CHC | 1,062 | 938 | 605 | 2,275 |
| Pr(Recent PWID\|CHC) (%) | 29.7 | 8.5 | 14.34 | 48.39 |
| Pr(Ex-PWID\|CHC) (%) | 49.87 | 9.28 | 28.39 | 64.95 |
| Pr(Non-PWID\|CHC) (%) | 18.41 | 13.5 | 2.22 | 53.98 |
| Between-study variance | 1.19 | 2.63 | 0.03 | 9.9 |

*Notes*: The number of individuals treated with DAAs is taken into account.

*Abbreviations*: $\rho_{rec}$, prevalence of recent PWID (proportion of the population that is recent PWID); $\rho_{ex}$, prevalence of ex-PWID (proportion of the population that is ex-PWID); $\pi_{rec}$, CHC prevalence among recent PWID; $\pi_{ex}$, CHC prevalence among ex-PWID; $\pi_{non}$, CHC prevalence among non-PWID; $\pi$ overall CHC prevalence in Luxembourg; LB, Lower Boundary; UP, Upper Boundary

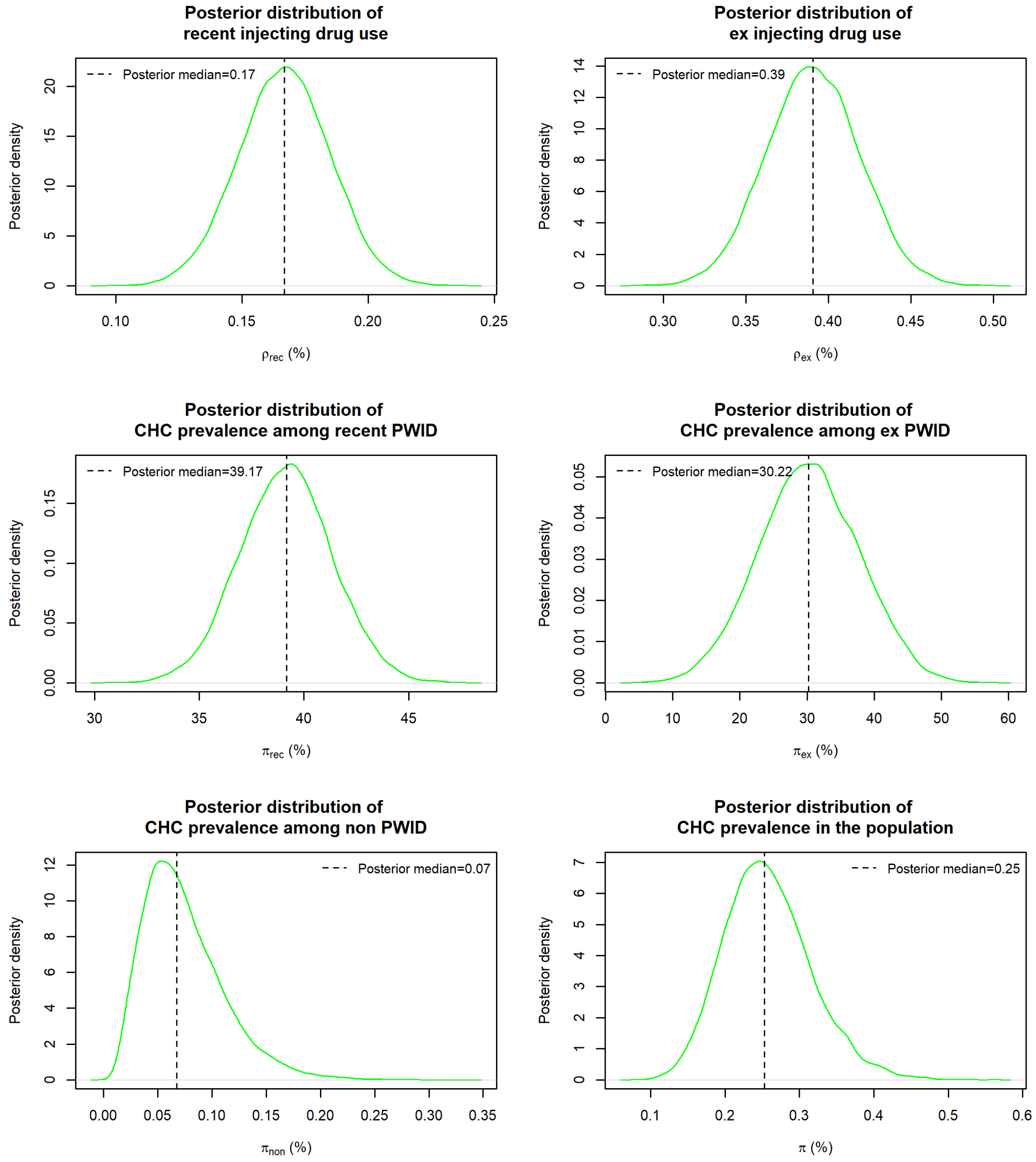


*Figure 1. Posterior distributions of the key model parameters using the approach that assumes no heterogeneity in the estimation of the CHC prevalence in the general population (primarily non-PWID) from different studies (i.e., similar to a fixed-effect meta-analysis), with the number of individuals treated with DAA taken into account.*

*Table 5. Results from the method assuming no heterogeneity in the estimation of the CHC prevalence in the general population (primarily non-PWID) from different studies (i.e., similar to a fixed-effect meta-analysis) including migrants from endemic countries as a separate group.*

| Parameter | Posterior median | Posterior sd | LB (2.5%) | UB (97.5%) |
|---|---|---|---|---|
| $\rho_{rec}$ (%) | 0.17 | 0.02 | 0.13 | 0.2 |
| $\rho_{ex}$ (%) | 0.39 | 0.03 | 0.34 | 0.44 |
| $\rho_{mig}$ (%) | 25 | 0 | 25 | 25 |
| $\pi_{rec}$ (%) | 41.63 | 2.24 | 37.28 | 46.09 |
| $\pi_{ex}$ (%) | 38.37 | 7.63 | 23.31 | 52.95 |
| $\pi_{mig}$ (%) | 1.29 | 0.46 | 0.43 | 2.22 |
| $\pi_{non}$ (%) | 0.09 | 0.04 | 0.03 | 0.19 |
| $\pi$ (%) | 0.61 | 0.13 | 0.35 | 0.88 |
| Number with CHC | 3,019 | 659 | 1,737 | 4,312 |
| Pr(Recent PWID\|CHC) (%) | 11.33 | 3.12 | 7.39 | 19.53 |
| Pr(Ex-PWID\|CHC) (%) | 24.49 | 5.98 | 15.05 | 38.73 |
| Pr(Mig\|CHC) (%) | 52.6 | 9.66 | 28.8 | 66.71 |
| Pr(Non-PWID\|CHC) (%) | 11.12 | 4.73 | 4.43 | 22.62 |

*Notes*: The number of individuals treated with DAAs is taken into account.

*Abbreviations*: $\rho_{rec}$, prevalence of recent PWID (proportion of the population that is recent PWID); $\rho_{ex}$, prevalence of ex-PWID (proportion of the population that is ex-PWID); $\rho_{mig}$, prevalence of migrants from endemic countries (proportion of the population that belongs to this group); $\pi_{rec}$, CHC prevalence among recent PWID; $\pi_{ex}$, CHC prevalence among ex-PWID; $\pi_{mig}$, CHC prevalence among migrants from endemic countries; $\pi_{non}$, CHC prevalence among non-PWID; $\pi$ overall CHC prevalence in Luxembourg; LB, Lower Boundary; UP, Upper Boundary

*Table 6. Results from the method assuming no heterogeneity in the estimation of the CHC prevalence in the general population (primarily non-PWID) from different studies (i.e., similar to a fixed-effect meta-analysis) including migrants applicant for international protection as a separate group.*

| Parameter | Posterior median | Posterior sd | LB (2.5%) | UB (97.5%) |
|---|---|---|---|---|
| $\rho_{rec}$ (%) | 0.17 | 0.02 | 0.13 | 0.2 |
| $\rho_{ex}$ (%) | 0.39 | 0.03 | 0.34 | 0.44 |
| $\rho_{mig}$ (%) | 0.52 | 0 | 0.52 | 0.52 |
| $\pi_{rec}$ (%) | 39.17 | 2.2 | 34.89 | 43.55 |
| $\pi_{ex}$ (%) | 30.48 | 7.3 | 16.18 | 44.84 |
| $\pi_{mig}$ (%) | 0.46 | 0.15 | 0.24 | 0.81 |
| $\pi_{non}$ (%) | 0.07 | 0.04 | 0.02 | 0.16 |
| $\pi$ (%) | 0.26 | 0.06 | 0.16 | 0.38 |
| Number with CHC | 1,259 | 279 | 784 | 1,885 |
| Pr(Recent PWID\|CHC) (%) | 25.45 | 5.8 | 16.71 | 39.39 |
| Pr(Ex-PWID\|CHC) (%) | 46.01 | 7.12 | 31.23 | 59.22 |
| Pr(Mig\|CHC) (%) | 0.95 | 0.3 | 0.49 | 1.65 |
| Pr(Non-PWID\|CHC) (%) | 26.77 | 8.61 | 11.61 | 45.1 |

*Notes*: The number of individuals treated with DAAs is taken into account.

*Abbreviations*: $\rho_{rec}$, prevalence of recent PWID (proportion of the population that is recent PWID); $\rho_{ex}$, prevalence of ex-PWID (proportion of the population that is ex-PWID); $\rho_{mig}$, prevalence of migrants applicant for international protection (proportion of the population that belongs to this group); $\pi_{rec}$, CHC prevalence among recent PWID; $\pi_{ex}$, CHC prevalence among ex-PWID; $\pi_{mig}$, CHC prevalence among migrants applicant for international protection; $\pi_{non}$, CHC prevalence among non-PWID; $\pi$ overall CHC prevalence in Luxembourg; LB, Lower Boundary; UP, Upper Boundary

# APPENDIX

## Fit of the multi-state Markov model

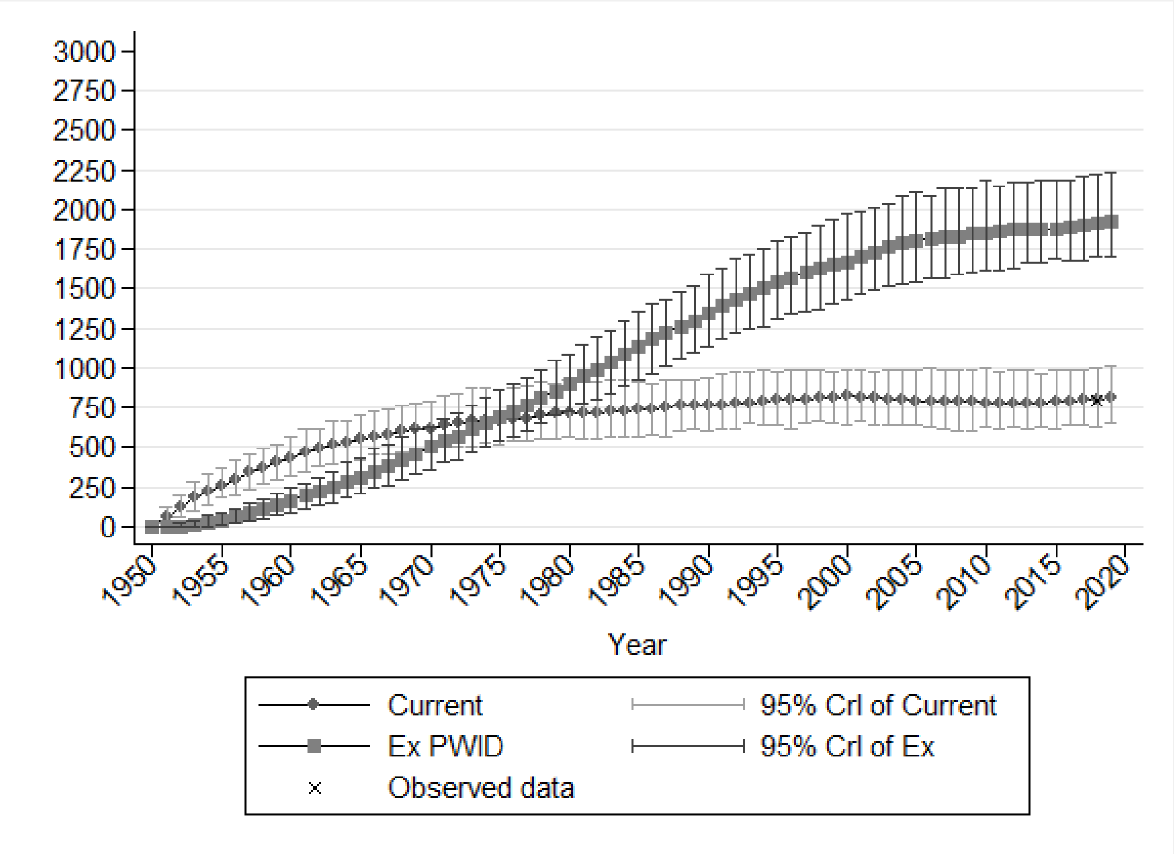


*Figure 2: Model predictions for the numbers of current and ex-PWID (people who inject drugs). The solid black line and shaded grey error bars show the median and 95% credible intervals (95% CrI) for the model projections. For comparison, asterisks indicate the observed country's data.*

## Stan code for Bayesian multiparameter evidence synthesis

```
data {
  int<lower=0> N1579; // Population of 15-79 in `Country`
  int<lower=1> Knon; // Number of studies in the ECDC data for `Country`
  int<lower=1> Kever; // Number of studies in the EMCDDA data for ever
users in `Country`
  int<lower=1> Kcur; // Number of studies in the EMCDDA barometer (for
HCV prevalence of PWID) in `Country`
  int<lower=1> NDAA; // Total number of DAAs
  int<lower=1> NDAA_cur; // Relevant number of DAAs in relation to the
recent PWID study

  real p_cur_mean; // Prior mean for the prevalence of current use in
`Country`
  real<lower=0> p_cur_sd; // Prior sd for the prevalence of current use
in `Country`

  real p_ex_mean; // Prior mean for the prevalence of ex-use in `Country`
  real<lower=0> p_ex_sd; // Prior sd for the prevalence of ex-use in
`Country`

  int<lower=0> Nst_CHC_cur[Kcur]; // Number of individuals in the study
estimating CHC among recent PWID
  int<lower=0> Yst_CHC_cur[Kcur]; // Number of HCV+ in the study
estimating CHC among recent PWID
  //real p_CHC_cur_mean; // Prior mean for the CHC prevalence among
recent PWID in `Country`
  //real<lower=0> p_CHC_cur_sd; // Prior sd for the CHC prevalence among
recent PWID in `Country`

  int<lower=0> Nst_hcv_ever[Kever]; // Number of individuals in the study
estimating anti-HCV among ever IDU
```

```
  int<lower=0> Yst_hcv_ever[Kever]; // Number of HCV+ in the study
estimating anti-HCV among ever PWID

  int<lower=0> Nst_hcv_non[Knon]; // Number of individuals in the study
estimating anti-HCV among non PWID
  int<lower=0> Yst_hcv_non[Knon]; // Number of HCV+ in the study
estimating anti-HCV among non PWID

  vector<lower=0>[3] alpha; // parameter of the Diriclet prior

  real HCVclear_mean; // Prior mean for the HCV clearance probability
  real<lower=0> HCVclear_sd; // Prior sd for the HCV clearance
probability

  real SVR_mean; // Prior mean for the SVR among non-PWID
  real<lower=0> SVR_sd; // Prior sd for the SVR among non-PWID

  real SVR_PWID_mean; // Prior mean for the SVR among PWID
  real<lower=0> SVR_PWID_sd; // Prior sd for the SVR among PWID
}

// Block defining the original parameters
parameters {
  // The parameters to be sampled
  simplex[3] rho; // Prevalence of the three risk groups
  real<lower=0,upper=1> CHCpi_cur; // P(CHC+|Cur)
  real<lower=0,upper=1> CHCpi_ex; // P(CHC+|Ex)
  real<lower=0,upper=1> CHCpi_non; // P(CHC+|Non)
  real<lower=0,upper=1> SVR; // SVR of DAAs among non-PWID
  real<lower=0,upper=1> SVR_PWID; // SVR of DAAs among PWID

  real<lower=0,upper=1-CHCpi_cur> HCVclear; // Probability of HCV
```

```
clearance; upper bound = 1-prevalence of chronic HCV
}


transformed parameters {
  // Change scales
  real<lower=0,upper=1> rho_ever;
  real<lower=0,upper=1> CHCpi_ever;
  real<lower=0,upper=1> pi_ever;
  real<lower=0,upper=1> pi_non;

  rho_ever = rho[1] + rho[2];
  CHCpi_ever = CHCpi_cur*rho[1]/rho_ever + CHCpi_ex*rho[2]/rho_ever;
  pi_ever = CHCpi_ever/(1-HCVclear);
  pi_non = CHCpi_non/(1-HCVclear);
}


// Binomial regression model
model {
  // Priors
  rho ~ dirichlet(alpha);

  //////////////////////////////
  // Likelihood contributions //
  //////////////////////////////

  // Probability of HCV clearance
  HCVclear ~ normal(HCVclear_mean,HCVclear_sd);

  // Prevalence of current use
  rho[1] ~ normal(p_cur_mean,p_cur_sd);
```

```
  // Prevalence of ex-use
  rho[2] ~ normal(p_ex_mean,p_ex_sd);

  // Prevalence of chronic HCV among current users
  Yst_CHC_cur ~ binomial(Nst_CHC_cur,CHCpi_cur);
  //CHCpi_cur ~ normal(p_CHC_cur_mean,p_CHC_cur_sd);

  // Prevalence of HCV among ever users
  Yst_hcv_ever ~ binomial(Nst_hcv_ever,pi_ever);

  // HCV+ among non
  Yst_hcv_non ~ binomial(Nst_hcv_non,pi_non);

  // SVR of DAAs among non-PWID
  SVR ~ normal(SVR_mean,SVR_sd);

  // SVR of DAAs among PWID
  SVR_PWID ~ normal(SVR_PWID_mean,SVR_PWID_sd);
}


generated quantities {
  // Functions of parameters
  real<lower=0,upper=100> overalCHC;
  real logit_CHCpi_cur;
  real logit_CHCpi_ex;
  real logit_CHCpi_non;
  real logit_rho_cur;
  real logit_rho_ex;
  real logit_rho_non;
  real<lower=0,upper=1> pEverGivenCHC;
```

```
  real<lower=0,upper=1> pCurGivenCHC;
  real<lower=0,upper=1> pExGivenCHC;
  real<lower=0,upper=1> pNonGivenCHC;
  real logit_HCVclear;
  real<lower=0> NumberCHC;

  real<lower=0,upper=1> CHCDAApi_non; // P(CHC+|Non) adjusted for DAA
  real<lower=0,upper=1> CHCDAApi_cur; // P(CHC+|Cur) adjusted for DAA
  real<lower=0,upper=1> CHCDAApi_ex; // P(CHC+|Ex) adjusted for DAA
  real<lower=0,upper=1> CHCDAApi_ever; // P(CHC+|Ever) adjusted for DAA

  real<lower=0,upper=100> overalCHC_DAA;
  real pEverGivenCHC_DAA;
  real pCurGivenCHC_DAA;
  real pExGivenCHC_DAA;
  real pNonGivenCHC_DAA;
  real<lower=0> NumberCHC_DAA;

  // Overall HCV prevalence
  overalCHC = 100*(rho[1]*CHCpi_cur + rho[2]*CHCpi_ex +
rho[3]*CHCpi_non);
  pEverGivenCHC = CHCpi_ever*rho_ever/(overalCHC/100);
  pCurGivenCHC = CHCpi_cur*rho[1]/(overalCHC/100);
  pExGivenCHC = CHCpi_ex*rho[2]/(overalCHC/100);
  pNonGivenCHC = CHCpi_non*(1-rho_ever)/(overalCHC/100);
  NumberCHC = round(overalCHC*N1579/100);

  CHCDAApi_non = ( N1579*rho[3]*CHCpi_non -
NDAA*pNonGivenCHC*SVR)/(N1579*rho[3]);
  CHCDAApi_cur = ( N1579*rho[1]*CHCpi_cur -
NDAA_cur*pCurGivenCHC*SVR_PWID_mean)/(N1579*rho[1]);
  CHCDAApi_ex = ( N1579*rho[2]*CHCpi_ex -
```

```
NDAA*pExGivenCHC*SVR_PWID_mean)/(N1579*rho[2]);
  CHCDAApi_ever = CHCDAApi_cur*rho[1]/rho_ever +
CHCDAApi_ex*rho[2]/rho_ever;

  overalCHC_DAA = 100*(rho[1]*CHCDAApi_cur + rho[2]*CHCDAApi_ex +
rho[3]*CHCDAApi_non);
  pEverGivenCHC_DAA = CHCDAApi_ever*rho_ever/(overalCHC_DAA/100);
  pCurGivenCHC_DAA = CHCDAApi_cur*rho[1]/(overalCHC_DAA/100);
  pExGivenCHC_DAA = CHCDAApi_ex*rho[2]/(overalCHC_DAA/100);
  pNonGivenCHC_DAA = CHCDAApi_non*(1-rho_ever)/(overalCHC_DAA/100);
  NumberCHC_DAA = round(overalCHC_DAA*N1579/100);

  logit_rho_cur = logit(rho[1]);
  logit_rho_ex = logit(rho[2]);
  logit_rho_non = logit(rho[3]);
  logit_CHCpi_cur = logit(CHCpi_cur);
  logit_CHCpi_ex = logit(CHCpi_ex);
  logit_CHCpi_non = logit(CHCpi_non);
  logit_HCVclear = logit(HCVclear);
}
```

## Multi-state Markov model

```
#include <stdio.h>
#include <stdlib.h>
#include <string.h>
#include <math.h>
#include <time.h>

#define loops 100
int populationPerYearAndAge[2021][66];
double deathRatePerAge[66];
int totalPopulationPerYear[2021];

FILE *F_DeathRate=fopen("greece-deathRates.txt","r");
FILE *F_Population=fopen("greece-population.txt","r");
FILE *out;

double pa_start = 0.00012;                    // Setting Variable
double pa_step  = 0.000002;                   // Setting Variable
double pa_stop  = 0.000123;                   // Setting Variable

double pa;
double pg = 1.0/11.7;   // Assumed average active injecting career of
11.7 years
double pk = 0.004*12.0;     // Relapse rate of 0.004/month

struct people{
    int age;
    int state;
};

char filename[100];
struct people person[50000000];
```

```
long int totalPersons=0;

void setPopulationAge()
{
    for( int j=0; j<71; j++){
        for( int i=0; i<51; i++){

fscanf(F_Population,"%d",&populationPerYearAndAge[1950+j][15+i]);
        }
    }
}

void setDeathRate()
{
    for( int i=0; i<51; i++){
        fscanf(F_DeathRate,"%lf",&deathRatePerAge[15+i*5]);
        for( int j=0; j<5; j++){
            deathRatePerAge[15+i*5+j] = deathRatePerAge[15+i*5];
        }
    }
}

void getTotalPopulationPerAge()
{
    for( int j=0; j<71; j++){
        for( int i=0; i<51; i++){
            totalPopulationPerYear[1950+j] +=
populationPerYearAndAge[1950+j][15+i];
        }
    }
}
```

```
void printTotalPopulationPerAge()
{
    printf("---- - ----------\n");
    printf("Year - Population\n");
    printf("---- - ----------\n");
    for( int i=1950; i<2021; i++){
        printf("%d - %d\n",i,totalPopulationPerYear[i]);
    }
}

void printDeathRatePerAge()
{
    printf("--- - -----\n");
    printf("Age - Rate\n");
    printf("--- - -----\n");
    for( int i=15; i<65; i++){
        printf("%d - %lf\n",i,deathRatePerAge[i]);
    }
}

void printTotalPersonPerState( int year)
{
    int count[4]= {0,0,0,0};
    for( int i=0; i<totalPersons; i++){
        count[person[i].state]++;
    }

//printf("%d\t%ld\t%ld\t%ld\t%ld\n",year,count[0],count[1],count[2],count
[3]);
    if(year==2014)

printf("%d\t%ld\t%ld\t%ld\t%ld\t%lf\n",year,count[0],count[1],count[2],co
```

```
unt[3],pa);
    if( year>2009){
        fprintf(out,
"%d\t%ld\t%ld\t%ld\t%ld\n",year,count[0],count[1],count[2],count[3]);
    }
}

void initializePopulation()
{
    int cnt=0;
    for( int j=15; j<65; j++){
        for( int i=0; i<populationPerYearAndAge[1950][j]*100; i++){
            person[cnt].age=j;
            person[cnt].state=0;
            cnt++;
        }
    }
    totalPersons = cnt;
}

bool checkRate(double rate)
{
    if (rate <0.001){
        double k = rand()/(1.00+RAND_MAX);
        if(k<0.001){
            rate = rate*1000;
        }
        else{
            return false;
        }
    }
    double k = rand()/(1.00+RAND_MAX);
```

```
    if ( k < rate )
        return true;
    else
        return false;
}

void changeStatusAndAge()
{
    for( int i=0; i<totalPersons; i++){
        // change state
        double k = rand()/(1.00+RAND_MAX);
        if( person[i].state==0){
            if ( checkRate(pa) &&  person[i].age<40){
                person[i].state=1;
            }
        }
        else if( person[i].state==1){
            if ( k < pg ){
                person[i].state=2;
            }
        }
        else if( person[i].state==2){
            if ( k < pk ){
                person[i].state=1;
            }
        }
        // After their 49th year, all active PWID are assumed to cease
injecting
        if(person[i].age>49 && person[i].state==1){
            person[i].state=2;
        }
        // After their 64th year, remove
```

```
        if(person[i].age>64){
            person[i].state=3;
        }
        // death rate depending on age and state
        k = rand()/(1.00+RAND_MAX);
        if( person[i].state==0){
            if ( k < deathRatePerAge[person[i].age] ){
                person[i].state=3;
            }
        }
        else if( person[i].state==1){
            if ( k < deathRatePerAge[person[i].age] * 13.0 ){
                person[i].state=3;
            }
        }
        else if( person[i].state==2){
            if ( k < deathRatePerAge[person[i].age] ){
                person[i].state=3;
            }
        }
        // increase age one year
        person[i].age++;
    }
}

void addNewPersons(int year)
{
    // add new person [15 years old and non PWID]
    int cnt=totalPersons;
    for( int i=0; i<populationPerYearAndAge[year+1][15]*100; i++){
// 100 instead of 1000 for the population to reduce execution time
        person[cnt].age=15;
```

```
        person[cnt].state=0;
        cnt++;
    }
    // increase the total number of population
    totalPersons = cnt;
}
int main()
{
    srand(time(NULL));

    setPopulationAge();
    setDeathRate();

    getTotalPopulationPerAge();
    pa = pa_start;
    while ( pa < pa_stop){

        snprintf(filename, 100, "result_%lf.txt",pa);
        out=fopen(filename,"w");

        for( int iter=0; iter<loops ;iter++){
            initializePopulation();
            for( int year=1950; year<2020; year++){
                printTotalPersonPerState(year);
                changeStatusAndAge();
                addNewPersons(year);
            }
        }

        fclose(out);
        pa = pa + pa_step;
    }
```

```
}
```

## REFERENCES

European Centre for Disease Prevention and Control. 2016. "Epidemiological Assessment of Hepatitis b and c Among Migrants in the EU/EEA." https://www.ecdc.europa.eu/sites/default/files/media/en/publications/Publications/epidemiological-assessment-hepatitis-B-and-C-among-migrants-EU-EEA.pdf.

European Centre for Disease Prevention and Control (ECDC). 2016. "Systematic Review on Hepatitis b and c Prevalence in the EU/EEA." https://www.ecdc.europa.eu/sites/default/files/media/en/publications/Publications/systematic-review-hepatitis-B-C-prevalence.pdf.

———. 2021. "Hepatitis c Prevalence Database." https://www.ecdc.europa.eu/en/all-topics-z/hepatitis-c/tools/hepatitis-c-prevalence-database.

Graf, Christiana, Marcus M Mücke, Georg Dultz, Kai-Henrik Peiffer, Alica Kubesch, Patrick Ingiliz, Stefan Zeuzem, Eva Herrmann, and Johannes Vermehren. 2019. "Efficacy of Direct-acting Antivirals for Chronic Hepatitis C Virus Infection in People Who Inject Drugs or Receive Opioid Substitution Therapy: A Systematic Review and Meta-analysis." *Clinical Infectious Diseases* 70 (11): 2355–65. https://doi.org/10.1093/cid/ciz696.

Grebely, Jason, Sarah Larney, Amy Peacock, Samantha Colledge, Janni Leung, Matthew Hickman, Peter Vickerman, et al. 2019. "Global, Regional, and Country-Level Estimates of Hepatitis c Infection Among People Who Have Recently Injected Drugs." *Addiction* 114 (1): 150–66. https://doi.org/https://doi.org/10.1111/add.14393.

Lampertico, Pietro, Jose A. Carrión, Michael Curry, Juan Turnes, Markus Cornberg, Francesco Negro, Ashley Brown, et al. 2020. "Real-World Effectiveness and Safety of Glecaprevir/Pibrentasvir for the Treatment of Patients with Chronic HCV Infection: A meta-Analysis." *Journal of Hepatology* 72 (6): 1112–21. https://doi.org/10.1016/j.jhep.2020.01.025.

Lin, Lifeng, and Haitao Chu. 2020. "Meta-Analysis of Proportions Using Generalized Linear Mixed Models." *Epidemiology* 31 (5).

https://journals.lww.com/epidem/Fulltext/2020/09000/Meta_analysis_of_Proportions_Using_Generalized.16.aspx.

McDonald, Scott A., Rosmawati Mohamed, Maznah Dahlui, Herlianna Naning, and Adeeba Kamarulzaman. 2014. "Bridging the Data Gaps in the Epidemiology of Hepatitis c Virus Infection in Malaysia Using Multi-Parameter Evidence Synthesis." *BMC Infectious Diseases* 14 (1): 564. https://doi.org/10.1186/s12879-014-0564-6.

Micallef, J. M., J. M. Kaldor, and G. J. Dore. 2006. "Spontaneous Viral Clearance Following Acute Hepatitis c Infection: A Systematic Review of Longitudinal Studies." *Journal of Viral Hepatitis* 13 (1): 34–41. https://doi.org/https://doi.org/10.1111/j.1365-2893.2005.00651.x.

Presanis, Anne M., O. Noel Gill, Timothy R. Chadborn, Caterina Hill, Vivian Hope, Louise Logan, Brian D. Rice, Valerie C. Delpech, A. E. Ades, and Daniela De Angelis. 2010. "Insights into the Rise in HIV Infections, 2001 to 2008: A Bayesian Synthesis of Prevalence Evidence." *AIDS* 24 (18). https://journals.lww.com/aidsonline/Fulltext/2010/11270/Insights_into_the_rise_in_HIV_infections,_2001_to.12.aspx.

Sweeting, M. J., D. De Angelis, M. Hickman, and A. E. Ades. 2008. "Estimating hepatitis C prevalence in England and Wales by synthesizing evidence from multiple data sources. Assessing data conflict and model fit." *Biostatistics* 9 (4): 715–34. https://doi.org/10.1093/biostatistics/kxn004.

Tan, Sarah, Susanna Makela, Daliah Heller, Kevin Konty, Sharon Balter, Tian Zheng, and James H. Stark. 2018. "A Bayesian Evidence Synthesis Approach to Estimate Disease Prevalence in Hard-to-Reach Populations: Hepatitis c in New York City." *Epidemics* 23: 96–109. https://doi.org/https://doi.org/10.1016/j.epidem.2018.01.002.

Thein, Hla-Hla, Qilong Yi, Gregory J. Dore, and Murray D. Krahn. 2008. "Estimation of Stage-Specific Fibrosis Progression Rates in Chronic Hepatitis c Virus Infection: A Meta-Analysis and Meta-Regression." *Hepatology* 48 (2): 418–31. https://doi.org/https://doi.org/10.1002/hep.22375.

Trickey, Adam, Hannah Fraser, Aaron G Lim, Amy Peacock, Samantha Colledge, Josephine G Walker, Janni Leung, et al. 2019. "The Contribution of Injection Drug Use to Hepatitis c Virus Transmission Globally, Regionally, and at Country Level: A Modelling Study." *The Lancet Gastroenterology & Hepatology* 4 (6): 435–44. https://doi.org/https://doi.org/10.1016/S2468-1253(19)30085-8.

Veen, Maaike G. van, Anne M. Presanis, Stefano Conti, Maria Xiridou, Annemarie R. Stengaard, Martin C. Donoghoe, Ard I. van Sighem, Marianne A. van der Sande, and Daniela De Angelis. 2011. "National Estimate of HIV Prevalence in the Netherlands: Comparison and Applicability of Different Estimation Tools." *AIDS* 25 (2). https://journals.lww.com/aidsonline/Fulltext/2011/01140/National_estimate_of_HIV_prevalence_in_the.14.aspx.

# Estimating the prevalence of chronic hepatitis C infection (CHC) in Malta using Bayesian multiparameter evidence synthesis

02/07/2022

## Table of Contents

Introduction ........ 2

Methods ........ 2

Prevalence of ex-PWID ........ 3

Prevalence of recent PWID ........ 3

Prevalence of CHC among recent PWID ........ 4

Prevalence of CHC among ex-PWID ........ 4

Prevalence of CHC among non-PWID ........ 5

Sensitivity analysis including migrants from endemic countries ........ 6

Results ........ 7

Tables and Figures ........ 9

Appendix ........ 16

Fit of the multi-state Markov model ........ 16

Stan code for Bayesian multiparameter evidence synthesis ........ 17

Multi-state Markov model ........ 23

References ........ 31

# INTRODUCTION

Patients with chronic hepatitis C (HCV) infection often remain asymptomatic for decades and even for life, but a proportion of them develop active chronic hepatitis, which is a progressive disease (Thein et al. 2008). Because of the largely asymptomatic nature of HCV infection, notification data reflect national screening and testing practices and do not give accurate insights into the prevalence of HCV infection. Although there are studies that have estimated the prevalence of HCV antibodies (anti-HCV) in specific groups such as the general population, first-time blood donors, or people who inject drugs (PWID) at drug treatment centers, those alone cannot be combined to produce national estimates unless some additional information regarding the composition of each HCV risk group and its prevalence in the population are known (Sweeting et al. 2008; McDonald et al. 2014; Tan et al. 2018).

# METHODS

Bayesian multiparameter evidence synthesis (MPES) has been a popular approach to formally estimate anti-HCV or HIV prevalence (Sweeting et al. 2008; McDonald et al. 2014; Tan et al. 2018; Presanis et al. 2010; Veen et al. 2011). In this project, we extend this method to estimating the prevalence of chronic hepatitis C infection (CHC), defined as a HCV-RNA positive result [i.e., active (viremic) infection is used as a proxy of chronic disease], with the population (15-79 years) split into three main non-overlapping risk groups, i.e., recent PWID (those who have injected in the last year; without including probably those injecting for chemsex), ex-PWID, and non-PWID. A unified model is assumed including parameters associated with the CHC prevalence of recent, ex-PWID, and non-PWID (denoted by $\pi_{rec}$, $\pi_{ex}$, and $\pi_{non}$, respectively) and the prevalence of these risk groups in the population (denoted by $\rho_{rec}$, $\rho_{ex}$, and $\rho_{non}$, respectively; $\rho_{rec} + \rho_{ex} + \rho_{non} = 1$). To estimate the CHC prevalence in the whole population, $\pi$, we used the formula:

$$\pi = \pi_{rec}\rho_{rec} + \pi_{ex}\rho_{ex} + \pi_{non}\rho_{non} \quad (1)$$

Bayesian synthesis considers all available data, allowing for an evidence-based estimation of CHC prevalence in the population, with inherent uncertainty properly accounted for. Our aim is to estimate the CHC prevalence for the total (15-79) population of Malta in 2019 using sources of information over the last decade (2010-2019).

## Prevalence of ex-PWID

Data on the prevalence of ex-PWID (proportion of the population that is ex-PWID) are sparse and generally often unreliable. To overcome this limitation, we apply the method proposed by McDonald et al. (2014), which requires simulating a multi-state Markov model representing the non-PWID, recent PWID, and ex-PWID. In the multi-state Markov model, the simulation starts in 1950 and examines the population aged 15-79 years old. A certain number of 15-year-old individuals enter the simulation process each year. These numbers of 15-year-old individuals are obtained from the United Nations Statistic Division UNSD, being different for each country. The ageing of the population is also taken into account. Country-specific overall mortality data are retrieved from the Life Tables of the World Health Organization WHO. The duration of injecting career is also country-specific and could be obtained from the paper of Hines et al. (2020). However, since no data for Malta are reported in Hines et al. (2020), we used the duration of injecting career from Cyprus.

To estimate the number of ex-PWID, the model can be calibrated on the number of recent PWID provided in the EMCDDA barometer or the EMCDDA statistical bulletin (the code of the Markov model can be found in the Appendix). If there are no available data in the barometer, estimates from the systematic review of Grebely et al. (2019) could be used. Currently, the multi-state Markov model was calibrated on the estimated number of daily opiate users reported in the National Report on the Drug Situation in Malta in 2015, a country feedback document returned to ECDC by the focal point.

After applying the model for Malta, we compute the number (and the corresponding Confidence Interval - CI) of ex-PWID in 2019. The number of ex-PWID is then divided by the population size (15-79 years) in the same year, and a Normal distribution, constrained such that $\rho_{ex} \in (0,1)$, is assumed for $\rho_{ex}$. The standard deviation of this distribution is specified to approximately correspond to the respective CI, i.e. $\left(UB_{\rho_{ex}} - LB_{\rho_{ex}}\right)/(2 \times 1.96)$, where $UB_{\rho_{ex}}$ and $LB_{\rho_{ex}}$ denote the upper and lower limit of the CI.

## Prevalence of recent PWID

The number of recent PWID in Malta in 2019 is also projected by the multi-state Markov model described in the previous subsection. To estimate $\rho_{rec}$, the number of recent PWID is divided

by the population size (15-79 years). Similar to the previous subsection, a Normal distribution, constrained such that $\rho_{rec} \in (0,1)$, is assumed for $\rho_{rec}$, with the standard deviation specified to correspond to the CI obtained from the multi-state Markov model.

## Prevalence of CHC among recent PWID

The prevalence of CHC among recent PWID was informed by CHC prevalence data reported in the paper of Grebely et al. (2019) in 2011-14. However, some people may have been treated with direct-acting antivirals (DAAs), with the sustained virologic response ($SVR_{PWID}$) among PWID estimated to be 88% (95% CI: 80% to 93%) (Graf et al. 2019). Based on the information provided by the national focal point, the number of individuals treated with DAAs up to 2019 is approximately equal to 265. However, the proportions of each risk group among those treated with DAAs, i.e. $\rho_{rec|DAA}$, $\rho_{ex|DAA}$, and $\rho_{non|DAA}$ ($\rho_{rec|DAA} + \rho_{ex|DAA} + \rho_{non|DAA} = 1$), are not currently available in Malta. In this report, we make the assumption that $\rho_{rec|DAA}$ is equal to the proportion of recent PWID among CHC-positive individuals, i.e. Pr(Recent PWID|CHC), as estimated by our model when the DAA uptake is ignored (Table 2). Similarly, we assume that $\rho_{ex|DAA} = \text{Pr(Ex-PWID|CHC)}$ and $\rho_{non|DAA} = \text{Pr(Non-PWID|CHC)}$. Thus, the CHC prevalence among recent PWID, adjusted for DAAs, can be estimated by

$$\pi_{rec} = \frac{N_{15,79}\rho_{rec}\tilde{\pi}_{rec} - N_{DAA}\rho_{rec|DAA}SVR_{PWID}}{N_{15,79}\rho_{rec}}, \quad (2)$$

where $\tilde{\pi}_{rec}$ denotes the CHC estimate derived solely from the data reported in Grebely et al. (2019).

## Prevalence of CHC among ex-PWID

Information on CHC prevalence among ex-PWID (i.e., $\pi_{ex}$) is difficult to obtain directly. To overcome this, national anti-HCV prevalence data from ever PWID obtained after personal communication with the national focal point were used. However, as these data refer to the anti-HCV prevalence, they are adjusted similarly to the procedure described in the previous subsection, i.e.

$$\pi_{ever} = \frac{N_{15,79}\rho_{ever}\pi(\text{anti-HCV})_{ever}(1-\rho_{clear}) - N_{DAA}\rho_{ever|DAA}SVR_{PWID}}{N_{15,79}\rho_{ever}}. \quad (3)$$

where $\pi(\text{anti-HCV})_{ever}$ denotes the anti-HCV prevalence among ever PWID, $\rho_{ever} = \rho_{rec} + \rho_{ex}$, $\rho_{clear}$ denotes the spontaneous viral clearance [assumed to be equal to 0.26 (95% CI 0.22–0.29); (Micallef, Kaldor, and Dore 2006)], and $\rho_{ever|DAA}$ the proportion of ever PWID among individuals treated with DAAs. Recall that it is assumed that $\rho_{ever|DAA}$ is assumed to be equal to Pr(Ever PWID|CHC), as estimated by our model ignoring the effect of DAAs.

Then, since an estimate of $\pi_{ever}$ is available, $\pi_{ex}$ can be indirectly estimated since $\pi_{ever}$ is equal to a weighted average of $\pi_{rec}$ and $\pi_{ex}$, as shown by the following formula

$$\pi_{ever} = \frac{\rho_{rec}}{\rho_{ever}}\pi_{rec} + \frac{\rho_{ex}}{\rho_{ever}}\pi_{ex}, \quad (4)$$

or, equivalently,

$$\pi_{ex} = \left(\pi_{ever} - \frac{\rho_{rec}}{\rho_{ever}}\pi_{rec}\right) \times \frac{\rho_{ever}}{\rho_{ex}}. \quad (5)$$

## Prevalence of CHC among non-PWID

To estimate the CHC prevalence among non-PWID in a country (after 2010), the ECDC database (European Centre for Disease Prevention and Control (ECDC) 2021) was used. The ECDC group has carefully and critically collected information from relevant studies across the EU/EEA region in cooperation with the focal points of each country. On the basis of the representativeness of each study and other factors (European Centre for Disease Prevention and Control (ECDC) 2016), a risk of bias score was assigned to each study, with higher values indicating higher-quality studies. When higher-quality studies are available in a country (≥ 4), they are used to estimate the overall CHC prevalence in that country among non-PWID. If there are no higher-quality studies, general population estimates with a lower quality (<4) are pooled. If no general population prevalence estimates are available, data on the prevalence of HCV in pregnant women could be used as a prevalence measure. Finally, if data on pregnant women are lacking too, first-time blood donor studies could be an option. However, if the national focal point agrees or recommends, estimates from a neighboring country could be used instead, or we could use any other relevant information provided/suggested by the national focal point.

We directly use CHC prevalence data from studies in the general population, if available. If the data in the general population refer to anti-HCV prevalence (without any data on the viremic population), we adjust the estimates to get the CHC prevalence based on the spontaneous HCV clearance estimate of 26% and the number of individuals treated with DAAs in the general population, as previously described, i.e.

$$\pi_{non} = \frac{N_{15,79}\rho_{non}\pi(\text{anti-HCV})_{non}(1-\rho_{clear}) - N_{DAA}\rho_{non|DAA}SVR}{N_{15,79}\rho_{non}}. \quad (6)$$

where $\pi(\text{anti-HCV})_{non}$ denotes the anti-HCV prevalence among non-PWID and $SVR$ is the sustained virologic response of DAAs in the general population estimated to be 96.7% (95% CI: 95.4% to 98.1%) (Lampertico et al. 2020).

As $\rho_{rec}$ and $\rho_{ex}$ are likely to be low, $\pi_{non}$ will be the most critical factor for the estimation of the overall CHC prevalence in the population. To adjust for potential heterogeneity between the CHC prevalence estimates from different studies in the non-PWID population in a country, as a sensitivity analysis, we also apply a random-effect meta-analytic approach to pool the CHC prevalence across studies on non-PWID (Lin and Chu 2020). The random-effect approach is carried out only when at least 3 studies are available in the general population of a certain country, and the estimates are compared with the corresponding estimates from the approach assuming no heterogeneity (similar to a fixed-effect meta-analysis). If the estimates are inconsistent, possible explanations are investigated and discussed. If there is substantial heterogeneity between studies, it would help more to explore the causes; for example, following national focal point suggestions, some studies could be excluded to reduce heterogeneity.

## Sensitivity analysis including migrants from endemic countries

A sensitivity analysis including data for the viremic population among migrants based on the report of the ECDC group (European Centre for Disease Prevention and Control 2016) was carried out. Migrants from endemic countries represent 5% of the adult population in Malta (Table 8 in European Centre for Disease Prevention and Control (2016)), with the respective CHC prevalence being equal to $\pi_{mig} = 1.7\%$ (Table 9 in European Centre for Disease Prevention and Control (2016)). The overall CHC prevalence is now estimated by

$$\pi = \pi_{rec}\rho_{rec} + \pi_{ex}\rho_{ex} + \pi_{mig}\rho_{mig} + \pi_{non}\rho_{non},$$

where $\rho_{rec} + \rho_{ex} + \rho_{mig} + \rho_{non} = 1$. A treatment adjustment similar to that described in the previous subsections was also performed.

## RESULTS

The above-mentioned approach was used to estimate the prevalence of CHC in 2019 (considering ages between 15-79 years: 405,209).

The aggregated data used by our approach are briefly presented in Table 1. The multi-state Markov model was calibrated on the estimated number of daily opiate users reported in the National Report on the Drug Situation in Malta in 2015, a country feedback document returned to ECDC by the focal point.

In Malta, there were 5 studies on first-time blood donors, which included only anti-HCV data. To estimate the CHC prevalence in the general population (primarily non-PWID), we used a fixed-effect meta-analytic approach, with the corresponding results, when information on DAAs is ignored, presented in Table 2. However, based on data provided by the focal point, approximately 265 individuals were treated with DAAs from June 2018 to December 2019 in Malta, with the proportion of recent PWID, ex-PWID, and non-PWID among the 265 treated individuals assumed to be equal to Pr(Recent PWID|CHC) ≈ 22.88% Pr(Ex-PWID|CHC) ≈ 63.94% and Pr(Non-PWID|CHC) ≈ 12.6%, respectively (Table 2).

The corresponding results accounting for the DAA uptake are presented in Table 3. The prevalence of recent and ex-PWID was low in Malta (about 0.4% and 1.01%, respectively) corresponding to 1,610 (95% CI: 1,370-1,860) recent PWID and 4,100 (95% CI: 3,750-4,540) ex-PWID in the population. However, the CHC prevalence in these groups was substantial (Table 3) being 15.5% and 16.91%, respectively. This translates to 249 (95% CI: 128-382) and 691 (95% CI: 445-974) recent and ex-PWID aged 15-79 living with CHC infection in Malta in 2019. The CHC prevalence in the general population was 0.03% (95% CI: 0.01%-0.07%), much lower than that of the high-risk groups. Taking all pieces of information into account, the overall CHC prevalence in Malta in 2019 was equal to 0.27% (95% CI: 0.2%-0.35%), which corresponds to 1,083 (95% CI: 812-1,398) individuals aged 15-79 years with CHC infection. The corresponding results under a random-effect meta-analysis for the studies in the general population were very similar and are provided in Table 4.

The results from our model including migrants from endemic countries as a separate group are presented in Table 5. However, this analysis comes with possible limitations; that is, including migrants as a separate group is valid only if migrants do not overlap with the remaining groups (recent PWID, ex-PWID, and non-PWID). Thus, if migrants do participate proportionally in the study(ies) in the general population, this analysis may result in biased overall CHC estimates (most probably in higher CHC prevalence estimates).

# TABLES AND FIGURES

*Table 1. Data (real) contributing to the estimation of CHC prevalence in Malta in 2019.*

| Parameter | Estimate (95%CI) | Number (95%CI) | Numerator | Denominator | Notes | Year of study |
|---|---|---|---|---|---|---|
| $\rho_{rec}$ | | 1,610 (1,370-1,860) | | | Method based on McDonald et al. | 2019 |
| $\rho_{ex}$ | | 4,100 (3,750-4,540) | | | Method based on McDonald et al. | 2019 |
| $\pi_{rec}$ | 18.9% (10.4%-28.4%) | | | | Grebely et al. | 2011-14 |
| $\pi(\text{anti-HCV})_{ever}$ | | | 67 | 248 | Country feedback (FSWS - Head Office) | 2019 |
| $\pi(\text{anti-HCV})_{non}$ | | | 0 | 1,755 | ECDC database (NA et al.); Risk of bias=NA | 2010 |
| $\pi(\text{anti-HCV})_{non}$ | | | 1 | 2,300 | ECDC database (NA et al.); | 2011 |

| Parameter | Estimate (95%CI) | Number (95%CI) | Numerator | Denominator | Notes | Year of study |
|---|---|---|---|---|---|---|
| | | | | | Risk of bias=NA | |
| $\pi(\text{anti-HCV})_{non}$ | | | 2 | 1,842 | ECDC database (NA et al.); Risk of bias=NA | 2014 |
| $\pi(\text{anti-HCV})_{non}$ | | | 1 | 2,163 | ECDC database (NA et al.); Risk of bias=NA | 2015 |
| $\pi(\text{anti-HCV})_{non}$ | | | 1 | 2,040 | ECDC database (NA et al.); Risk of bias=NA | 2016 |

*Notes*: Although it looks counter-intuitive, **higher risk of bias score denotes a higher-quality study** (range from 0 to 6)

*Table 2. Results from the method assuming no heterogeneity in the estimation of the CHC prevalence in the general population from different studies (i.e., similar to a fixed-effect meta-analysis).*

| Parameter | Posterior median | Posterior sd | LB (2.5%) | UB (97.5%) |
|---|---|---|---|---|
| $\rho_{rec}$ (%) | 0.4 | 0.03 | 0.34 | 0.46 |
| $\rho_{ex}$ (%) | 1.01 | 0.05 | 0.91 | 1.11 |
| $\pi_{rec}$ (%) | 18.88 | 4.55 | 9.96 | 27.72 |
| $\pi_{ex}$ (%) | 20.65 | 3.46 | 14.03 | 27.56 |
| $\pi_{non}$ (%) | 0.04 | 0.02 | 0.02 | 0.09 |
| $\pi$ (%) | 0.33 | 0.04 | 0.26 | 0.4 |
| Number with CHC | 1,320 | 150 | 1,051 | 1,638 |
| Pr(Recent PWID\|CHC) (%) | 22.88 | 6.37 | 11.64 | 36.44 |
| Pr(Ex-PWID\|CHC) (%) | 63.94 | 7.5 | 48.45 | 77.64 |
| Pr(Non-PWID\|CHC) (%) | 12.6 | 4.72 | 5.2 | 23.44 |

*Notes*: Information on DAAs is not taken into account.

*Abbreviations*: $\rho_{rec}$, prevalence of recent PWID (proportion of the population that is recent PWID); $\rho_{ex}$, prevalence of ex-PWID (proportion of the population that is ex-PWID); $\pi_{rec}$, CHC prevalence among recent PWID; $\pi_{ex}$, CHC prevalence among ex-PWID; $\pi_{non}$, CHC prevalence among non-PWID; $\pi$ overall CHC prevalence in Malta; LB, Lower Boundary; UP, Upper Boundary

*Table 3. Results from the method assuming no heterogeneity in the estimation of the CHC prevalence in the general population from different studies (i.e., similar to a fixed-effect meta-analysis).*

| Parameter | Posterior median | Posterior sd | LB (2.5%) | UB (97.5%) |
|---|---|---|---|---|
| $\rho_{rec}$ (%) | 0.4 | 0.03 | 0.34 | 0.46 |
| $\rho_{ex}$ (%) | 1.01 | 0.05 | 0.91 | 1.11 |
| $\pi_{rec}$ (%) | 15.5 | 3.79 | 8.13 | 22.98 |
| $\pi_{ex}$ (%) | 16.91 | 3.16 | 11.1 | 23.41 |
| $\pi_{non}$ (%) | 0.03 | 0.01 | 0.01 | 0.07 |
| $\pi$ (%) | 0.27 | 0.04 | 0.2 | 0.35 |
| Number with CHC | 1,083 | 150 | 812 | 1,398 |
| Pr(Recent PWID\|CHC) (%) | 22.97 | 6.44 | 11.59 | 36.88 |
| Pr(Ex-PWID\|CHC) (%) | 63.91 | 7.48 | 48.34 | 77.78 |
| Pr(Non-PWID\|CHC) (%) | 12.44 | 4.65 | 5.22 | 23.15 |

*Notes*: The number of individuals treated with DAAs is taken into account.

*Abbreviations*: $\rho_{rec}$, prevalence of recent PWID (proportion of the population that is recent PWID); $\rho_{ex}$, prevalence of ex-PWID (proportion of the population that is ex-PWID); $\pi_{rec}$, CHC prevalence among recent PWID; $\pi_{ex}$, CHC prevalence among ex-PWID; $\pi_{non}$, CHC prevalence among non-PWID; $\pi$ overall CHC prevalence in Malta; LB, Lower Boundary; UP, Upper Boundary

*Table 4. Results from the method assuming heterogeneity in the estimation of the CHC prevalence in the general population from different studies (i.e., similar to a random-effect meta-analysis).*

| Parameter | Posterior median | Posterior sd | LB (2.5%) | UB (97.5%) |
|---|---|---|---|---|
| $\rho_{rec}$ (%) | 0.4 | 0.03 | 0.34 | 0.46 |
| $\rho_{ex}$ (%) | 1.01 | 0.05 | 0.91 | 1.11 |
| $\pi_{rec}$ (%) | 15.35 | 3.78 | 8.1 | 22.89 |
| $\pi_{ex}$ (%) | 16.78 | 3.22 | 10.79 | 23.47 |
| $\pi_{non}$ (%) | 0.02 | 0.03 | 0 | 0.08 |
| $\pi$ (%) | 0.25 | 0.05 | 0.19 | 0.35 |
| Number with CHC | 1,032 | 185 | 755 | 1,412 |
| Pr(Recent PWID\|CHC) (%) | 23.8 | 6.85 | 11.92 | 38.63 |
| Pr(Ex-PWID\|CHC) (%) | 66.59 | 8.41 | 48.31 | 81.14 |
| Pr(Non-PWID\|CHC) (%) | 8.45 | 6.53 | 1.47 | 25.54 |
| Between-study variance | 1 | 1.99 | 0.07 | 7.1 |

*Notes*: The number of individuals treated with DAAs is taken into account.

*Abbreviations*: $\rho_{rec}$, prevalence of recent PWID (proportion of the population that is recent PWID); $\rho_{ex}$, prevalence of ex-PWID (proportion of the population that is ex-PWID); $\pi_{rec}$, CHC prevalence among recent PWID; $\pi_{ex}$, CHC prevalence among ex-PWID; $\pi_{non}$, CHC prevalence among non-PWID; $\pi$ overall CHC prevalence in Malta; LB, Lower Boundary; UP, Upper Boundary

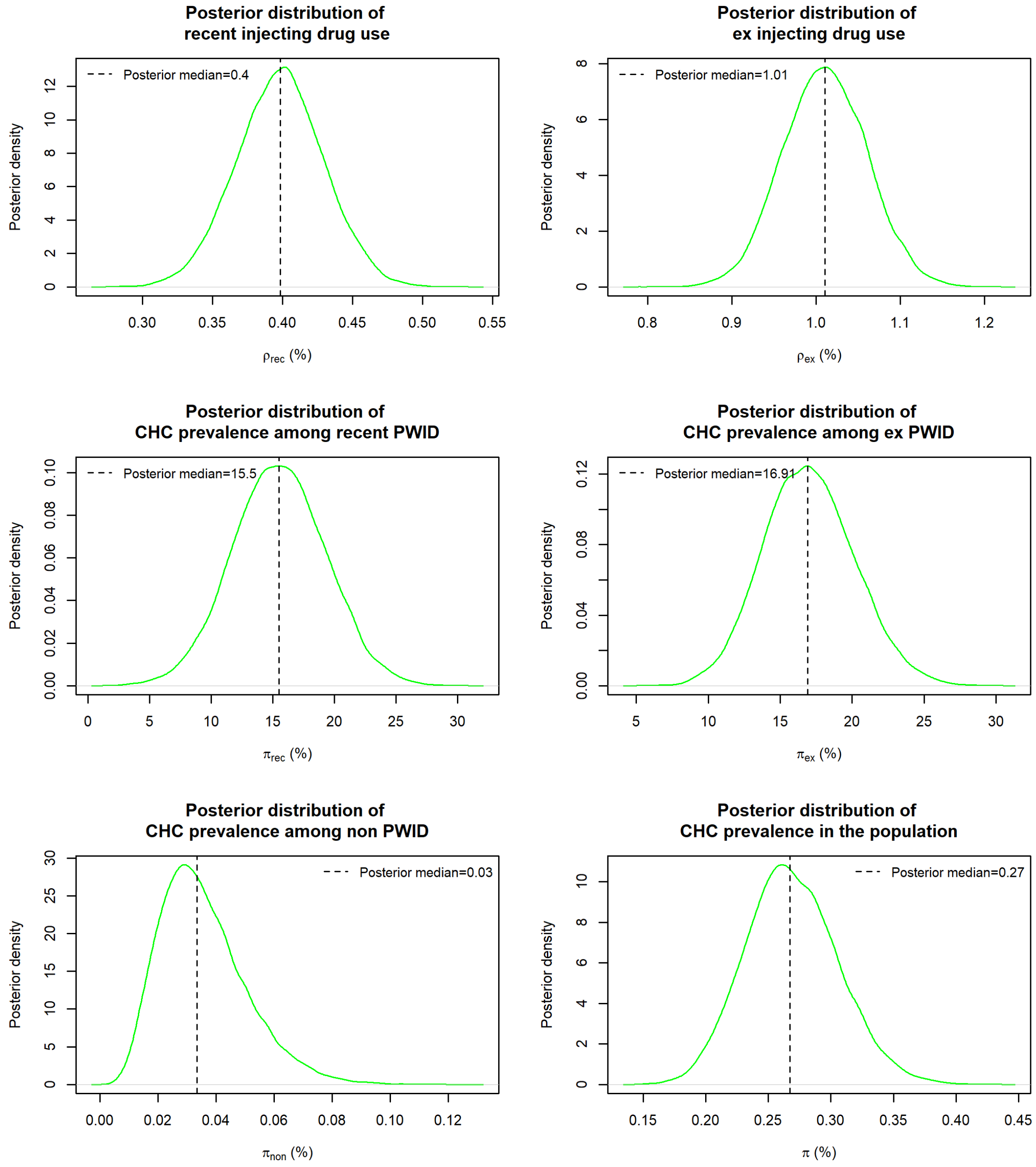


*Figure 1. Posterior distributions of the key model parameters using the approach that assumes no heterogeneity in the estimation of the CHC prevalence in the general population (primarily non-PWID) from different studies (i.e., similar to a fixed-effect meta-analysis), with the number of individuals treated with DAA taken into account.*

*Table 5. Results from the method assuming no heterogeneity in the estimation of the CHC prevalence in the general population (primarily non-PWID) from different studies (i.e., similar to a fixed-effect meta-analysis) including migrants from endemic countries as a separate group.*

| Parameter | Posterior median | Posterior sd | LB (2.5%) | UB (97.5%) |
|---|---|---|---|---|
| $\rho_{rec}$ (%) | 0.4 | 0.03 | 0.34 | 0.46 |
| $\rho_{ex}$ (%) | 1.01 | 0.05 | 0.91 | 1.11 |
| $\rho_{mig}$ (%) | 5 | 0 | 5 | 5 |
| $\pi_{rec}$ (%) | 16.23 | 3.94 | 8.58 | 24.02 |
| $\pi_{ex}$ (%) | 17.63 | 3.19 | 11.7 | 24.17 |
| $\pi_{mig}$ (%) | 1.43 | 0.32 | 0.81 | 2.08 |
| $\pi_{non}$ (%) | 0.04 | 0.02 | 0.01 | 0.07 |
| $\pi$ (%) | 0.35 | 0.04 | 0.27 | 0.43 |
| Number with CHC | 1,415 | 165 | 1,113 | 1,757 |
| Pr(Recent PWID\|CHC) (%) | 18.4 | 5.07 | 9.41 | 29.31 |
| Pr(Ex-PWID\|CHC) (%) | 51.16 | 6.7 | 37.51 | 63.77 |
| Pr(Mig\|CHC) (%) | 20.54 | 4 | 12.6 | 28.38 |
| Pr(Non-PWID\|CHC) (%) | 9.47 | 3.69 | 3.87 | 18.31 |

*Notes*: The number of individuals treated with DAAs is taken into account.

*Abbreviations*: $\rho_{rec}$, prevalence of recent PWID (proportion of the population that is recent PWID); $\rho_{ex}$, prevalence of ex-PWID (proportion of the population that is ex-PWID); $\rho_{mig}$, prevalence of migrants from endemic countries (proportion of the population that belongs to this group); $\pi_{rec}$, CHC prevalence among recent PWID; $\pi_{ex}$, CHC prevalence among ex-PWID; $\pi_{mig}$, CHC prevalence among migrants from endemic countries; $\pi_{non}$, CHC prevalence among non-PWID; $\pi$ overall CHC prevalence in Malta; LB, Lower Boundary; UP, Upper Boundary

# APPENDIX

## Fit of the multi-state Markov model

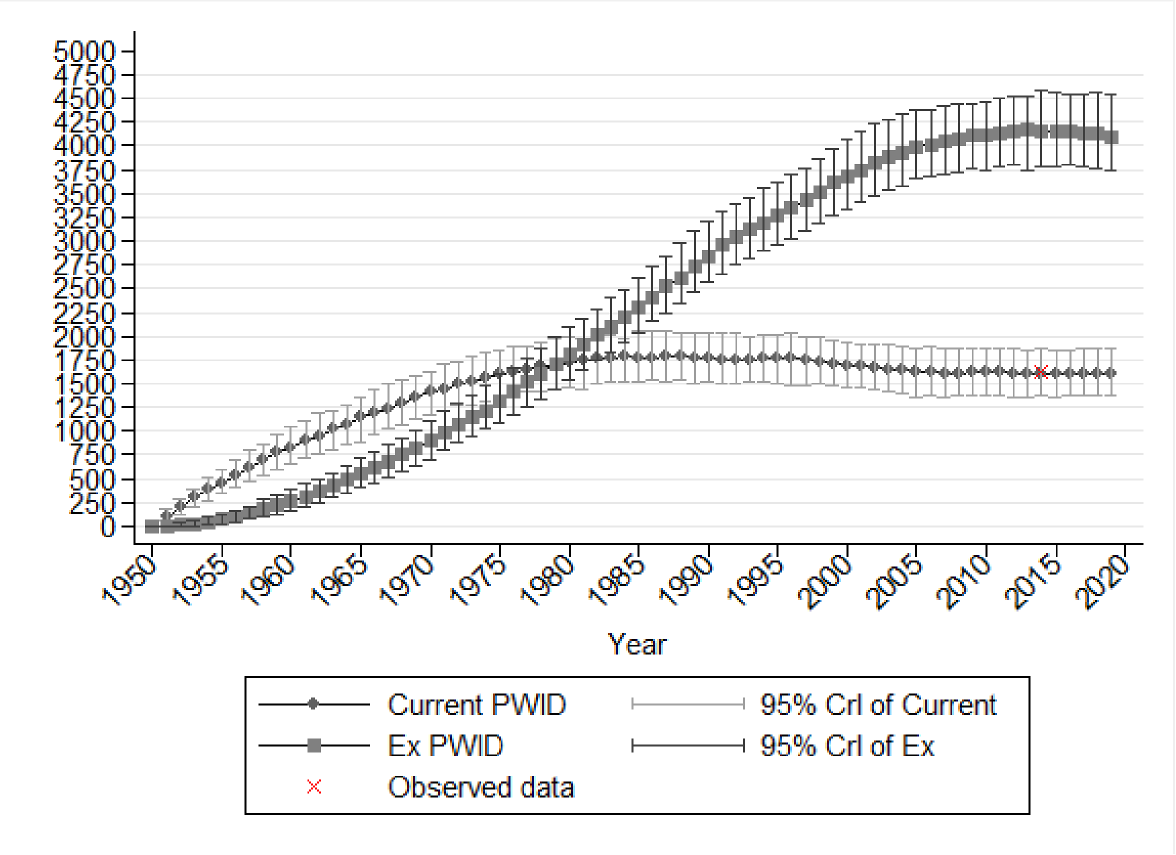


*Figure 2: Model predictions for the numbers of current and ex-PWID (people who inject drugs). The solid black line and shaded grey error bars show the median and 95% credible intervals (95% CrI) for the model projections. For comparison, asterisks indicate the observed country's data.*

## Stan code for Bayesian multiparameter evidence synthesis

```
data {
  int<lower=0> N1579; // Population of 15-79 in `Country`
  int<lower=1> Knon; // Number of studies in the ECDC data for `Country`
  int<lower=1> Kever; // Number of studies in the EMCDDA data for ever users in `Country`
  int<lower=1> Kcur; // Number of studies in the EMCDDA barometer (for HCV prevalence of PWID) in `Country`
  int<lower=1> NDAA; // Total number of DAAs from 2015 to 2019

  real p_cur_mean; // Prior mean for the prevalence of current use in `Country`
  real<lower=0> p_cur_sd; // Prior sd for the prevalence of current use in `Country`

  real p_ex_mean; // Prior mean for the prevalence of ex-use in `Country`
  real<lower=0> p_ex_sd; // Prior sd for the prevalence of ex-use in `Country`

  //int<lower=0> Nst_hcv_cur[Kcur]; // Number of individuals in the study estimating anti-HCV among recent PWID
  //int<lower=0> Yst_hcv_cur[Kcur]; // Number of HCV+ in the study estimating anti-HCV among recent PWID
  real p_CHC_cur_mean; // Prior mean for the CHC prevalence among recent PWID in `Country`
  real<lower=0> p_CHC_cur_sd; // Prior sd for the CHC prevalence among recent PWID in `Country`

  int<lower=0> Nst_hcv_ever[Kever]; // Number of individuals in the study estimating anti-HCV among ever IDU
  int<lower=0> Yst_hcv_ever[Kever]; // Number of HCV+ in the study estimating anti-HCV among ever PWID
```

```
  int<lower=0> Nst_hcv_non[Knon]; // Number of individuals in the study estimating anti-HCV among non PWID
  int<lower=0> Yst_hcv_non[Knon]; // Number of HCV+ in the study estimating anti-HCV among non PWID

  vector<lower=0>[3] alpha; // parameter of the Diriclet prior

  real HCVclear_mean; // Prior mean for the HCV clearance probability
  real<lower=0> HCVclear_sd; // Prior sd for the HCV clearance probability

  real SVR_mean; // Prior mean for the SVR among non-PWID
  real<lower=0> SVR_sd; // Prior sd for the SVR among non-PWID

  real SVR_PWID_mean; // Prior mean for the SVR among PWID
  real<lower=0> SVR_PWID_sd; // Prior sd for the SVR among PWID
}

// Block defining the original parameters
parameters {
  // The parameters to be sampled
  simplex[3] rho; // Prevalence of the three risk groups
  real<lower=0,upper=1> CHCpi_cur; // P(CHC+|Cur)
  real<lower=0,upper=1> CHCpi_ex; // P(CHC+|Ex)
  real<lower=0,upper=1> CHCpi_non; // P(CHC+|Non)
  real<lower=0,upper=1> SVR; // SVR of DAAs among non-PWID
  real<lower=0,upper=1> SVR_PWID; // SVR of DAAs among PWID

  real<lower=0,upper=1-CHCpi_cur> HCVclear; // Probability of HCV clearance; upper bound = 1-prevalence of chronic HCV
}
```

```
transformed parameters {
  // Change scales
  real<lower=0,upper=1> rho_ever;
  real<lower=0,upper=1> CHCpi_ever;
  real<lower=0,upper=1> pi_ever;
  real<lower=0,upper=1> pi_cur;
  real<lower=0,upper=1> pi_non;

  rho_ever = rho[1] + rho[2];
  CHCpi_ever = CHCpi_cur*rho[1]/rho_ever + CHCpi_ex*rho[2]/rho_ever;
  pi_ever = CHCpi_ever/(1-HCVclear);
  pi_cur = CHCpi_cur/(1-HCVclear);
  pi_non = CHCpi_non/(1-HCVclear);
}


// Binomial regression model
model {
  // Priors
  rho ~ dirichlet(alpha);

  //////////////////////////////
  // Likelihood contributions //
  //////////////////////////////

  // Probability of HCV clearance
  HCVclear ~ normal(HCVclear_mean,HCVclear_sd);

  // Prevalence of current use
  rho[1] ~ normal(p_cur_mean,p_cur_sd);
```

```
  // Prevalence of ex-use
  rho[2] ~ normal(p_ex_mean,p_ex_sd);

  // Prevalence of chronic HCV among current users
  //Yst_hcv_cur ~ binomial(Nst_hcv_cur,pi_cur);
  CHCpi_cur ~ normal(p_CHC_cur_mean,p_CHC_cur_sd);

  // Prevalence of HCV among ever users
  Yst_hcv_ever ~ binomial(Nst_hcv_ever,pi_ever);

  // HCV+ among non
  Yst_hcv_non ~ binomial(Nst_hcv_non,pi_non);

  // SVR of DAAs among non-PWID
  SVR ~ normal(SVR_mean,SVR_sd);

  // SVR of DAAs among PWID
  SVR_PWID ~ normal(SVR_PWID_mean,SVR_PWID_sd);
}


generated quantities {
  // Functions of parameters
  real<lower=0,upper=100> overalCHC;
  real logit_CHCpi_cur;
  real logit_CHCpi_ex;
  real logit_CHCpi_non;
  real logit_rho_cur;
  real logit_rho_ex;
  real logit_rho_non;
  real<lower=0,upper=1> pEverGivenCHC;
```

```
  real<lower=0,upper=1> pCurGivenCHC;
  real<lower=0,upper=1> pExGivenCHC;
  real<lower=0,upper=1> pNonGivenCHC;
  real logit_HCVclear;
  real<lower=0> NumberCHC;

  real<lower=0,upper=1> CHCDAApi_non; // P(CHC+|Non) adjusted for DAA
  real<lower=0,upper=1> CHCDAApi_cur; // P(CHC+|Cur) adjusted for DAA
  real<lower=0,upper=1> CHCDAApi_ex; // P(CHC+|Ex) adjusted for DAA
  real<lower=0,upper=1> CHCDAApi_ever; // P(CHC+|Ever) adjusted for DAA

  real<lower=0,upper=100> overalCHC_DAA;
  real pEverGivenCHC_DAA;
  real pCurGivenCHC_DAA;
  real pExGivenCHC_DAA;
  real pNonGivenCHC_DAA;
  real<lower=0> NumberCHC_DAA;

  // Overall HCV prevalence
  overalCHC = 100*(rho[1]*CHCpi_cur + rho[2]*CHCpi_ex +  rho[3]*CHCpi_non
);
  pEverGivenCHC = CHCpi_ever*rho_ever/(overalCHC/100);
  pCurGivenCHC = CHCpi_cur*rho[1]/(overalCHC/100);
  pExGivenCHC = CHCpi_ex*rho[2]/(overalCHC/100);
  pNonGivenCHC = CHCpi_non*(1-rho_ever)/(overalCHC/100);
  NumberCHC = round(overalCHC*N1579/100);

  CHCDAApi_non = ( N1579*rho[3]*CHCpi_non - NDAA*pNonGivenCHC*SVR)/(N1579
*rho[3]);
  CHCDAApi_cur = ( N1579*rho[1]*CHCpi_cur - NDAA*pCurGivenCHC*SVR_PWID_me
an)/(N1579*rho[1]);
  CHCDAApi_ex = ( N1579*rho[2]*CHCpi_ex - NDAA*pExGivenCHC*SVR_PWID_mean)
```

```
/(N1579*rho[2]);
  CHCDAApi_ever = CHCDAApi_cur*rho[1]/rho_ever + CHCDAApi_ex*rho[2]/rho_e
ver;

  overalCHC_DAA = 100*(rho[1]*CHCDAApi_cur + rho[2]*CHCDAApi_ex +  rho[3]
*CHCDAApi_non);
  pEverGivenCHC_DAA = CHCDAApi_ever*rho_ever/(overalCHC_DAA/100);
  pCurGivenCHC_DAA = CHCDAApi_cur*rho[1]/(overalCHC_DAA/100);
  pExGivenCHC_DAA = CHCDAApi_ex*rho[2]/(overalCHC_DAA/100);
  pNonGivenCHC_DAA = CHCDAApi_non*(1-rho_ever)/(overalCHC_DAA/100);
  NumberCHC_DAA = round(overalCHC_DAA*N1579/100);

  logit_rho_cur = logit(rho[1]);
  logit_rho_ex = logit(rho[2]);
  logit_rho_non = logit(rho[3]);
  logit_CHCpi_cur = logit(CHCpi_cur);
  logit_CHCpi_ex = logit(CHCpi_ex);
  logit_CHCpi_non = logit(CHCpi_non);
  logit_HCVclear = logit(HCVclear);
}
```

## Multi-state Markov model

```
#include <stdio.h>
#include <stdlib.h>
#include <string.h>
#include <math.h>
#include <time.h>

#define loops 100
int populationPerYearAndAge[2021][66];
double deathRatePerAge[66];
int totalPopulationPerYear[2021];

FILE *F_DeathRate=fopen("`Country`-deathRates.txt","r");
FILE *F_Population=fopen("`Country`-population.txt","r");
FILE *out;

double pa_start = 0.00012;                    // Setting Variable
double pa_step  = 0.000002;                   // Setting Variable
double pa_stop  = 0.000123;                   // Setting Variable

double pa;
double pg = 1.0/11.7;   // Assumed average active injecting career of  11
.7 years
double pk = 0.004*12.0;     // Relapse rate of 0.004/month

struct people{
    int age;
    int state;
};

char filename[100];
struct people person[50000000];
```

```
long int totalPersons=0;

void setPopulationAge()
{
    for( int j=0; j<71; j++){
        for( int i=0; i<51; i++){
            fscanf(F_Population,"%d",&populationPerYearAndAge[1950+j][15+
i]);
        }
    }
}

void setDeathRate()
{
    for( int i=0; i<51; i++){
        fscanf(F_DeathRate,"%lf",&deathRatePerAge[15+i*5]);
        for( int j=0; j<5; j++){
            deathRatePerAge[15+i*5+j] = deathRatePerAge[15+i*5];
        }
    }
}

void getTotalPopulationPerAge()
{
    for( int j=0; j<71; j++){
        for( int i=0; i<51; i++){
            totalPopulationPerYear[1950+j] += populationPerYearAndAge[195
0+j][15+i];
        }
    }
}
```

```
void printTotalPopulationPerAge()
{
    printf("---- - ----------\n");
    printf("Year - Population\n");
    printf("---- - ----------\n");
    for( int i=1950; i<2021; i++){
        printf("%d - %d\n",i,totalPopulationPerYear[i]);
    }
}

void printDeathRatePerAge()
{
    printf("--- - -----\n");
    printf("Age - Rate\n");
    printf("--- - -----\n");
    for( int i=15; i<65; i++){
        printf("%d - %lf\n",i,deathRatePerAge[i]);
    }
}

void printTotalPersonPerState( int year)
{
    int count[4]= {0,0,0,0};
    for( int i=0; i<totalPersons; i++){
        count[person[i].state]++;
    }
    //printf("%d\t%ld\t%ld\t%ld\t%ld\n",year,count[0],count[1],count[2],c
ount[3]);
    if(year==2014)
        printf("%d\t%ld\t%ld\t%ld\t%ld\t%lf\n",year,count[0],count[1],cou
nt[2],count[3],pa);
    if( year>2009){
```

```
        fprintf(out, "%d\t%ld\t%ld\t%ld\t%ld\n",year,count[0],count[1],co
unt[2],count[3]);
    }
}

void initializePopulation()
{
    int cnt=0;
    for( int j=15; j<65; j++){
        for( int i=0; i<populationPerYearAndAge[1950][j]*100; i++){
            person[cnt].age=j;
            person[cnt].state=0;
            cnt++;
        }
    }
    totalPersons = cnt;
}

bool checkRate(double rate)
{
    if (rate <0.001){
        double k = rand()/(1.00+RAND_MAX);
        if(k<0.001){
            rate = rate*1000;
        }
        else{
            return false;
        }
    }
    double k = rand()/(1.00+RAND_MAX);
    if ( k < rate )
        return true;
```

```
    else
        return false;
}

void changeStatusAndAge()
{
    for( int i=0; i<totalPersons; i++){
        // change state
        double k = rand()/(1.00+RAND_MAX);
        if( person[i].state==0){
            if ( checkRate(pa) &&  person[i].age<40){
                person[i].state=1;
            }
        }
        else if( person[i].state==1){
            if ( k < pg ){
                person[i].state=2;
            }
        }
        else if( person[i].state==2){
            if ( k < pk ){
                person[i].state=1;
            }
        }
        // After their 49th year, all active PWID are assumed to cease in
jecting
        if(person[i].age>49 && person[i].state==1){
            person[i].state=2;
        }
        // After their 64th year, remove
        if(person[i].age>64){
            person[i].state=3;
```

```
        }
        // death rate depending on age and state
        k = rand()/(1.00+RAND_MAX);
        if( person[i].state==0){
            if ( k < deathRatePerAge[person[i].age] ){
                person[i].state=3;
            }
        }
        else if( person[i].state==1){
            if ( k < deathRatePerAge[person[i].age] * 13.0 ){
                person[i].state=3;
            }
        }
        else if( person[i].state==2){
            if ( k < deathRatePerAge[person[i].age] ){
                person[i].state=3;
            }
        }
        // increase age one year
        person[i].age++;
    }
}

void addNewPersons(int year)
{
    // add new person [15 years old and non PWID]
    int cnt=totalPersons;
    for( int i=0; i<populationPerYearAndAge[year+1][15]*100; i++){
// 100 instead of 1000 for the population to reduce execution time
        person[cnt].age=15;
        person[cnt].state=0;
        cnt++;
```

```
    }
    // increase the total number of population
    totalPersons = cnt;
}
int main()
{
    srand(time(NULL));

    setPopulationAge();
    setDeathRate();

    getTotalPopulationPerAge();
    pa = pa_start;
    while ( pa < pa_stop){

        snprintf(filename, 100, "result_%lf.txt",pa);
        out=fopen(filename,"w");

        for( int iter=0; iter<loops ;iter++){
            initializePopulation();
            for( int year=1950; year<2020; year++){
                printTotalPersonPerState(year);
                changeStatusAndAge();
                addNewPersons(year);
            }
        }

        fclose(out);
        pa = pa + pa_step;
    }
```

```
}
```

## REFERENCES

European Centre for Disease Prevention and Control. 2016. "Epidemiological Assessment of Hepatitis b and c Among Migrants in the EU/EEA." https://www.ecdc.europa.eu/sites/default/files/media/en/publications/Publications/epidemiological-assessment-hepatitis-B-and-C-among-migrants-EU-EEA.pdf.

European Centre for Disease Prevention and Control (ECDC). 2016. "Systematic Review on Hepatitis b and c Prevalence in the EU/EEA." https://www.ecdc.europa.eu/sites/default/files/media/en/publications/Publications/systematic-review-hepatitis-B-C-prevalence.pdf.

———. 2021. "Hepatitis c Prevalence Database." https://www.ecdc.europa.eu/en/all-topics-z/hepatitis-c/tools/hepatitis-c-prevalence-database.

Graf, Christiana, Marcus M Mücke, Georg Dultz, Kai-Henrik Peiffer, Alica Kubesch, Patrick Ingiliz, Stefan Zeuzem, Eva Herrmann, and Johannes Vermehren. 2019. "Efficacy of Direct-acting Antivirals for Chronic Hepatitis C Virus Infection in People Who Inject Drugs or Receive Opioid Substitution Therapy: A Systematic Review and Meta-analysis." *Clinical Infectious Diseases* 70 (11): 2355–65. https://doi.org/10.1093/cid/ciz696.

Grebely, Jason, Sarah Larney, Amy Peacock, Samantha Colledge, Janni Leung, Matthew Hickman, Peter Vickerman, et al. 2019. "Global, Regional, and Country-Level Estimates of Hepatitis c Infection Among People Who Have Recently Injected Drugs." *Addiction* 114 (1): 150–66. https://doi.org/https://doi.org/10.1111/add.14393.

Hines, Lindsey A., Adam Trickey, Janni Leung, Sarah Larney, Amy Peacock, Louisa Degenhardt, Samantha Colledge, et al. 2020. "Associations Between National Development Indicators and the Age Profile of People Who Inject Drugs: Results from a Global Systematic Review and Meta-Analysis." *The Lancet Global Health* 8 (1): e76–91. https://doi.org/10.1016/S2214-109X(19)30462-0.

Lampertico, Pietro, Jose A. Carrión, Michael Curry, Juan Turnes, Markus Cornberg, Francesco Negro, Ashley Brown, et al. 2020. "Real-World Effectiveness and Safety of Glecaprevir/Pibrentasvir for the Treatment of Patients with Chronic HCV Infection:

A meta-Analysis." *Journal of Hepatology* 72 (6): 1112–21. https://doi.org/10.1016/j.jhep.2020.01.025.

Lin, Lifeng, and Haitao Chu. 2020. "Meta-Analysis of Proportions Using Generalized Linear Mixed Models." *Epidemiology* 31 (5). https://journals.lww.com/epidem/Fulltext/2020/09000/Meta_analysis_of_Proportions_Using_Generalized.16.aspx.

McDonald, Scott A., Rosmawati Mohamed, Maznah Dahlui, Herlianna Naning, and Adeeba Kamarulzaman. 2014. "Bridging the Data Gaps in the Epidemiology of Hepatitis c Virus Infection in Malaysia Using Multi-Parameter Evidence Synthesis." *BMC Infectious Diseases* 14 (1): 564. https://doi.org/10.1186/s12879-014-0564-6.

Micallef, J. M., J. M. Kaldor, and G. J. Dore. 2006. "Spontaneous Viral Clearance Following Acute Hepatitis c Infection: A Systematic Review of Longitudinal Studies." *Journal of Viral Hepatitis* 13 (1): 34–41. https://doi.org/https://doi.org/10.1111/j.1365-2893.2005.00651.x.

Presanis, Anne M., O. Noel Gill, Timothy R. Chadborn, Caterina Hill, Vivian Hope, Louise Logan, Brian D. Rice, Valerie C. Delpech, A. E. Ades, and Daniela De Angelis. 2010. "Insights into the Rise in HIV Infections, 2001 to 2008: A Bayesian Synthesis of Prevalence Evidence." *AIDS* 24 (18). https://journals.lww.com/aidsonline/Fulltext/2010/11270/Insights_into_the_rise_in_HIV_infections,_2001_to.12.aspx.

Sweeting, M. J., D. De Angelis, M. Hickman, and A. E. Ades. 2008. "Estimating hepatitis C prevalence in England and Wales by synthesizing evidence from multiple data sources. Assessing data conflict and model fit." *Biostatistics* 9 (4): 715–34. https://doi.org/10.1093/biostatistics/kxn004.

Tan, Sarah, Susanna Makela, Daliah Heller, Kevin Konty, Sharon Balter, Tian Zheng, and James H. Stark. 2018. "A Bayesian Evidence Synthesis Approach to Estimate Disease Prevalence in Hard-to-Reach Populations: Hepatitis c in New York City." *Epidemics* 23: 96–109. https://doi.org/https://doi.org/10.1016/j.epidem.2018.01.002.

Thein, Hla-Hla, Qilong Yi, Gregory J. Dore, and Murray D. Krahn. 2008. "Estimation of Stage-Specific Fibrosis Progression Rates in Chronic Hepatitis c Virus Infection: A Meta-Analysis and

Meta-Regression.” *Hepatology* 48 (2): 418–31. https://doi.org/https://doi.org/10.1002/hep.22375.

Veen, Maaike G. van, Anne M. Presanis, Stefano Conti, Maria Xiridou, Annemarie R. Stengaard, Martin C. Donoghoe, Ard I. van Sighem, Marianne A. van der Sande, and Daniela De Angelis. 2011. “National Estimate of HIV Prevalence in the Netherlands: Comparison and Applicability of Different Estimation Tools.” *AIDS* 25 (2). https://journals.lww.com/aidsonline/Fulltext/2011/01140/National_estimate_of_HIV_prevalence_in_the.14.aspx.

# Estimating the prevalence of chronic hepatitis C infection (CHC) in the Netherlands using Bayesian multiparameter evidence synthesis

13/07/2022

## Table of Contents


Introduction ........ 2
Methods ........ 2
Prevalence of ex-PWID ........ 3
Prevalence of recent PWID ........ 3
Prevalence of CHC among recent PWID ........ 4
Prevalence of CHC among ex-PWID ........ 4
Prevalence of CHC among non-PWID ........ 5
Sensitivity analyses ........ 6
Results ........ 7
Tables and Figures ........ 9
Appendix ........ 16
Fit of the multi-state Markov model ........ 16
Stan code for Bayesian multiparameter evidence synthesis ........ 17
Multi-state Markov model ........ 23
References ........ 31

## INTRODUCTION

Individuals with hepatitis C (HCV) infection often remain asymptomatic for decades and even for life, but a proportion of them develop chronic hepatitis, which is a progressive disease (Thein et al. 2008). Because of the largely asymptomatic nature of HCV infection, notification data reflect national screening and testing practices and do not give accurate insights into the prevalence of HCV infection. Although there are studies that have estimated the prevalence of HCV antibodies (anti-HCV) in specific groups such as the general population, first-time blood donors, or people who inject drugs (PWID) at drug treatment centers, those alone cannot be combined to produce national estimates unless some additional information regarding the composition of each HCV risk group and its prevalence in the population are known (Sweeting et al. 2008; McDonald et al. 2014; Tan et al. 2018).

## METHODS

Bayesian multiparameter evidence synthesis (MPES) has been a popular approach to formally estimate anti-HCV or HIV prevalence (Sweeting et al. 2008; McDonald et al. 2014; Tan et al. 2018; Presanis et al. 2010; Veen et al. 2011). In this project, we extend this method to estimating the prevalence of chronic hepatitis C infection (CHC), defined as an HCV-RNA positive result [i.e., active (viremic) infection is used as a proxy of chronic disease], with the population (15-79 years) split into three main non-overlapping risk groups, i.e., recent PWID (those who have injected in the last year; without including probably those injecting for chemsex), ex-PWID, and non-PWID. A unified model is assumed including parameters associated with the CHC prevalence of recent, ex-PWID, and non-PWID (denoted by $\pi_{rec}$, $\pi_{ex}$, and $\pi_{non}$, respectively) and the prevalence of these risk groups in the population (denoted by $\rho_{rec}$, $\rho_{ex}$, and $\rho_{non}$, respectively; $\rho_{rec} + \rho_{ex} + \rho_{non} = 1$). To estimate the CHC prevalence in the whole population, $\pi$, we used the formula:

$$\pi = \pi_{rec}\rho_{rec} + \pi_{ex}\rho_{ex} + \pi_{non}\rho_{non} \quad (1)$$

Bayesian synthesis considers all available data, allowing for an evidence-based estimation of CHC prevalence in the population, with inherent uncertainty properly accounted for. Our aim is to estimate the CHC prevalence for the total (15-79) population of the Netherlands in 2019 using sources of information over the last decade (2010-2019).

## Prevalence of ex-PWID

Data on the prevalence of ex-PWID (proportion of the population that is ex-PWID) are sparse and generally often unreliable. To overcome this limitation, we apply the method proposed by McDonald et al. (2014), which requires simulating a multi-state Markov model representing the non-PWID, recent PWID, and ex-PWID. In the multi-state Markov model, the simulation starts in 1950 and examines the population aged 15-79 years old. A certain number of 15-year-old individuals enter the simulation process each year. These numbers of 15-year-old individuals are obtained from the United Nations Statistic Division UNSD, being different for each country. The ageing of the population is also taken into account. Country-specific overall mortality data are retrieved from the Life Tables of the World Health Organization WHO. The duration of injecting career is also country-specific and obtained from the paper of Vos et al. (2013).

To estimate the number of ex-PWID, the model can be calibrated on the number of recent PWID provided in the EMCDDA barometer or the EMCDDA statistical bulletin (the code of the Markov model can be found in the Appendix). If there are no available data in the barometer, estimates from the systematic review of Grebely et al. (2019) could be used. Currently, the multi-state Markov model to estimate the prevalence of recent and ex-PWID was calibrated on the estimates reported in the EMCDDA barometer.

After applying the model for the Netherlands, we compute the number (and the corresponding Confidence Interval - CI) of ex-PWID in 2019. The number of ex-PWID is then divided by the population size (15-79 years) in the same year, and a Normal distribution, constrained such that $\rho_{ex} \in (0,1)$, is assumed for $\rho_{ex}$. The standard deviation of this distribution is specified to approximately correspond to the respective CI, i.e. $\left(UB_{\rho_{ex}} - LB_{\rho_{ex}}\right)/(2 \times 1.96)$, where $UB_{\rho_{ex}}$ and $LB_{\rho_{ex}}$ denote the upper and lower limit of the CI.

## Prevalence of recent PWID

The number of recent PWID in the Netherlands in 2019 is also projected by the multi-state Markov model described in the previous subsection. To estimate $\rho_{rec}$, the number of recent PWID is divided by the population size (15-79 years). Similar to the previous subsection, a Normal distribution, constrained such that $\rho_{rec} \in (0,1)$, is assumed for $\rho_{rec}$, with the standard deviation specified to correspond to the CI obtained from the multi-state Markov model.

## Prevalence of CHC among recent PWID

The prevalence of CHC among recent PWID was informed by CHC prevalence data reported in the paper of Grebely et al. (2019) for 2011-2014. However, some people may have been treated with direct-acting antivirals (DAAs), with the sustained virologic response ($SVR_{PWID}$) among PWID estimated to be 88% (95% CI: 80% to 93%) (Graf et al. 2019). Based on data from the Pharmaceutical Key Figures Foundation, as provided by the national focal points, the number of individuals treated with DAAs up to 2019 is approximately equal to 7,000. However, the proportions of each risk group among those treated with DAAs, i.e. $\rho_{rec|DAA}$, $\rho_{ex|DAA}$, and $\rho_{non|DAA}$ ($\rho_{rec|DAA} + \rho_{ex|DAA} + \rho_{non|DAA} = 1$), are not currently available in the Netherlands. In this report, we make the assumption that $\rho_{rec|DAA}$ is equal to the proportion of recent PWID among CHC-positive individuals, i.e. Pr(Recent PWID|CHC), as estimated by our model when the DAA uptake is ignored (Table 2). Similarly, we assume that $\rho_{ex|DAA}$ = Pr(Ex-PWID|CHC) and $\rho_{non|DAA}$ = Pr(Non-PWID|CHC). Thus, the CHC prevalence among recent PWID, adjusted for DAAs, can be estimated by

$$\pi_{rec} = \frac{N_{15,79}\rho_{rec}\tilde{\pi}_{rec} - N_{DAA}\rho_{rec|DAA}SVR_{PWID}}{N_{15,79}\rho_{rec}}, \quad (2)$$

where $\tilde{\pi}_{rec}$ denotes the CHC estimate derived solely from the data reported in Grebely et al. (2019).

## Prevalence of CHC among ex-PWID

Information on CHC prevalence among ex-PWID (i.e., $\pi_{ex}$) is difficult to obtain directly. To overcome this, anti-HCV prevalence data from ever PWID in Amsterdam in 2017, available in the EMCDDA statistical bulletin, were used. However, as these data refer to the anti-HCV prevalence, they should be adjusted similarly to the procedure described in the previous subsection, i.e.

$$\pi_{ever} = \frac{N_{15,79}\rho_{ever}\pi(\text{anti-HCV})_{ever}(1-\rho_{clear}) - N_{DAA}\rho_{ever|DAA}SVR_{PWID}}{N_{15,79}\rho_{ever}}, \quad (3)$$

where $\pi(\text{anti-HCV})_{ever}$ denotes the anti-HCV prevalence among ever PWID, $\rho_{ever} = \rho_{rec} + \rho_{ex}$, $\rho_{clear}$ denotes the spontaneous viral clearance [assumed to be equal to 0.26 (95% CI 0.22–

0.29); (Micallef, Kaldor, and Dore 2006)], and $\rho_{ever|DAA}$ the proportion of ever PWID among individuals treated with DAAs. Recall that it is assumed that $\rho_{ever|DAA}$ is assumed to be equal to Pr(Ever PWID|CHC), as estimated by our model ignoring the effect of DAAs.

Then, since an estimate of $\pi_{ever}$ is available, $\pi_{ex}$ can be indirectly estimated since $\pi_{ever}$ is equal to a weighted average of $\pi_{rec}$ and $\pi_{ex}$, as shown by the following formula

$$\pi_{ever} = \frac{\rho_{rec}}{\rho_{ever}}\pi_{rec} + \frac{\rho_{ex}}{\rho_{ever}}\pi_{ex}, \quad (4)$$

or, equivalently,

$$\pi_{ex} = \left(\pi_{ever} - \frac{\rho_{rec}}{\rho_{ever}}\pi_{rec}\right) \times \frac{\rho_{ever}}{\rho_{ex}}. \quad (5)$$

## Prevalence of CHC among non-PWID

To estimate the CHC prevalence among non-PWID in a country (after 2010), the ECDC database (European Centre for Disease Prevention and Control (ECDC) 2021) was used. The ECDC group has carefully and critically collected information from relevant studies across the EU/EEA region in cooperation with the focal points of each country. On the basis of the representativeness of each study and other factors (European Centre for Disease Prevention and Control (ECDC) 2016), a risk of bias score was assigned to each study, with higher values indicating higher-quality studies. When higher-quality studies are available in a country (≥ 4), they are used to estimate the overall CHC prevalence in that country among non-PWID. If there are no higher-quality studies, general population estimates with a lower quality (<4) are pooled. If no general population prevalence estimates are available, data on the prevalence of HCV in pregnant women could be used as a prevalence measure. Finally, if data on pregnant women are lacking too, first-time blood donor studies could be an option. However, if the national focal point agrees or recommends, estimates from a neighboring country could be used instead, or we could use any other relevant information provided/suggested by the national focal point.

In the Netherlands, there were 2 studies (Heil et al. 2018; Zuure et al. 2017) of medium and unknown quality, respectively, which included CHC data. Thus, individuals treated with DAAs should be removed, with the sustained virologic response ($SVR$) in the general population

estimated to be 96.7% (95% CI: 95.4% to 98.1%) (Lampertico et al. 2020). Similarly to the procedure described in the previous subsections, the CHC prevalence among non-PWID, adjusted for DAAs, can be estimated by

$$\pi_{non} = \frac{N_{15,79}\rho_{non}\tilde{\pi}_{non} - N_{DAA}\rho_{non|DAA}SVR}{N_{15,79}\rho_{non}}. \quad (6)$$

where $\tilde{\pi}_{non}$ denotes the CHC prevalence estimate ignoring the contribution of DAAs.

As $\rho_{rec}$ and $\rho_{ex}$ are likely to be low, $\pi_{non}$ will be the most critical factor for the estimation of the overall CHC prevalence in the population. To adjust for potential heterogeneity between the CHC prevalence estimates from different studies in the non-PWID population in a country, as a sensitivity analysis, we also apply a random-effect meta-analytic approach to pool the CHC prevalence across studies on non-PWID (Lin and Chu 2020). The random-effect approach is carried out only when at least 3 studies are available in the general population of a certain country, and the estimates are compared with the corresponding estimates from the approach assuming no heterogeneity (similar to a fixed-effect meta-analysis). If the estimates are inconsistent, possible explanations are investigated and discussed. If there is substantial heterogeneity between studies, it would help more to explore the causes; for example, following national focal point suggestions, some studies could be excluded to reduce heterogeneity.

## Sensitivity analyses

As discussed with the national focal points, men having sex with men (MSM) may contribute considerably to the HCV epidemic in the Netherlands. Therefore, if we do not explicitly account for MSM, the results for the non-PWID population could be biased by the proportion of MSM included. It should be also noted that the proportion of MSM who inject drugs in the Netherlands is very low (Coyer et al. 2022; Newsum et al. 2021; Smit et al. 2021). Thus, there is no serious overlapping of the MSM and the PWID population, which implies that MSM could be considered as a fourth risk group in our analysis. Given that the HCV epidemic has been mostly concentrated in MSM with HIV (Newsum et al. 2018) and data for MSM without HIV are more sparse, we considered MSM with HIV as a separate risk group. The prevalence of MSM with HIV ($\rho_{MSM}$) is about 0.1% in the Netherlands (Koopsen et al. 2019) (corresponding number 13,650), whereas the CHC prevalence among MSM with HIV in 2019 (denoted by $\pi_{MSM}$) was informed

by data reported in the paper of Isfordink et al. (2022). Then the overall CHC prevalence can be estimated by

$$\pi = \pi_{rec}\rho_{rec} + \pi_{ex}\rho_{ex} + \pi_{MSM}\rho_{MSM} + \pi_{non}\rho_{non},$$

where $\rho_{rec} + \rho_{ex} + \rho_{MSM} + \rho_{non} = 1$. A treatment adjustment similar to that described in the previous subsections was also performed.

A second sensitivity analysis including first-generation migrants from HCV endemic countries as a separate risk group was also carried out. Based on the paper of Koopsen et al. (2019), first-generation migrants from HCV endemic countries represent 11.1% of the adult population in the Netherlands (Table 2 in Koopsen et al. 2019), with the respective CHC prevalence being equal to $\pi_{mig} = 0.9\%$ (Table 2 in Koopsen et al. 2019). The overall CHC prevalence can be now estimated by

$$\pi = \pi_{rec}\rho_{rec} + \pi_{ex}\rho_{ex} + \pi_{mig}\rho_{mig} + \pi_{non}\rho_{non},$$

where $\rho_{rec} + \rho_{ex} + \rho_{mig} + \rho_{non} = 1$. A treatment adjustment similar to that described in the previous subsections was also performed.

# RESULTS

The above-mentioned approach was used to estimate the prevalence of CHC in 2019 (considering ages between 15-79 years: 13,743,524).

The aggregated data used by our approach are briefly presented in Table 1. The multi-state Markov model to estimate the prevalence of recent and ex-PWID was calibrated on the estimates reported in the EMCDDA barometer.

In the Netherlands, there were 2 studies (Heil et al. 2018; Zuure et al. 2017) on non-PWID of medium and unknown quality, respectively, which included CHC data. To estimate the CHC prevalence in the general population (primarily non-PWID), we used a fixed-effect meta-analytic approach, with the corresponding results, when information on DAAs is ignored, presented in Table 2. However, based on data provided by the focal point, approximately 7,000 individuals were treated with DAAs from 2015 to 2019 in the Netherlands, with the proportion of recent PWID, ex-PWID, and non-PWID among the 7,000 treated individuals assumed to be

equal to Pr(Recent PWID|CHC) ≈ 2.66% Pr(Ex-PWID|CHC) ≈ 22.48% and Pr(Non-PWID|CHC) ≈ 74.86%, respectively (Table 2).

The corresponding results accounting for the DAA uptake are presented in Table 3. The prevalence of recent and ex-PWID was low in the Netherlands (about 0.01% and 0.03%, respectively) corresponding to 800 (95% CI: 630-970) recent PWID and 4,410 (95% CI: 3,990-4,830) ex-PWID in the population. However, the CHC prevalence in these groups was substantial (Table 3) being 20.98% and 31.7%, respectively. This translates to 165 (95% CI: 0-280) and 1,393 (95% CI: 0-2,387) recent and ex-PWID aged 15-79 living with CHC infection in the Netherlands in 2019. The CHC prevalence in the general population was 0.03% (95% CI: 0%-0.14%), much lower than that of the high-risk groups. Taking all pieces of information into account, the overall CHC prevalence in the Netherlands in 2019 was equal to 0.04% (95% CI: 0%-0.16%), which corresponds to 5,927 (95% CI: 0-21,637.12) individuals aged 15-79 years with CHC infection. When MSM with HIV are considered as a separate risk group (Table 4), the results remain very similar, i.e. the total CHC prevalence is equal to 0.04% (95% CI: 0%-0.16%), which corresponds to 6,183 (95% CI: 0-21,759.12) individuals aged 15-79 years with CHC infection.

The results from our model including migrants from endemic countries as a separate group are presented in Table 5. However, this analysis comes with possible limitations; that is, including migrants as a separate group is valid only if migrants do not overlap with the remaining groups (recent PWID, ex-PWID, and non-PWID). Thus, if migrants do participate proportionally in the study(ies) in the general population, this analysis may result in biased overall CHC estimates (most probably in higher CHC prevalence estimates). Thus, results from Table 5 can be interpreted only as an upper bound for the CHC prevalence in the Netherlands.

# TABLES AND FIGURES

*Table 1. Data (real) contributing to the estimation of CHC prevalence in the Netherlands in 2019.*

| Parameter | Estimate (95%CI) | Number (95%CI) | Numerator | Denominator | Notes | Year of study |
|---|---|---|---|---|---|---|
| $\rho_{rec}$ | | 800 (630-970) | | | Method based on McDonald et al. | 2019 |
| $\rho_{ex}$ | | 4,410 (3,990-4,830) | | | Method based on McDonald et al. | 2019 |
| $\pi_{rec}$ | 41.5% (36.7%-46.3%) | | | | Grebely et al. | 2011-2014 |
| $\pi(\text{anti-HCV})_{ever}$ | | | 12 | 14 | EMCDDA database | 2017 |
| $\pi_{MSM}$ | | | 448 | 11,489 | (Isfordink et al. 2022) | 2015 |
| $\pi_{MSM}$ | | | 63 | 12,697 | (Isfordink et al. 2022) | 2019 |
| $\pi_{non}$ | | | 0 | 3,427 | ECDC database (Heil et al.); Risk of bias=2† | 2014 |
| $\pi_{non}$ | | | 2 | 500 | ECDC database | 2014 |

| Parameter | Estimate (95%CI) | Number (95%CI) | Numerator | Denominator | Notes | Year of study |
|---|---|---|---|---|---|---|
| | | | | | (Zuure et al.); Risk of bias=NA†† | |

*Notes*: Although it looks counter-intuitive, **higher risk of bias score denotes a higher-quality study** (range from 0 to 6); † Seven individuals reported a history of injecting drug use; †† Including only those of Dutch origin.

*Table 2. Results from the method assuming no heterogeneity in the estimation of the CHC prevalence in the general population from different studies (i.e., similar to a fixed-effect meta-analysis).*

| Parameter | Posterior median | Posterior sd | LB (2.5%) | UB (97.5%) |
|---|---|---|---|---|
| $\rho_{rec}$ (%) | 0.01 | 0 | 0 | 0.01 |
| $\rho_{ex}$ (%) | 0.03 | 0 | 0.03 | 0.04 |
| $\pi_{rec}$ (%) | 41.52 | 2.42 | 36.76 | 46.21 |
| $\pi_{ex}$ (%) | 64.67 | 8.56 | 44.28 | 77.18 |
| $\pi_{non}$ (%) | 0.07 | 0.04 | 0.02 | 0.18 |
| $\pi$ (%) | 0.09 | 0.04 | 0.04 | 0.21 |
| Number with CHC | 12,442 | 5,958 | 5,308 | 28,236 |
| Pr(Recent PWID\|CHC) (%) | 2.66 | 1.38 | 1.12 | 6.37 |
| Pr(Ex-PWID\|CHC) (%) | 22.48 | 11.27 | 9.33 | 52.97 |
| Pr(Non-PWID\|CHC) (%) | 74.86 | 12.52 | 41.16 | 89.47 |

*Notes*: Information on DAAs is not taken into account.

*Abbreviations*: $\rho_{rec}$, prevalence of recent PWID (proportion of the population that is recent PWID); $\rho_{ex}$, prevalence of ex-PWID (proportion of the population that is ex-PWID); $\pi_{rec}$, CHC prevalence among recent PWID; $\pi_{ex}$, CHC prevalence among ex-PWID; $\pi_{non}$, CHC prevalence among non-PWID; $\pi$ overall CHC prevalence in the Netherlands; LB, Lower Boundary; UP, Upper Boundary

*Table 3. Results from the method assuming no heterogeneity in the estimation of the CHC prevalence in the general population from different studies (i.e., similar to a fixed-effect meta-analysis).*

| Parameter | Posterior median | Posterior sd | LB (2.5%) | UB (97.5%) |
|---|---|---|---|---|
| $\rho_{rec}$ (%) | 0.01 | 0 | 0 | 0.01 |
| $\rho_{ex}$ (%) | 0.03 | 0 | 0.03 | 0.04 |
| $\pi_{rec}$ (%) | 20.98 | 10.31 | 0 | 33.16 |
| $\pi_{ex}$ (%) | 31.7 | 16.2 | 0 | 53.79 |
| $\pi_{non}$ (%) | 0.03 | 0.04 | 0 | 0.14 |
| $\pi$ (%) | 0.04 | 0.04 | 0 | 0.16 |
| Number with CHC | 5,927 | 5,986 | 0 | 21,637 |
| Pr(Recent PWID\|CHC) (%) | 2.74 | 560.27 | 0.96 | 8.71 |
| Pr(Ex-PWID\|CHC) (%) | 23.24 | 4038.85 | 7.98 | 71.74 |
| Pr(Non-PWID\|CHC) (%) | 73.94 | 4598.92 | 19.83 | 90.92 |

*Notes*: The number of individuals treated with DAAs is taken into account.

*Abbreviations*: $\rho_{rec}$, prevalence of recent PWID (proportion of the population that is recent PWID); $\rho_{ex}$, prevalence of ex-PWID (proportion of the population that is ex-PWID); $\pi_{rec}$, CHC prevalence among recent PWID; $\pi_{ex}$, CHC prevalence among ex-PWID; $\pi_{non}$, CHC prevalence among non-PWID; $\pi$ overall CHC prevalence in the Netherlands; LB, Lower Boundary; UP, Upper Boundary

*Table 4. Results from the method assuming no heterogeneity in the estimation of the CHC prevalence in the general population (primarily non-PWID) from different studies (i.e., similar to a fixed-effect meta-analysis) including MSM with HIV as a separate risk group.*

| Parameter | Posterior median | Posterior sd | LB (2.5%) | UB (97.5%) |
|---|---|---|---|---|
| $\rho_{rec}$ (%) | 0.01 | 0 | 0 | 0.01 |
| $\rho_{ex}$ (%) | 0.03 | 0 | 0.03 | 0.04 |
| $\rho_{MSM}$ (%) | 0.1 | 0 | 0.1 | 0.1 |
| $\pi_{rec}$ (%) | 21.73 | 9.37 | 0 | 33.47 |
| $\pi_{ex}$ (%) | 32.89 | 14.87 | 0 | 54.25 |
| $\pi_{MSM}$ (%) | 0.5 | 0.06 | 0.39 | 0.64 |
| $\pi_{non}$ (%) | 0.03 | 0.04 | 0 | 0.14 |
| $\pi$ (%) | 0.04 | 0.04 | 0 | 0.16 |
| Number with CHC | 6,183 | 5,927 | 0 | 21,759 |
| Pr(Recent PWID\|CHC) (%) | 2.76 | 152.16 | 1.06 | 8.02 |
| Pr(Ex-PWID\|CHC) (%) | 23.28 | 1144.03 | 8.77 | 65.82 |
| Pr(MSM\|CHC) (%) | 1 | 1230.02 | 0 | 13.48 |
| Pr(Non-PWID\|CHC) (%) | 73.09 | 2525.08 | 15.95 | 90.32 |

*Notes*: The number of individuals treated with DAAs is taken into account.

*Abbreviations*: $\rho_{rec}$, prevalence of recent PWID (proportion of the population that is recent PWID); $\rho_{ex}$, prevalence of ex-PWID (proportion of the population that is ex-PWID); $\rho_{MSM}$, prevalence of MSM with HIV (proportion of the population that belongs to this group); $\pi_{rec}$, CHC prevalence among recent PWID; $\pi_{ex}$, CHC prevalence among ex-PWID; $\pi_{resultsFixedMSM}$, CHC prevalence among MSM with HIV; $\pi_{non}$, CHC prevalence among non-PWID; $\pi$ overall CHC prevalence in the Netherlands; LB, Lower Boundary; UP, Upper Boundary; MSM, men having sex with men.

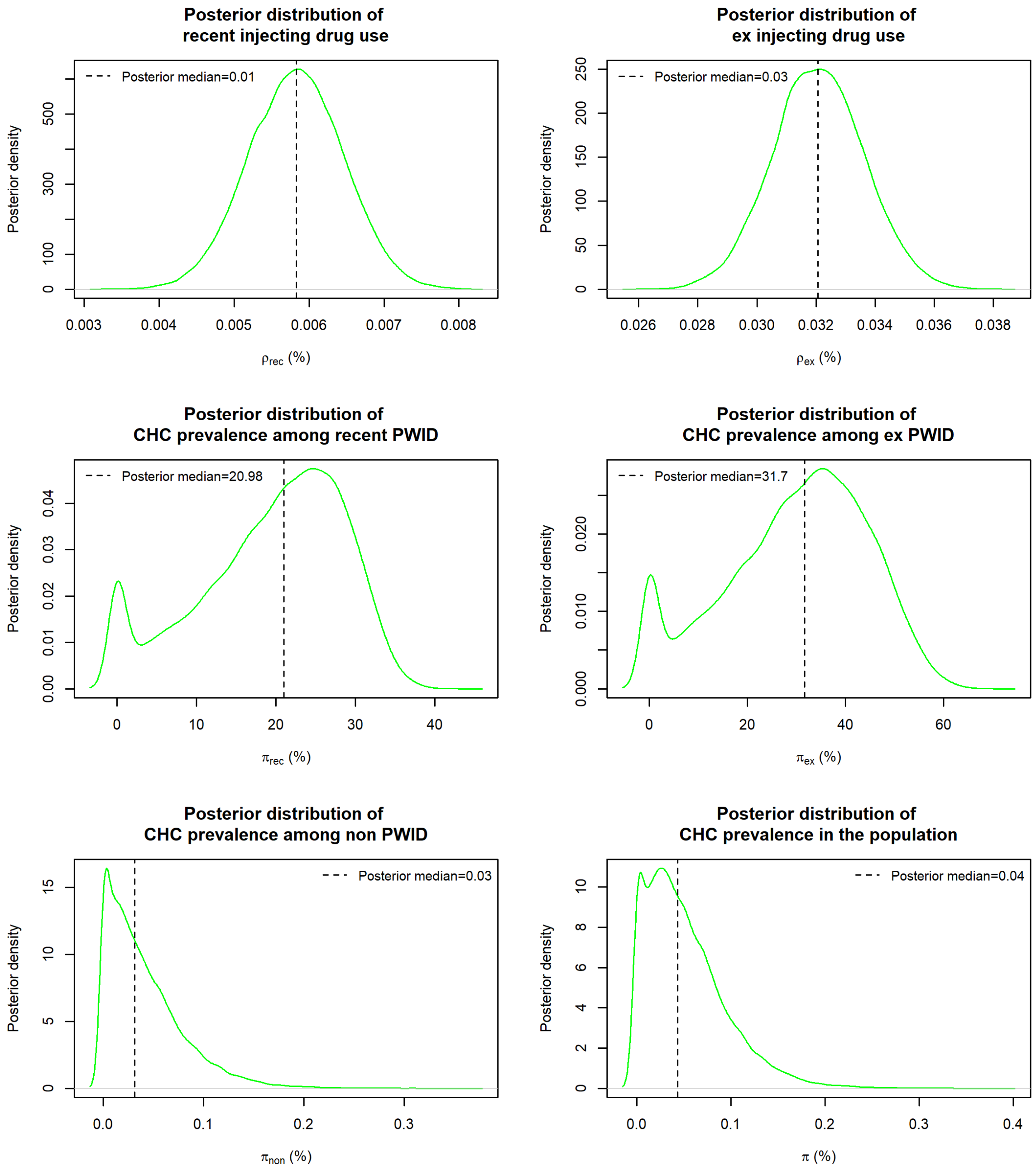


*Figure 1. Posterior distributions of the key model parameters using the approach that assumes no heterogeneity in the estimation of the CHC prevalence in the general population (primarily non-PWID) from different studies (i.e., similar to a fixed-effect meta-analysis), with the number of individuals treated with DAA taken into account.*

*Table 5. Results from the method assuming no heterogeneity in the estimation of the CHC prevalence in the general population (primarily non-PWID) from different studies (i.e., similar to a fixed-effect meta-analysis) including migrants from endemic countries as a separate group, with data based on Koopsen et al. (2019).*

| Parameter | Posterior median | Posterior sd | LB (2.5%) | UB (97.5%) |
|---|---|---|---|---|
| $\rho_{rec}$ (%) | 0.01 | 0 | 0 | 0.01 |
| $\rho_{ex}$ (%) | 0.03 | 0 | 0.03 | 0.04 |
| $\rho_{mig}$ (%) | 11.11 | 0 | 11.11 | 11.11 |
| $\pi_{rec}$ (%) | 31.29 | 3.58 | 23.04 | 37.04 |
| $\pi_{ex}$ (%) | 48.14 | 8.08 | 30.2 | 61.22 |
| $\pi_{mig}$ (%) | 0.66 | 0.25 | 0.2 | 1.16 |
| $\pi_{non}$ (%) | 0.05 | 0.04 | 0.01 | 0.15 |
| $\pi$ (%) | 0.14 | 0.05 | 0.06 | 0.25 |
| Number with CHC | 18,882 | 6,780 | 7,760 | 34,240 |
| Pr(Recent PWID\|CHC) (%) | 1.33 | 0.48 | 0.77 | 2.55 |
| Pr(Ex-PWID\|CHC) (%) | 11.22 | 3.98 | 6.34 | 21.29 |
| Pr(Mig\|CHC) (%) | 53.56 | 12.91 | 25.87 | 75.61 |
| Pr(Non-PWID\|CHC) (%) | 33.21 | 13.71 | 9.82 | 61.86 |

*Notes*: The number of individuals treated with DAAs is taken into account.

*Abbreviations*: $\rho_{rec}$, prevalence of recent PWID (proportion of the population that is recent PWID); $\rho_{ex}$, prevalence of ex-PWID (proportion of the population that is ex-PWID); $\rho_{mig}$, prevalence of migrants from endemic countries (proportion of the population that belongs to this group); $\pi_{rec}$, CHC prevalence among recent PWID; $\pi_{ex}$, CHC prevalence among ex-PWID; $\pi_{mig}$, CHC prevalence among migrants from endemic countries; $\pi_{non}$, CHC prevalence among non-PWID; $\pi$ overall CHC prevalence in the Netherlands; LB, Lower Boundary; UP, Upper Boundary

# APPENDIX

## Fit of the multi-state Markov model

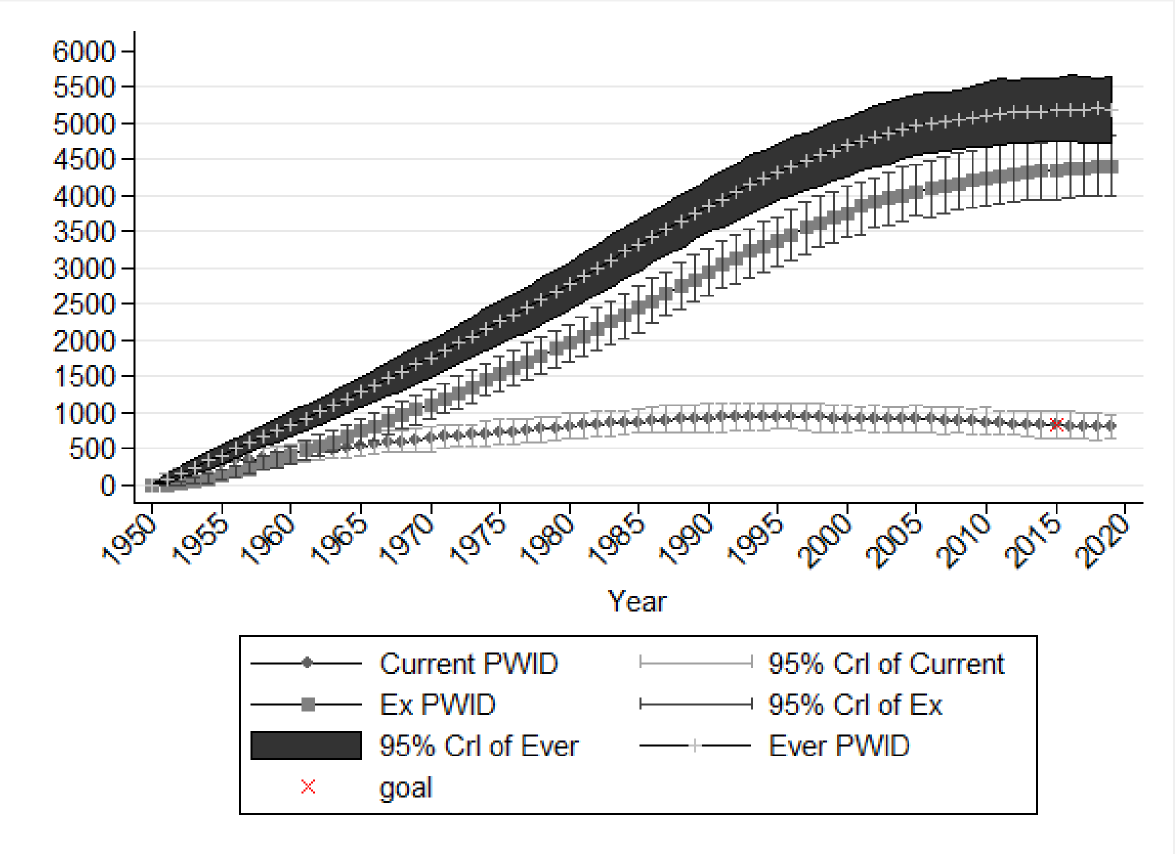


*Figure 2: Model predictions for the numbers of current and ex-PWID (people who inject drugs). The solid black line and shaded grey error bars show the median and 95% credible intervals (95% CrI) for the model projections. For comparison, asterisks indicate the observed country's data.*

## Stan code for Bayesian multiparameter evidence synthesis

```
data {
  int<lower=0> N1579; // Population of 15-79 in `Country`
  int<lower=1> Knon; // Number of studies in the ECDC data for `Country`
  int<lower=1> Kever; // Number of studies in the EMCDDA data for ever users in `Country`
  int<lower=1> Kcur; // Number of studies in the EMCDDA barometer (for HCV prevalence of PWID) in `Country`
  int<lower=1> NDAA; // Total number of DAAs from 2015 to 2019

  real p_cur_mean; // Prior mean for the prevalence of current use in `Country`
  real<lower=0> p_cur_sd; // Prior sd for the prevalence of current use in `Country`

  real p_ex_mean; // Prior mean for the prevalence of ex-use in `Country`
  real<lower=0> p_ex_sd; // Prior sd for the prevalence of ex-use in `Country`

  //int<lower=0> Nst_hcv_cur[Kcur]; // Number of individuals in the study estimating anti-HCV among recent PWID
  //int<lower=0> Yst_hcv_cur[Kcur]; // Number of HCV+ in the study estimating anti-HCV among recent PWID
  real p_CHC_cur_mean; // Prior mean for the CHC prevalence among recent PWID in `Country`
  real<lower=0> p_CHC_cur_sd; // Prior sd for the CHC prevalence among recent PWID in `Country`

  int<lower=0> Nst_hcv_ever[Kever]; // Number of individuals in the study estimating HCV among ever IDU
  int<lower=0> Yst_hcv_ever[Kever]; // Number of HCV+ in the study estimating HCV among ever PWID
```

```
  int<lower=0> Nst_CHC_non[Knon]; // Number of individuals in the study e
stimating CHC among non PWID
  int<lower=0> Yst_CHC_non[Knon]; // Number of HCV+ in the study estimati
ng CHC among non PWID

  vector<lower=0>[3] alpha; // parameter of the Diriclet prior

  real HCVclear_mean; // Prior mean for the HCV clearance probability
  real<lower=0> HCVclear_sd; // Prior sd for the HCV clearance probabilit
y

  real SVR_mean; // Prior mean for the SVR among non-PWID
  real<lower=0> SVR_sd; // Prior sd for the SVR among non-PWID

  real SVR_PWID_mean; // Prior mean for the SVR among PWID
  real<lower=0> SVR_PWID_sd; // Prior sd for the SVR among PWID
}

// Block defining the original parameters
parameters {
  // The parameters to be sampled
  simplex[3] rho; // Prevalence of the three risk groups
  real<lower=0,upper=1> CHCpi_cur; // P(CHC+|Cur)
  real<lower=0,upper=1> CHCpi_ex; // P(CHC+|Ex)
  real<lower=0,upper=1> CHCpi_non; // P(CHC+|Non)
  real<lower=0,upper=1> SVR; // SVR of DAAs among non-PWID
  real<lower=0,upper=1> SVR_PWID; // SVR of DAAs among PWID

  real<lower=0,upper=1-CHCpi_cur> HCVclear; // Probability of HCV clearan
ce; upper bound = 1-prevalence of chronic HCV
}
```

```
transformed parameters {
  // Change scales
  real<lower=0,upper=1> rho_ever;
  real<lower=0,upper=1> CHCpi_ever;
  real<lower=0,upper=1> pi_ever;

  rho_ever = rho[1] + rho[2];
  CHCpi_ever = CHCpi_cur*rho[1]/rho_ever + CHCpi_ex*rho[2]/rho_ever;
  pi_ever = CHCpi_ever/(1-HCVclear);
}


// Binomial regression model
model {
  // Priors
  rho ~ dirichlet(alpha);

  //////////////////////////////
  // Likelihood contributions //
  //////////////////////////////

  // Probability of HCV clearance
  HCVclear ~ normal(HCVclear_mean,HCVclear_sd);

  // Prevalence of current use
  rho[1] ~ normal(p_cur_mean,p_cur_sd);

  // Prevalence of ex-use
  rho[2] ~ normal(p_ex_mean,p_ex_sd);
```

```
  // Prevalence of chronic HCV among current users
  CHCpi_cur ~ normal(p_CHC_cur_mean,p_CHC_cur_sd);
  //Yst_hcv_cur ~ binomial(Nst_hcv_cur,pi_cur);

  // Prevalence of HCV among ever users
  Yst_hcv_ever ~ binomial(Nst_hcv_ever,pi_ever);

  // HCV+ among non
  Yst_CHC_non ~ binomial(Nst_CHC_non,CHCpi_non);

  // SVR of DAAs among non-PWID
  SVR ~ normal(SVR_mean,SVR_sd);

  // SVR of DAAs among PWID
  SVR_PWID ~ normal(SVR_PWID_mean,SVR_PWID_sd);
}


generated quantities {
  // Functions of parameters
  real<lower=0,upper=100> overalCHC;
  real logit_CHCpi_cur;
  real logit_CHCpi_ex;
  real logit_CHCpi_non;
  real logit_rho_cur;
  real logit_rho_ex;
  real logit_rho_non;
  real<lower=0,upper=1> pEverGivenCHC;
  real<lower=0,upper=1> pCurGivenCHC;
  real<lower=0,upper=1> pExGivenCHC;
  real<lower=0,upper=1> pNonGivenCHC;
  real logit_HCVclear;
```

```
  real<lower=0> NumberCHC;

  real CHCDAApi_non; // P(CHC+|Non) adjusted for DAA
  real CHCDAApi_cur; // P(CHC+|Cur) adjusted for DAA
  real CHCDAApi_ex; // P(CHC+|Ex) adjusted for DAA
  real CHCDAApi_ever; // P(CHC+|Ever) adjusted for DAA

  real overalCHC_DAA;
  real pEverGivenCHC_DAA;
  real pCurGivenCHC_DAA;
  real pExGivenCHC_DAA;
  real pNonGivenCHC_DAA;
  real NumberCHC_DAA;

  // Overall HCV prevalence
  overalCHC = 100*(rho[1]*CHCpi_cur + rho[2]*CHCpi_ex +  rho[3]*CHCpi_non
);
  pEverGivenCHC = CHCpi_ever*rho_ever/(overalCHC/100);
  pCurGivenCHC = CHCpi_cur*rho[1]/(overalCHC/100);
  pExGivenCHC = CHCpi_ex*rho[2]/(overalCHC/100);
  pNonGivenCHC = CHCpi_non*(1-rho_ever)/(overalCHC/100);
  NumberCHC = round(overalCHC*N1579/100);

  CHCDAApi_non = ( N1579*rho[3]*CHCpi_non - NDAA*pNonGivenCHC*SVR)/(N1579
*rho[3]);
  CHCDAApi_cur = ( N1579*rho[1]*CHCpi_cur - NDAA*pCurGivenCHC*SVR_PWID)/(
N1579*rho[1]);
  CHCDAApi_ex = ( N1579*rho[2]*CHCpi_ex - NDAA*pExGivenCHC*SVR_PWID)/(N15
79*rho[2]);
  CHCDAApi_ever = CHCDAApi_cur*rho[1]/rho_ever + CHCDAApi_ex*rho[2]/rho_e
ver;
```

```
  overalCHC_DAA = 100*(rho[1]*CHCDAApi_cur + rho[2]*CHCDAApi_ex +  rho[3]
*CHCDAApi_non);
  pEverGivenCHC_DAA = CHCDAApi_ever*rho_ever/(overalCHC_DAA/100);
  pCurGivenCHC_DAA = CHCDAApi_cur*rho[1]/(overalCHC_DAA/100);
  pExGivenCHC_DAA = CHCDAApi_ex*rho[2]/(overalCHC_DAA/100);
  pNonGivenCHC_DAA = CHCDAApi_non*(1-rho_ever)/(overalCHC_DAA/100);
  NumberCHC_DAA = round(overalCHC_DAA*N1579/100);


  logit_rho_cur = logit(rho[1]);
  logit_rho_ex = logit(rho[2]);
  logit_rho_non = logit(rho[3]);
  logit_CHCpi_cur = logit(CHCpi_cur);
  logit_CHCpi_ex = logit(CHCpi_ex);
  logit_CHCpi_non = logit(CHCpi_non);
  logit_HCVclear = logit(HCVclear);
}
```

## Multi-state Markov model

```
#include <stdio.h>
#include <stdlib.h>
#include <string.h>
#include <math.h>
#include <time.h>

#define loops 100
int populationPerYearAndAge[2021][66];
double deathRatePerAge[66];
int totalPopulationPerYear[2021];

FILE *F_DeathRate=fopen("`Country`-deathRates.txt","r");
FILE *F_Population=fopen("`Country`-population.txt","r");
FILE *out;

double pa_start = 0.00012;                    // Setting Variable
double pa_step  = 0.000002;                   // Setting Variable
double pa_stop  = 0.000123;                   // Setting Variable

double pa;
double pg = 1.0/11.7;    // Assumed average active injecting career of  11
.7 years
double pk = 0.004*12.0;      // Relapse rate of 0.004/month

struct people{
    int age;
    int state;
};

char filename[100];
struct people person[50000000];
```

```
long int totalPersons=0;

void setPopulationAge()
{
    for( int j=0; j<71; j++){
        for( int i=0; i<51; i++){
            fscanf(F_Population,"%d",&populationPerYearAndAge[1950+j][15+
i]);
        }
    }
}

void setDeathRate()
{
    for( int i=0; i<51; i++){
        fscanf(F_DeathRate,"%lf",&deathRatePerAge[15+i*5]);
        for( int j=0; j<5; j++){
            deathRatePerAge[15+i*5+j] = deathRatePerAge[15+i*5];
        }
    }
}

void getTotalPopulationPerAge()
{
    for( int j=0; j<71; j++){
        for( int i=0; i<51; i++){
            totalPopulationPerYear[1950+j] += populationPerYearAndAge[195
0+j][15+i];
        }
    }
}
```

```
void printTotalPopulationPerAge()
{
    printf("---- - ----------\n");
    printf("Year - Population\n");
    printf("---- - ----------\n");
    for( int i=1950; i<2021; i++){
        printf("%d - %d\n",i,totalPopulationPerYear[i]);
    }
}

void printDeathRatePerAge()
{
    printf("--- - -----\n");
    printf("Age - Rate\n");
    printf("--- - -----\n");
    for( int i=15; i<65; i++){
        printf("%d - %lf\n",i,deathRatePerAge[i]);
    }
}

void printTotalPersonPerState( int year)
{
    int count[4]= {0,0,0,0};
    for( int i=0; i<totalPersons; i++){
        count[person[i].state]++;
    }
    //printf("%d\t%ld\t%ld\t%ld\t%ld\n",year,count[0],count[1],count[2],c
ount[3]);
    if(year==2014)
        printf("%d\t%ld\t%ld\t%ld\t%ld\t%lf\n",year,count[0],count[1],cou
nt[2],count[3],pa);
    if( year>2009){
```

```
        fprintf(out, "%d\t%ld\t%ld\t%ld\t%ld\n",year,count[0],count[1],co
unt[2],count[3]);
    }
}

void initializePopulation()
{
    int cnt=0;
    for( int j=15; j<65; j++){
        for( int i=0; i<populationPerYearAndAge[1950][j]*100; i++){
            person[cnt].age=j;
            person[cnt].state=0;
            cnt++;
        }
    }
    totalPersons = cnt;
}

bool checkRate(double rate)
{
    if (rate <0.001){
        double k = rand()/(1.00+RAND_MAX);
        if(k<0.001){
            rate = rate*1000;
        }
        else{
            return false;
        }
    }
    double k = rand()/(1.00+RAND_MAX);
    if ( k < rate )
        return true;
```

```
    else
        return false;
}

void changeStatusAndAge()
{
    for( int i=0; i<totalPersons; i++){
        // change state
        double k = rand()/(1.00+RAND_MAX);
        if( person[i].state==0){
            if ( checkRate(pa) &&  person[i].age<40){
                person[i].state=1;
            }
        }
        else if( person[i].state==1){
            if ( k < pg ){
                person[i].state=2;
            }
        }
        else if( person[i].state==2){
            if ( k < pk ){
                person[i].state=1;
            }
        }
        // After their 49th year, all active PWID are assumed to cease in
jecting
        if(person[i].age>49 && person[i].state==1){
            person[i].state=2;
        }
        // After their 64th year, remove
        if(person[i].age>64){
            person[i].state=3;
```

```
        }
        // death rate depending on age and state
        k = rand()/(1.00+RAND_MAX);
        if( person[i].state==0){
            if ( k < deathRatePerAge[person[i].age] ){
                person[i].state=3;
            }
        }
        else if( person[i].state==1){
            if ( k < deathRatePerAge[person[i].age] * 13.0 ){
                person[i].state=3;
            }
        }
        else if( person[i].state==2){
            if ( k < deathRatePerAge[person[i].age] ){
                person[i].state=3;
            }
        }
        // increase age one year
        person[i].age++;
    }
}

void addNewPersons(int year)
{
    // add new person [15 years old and non PWID]
    int cnt=totalPersons;
    for( int i=0; i<populationPerYearAndAge[year+1][15]*100; i++){
// 100 instead of 1000 for the population to reduce execution time
        person[cnt].age=15;
        person[cnt].state=0;
        cnt++;
```

```
    }
    // increase the total number of population
    totalPersons = cnt;
}
int main()
{
    srand(time(NULL));

    setPopulationAge();
    setDeathRate();

    getTotalPopulationPerAge();
    pa = pa_start;
    while ( pa < pa_stop){

        snprintf(filename, 100, "result_%lf.txt",pa);
        out=fopen(filename,"w");

        for( int iter=0; iter<loops ;iter++){
            initializePopulation();
            for( int year=1950; year<2020; year++){
                printTotalPersonPerState(year);
                changeStatusAndAge();
                addNewPersons(year);
            }
        }

        fclose(out);
        pa = pa + pa_step;
    }
```

```
}
```

# REFERENCES

Coyer, Liza, Anders Boyd, Udi Davidovich, Ward P. H. van Bilsen, Maria Prins, and Amy Matser. 2022. "Increase in Recreational Drug Use Between 2008 and 2018: Results from a Prospective Cohort Study Among HIV-Negative Men Who Have Sex with Men." *Addiction* 117 (3): 656–65. https://doi.org/https://doi.org/10.1111/add.15666.

European Centre for Disease Prevention and Control (ECDC). 2016. "Systematic Review on Hepatitis b and c Prevalence in the EU/EEA." https://www.ecdc.europa.eu/sites/default/files/media/en/publications/Publications/systematic-review-hepatitis-B-C-prevalence.pdf.

———. 2021. "Hepatitis c Prevalence Database." https://www.ecdc.europa.eu/en/all-topics-z/hepatitis-c/tools/hepatitis-c-prevalence-database.

Graf, Christiana, Marcus M Mücke, Georg Dultz, Kai-Henrik Peiffer, Alica Kubesch, Patrick Ingiliz, Stefan Zeuzem, Eva Herrmann, and Johannes Vermehren. 2019. "Efficacy of Direct-acting Antivirals for Chronic Hepatitis C Virus Infection in People Who Inject Drugs or Receive Opioid Substitution Therapy: A Systematic Review and Meta-analysis." *Clinical Infectious Diseases* 70 (11): 2355–65. https://doi.org/10.1093/cid/ciz696.

Grebely, Jason, Sarah Larney, Amy Peacock, Samantha Colledge, Janni Leung, Matthew Hickman, Peter Vickerman, et al. 2019. "Global, Regional, and Country-Level Estimates of Hepatitis c Infection Among People Who Have Recently Injected Drugs." *Addiction* 114 (1): 150–66. https://doi.org/https://doi.org/10.1111/add.14393.

Heil, Jeanne, Christian J. P. A. Hoebe, Jochen W. L. Cals, Henriëtte L. G. ter Waarbeek, Inge H. M. van Loo, and Nicole H. T. M. Dukers-Muijrers. 2018. "Detecting Hepatitis b and c by Combined Public Health and Primary Care Birth Cohort Testing." *The Annals of Family Medicine* 16 (1): 21–27. https://doi.org/10.1370/afm.2166.

Isfordink, Cas J., Colette Smit, Anders Boyd, Marieke J. A. de Regt, Bart J. A. Rijnders, Reinout van Crevel, Robin P. Ackens, Peter Reiss, Joop E. Arends, and Marc van der Valk. 2022. "Low Hepatitis c Virus-Viremia Prevalence yet Continued Barriers to Direct-Acting Antiviral Treatment in People Living with HIV in the Netherlands." *AIDS* 36 (6).

https://journals.lww.com/aidsonline/Fulltext/2022/05010/Low_hepatitis_C_virus_viremia_prevalence_yet.3.aspx.

Koopsen, J., J. E. van Steenbergen, J. H. Richardus, M. Prins, E. L. M. Op de Coul, E. A. Croes, J. Heil, F. R. Zuure, and I. K. Veldhuijzen. 2019. "Chronic Hepatitis b and c Infections in the Netherlands: Estimated Prevalence in Risk Groups and the General Population." *Epidemiology and Infection* 147: e147. https://doi.org/10.1017/S0950268819000359.

Lampertico, Pietro, Jose A. Carrión, Michael Curry, Juan Turnes, Markus Cornberg, Francesco Negro, Ashley Brown, et al. 2020. "Real-World Effectiveness and Safety of Glecaprevir/Pibrentasvir for the Treatment of Patients with Chronic HCV Infection: A meta-Analysis." *Journal of Hepatology* 72 (6): 1112–21. https://doi.org/10.1016/j.jhep.2020.01.025.

Lin, Lifeng, and Haitao Chu. 2020. "Meta-Analysis of Proportions Using Generalized Linear Mixed Models." *Epidemiology* 31 (5). https://journals.lww.com/epidem/Fulltext/2020/09000/Meta_analysis_of_Proportions_Using_Generalized.16.aspx.

McDonald, Scott A., Rosmawati Mohamed, Maznah Dahlui, Herlianna Naning, and Adeeba Kamarulzaman. 2014. "Bridging the Data Gaps in the Epidemiology of Hepatitis c Virus Infection in Malaysia Using Multi-Parameter Evidence Synthesis." *BMC Infectious Diseases* 14 (1): 564. https://doi.org/10.1186/s12879-014-0564-6.

Micallef, J. M., J. M. Kaldor, and G. J. Dore. 2006. "Spontaneous Viral Clearance Following Acute Hepatitis c Infection: A Systematic Review of Longitudinal Studies." *Journal of Viral Hepatitis* 13 (1): 34–41. https://doi.org/https://doi.org/10.1111/j.1365-2893.2005.00651.x.

Newsum, Astrid M., Amy Matser, Janke Schinkel, Marc van der Valk, Kees Brinkman, Arne van Eeden, Fanny N. Lauw, et al. 2021. "Incidence of HCV Reinfection Among HIV-Positive MSM and Its Association with Sexual Risk Behavior: A Longitudinal Analysis." *Clinical Infectious Diseases* 73 (3): 460–67. https://doi.org/10.1093/cid/ciaa645.

Newsum, Astrid M., Martijn S. van Rooijen, Michelle Kroone, Sylvia M. Bruisten, Amy Matser, Arjan Hogewoning, Maria Prins, and Titia Heijman. 2018. "Stable Low Hepatitis c Virus

Antibody Prevalence Among HIV-Negative Men Who Have Sex with Men Attending the Sexually Transmitted Infection Outpatient Clinic in Amsterdam, 2007 to 2017." *Sexually Transmitted Diseases* 45 (12). https://journals.lww.com/stdjournal/Fulltext/2018/12000/Stable_Low_Hepatitis_C_Virus_Antibody_Prevalence.8.aspx.

Presanis, Anne M., O. Noel Gill, Timothy R. Chadborn, Caterina Hill, Vivian Hope, Louise Logan, Brian D. Rice, Valerie C. Delpech, A. E. Ades, and Daniela De Angelis. 2010. "Insights into the Rise in HIV Infections, 2001 to 2008: A Bayesian Synthesis of Prevalence Evidence." *AIDS* 24 (18). https://journals.lww.com/aidsonline/Fulltext/2010/11270/Insights_into_the_rise_in_HIV_infections,_2001_to.12.aspx.

Smit, Colette, Anders Boyd, Bart J. A. Rijnders, Thijs J. W. van de Laar, Eliane M. Leyten, Wouter F. Bierman, Kees Brinkman, et al. 2021. "HCV Micro-Elimination in Individuals with HIV in the Netherlands 4 Years After Universal Access to Direct-Acting Antivirals: A Retrospective Cohort Study." *The Lancet HIV* 8 (2): e96–105. https://www.sciencedirect.com/science/article/pii/S2352301820303015.

Sweeting, M. J., D. De Angelis, M. Hickman, and A. E. Ades. 2008. "Estimating hepatitis C prevalence in England and Wales by synthesizing evidence from multiple data sources. Assessing data conflict and model fit." *Biostatistics* 9 (4): 715–34. https://doi.org/10.1093/biostatistics/kxn004.

Tan, Sarah, Susanna Makela, Daliah Heller, Kevin Konty, Sharon Balter, Tian Zheng, and James H. Stark. 2018. "A Bayesian Evidence Synthesis Approach to Estimate Disease Prevalence in Hard-to-Reach Populations: Hepatitis c in New York City." *Epidemics* 23: 96–109. https://doi.org/https://doi.org/10.1016/j.epidem.2018.01.002.

Thein, Hla-Hla, Qilong Yi, Gregory J. Dore, and Murray D. Krahn. 2008. "Estimation of Stage-Specific Fibrosis Progression Rates in Chronic Hepatitis c Virus Infection: A Meta-Analysis and Meta-Regression." *Hepatology* 48 (2): 418–31. https://doi.org/https://doi.org/10.1002/hep.22375.

Veen, Maaike G. van, Anne M. Presanis, Stefano Conti, Maria Xiridou, Annemarie R. Stengaard, Martin C. Donoghoe, Ard I. van Sighem, Marianne A. van der Sande, and Daniela De Angelis.

2011. "National Estimate of HIV Prevalence in the Netherlands: Comparison and Applicability of Different Estimation Tools." *AIDS* 25 (2). https://journals.lww.com/aidsonline/Fulltext/2011/01140/National_estimate_of_HIV_prevalence_in_the.14.aspx.

Vos, Anneke S. de, Jannie J. van der Helm, Amy Matser, Maria Prins, and Mirjam E. E. Kretzschmar. 2013. "Decline in Incidence of HIV and Hepatitis c Virus Infection Among Injecting Drug Users in Amsterdam; Evidence for Harm Reduction?" *Addiction* 108 (6): 1070–81. https://doi.org/https://doi.org/10.1111/add.12125.

Zuure, F., J. Bil, M. Visser, G. van Rijckevorsel, M. Snijder, P. Blom, G. Sonder, J. Schinkel, and M. Prins. 2017. "FRI-459 - Prevalence of Hepatitis b Virus and Hepatitis c Virus Infection Among Six Ethnic Groups Living in Amsterdam, the Netherlands - the HELIUS Study." *Journal of Hepatology* 66 (1, Supplement): S405. https://doi.org/https://doi.org/10.1016/S0168-8278(17)31169-8.

# Estimating the prevalence of chronic hepatitis C infection (CHC) in Norway using Bayesian multiparameter evidence synthesis

04/07/2022

## Table of Contents


Introduction ........ 2

Methods ........ 2

Prevalence of ex-PWID ........ 3

Prevalence of recent PWID ........ 3

Prevalence of CHC among recent PWID ........ 4

Prevalence of CHC among ex-PWID ........ 4

Prevalence of CHC among non-PWID ........ 5

Results ........ 6

Tables and Figures ........ 7

Appendix ........ 11

Fit of the multi-state Markov model ........ 11

Stan code for Bayesian multiparameter evidence synthesis ........ 12

Multi-state Markov model ........ 18

References ........ 26

# INTRODUCTION

Patients with chronic hepatitis C (HCV) infection often remain asymptomatic for decades and even for life, but a proportion of them develop active chronic hepatitis, which is a progressive disease (Thein et al. 2008). Because of the largely asymptomatic nature of HCV infection, notification data reflect national screening and testing practices and do not give accurate insights into the prevalence of HCV infection. Although there are studies that have estimated the prevalence of HCV antibodies (anti-HCV) in specific groups such as the general population, first-time blood donors, or people who inject drugs (PWID) at drug treatment centers, those alone cannot be combined to produce national estimates unless some additional information regarding the composition of each HCV risk group and its prevalence in the population are known (Sweeting et al. 2008; McDonald et al. 2014; Tan et al. 2018).

# METHODS

Bayesian multiparameter evidence synthesis (BMES) has been a popular approach to formally estimate anti-HCV or HIV prevalence (Sweeting et al. 2008; McDonald et al. 2014; Tan et al. 2018; Presanis et al. 2010; Veen et al. 2011). In this project, we extend this method to estimating the CHC prevalence, with the population (15-79 years) split into three main non-overlapping risk groups, i.e., recent PWID (those who have injected in the last year), ex-PWID, and non-PWID. A unified model is assumed including parameters associated with the CHC prevalence of recent, ex-PWID, and non-PWID (denoted by $\pi_{rec}$, $\pi_{ex}$, and $\pi_{non}$, respectively) and the prevalence of these risk groups in the population (denoted by $\rho_{rec}$, $\rho_{ex}$, and $\rho_{non}$, respectively; $\rho_{rec} + \rho_{ex} + \rho_{non} = 1$). To estimate the CHC prevalence in the whole population, $\pi$, we used the formula:

$$\pi = \pi_{rec}\rho_{rec} + \pi_{ex}\rho_{ex} + \pi_{non}\rho_{non} \quad (1)$$

Bayesian synthesis considers all available data, allowing for an evidence-based estimation of CHC prevalence in the population, with inherent uncertainty properly accounted for. Our aim is to estimate the CHC prevalence for the total (15-79) population of Norway in 2019 using sources of information over the last decade (2010-2019).

## Prevalence of ex-PWID

Data on the prevalence of ex-PWID are sparse and generally often unreliable. To overcome this limitation, we apply the method proposed by McDonald et al. (2014), which requires simulating a multi-state Markov model representing the non-PWID, recent PWID, and ex-PWID. In the multi-state Markov model, the simulation starts in 1950 and examines the population aged 15-79 years old. A certain number of 15-year-old individuals enter the simulation process each year. These numbers of 15-year-old individuals are obtained from the United Nations Statistic Division UNSD, being different for each country. The ageing of the population is also taken into account. Country-specific overall mortality data are retrieved from the Life Tables of the World Health Organization WHO. The duration of injecting career is also country-specific and obtained from the paper of Hines et al. (2020).

To estimate the number of ex-PWID, the model can be calibrated on the number of recent PWID provided in the EMCDDA barometer or the EMCDDA statistical bulletin (the code of the Markov model can be found in the Appendix). If there are no available data in the barometer, estimates from the systematic review of Grebely et al. (2019) could be used. Currently, the multi-state Markov model to estimate the prevalence of recent and ex-PWID was calibrated on the estimated number of recent PWID reported in the EMCDDA barometer and the estimated number of ex-PWID available in Meijerink et al. (2017).

After applying the model for Norway, we compute the number (and the corresponding Confidence Interval - CI) of ex-PWID in 2019. The number of ex-PWID is then divided by the population size (15-79 years) in the same year, and a Normal distribution, constrained such that $\rho_{ex} \in (0,1)$, is assumed for $\rho_{ex}$. The standard deviation of this distribution is specified to approximately correspond to the respective CI, i.e. $\left(UB_{\rho_{ex}} - LB_{\rho_{ex}}\right)/(2 \times 1.96)$, where $UB_{\rho_{ex}}$ and $LB_{\rho_{ex}}$ denote the upper and lower limit of the CI.

## Prevalence of recent PWID

The number of recent PWID in Norway in 2019 is also projected by the multi-state Markov model described in the previous subsection. To estimate $\rho_{rec}$, the number of recent PWID is divided by the population size (15-79 years). Similar to the previous subsection, a Normal

distribution, constrained such that $\rho_{rec} \in (0,1)$, is assumed for $\rho_{rec}$, with the standard deviation specified to correspond to the CI obtained from the multi-state Markov model.

## Prevalence of CHC among recent PWID

The prevalence (and the 95% CI) of CHC among recent PWID was informed by data from a prevalence study on recent PWID in 2018, provided by EMCDDA through personal communication. The Binomial distribution was used in the model. However, after 2018, some people may have been treated with direct-acting antivirals (DAAs), with the sustained virologic response ($SVR_{PWID}$) among PWID estimated to be 88% (95% CI: 80% to 93%) (Graf et al. 2019). Based on information provided by the national focal point, the number of DAA prescriptions in 2016, 2017, 2018, and 2019 are 1,173, 1,953, 3,181, and 2,185, respectively. However, the proportions of each risk group among those treated with DAAs, i.e. $\rho_{rec|DAA}$, $\rho_{ex|DAA}$, and $\rho_{non|DAA}$ ($\rho_{rec|DAA} + \rho_{ex|DAA} + \rho_{non|DAA} = 1$), are not currently available in Norway. In this report, we make the assumption that $\rho_{rec|DAA}$ is equal to the proportion of recent PWID among CHC-positive individuals, i.e. Pr(Recent PWID|CHC), as estimated by our model when the DAA uptake is ignored (Table 2). Similarly, we assume that $\rho_{ex|DAA} =$ Pr(Ex-PWID|CHC) and $\rho_{non|DAA} =$ Pr(Non-PWID|CHC). Thus, the CHC prevalence among recent PWID, adjusted for DAAs, can be estimated by

$$\pi_{rec} = \frac{N_{15,79}\rho_{rec}\tilde{\pi}_{rec} - 2185 \times \rho_{rec|DAA} \times SVR_{PWID}}{N_{15,79}\rho_{rec}}, \quad (2)$$

where $\tilde{\pi}_{rec}$ denotes the CHC estimate among recent PWID provided by EMCDDA.

## Prevalence of CHC among ex-PWID

Information on CHC prevalence among ex-PWID (i.e., $\pi_{ex}$) is difficult to obtain directly. To overcome this, non-published CHC prevalence data among ever PWID in 2017, reported in the country feedback document returned to ECDC by the focal point, were used. Thus, once an estimate of $\pi_{ever}$ is available, $\pi_{ex}$ can be indirectly estimated since $\pi_{ever}$ is equal to a weighted average of $\pi_{rec}$ and $\pi_{ex}$, as shown by the following formula

$$\pi_{ever} = \frac{\rho_{rec}}{\rho_{rec} + \rho_{ex}}\pi_{rec} + \frac{\rho_{ex}}{\rho_{rec} + \rho_{ex}}\pi_{ex}, \quad (3)$$

or, equivalently,

$$\pi_{ex} = \left(\pi_{ever} - \frac{\rho_{rec}}{\rho_{rec} + \rho_{ex}}\pi_{rec}\right) \times \frac{\rho_{rec} + \rho_{ex}}{\rho_{ex}}. \quad (4)$$

However, some ex-PWID may have been treated with DAAs after 2017. Similarly to the procedure described in the previous subsection, the CHC prevalence among ex-PWID, adjusted for DAAs, can be estimated by

$$\pi_{ex} = \frac{N_{15,79}\rho_{ex}\tilde{\pi}_{ex} - 5366 \times \rho_{ex|DAA} \times SVR_{PWID}}{N_{15,79}\rho_{ex}}, \quad (5)$$

where $\tilde{\pi}_{ex}$ denotes the CHC estimate ignoring information on DAAs.

## Prevalence of CHC among non-PWID

To estimate the CHC prevalence among non-PWID in a country (after 2010), the ECDC database (European Centre for Disease Prevention and Control (ECDC) 2021) was used. The ECDC group has carefully and critically collected information from relevant studies across the EU/EEA region in cooperation with the focal points of each country. On the basis of the representativeness of each study and other factors (European Centre for Disease Prevention and Control (ECDC) 2016), a risk of bias score was assigned to each study, with higher values indicating higher-quality studies. When higher-quality studies are available in a country (≥ 4), they are used to estimate the overall CHC prevalence in that country among non-PWID. If there are no higher-quality studies, general population estimates with a lower quality (<4) are pooled. If no general population prevalence estimates are available, data on the prevalence of HCV in pregnant women could be used as a prevalence measure. Finally, if data on pregnant women are lacking too, first-time blood donor studies could be an option. However, if the national focal point agrees or recommends, estimates from a neighboring country could be used instead, or we could use any other relevant information provided/suggested by the national focal point.

In Norway, there was 1 study on non-PWID of high quality in 2015, which included CHC data (Kileng et al. 2019). Thus, individuals treated with DAAs should be removed, with the sustained virologic response ($SVR$) in the general population estimated to be 96.7% (95% CI: 95.4% to

98.1%) (Lampertico et al. 2020). Similarly to the procedure described in the previous subsections, the CHC prevalence among non-PWID, adjusted for DAAs, can be estimated by

$$\pi_{non} = \frac{N_{15,79}\rho_{non}\tilde{\pi}_{non} - 8492 \times \rho_{non|DAA} \times SVR}{N_{15,79}\rho_{non}}. \quad (6)$$

where $\tilde{\pi}_{non}$ denotes the CHC estimate obtained solely from Kileng et al. (2019).

# RESULTS

The above-mentioned approach was used to estimate the prevalence of CHC in 2019 (considering ages between 15-79 years: 4,167,255).

The aggregated data used by our approach are briefly presented in Table 1. The multi-state Markov model to estimate the prevalence of recent and ex-PWID was calibrated on the estimated number of recent PWID reported in the EMCDDA barometer and the estimated number of ex-PWID available in Meijerink et al. (2017). In Norway, there was 1 study on non-PWID of high quality, which included CHC data (Kileng et al. 2019). To estimate the CHC prevalence in the general population (primarily non-PWID), we used a fixed-effect meta-analytic approach, with the corresponding results, when information on DAAs is ignored, presented in Table 2.

However, based on data provided by the focal point, approximately 8,492 individuals were treated with DAAs up to 2019 in Norway, with the proportions of recent, ex, and non-PWID among individuals treated with DAAs assumed to be approximately equal to $\rho_{rec|DAA} =$ Pr(Recent PWID|CHC) $\approx$ 14.55%, $\rho_{ex|DAA} =$ Pr(Ex-PWID|CHC) $\approx$ 51.76% and $\rho_{non|DAA} =$ Pr(Non-PWID|CHC) $\approx$ 33.57% (Table 2).

The corresponding results accounting for DAA uptake are presented in Table 3. The prevalence of recent and ex-PWID was low in Norway (about 0.2% and 0.37%, respectively) corresponding to 8,200 (95% CI: 7,560-8,750) recent PWID and 15,430 (95% CI: 14,640-16,220) ex-PWID in the population. However, the CHC prevalence in these groups was substantial (Table 3) being 22.49% and 33.26%, respectively. This translates to 1,844 (95% CI: 1,465-2,273) and 5,133 (95% CI: 2,931-7,639) recent and ex-PWID aged 15-79 living with CHC infection in Norway in 2019. The CHC prevalence in the general population was 0.05% (95% CI: 0.02%-0.09%), much

lower than that of the high-risk groups. Taking all pieces of information into account, the overall CHC prevalence in Norway in 2019 was equal to 0.22% (95% CI: 0.14%-0.3%), which corresponds to 9,164 (95% CI: 5,954-12,631) individuals aged 15-79 years with CHC infection.

# TABLES AND FIGURES

*Table 1. Data (real) contributing to the estimation of CHC prevalence in Norway in 2019.*

| Parameter | Number (95%CI) | Numerator | Denominator | Notes | Year of study |
|---|---|---|---|---|---|
| $\rho_{rec}$ | 8,200 (7,560-8,750) | | | Method based on McDonald et al. | 2019 |
| $\rho_{ex}$ | 15,430 (14,640-16,220) | | | Method based on McDonald et al. | 2019 |
| $\pi_{rec}$ | | 69 | 267 | EMCDDA email | 2018 |
| $\pi_{ever}$ | | 27 | 66 | Country feedback data | 2017 |
| $\pi_{non}$ | | 24 | 20,937 | ECDC database (Kileng et al.); Risk of bias=4† | 2015 |

*Notes*: Higher risk of bias score denotes a higher-quality study (range from 0 to 6); † 9 CHC-positive individuals reported injecting drug use.

*Table 2. Results from the method assuming no heterogeneity in the estimation of the CHC prevalence in the general population from different studies (i.e., similar to a fixed-effect meta-analysis).*

| Parameter | Posterior median | Posterior sd | LB (2.5%) | UB (97.5%) |
|---|---|---|---|---|
| $\rho_{rec}$ (%) | 0.2 | 0.01 | 0.18 | 0.21 |
| $\rho_{ex}$ (%) | 0.37 | 0.01 | 0.35 | 0.39 |
| $\pi_{rec}$ (%) | 26.01 | 2.67 | 20.91 | 31.44 |
| $\pi_{ex}$ (%) | 49.09 | 9.2 | 31.51 | 67.81 |
| $\pi_{non}$ (%) | 0.12 | 0.02 | 0.08 | 0.17 |
| $\pi$ (%) | 0.35 | 0.04 | 0.27 | 0.44 |
| Number with CHC | 14,627 | 1,727 | 11,429 | 18,207 |
| Pr(Recent PWID\|CHC) (%) | 14.55 | 2.38 | 10.69 | 20.05 |
| Pr(Ex PWID\|CHC) (%) | 51.76 | 6.04 | 38.97 | 62.51 |
| Pr(Non-PWID\|CHC) (%) | 33.57 | 5.51 | 23.49 | 45.03 |

*Notes*: Information on DAAs is not taken into account.

*Abbreviations*: $\rho_{rec}$, prevalence of recent PWID; $\rho_{ex}$, prevalence of ex-PWID; $\pi_{rec}$, CHC prevalence among recent PWID; $\pi_{ex}$, CHC prevalence among ex-PWID; $\pi_{non}$, CHC prevalence among non-PWID; $\pi$ overall CHC prevalence in Norway; LB, Lower Boundary; UP, Upper Boundary

*Table 3. Results from the method assuming no heterogeneity in the estimation of the CHC prevalence in the general population from different studies (i.e., similar to a fixed-effect meta-analysis).*

| Parameter | Posterior median | Posterior sd | LB (2.5%) | UB (97.5%) |
|---|---|---|---|---|
| $\rho_{rec}$ (%) | 0.2 | 0.01 | 0.18 | 0.21 |
| $\rho_{ex}$ (%) | 0.37 | 0.01 | 0.35 | 0.39 |
| $\pi_{rec}$ (%) | 22.49 | 2.34 | 18.09 | 27.34 |
| $\pi_{ex}$ (%) | 33.26 | 7.77 | 19.02 | 49.43 |
| $\pi_{non}$ (%) | 0.05 | 0.02 | 0.02 | 0.09 |
| $\pi$ (%) | 0.22 | 0.04 | 0.14 | 0.3 |
| Number with CHC | 9,164 | 1,698 | 5,954 | 12,631 |
| Pr(Recent PWID\|CHC) (%) | 20.19 | 4.23 | 14.11 | 30.65 |
| Pr(Ex-PWID\|CHC) (%) | 56.06 | 5.37 | 44.65 | 65.62 |
| Pr(Non-PWID\|CHC) (%) | 23.19 | 4.83 | 14.52 | 33.36 |

*Notes*: The number of individuals treated with DAAs is taken into account.

*Abbreviations*: $\rho_{rec}$, prevalence of recent PWID; $\rho_{ex}$, prevalence of ex-PWID; $\pi_{rec}$, CHC prevalence among recent PWID; $\pi_{ex}$, CHC prevalence among ex-PWID; $\pi_{non}$, CHC prevalence among non-PWID; $\pi$ overall CHC prevalence in Norway; LB, Lower Boundary; UP, Upper Boundary

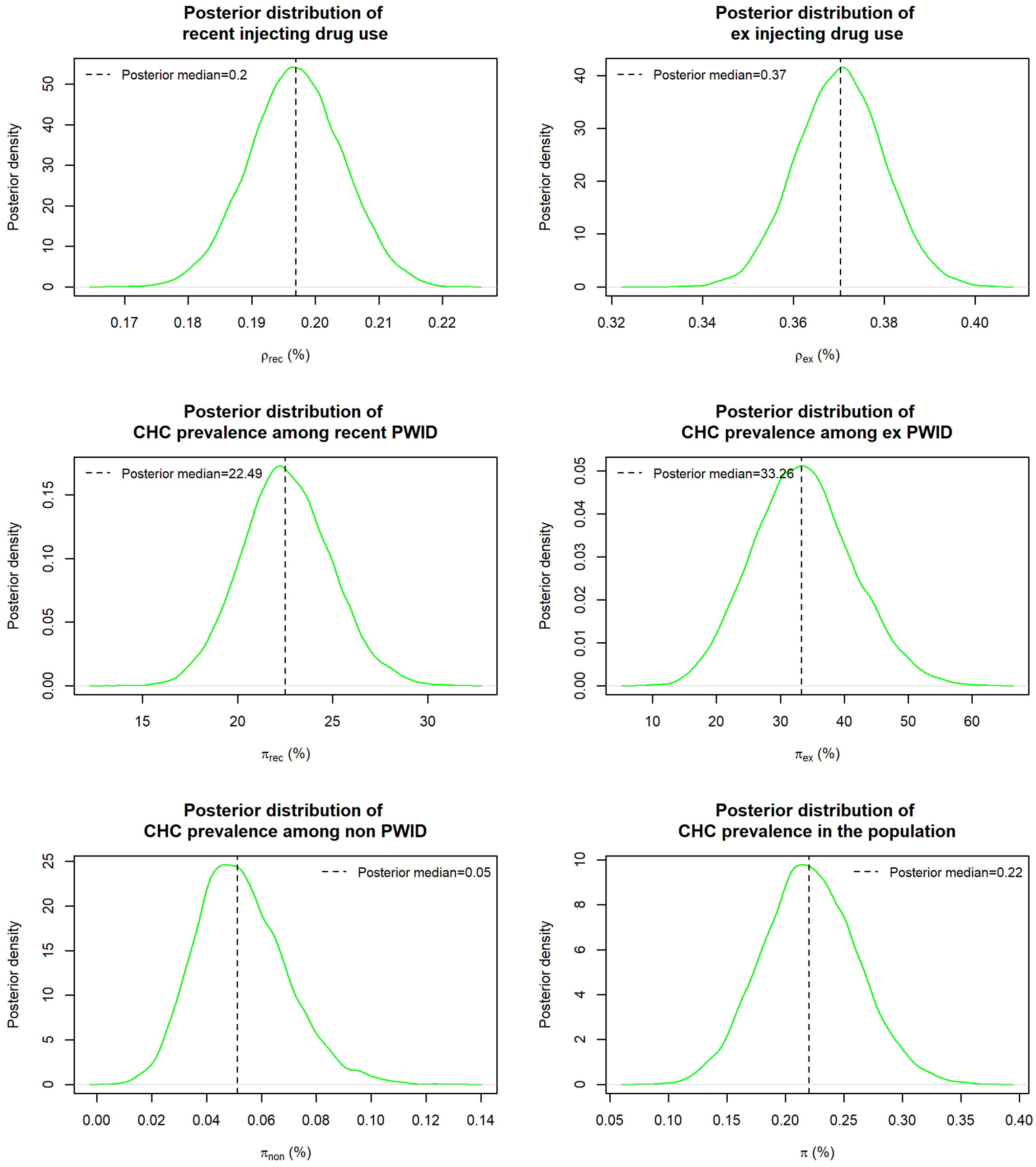


*Figure 1. Posterior distributions of the key model parameters using the approach that assumes no heterogeneity in the estimation of the CHC prevalence in the general population (primarily non-PWID) from different studies (i.e., similar to a fixed-effect meta-analysis), with the number of individuals treated with DAA taken into account.*

# APPENDIX

## Fit of the multi-state Markov model

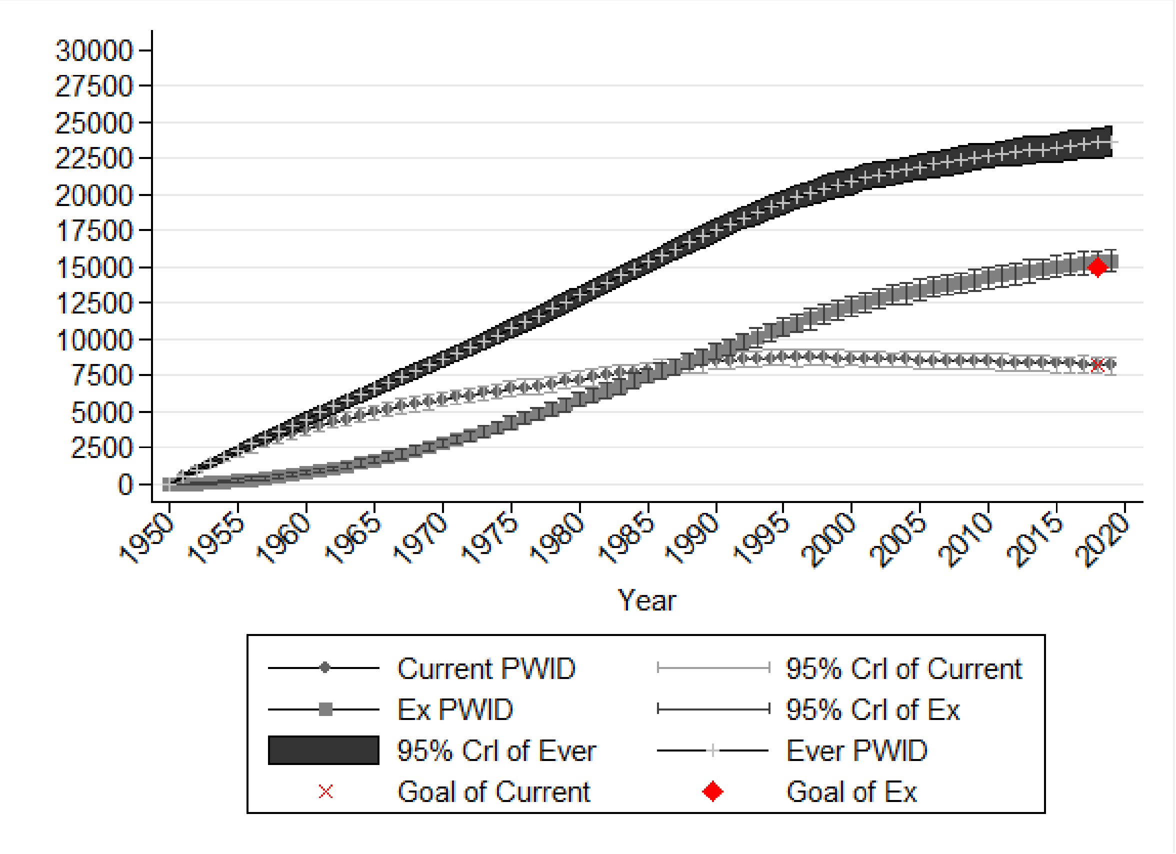


*Figure 2: Model predictions for the numbers of current and ex-PWID (people who inject drugs). The solid black line and shaded grey error bars show the median and 95% credible intervals (95% CrI) for the model projections. For comparison, asterisks indicate the observed country's data.*

## Stan code for Bayesian multiparameter evidence synthesis

```
data {
  int<lower=0> N1579; // Population of 15-64 in `Country`
  int<lower=1> Knon; // Number of studies in the ECDC data for `Country`
  int<lower=1> Kever; // Number of studies in the EMCDDA data for ever us
ers in `Country`
  int<lower=1> Kcur; // Number of studies in the EMCDDA barometer (for HC
V prevalence of PWID) in `Country`
  int<lower=1> NDAA_non; // Relevant number of DAAs in relation to the no
n-PWID study
  int<lower=1> NDAA_cur; // Relevant number of DAAs in relation to the re
cent PWID study
  int<lower=1> NDAA_ex; // Relevant number of DAAs in relation to the ex-
PWID study

  real p_cur_mean; // Prior mean for the prevalence of current use in `Co
untry`
  real<lower=0> p_cur_sd; // Prior sd for the prevalence of current use i
n `Country`

  real p_ex_mean; // Prior mean for the prevalence of ex-use in `Country`
  real<lower=0> p_ex_sd; // Prior sd for the prevalence of ex-use in `Cou
ntry`

  int<lower=0> Nst_CHC_cur[Kcur]; // Number of individuals in the study e
stimating CHC among recent PWID
  int<lower=0> Yst_CHC_cur[Kcur]; // Number of HCV+ in the study estimati
ng CHC among recent PWID

  int<lower=0> Nst_CHC_ever[Kever]; // Number of individuals in the study
estimating HCV among ever IDU
  int<lower=0> Yst_CHC_ever[Kever]; // Number of HCV+ in the study estima
```

```
ting CHC among ever PWID

  int<lower=0> Nst_CHC_non[Knon]; // Number of individuals in the study e
stimating CHC among non PWID
  int<lower=0> Yst_CHC_non[Knon]; // Number of HCV+ in the study estimati
ng CHC among non PWID

  vector<lower=0>[3] alpha; // parameter of the Diriclet prior

  real HCVclear_mean; // Prior mean for the HCV clearance probability
  real<lower=0> HCVclear_sd; // Prior sd for the HCV clearance probabilit
y

  real SVR_mean; // Prior mean for the SVR among non-PWID
  real<lower=0> SVR_sd; // Prior sd for the SVR among non-PWID

  real SVR_PWID_mean; // Prior mean for the SVR among PWID
  real<lower=0> SVR_PWID_sd; // Prior sd for the SVR among PWID

}

// Block defining the original parameters
parameters {
  // The parameters to be sampled
  simplex[3] rho; // Prevalence of the three risk groups
  real<lower=0,upper=1> CHCpi_cur; // P(CHC+|Cur)
  real<lower=0,upper=1> CHCpi_ex; // P(CHC+|Ex)
  real<lower=0,upper=1> CHCpi_non; // P(CHC+|Non)
  real<lower=0,upper=1> SVR; // SVR of DAAs among non-PWID
  real<lower=0,upper=1> SVR_PWID; // SVR of DAAs among PWID

  real<lower=0,upper=1-CHCpi_cur> HCVclear; // Probability of HCV clearan
```

```
ce; upper bound = 1-prevalence of chronic HCV
}

transformed parameters {
  // Change scales
  real<lower=0,upper=1> rho_ever;
  real<lower=0,upper=1> CHCpi_ever;

  rho_ever = rho[1] + rho[2];
  CHCpi_ever = CHCpi_cur*rho[1]/rho_ever + CHCpi_ex*rho[2]/rho_ever;
}


// Binomial regression model
model {
  // Priors
  rho ~ dirichlet(alpha);

  //////////////////////////////
  // Likelihood contributions //
  //////////////////////////////

  // Probability of HCV clearance
  HCVclear ~ normal(HCVclear_mean,HCVclear_sd);

  // Prevalence of current use
  rho[1] ~ normal(p_cur_mean,p_cur_sd);

  // Prevalence of ex-use
  rho[2] ~ normal(p_ex_mean,p_ex_sd);

  // Prevalence of chronic HCV among current users
```

```
  //CHCpi_cur ~ normal(p_CHC_cur_mean,p_CHC_cur_sd);
  Yst_CHC_cur ~ binomial(Nst_CHC_cur,CHCpi_cur);

  // Prevalence of HCV among ever users
  Yst_CHC_ever ~ binomial(Nst_CHC_ever,CHCpi_ever);

  // HCV+ among non
  Yst_CHC_non ~ binomial(Nst_CHC_non,CHCpi_non);

  // SVR of DAAs among non-PWID
  SVR ~ normal(SVR_mean,SVR_sd);

  // SVR of DAAs among PWID
  SVR_PWID ~ normal(SVR_PWID_mean,SVR_PWID_sd);
}


generated quantities {
  // Functions of parameters
  real<lower=0,upper=100> overalCHC;
  real logit_CHCpi_cur;
  real logit_CHCpi_ex;
  real logit_CHCpi_non;
  real logit_rho_cur;
  real logit_rho_ex;
  real logit_rho_non;
  real<lower=0,upper=1> pEverGivenCHC;
  real<lower=0,upper=1> pCurGivenCHC;
  real<lower=0,upper=1> pExGivenCHC;
  real<lower=0,upper=1> pNonGivenCHC;
  real logit_HCVclear;
  real<lower=0> NumberCHC;
```

```
  real<lower=0,upper=1> CHCDAApi_non; // P(CHC+|Non) adjusted for DAA
  real<lower=0,upper=1> CHCDAApi_cur; // P(CHC+|Cur) adjusted for DAA
  real<lower=0,upper=1> CHCDAApi_ex; // P(CHC+|Ex) adjusted for DAA
  real<lower=0,upper=1> CHCDAApi_ever; // P(CHC+|Ever) adjusted for DAA

  real<lower=0,upper=100> overalCHC_DAA;
  real<lower=0,upper=1> pEverGivenCHC_DAA;
  real<lower=0,upper=1> pCurGivenCHC_DAA;
  real<lower=0,upper=1> pExGivenCHC_DAA;
  real<lower=0,upper=1> pNonGivenCHC_DAA;
  real<lower=0> NumberCHC_DAA;

  // Overall HCV prevalence
  overalCHC = 100*(rho[1]*CHCpi_cur + rho[2]*CHCpi_ex +  rho[3]*CHCpi_non
);
  pEverGivenCHC = CHCpi_ever*rho_ever/(overalCHC/100);
  pCurGivenCHC = CHCpi_cur*rho[1]/(overalCHC/100);
  pExGivenCHC = CHCpi_ex*rho[2]/(overalCHC/100);
  pNonGivenCHC = CHCpi_non*(1-rho_ever)/(overalCHC/100);
  NumberCHC = round(overalCHC*N1579/100);

  CHCDAApi_non = ( N1579*rho[3]*CHCpi_non - NDAA_non*pNonGivenCHC*SVR)/(N
1579*rho[3]);
  CHCDAApi_cur = ( N1579*rho[1]*CHCpi_cur - NDAA_cur*pCurGivenCHC*SVR_PWI
D_mean)/(N1579*rho[1]);
  CHCDAApi_ex = ( N1579*rho[2]*CHCpi_ex - NDAA_ex*pExGivenCHC*SVR_PWID_me
an)/(N1579*rho[2]);
  CHCDAApi_ever = CHCDAApi_cur*rho[1]/rho_ever + CHCDAApi_ex*rho[2]/rho_e
ver;

  overalCHC_DAA = 100*(rho[1]*CHCDAApi_cur + rho[2]*CHCDAApi_ex +  rho[3]
*CHCDAApi_non);
```

```
  pEverGivenCHC_DAA = CHCDAApi_ever*rho_ever/(overalCHC_DAA/100);
  pCurGivenCHC_DAA = CHCDAApi_cur*rho[1]/(overalCHC_DAA/100);
  pExGivenCHC_DAA = CHCDAApi_ex*rho[2]/(overalCHC_DAA/100);
  pNonGivenCHC_DAA = CHCDAApi_non*(1-rho_ever)/(overalCHC_DAA/100);
  NumberCHC_DAA = round(overalCHC_DAA*N1579/100);

  logit_rho_cur = logit(rho[1]);
  logit_rho_ex = logit(rho[2]);
  logit_rho_non = logit(rho[3]);
  logit_CHCpi_cur = logit(CHCpi_cur);
  logit_CHCpi_ex = logit(CHCpi_ex);
  logit_CHCpi_non = logit(CHCpi_non);
  logit_HCVclear = logit(HCVclear);
}
```

## Multi-state Markov model

```
#include <stdio.h>
#include <stdlib.h>
#include <string.h>
#include <math.h>
#include <time.h>

#define loops 100
int populationPerYearAndAge[2021][66];
double deathRatePerAge[66];
int totalPopulationPerYear[2021];

FILE *F_DeathRate=fopen("`Country`-deathRates.txt","r");
FILE *F_Population=fopen("`Country`-population.txt","r");
FILE *out;

double pa_start = 0.00012; // Setting Variable
double pa_step = 0.000002; // Setting Variable
double pa_stop = 0.000123; // Setting Variable

double pa;
double pg = 1.0/11.7; // Assumed average active injecting career of 11
.7 years
double pk = 0.004*12.0; // Relapse rate of 0.004/month

struct people{
    int age;
    int state;
};

char filename[100];
struct people person[50000000];
```

```
long int totalPersons=0;

void setPopulationAge()
{
    for( int j=0; j<71; j++){
        for( int i=0; i<51; i++){
            fscanf(F_Population,"%d",&populationPerYearAndAge[1950+j][15+
i]);
        }
    }
}

void setDeathRate()
{
    for( int i=0; i<51; i++){
        fscanf(F_DeathRate,"%lf",&deathRatePerAge[15+i*5]);
        for( int j=0; j<5; j++){
            deathRatePerAge[15+i*5+j] = deathRatePerAge[15+i*5];
        }
    }
}

void getTotalPopulationPerAge()
{
    for( int j=0; j<71; j++){
        for( int i=0; i<51; i++){
            totalPopulationPerYear[1950+j] += populationPerYearAndAge[195
0+j][15+i];
        }
    }
}
```

```
void printTotalPopulationPerAge()
{
    printf("---- - ----------\n");
    printf("Year - Population\n");
    printf("---- - ----------\n");
    for( int i=1950; i<2021; i++){
        printf("%d - %d\n",i,totalPopulationPerYear[i]);
    }
}

void printDeathRatePerAge()
{
    printf("--- - -----\n");
    printf("Age - Rate\n");
    printf("--- - -----\n");
    for( int i=15; i<65; i++){
        printf("%d - %lf\n",i,deathRatePerAge[i]);
    }
}

void printTotalPersonPerState( int year)
{
    int count[4]= {0,0,0,0};
    for( int i=0; i<totalPersons; i++){
        count[person[i].state]++;
    }
    //printf("%d\t%ld\t%ld\t%ld\t%ld\n",year,count[0],count[1],count[2],c
ount[3]);
    if(year==2014)
        printf("%d\t%ld\t%ld\t%ld\t%ld\t%lf\n",year,count[0],count[1],cou
nt[2],count[3],pa);
    if( year>2009){
```

```
        fprintf(out, "%d\t%ld\t%ld\t%ld\t%ld\n",year,count[0],count[1],co
unt[2],count[3]);
    }
}

void initializePopulation()
{
    int cnt=0;
    for( int j=15; j<65; j++){
        for( int i=0; i<populationPerYearAndAge[1950][j]*100; i++){
            person[cnt].age=j;
            person[cnt].state=0;
            cnt++;
        }
    }
    totalPersons = cnt;
}

bool checkRate(double rate)
{
    if (rate <0.001){
        double k = rand()/(1.00+RAND_MAX);
        if(k<0.001){
            rate = rate*1000;
        }
        else{
            return false;
        }
    }
    double k = rand()/(1.00+RAND_MAX);
    if ( k < rate )
        return true;
```

```
    else
        return false;
}

void changeStatusAndAge()
{
    for( int i=0; i<totalPersons; i++){
        // change state
        double k = rand()/(1.00+RAND_MAX);
        if( person[i].state==0){
            if ( checkRate(pa) && person[i].age<40){
                person[i].state=1;
            }
        }
        else if( person[i].state==1){
            if ( k < pg ){
                person[i].state=2;
            }
        }
        else if( person[i].state==2){
            if ( k < pk ){
                person[i].state=1;
            }
        }
        // After their 49th year, all active PWID are assumed to cease in
jecting
        if(person[i].age>49 && person[i].state==1){
            person[i].state=2;
        }
        // After their 64th year, remove
        if(person[i].age>64){
            person[i].state=3;
```

```
        }
        // death rate depending on age and state
        k = rand()/(1.00+RAND_MAX);
        if( person[i].state==0){
            if ( k < deathRatePerAge[person[i].age] ){
                person[i].state=3;
            }
        }
        else if( person[i].state==1){
            if ( k < deathRatePerAge[person[i].age] * 13.0 ){
                person[i].state=3;
            }
        }
        else if( person[i].state==2){
            if ( k < deathRatePerAge[person[i].age] ){
                person[i].state=3;
            }
        }
        // increase age one year
        person[i].age++;
    }
}

void addNewPersons(int year)
{
    // add new person [15 years old and non PWID]
    int cnt=totalPersons;
    for( int i=0; i<populationPerYearAndAge[year+1][15]*100; i++){
// 100 instead of 1000 for the population to reduce execution time
        person[cnt].age=15;
        person[cnt].state=0;
        cnt++;
```

```
    }
    //      increase      the      total      number      of      population
    totalPersons                         =                         cnt;
}
int                                                                main()
{
    srand(time(NULL));

    setPopulationAge();
    setDeathRate();

    getTotalPopulationPerAge();
    pa                          =                            pa_start;
    while            (            pa            <            pa_stop){

        snprintf(filename,          100,           "result_%lf.txt",pa);
        out=fopen(filename,"w");

        for(        int        iter=0;        iter<loops        ;iter++){
            initializePopulation();
            for(      int      year=1950;      year<2020;      year++){
                printTotalPersonPerState(year);
                changeStatusAndAge();
                addNewPersons(year);
            }
        }

        fclose(out);
        pa            =            pa            +            pa_step;
    }
```

```
}
```

# REFERENCES

European Centre for Disease Prevention and Control (ECDC). 2016. "Systematic Review on Hepatitis b and c Prevalence in the EU/EEA." https://www.ecdc.europa.eu/sites/default/files/media/en/publications/Publications/systematic-review-hepatitis-B-C-prevalence.pdf.

———. 2021. "Hepatitis c Prevalence Database." https://www.ecdc.europa.eu/en/all-topics-z/hepatitis-c/tools/hepatitis-c-prevalence-database.

Graf, Christiana, Marcus M Mücke, Georg Dultz, Kai-Henrik Peiffer, Alica Kubesch, Patrick Ingiliz, Stefan Zeuzem, Eva Herrmann, and Johannes Vermehren. 2019. "Efficacy of Direct-acting Antivirals for Chronic Hepatitis C Virus Infection in People Who Inject Drugs or Receive Opioid Substitution Therapy: A Systematic Review and Meta-analysis." *Clinical Infectious Diseases* 70 (11): 2355–65. https://doi.org/10.1093/cid/ciz696.

Grebely, Jason, Sarah Larney, Amy Peacock, Samantha Colledge, Janni Leung, Matthew Hickman, Peter Vickerman, et al. 2019. "Global, Regional, and Country-Level Estimates of Hepatitis c Infection Among People Who Have Recently Injected Drugs." *Addiction* 114 (1): 150–66. https://doi.org/https://doi.org/10.1111/add.14393.

Hines, Lindsey A., Adam Trickey, Janni Leung, Sarah Larney, Amy Peacock, Louisa Degenhardt, Samantha Colledge, et al. 2020. "Associations Between National Development Indicators and the Age Profile of People Who Inject Drugs: Results from a Global Systematic Review and Meta-Analysis." *The Lancet Global Health* 8 (1): e76–91. https://doi.org/10.1016/S2214-109X(19)30462-0.

Kileng, H., T. Gutteberg, R. Goll, and E. J. Paulssen. 2019. "Screening for Hepatitis c in a General Adult Population in a Low-Prevalence Area: The Tromsø Study." *BMC Infectious Diseases* 19 (1): 189. https://doi.org/10.1186/s12879-019-3832-7.

Lampertico, Pietro, Jose A. Carrión, Michael Curry, Juan Turnes, Markus Cornberg, Francesco Negro, Ashley Brown, et al. 2020. "Real-World Effectiveness and Safety of Glecaprevir/Pibrentasvir for the Treatment of Patients with Chronic HCV Infection:

A meta-Analysis.” *Journal of Hepatology* 72 (6): 1112–21. https://doi.org/10.1016/j.jhep.2020.01.025.

McDonald, Scott A., Rosmawati Mohamed, Maznah Dahlui, Herlianna Naning, and Adeeba Kamarulzaman. 2014. “Bridging the Data Gaps in the Epidemiology of Hepatitis c Virus Infection in Malaysia Using Multi-Parameter Evidence Synthesis.” *BMC Infectious Diseases* 14 (1): 564. https://doi.org/10.1186/s12879-014-0564-6.

Meijerink, Hinta, Richard A. White, Astrid Løvlie, Birgitte Freiesleben de Blasio, Olav Dalgard, Ellen J. Amundsen, Espen Melum, and Hilde Kløvstad. 2017. “Modelling the Burden of Hepatitis c Infection Among People Who Inject Drugs in Norway, 1973–2030.” *BMC Infectious Diseases* 17 (1): 541. https://doi.org/10.1186/s12879-017-2631-2.

Presanis, Anne M., O. Noel Gill, Timothy R. Chadborn, Caterina Hill, Vivian Hope, Louise Logan, Brian D. Rice, Valerie C. Delpech, A. E. Ades, and Daniela De Angelis. 2010. “Insights into the Rise in HIV Infections, 2001 to 2008: A Bayesian Synthesis of Prevalence Evidence.” *AIDS* 24 (18). https://journals.lww.com/aidsonline/Fulltext/2010/11270/Insights_into_the_rise_in_HIV_infections,_2001_to.12.aspx.

Sweeting, M. J., D. De Angelis, M. Hickman, and A. E. Ades. 2008. “Estimating hepatitis C prevalence in England and Wales by synthesizing evidence from multiple data sources. Assessing data conflict and model fit.” *Biostatistics* 9 (4): 715–34. https://doi.org/10.1093/biostatistics/kxn004.

Tan, Sarah, Susanna Makela, Daliah Heller, Kevin Konty, Sharon Balter, Tian Zheng, and James H. Stark. 2018. “A Bayesian Evidence Synthesis Approach to Estimate Disease Prevalence in Hard-to-Reach Populations: Hepatitis c in New York City.” *Epidemics* 23: 96–109. https://doi.org/https://doi.org/10.1016/j.epidem.2018.01.002.

Thein, Hla-Hla, Qilong Yi, Gregory J. Dore, and Murray D. Krahn. 2008. “Estimation of Stage-Specific Fibrosis Progression Rates in Chronic Hepatitis c Virus Infection: A Meta-Analysis and Meta-Regression.” *Hepatology* 48 (2): 418–31. https://doi.org/https://doi.org/10.1002/hep.22375.

Veen, Maaike G. van, Anne M. Presanis, Stefano Conti, Maria Xiridou, Annemarie R. Stengaard, Martin C. Donoghoe, Ard I. van Sighem, Marianne A. van der Sande, and Daniela De Angelis. 2011. “National Estimate of HIV Prevalence in the Netherlands: Comparison and Applicability of Different Estimation Tools.” *AIDS* 25 (2). https://journals.lww.com/aidsonline/Fulltext/2011/01140/National_estimate_of_HIV_prevalence_in_the.14.aspx.

# Estimating the prevalence of chronic hepatitis C infection (CHC) in Poland using Bayesian multiparameter evidence synthesis

18/06/2022

## Table of Contents


Introduction ........ 2

Methods ........ 2

Prevalence of ex-PWID ........ 3

Prevalence of recent PWID ........ 3

Prevalence of CHC among recent PWID ........ 4

Prevalence of CHC among ex-PWID ........ 5

Prevalence of CHC among non-PWID ........ 5

Sensitivity analysis including migrants from endemic countries ........ 7

Results ........ 7

Tables and Figures ........ 9

Appendix ........ 14

Fit of the multi-state Markov model ........ 14

Stan code for Bayesian multiparameter evidence synthesis ........ 15

Multi-state Markov model ........ 21

References ........ 29

# INTRODUCTION

Patients with chronic hepatitis C (HCV) infection often remain asymptomatic for decades and even for life, but a proportion of them develop active chronic hepatitis, which is a progressive disease (Thein et al. 2008). Because of the largely asymptomatic nature of HCV infection, notification data reflect national screening and testing practices and do not give accurate insights into the prevalence of HCV infection. Although there are studies that have estimated the prevalence of HCV antibodies (anti-HCV) in specific groups such as the general population, first-time blood donors, or people who inject drugs (PWID) at drug treatment centers, those alone cannot be combined to produce national estimates unless some additional information regarding the composition of each HCV risk group and its prevalence in the population are known (Sweeting et al. 2008; McDonald et al. 2014; Tan et al. 2018).

# METHODS

Bayesian multiparameter evidence synthesis (MPES) has been a popular approach to formally estimate anti-HCV or HIV prevalence (Sweeting et al. 2008; McDonald et al. 2014; Tan et al. 2018; Presanis et al. 2010; Veen et al. 2011). In this project, we extend this method to estimating the prevalence of chronic hepatitis C infection (CHC), defined as a HCV-RNA positive result [i.e., active (viremic) infection is used as a proxy of chronic disease], with the population (15-79 years) split into three main non-overlapping risk groups, i.e., recent PWID (those who have injected in the last year; without including probably those injecting for chemsex), ex-PWID, and non-PWID. A unified model is assumed including parameters associated with the CHC prevalence of recent, ex-PWID, and non-PWID (denoted by $\pi_{rec}$, $\pi_{ex}$, and $\pi_{non}$, respectively) and the prevalence of these risk groups in the population (denoted by $\rho_{rec}$, $\rho_{ex}$, and $\rho_{non}$, respectively; $\rho_{rec} + \rho_{ex} + \rho_{non} = 1$). To estimate the CHC prevalence in the whole population, $\pi$, we used the formula:

$$\pi = \pi_{rec}\rho_{rec} + \pi_{ex}\rho_{ex} + \pi_{non}\rho_{non} \quad (1)$$

Bayesian synthesis considers all available data, allowing for an evidence-based estimation of CHC prevalence in the population, with inherent uncertainty properly accounted for. Our aim is to estimate the CHC prevalence for the total (15-79) population of Poland in 2019 using sources of information over the last decade (2010-2019).

## Prevalence of ex-PWID

Data on the prevalence of ex-PWID (proportion of the population that is ex-PWID) are sparse and generally often unreliable. To overcome this limitation, we apply the method proposed by McDonald et al. (2014), which requires simulating a multi-state Markov model representing the non-PWID, recent PWID, and ex-PWID. In the multi-state Markov model, the simulation starts in 1950 and examines the population aged 15-79 years old. A certain number of 15-year-old individuals enter the simulation process each year. These numbers of 15-year-old individuals are obtained from the United Nations Statistic Division UNSD, being different for each country. The ageing of the population is also taken into account. Country-specific overall mortality data are retrieved from the Life Tables of the World Health Organization WHO. The duration of injecting career is also country-specific and obtained from the paper of Hines et al. (2020).

To estimate the number of ex-PWID, the model can be calibrated on the number of recent PWID provided in the EMCDDA barometer or the EMCDDA statistical bulletin (the code of the Markov model can be found in the Appendix). If there are no available data in the barometer, estimates from the systematic review of Grebely et al. (2019) could be used. Alternatively, if the national focal points suggest or provide different and updated or more accurate data for calibration purposes, we will consider their advice and adjust the model accordingly.

After applying the model for Poland, we compute the number (and the corresponding Confidence Interval - CI) of ex-PWID in 2019. The number of ex-PWID is then divided by the population size (15-79 years) in the same year, and a Normal distribution, constrained such that $\rho_{ex} \in (0,1)$, is assumed for $\rho_{ex}$. The standard deviation of this distribution is specified to approximately correspond to the respective CI, i.e. $\left(UB_{\rho_{ex}} - LB_{\rho_{ex}}\right)/(2 \times 1.96)$, where $UB_{\rho_{ex}}$ and $LB_{\rho_{ex}}$ denote the upper and lower limit of the CI.

## Prevalence of recent PWID

The number of recent PWID in Poland in 2019 is also projected by the multi-state Markov model described in the previous subsection. To estimate $\rho_{rec}$, the number of recent PWID is divided by the population size (15-79 years). Similar to the previous subsection, a Normal distribution, constrained such that $\rho_{rec} \in (0,1)$, is assumed for $\rho_{rec}$, with the standard deviation specified to correspond to the CI obtained from the multi-state Markov model.

## Prevalence of CHC among recent PWID

The prevalence (and the 95% CI) of CHC among recent PWID was informed by anti-HCV prevalence data from a bio-behavioral study in 2004-2005 (Rosińska, Sierosławski, and Wiessing 2015) (injection in the last month; Table 1 of Rosińska, Sierosławski, and Wiessing (2015)), reported also in the paper of Grebely et al. (2019). The Binomial distribution was used in the model to inform $\pi_{rec}$. However, as the data reported in Rosińska, Sierosławski, and Wiessing (2015) refer to the anti-HCV prevalence and not to the CHC prevalence, they could not be used directly. This issue can be addressed by noting that spontaneous viral clearance occurs in approximately one in four people with acute hepatitis C (Micallef, Kaldor, and Dore 2006). To account for the variability of the HCV clearance probabilities, we used the result reported in Micallef, Kaldor, and Dore (2006), i.e., the proportion of HCV clearance (denoted by $\rho_{clear}$) is equal to 0.26 (95% CI 0.22–0.29). Thus, estimates of the CHC prevalence among recent PWID ($\pi_{rec}$) can be obtained using the formula $\pi_{rec} = \pi(\text{anti-HCV})_{rec}(1 - \rho_{clear})$, where $\pi(\text{anti-HCV})_{rec}$ denotes the anti-HCV prevalence among recent PWID. However, some people may have been treated with direct-acting antivirals (DAAs), with the sustained virologic response ($SVR_{PWID}$) among PWID estimated to be 88% (95% CI: 80% to 93%) (Graf et al. 2019). Based on the information provided by the national focal point, the number of individuals treated with DAAs up to 2019 is equal to 35,554.

After personal communication with the focal points, it was mentioned that active drug use is a contraindication to treatment in Poland. Therefore, in this report, we assume that the proportion of recent PWID among individuals treated with DAAs, denoted by $\rho_{rec|DAA}$, is equal to zero, and thus, treatment adjustment for the CHC prevalence among recent PWID was not performed. The proportion of ex- and non-PWID among those treated with DAAs, denoted by $\rho_{ex|DAA}$ and $\rho_{non|DAA}$, respectively, were assumed to be proportional to the corresponding proportions of ex- and non-PWID among CHC-positive individuals, i.e. $\rho_{ex|DAA} = \Pr(\text{Ex-PWID}|\text{CHC})/\{\Pr(\text{Ex-PWID}|\text{CHC}) + \Pr(\text{Non-PWID}|\text{CHC})\}$ and $\rho_{non|DAA} = \Pr(\text{Non-PWID}|\text{CHC})/\{\Pr(\text{Ex-PWID}|\text{CHC}) + \Pr(\text{Non-PWID}|\text{CHC})\}$, as estimated by our model when the DAA uptake is ignored (Table 2).

## Prevalence of CHC among ex-PWID

Information on CHC prevalence among ex-PWID (i.e., $\pi_{ex}$) is difficult to obtain directly. To overcome this, anti-HCV prevalence data from ever PWID based on a cross-sectional study conducted at four locations in 2017, available in the EMCDDA statistical bulletin and the country feedback document returned to ECDC by the focal point (the final report is also available), were used. Thus, once an estimate of $\pi(\text{anti-HCV})_{ever}$ is available, $\pi(\text{anti-HCV})_{ex}$ can be indirectly estimated since $\pi(\text{anti-HCV})_{ever}$ is equal to a weighted average of $\pi(\text{anti-HCV})_{rec}$ and $\pi(\text{anti-HCV})_{ex}$, as shown by the following formula

$$\pi(\text{anti-HCV})_{ever} = \frac{\rho_{rec}}{\rho_{rec} + \rho_{ex}} \pi(\text{anti-HCV})_{rec} + \frac{\rho_{ex}}{\rho_{rec} + \rho_{ex}} \pi(\text{anti-HCV})_{ex}, \quad (2)$$

or, equivalently,

$$\pi(\text{anti-HCV})_{ex} = \left\{ \pi(\text{anti-HCV})_{ever} - \frac{\rho_{rec}}{\rho_{rec} + \rho_{ex}} \pi(\text{anti-HCV})_{rec} \right\} \times \frac{\rho_{rec} + \rho_{ex}}{\rho_{ex}}, \quad (3)$$

However, some ex-PWID may have been treated with DAAs. Similarly to the procedure described in the previous subsection, the CHC prevalence among ex-PWID, adjusted for DAAs and spontaneous HCV clearance, can be estimated by

$$\pi_{ex} = \frac{N_{15,79} \rho_{ex} \pi(\text{anti-HCV})_{ex} (1 - \rho_{clear}) - N_{DAA} \rho_{ex|DAA} SVR_{PWID}}{N_{15,79} \rho_{ex}}. \quad (4)$$

Recall that the proportion of ex-PWID among individuals treated with DAAs, i.e. $\rho_{ex|DAA}$, is assumed to be proportional to the corresponding proportions of ex- and non-PWID among CHC-positive individuals, i.e. $\rho_{ex|DAA} = \Pr(\text{Ex-PWID}|\text{CHC}) / \{\Pr(\text{Ex-PWID}|\text{CHC}) + \Pr(\text{Non-PWID}|\text{CHC})\}$, as estimated by our model when the DAA uptake is ignored (Table 2).

## Prevalence of CHC among non-PWID

To estimate the CHC prevalence among non-PWID in a country (after 2010), the ECDC database (European Centre for Disease Prevention and Control (ECDC) 2021) was used. The ECDC group has carefully and critically collected information from relevant studies across the EU/EEA region in cooperation with the focal points of each country. On the basis of the representativeness of each study and other factors (European Centre for Disease Prevention

and Control (ECDC) 2016), a risk of bias score was assigned to each study, with higher values indicating higher-quality studies. When higher-quality studies are available in a country ($\geq 4$), they are used to estimate the overall CHC prevalence in that country among non-PWID. If there are no higher-quality studies, general population estimates with a lower quality (<4) are pooled. If no general population prevalence estimates are available, data on the prevalence of HCV in pregnant women could be used as a prevalence measure. Finally, if data on pregnant women are lacking too, first-time blood donor studies could be an option. However, if the national focal point agrees or recommends, estimates from a neighboring country could be used instead, or we could use any other relevant information provided/suggested by the national focal point.

In Poland, there was 1 study on non-PWID of high quality in 2016, which included CHC data (Rosińska et al. 2017). Thus, individuals treated with DAAs should be removed, with the sustained virologic response ($SVR$) in the general population estimated to be 96.7% (95% CI: 95.4% to 98.1%) (Lampertico et al. 2020). Similarly to the procedure described in the previous subsections, the CHC prevalence among non-PWID, adjusted for DAAs, can be estimated by

$$\pi_{non} = \frac{N_{15,79}\rho_{non}\tilde{\pi}_{non} - N_{DAA}\rho_{non|DAA}SVR}{N_{15,79}\rho_{non}}. \quad (5)$$

where $\tilde{\pi}_{non}$ denotes the CHC prevalence estimate based solely on Rosińska et al. (2017).

As $\rho_{rec}$ and $\rho_{ex}$ are likely to be low, $\pi_{non}$ will be the most critical factor for the estimation of the overall CHC prevalence in the population. To adjust for potential heterogeneity between the CHC prevalence estimates from different studies in the non-PWID population in a country, as a sensitivity analysis, we also apply a random-effect meta-analytic approach to pool the CHC prevalence across studies on non-PWID (Lin and Chu 2020). The random-effect approach is carried out only when at least 3 studies are available in the general population of a certain country, and the estimates are compared with the corresponding estimates from the approach assuming no heterogeneity (similar to a fixed-effect meta-analysis). If the estimates are inconsistent, possible explanations are investigated and discussed. If there is substantial heterogeneity between studies, it would help more to explore the causes; for example, following national focal point suggestions, some studies could be excluded to reduce heterogeneity.

## Sensitivity analysis including migrants from endemic countries

A sensitivity analysis including data for the viremic population among migrants based on the report of the ECDC group (European Centre for Disease Prevention and Control 2016) was carried out. Migrants from endemic countries represent 1.4% of the adult population in Poland (Table 8 in European Centre for Disease Prevention and Control (2016)), with the respective CHC prevalence being equal to $\pi_{mig} = 2.1\%$ (Table 9 in European Centre for Disease Prevention and Control (2016)). The overall CHC prevalence is now estimated by

$$\pi = \pi_{rec}\rho_{rec} + \pi_{ex}\rho_{ex} + \pi_{mig}\rho_{mig} + \pi_{non}\rho_{non},$$

where $\rho_{rec} + \rho_{ex} + \rho_{mig} + \rho_{non} = 1$. A treatment adjustment similar to that described in the previous subsections was also performed.

# RESULTS

The above-mentioned approach was used to estimate the prevalence of CHC in 2019 (considering ages between 15-79 years: 30,478,645).

The aggregated data used by our approach are briefly presented in Table 1. The multi-state Markov model was calibrated on the estimated number of recent PWID in 2012 reported in the 2014 National Report (2013 Data) to the EMCDDA by the Polish REITOX Focal Point. However, in the National Report, the number of current PWIDs was estimated based on research conducted among problem drug users and did not include rare or accidental users. As a result, the ex-PWID number calculated based on the McDonald et al. (2014) method may be underestimated. In Poland, there was 1 study of high quality, which included CHC data (Rosińska et al. 2017). To estimate the CHC prevalence in the general population (primarily non-PWID), we used a fixed-effect meta-analytic approach, with the corresponding results, when information on DAAs is ignored, presented in Table 2. However, based on data provided by the focal point, approximately 35,554 individuals were treated with DAAs from 2016 to 2019 in Poland, with the proportion of ex-PWID and non-PWID among the 35,554 treated individuals assumed to be equal to Pr(Ex-PWID|CHC)/{Pr(Non-PWID|CHC) + Pr(Ex-PWID|CHC)} ≈ 0.93% and Pr(Non-PWID|CHC)/{Pr(Non-PWID|CHC) + Pr(Ex-PWID|CHC)} ≈ 99.07%, respectively (Table 2).

The corresponding results accounting for the DAA uptake are presented in Table 3. The prevalence of recent and ex-PWID was low in Poland (about 0.02% and 0.05%, respectively) corresponding to 7,185 (95% CI: 6,650-7,730) recent PWID and 14,475 (95% CI: 13,800-15,350) ex-PWID in the population. However, the CHC prevalence in these groups was substantial (Table 3) being 50.17% and 6.95%, respectively. This translates to 3,602 (95% CI: 3,235-3,995) and 1,007 (95% CI: 210-1,959) recent and ex-PWID aged 15-79 living with CHC infection in Poland in 2019. The CHC prevalence in the general population was 0.34% (95% CI: 0.26%-0.44%), much lower than that of the high-risk groups. Taking all pieces of information into account, the overall CHC prevalence in Poland in 2019 was equal to 0.36% (95% CI: 0.27%-0.45%), which corresponds to 108,210 (95% CI: 82,261-137,566) individuals aged 15-79 years with CHC infection.

The results from our model including migrants from endemic countries as a separate group are presented in Table 4. However, this analysis comes with possible limitations; that is, including migrants as a separate group is valid only if migrants do not overlap with the remaining groups (recent PWID, ex-PWID, and non-PWID). In any case, the results from Table 4 are quite similar to those reported in Table 3, thus, including or not migrants as a separate non-overlapping group has a negligible effect on the total CHC prevalence estimate in Poland.

# TABLES AND FIGURES

*Table 1. Data (real) contributing to the estimation of CHC prevalence in Poland in 2019.*

| Parameter | Number (95%CI) | Numerator | Denominator | Notes | Year of study |
|---|---|---|---|---|---|
| $\rho_{rec}$ | 7,185 (6,650-7,730) | | | Method based on McDonald et al. | 2019 |
| $\rho_{ex}$ | 14,475 (13,800-15,350) | | | Method based on McDonald et al. | 2019 |
| $\pi(\text{anti-HCV})_{rec}$ | | 406 | 599 | (Rosińska, Sierosławski, and Wiessing 2015) | 2004-2005 |
| $\pi(\text{anti-HCV})_{ever}$ | | 52†† | 171 | EMCDDA database | 2017 |
| $\pi_{non}$ | | 95 | 21,180 | ECDC database (Rosinska et al.); Risk of bias=6† | 2016 |

*Notes*: Although it looks counter-intuitive, **a higher risk of bias score denotes a higher-quality study** (range from 0 to 6); † After excluding individuals who reported having injected drugs [based on Table 1 of Rosińska et al. (2017); missing data were excluded as well]; †† Adjusted to take into account the respondent-driven sampling weights.

*Table 2. Results from the method assuming no heterogeneity in the estimation of the CHC prevalence in the general population from different studies (i.e., similar to a fixed-effect meta-analysis).*

| Parameter | Posterior median | Posterior sd | LB (2.5%) | UB (97.5%) |
|---|---|---|---|---|
| $\rho_{rec}$ (%) | 0.02 | 0 | 0.02 | 0.03 |
| $\rho_{ex}$ (%) | 0.05 | 0 | 0.04 | 0.05 |
| $\pi_{rec}$ (%) | 50.16 | 1.84 | 46.56 | 53.79 |
| $\pi_{ex}$ (%) | 8.99 | 3.88 | 1.96 | 17.13 |
| $\pi_{non}$ (%) | 0.45 | 0.05 | 0.37 | 0.55 |
| $\pi$ (%) | 0.47 | 0.05 | 0.38 | 0.56 |
| Number with CHC | 142,431 | 14,040 | 116,663 | 171,978 |
| Pr(Recent PWID\|CHC) (%) | 2.53 | 0.29 | 2.04 | 3.16 |
| Pr(Ex-PWID\|CHC) (%) | 0.91 | 0.41 | 0.19 | 1.8 |
| Pr(Non-PWID\|CHC) (%) | 96.56 | 0.53 | 95.4 | 97.44 |

*Notes*: Information on DAAs is not taken into account.

*Abbreviations*: $\rho_{rec}$, prevalence of recent PWID (proportion of the population that is recent PWID); $\rho_{ex}$, prevalence of ex-PWID (proportion of the population that is ex-PWID); $\pi_{rec}$, CHC prevalence among recent PWID; $\pi_{ex}$, CHC prevalence among ex-PWID; $\pi_{non}$, CHC prevalence among non-PWID; $\pi$ overall CHC prevalence in Poland; LB, Lower Boundary; UP, Upper Boundary

*Table 3. Results from the method assuming no heterogeneity in the estimation of the CHC prevalence in the general population from different studies (i.e., similar to a fixed-effect meta-analysis).*

| Parameter | Posterior median | Posterior sd | LB (2.5%) | UB (97.5%) |
|---|---|---|---|---|
| $\rho_{rec}$ (%) | 0.02 | 0 | 0.02 | 0.03 |
| $\rho_{ex}$ (%) | 0.05 | 0 | 0.04 | 0.05 |
| $\pi_{rec}$ (%) | 50.17 | 1.85 | 46.63 | 53.83 |
| $\pi_{ex}$ (%) | 6.95 | 3.06 | 1.46 | 13.44 |
| $\pi_{non}$ (%) | 0.34 | 0.05 | 0.26 | 0.44 |
| $\pi$ (%) | 0.36 | 0.05 | 0.27 | 0.45 |
| Number with CHC | 108,210 | 14,172 | 82,261 | 137,566 |
| Pr(Recent PWID\|CHC) (%) | 3.33 | 0.48 | 2.56 | 4.45 |
| Pr(Ex-PWID\|CHC) (%) | 0.93 | 0.43 | 0.19 | 1.86 |
| Pr(Non-PWID\|CHC) (%) | 95.73 | 0.69 | 94.17 | 96.84 |

*Notes*: The number of individuals treated with DAAs is taken into account.

*Abbreviations*: $\rho_{rec}$, prevalence of recent PWID (proportion of the population that is recent PWID); $\rho_{ex}$, prevalence of ex-PWID (proportion of the population that is ex-PWID); $\pi_{rec}$, CHC prevalence among recent PWID; $\pi_{ex}$, CHC prevalence among ex-PWID; $\pi_{non}$, CHC prevalence among non-PWID; $\pi$ overall CHC prevalence in Poland; LB, Lower Boundary; UP, Upper Boundary

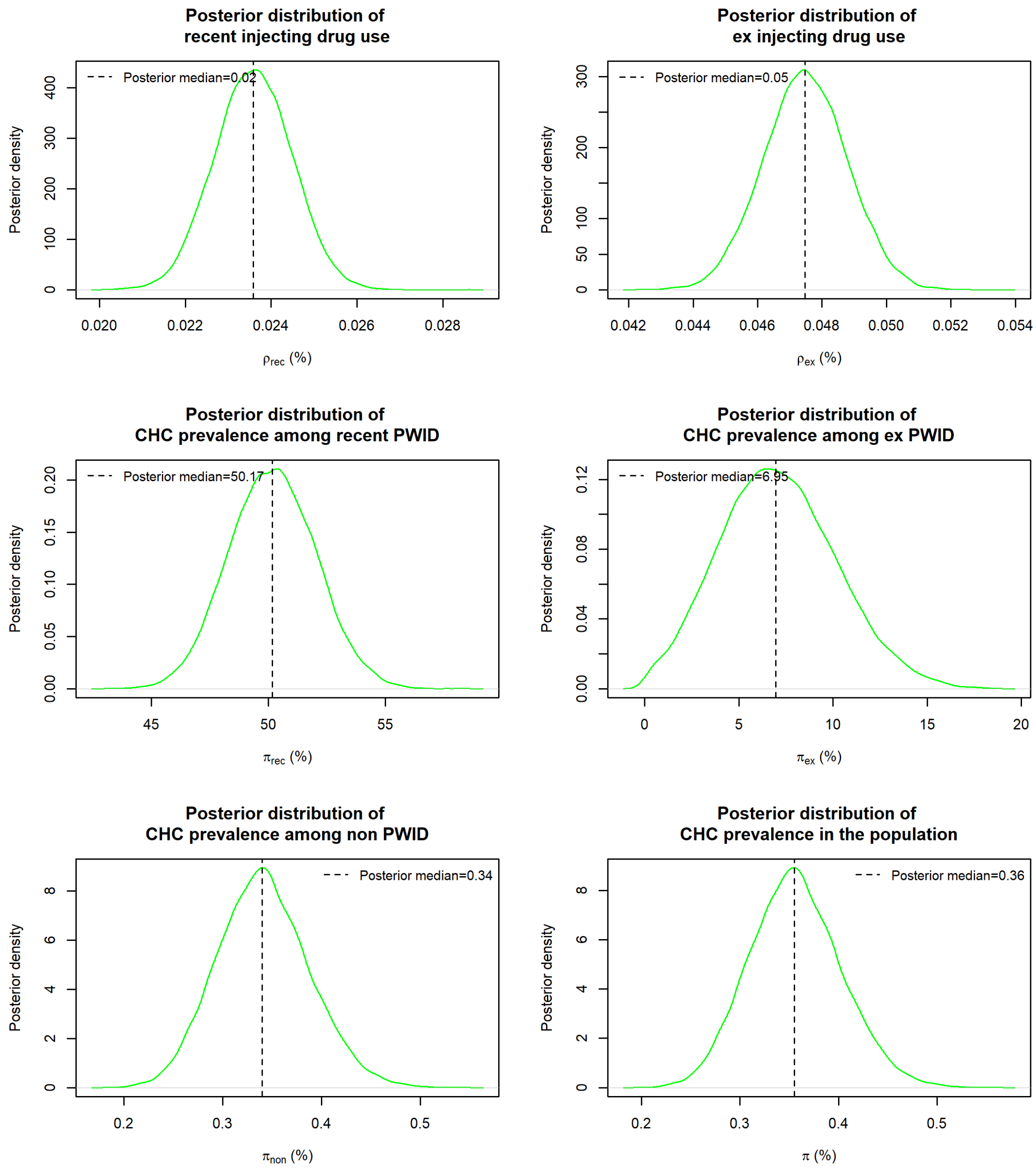


*Figure 1. Posterior distributions of the key model parameters using the approach that assumes no heterogeneity in the estimation of the CHC prevalence in the general population (primarily non-PWID) from different studies (i.e., similar to a fixed-effect meta-analysis), with the number of individuals treated with DAA taken into account.*

*Table 4. Results from the method assuming no heterogeneity in the estimation of the CHC prevalence in the general population (primarily non-PWID) from different studies (i.e., similar to a fixed-effect meta-analysis) including migrants from endemic countries as a separate group.*

| Parameter | Posterior median | Posterior sd | LB (2.5%) | UB (97.5%) |
|---|---|---|---|---|
| $\rho_{rec}$ (%) | 0.02 | 0 | 0.02 | 0.03 |
| $\rho_{ex}$ (%) | 0.05 | 0 | 0.04 | 0.05 |
| $\rho_{mig}$ (%) | 1.4 | 0 | 1.4 | 1.4 |
| $\pi_{rec}$ (%) | 50.13 | 1.84 | 46.54 | 53.76 |
| $\pi_{ex}$ (%) | 7.11 | 3.08 | 1.49 | 13.45 |
| $\pi_{mig}$ (%) | 1.61 | 0.43 | 0.78 | 2.45 |
| $\pi_{non}$ (%) | 0.35 | 0.04 | 0.26 | 0.44 |
| $\pi$ (%) | 0.38 | 0.05 | 0.29 | 0.47 |
| Number with CHC | 115,194 | 13,914 | 89,625 | 143,915 |
| Pr(Recent PWID\|CHC) (%) | 2.41 | 0.26 | 1.96 | 2.97 |
| Pr(Ex-PWID\|CHC) (%) | 0.88 | 0.39 | 0.18 | 1.7 |
| Pr(Mig\|CHC) (%) | 5.98 | 1.59 | 2.99 | 9.21 |
| Pr(Non-PWID\|CHC) (%) | 90.72 | 1.71 | 87.16 | 93.9 |

*Notes*: The number of individuals treated with DAAs is taken into account.

*Abbreviations*: $\rho_{rec}$, prevalence of recent PWID (proportion of the population that is recent PWID); $\rho_{ex}$, prevalence of ex-PWID (proportion of the population that is ex-PWID); $\rho_{mig}$, prevalence of migrants from endemic countries (proportion of the population that belongs to this group); $\pi_{rec}$, CHC prevalence among recent PWID; $\pi_{ex}$, CHC prevalence among ex-PWID; $\pi_{mig}$, CHC prevalence among migrants from endemic countries; $\pi_{non}$, CHC prevalence among non-PWID; $\pi$ overall CHC prevalence in Poland; LB, Lower Boundary; UP, Upper Boundary

# APPENDIX

## Fit of the multi-state Markov model

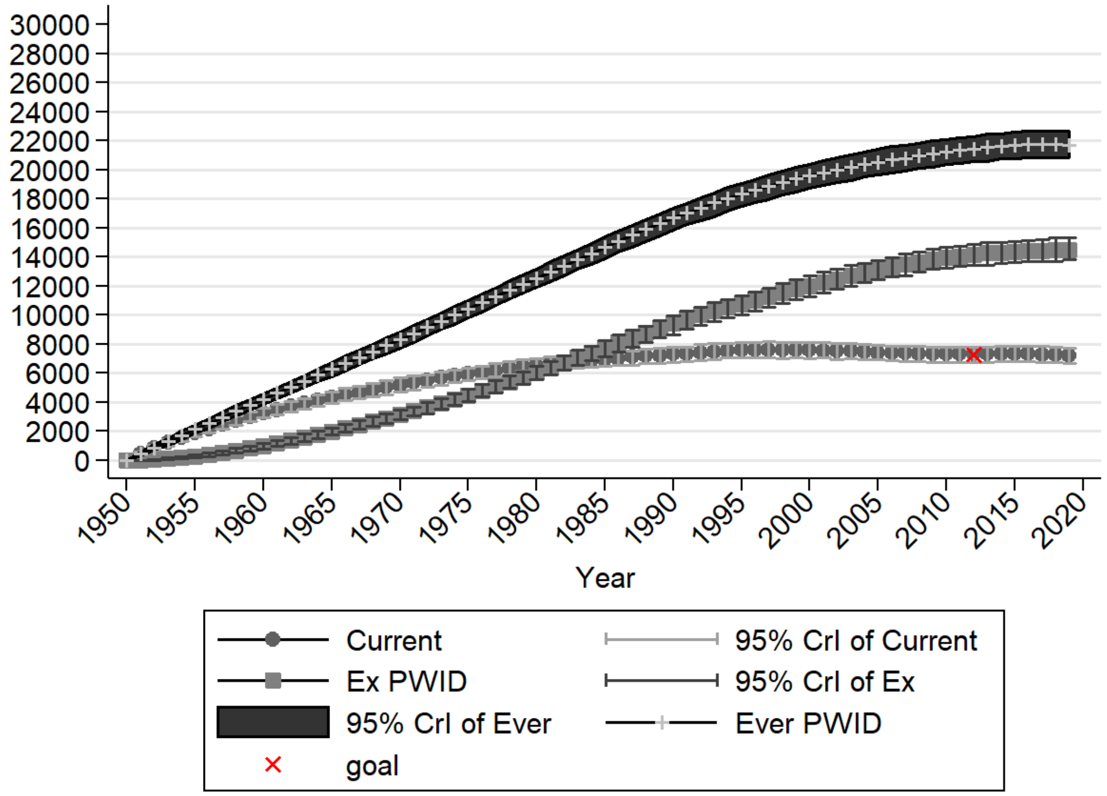


*Figure 2: Model predictions for the numbers of current and ex-PWID (people who inject drugs). The solid black line and shaded grey error bars show the median and 95% credible intervals (95% CrI) for the model projections. For comparison, asterisks indicate the observed country's data.*

## Stan code for Bayesian multiparameter evidence synthesis

```
data {
  int<lower=0> N1579; // Population of 15-79 in `Country`
  int<lower=1> Knon; // Number of studies in the ECDC data for `Country`
  int<lower=1> Kever; // Number of studies in the EMCDDA data for ever us
ers in `Country`
  int<lower=1> Kcur; // Number of studies in the EMCDDA barometer (for HC
V prevalence of PWID) in `Country`
  int<lower=1> NDAA; // Total number of DAAs from 2015 to 2019

  real p_cur_mean; // Prior mean for the prevalence of current use in `Co
untry`
  real<lower=0> p_cur_sd; // Prior sd for the prevalence of current use i
n `Country`

  real p_ex_mean; // Prior mean for the prevalence of ex-use in `Country`
  real<lower=0> p_ex_sd; // Prior sd for the prevalence of ex-use in `Cou
ntry`

  int<lower=0> Nst_hcv_cur[Kcur]; // Number of individuals in the study e
stimating anti-HCV among recent PWID
  int<lower=0> Yst_hcv_cur[Kcur]; // Number of HCV+ in the study estimati
ng anti-HCV among recent PWID

  int<lower=0> Nst_hcv_ever[Kever]; // Number of individuals in the study
estimating anti-HCV among ever IDU
  int<lower=0> Yst_hcv_ever[Kever]; // Number of HCV+ in the study estima
ting anti-HCV among ever PWID

  int<lower=0> Nst_CHC_non[Knon]; // Number of individuals in the study e
stimating CHC among non PWID
  int<lower=0> Yst_CHC_non[Knon]; // Number of HCV+ in the study estimati
```

```
ng CHC among non PWID

  vector<lower=0>[3] alpha; // parameter of the Diriclet prior

  real HCVclear_mean; // Prior mean for the HCV clearance probability
  real<lower=0> HCVclear_sd; // Prior sd for the HCV clearance probabilit
y

  real SVR_mean; // Prior mean for the SVR among non-PWID
  real<lower=0> SVR_sd; // Prior sd for the SVR among non-PWID

  real SVR_PWID_mean; // Prior mean for the SVR among PWID
  real<lower=0> SVR_PWID_sd; // Prior sd for the SVR among PWID
}

// Block defining the original parameters
parameters {
  // The parameters to be sampled
  simplex[3] rho; // Prevalence of the three risk groups
  real<lower=0,upper=1> CHCpi_cur; // P(CHC+|Cur)
  real<lower=0,upper=1> CHCpi_ex; // P(CHC+|Ex)
  real<lower=0,upper=1> CHCpi_non; // P(CHC+|Non)
  real<lower=0,upper=1> SVR; // SVR of DAAs among non-PWID
  real<lower=0,upper=1> SVR_PWID; // SVR of DAAs among PWID

  real<lower=0,upper=1-CHCpi_cur> HCVclear; // Probability of HCV clearan
ce; upper bound = 1-prevalence of chronic HCV
}


transformed parameters {
  // Change scales
```

```
  real<lower=0,upper=1> rho_ever;
  real<lower=0,upper=1> CHCpi_ever;
  real<lower=0,upper=1> pi_ever;
  real<lower=0,upper=1> pi_cur;

  rho_ever = rho[1] + rho[2];
  CHCpi_ever = CHCpi_cur*rho[1]/rho_ever + CHCpi_ex*rho[2]/rho_ever;
  pi_ever = CHCpi_ever/(1-HCVclear);
  pi_cur = CHCpi_cur/(1-HCVclear);
}


// Binomial regression model
model {
  // Priors
  rho ~ dirichlet(alpha);

  //////////////////////////////
  // Likelihood contributions //
  //////////////////////////////

  // Probability of HCV clearance
  HCVclear ~ normal(HCVclear_mean,HCVclear_sd);

  // Prevalence of current use
  rho[1] ~ normal(p_cur_mean,p_cur_sd);

  // Prevalence of ex-use
  rho[2] ~ normal(p_ex_mean,p_ex_sd);

  // Prevalence of chronic HCV among current users
  Yst_hcv_cur ~ binomial(Nst_hcv_cur,pi_cur);
```

```
  // Prevalence of HCV among ever users
  Yst_hcv_ever ~ binomial(Nst_hcv_ever,pi_ever);

  // HCV+ among non
  Yst_CHC_non ~ binomial(Nst_CHC_non,CHCpi_non);

  // SVR of DAAs among non-PWID
  SVR ~ normal(SVR_mean,SVR_sd);

  // SVR of DAAs among PWID
  SVR_PWID ~ normal(SVR_PWID_mean,SVR_PWID_sd);
}


generated quantities {
  // Functions of parameters
  real<lower=0,upper=100> overalCHC;
  real logit_CHCpi_cur;
  real logit_CHCpi_ex;
  real logit_CHCpi_non;
  real logit_rho_cur;
  real logit_rho_ex;
  real logit_rho_non;
  real<lower=0,upper=1> pEverGivenCHC;
  real<lower=0,upper=1> pCurGivenCHC;
  real<lower=0,upper=1> pExGivenCHC;
  real<lower=0,upper=1> pNonGivenCHC;
  real logit_HCVclear;
  real<lower=0> NumberCHC;

  real<lower=0,upper=1> CHCDAApi_non; // P(CHC+|Non) adjusted for DAA
```

```
  //real<lower=0,upper=1> CHCDAApi_cur; // P(CHC+|Cur) adjusted for DAA
  real<lower=0,upper=1> CHCDAApi_ex; // P(CHC+|Ex) adjusted for DAA
  real<lower=0,upper=1> CHCDAApi_ever; // P(CHC+|Ever) adjusted for DAA

  real<lower=0,upper=100> overalCHC_DAA;
  real pEverGivenCHC_DAA;
  real pCurGivenCHC_DAA;
  real pExGivenCHC_DAA;
  real pNonGivenCHC_DAA;
  real<lower=0> NumberCHC_DAA;

  // Overall HCV prevalence
  overalCHC = 100*(rho[1]*CHCpi_cur + rho[2]*CHCpi_ex + rho[3]*CHCpi_non)
;
  pEverGivenCHC = CHCpi_ever*rho_ever/(overalCHC/100);
  pCurGivenCHC = CHCpi_cur*rho[1]/(overalCHC/100);
  pExGivenCHC = CHCpi_ex*rho[2]/(overalCHC/100);
  pNonGivenCHC = CHCpi_non*(1-rho_ever)/(overalCHC/100);
  NumberCHC = round(overalCHC*N1579/100);

  CHCDAApi_non = ( N1579*rho[3]*CHCpi_non - NDAA*pNonGivenCHC*SVR/(pNonGi
venCHC+pExGivenCHC))/(N1579*rho[3]);
  //CHCDAApi_cur = ( N1579*rho[1]*CHCpi_cur - NDAA*pCurGivenCHC*SVR_PWID_
mean)/(N1579*rho[1]);
  CHCDAApi_ex = ( N1579*rho[2]*CHCpi_ex - NDAA*pExGivenCHC*SVR_PWID_mean/
(pNonGivenCHC+pExGivenCHC))/(N1579*rho[2]);
  CHCDAApi_ever = CHCpi_cur*rho[1]/rho_ever + CHCDAApi_ex*rho[2]/rho_ever
;

  overalCHC_DAA = 100*(rho[1]*CHCpi_cur + rho[2]*CHCDAApi_ex +  rho[3]*CH
CDAApi_non);
  pEverGivenCHC_DAA = CHCDAApi_ever*rho_ever/(overalCHC_DAA/100);
```

```
  pCurGivenCHC_DAA = CHCpi_cur*rho[1]/(overalCHC_DAA/100);
  pExGivenCHC_DAA = CHCDAApi_ex*rho[2]/(overalCHC_DAA/100);
  pNonGivenCHC_DAA = CHCDAApi_non*(1-rho_ever)/(overalCHC_DAA/100);
  NumberCHC_DAA = round(overalCHC_DAA*N1579/100);

  logit_rho_cur = logit(rho[1]);
  logit_rho_ex = logit(rho[2]);
  logit_rho_non = logit(rho[3]);
  logit_CHCpi_cur = logit(CHCpi_cur);
  logit_CHCpi_ex = logit(CHCpi_ex);
  logit_CHCpi_non = logit(CHCpi_non);
  logit_HCVclear = logit(HCVclear);
}
```

### Multi-state Markov model

```
#include <stdio.h>
#include <stdlib.h>
#include <string.h>
#include <math.h>
#include <time.h>

#define loops 100
int populationPerYearAndAge[2021][66];
double deathRatePerAge[66];
int totalPopulationPerYear[2021];

FILE *F_DeathRate=fopen("`Country`-deathRates.txt","r");
FILE *F_Population=fopen("`Country`-population.txt","r");
FILE *out;

double pa_start = 0.00012;                    // Setting Variable
double pa_step  = 0.000002;                   // Setting Variable
double pa_stop  = 0.000123;                   // Setting Variable

double pa;
double pg = 1.0/11.7;    // Assumed average active injecting career of  11
.7 years
double pk = 0.004*12.0;     // Relapse rate of 0.004/month

struct people{
    int age;
    int state;
};

char filename[100];
struct people person[50000000];
```

```
long int totalPersons=0;

void setPopulationAge()
{
    for( int j=0; j<71; j++){
        for( int i=0; i<51; i++){
            fscanf(F_Population,"%d",&populationPerYearAndAge[1950+j][15+
i]);
        }
    }
}

void setDeathRate()
{
    for( int i=0; i<51; i++){
        fscanf(F_DeathRate,"%lf",&deathRatePerAge[15+i*5]);
        for( int j=0; j<5; j++){
            deathRatePerAge[15+i*5+j] = deathRatePerAge[15+i*5];
        }
    }
}

void getTotalPopulationPerAge()
{
    for( int j=0; j<71; j++){
        for( int i=0; i<51; i++){
            totalPopulationPerYear[1950+j] += populationPerYearAndAge[195
0+j][15+i];
        }
    }
}
```

```
void printTotalPopulationPerAge()
{
    printf("---- - ----------\n");
    printf("Year - Population\n");
    printf("---- - ----------\n");
    for( int i=1950; i<2021; i++){
        printf("%d - %d\n",i,totalPopulationPerYear[i]);
    }
}

void printDeathRatePerAge()
{
    printf("--- - -----\n");
    printf("Age - Rate\n");
    printf("--- - -----\n");
    for( int i=15; i<65; i++){
        printf("%d - %lf\n",i,deathRatePerAge[i]);
    }
}

void printTotalPersonPerState( int year)
{
    int count[4]= {0,0,0,0};
    for( int i=0; i<totalPersons; i++){
        count[person[i].state]++;
    }
    //printf("%d\t%ld\t%ld\t%ld\t%ld\n",year,count[0],count[1],count[2],c
ount[3]);
    if(year==2014)
        printf("%d\t%ld\t%ld\t%ld\t%ld\t%lf\n",year,count[0],count[1],cou
nt[2],count[3],pa);
    if( year>2009){
```

```
        fprintf(out, "%d\t%ld\t%ld\t%ld\t%ld\n",year,count[0],count[1],co
unt[2],count[3]);
    }
}

void initializePopulation()
{
    int cnt=0;
    for( int j=15; j<65; j++){
        for( int i=0; i<populationPerYearAndAge[1950][j]*100; i++){
            person[cnt].age=j;
            person[cnt].state=0;
            cnt++;
        }
    }
    totalPersons = cnt;
}

bool checkRate(double rate)
{
    if (rate <0.001){
        double k = rand()/(1.00+RAND_MAX);
        if(k<0.001){
            rate = rate*1000;
        }
        else{
            return false;
        }
    }
    double k = rand()/(1.00+RAND_MAX);
    if ( k < rate )
        return true;
```

```
    else
        return false;
}

void changeStatusAndAge()
{
    for( int i=0; i<totalPersons; i++){
        // change state
        double k = rand()/(1.00+RAND_MAX);
        if( person[i].state==0){
            if ( checkRate(pa) &&  person[i].age<40){
                person[i].state=1;
            }
        }
        else if( person[i].state==1){
            if ( k < pg ){
                person[i].state=2;
            }
        }
        else if( person[i].state==2){
            if ( k < pk ){
                person[i].state=1;
            }
        }
        // After their 49th year, all active PWID are assumed to cease in
jecting
        if(person[i].age>49 && person[i].state==1){
            person[i].state=2;
        }
        // After their 64th year, remove
        if(person[i].age>64){
            person[i].state=3;
```

```
        }
        // death rate depending on age and state
        k = rand()/(1.00+RAND_MAX);
        if( person[i].state==0){
            if ( k < deathRatePerAge[person[i].age] ){
                person[i].state=3;
            }
        }
        else if( person[i].state==1){
            if ( k < deathRatePerAge[person[i].age] * 13.0 ){
                person[i].state=3;
            }
        }
        else if( person[i].state==2){
            if ( k < deathRatePerAge[person[i].age] ){
                person[i].state=3;
            }
        }
        // increase age one year
        person[i].age++;
    }
}

void addNewPersons(int year)
{
    // add new person [15 years old and non PWID]
    int cnt=totalPersons;
    for( int i=0; i<populationPerYearAndAge[year+1][15]*100; i++){
// 100 instead of 1000 for the population to reduce execution time
        person[cnt].age=15;
        person[cnt].state=0;
        cnt++;
```

```
    }
    // increase the total number of population
    totalPersons = cnt;
}
int main()
{
    srand(time(NULL));

    setPopulationAge();
    setDeathRate();

    getTotalPopulationPerAge();
    pa = pa_start;
    while ( pa < pa_stop){

        snprintf(filename, 100, "result_%lf.txt",pa);
        out=fopen(filename,"w");

        for( int iter=0; iter<loops ;iter++){
            initializePopulation();
            for( int year=1950; year<2020; year++){
                printTotalPersonPerState(year);
                changeStatusAndAge();
                addNewPersons(year);
            }
        }

        fclose(out);
        pa = pa + pa_step;
    }
```

```
}
```

# REFERENCES

European Centre for Disease Prevention and Control. 2016. "Epidemiological Assessment of Hepatitis b and c Among Migrants in the EU/EEA." https://www.ecdc.europa.eu/sites/default/files/media/en/publications/Publications/epidemiological-assessment-hepatitis-B-and-C-among-migrants-EU-EEA.pdf.

European Centre for Disease Prevention and Control (ECDC). 2016. "Systematic Review on Hepatitis b and c Prevalence in the EU/EEA." https://www.ecdc.europa.eu/sites/default/files/media/en/publications/Publications/systematic-review-hepatitis-B-C-prevalence.pdf.

———. 2021. "Hepatitis c Prevalence Database." https://www.ecdc.europa.eu/en/all-topics-z/hepatitis-c/tools/hepatitis-c-prevalence-database.

Graf, Christiana, Marcus M Mücke, Georg Dultz, Kai-Henrik Peiffer, Alica Kubesch, Patrick Ingiliz, Stefan Zeuzem, Eva Herrmann, and Johannes Vermehren. 2019. "Efficacy of Direct-acting Antivirals for Chronic Hepatitis C Virus Infection in People Who Inject Drugs or Receive Opioid Substitution Therapy: A Systematic Review and Meta-analysis." *Clinical Infectious Diseases* 70 (11): 2355–65. https://doi.org/10.1093/cid/ciz696.

Grebely, Jason, Sarah Larney, Amy Peacock, Samantha Colledge, Janni Leung, Matthew Hickman, Peter Vickerman, et al. 2019. "Global, Regional, and Country-Level Estimates of Hepatitis c Infection Among People Who Have Recently Injected Drugs." *Addiction* 114 (1): 150–66. https://doi.org/https://doi.org/10.1111/add.14393.

Hines, Lindsey A., Adam Trickey, Janni Leung, Sarah Larney, Amy Peacock, Louisa Degenhardt, Samantha Colledge, et al. 2020. "Associations Between National Development Indicators and the Age Profile of People Who Inject Drugs: Results from a Global Systematic Review and Meta-Analysis." *The Lancet Global Health* 8 (1): e76–91. https://doi.org/10.1016/S2214-109X(19)30462-0.

Lampertico, Pietro, Jose A. Carrión, Michael Curry, Juan Turnes, Markus Cornberg, Francesco Negro, Ashley Brown, et al. 2020. "Real-World Effectiveness and Safety of Glecaprevir/Pibrentasvir for the Treatment of Patients with Chronic HCV Infection:

A meta-Analysis." *Journal of Hepatology* 72 (6): 1112–21. https://doi.org/10.1016/j.jhep.2020.01.025.

Lin, Lifeng, and Haitao Chu. 2020. "Meta-Analysis of Proportions Using Generalized Linear Mixed Models." *Epidemiology* 31 (5). https://journals.lww.com/epidem/Fulltext/2020/09000/Meta_analysis_of_Proportions_Using_Generalized.16.aspx.

McDonald, Scott A., Rosmawati Mohamed, Maznah Dahlui, Herlianna Naning, and Adeeba Kamarulzaman. 2014. "Bridging the Data Gaps in the Epidemiology of Hepatitis c Virus Infection in Malaysia Using Multi-Parameter Evidence Synthesis." *BMC Infectious Diseases* 14 (1): 564. https://doi.org/10.1186/s12879-014-0564-6.

Micallef, J. M., J. M. Kaldor, and G. J. Dore. 2006. "Spontaneous Viral Clearance Following Acute Hepatitis c Infection: A Systematic Review of Longitudinal Studies." *Journal of Viral Hepatitis* 13 (1): 34–41. https://doi.org/https://doi.org/10.1111/j.1365-2893.2005.00651.x.

Presanis, Anne M., O. Noel Gill, Timothy R. Chadborn, Caterina Hill, Vivian Hope, Louise Logan, Brian D. Rice, Valerie C. Delpech, A. E. Ades, and Daniela De Angelis. 2010. "Insights into the Rise in HIV Infections, 2001 to 2008: A Bayesian Synthesis of Prevalence Evidence." *AIDS* 24 (18). https://journals.lww.com/aidsonline/Fulltext/2010/11270/Insights_into_the_rise_in_HIV_infections,_2001_to.12.aspx.

Rosińska, Magdalena, Natalia Parda, Agnieszka Kołakowska, Paulina Godzik, Karolina Zakrzewska, Kazimierz Madaliński, Andrzej Zieliński, Anna Boguradzka, Rafał Gierczyński, and Małgorzata Stępień. 2017. "Factors Associated with Hepatitis c Prevalence Differ by the Stage of Liver Fibrosis: A Cross-Sectional Study in the General Population in Poland, 2012-2016." *PLOS ONE* 12 (9): e0185055. https://doi.org/10.1371/journal.pone.0185055.

Rosińska, Magdalena, Janusz Sierosławski, and Lucas Wiessing. 2015. "High Regional Variability of HIV, HCV and Injecting Risks Among People Who Inject Drugs in Poland: Comparing a Cross-Sectional Bio-Behavioural Study with Case-Based Surveillance." *BMC Infectious Diseases* 15 (1): 83. https://doi.org/10.1186/s12879-015-0828-9.

Sweeting, M. J., D. De Angelis, M. Hickman, and A. E. Ades. 2008. "Estimating hepatitis C prevalence in England and Wales by synthesizing evidence from multiple data sources. Assessing data conflict and model fit." *Biostatistics* 9 (4): 715–34. https://doi.org/10.1093/biostatistics/kxn004.

Tan, Sarah, Susanna Makela, Daliah Heller, Kevin Konty, Sharon Balter, Tian Zheng, and James H. Stark. 2018. "A Bayesian Evidence Synthesis Approach to Estimate Disease Prevalence in Hard-to-Reach Populations: Hepatitis c in New York City." *Epidemics* 23: 96–109. https://doi.org/https://doi.org/10.1016/j.epidem.2018.01.002.

Thein, Hla-Hla, Qilong Yi, Gregory J. Dore, and Murray D. Krahn. 2008. "Estimation of Stage-Specific Fibrosis Progression Rates in Chronic Hepatitis c Virus Infection: A Meta-Analysis and Meta-Regression." *Hepatology* 48 (2): 418–31. https://doi.org/https://doi.org/10.1002/hep.22375.

Veen, Maaike G. van, Anne M. Presanis, Stefano Conti, Maria Xiridou, Annemarie R. Stengaard, Martin C. Donoghoe, Ard I. van Sighem, Marianne A. van der Sande, and Daniela De Angelis. 2011. "National Estimate of HIV Prevalence in the Netherlands: Comparison and Applicability of Different Estimation Tools." *AIDS* 25 (2). https://journals.lww.com/aidsonline/Fulltext/2011/01140/National_estimate_of_HIV_prevalence_in_the.14.aspx.

# Estimating the prevalence of chronic hepatitis C infection (CHC) in Portugal using Bayesian multiparameter evidence synthesis

10/05/2023

## Table of Contents


Introduction .......... 2

Methods .......... 2

Prevalence of ex-PWID .......... 3

Prevalence of recent PWID .......... 3

Prevalence of CHC among recent PWID .......... 4

Prevalence of CHC among ex-PWID .......... 4

Prevalence of CHC among non-PWID .......... 5

Sensitivity analysis including migrants from endemic countries .......... 6

Results .......... 6

Tables and Figures .......... 8

Appendix .......... 12

Fit of the multi-state Markov model .......... 12

Stan code for Bayesian multiparameter evidence synthesis .......... 13

Multi-state Markov model .......... 18

References .......... 26

# INTRODUCTION

Patients with chronic hepatitis C (HCV) infection often remain asymptomatic for decades and even for life, but a proportion of them develop active chronic hepatitis, which is a progressive disease (Thein et al. 2008). Because of the largely asymptomatic nature of HCV infection, notification data reflect national screening and testing practices and do not give accurate insights into the prevalence of HCV infection. Although there are studies that have estimated the prevalence of HCV antibodies (anti-HCV) in specific groups such as the general population, first-time blood donors, or people who inject drugs (PWID) at drug treatment centers, those alone cannot be combined to produce national estimates unless some additional information regarding the composition of each HCV risk group and its prevalence in the population are known (Sweeting et al. 2008; McDonald et al. 2014; Tan et al. 2018).

# METHODS

Bayesian multiparameter evidence synthesis (BMES) has been a popular approach to formally estimate anti-HCV or HIV prevalence (Sweeting et al. 2008; McDonald et al. 2014; Tan et al. 2018; Presanis et al. 2010; Veen et al. 2011). In this project, we extend this method to estimating the CHC prevalence, with the population (15-79 years) split into three main non-overlapping risk groups, i.e., recent PWID (those who have injected in the last year), ex-PWID, and non-PWID. A unified model is assumed including parameters associated with the CHC prevalence of recent, ex-PWID, and non-PWID (denoted by $\pi_{rec}$, $\pi_{ex}$, and $\pi_{non}$, respectively) and the prevalence of these risk groups in the population (denoted by $\rho_{rec}$, $\rho_{ex}$, and $\rho_{non}$, respectively; $\rho_{rec} + \rho_{ex} + \rho_{non} = 1$). To estimate the CHC prevalence in the whole population, $\pi$, we used the formula:

$$\pi = \pi_{rec}\rho_{rec} + \pi_{ex}\rho_{ex} + \pi_{non}\rho_{non} \quad (1)$$

Bayesian synthesis considers all available data, allowing for an evidence-based estimation of CHC prevalence in the population, with inherent uncertainty properly accounted for. Our aim is to estimate the CHC prevalence for the total (15-79) population of Portugal in 2019 using sources of information over the last decade (2010-2019).

## Prevalence of ex-PWID

Data on the prevalence of ex-PWID are sparse and generally often unreliable. To overcome this limitation, we apply the method proposed by McDonald et al. (2014), which requires simulating a multi-state Markov model representing the non-PWID, recent PWID, and ex-PWID. In the multi-state Markov model, the simulation starts in 1950 and examines the population aged 15-79 years old. A certain number of 15-year-old individuals enter the simulation process each year. These numbers of 15-year-old individuals are obtained from the United Nations Statistic Division UNSD, being different for each country. The ageing of the population is also taken into account. Country-specific overall mortality data are retrieved from the Life Tables of the World Health Organization WHO. The duration of injecting career is also country-specific and obtained from the paper of Trickey et al. (2019).

To estimate the number of ex-PWID, the model can be calibrated on the number of recent PWID provided in the EMCDDA barometer or the EMCDDA statistical bulletin (the code of the Markov model can be found in the Appendix). If there are no available data in the barometer, estimates from the systematic review of Grebely et al. (2019) could be used. Alternatively, if the national focal points suggest or provide different and updated or more accurate data for calibration purposes, we will consider their advice and adjust the model accordingly.

After applying the model for Portugal, we compute the number (and the corresponding Confidence Interval - CI) of ex-PWID in 2019. The number of ex-PWID is then divided by the population size (15-79 years) in the same year, and a Normal distribution, constrained such that $\rho_{ex} \in (0,1)$, is assumed for $\rho_{ex}$. The standard deviation of this distribution is specified to approximately correspond to the respective CI, i.e. $(UB_{\rho_{ex}} - LB_{\rho_{ex}})/(2 \times 1.96)$, where $UB_{\rho_{ex}}$ and $LB_{\rho_{ex}}$ denote the upper and lower limit of the CI.

## Prevalence of recent PWID

The number of recent PWID in Portugal in 2019 is also projected by the multi-state Markov model described in the previous subsection. To estimate $\rho_{rec}$, the number of recent PWID is divided by the population size (15-79 years). Similar to the previous subsection, a Normal distribution, constrained such that $\rho_{rec} \in (0,1)$, is assumed for $\rho_{rec}$, with the standard deviation specified to correspond to the CI obtained from the multi-state Markov model.

## Prevalence of CHC among recent PWID

The prevalence (and the 95% CI) of CHC among recent PWID can be informed from studies in the EMCDDA statistical bulletin. If information on the CHC prevalence is available, the Binomial distribution in the model to inform $\pi_{rec}$ was used. However, as these EMCDDA data typically refer to the anti-HCV prevalence and not to the CHC prevalence, they could not be used directly. This issue is addressed by noting that spontaneous viral clearance occurs in approximately one in four people with acute hepatitis C (Micallef, Kaldor, and Dore 2006). To account for the variability of the HCV clearance probabilities, we used the result reported in Micallef, Kaldor, and Dore (2006), i.e., the proportion of HCV clearance (denoted by $\rho_{clear}$) is equal to 0.26 (95% CI 0.22–0.29). Thus, estimates of the CHC prevalence among recent PWID ($\pi_{rec}$) can be obtained using the formula $\pi_{rec} = \pi(\text{anti-HCV})_{rec}(1 - \rho_{clear})$, where $\pi(\text{anti-HCV})_{rec}$ denotes the anti-HCV prevalence among recent PWID.

Apart from spontaneous clearance, some people may have been treated with direct acting antivirals (DAAs). Since treatment data may be weak and unstandardised or unavailable for most EU/EEA countries, we have not formally taken treatment into account.

If information on the HCV prevalence (CHC or anti-HCV) among recent PWID in the EMCDDA database is not available, the required information is obtained from the paper of Grebely et al. (2019). Currently, CHC prevalence estimates from the paper of Grebely et al. (2019) were used. If the national focal point recommends updated formal estimates, the model input could be adjusted accordingly.

## Prevalence of CHC among ex-PWID

Information on CHC prevalence among ex-PWID (i.e., $\pi_{ex}$) is difficult to obtain directly. To overcome this, nationwide data on HCV prevalence among ever users through the EMCDDA database was utilized. However, if these data refer to the anti-HCV prevalence, they are adjusted according to the procedure described in the previous subsection, i.e. $\pi_{ever} = \pi(\text{anti-HCV})_{ever}(1 - \rho_{clear})$, where $\pi(\text{anti-HCV})_{ever}$ denotes the anti-HCV prevalence among ever PWID. In any case, if an estimate of $\pi_{ever}$ is available, $\pi_{ex}$ can be indirectly estimated since $\pi_{ever}$ is equal to a weighted average of $\pi_{rec}$ and $\pi_{ex}$, as shown by the following formula

$$\pi_{ever} = \frac{\rho_{rec}}{\rho_{rec} + \rho_{ex}} \pi_{rec} + \frac{\rho_{ex}}{\rho_{rec} + \rho_{ex}} \pi_{ex}, \quad (2)$$

or, equivalently,

$$\pi_{ex} = \left(\pi_{ever} - \frac{\rho_{rec}}{\rho_{rec} + \rho_{ex}} \pi_{rec}\right) \times \frac{\rho_{rec} + \rho_{ex}}{\rho_{ex}}, \quad (3)$$

Currently, national anti-HCV prevalence data from ever PWID in 2019, available in the EMCDDA statistical bulletin, were used. In any case, the model could be updated with any other relevant study/information suggested by the national focal point.

## Prevalence of CHC among non-PWID

To estimate the CHC prevalence among non-PWID in a country (after 2010), the ECDC database (European Centre for Disease Prevention and Control (ECDC) 2021) was used. The ECDC group has carefully and critically collected information from relevant studies across the EU/EEA region in cooperation with the focal points of each country. On the basis of the representativeness of each study and other factors (European Centre for Disease Prevention and Control (ECDC) 2016), a risk of bias score was assigned to each study, with higher values indicating higher-quality studies. When higher-quality studies are available in a country (≥ 4), they are used to estimate the overall CHC prevalence in that country among non-PWID. If there are no higher-quality studies, general population estimates with a lower quality (<4) are pooled. If no general population prevalence estimates are available, data on the prevalence of HCV in pregnant women could be used as a prevalence measure. Finally, if data on pregnant women are lacking too, first-time blood donor studies could be an option. However, if the national focal point agrees or recommends, estimates from a neighboring country could be used instead, or we could use any other relevant information provided/suggested by the national focal point.

We directly use CHC prevalence data from studies in the general population, if available. If the data in the general population refer to anti-HCV prevalence (without any data on the viremic population), we adjust the estimates to get CHC prevalence based on the spontaneous HCV clearance estimate of 26%, described in the previous subsection.

As $\rho_{rec}$ and $\rho_{ex}$ are likely to be low, $\pi_{non}$ will be the most critical factor for the estimation of the overall CHC prevalence in the population. To adjust for potential heterogeneity between the CHC prevalence estimates from different studies in the non-PWID population in a country, as a sensitivity analysis, we also apply a random-effect meta-analytic approach to pool the CHC prevalence across studies on non-PWID (Lin and Chu 2020). The random-effect approach is carried out only when at least 3 studies are available in the general population of a certain country, and the estimates are compared with the corresponding estimates from the approach assuming no heterogeneity (similar to a fixed-effect meta-analysis). If the estimates are inconsistent, possible explanations are investigated and discussed. If there is substantial heterogeneity between studies, it would help more to explore the causes; for example, following national focal point suggestions, some studies could be excluded to reduce heterogeneity.

## Sensitivity analysis including migrants from endemic countries

A sensitivity analysis including data for the viremic population among migrants based on the report of the ECDC group (European Centre for Disease Prevention and Control 2016) was carried out. Migrants from endemic countries represent 7.1% of the adult population in Portugal (Table 8 in European Centre for Disease Prevention and Control (2016)), with the respective CHC prevalence being equal to $\pi_{mig} = 2.1\%$ (Table 9 in European Centre for Disease Prevention and Control (2016)). The overall CHC prevalence is now estimated by

$$\pi = \rho_{rec}\pi_{rec} + \pi_{ex}\rho_{ex} + \pi_{mig}\rho_{mig} + \pi_{non}\rho_{non},$$

where $\rho_{rec} + \rho_{ex} + \rho_{mig} + \rho_{non} = 1$.

# RESULTS

The above-mentioned approach was used to estimate the prevalence of CHC in 2019 (considering ages between 15-79 years: 8,207,595).

The aggregated data used by our approach are briefly presented in Table 1. The multi-state Markov model to estimate the prevalence of recent and ex-PWID was calibrated on the estimates reported in the EMCDDA barometer. In Portugal, there was 1 study in the general population of high quality, which included CHC data (Carvalhana et al. 2016). To estimate the CHC prevalence in the general population (primarily non-PWID), we used a fixed-effect meta-

analytic approach, with the corresponding results presented in Table 2. The prevalence of recent and ex-PWID was low in Portugal (about 0.15% and 0.41%, respectively) corresponding to 12,500 (95% CI: 11,650-13,150) recent PWID and 33,950 (95% CI: 32,850-35,100) ex-PWID in the population. However, the CHC prevalence in these groups was substantial (Table 2) being 65.74% and 57.27%, respectively. This translates to 8,210 (95% CI: 7,293-9,149) and 19,445 (95% CI: 17,323-21,660) recent and ex-PWID aged 15-79 living with CHC infection in Portugal in 2019. The CHC prevalence in the general population was 0.16% (95% CI: 0.04%-0.45%), much lower than that of the high-risk groups. Taking all pieces of information into account, the overall CHC prevalence in Portugal in 2019 was equal to 0.5% (95% CI: 0.37%-0.78%), which corresponds to 41,161 (95% CI: 30,370-64,216) individuals aged 15-79 years with CHC infection.

The results from our model including migrants from endemic countries as a separate group are presented in Table 3. However, this analysis comes with possible limitations; that is, including migrants as a separate group is valid only if migrants do not overlap with the remaining groups (recent PWID, ex-PWID, and non-PWID). Thus, if migrants do participate proportionally in the study(ies) in the general population, this analysis may result in biased overall CHC estimates (most probably in higher CHC prevalence estimates). However, if the national focal points consider that including migrants as a separate group is valid, we could consider results in Table 3 as the main analysis.

# TABLES AND FIGURES

*Table 1. Data (real) contributing to the estimation of CHC prevalence in Portugal in 2019.*

| Parameter | Estimate (95%CI) | Number (95%CI) | Numerator | Denominator | Notes | Year of study |
|---|---|---|---|---|---|---|
| $\rho_{rec}$ | | 12,500 (11,650-13,150) | | | Method based on McDonald et al. | 2019 |
| $\rho_{ex}$ | | 33,950 (32,850-35,100) | | | Method based on McDonald et al. | 2019 |
| $\pi_{rec}$ | 65.8% (59.1%-72.2%) | | | | Grebely et al. | 2013-2016 |
| $\pi(\text{anti-HCV})_{ever}$ | | | 262 | 325 | EMCDDA database | 2019 |
| $\pi_{non}$ | | | 2 | 1,627 | ECDC database (Carvalhana et al.); Risk of bias=5 | 2014 |

*Notes*: Higher risk of bias score denotes a higher-quality study (range from 0 to 6);

*Table 2. Results from the method assuming no heterogeneity in the estimation of the CHC prevalence in the general population from different studies (i.e., similar to a fixed-effect meta-analysis).*

| Parameter | Posterior median | Posterior sd | LB (2.5%) | UB (97.5%) |
|---|---|---|---|---|
| $\rho_{rec}$ (%) | 0.15 | 0 | 0.14 | 0.16 |
| $\rho_{ex}$ (%) | 0.41 | 0.01 | 0.4 | 0.43 |
| $\pi_{rec}$ (%) | 65.74 | 3.22 | 59.31 | 71.87 |
| $\pi_{ex}$ (%) | 57.27 | 3.13 | 51.26 | 63.49 |
| $\pi_{non}$ (%) | 0.16 | 0.11 | 0.04 | 0.45 |
| $\pi$ (%) | 0.5 | 0.11 | 0.37 | 0.78 |
| Number with CHC | 41,161 | 8,796 | 30,370 | 64,216 |
| Pr(Ever PWID\|CHC) (%) | 67.35 | 12.31 | 43.13 | 90.12 |
| Pr(Non-PWID\|CHC) (%) | 32.65 | 12.31 | 9.88 | 56.87 |

*Notes*: $\rho_{rec}$, prevalence of recent PWID; $\rho_{ex}$, prevalence of ex-PWID; $\pi_{rec}$, CHC prevalence among recent PWID; $\pi_{ex}$, CHC prevalence among ex-PWID; $\pi_{non}$, CHC prevalence among non-PWID; $\pi$ overall CHC prevalence in Portugal; LB, Lower Boundary; UP, Upper Boundary

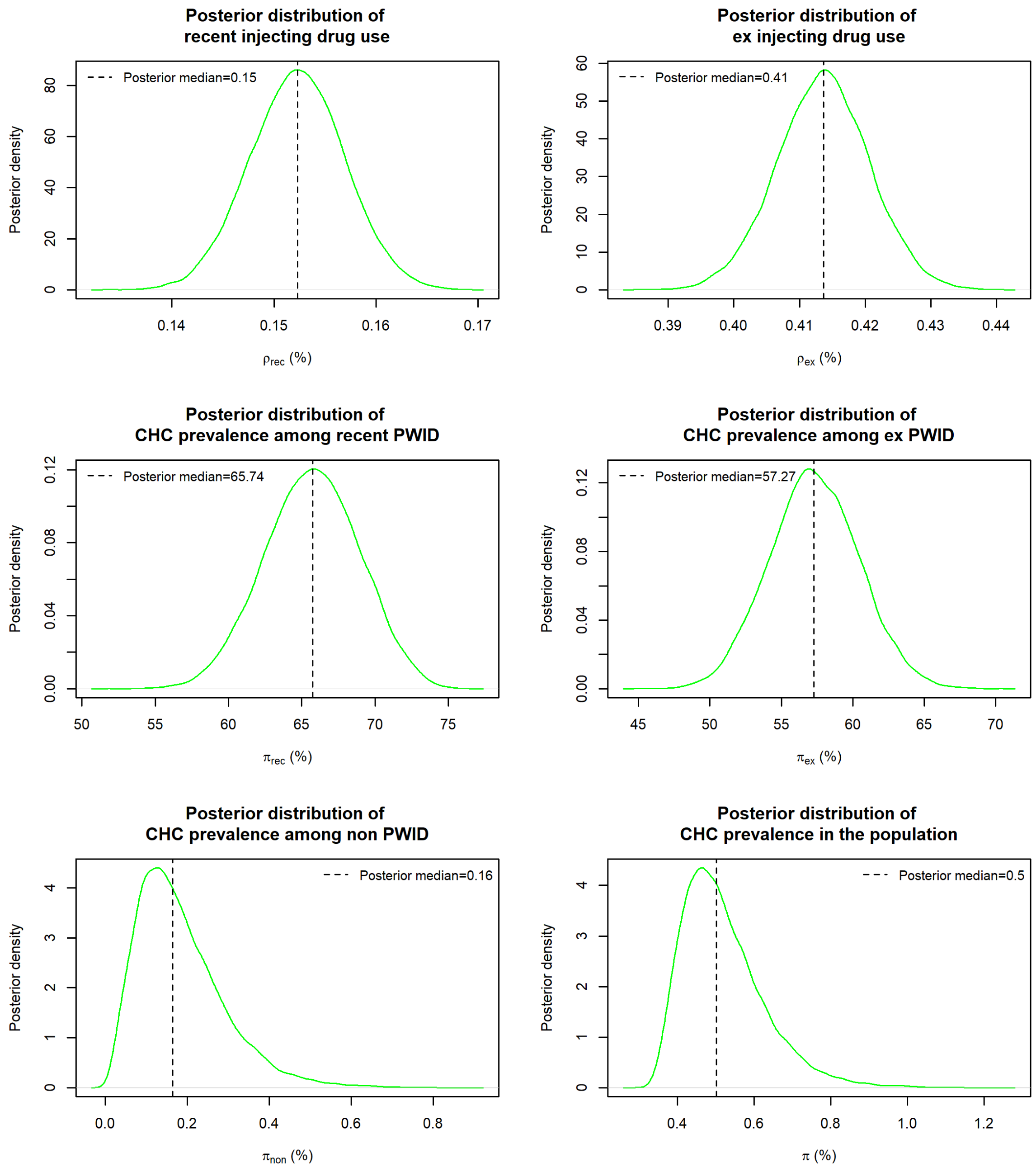


*Figure 1. Posterior distributions of the key model parameters using the approach that assumes no heterogeneity in the estimation of the CHC prevalence in the general population (primarily non-PWID) from different studies (i.e., similar to a fixed-effect meta-analysis).*

*Table 3. Results from the method assuming no heterogeneity in the estimation of the CHC prevalence in the general population (primarily non-PWID) from different studies (i.e., similar to a fixed-effect meta-analysis) including migrants from endemic countries as a separate group.*

| Parameter | Posterior median | Posterior sd | LB (2.5%) | UB (97.5%) |
|---|---|---|---|---|
| $\rho_{rec}$ (%) | 0.15 | 0 | 0.14 | 0.16 |
| $\rho_{ex}$ (%) | 0.41 | 0.01 | 0.4 | 0.43 |
| $\rho_{mig}$ (%) | 7.1 | 0 | 7.1 | 7.1 |
| $\pi_{rec}$ (%) | 65.74 | 3.26 | 59.24 | 71.88 |
| $\pi_{ex}$ (%) | 57.28 | 3.18 | 51.11 | 63.54 |
| $\pi_{mig}$ (%) | 2.1 | 0.67 | 0.8 | 3.42 |
| $\pi_{non}$ (%) | 0.16 | 0.11 | 0.04 | 0.45 |
| $\pi$ (%) | 0.64 | 0.11 | 0.48 | 0.91 |
| Number with CHC | 52,741 | 9,007 | 39,492 | 74,982 |
| Pr(Ever PWID\|CHC) (%) | 52.6 | 8.39 | 36.82 | 69.58 |
| Pr(Mig\|CHC) (%) | 22.92 | 6.53 | 9.67 | 35.2 |
| Pr(Non-PWID\|CHC) (%) | 23.82 | 10.42 | 6.77 | 46.5 |

*Notes*: $\rho_{rec}$, prevalence of recent PWID; $\rho_{ex}$, prevalence of ex-PWID; $\rho_{mig}$, prevalence of migrants from endemic countries; $\pi_{rec}$, CHC prevalence among recent PWID; $\pi_{ex}$, CHC prevalence among ex-PWID; $\pi_{mig}$, CHC prevalence among migrants from endemic countries; $\pi_{non}$, CHC prevalence among non-PWID; $\pi$ overall CHC prevalence in Portugal; LB, Lower Boundary; UP, Upper Boundary

# APPENDIX

## Fit of the multi-state Markov model

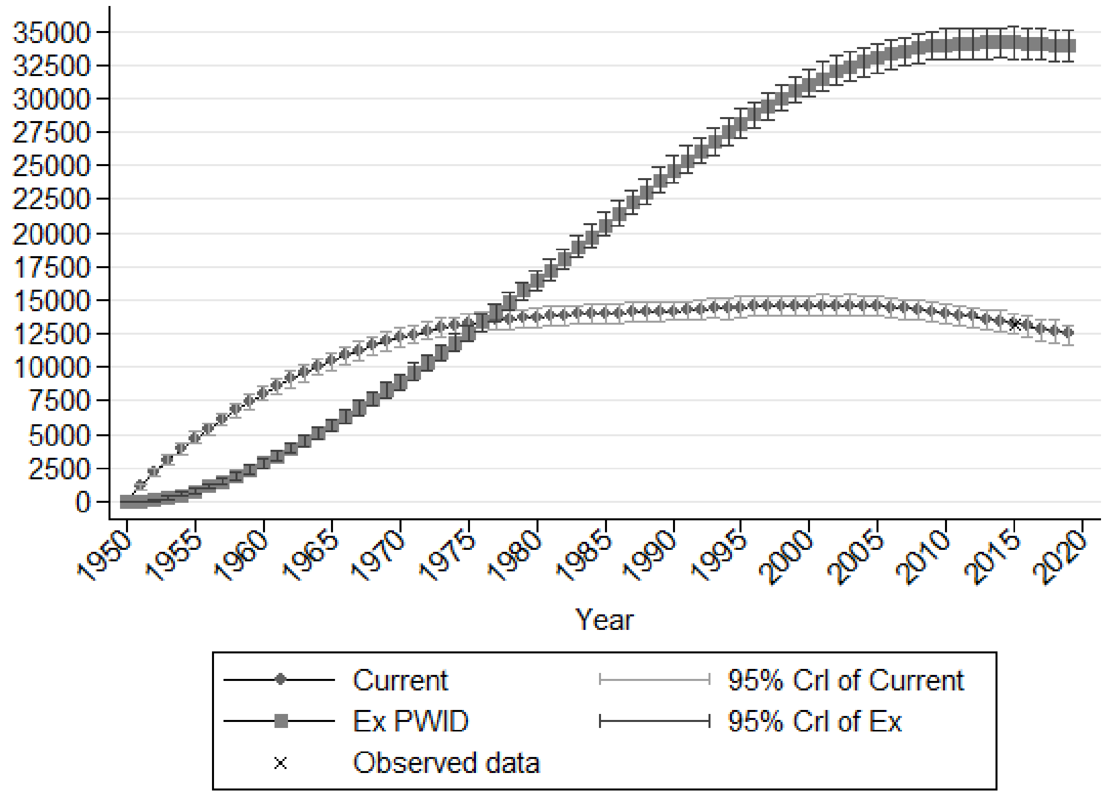


*Figure 2: Model predictions for the numbers of current and ex-PWID (people who inject drugs). The solid black line and shaded grey error bars show the median and 95% credible intervals (95% CrI) for the model projections. For comparison, asterisks indicate the observed country's data.*

## Stan code for Bayesian multiparameter evidence synthesis

```
data {
  int<lower=0> N1579; // Population of 15-79 in `Country`
  int<lower=1> Knon; // Number of studies in the ECDC data for `Country`
  int<lower=1> Kever; // Number of studies in the EMCDDA data for ever users in `Country`
  int<lower=1> Kcur; // Number of studies in the EMCDDA barometer (for HCV prevalence of PWID) in `Country`

  real p_cur_mean; // Prior mean for the prevalence of current use in `Country`
  real<lower=0> p_cur_sd; // Prior sd for the prevalence of current use in `Country`

  real p_ex_mean; // Prior mean for the prevalence of ex-use in `Country`
  real<lower=0> p_ex_sd; // Prior sd for the prevalence of ex-use in `Country`

  //int<lower=0> Nst_CHC_cur[Kcur]; // Number of individuals in the study estimating chronic HCV among recent PWID
  //int<lower=0> Yst_CHC_cur[Kcur]; // Number of HCV+ in the study estimating chronic HCV among recent PWID
  real p_CHC_cur_mean; // Prior mean for the CHC prevalence among recent PWID in `Country`
  real<lower=0> p_CHC_cur_sd; // Prior sd for the CHC prevalence among recent PWID in `Country`

  int<lower=0> Nst_hcv_ever[Kever]; // Number of individuals in the study estimating anti-HCV among ever IDU
  int<lower=0> Yst_hcv_ever[Kever]; // Number of HCV+ in the study estimating anti-HCV among ever PWID
```

```
  int<lower=0> Nst_CHC_non[Knon]; // Number of individuals in the study e
stimating CHC among non PWID
  int<lower=0> Yst_CHC_non[Knon]; // Number of HCV+ in the study estimati
ng CHC among non PWID

  vector<lower=0>[3] alpha; // parameter of the Diriclet prior

  real HCVclear_mean; // Prior mean for the HCV clearance probability
  real<lower=0> HCVclear_sd; // Prior sd for the HCV clearance probabilit
y
}

// Block defining the original parameters
parameters {
  // The parameters to be sampled
  simplex[3] rho; // Prevalence of the three risk groups
  real<lower=0,upper=1> CHCpi_cur; // P(CHC+|Cur)
  real<lower=0,upper=1> CHCpi_ex; // P(CHC+|Ex)
  real<lower=0,upper=1> CHCpi_non; // P(CHC+|Non)

  real<lower=0,upper=1-CHCpi_cur> HCVclear; // Probability of HCV clearan
ce; upper bound = 1-prevalence of chronic HCV
}


transformed parameters {
  // Change scales
  real<lower=0,upper=1> rho_ever;
  real<lower=0,upper=1> CHCpi_ever;
  real<lower=0,upper=1> pi_ever;
  real<lower=0,upper=1> pi_non;
```

```
  rho_ever = rho[1] + rho[2];
  CHCpi_ever = CHCpi_cur*rho[1]/rho_ever + CHCpi_ex*rho[2]/rho_ever;
  pi_ever = CHCpi_ever/(1-HCVclear);
  pi_non = CHCpi_non/(1-HCVclear);
}


// Binomial regression model
model {
  // Priors
  rho ~ dirichlet(alpha);

  //////////////////////////////
  // Likelihood contributions //
  //////////////////////////////

  // Probability of HCV clearance
  HCVclear ~ normal(HCVclear_mean,HCVclear_sd);

  // Prevalence of current use
  rho[1] ~ normal(p_cur_mean,p_cur_sd);

  // Prevalence of ex-use
  rho[2] ~ normal(p_ex_mean,p_ex_sd);

  // Prevalence of chronic HCV among current users
  //Yst_CHC_cur ~ binomial(Nst_CHC_cur,CHCpi_cur);
  CHCpi_cur ~ normal(p_CHC_cur_mean,p_CHC_cur_sd);

  // Prevalence of HCV among ever users
  Yst_hcv_ever ~ binomial(Nst_hcv_ever,pi_ever);
```

```
  // HCV+ among non
  Yst_CHC_non ~ binomial(Nst_CHC_non,CHCpi_non);
}


generated quantities {
  // Functions of parameters
  real<lower=0,upper=100> overalCHC;
  real logit_CHCpi_cur;
  real logit_CHCpi_ex;
  real logit_CHCpi_non;
  real logit_rho_cur;
  real logit_rho_ex;
  real logit_rho_non;
  real<lower=0,upper=1> pEverGivenCHC;
  real<lower=0,upper=1> pNonGivenCHC;
  real logit_HCVclear;
  real<lower=0> NumberCHC;

  // Overall HCV prevalence
  overalCHC = 100*(rho[1]*CHCpi_cur + rho[2]*CHCpi_ex +  rho[3]*CHCpi_non
);
  pEverGivenCHC = CHCpi_ever*rho_ever/(overalCHC/100);
  pNonGivenCHC = CHCpi_non*(1-rho_ever)/(overalCHC/100);
  NumberCHC = round(overalCHC*N1579/100);

  logit_rho_cur = logit(rho[1]);
  logit_rho_ex = logit(rho[2]);
  logit_rho_non = logit(rho[3]);
  logit_CHCpi_cur = logit(CHCpi_cur);
  logit_CHCpi_ex = logit(CHCpi_ex);
  logit_CHCpi_non = logit(CHCpi_non);
```

```
  logit_HCVclear = logit(HCVclear);
}
```

## Multi-state Markov model

```
#include <stdio.h>
#include <stdlib.h>
#include <string.h>
#include <math.h>
#include <time.h>

#define loops 100
int populationPerYearAndAge[2021][66];
double deathRatePerAge[66];
int totalPopulationPerYear[2021];

FILE *F_DeathRate=fopen("greece-deathRates.txt","r");
FILE *F_Population=fopen("greece-population.txt","r");
FILE *out;

double pa_start = 0.00012;                    // Setting Variable
double pa_step  = 0.000002;                   // Setting Variable
double pa_stop  = 0.000123;                   // Setting Variable

double pa;
double pg = 1.0/11.7;   // Assumed average active injecting career of  11
.7 years
double pk = 0.004*12.0;     // Relapse rate of 0.004/month

struct people{
    int age;
    int state;
};

char filename[100];
struct people person[50000000];
```

```
long int totalPersons=0;

void setPopulationAge()
{
    for( int j=0; j<71; j++){
        for( int i=0; i<51; i++){
            fscanf(F_Population,"%d",&populationPerYearAndAge[1950+j][15+
i]);
        }
    }
}

void setDeathRate()
{
    for( int i=0; i<51; i++){
        fscanf(F_DeathRate,"%lf",&deathRatePerAge[15+i*5]);
        for( int j=0; j<5; j++){
            deathRatePerAge[15+i*5+j] = deathRatePerAge[15+i*5];
        }
    }
}

void getTotalPopulationPerAge()
{
    for( int j=0; j<71; j++){
        for( int i=0; i<51; i++){
            totalPopulationPerYear[1950+j] += populationPerYearAndAge[195
0+j][15+i];
        }
    }
}
```

```
void printTotalPopulationPerAge()
{
    printf("---- - ----------\n");
    printf("Year - Population\n");
    printf("---- - ----------\n");
    for( int i=1950; i<2021; i++){
        printf("%d - %d\n",i,totalPopulationPerYear[i]);
    }
}

void printDeathRatePerAge()
{
    printf("--- - -----\n");
    printf("Age - Rate\n");
    printf("--- - -----\n");
    for( int i=15; i<65; i++){
        printf("%d - %lf\n",i,deathRatePerAge[i]);
    }
}

void printTotalPersonPerState( int year)
{
    int count[4]= {0,0,0,0};
    for( int i=0; i<totalPersons; i++){
        count[person[i].state]++;
    }
    //printf("%d\t%ld\t%ld\t%ld\t%ld\n",year,count[0],count[1],count[2],c
ount[3]);
    if(year==2014)
        printf("%d\t%ld\t%ld\t%ld\t%ld\t%lf\n",year,count[0],count[1],cou
nt[2],count[3],pa);
    if( year>2009){
```

```
        fprintf(out, "%d\t%ld\t%ld\t%ld\t%ld\n",year,count[0],count[1],co
unt[2],count[3]);
    }
}

void initializePopulation()
{
    int cnt=0;
    for( int j=15; j<65; j++){
        for( int i=0; i<populationPerYearAndAge[1950][j]*100; i++){
            person[cnt].age=j;
            person[cnt].state=0;
            cnt++;
        }
    }
    totalPersons = cnt;
}

bool checkRate(double rate)
{
    if (rate <0.001){
        double k = rand()/(1.00+RAND_MAX);
        if(k<0.001){
            rate = rate*1000;
        }
        else{
            return false;
        }
    }
    double k = rand()/(1.00+RAND_MAX);
    if ( k < rate )
        return true;
```

```
    else
        return false;
}

void changeStatusAndAge()
{
    for( int i=0; i<totalPersons; i++){
        // change state
        double k = rand()/(1.00+RAND_MAX);
        if( person[i].state==0){
            if ( checkRate(pa) &&  person[i].age<40){
                person[i].state=1;
            }
        }
        else if( person[i].state==1){
            if ( k < pg ){
                person[i].state=2;
            }
        }
        else if( person[i].state==2){
            if ( k < pk ){
                person[i].state=1;
            }
        }
        // After their 49th year, all active PWID are assumed to cease in
jecting
        if(person[i].age>49 && person[i].state==1){
            person[i].state=2;
        }
        // After their 64th year, remove
        if(person[i].age>64){
            person[i].state=3;
```

```
        }
        // death rate depending on age and state
        k = rand()/(1.00+RAND_MAX);
        if( person[i].state==0){
            if ( k < deathRatePerAge[person[i].age] ){
                person[i].state=3;
            }
        }
        else if( person[i].state==1){
            if ( k < deathRatePerAge[person[i].age] * 13.0 ){
                person[i].state=3;
            }
        }
        else if( person[i].state==2){
            if ( k < deathRatePerAge[person[i].age] ){
                person[i].state=3;
            }
        }
        // increase age one year
        person[i].age++;
    }
}

void addNewPersons(int year)
{
    // add new person [15 years old and non PWID]
    int cnt=totalPersons;
    for( int i=0; i<populationPerYearAndAge[year+1][15]*100; i++){
// 100 instead of 1000 for the population to reduce execution time
        person[cnt].age=15;
        person[cnt].state=0;
        cnt++;
```

```
    }
    // increase the total number of population
    totalPersons = cnt;
}
int main()
{
    srand(time(NULL));

    setPopulationAge();
    setDeathRate();

    getTotalPopulationPerAge();
    pa = pa_start;
    while ( pa < pa_stop){

        snprintf(filename, 100, "result_%lf.txt",pa);
        out=fopen(filename,"w");

        for( int iter=0; iter<loops ;iter++){
            initializePopulation();
            for( int year=1950; year<2020; year++){
                printTotalPersonPerState(year);
                changeStatusAndAge();
                addNewPersons(year);
            }
        }

        fclose(out);
        pa = pa + pa_step;
    }
```

```
}
```

# REFERENCES

Carvalhana, Sofia C., Jorge Leitão, Ana C. Alves, Mafalda Bourbon, and Helena Cortez-Pinto. 2016. "Hepatitis b and c Prevalence in Portugal: Disparity Between the General Population and High-Risk Groups." *European Journal of Gastroenterology & Hepatology* 28 (6). https://journals.lww.com/eurojgh/Fulltext/2016/06000/Hepatitis_B_and_C_prevalence_in_Portugal_.5.aspx.

European Centre for Disease Prevention and Control. 2016. "Epidemiological Assessment of Hepatitis b and c Among Migrants in the EU/EEA." https://www.ecdc.europa.eu/sites/default/files/media/en/publications/Publications/epidemiological-assessment-hepatitis-B-and-C-among-migrants-EU-EEA.pdf.

European Centre for Disease Prevention and Control (ECDC). 2016. "Systematic Review on Hepatitis b and c Prevalence in the EU/EEA." https://www.ecdc.europa.eu/sites/default/files/media/en/publications/Publications/systematic-review-hepatitis-B-C-prevalence.pdf.

———. 2021. "Hepatitis c Prevalence Database." https://www.ecdc.europa.eu/en/all-topics-z/hepatitis-c/tools/hepatitis-c-prevalence-database.

Grebely, Jason, Sarah Larney, Amy Peacock, Samantha Colledge, Janni Leung, Matthew Hickman, Peter Vickerman, et al. 2019. "Global, Regional, and Country-Level Estimates of Hepatitis c Infection Among People Who Have Recently Injected Drugs." *Addiction* 114 (1): 150–66. https://doi.org/https://doi.org/10.1111/add.14393.

Lin, Lifeng, and Haitao Chu. 2020. "Meta-Analysis of Proportions Using Generalized Linear Mixed Models." *Epidemiology* 31 (5). https://journals.lww.com/epidem/Fulltext/2020/09000/Meta_analysis_of_Proportions_Using_Generalized.16.aspx.

McDonald, Scott A., Rosmawati Mohamed, Maznah Dahlui, Herlianna Naning, and Adeeba Kamarulzaman. 2014. "Bridging the Data Gaps in the Epidemiology of Hepatitis c Virus Infection in Malaysia Using Multi-Parameter Evidence Synthesis." *BMC Infectious Diseases* 14 (1): 564. https://doi.org/10.1186/s12879-014-0564-6.

Micallef, J. M., J. M. Kaldor, and G. J. Dore. 2006. "Spontaneous Viral Clearance Following Acute Hepatitis c Infection: A Systematic Review of Longitudinal Studies." *Journal of Viral Hepatitis* 13 (1): 34–41. https://doi.org/https://doi.org/10.1111/j.1365-2893.2005.00651.x.

Presanis, Anne M., O. Noel Gill, Timothy R. Chadborn, Caterina Hill, Vivian Hope, Louise Logan, Brian D. Rice, Valerie C. Delpech, A. E. Ades, and Daniela De Angelis. 2010. "Insights into the Rise in HIV Infections, 2001 to 2008: A Bayesian Synthesis of Prevalence Evidence." *AIDS* 24 (18). https://journals.lww.com/aidsonline/Fulltext/2010/11270/Insights_into_the_rise_in_HIV_infections,_2001_to.12.aspx.

Sweeting, M. J., D. De Angelis, M. Hickman, and A. E. Ades. 2008. "Estimating hepatitis C prevalence in England and Wales by synthesizing evidence from multiple data sources. Assessing data conflict and model fit." *Biostatistics* 9 (4): 715–34. https://doi.org/10.1093/biostatistics/kxn004.

Tan, Sarah, Susanna Makela, Daliah Heller, Kevin Konty, Sharon Balter, Tian Zheng, and James H. Stark. 2018. "A Bayesian Evidence Synthesis Approach to Estimate Disease Prevalence in Hard-to-Reach Populations: Hepatitis c in New York City." *Epidemics* 23: 96–109. https://doi.org/https://doi.org/10.1016/j.epidem.2018.01.002.

Thein, Hla-Hla, Qilong Yi, Gregory J. Dore, and Murray D. Krahn. 2008. "Estimation of Stage-Specific Fibrosis Progression Rates in Chronic Hepatitis c Virus Infection: A Meta-Analysis and Meta-Regression." *Hepatology* 48 (2): 418–31. https://doi.org/https://doi.org/10.1002/hep.22375.

Trickey, Adam, Hannah Fraser, Aaron G Lim, Amy Peacock, Samantha Colledge, Josephine G Walker, Janni Leung, et al. 2019. "The Contribution of Injection Drug Use to Hepatitis c Virus Transmission Globally, Regionally, and at Country Level: A Modelling Study." *The Lancet Gastroenterology & Hepatology* 4 (6): 435–44. https://doi.org/https://doi.org/10.1016/S2468-1253(19)30085-8.

Veen, Maaike G. van, Anne M. Presanis, Stefano Conti, Maria Xiridou, Annemarie R. Stengaard, Martin C. Donoghoe, Ard I. van Sighem, Marianne A. van der Sande, and Daniela De Angelis. 2011. "National Estimate of HIV Prevalence in the Netherlands: Comparison and Applicability of Different Estimation Tools." *AIDS* 25 (2).

https://journals.lww.com/aidsonline/Fulltext/2011/01140/National_estimate_of_HIV_preval
ence_in_the.14.aspx.

# Estimating the prevalence of chronic hepatitis C infection (CHC) in Romania using Bayesian multiparameter evidence synthesis

16/05/2022

## Table of Contents


Introduction ........ 2

Methods ........ 2

Prevalence of ex-PWID ........ 3

Prevalence of recent PWID ........ 3

Prevalence of CHC among recent PWID ........ 4

Prevalence of CHC among ex-PWID ........ 4

Prevalence of CHC among non-PWID ........ 5

Sensitivity analysis including migrants from endemic countries ........ 6

Results ........ 6

Tables and Figures ........ 8

Appendix ........ 13

Fit of the multi-state Markov model ........ 13

Stan code for Bayesian multiparameter evidence synthesis ........ 14

Multi-state Markov model ........ 18

References ........ 26

## INTRODUCTION

Patients with chronic hepatitis C (HCV) infection often remain asymptomatic for decades and even for life, but a proportion of them develop active chronic hepatitis, which is a progressive disease (Thein et al. 2008). Because of the largely asymptomatic nature of HCV infection, notification data reflect national screening and testing practices and do not give accurate insights into the prevalence of HCV infection. Although there are studies that have estimated the prevalence of HCV antibodies (anti-HCV) in specific groups such as the general population, first-time blood donors, or people who inject drugs (PWID) at drug treatment centers, those alone cannot be combined to produce national estimates unless some additional information regarding the composition of each HCV risk group and its prevalence in the population are known (Sweeting et al. 2008; McDonald et al. 2014; Tan et al. 2018).

## METHODS

Bayesian multiparameter evidence synthesis (MPES) has been a popular approach to formally estimate anti-HCV or HIV prevalence (Sweeting et al. 2008; McDonald et al. 2014; Tan et al. 2018; Presanis et al. 2010; Veen et al. 2011). In this project, we extend this method to estimating the CHC prevalence, with the population (15-79 years) split into three main non-overlapping risk groups, i.e., recent PWID (those who have injected in the last year), ex-PWID, and non-PWID. A unified model is assumed including parameters associated with the CHC prevalence of recent, ex-PWID, and non-PWID (denoted by $\pi_{rec}$, $\pi_{ex}$, and $\pi_{non}$, respectively) and the prevalence of these risk groups in the population (proportion of the population that belongs to each group), denoted by $\rho_{rec}$, $\rho_{ex}$, and $\rho_{non}$, respectively; $\rho_{rec} + \rho_{ex} + \rho_{non} = 1$. To estimate the CHC prevalence in the whole population, $\pi$, we used the formula:

$$\pi = \pi_{rec}\rho_{rec} + \pi_{ex}\rho_{ex} + \pi_{non}\rho_{non} \quad (1)$$

Bayesian synthesis considers all available data, allowing for an evidence-based estimation of CHC prevalence in the population, with inherent uncertainty properly accounted for. Our aim is to estimate the CHC prevalence for the total (15-79) population of Romania in 2019 using sources of information over the last decade (2010-2019).

## Prevalence of ex-PWID

Data on the prevalence of ex-PWID (proportion of the population that is ex-PWID) are sparse and generally often unreliable. To overcome this limitation, we apply the method proposed by McDonald et al. (2014), which requires simulating a multi-state Markov model representing the non-PWID, recent PWID, and ex-PWID. In the multi-state Markov model, the simulation starts in 1950 and examines the population aged 15-79 years old. A certain number of 15-year-old individuals enter the simulation process each year. These numbers of 15-year-old individuals are obtained from the United Nations Statistic Division UNSD, being different for each country. The ageing of the population is also taken into account. Country-specific overall mortality data are retrieved from the Life Tables of the World Health Organization WHO. The duration of injecting career is also country-specific and obtained from the paper of Hines et al. (2020).

To estimate the number of ex-PWID, the model can be calibrated on the number of recent PWID provided in the EMCDDA barometer or the EMCDDA statistical bulletin (the code of the Markov model can be found in the Appendix). If there are no available data in the barometer, estimates from the systematic review of Grebely et al. (2019) could be used. Alternatively, if the national focal points suggest or provide different and updated or more accurate data for calibration purposes, we will consider their advice and adjust the model accordingly.

After applying the model for Romania, we compute the number (and the corresponding Confidence Interval - CI) of ex-PWID in 2019. The number of ex-PWID is then divided by the population size (15-79 years) in the same year, and a Normal distribution, constrained such that $\rho_{ex} \in (0,1)$, is assumed for $\rho_{ex}$. The standard deviation of this distribution is specified to approximately correspond to the respective CI, i.e. $\left(UB_{\rho_{ex}} - LB_{\rho_{ex}}\right)/(2 \times 1.96)$, where $UB_{\rho_{ex}}$ and $LB_{\rho_{ex}}$ denote the upper and lower limit of the CI.

## Prevalence of recent PWID

The number of recent PWID in Romania in 2019 is also projected by the multi-state Markov model described in the previous subsection. To estimate $\rho_{rec}$, the number of recent PWID is divided by the population size (15-79 years). Similar to the previous subsection, a Normal distribution, constrained such that $\rho_{rec} \in (0,1)$, is assumed for $\rho_{rec}$, with the standard deviation specified to correspond to the CI obtained from the multi-state Markov model.

## Prevalence of CHC among recent PWID

The prevalence (and the 95% CI) of CHC among recent PWID can be informed from studies in the EMCDDA statistical bulletin. If information on the CHC prevalence is available, the Binomial distribution in the model to inform $\pi_{rec}$ was used. However, as these EMCDDA data typically refer to the anti-HCV prevalence and not to the CHC prevalence, they could not be used directly. This issue is addressed by noting that spontaneous viral clearance occurs in approximately one in four people with acute hepatitis C (Micallef, Kaldor, and Dore 2006). To account for the variability of the HCV clearance probabilities, we used the result reported in Micallef, Kaldor, and Dore (2006), i.e., the proportion of HCV clearance (denoted by $\rho_{clear}$) is equal to 0.26 (95% CI 0.22–0.29). Thus, estimates of the CHC prevalence among recent PWID ($\pi_{rec}$) can be obtained using the formula $\pi_{rec} = \pi(\text{anti-HCV})_{rec}(1 - \rho_{clear})$, where $\pi(\text{anti-HCV})_{rec}$ denotes the anti-HCV prevalence among recent PWID.

Apart from spontaneous clearance, some people may have been treated with direct acting antivirals (DAAs). Since treatment data may be weak and unstandardised or unavailable for most EU/EEA countries, we have not formally taken treatment into account.

If information on the HCV prevalence (CHC or anti-HCV) among recent PWID in the EMCDDA database is not available, the required information is obtained from the paper of Grebely et al. (2019). Currently, CHC prevalence data from the paper of Grebely et al. (2019) were used.

## Prevalence of CHC among ex-PWID

Information on CHC prevalence among ex-PWID (i.e., $\pi_{ex}$) is difficult to obtain directly. To overcome this, nationwide data on the HCV prevalence among ever users through the EMCDDA database was utilized. However, if these data refer to the anti-HCV prevalence, they are adjusted according to the procedure described in the previous subsection, i.e. $\pi_{ever} = \pi(\text{anti-HCV})_{ever}(1 - \rho_{clear})$, where $\pi(\text{anti-HCV})_{ever}$ denotes the anti-HCV prevalence among ever PWID. In any case, if an estimate of $\pi_{ever}$ is available, $\pi_{ex}$ can be indirectly estimated since $\pi_{ever}$ is equal to a weighted average of $\pi_{rec}$ and $\pi_{ex}$, as shown by the following formula

$$\pi_{ever} = \frac{\rho_{rec}}{\rho_{rec} + \rho_{ex}}\pi_{rec} + \frac{\rho_{ex}}{\rho_{rec} + \rho_{ex}}\pi_{ex}, \quad (2)$$

or, equivalently,

$$\pi_{ex} = \left(\pi_{ever} - \frac{\rho_{rec}}{\rho_{rec} + \rho_{ex}}\pi_{rec}\right) \times \frac{\rho_{rec} + \rho_{ex}}{\rho_{ex}}, \quad (3)$$

Currently, anti-HCV prevalence data from ever PWID in Bucharest in 2017, available in the EMCDDA statistical bulletin, were used.

## Prevalence of CHC among non-PWID

To estimate the CHC prevalence among non-PWID in a country (after 2010), the ECDC database (European Centre for Disease Prevention and Control (ECDC) 2021) was used. The ECDC group has carefully and critically collected information from relevant studies across the EU/EEA region in cooperation with the focal points of each country. On the basis of the representativeness of each study and other factors (European Centre for Disease Prevention and Control (ECDC) 2016), a risk of bias score was assigned to each study, with higher values indicating higher-quality studies. When higher-quality studies are available in a country (≥ 4), they are used to estimate the overall CHC prevalence in that country among non-PWID. If there are no higher-quality studies, general population estimates with a lower quality (<4) are pooled. If no general population prevalence estimates are available, data on the prevalence of HCV in pregnant women could be used as a prevalence measure. Finally, if data on pregnant women are lacking too, first-time blood donor studies could be an option. However, if the national focal point agrees or recommends, estimates from a neighboring country could be used instead, or we could use any other relevant information provided/suggested by the national focal point.

We directly use CHC prevalence data from studies in the general population, if available. If the data in the general population refer to anti-HCV prevalence (without any data on the viremic population), we adjust the estimates to get CHC prevalence based on the spontaneous HCV clearance estimate of 26%, described in the previous subsection.

As $\rho_{rec}$ and $\rho_{ex}$ are likely to be low, $\pi_{non}$ will be the most critical factor for the estimation of the overall CHC prevalence in the population. To adjust for potential heterogeneity between the CHC prevalence estimates from different studies in the non-PWID population in a country, as a sensitivity analysis, we also apply a random-effect meta-analytic approach to pool the CHC

prevalence across studies on non-PWID (Lin and Chu 2020). The random-effect approach is carried out only when at least 3 studies are available in the general population of a certain country, and the estimates are compared with the corresponding estimates from the approach assuming no heterogeneity (similar to a fixed-effect meta-analysis). If the estimates are inconsistent, possible explanations are investigated and discussed. If there is substantial heterogeneity between studies, it would help more to explore the causes; for example, following national focal point suggestions, some studies could be excluded to reduce heterogeneity.

## Sensitivity analysis including migrants from endemic countries

A sensitivity analysis including data for the viremic population among migrants based on the report of the ECDC group (European Centre for Disease Prevention and Control 2016) was carried out. Migrants from endemic countries represent 0.6% of the adult population in Romania (Table 8 in European Centre for Disease Prevention and Control (2016)), with the respective CHC prevalence being equal to $\pi_{mig} = 2\%$ (Table 9 in European Centre for Disease Prevention and Control (2016)). The overall CHC prevalence is now estimated by

$$\pi = \pi_{rec}\rho_{rec} + \pi_{ex}\rho_{ex} + \pi_{mig}\rho_{mig} + \pi_{non}\rho_{non},$$

where $\rho_{rec} + \rho_{ex} + \rho_{mig} + \rho_{non} = 1$.

# RESULTS

The above-mentioned approach was used to estimate the prevalence of CHC in 2019 (considering ages between 15-79 years: 15,465,818).

The aggregated data used by our approach are briefly presented in Table 1. The multi-state Markov model was calibrated on the estimates reported in the systematic review of Grebely et al. (2019). In Romania, there were 2 studies of medium quality, which included CHC data (Gheorghe et al. 2020; Huiban et al. 2021). To estimate the CHC prevalence in the general population (primarily non-PWID), we used a fixed-effect meta-analytic approach, with the corresponding results presented in Table 2. The prevalence of recent and ex-PWID (proportion of the population that belongs to these groups) was low in Romania (about 0.49% and 1.31%, respectively) corresponding to 75,485 (95% CI: 73,650-77,680) recent PWID and 203,220 (95% CI: 199,600-206,670) ex-PWID in the population. However, the CHC prevalence in these

groups was substantial (Table 2) being 62.94% and 56.97%, respectively. This translates to 47,517 (95% CI: 44,085-50,887) and 115,778 (95% CI: 104,141-127,216) recent and ex-PWID aged 15-79 living with CHC infection in Romania in 2019. The CHC prevalence in the general population was 1.22% (95% CI: 1.1%-1.36%), much lower than that of the high-risk groups. Taking all pieces of information into account, the overall CHC prevalence in Romania in 2019 was equal to 2.26% (95% CI: 2.11%-2.41%), which corresponds to 348,939 (95% CI: 326,554-372,034) individuals aged 15-79 years with CHC infection.

The results from our model including migrants from endemic countries as a separate group are presented in Table 3. However, this analysis comes with possible limitations; that is, including migrants as a separate group is valid only if migrants do not overlap with the remaining groups (recent PWID, ex-PWID, and non-PWID). In any case, the results from Table 3 are identical to those reported in Table 2, thus, including or not migrants as a separate non-overlapping group has no effect on the total CHC prevalence estimate in Romania.

# TABLES AND FIGURES

*Table 1. Data (real) contributing to the estimation of CHC prevalence in Romania in 2019.*

| Parameter | Estimate (95%CI) | Number (95%CI) | Numerator | Denominator | Notes | Year of study |
|---|---|---|---|---|---|---|
| $\rho_{rec}$ | | 75,485 (73,650-77,680) | | | Method based on McDonald et al. | 2019 |
| $\rho_{ex}$ | | 203,220 (199,600-206,670) | | | Method based on McDonald et al. | 2019 |
| $\pi_{rec}$ | 62.9% (58.7%-67%) | | | | Grebely et al. | 2009 |
| $\pi(\text{anti-HCV})_{ever}$ | | | 352 | 444 | EMCDDA database | 2017 |
| $\pi_{non}$ | | | 65 | 2,866 | ECDC database (Huiban et al.); Risk of bias=3† | 2019 |
| $\pi_{non}$ | | | 276 | 25,085 | ECDC database (Gheorghe et al.); Risk of bias=3†† | 2019 |

*Notes*: Although it looks counter-intuitive, **higher risk of bias score denotes a higher-quality study** (range from 0 to 6); † After excluding PWID from the population: 1 anti-HCV positive out of 79 PWID; †† After excluding PWID from the population: 56 PWID with 16.07% anti-HCV prevalence.

*Table 2. Results from the method assuming no heterogeneity in the estimation of the CHC prevalence in the general population from different studies (i.e., similar to a fixed-effect meta-analysis).*

| Parameter | Posterior median | Posterior sd | LB (2.5%) | UB (97.5%) |
|---|---|---|---|---|
| $\rho_{rec}$ (%) | 0.49 | 0.01 | 0.48 | 0.5 |
| $\rho_{ex}$ (%) | 1.31 | 0.01 | 1.29 | 1.34 |
| $\pi_{rec}$ (%) | 62.94 | 2.14 | 58.71 | 67.11 |
| $\pi_{ex}$ (%) | 56.97 | 2.86 | 51.36 | 62.49 |
| $\pi_{non}$ (%) | 1.22 | 0.07 | 1.1 | 1.36 |
| $\pi$ (%) | 2.26 | 0.08 | 2.11 | 2.41 |
| Number with CHC | 348,939 | 11,644 | 326,554 | 372,034 |
| Pr(Ever PWID\|CHC) (%) | 46.81 | 1.62 | 43.61 | 49.97 |
| Pr(Non-PWID\|CHC) (%) | 53.19 | 1.62 | 50.03 | 56.39 |

*Notes*: $\rho_{rec}$, prevalence of recent PWID (proportion of the population that is recent PWID); $\rho_{ex}$, prevalence of ex-PWID (proportion of the population that is ex-PWID); $\pi_{rec}$, CHC prevalence among recent PWID; $\pi_{ex}$, CHC prevalence among ex-PWID; $\pi_{non}$, CHC prevalence among non-PWID; $\pi$ overall CHC prevalence in Romania; LB, Lower Boundary; UP, Upper Boundary

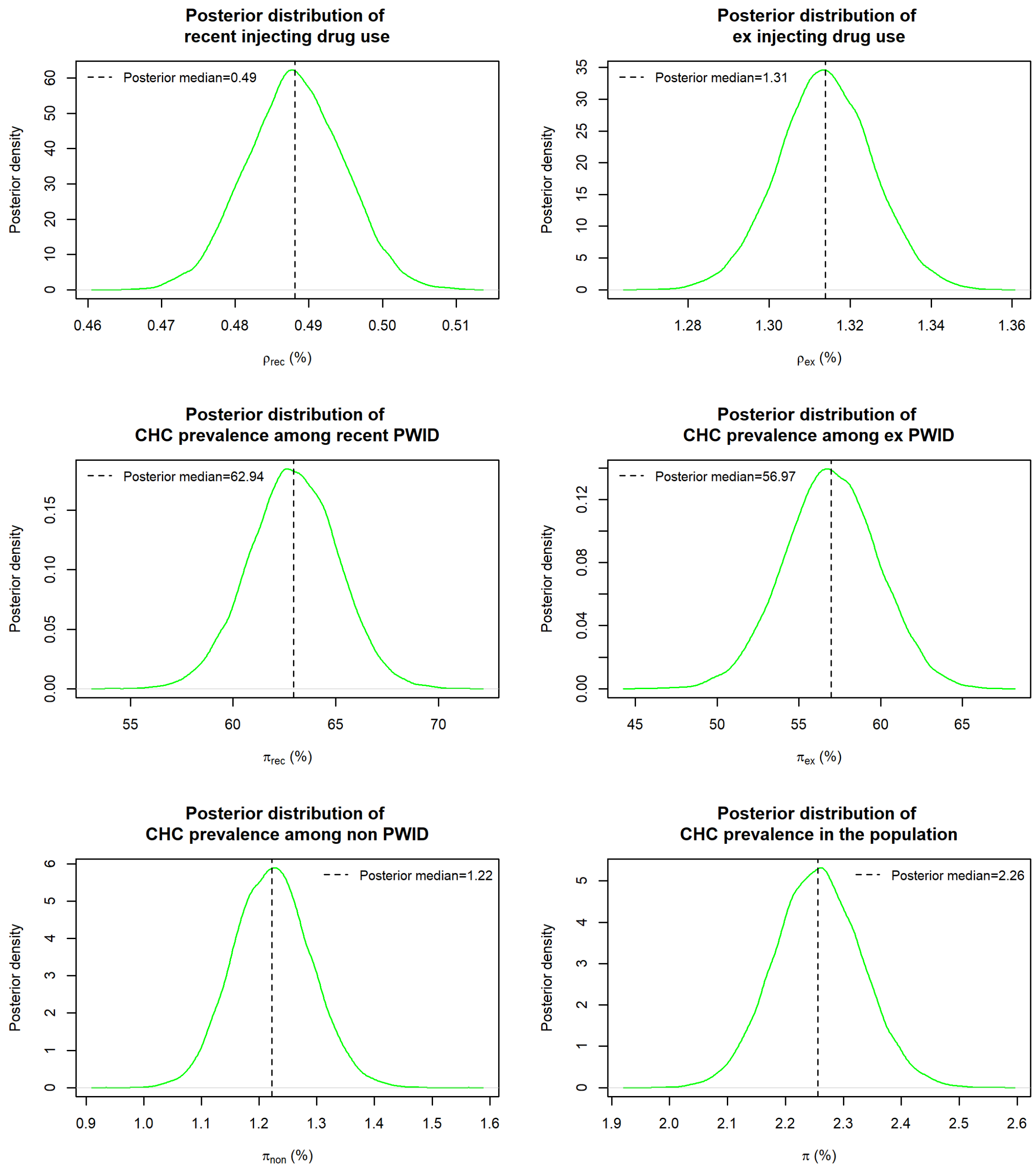


*Figure 1. Posterior distributions of the key model parameters using the approach that assumes no heterogeneity in the estimation of the CHC prevalence in the general population (primarily non-PWID) from different studies (i.e., similar to a fixed-effect meta-analysis).*

*Table 3. Results from the method assuming no heterogeneity in the estimation of the CHC prevalence in the general population (primarily non-PWID) from different studies (i.e., similar to a fixed-effect meta-analysis) including migrants from endemic countries as a separate group.*

| Parameter | Posterior median | Posterior sd | LB (2.5%) | UB (97.5%) |
|---|---|---|---|---|
| $\rho_{rec}$ (%) | 0.49 | 0.01 | 0.47 | 0.5 |
| $\rho_{ex}$ (%) | 1.31 | 0.01 | 1.29 | 1.34 |
| $\rho_{mig}$ (%) | 0.6 | 0 | 0.6 | 0.6 |
| $\pi_{rec}$ (%) | 62.91 | 2.14 | 58.77 | 67.09 |
| $\pi_{ex}$ (%) | 57.07 | 2.86 | 51.35 | 62.65 |
| $\pi_{mig}$ (%) | 2 | 0.5 | 1.02 | 2.99 |
| $\pi_{non}$ (%) | 1.22 | 0.07 | 1.1 | 1.35 |
| $\pi$ (%) | 2.26 | 0.07 | 2.12 | 2.41 |
| Number with CHC | 349,771 | 11,386 | 328,149 | 372,628 |
| Pr(Ever PWID\|CHC) (%) | 46.71 | 1.58 | 43.63 | 49.84 |
| Pr(Mig\|CHC) (%) | 0.53 | 0.13 | 0.27 | 0.8 |
| Pr(Non-PWID\|CHC) (%) | 52.76 | 1.59 | 49.61 | 55.86 |

*Notes*: $\rho_{rec}$, prevalence of recent PWID (proportion of the population that is recent PWID); $\rho_{ex}$, prevalence of ex-PWID (proportion of the population that is ex-PWID); $\rho_{mig}$, prevalence of migrants from endemic countries (proportion of the population that belongs to this group); $\pi_{rec}$, CHC prevalence among recent PWID; $\pi_{ex}$, CHC prevalence among ex-PWID; $\pi_{mig}$, CHC prevalence among migrants from endemic countries; $\pi_{non}$, CHC prevalence among non-PWID; $\pi$ overall CHC prevalence in Romania; LB, Lower Boundary; UP, Upper Boundary

# APPENDIX

## Fit of the multi-state Markov model

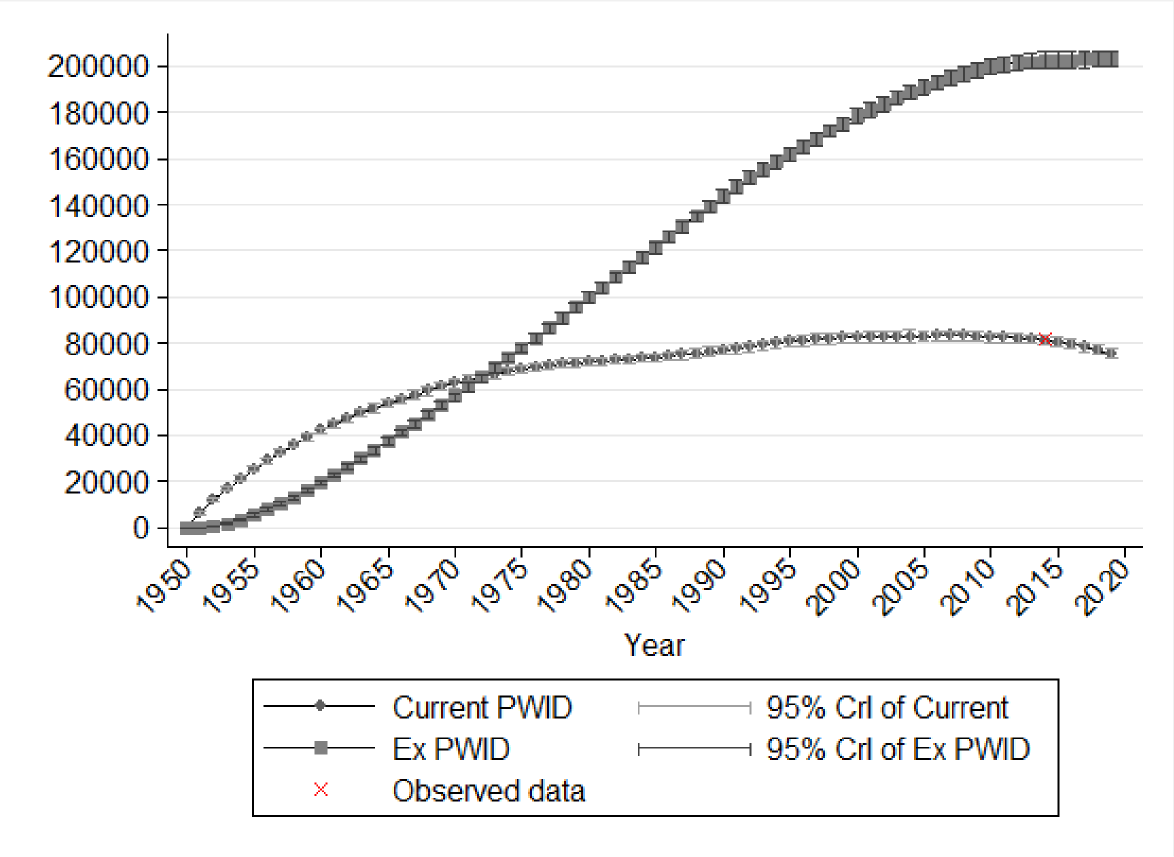


*Figure 2: Model predictions for the numbers of current and ex-PWID (people who inject drugs). The solid black line and shaded grey error bars show the median and 95% credible intervals (95% CrI) for the model projections. For comparison, asterisks indicate the observed country's data.*

## Stan code for Bayesian multiparameter evidence synthesis

```
data {
  int<lower=0> N1579; // Population of 15-79 in `Country`
  int<lower=1> Knon; // Number of studies in the ECDC data for `Country`
  int<lower=1> Kever; // Number of studies in the EMCDDA data for ever us
ers in `Country`
  int<lower=1> Kcur; // Number of studies in the EMCDDA barometer (for HC
V prevalence of PWID) in `Country`

  real p_cur_mean; // Prior mean for the prevalence of current use in `Co
untry`
  real<lower=0> p_cur_sd; // Prior sd for the prevalence of current use i
n `Country`

  real p_ex_mean; // Prior mean for the prevalence of ex-use in `Country`
  real<lower=0> p_ex_sd; // Prior sd for the prevalence of ex-use in `Cou
ntry`

  //int<lower=0> Nst_hcv_cur[Kcur]; // Number of individuals in the study
estimating anti-HCV among recent PWID
  //int<lower=0> Yst_hcv_cur[Kcur]; // Number of HCV+ in the study estima
ting anti-HCV among recent PWID
  real p_CHC_cur_mean; // Prior mean for the CHC prevalence among recent
PWID in `Country`
  real<lower=0> p_CHC_cur_sd; // Prior sd for the CHC prevalence among re
cent PWID in `Country`

  int<lower=0> Nst_hcv_ever[Kever]; // Number of individuals in the study
estimating HCV among ever IDU
  int<lower=0> Yst_hcv_ever[Kever]; // Number of HCV+ in the study estima
ting HCV among ever PWID
```

```
  int<lower=0> Nst_CHC_non[Knon]; // Number of individuals in the study e
stimating CHC among non PWID
  int<lower=0> Yst_CHC_non[Knon]; // Number of HCV+ in the study estimati
ng CHC among non PWID

  vector<lower=0>[3] alpha; // parameter of the Diriclet prior

  real HCVclear_mean; // Prior mean for the HCV clearance probability
  real<lower=0> HCVclear_sd; // Prior sd for the HCV clearance probabilit
y
}

// Block defining the original parameters
parameters {
  // The parameters to be sampled
  simplex[3] rho; // Prevalence of the three risk groups
  real<lower=0,upper=1> CHCpi_cur; // P(CHC+|Cur)
  real<lower=0,upper=1> CHCpi_ex; // P(CHC+|Ex)
  real<lower=0,upper=1> CHCpi_non; // P(CHC+|Non)

  real<lower=0,upper=1-CHCpi_cur> HCVclear; // Probability of HCV clearan
ce; upper bound = 1-prevalence of chronic HCV
}


transformed parameters {
  // Change scales
  real<lower=0,upper=1> rho_ever;
  real<lower=0,upper=1> CHCpi_ever;
  real<lower=0,upper=1> pi_ever;

  rho_ever = rho[1] + rho[2];
```

```
  CHCpi_ever = CHCpi_cur*rho[1]/rho_ever + CHCpi_ex*rho[2]/rho_ever;
  pi_ever = CHCpi_ever/(1-HCVclear);
}


// Binomial regression model
model {
  // Priors
  rho ~ dirichlet(alpha);

  ///////////////////////////////
  // Likelihood contributions //
  ///////////////////////////////

  // Probability of HCV clearance
  HCVclear ~ normal(HCVclear_mean,HCVclear_sd);

  // Prevalence of current use
  rho[1] ~ normal(p_cur_mean,p_cur_sd);

  // Prevalence of ex-use
  rho[2] ~ normal(p_ex_mean,p_ex_sd);

  // Prevalence of chronic HCV among current users
  CHCpi_cur ~ normal(p_CHC_cur_mean,p_CHC_cur_sd);
  //Yst_hcv_cur ~ binomial(Nst_hcv_cur,pi_cur);

  // Prevalence of HCV among ever users
  Yst_hcv_ever ~ binomial(Nst_hcv_ever,pi_ever);

  // HCV+ among non
  Yst_CHC_non ~ binomial(Nst_CHC_non,CHCpi_non);
```

```
}


generated quantities {
  // Functions of parameters
  real<lower=0,upper=100> overalCHC;
  real logit_CHCpi_cur;
  real logit_CHCpi_ex;
  real logit_CHCpi_non;
  real logit_rho_cur;
  real logit_rho_ex;
  real logit_rho_non;
  real<lower=0,upper=1> pEverGivenCHC;
  real<lower=0,upper=1> pNonGivenCHC;
  real logit_HCVclear;
  real<lower=0> NumberCHC;

  // Overall HCV prevalence
  overalCHC = 100*(rho[1]*CHCpi_cur + rho[2]*CHCpi_ex +  rho[3]*CHCpi_non
);
  pEverGivenCHC = CHCpi_ever*rho_ever/(overalCHC/100);
  pNonGivenCHC = CHCpi_non*(1-rho_ever)/(overalCHC/100);
  NumberCHC = round(overalCHC*N1579/100);

  logit_rho_cur = logit(rho[1]);
  logit_rho_ex = logit(rho[2]);
  logit_rho_non = logit(rho[3]);
  logit_CHCpi_cur = logit(CHCpi_cur);
  logit_CHCpi_ex = logit(CHCpi_ex);
  logit_CHCpi_non = logit(CHCpi_non);
  logit_HCVclear = logit(HCVclear);
}
```

## Multi-state Markov model

```
#include <stdio.h>
#include <stdlib.h>
#include <string.h>
#include <math.h>
#include <time.h>

#define loops 100
int populationPerYearAndAge[2021][66];
double deathRatePerAge[66];
int totalPopulationPerYear[2021];

FILE *F_DeathRate=fopen("`Country`-deathRates.txt","r");
FILE *F_Population=fopen("`Country`-population.txt","r");
FILE *out;

double pa_start = 0.00012;                          // Setting Variable
double pa_step  = 0.000002;                         // Setting Variable
double pa_stop  = 0.000123;                         // Setting Variable

double pa;
double pg = 1.0/11.7;    // Assumed average active injecting career of  11
.7 years
double pk = 0.004*12.0;      // Relapse rate of 0.004/month

struct people{
    int age;
    int state;
};

char filename[100];
struct people person[50000000];
```

```
long int totalPersons=0;

void setPopulationAge()
{
    for( int j=0; j<71; j++){
        for( int i=0; i<51; i++){
            fscanf(F_Population,"%d",&populationPerYearAndAge[1950+j][15+
i]);
        }
    }
}

void setDeathRate()
{
    for( int i=0; i<51; i++){
        fscanf(F_DeathRate,"%lf",&deathRatePerAge[15+i*5]);
        for( int j=0; j<5; j++){
            deathRatePerAge[15+i*5+j] = deathRatePerAge[15+i*5];
        }
    }
}

void getTotalPopulationPerAge()
{
    for( int j=0; j<71; j++){
        for( int i=0; i<51; i++){
            totalPopulationPerYear[1950+j] += populationPerYearAndAge[195
0+j][15+i];
        }
    }
}
```

```
void printTotalPopulationPerAge()
{
    printf("---- - ----------\n");
    printf("Year - Population\n");
    printf("---- - ----------\n");
    for( int i=1950; i<2021; i++){
        printf("%d - %d\n",i,totalPopulationPerYear[i]);
    }
}

void printDeathRatePerAge()
{
    printf("--- - -----\n");
    printf("Age - Rate\n");
    printf("--- - -----\n");
    for( int i=15; i<65; i++){
        printf("%d - %lf\n",i,deathRatePerAge[i]);
    }
}

void printTotalPersonPerState( int year)
{
    int count[4]= {0,0,0,0};
    for( int i=0; i<totalPersons; i++){
        count[person[i].state]++;
    }
    //printf("%d\t%ld\t%ld\t%ld\t%ld\n",year,count[0],count[1],count[2],c
ount[3]);
    if(year==2014)
        printf("%d\t%ld\t%ld\t%ld\t%ld\t%lf\n",year,count[0],count[1],cou
nt[2],count[3],pa);
    if( year>2009){
```

```
        fprintf(out, "%d\t%ld\t%ld\t%ld\t%ld\n",year,count[0],count[1],co
unt[2],count[3]);
    }
}

void initializePopulation()
{
    int cnt=0;
    for( int j=15; j<65; j++){
        for( int i=0; i<populationPerYearAndAge[1950][j]*100; i++){
            person[cnt].age=j;
            person[cnt].state=0;
            cnt++;
        }
    }
    totalPersons = cnt;
}

bool checkRate(double rate)
{
    if (rate <0.001){
        double k = rand()/(1.00+RAND_MAX);
        if(k<0.001){
            rate = rate*1000;
        }
        else{
            return false;
        }
    }
    double k = rand()/(1.00+RAND_MAX);
    if ( k < rate )
        return true;
```

```
    else
        return false;
}

void changeStatusAndAge()
{
    for( int i=0; i<totalPersons; i++){
        // change state
        double k = rand()/(1.00+RAND_MAX);
        if( person[i].state==0){
            if ( checkRate(pa) &&  person[i].age<40){
                person[i].state=1;
            }
        }
        else if( person[i].state==1){
            if ( k < pg ){
                person[i].state=2;
            }
        }
        else if( person[i].state==2){
            if ( k < pk ){
                person[i].state=1;
            }
        }
        // After their 49th year, all active PWID are assumed to cease in
jecting
        if(person[i].age>49 && person[i].state==1){
            person[i].state=2;
        }
        // After their 64th year, remove
        if(person[i].age>64){
            person[i].state=3;
```

```
        }
        // death rate depending on age and state
        k = rand()/(1.00+RAND_MAX);
        if( person[i].state==0){
            if ( k < deathRatePerAge[person[i].age] ){
                person[i].state=3;
            }
        }
        else if( person[i].state==1){
            if ( k < deathRatePerAge[person[i].age] * 13.0 ){
                person[i].state=3;
            }
        }
        else if( person[i].state==2){
            if ( k < deathRatePerAge[person[i].age] ){
                person[i].state=3;
            }
        }
        // increase age one year
        person[i].age++;
    }
}

void addNewPersons(int year)
{
    // add new person [15 years old and non PWID]
    int cnt=totalPersons;
    for( int i=0; i<populationPerYearAndAge[year+1][15]*100; i++){
// 100 instead of 1000 for the population to reduce execution time
        person[cnt].age=15;
        person[cnt].state=0;
        cnt++;
```

```
    }
    // increase the total number of population
    totalPersons = cnt;
}
int main()
{
    srand(time(NULL));

    setPopulationAge();
    setDeathRate();

    getTotalPopulationPerAge();
    pa = pa_start;
    while ( pa < pa_stop){

        snprintf(filename, 100, "result_%lf.txt",pa);
        out=fopen(filename,"w");

        for( int iter=0; iter<loops ;iter++){
            initializePopulation();
            for( int year=1950; year<2020; year++){
                printTotalPersonPerState(year);
                changeStatusAndAge();
                addNewPersons(year);
            }
        }

        fclose(out);
        pa = pa + pa_step;
    }
```

```
}
```

## REFERENCES

European Centre for Disease Prevention and Control. 2016. "Epidemiological Assessment of Hepatitis b and c Among Migrants in the EU/EEA." https://www.ecdc.europa.eu/sites/default/files/media/en/publications/Publications/epidemiological-assessment-hepatitis-B-and-C-among-migrants-EU-EEA.pdf.

European Centre for Disease Prevention and Control (ECDC). 2016. "Systematic Review on Hepatitis b and c Prevalence in the EU/EEA." https://www.ecdc.europa.eu/sites/default/files/media/en/publications/Publications/systematic-review-hepatitis-B-C-prevalence.pdf.

———. 2021. "Hepatitis c Prevalence Database." https://www.ecdc.europa.eu/en/all-topics-z/hepatitis-c/tools/hepatitis-c-prevalence-database.

Gheorghe, Liana, Speranta Iacob, Irma Csiki, Laura Huiban, Monica Cojocaru, Camelia Cojocariu, Roxana Nemteanu, et al. 2020. "The Prevalence of HCV Infection and Risk Factors in a Hospital-Based Population Screening, a First Step to the Micro-Elimination of HCV Infection in Medical Institutions from Romania - Results of the HepC ALERT Study." *Journal of Gastrointestinal and Liver Diseases* 29 (4): 587–93. https://doi.org/10.15403/jgld-3084.

Grebely, Jason, Sarah Larney, Amy Peacock, Samantha Colledge, Janni Leung, Matthew Hickman, Peter Vickerman, et al. 2019. "Global, Regional, and Country-Level Estimates of Hepatitis c Infection Among People Who Have Recently Injected Drugs." *Addiction* 114 (1): 150–66. https://doi.org/https://doi.org/10.1111/add.14393.

Hines, Lindsey A., Adam Trickey, Janni Leung, Sarah Larney, Amy Peacock, Louisa Degenhardt, Samantha Colledge, et al. 2020. "Associations Between National Development Indicators and the Age Profile of People Who Inject Drugs: Results from a Global Systematic Review and Meta-Analysis." *The Lancet Global Health* 8 (1): e76–91. https://doi.org/10.1016/S2214-109X(19)30462-0.

Huiban, Laura, Carol Stanciu, Cristina Maria Muzica, Tudor Cuciureanu, Stefan Chiriac, Sebastian Zenovia, Vladut Mirel Burduloi, et al. 2021. "Hepatitis c Virus Prevalence and Risk

Factors in a Village in Northeastern Romania-a Population-Based Screening-the First Step to Viral Micro-Elimination." *Healthcare* 9 (6). https://doi.org/10.3390/healthcare9060651.

Lin, Lifeng, and Haitao Chu. 2020. "Meta-Analysis of Proportions Using Generalized Linear Mixed Models." *Epidemiology* 31 (5). https://journals.lww.com/epidem/Fulltext/2020/09000/Meta_analysis_of_Proportions_Using_Generalized.16.aspx.

McDonald, Scott A., Rosmawati Mohamed, Maznah Dahlui, Herlianna Naning, and Adeeba Kamarulzaman. 2014. "Bridging the Data Gaps in the Epidemiology of Hepatitis c Virus Infection in Malaysia Using Multi-Parameter Evidence Synthesis." *BMC Infectious Diseases* 14 (1): 564. https://doi.org/10.1186/s12879-014-0564-6.

Micallef, J. M., J. M. Kaldor, and G. J. Dore. 2006. "Spontaneous Viral Clearance Following Acute Hepatitis c Infection: A Systematic Review of Longitudinal Studies." *Journal of Viral Hepatitis* 13 (1): 34–41. https://doi.org/https://doi.org/10.1111/j.1365-2893.2005.00651.x.

Presanis, Anne M., O. Noel Gill, Timothy R. Chadborn, Caterina Hill, Vivian Hope, Louise Logan, Brian D. Rice, Valerie C. Delpech, A. E. Ades, and Daniela De Angelis. 2010. "Insights into the Rise in HIV Infections, 2001 to 2008: A Bayesian Synthesis of Prevalence Evidence." *AIDS* 24 (18). https://journals.lww.com/aidsonline/Fulltext/2010/11270/Insights_into_the_rise_in_HIV_infections,_2001_to.12.aspx.

Sweeting, M. J., D. De Angelis, M. Hickman, and A. E. Ades. 2008. "Estimating hepatitis C prevalence in England and Wales by synthesizing evidence from multiple data sources. Assessing data conflict and model fit." *Biostatistics* 9 (4): 715–34. https://doi.org/10.1093/biostatistics/kxn004.

Tan, Sarah, Susanna Makela, Daliah Heller, Kevin Konty, Sharon Balter, Tian Zheng, and James H. Stark. 2018. "A Bayesian Evidence Synthesis Approach to Estimate Disease Prevalence in Hard-to-Reach Populations: Hepatitis c in New York City." *Epidemics* 23: 96–109. https://doi.org/https://doi.org/10.1016/j.epidem.2018.01.002.

Thein, Hla-Hla, Qilong Yi, Gregory J. Dore, and Murray D. Krahn. 2008. "Estimation of Stage-Specific Fibrosis Progression Rates in Chronic Hepatitis c Virus Infection: A Meta-Analysis and

Meta-Regression." *Hepatology* 48 (2): 418–31. https://doi.org/https://doi.org/10.1002/hep.22375.

Veen, Maaike G. van, Anne M. Presanis, Stefano Conti, Maria Xiridou, Annemarie R. Stengaard, Martin C. Donoghoe, Ard I. van Sighem, Marianne A. van der Sande, and Daniela De Angelis. 2011. "National Estimate of HIV Prevalence in the Netherlands: Comparison and Applicability of Different Estimation Tools." *AIDS* 25 (2). https://journals.lww.com/aidsonline/Fulltext/2011/01140/National_estimate_of_HIV_prevalence_in_the.14.aspx.

# Estimating the prevalence of chronic hepatitis C infection (CHC) in Slovakia using Bayesian multiparameter evidence synthesis

12/07/2022

## Table of Contents

Introduction ........ 2
Methods ........ 2
Prevalence of ex-PWID ........ 3
Prevalence of recent PWID ........ 3
Prevalence of CHC among recent PWID ........ 4
Prevalence of CHC among ex-PWID ........ 4
Prevalence of CHC among non-PWID ........ 5
Sensitivity analyses ........ 7
Results ........ 7
Tables and Figures ........ 9
Appendix ........ 13
Fit of the multi-state Markov model ........ 13
Stan code for Bayesian multiparameter evidence synthesis ........ 14
Multi-state Markov model ........ 20
References ........ 28

# INTRODUCTION

Patients with chronic hepatitis C (HCV) infection often remain asymptomatic for decades and even for life, but a proportion of them develop active chronic hepatitis, which is a progressive disease (Thein et al. 2008). Because of the largely asymptomatic nature of HCV infection, notification data reflect national screening and testing practices and do not give accurate insights into the prevalence of HCV infection. Although there are studies that have estimated the prevalence of HCV antibodies (anti-HCV) in specific groups such as the general population, first-time blood donors, or people who inject drugs (PWID) at drug treatment centers, those alone cannot be combined to produce national estimates unless some additional information regarding the composition of each HCV risk group and its prevalence in the population are known (Sweeting et al. 2008; McDonald et al. 2014; Tan et al. 2018).

# METHODS

Bayesian multiparameter evidence synthesis (BMES) has been a popular approach to formally estimate anti-HCV or HIV prevalence (Sweeting et al. 2008; McDonald et al. 2014; Tan et al. 2018; Presanis et al. 2010; Veen et al. 2011). In this project, we extend this method to estimating the CHC prevalence, with the population (15-79 years) split into three main non-overlapping risk groups, i.e., recent PWID (those who have injected in the last year), ex-PWID, and non-PWID. A unified model is assumed including parameters associated with the CHC prevalence of recent, ex-PWID, and non-PWID (denoted by $\pi_{rec}$, $\pi_{ex}$, and $\pi_{non}$, respectively) and the prevalence of these risk groups in the population (denoted by $\rho_{rec}$, $\rho_{ex}$, and $\rho_{non}$, respectively; $\rho_{rec} + \rho_{ex} + \rho_{non} = 1$). To estimate the CHC prevalence in the whole population, $\pi$, we used the formula:

$$\pi = \pi_{rec}\rho_{rec} + \pi_{ex}\rho_{ex} + \pi_{non}\rho_{non} \quad (1)$$

Bayesian synthesis considers all available data, allowing for an evidence-based estimation of CHC prevalence in the population, with inherent uncertainty properly accounted for. Our aim is to estimate the CHC prevalence for the total (15-79) population of Slovakia in 2019 using sources of information over the last decade (2010-2019).

## Prevalence of ex-PWID

Data on the prevalence of ex-PWID are sparse and generally often unreliable. To overcome this limitation, we apply the method proposed by McDonald et al. (2014), which requires simulating a multi-state Markov model representing the non-PWID, recent PWID, and ex-PWID. In the multi-state Markov model, the simulation starts in 1950 and examines the population aged 15-79 years old. A certain number of 15-year-old individuals enter the simulation process each year. These numbers of 15-year-old individuals are obtained from the United Nations Statistic Division UNSD, being different for each country. The ageing of the population is also taken into account. Country-specific overall mortality data are retrieved from the Life Tables of the World Health Organization WHO. The duration of injecting career is also country-specific and obtained from the paper of Trickey et al. (2019).

To estimate the number of ex-PWID, the model can be calibrated on the number of recent PWID provided in the EMCDDA barometer or the EMCDDA statistical bulletin (the code of the Markov model can be found in the Appendix). If there are no available data in the barometer, estimates from the systematic review of Grebely et al. (2019) could be used. Currently, the multi-state Markov model was calibrated on the estimated prevalence of recent PWID reported in the systematic review of Grebely et al. (2019).

After applying the model for Slovakia, we compute the number (and the corresponding Confidence Interval - CI) of ex-PWID in 2019. The number of ex-PWID is then divided by the population size (15-79 years) in the same year, and a Normal distribution, constrained such that $\rho_{ex} \in (0,1)$, is assumed for $\rho_{ex}$. The standard deviation of this distribution is specified to approximately correspond to the respective CI, i.e. $\left(UB_{\rho_{ex}} - LB_{\rho_{ex}}\right)/(2 \times 1.96)$, where $UB_{\rho_{ex}}$ and $LB_{\rho_{ex}}$ denote the upper and lower limit of the CI.

## Prevalence of recent PWID

The number of recent PWID in Slovakia in 2019 is also projected by the multi-state Markov model described in the previous subsection. To estimate $\rho_{rec}$, the number of recent PWID is divided by the population size (15-79 years). Similar to the previous subsection, a Normal distribution, constrained such that $\rho_{rec} \in (0,1)$, is assumed for $\rho_{rec}$, with the standard deviation specified to correspond to the CI obtained from the multi-state Markov model.

## Prevalence of CHC among recent PWID

The prevalence (and the 95% CI) of CHC among recent PWID was informed by CHC prevalence data from the paper of Grebely et al. (2019) in 2008 and 2014. However, some people may have been treated with direct-acting antivirals (DAAs), with the sustained virologic response ($SVR_{PWID}$) among PWID estimated to be 88% (95% CI: 80% to 93%) (Graf et al. 2019). Based on the information provided by the national focal point, the number of individuals treated with DAAs up to 2019 is equal to 380.

After personal communication with the focal points, it was mentioned that treatment can be initiated as long as a CHC-positive individual is at least 1 year free of drugs. Therefore, in this report, we assume that the proportion of recent PWID among individuals treated with DAAs, denoted by $\rho_{rec|DAA}$, is equal to zero, and thus, a treatment adjustment for the CHC prevalence among recent PWID was not performed. The proportion of ex- and non-PWID among those treated with DAAs, denoted by $\rho_{ex|DAA}$ and $\rho_{non|DAA}$, respectively, were assumed to be proportional to the corresponding proportions of ex- and non-PWID among CHC-positive individuals, i.e. $\rho_{ex|DAA} = \frac{\text{Pr(Ex-PWID|CHC)}}{\text{Pr(Ex-PWID|CHC)+Pr(Non-PWID|CHC)}}$ and $\rho_{non|DAA} = \frac{\text{Pr(Non-PWID|CHC)}}{\text{Pr(Ex-PWID|CHC)+Pr(Non-PWID|CHC)}}$, as estimated by our model when the DAA uptake is ignored (Table 2).

## Prevalence of CHC among ex-PWID

Information on CHC prevalence among ex-PWID (i.e., $\pi_{ex}$) is difficult to obtain directly. To overcome this, anti-HCV prevalence data from ever PWID in Bratislava in 2019, available in the EMCDDA statistical bulletin, were used. However, as these EMCDDA refer to the anti-HCV prevalence and not to the CHC prevalence, they cannot be used directly. In the absence of treatment, this issue could be addressed by noting that spontaneous viral clearance occurs in approximately one in four people with acute hepatitis C (Micallef, Kaldor, and Dore 2006). To account for the variability of the HCV clearance probabilities, we used the result reported in Micallef, Kaldor, and Dore (2006), i.e., the proportion of HCV clearance (denoted by $\rho_{clear}$) is equal to 0.26 (95% CI 0.22–0.29). Thus, estimates of the CHC prevalence among ever PWID ($\pi_{ever}$) could be obtained using the formula $\pi_{ever} = \pi(\text{anti-HCV})_{ever}(1 - \rho_{clear})$, where

$\pi(\text{anti-HCV})_{ever}$ denotes the anti-HCV prevalence among ever PWID. In any case, once an estimate of $\pi_{ever}$ is available, $\pi_{ex}$ can be indirectly estimated since $\pi_{ever}$ is equal to a weighted average of $\pi_{rec}$ and $\pi_{ex}$, as shown by the following formula

$$\pi_{ever} = \frac{\rho_{rec}}{\rho_{rec} + \rho_{ex}} \pi_{rec} + \frac{\rho_{ex}}{\rho_{rec} + \rho_{ex}} \pi_{ex}, \quad (2)$$

or, equivalently,

$$\pi_{ex} = \left\{ \pi_{ever} - \frac{\rho_{rec}}{\rho_{rec} + \rho_{ex}} \pi_{rec} \right\} \times \frac{\rho_{rec} + \rho_{ex}}{\rho_{ex}}, \quad (3)$$

However, some ex-PWID may have been treated with DAAs. To adjust the CHC prevalence among ex-PWID for DAAs, one can use the formula

$$\pi_{ex} = \frac{N_{15,79}\rho_{ex}\tilde{\pi}_{ex} - N_{DAA}\rho_{ex|DAA}SVR_{PWID}}{N_{15,79}\rho_{ex}}. \quad (4)$$

where $\tilde{\pi}_{ex}$ denotes the CHC prevalence estimate among ex-PWID when the contribution of DAAs is ignored. Recall also that the proportion of ex-PWID among individuals treated with DAAs, i.e. $\rho_{ex|DAA}$, is assumed to be proportional to the corresponding proportions of ex- and non-PWID among CHC-positive individuals, i.e. $\rho_{ex|DAA} = \frac{\Pr(\text{Ex-PWID}|\text{CHC})}{\Pr(\text{Ex-PWID}|\text{CHC}) + \Pr(\text{Non-PWID}|\text{CHC})}$, as estimated by our model when the DAA uptake is ignored (Table 2).

## Prevalence of CHC among non-PWID

To estimate the CHC prevalence among non-PWID in a country (after 2010), the ECDC database (European Centre for Disease Prevention and Control (ECDC) 2021) was used. The ECDC group has carefully and critically collected information from relevant studies across the EU/EEA region in cooperation with the focal points of each country. On the basis of the representativeness of each study and other factors (European Centre for Disease Prevention and Control (ECDC) 2016), a risk of bias score was assigned to each study, with higher values indicating higher-quality studies. When higher-quality studies are available in a country (≥ 4), they are used to estimate the overall CHC prevalence in that country among non-PWID. If there are no higher-quality studies, general population estimates with a lower quality (<4) are pooled. If no general population prevalence estimates are available, data on the prevalence of

HCV in pregnant women could be used as a prevalence measure. Finally, if data on pregnant women are lacking too, first-time blood donor studies could be an option. However, if the national focal point agrees or recommends, estimates from a neighboring country could be used instead, or we could use any other relevant information provided/suggested by the national focal point.

In Slovakia, there was 1 study in the general population of high quality, which included only anti-HCV data (Immunological overview in the Slovak republic in 2018). Thus, individuals treated with DAAs should be removed, with the sustained virologic response ($SVR$) in the general population estimated to be 96.7% (95% CI: 95.4% to 98.1%) (Lampertico et al. 2020). Similarly to the procedure described in the previous subsections, the CHC prevalence among non-PWID, adjusted for DAAs and the spontaneous HCV clearance probabilities, can be estimated by

$$\pi_{non} = \frac{N_{15,79}\rho_{non}\pi(\text{anti-HCV})_{non}(1-\rho_{clear}) - N_{DAA}\rho_{non|DAA}SVR}{N_{15,79}\rho_{non}}. \quad (5)$$

where $\pi(\text{anti-HCV})_{non}$ denotes the anti-HCV prevalence estimate based solely on the Immunological overview in the Slovak republic in 2018.

As $\rho_{rec}$ and $\rho_{ex}$ are likely to be low, $\pi_{non}$ will be the most critical factor for the estimation of the overall CHC prevalence in the population. To adjust for potential heterogeneity between the CHC prevalence estimates from different studies in the non-PWID population in a country, as a sensitivity analysis, we also apply a random-effect meta-analytic approach to pool the CHC prevalence across studies on non-PWID (Lin and Chu 2020). The random-effect approach is carried out only when at least 3 studies are available in the general population of a certain country, and the estimates are compared with the corresponding estimates from the approach assuming no heterogeneity (similar to a fixed-effect meta-analysis). If the estimates are inconsistent, possible explanations are investigated and discussed. If there is substantial heterogeneity between studies, it would help more to explore the causes; for example, following national focal point suggestions, some studies could be excluded to reduce heterogeneity.

## Sensitivity analyses

A sensitivity analysis including additional populations as separate risk groups could be applied. As discussed with the national focal points, migrants are not an issue in Slovakia. The Roma population, which accounts for about 8% of the population in Slovakia, could be an issue. However, as the CHC prevalence is very low in this population (Veseliny et al. 2014), which is probably due to very low intravenous drug use, we did not perform a sensitivity analysis including Roma as a separate risk group.

# RESULTS

The above-mentioned approach was used to estimate the prevalence of CHC in 2019 (considering ages between 15-79 years: 4,412,866).

The aggregated data used by our approach are briefly presented in Table 1. The multi-state Markov model was calibrated on the estimated prevalence of recent PWID reported in the systematic review of Grebely et al. (2019). In Slovakia, there was 1 study in the general population of high quality, which included only anti-HCV data (Immunological overview in the Slovak republic in 2018). To estimate the CHC prevalence in the general population (primarily non-PWID), we used a fixed-effect meta-analytic approach, with the corresponding results, when information on DAAs is ignored, presented in Table 2. However, based on data provided by the focal point, approximately 380 individuals were treated with DAAs from 2017 to 2019 in Slovakia, with the proportion of ex-PWID and non-PWID among the 380 treated individuals assumed to be equal to $\frac{\text{Pr(Ex-PWID|CHC)}}{\text{Pr(Non-PWID|CHC)+Pr(Ex-PWID|CHC)}} \approx 81.31\%$ (95%CI: 62.52%-92.95%) and $\frac{\text{Pr(Non-PWID|CHC)}}{\text{Pr(Non-PWID|CHC)+Pr(Ex-PWID|CHC)}} \approx 18.69\%$ (95% CI: 7.05%-37.48%), respectively (Table 2).

The corresponding results accounting for the DAA uptake are presented in Table 3. The prevalence of recent and ex-PWID was low in Slovakia (about 0.45% and 0.97%, respectively) corresponding to 19,645 (95% CI: 18,700-20,350) recent PWID and 42,950 (95% CI: 41,700-44,050) ex-PWID in the population. However, the CHC prevalence in these groups was substantial (Table 3) being 42.21% and 35.93%, respectively. This translates to 8,302 (95% CI: 5,201-11,342) and 15,421 (95% CI: 8,707-22,074) recent and ex-PWID aged 15-79 living with CHC infection in Slovakia in 2019. The CHC prevalence in the general population was 0.08%

(95% CI: 0.03%-0.18%), much lower than that of the high-risk groups. Taking all pieces of information into account, the overall CHC prevalence in Slovakia in 2019 was equal to 0.62% (95% CI: 0.47%-0.78%), which corresponds to 27,407 (95% CI: 20,658-34,501) individuals aged 15-79 years with CHC infection.

# TABLES AND FIGURES

*Table 1. Data (real) contributing to the estimation of CHC prevalence in Slovakia in 2019.*

| Parameter | Estimate (95%CI) | Number (95%CI) | Numerator | Denominator | Notes | Year of study |
|---|---|---|---|---|---|---|
| $\rho_{rec}$ | | 19,645 (18,700-20,350) | | | Method based on McDonald et al. | 2019 |
| $\rho_{ex}$ | | 42,950 (41,700-44,050) | | | Method based on McDonald et al. | 2019 |
| $\pi_{rec}$ | 42.1% (26.6%-57.7%) | | | | Grebely et al. | 2008 and 2014 |
| $\pi(\text{anti-HCV})_{ever}$ | | | 29 | 56 | EMCDDA database | 2019 |
| $\pi(\text{anti-HCV})_{non}$ | | | 4 | 4,215 | Country feedback document (high-quality study) | 2018 |

*Notes*: Although it looks counter-intuitive, **a higher risk of bias score denotes a higher-quality study** (range from 0 to 6);

*Table 2. Results from the method assuming no heterogeneity in the estimation of the CHC prevalence in the general population from different studies (i.e., similar to a fixed-effect meta-analysis).*

| Parameter | Posterior median | Posterior sd | LB (2.5%) | UB (97.5%) |
|---|---|---|---|---|
| $\rho_{rec}$ (%) | 0.45 | 0.01 | 0.43 | 0.46 |
| $\rho_{ex}$ (%) | 0.97 | 0.01 | 0.95 | 1 |
| $\pi_{rec}$ (%) | 41.98 | 7.92 | 26.51 | 57.72 |
| $\pi_{ex}$ (%) | 36.59 | 8.14 | 20.98 | 52.65 |
| $\pi_{non}$ (%) | 0.08 | 0.04 | 0.03 | 0.18 |
| $\pi$ (%) | 0.63 | 0.08 | 0.48 | 0.79 |
| Number with CHC | 27,708 | 3,568 | 21,001 | 35,012 |
| Pr(Recent PWID\|CHC) (%) | 29.71 | 7.02 | 17.76 | 45.2 |
| Pr(Ex-PWID\|CHC) (%) | 56.67 | 8.38 | 38.47 | 71.23 |
| Pr(Non-PWID\|CHC) (%) | 12.99 | 5.38 | 4.92 | 25.65 |

*Notes*: Information on DAAs is not taken into account.

*Abbreviations*: $\rho_{rec}$, prevalence of recent PWID (proportion of the population that is recent PWID); $\rho_{ex}$, prevalence of ex-PWID (proportion of the population that is ex-PWID); $\pi_{rec}$, CHC prevalence among recent PWID; $\pi_{ex}$, CHC prevalence among ex-PWID; $\pi_{non}$, CHC prevalence among non-PWID; $\pi$ overall CHC prevalence in Slovakia; LB, Lower Boundary; UP, Upper Boundary

*Table 3. Results from the method assuming no heterogeneity in the estimation of the CHC prevalence in the general population from different studies (i.e., similar to a fixed-effect meta-analysis).*

| Parameter | Posterior median | Posterior sd | LB (2.5%) | UB (97.5%) |
|---|---|---|---|---|
| $\rho_{rec}$ (%) | 0.45 | 0.01 | 0.43 | 0.46 |
| $\rho_{ex}$ (%) | 0.97 | 0.01 | 0.95 | 1 |
| $\pi_{rec}$ (%) | 42.21 | 7.98 | 26.55 | 57.67 |
| $\pi_{ex}$ (%) | 35.93 | 8.03 | 20.24 | 51.4 |
| $\pi_{non}$ (%) | 0.08 | 0.04 | 0.03 | 0.18 |
| $\pi$ (%) | 0.62 | 0.08 | 0.47 | 0.78 |
| Number with CHC | 27,407 | 3,545 | 20,658 | 34,501 |
| Pr(Recent PWID\|CHC) (%) | 30.2 | 7.2 | 18.18 | 46.37 |
| Pr(Ex-PWID\|CHC) (%) | 56.21 | 8.44 | 37.9 | 71.01 |
| Pr(Non-PWID\|CHC) (%) | 12.88 | 5.32 | 4.82 | 25.31 |

*Notes*: The number of individuals treated with DAAs is taken into account.

*Abbreviations*: $\rho_{rec}$, prevalence of recent PWID (proportion of the population that is recent PWID); $\rho_{ex}$, prevalence of ex-PWID (proportion of the population that is ex-PWID); $\pi_{rec}$, CHC prevalence among recent PWID; $\pi_{ex}$, CHC prevalence among ex-PWID; $\pi_{non}$, CHC prevalence among non-PWID; $\pi$ overall CHC prevalence in Slovakia; LB, Lower Boundary; UP, Upper Boundary

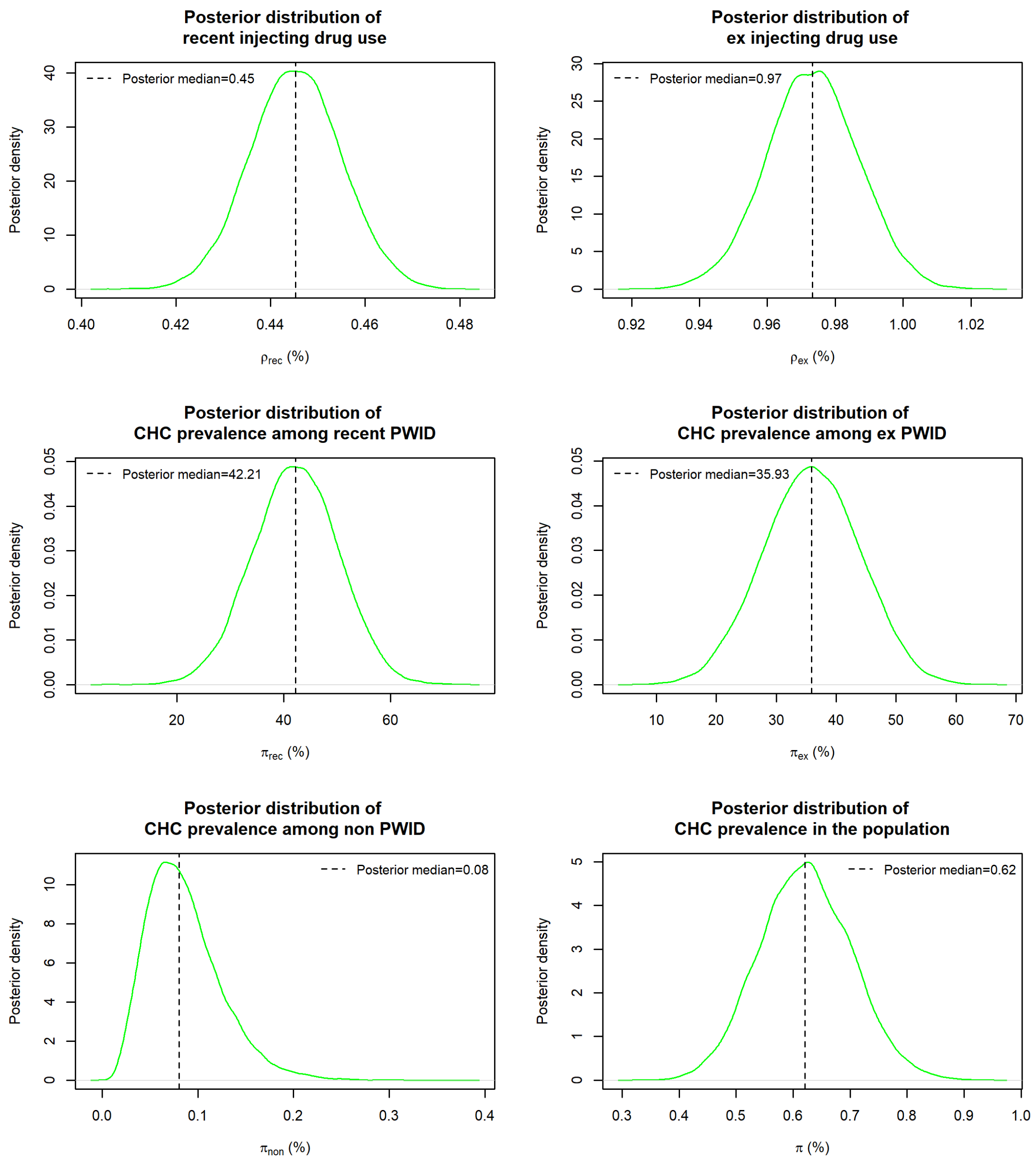


*Figure 1. Posterior distributions of the key model parameters using the approach that assumes no heterogeneity in the estimation of the CHC prevalence in the general population (primarily non-PWID) from different studies (i.e., similar to a fixed-effect meta-analysis), with the number of individuals treated with DAA taken into account.*

# APPENDIX

## Fit of the multi-state Markov model

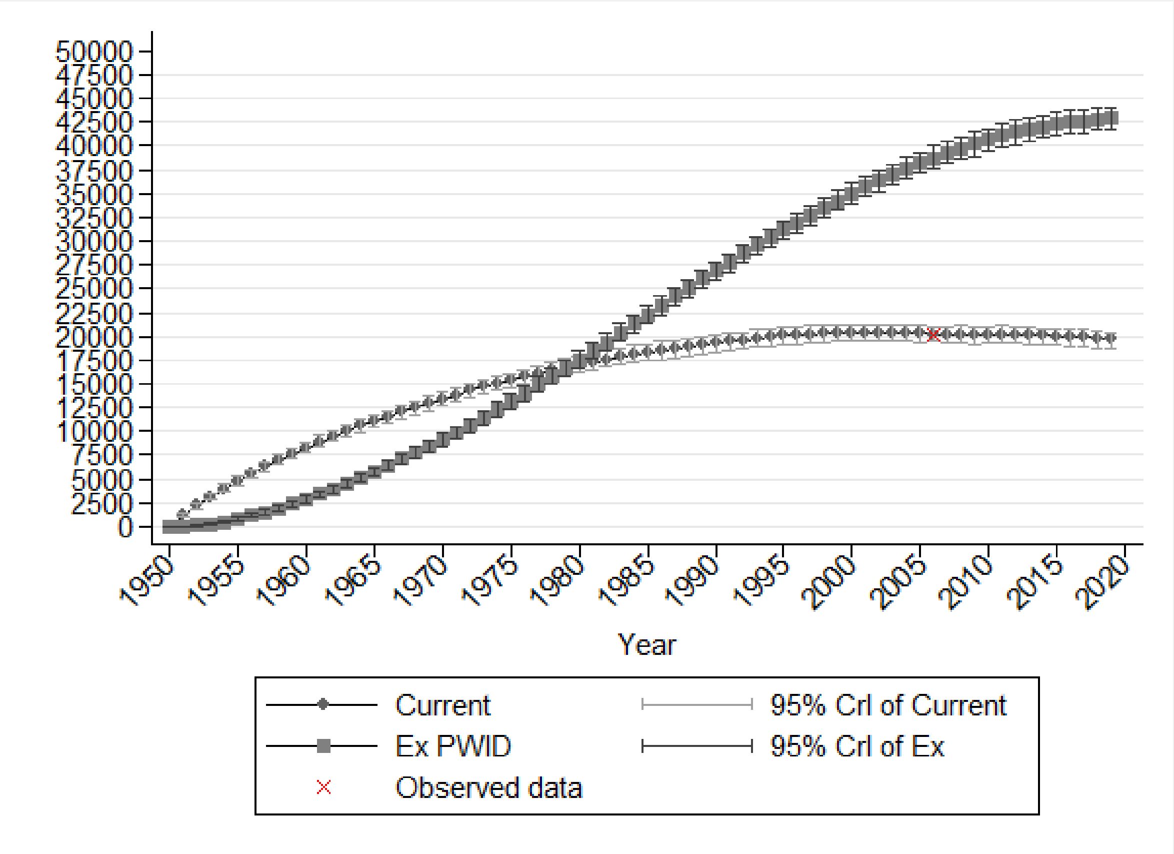


*Figure 2: Model predictions for the numbers of current and ex-PWID (people who inject drugs). The solid black line and shaded grey error bars show the median and 95% credible intervals (95% CrI) for the model projections. For comparison, asterisks indicate the observed country's data.*

## Stan code for Bayesian multiparameter evidence synthesis

```
data {
  int<lower=0> N1579; // Population of 15-79 in `Country`
  int<lower=1> Knon; // Number of studies in the ECDC data for `Country`
  int<lower=1> Kever; // Number of studies in the EMCDDA data for ever us
ers in `Country`
  int<lower=1> Kcur; // Number of studies in the EMCDDA barometer (for HC
V prevalence of PWID) in `Country`
  int<lower=1> NDAA; // Total number of DAAs from 2016 to 2019

  real p_cur_mean; // Prior mean for the prevalence of current use in `Co
untry`
  real<lower=0> p_cur_sd; // Prior sd for the prevalence of current use i
n `Country`

  real p_ex_mean; // Prior mean for the prevalence of ex-use in `Country`
  real<lower=0> p_ex_sd; // Prior sd for the prevalence of ex-use in `Cou
ntry`

  //int<lower=0> Nst_CHC_cur[Kcur]; // Number of individuals in the study
estimating chronic HCV among recent PWID
  //int<lower=0> Yst_CHC_cur[Kcur]; // Number of HCV+ in the study estima
ting chronic HCV among recent PWID
  real p_CHC_cur_mean; // Prior mean for the CHC prevalence among recent
PWID in `Country`
  real<lower=0> p_CHC_cur_sd; // Prior sd for the CHC prevalence among re
cent PWID in `Country`

  int<lower=0> Nst_hcv_ever[Kever]; // Number of individuals in the study
estimating anti-HCV among ever IDU
  int<lower=0> Yst_hcv_ever[Kever]; // Number of HCV+ in the study estima
ting anti-HCV among ever PWID
```

```
  int<lower=0> Nst_hcv_non[Knon]; // Number of individuals in the study e
stimating anti-HCV among non PWID
  int<lower=0> Yst_hcv_non[Knon]; // Number of HCV+ in the study estimati
ng anti-HCV among non PWID

  vector<lower=0>[3] alpha; // parameter of the Diriclet prior

  real HCVclear_mean; // Prior mean for the HCV clearance probability
  real<lower=0> HCVclear_sd; // Prior sd for the HCV clearance probabilit
y

  real SVR_mean; // Prior mean for the SVR among non-PWID
  real<lower=0> SVR_sd; // Prior sd for the SVR among non-PWID

  real SVR_PWID_mean; // Prior mean for the SVR among PWID
  real<lower=0> SVR_PWID_sd; // Prior sd for the SVR among PWID
}

// Block defining the original parameters
parameters {
  // The parameters to be sampled
  simplex[3] rho; // Prevalence of the three risk groups
  real<lower=0,upper=1> CHCpi_cur; // P(CHC+|Cur)
  real<lower=0,upper=1> CHCpi_ex; // P(CHC+|Ex)
  real<lower=0,upper=1> CHCpi_non; // P(CHC+|Non)
  real<lower=0,upper=1> SVR; // SVR of DAAs among non-PWID
  real<lower=0,upper=1> SVR_PWID; // SVR of DAAs among PWID

  real<lower=0,upper=1-CHCpi_cur> HCVclear; // Probability of HCV clearan
ce; upper bound = 1-prevalence of chronic HCV
}
```

```
transformed parameters {
  // Change scales
  real<lower=0,upper=1> rho_ever;
  real<lower=0,upper=1> CHCpi_ever;
  real<lower=0,upper=1> pi_ever;
  real<lower=0,upper=1> pi_non;

  rho_ever = rho[1] + rho[2];
  CHCpi_ever = CHCpi_cur*rho[1]/rho_ever + CHCpi_ex*rho[2]/rho_ever;
  pi_ever = CHCpi_ever/(1-HCVclear);
  pi_non = CHCpi_non/(1-HCVclear);
}


// Binomial regression model
model {
  // Priors
  rho ~ dirichlet(alpha);

  ///////////////////////////////
  // Likelihood contributions //
  ///////////////////////////////

  // Probability of HCV clearance
  HCVclear ~ normal(HCVclear_mean,HCVclear_sd);

  // Prevalence of current use
  rho[1] ~ normal(p_cur_mean,p_cur_sd);

  // Prevalence of ex-use
```

```
  rho[2] ~ normal(p_ex_mean,p_ex_sd);

  // Prevalence of chronic HCV among current users
  //Yst_CHC_cur ~ binomial(Nst_CHC_cur,CHCpi_cur);
  CHCpi_cur ~ normal(p_CHC_cur_mean,p_CHC_cur_sd);

  // Prevalence of HCV among ever users
  Yst_hcv_ever ~ binomial(Nst_hcv_ever,pi_ever);

  // HCV+ among non
  Yst_hcv_non ~ binomial(Nst_hcv_non,pi_non);

  // SVR of DAAs among non-PWID
  SVR ~ normal(SVR_mean,SVR_sd);

  // SVR of DAAs among PWID
  SVR_PWID ~ normal(SVR_PWID_mean,SVR_PWID_sd);
}


generated quantities {
  // Functions of parameters
  real<lower=0,upper=100> overalCHC;
  real logit_CHCpi_cur;
  real logit_CHCpi_ex;
  real logit_CHCpi_non;
  real logit_rho_cur;
  real logit_rho_ex;
  real logit_rho_non;
  real<lower=0,upper=1> pEverGivenCHC;
  real<lower=0,upper=1> pCurGivenCHC;
  real<lower=0,upper=1> pExGivenCHC;
```

```
  real<lower=0,upper=1> pNonGivenCHC;
  real logit_HCVclear;
  real<lower=0> NumberCHC;

  real<lower=0,upper=1> CHCDAApi_non; // P(CHC+|Non) adjusted for DAA
  real<lower=0,upper=1> CHCDAApi_cur; // P(CHC+|Cur) adjusted for DAA
  real<lower=0,upper=1> CHCDAApi_ex; // P(CHC+|Ex) adjusted for DAA
  real<lower=0,upper=1> CHCDAApi_ever; // P(CHC+|Ever) adjusted for DAA

  real<lower=0,upper=100> overalCHC_DAA;
  real pEverGivenCHC_DAA;
  real pCurGivenCHC_DAA;
  real pExGivenCHC_DAA;
  real pNonGivenCHC_DAA;
  real<lower=0> NumberCHC_DAA;

  // Overall HCV prevalence
  overalCHC = 100*(rho[1]*CHCpi_cur + rho[2]*CHCpi_ex +  rho[3]*CHCpi_non
);
  pEverGivenCHC = CHCpi_ever*rho_ever/(overalCHC/100);
  pCurGivenCHC = CHCpi_cur*rho[1]/(overalCHC/100);
  pExGivenCHC = CHCpi_ex*rho[2]/(overalCHC/100);
  pNonGivenCHC = CHCpi_non*(1-rho_ever)/(overalCHC/100);
  NumberCHC = round(overalCHC*N1579/100);

  CHCDAApi_non = ( N1579*rho[3]*CHCpi_non - NDAA*(pNonGivenCHC/(pNonGiven
CHC+pExGivenCHC))*SVR)/(N1579*rho[3]);
  CHCDAApi_cur = ( N1579*rho[1]*CHCpi_cur - NDAA*0*SVR_PWID)/(N1579*rho[1
]);
  CHCDAApi_ex = ( N1579*rho[2]*CHCpi_ex - NDAA*(pExGivenCHC/(pNonGivenCHC
+pExGivenCHC))*SVR_PWID)/(N1579*rho[2]);
  CHCDAApi_ever = CHCDAApi_cur*rho[1]/rho_ever + CHCDAApi_ex*rho[2]/rho_e
```

```
ver;

  overalCHC_DAA = 100*(rho[1]*CHCDAApi_cur + rho[2]*CHCDAApi_ex + rho[3]*
CHCDAApi_non);
  pEverGivenCHC_DAA = CHCDAApi_ever*rho_ever/(overalCHC_DAA/100);
  pCurGivenCHC_DAA = CHCDAApi_cur*rho[1]/(overalCHC_DAA/100);
  pExGivenCHC_DAA = CHCDAApi_ex*rho[2]/(overalCHC_DAA/100);
  pNonGivenCHC_DAA = CHCDAApi_non*(1-rho_ever)/(overalCHC_DAA/100);
  NumberCHC_DAA = round(overalCHC_DAA*N1579/100);

  logit_rho_cur = logit(rho[1]);
  logit_rho_ex = logit(rho[2]);
  logit_rho_non = logit(rho[3]);
  logit_CHCpi_cur = logit(CHCpi_cur);
  logit_CHCpi_ex = logit(CHCpi_ex);
  logit_CHCpi_non = logit(CHCpi_non);
  logit_HCVclear = logit(HCVclear);
}
```

## Multi-state Markov model

```
#include <stdio.h>
#include <stdlib.h>
#include <string.h>
#include <math.h>
#include <time.h>

#define loops 100
int populationPerYearAndAge[2021][66];
double deathRatePerAge[66];
int totalPopulationPerYear[2021];

FILE *F_DeathRate=fopen("greece-deathRates.txt","r");
FILE *F_Population=fopen("greece-population.txt","r");
FILE *out;

double pa_start = 0.00012;                    // Setting Variable
double pa_step  = 0.000002;                   // Setting Variable
double pa_stop  = 0.000123;                   // Setting Variable

double pa;
double pg = 1.0/11.7;   // Assumed average active injecting career of  11
.7 years
double pk = 0.004*12.0;     // Relapse rate of 0.004/month

struct people{
    int age;
    int state;
};

char filename[100];
struct people person[50000000];
```

```
long int totalPersons=0;

void setPopulationAge()
{
    for( int j=0; j<71; j++){
        for( int i=0; i<51; i++){
            fscanf(F_Population,"%d",&populationPerYearAndAge[1950+j][15+
i]);
        }
    }
}

void setDeathRate()
{
    for( int i=0; i<51; i++){
        fscanf(F_DeathRate,"%lf",&deathRatePerAge[15+i*5]);
        for( int j=0; j<5; j++){
            deathRatePerAge[15+i*5+j] = deathRatePerAge[15+i*5];
        }
    }
}

void getTotalPopulationPerAge()
{
    for( int j=0; j<71; j++){
        for( int i=0; i<51; i++){
            totalPopulationPerYear[1950+j] += populationPerYearAndAge[195
0+j][15+i];
        }
    }
}
```

```
void printTotalPopulationPerAge()
{
    printf("---- - ----------\n");
    printf("Year - Population\n");
    printf("---- - ----------\n");
    for( int i=1950; i<2021; i++){
        printf("%d - %d\n",i,totalPopulationPerYear[i]);
    }
}

void printDeathRatePerAge()
{
    printf("--- - -----\n");
    printf("Age - Rate\n");
    printf("--- - -----\n");
    for( int i=15; i<65; i++){
        printf("%d - %lf\n",i,deathRatePerAge[i]);
    }
}

void printTotalPersonPerState( int year)
{
    int count[4]= {0,0,0,0};
    for( int i=0; i<totalPersons; i++){
        count[person[i].state]++;
    }
    //printf("%d\t%ld\t%ld\t%ld\t%ld\n",year,count[0],count[1],count[2],c
ount[3]);
    if(year==2014)
        printf("%d\t%ld\t%ld\t%ld\t%ld\t%lf\n",year,count[0],count[1],cou
nt[2],count[3],pa);
    if( year>2009){
```

```
        fprintf(out, "%d\t%ld\t%ld\t%ld\t%ld\n",year,count[0],count[1],co
unt[2],count[3]);
    }
}

void initializePopulation()
{
    int cnt=0;
    for( int j=15; j<65; j++){
        for( int i=0; i<populationPerYearAndAge[1950][j]*100; i++){
            person[cnt].age=j;
            person[cnt].state=0;
            cnt++;
        }
    }
    totalPersons = cnt;
}

bool checkRate(double rate)
{
    if (rate <0.001){
        double k = rand()/(1.00+RAND_MAX);
        if(k<0.001){
            rate = rate*1000;
        }
        else{
            return false;
        }
    }
    double k = rand()/(1.00+RAND_MAX);
    if ( k < rate )
        return true;
```

```
    else
        return false;
}

void changeStatusAndAge()
{
    for( int i=0; i<totalPersons; i++){
        // change state
        double k = rand()/(1.00+RAND_MAX);
        if( person[i].state==0){
            if ( checkRate(pa) &&  person[i].age<40){
                person[i].state=1;
            }
        }
        else if( person[i].state==1){
            if ( k < pg ){
                person[i].state=2;
            }
        }
        else if( person[i].state==2){
            if ( k < pk ){
                person[i].state=1;
            }
        }
        // After their 49th year, all active PWID are assumed to cease in
jecting
        if(person[i].age>49 && person[i].state==1){
            person[i].state=2;
        }
        // After their 64th year, remove
        if(person[i].age>64){
            person[i].state=3;
```

```
        }
        // death rate depending on age and state
        k = rand()/(1.00+RAND_MAX);
        if( person[i].state==0){
            if ( k < deathRatePerAge[person[i].age] ){
                person[i].state=3;
            }
        }
        else if( person[i].state==1){
            if ( k < deathRatePerAge[person[i].age] * 13.0 ){
                person[i].state=3;
            }
        }
        else if( person[i].state==2){
            if ( k < deathRatePerAge[person[i].age] ){
                person[i].state=3;
            }
        }
        // increase age one year
        person[i].age++;
    }
}

void addNewPersons(int year)
{
    // add new person [15 years old and non PWID]
    int cnt=totalPersons;
    for( int i=0; i<populationPerYearAndAge[year+1][15]*100; i++){
// 100 instead of 1000 for the population to reduce execution time
        person[cnt].age=15;
        person[cnt].state=0;
        cnt++;
```

```
    }
    // increase the total number of population
    totalPersons = cnt;
}
int main()
{
    srand(time(NULL));

    setPopulationAge();
    setDeathRate();

    getTotalPopulationPerAge();
    pa = pa_start;
    while ( pa < pa_stop){

        snprintf(filename, 100, "result_%lf.txt",pa);
        out=fopen(filename,"w");

        for( int iter=0; iter<loops ;iter++){
            initializePopulation();
            for( int year=1950; year<2020; year++){
                printTotalPersonPerState(year);
                changeStatusAndAge();
                addNewPersons(year);
            }
        }

        fclose(out);
        pa = pa + pa_step;
    }
```

```
}
```

# REFERENCES

European Centre for Disease Prevention and Control (ECDC). 2016. "Systematic Review on Hepatitis b and c Prevalence in the EU/EEA." https://www.ecdc.europa.eu/sites/default/files/media/en/publications/Publications/systematic-review-hepatitis-B-C-prevalence.pdf.

———. 2021. "Hepatitis c Prevalence Database." https://www.ecdc.europa.eu/en/all-topics-z/hepatitis-c/tools/hepatitis-c-prevalence-database.

Graf, Christiana, Marcus M Mücke, Georg Dultz, Kai-Henrik Peiffer, Alica Kubesch, Patrick Ingiliz, Stefan Zeuzem, Eva Herrmann, and Johannes Vermehren. 2019. "Efficacy of Direct-acting Antivirals for Chronic Hepatitis C Virus Infection in People Who Inject Drugs or Receive Opioid Substitution Therapy: A Systematic Review and Meta-analysis." *Clinical Infectious Diseases* 70 (11): 2355–65. https://doi.org/10.1093/cid/ciz696.

Grebely, Jason, Sarah Larney, Amy Peacock, Samantha Colledge, Janni Leung, Matthew Hickman, Peter Vickerman, et al. 2019. "Global, Regional, and Country-Level Estimates of Hepatitis c Infection Among People Who Have Recently Injected Drugs." *Addiction* 114 (1): 150–66. https://doi.org/https://doi.org/10.1111/add.14393.

Lampertico, Pietro, Jose A. Carrión, Michael Curry, Juan Turnes, Markus Cornberg, Francesco Negro, Ashley Brown, et al. 2020. "Real-World Effectiveness and Safety of Glecaprevir/Pibrentasvir for the Treatment of Patients with Chronic HCV Infection: A meta-Analysis." *Journal of Hepatology* 72 (6): 1112–21. https://doi.org/10.1016/j.jhep.2020.01.025.

Lin, Lifeng, and Haitao Chu. 2020. "Meta-Analysis of Proportions Using Generalized Linear Mixed Models." *Epidemiology* 31 (5). https://journals.lww.com/epidem/Fulltext/2020/09000/Meta_analysis_of_Proportions_Using_Generalized.16.aspx.

McDonald, Scott A., Rosmawati Mohamed, Maznah Dahlui, Herlianna Naning, and Adeeba Kamarulzaman. 2014. "Bridging the Data Gaps in the Epidemiology of Hepatitis c Virus Infection

in Malaysia Using Multi-Parameter Evidence Synthesis." *BMC Infectious Diseases* 14 (1): 564. https://doi.org/10.1186/s12879-014-0564-6.

Micallef, J. M., J. M. Kaldor, and G. J. Dore. 2006. "Spontaneous Viral Clearance Following Acute Hepatitis c Infection: A Systematic Review of Longitudinal Studies." *Journal of Viral Hepatitis* 13 (1): 34–41. https://doi.org/https://doi.org/10.1111/j.1365-2893.2005.00651.x.

Presanis, Anne M., O. Noel Gill, Timothy R. Chadborn, Caterina Hill, Vivian Hope, Louise Logan, Brian D. Rice, Valerie C. Delpech, A. E. Ades, and Daniela De Angelis. 2010. "Insights into the Rise in HIV Infections, 2001 to 2008: A Bayesian Synthesis of Prevalence Evidence." *AIDS* 24 (18). https://journals.lww.com/aidsonline/Fulltext/2010/11270/Insights_into_the_rise_in_HIV_infections,_2001_to.12.aspx.

Sweeting, M. J., D. De Angelis, M. Hickman, and A. E. Ades. 2008. "Estimating hepatitis C prevalence in England and Wales by synthesizing evidence from multiple data sources. Assessing data conflict and model fit." *Biostatistics* 9 (4): 715–34. https://doi.org/10.1093/biostatistics/kxn004.

Tan, Sarah, Susanna Makela, Daliah Heller, Kevin Konty, Sharon Balter, Tian Zheng, and James H. Stark. 2018. "A Bayesian Evidence Synthesis Approach to Estimate Disease Prevalence in Hard-to-Reach Populations: Hepatitis c in New York City." *Epidemics* 23: 96–109. https://doi.org/https://doi.org/10.1016/j.epidem.2018.01.002.

Thein, Hla-Hla, Qilong Yi, Gregory J. Dore, and Murray D. Krahn. 2008. "Estimation of Stage-Specific Fibrosis Progression Rates in Chronic Hepatitis c Virus Infection: A Meta-Analysis and Meta-Regression." *Hepatology* 48 (2): 418–31. https://doi.org/https://doi.org/10.1002/hep.22375.

Trickey, Adam, Hannah Fraser, Aaron G Lim, Amy Peacock, Samantha Colledge, Josephine G Walker, Janni Leung, et al. 2019. "The Contribution of Injection Drug Use to Hepatitis c Virus Transmission Globally, Regionally, and at Country Level: A Modelling Study." *The Lancet Gastroenterology & Hepatology* 4 (6): 435–44. https://doi.org/https://doi.org/10.1016/S2468-1253(19)30085-8.

Veen, Maaike G. van, Anne M. Presanis, Stefano Conti, Maria Xiridou, Annemarie R. Stengaard, Martin C. Donoghoe, Ard I. van Sighem, Marianne A. van der Sande, and Daniela De Angelis. 2011. "National Estimate of HIV Prevalence in the Netherlands: Comparison and Applicability of Different Estimation Tools." *AIDS* 25 (2). https://journals.lww.com/aidsonline/Fulltext/2011/01140/National_estimate_of_HIV_prevalence_in_the.14.aspx.

Veseliny, Eduard, Martin Janicko, Sylvia Drazilova, Leonard Siegfried, Lydia Pastvova, Ivan Schreter, Pavol Kristian, et al. 2014. "High Hepatitis b and Low Hepatitis c Prevalence in Roma Population in Eastern Slovakia." *Central European Journal of Public Health* 22 (88): S51–56. https://doi.org/10.21101/cejph.a3902.

# Estimating the prevalence of chronic hepatitis C infection (CHC) in Slovenia using Bayesian multiparameter evidence synthesis

07/07/2022

## Table of Contents

Introduction .......... 2
Methods .......... 2
Prevalence of ex-PWID .......... 3
Prevalence of recent PWID .......... 3
Prevalence of CHC among recent PWID .......... 4
Prevalence of CHC among ex-PWID .......... 4
Prevalence of CHC among non-PWID .......... 5
Results .......... 6
Tables and Figures .......... 8
Appendix .......... 12
Fit of the multi-state Markov model .......... 12
Stan code for Bayesian multiparameter evidence synthesis .......... 13
Multi-state Markov model .......... 19
References .......... 27

# INTRODUCTION

Patients with chronic hepatitis C (HCV) infection often remain asymptomatic for decades and even for life, but a proportion of them develop active chronic hepatitis, which is a progressive disease (Thein et al. 2008). Because of the largely asymptomatic nature of HCV infection, notification data reflect national screening and testing practices and do not give accurate insights into the prevalence of HCV infection. Although there are studies that have estimated the prevalence of HCV antibodies (anti-HCV) in specific groups such as the general population, first-time blood donors, or people who inject drugs (PWID) at drug treatment centers, those alone cannot be combined to produce national estimates unless some additional information regarding the composition of each HCV risk group and its prevalence in the population are known (Sweeting et al. 2008; McDonald et al. 2014; Tan et al. 2018).

# METHODS

Bayesian multiparameter evidence synthesis (MPES) has been a popular approach to formally estimate anti-HCV or HIV prevalence (Sweeting et al. 2008; McDonald et al. 2014; Tan et al. 2018; Presanis et al. 2010; Veen et al. 2011). In this project, we extend this method to estimating the CHC prevalence, with the population (15-79 years) split into three main non-overlapping risk groups, i.e., recent PWID (those who have injected in the last year), ex-PWID, and non-PWID. A unified model is assumed including parameters associated with the CHC prevalence of recent, ex-PWID, and non-PWID (denoted by $\pi_{rec}$, $\pi_{ex}$, and $\pi_{non}$, respectively) and the prevalence of these risk groups in the population (denoted by $\rho_{rec}$, $\rho_{ex}$, and $\rho_{non}$, respectively; $\rho_{rec} + \rho_{ex} + \rho_{non} = 1$). To estimate the CHC prevalence in the whole population, $\pi$, we used the formula:

$$\pi = \pi_{rec}\rho_{rec} + \pi_{ex}\rho_{ex} + \pi_{non}\rho_{non} \quad (1)$$

Bayesian synthesis considers all available data, allowing for an evidence-based estimation of CHC prevalence in the population, with inherent uncertainty properly accounted for. Our aim is to estimate the CHC prevalence for the total (15-79) population of Slovenia in 2019 using sources of information over the last decade (2010-2019).

## Prevalence of ex-PWID

Data on the prevalence of ex-PWID are sparse and generally often unreliable. To overcome this limitation, we apply the method proposed by McDonald et al. (2014), which requires simulating a multi-state Markov model representing the non-PWID, recent PWID, and ex-PWID. In the multi-state Markov model, the simulation starts in 1950 and examines the population aged 15-79 years old. A certain number of 15-year-old individuals enter the simulation process each year. These numbers of 15-year-old individuals are obtained from the United Nations Statistic Division UNSD, being different for each country. The ageing of the population is also taken into account. Country-specific overall mortality data are retrieved from the Life Tables of the World Health Organization WHO. The duration of injecting career is also country-specific and obtained from the paper of Hines et al. (2020). However, since no data for Slovenia are reported in Hines et al. (2020), we used the average duration of injecting career in Western Europe (Hines et al. 2020).

To estimate the number of ex-PWID, the model can be calibrated on the number of recent PWID provided in the EMCDDA barometer or the EMCDDA statistical bulletin (the code of the Markov model can be found in the Appendix). If there are no available data in the barometer, estimates from the systematic review of Grebely et al. (2019) could be used. Currently, the multi-state Markov model was calibrated on the estimated number of recent PWID reported in the Report on the Drug Situation 2021 of the Republic of Slovenia ($n \approx 3015$), i.e. on the 70.9% of 4252 high-risk opioid users (HROU) (Kvaternik and K 2021; Kvaternik and Z 2021).

After applying the model for Slovenia, we compute the number (and the corresponding Confidence Interval - CI) of ex-PWID in 2019. The number of ex-PWID is then divided by the population size (15-79 years) in the same year, and a Normal distribution, constrained such that $\rho_{ex} \in (0,1)$, is assumed for $\rho_{ex}$. The standard deviation of this distribution is specified to approximately correspond to the respective CI, i.e. $\left(UB_{\rho_{ex}} - LB_{\rho_{ex}}\right)/(2 \times 1.96)$, where $UB_{\rho_{ex}}$ and $LB_{\rho_{ex}}$ denote the upper and lower limit of the CI.

## Prevalence of recent PWID

The number of recent PWID in Slovenia in 2019 is also projected by the multi-state Markov model described in the previous subsection. To estimate $\rho_{rec}$, the number of recent PWID is

divided by the population size (15-79 years). Similar to the previous subsection, a Normal distribution, constrained such that $\rho_{rec} \in (0,1)$, is assumed for $\rho_{rec}$, with the standard deviation specified to correspond to the CI obtained from the multi-state Markov model.

## Prevalence of CHC among recent PWID

The prevalence of CHC among recent PWID was informed by CHC prevalence data from the paper of Grebely et al. (2019) in 2011-2014. However, some people may have been treated with direct-acting antivirals (DAAs). Based on the information kindly provided by Prof. Maticic, the number of cured PWIDs in 2015-2017 is equal to 146 (M. Maticic et al. 2019; Mojca Maticic et al. 2019), whereas the total number of cured individuals in 2018-19 in Slovenia is equal to 572. Moreover, the proportion of PWID among treated individuals in 2020-2021 is equal to 222/290, which was extrapolated to inform the unknown proportion of PWID among the total number of cured individuals in 2018-19 (denoted by $\rho_{PWID|cured}$). However, the proportions of recent and ex-PWID among PWID cured with DAAs are not currently available in Slovenia. In this report, we make the assumption that these are proportional to the corresponding proportions among CHC-positive individuals, i.e. Pr(Recent PWID|CHC) and Pr(Ex-PWID|CHC), as estimated by our model when the DAA uptake is ignored (Table 2). Thus, the CHC prevalence among recent PWID, adjusted for DAAs, can be estimated by

$$\pi_{rec} = \frac{N_{15,79}\rho_{rec}\tilde{\pi}_{rec} - \left(146 + 572\rho_{PWID|cured}\right)\frac{\text{Pr(Recent PWID|CHC)}}{\text{Pr(Recent PWID|CHC)} + \text{Pr(Ex-PWID|CHC)}}}{N_{15,79}\rho_{rec}}, \quad (2)$$

where $\tilde{\pi}_{rec}$ denotes the CHC estimate derived solely by CHC data from the paper of Grebely et al. (2019) in 2011-2014.

## Prevalence of CHC among ex-PWID

Information on CHC prevalence among ex-PWID (i.e., $\pi_{ex}$) is difficult to obtain directly. To overcome this, national anti-HCV prevalence data among ever PWID in 2019, available in the EMCDDA statistical bulletin, were used. However, as these data refer to the anti-HCV prevalence, they should be adjusted similarly to the procedure described in the previous subsection, i.e.

$$\pi_{ever} = \frac{N_{15,79}\rho_{ever}\pi(\text{anti-HCV})_{ever}(1-\rho_{clear}) - \left(146 + 572\rho_{PWID|cured}\right)}{N_{15,79}\rho_{ever}}, \quad (3)$$

where $\pi(\text{anti-HCV})_{ever}$ denotes the anti-HCV prevalence among ever PWID, $\rho_{ever} = \rho_{rec} + \rho_{ex}$, and $\rho_{clear}$ denotes the spontaneous viral clearance [assumed to be equal to 0.26 (95% CI 0.22–0.29); (Micallef, Kaldor, and Dore 2006)].

Then, since an estimate of $\pi_{ever}$ is available, $\pi_{ex}$ can be indirectly estimated since $\pi_{ever}$ is equal to a weighted average of $\pi_{rec}$ and $\pi_{ex}$, as shown by the following formula

$$\pi_{ever} = \frac{\rho_{rec}}{\rho_{ever}}\pi_{rec} + \frac{\rho_{ex}}{\rho_{ever}}\pi_{ex}, \quad (4)$$

or, equivalently,

$$\pi_{ex} = \left(\pi_{ever} - \frac{\rho_{rec}}{\rho_{ever}}\pi_{rec}\right) \times \frac{\rho_{ever}}{\rho_{ex}}. \quad (5)$$

## Prevalence of CHC among non-PWID

To estimate the CHC prevalence among non-PWID in a country (after 2010), the ECDC database (European Centre for Disease Prevention and Control (ECDC) 2021) was used. The ECDC group has carefully and critically collected information from relevant studies across the EU/EEA region in cooperation with the focal points of each country. On the basis of the representativeness of each study and other factors (European Centre for Disease Prevention and Control (ECDC) 2016), a risk of bias score was assigned to each study, with higher values indicating higher-quality studies. When higher-quality studies are available in a country (≥ 4), they are used to estimate the overall CHC prevalence in that country among non-PWID. If there are no higher-quality studies, general population estimates with a lower quality (<4) are pooled. If no general population prevalence estimates are available, data on the prevalence of HCV in pregnant women could be used as a prevalence measure. Finally, if data on pregnant women are lacking too, first-time blood donor studies could be an option. However, if the national focal point agrees or recommends, estimates from a neighboring country could be used instead, or we could use any other relevant information provided/suggested by the national focal point.

We directly use CHC prevalence data from studies in the general population, if available. In Slovenia, there was 1 study on pregnant women, which included CHC data (unpublished data, personal communication with Prof. Irena Klavs). Since recent, i.e. in 2019, CHC data are available for non-PWID, we did not perform a treatment adjustment in the general population.

# RESULTS

The above-mentioned approach was used to estimate the prevalence of CHC in 2019 (considering ages between 15-79 years: 1,656,169).

The aggregated data used by our approach are briefly presented in Table 1. The multi-state Markov model was calibrated on the estimated number of recent PWID reported in the Report on the Drug Situation 2021 of the Republic of Slovenia ($n \approx 3015$), i.e. on the 70.9% of 4252 high-risk opioid users (HROU) (Kvaternik and K 2021; Kvaternik and Z 2021). In Slovenia, there was 1 study on pregnant women, which included CHC data (unpublished data, personal communication with Prof. Irena Klavs).

To estimate the CHC prevalence in the general population (primarily non-PWID), we used a fixed-effect meta-analytic approach, with the corresponding results, when information on DAAs is ignored, presented in Table 2. However, based on data provided by the national focal points, approximately 583 individuals were cured with DAAs up to 2019 in Slovenia, with the proportion of recent PWID and ex-PWID among them assumed to be equal to $\frac{\Pr(\text{Recent PWID}|\text{CHC})}{\Pr(\text{Recent PWID}|\text{CHC})+\Pr(\text{Ex-PWID}|\text{CHC})} \approx$ 48.43% (95% CI:26.37-90.84) and $\frac{\Pr(\text{Ex-PWID}|\text{CHC})}{\Pr(\text{Recent PWID}|\text{CHC})+\Pr(\text{Ex-PWID}|\text{CHC})} \approx$ 51.57% (95% CI:9.16-73.63), respectively, as estimated by our model when information on DAAs is ignored (Table 2).

The corresponding results accounting for the DAA uptake are presented in Table 3. The prevalence of recent and ex-PWID was low in Slovenia (about 0.18% and 0.51%, respectively) corresponding to 3,030 (95% CI: 2,640-3,320) recent PWID and 8,470 (95% CI: 7,890-9,110) ex-PWID in the population. However, the CHC prevalence in these groups was relatively high (Table 3) being 13.45% and 5.21%, respectively. This translates to 407 (95% CI: 166-573) and 441 (95% CI: 20-1,419) recent and ex-PWID aged 15-79 living with CHC infection in Slovenia in 2019. The CHC prevalence in the general population was 0.01% (95% CI: 0%-0.05%), much

lower than that of the high-risk groups. Taking all pieces of information into account, the overall CHC prevalence in Slovenia in 2019 was equal to 0.07% (95% CI: 0.02%-0.14%), which corresponds to 1,078 (95% CI: 317-2,319) individuals aged 15-79 years with CHC infection.

# TABLES AND FIGURES

*Table 1. Data (real) contributing to the estimation of CHC prevalence in Slovenia in 2019.*

| Parameter | Estimate (95%CI) | Number (95%CI) | Numerator | Denominator | Notes | Year of study |
|---|---|---|---|---|---|---|
| $\rho_{rec}$ | | 3,030 (2,640-3,320) | | | Method based on McDonald et al. | 2019 |
| $\rho_{ex}$ | | 8,470 (7,890-9,110) | | | Method based on McDonald et al. | 2019 |
| $\pi_{rec}$ | 22.9% (19.6%-26.2%) | | | | Grebely et al. | 2011-2014 |
| $\pi(\text{anti-HCV})_{ever}$ | | | 6 | 39 | EMCDDA database | 2019 |
| $\pi_{non}$ | | | 0 | 7,193 | Unpublished data, personal communication with Prof. Irena Klavs; Risk of bias=NA | 2019 |

*Notes*: Although it looks counter-intuitive, **a higher risk of bias score denotes a higher-quality study** (range from 0 to 6).

*Table 2. Results from the method assuming no heterogeneity in the estimation of the CHC prevalence in the general population from different studies (i.e., similar to a fixed-effect meta-analysis).*

| Parameter | Posterior median | Posterior sd | LB (2.5%) | UB (97.5%) |
|---|---|---|---|---|
| $\rho_{rec}$ (%) | 0.18 | 0.01 | 0.16 | 0.2 |
| $\rho_{ex}$ (%) | 0.51 | 0.02 | 0.47 | 0.55 |
| $\pi_{rec}$ (%) | 22.88 | 1.68 | 19.63 | 26.22 |
| $\pi_{ex}$ (%) | 8.72 | 5.63 | 0.85 | 22.21 |
| $\pi_{non}$ (%) | 0.01 | 0.01 | 0 | 0.05 |
| $\pi$ (%) | 0.1 | 0.03 | 0.05 | 0.18 |
| Number with CHC | 1,661 | 527 | 894 | 2,910 |
| Pr(Recent PWID\|CHC) (%) | 41.66 | 14.17 | 23.11 | 78.48 |
| Pr(Ex-PWID\|CHC) (%) | 44.95 | 16.58 | 6.95 | 70.16 |
| Pr(Non-PWID\|CHC) (%) | 10.02 | 10.96 | 0.39 | 40.45 |

*Notes*: Information on DAAs is not taken into account.

*Abbreviations*: $\rho_{rec}$, prevalence of recent PWID (proportion of the population that is recent PWID); $\rho_{ex}$, prevalence of ex-PWID (proportion of the population that is ex-PWID); $\pi_{rec}$, CHC prevalence among recent PWID; $\pi_{ex}$, CHC prevalence among ex-PWID; $\pi_{non}$, CHC prevalence among non-PWID; $\pi$ overall CHC prevalence in Slovenia; LB, Lower Boundary; UP, Upper Boundary

*Table 3. Results from the method assuming no heterogeneity in the estimation of the CHC prevalence in the general population from different studies (i.e., similar to a fixed-effect meta-analysis).*

| Parameter | Posterior median | Posterior sd | LB (2.5%) | UB (97.5%) |
|---|---|---|---|---|
| $\rho_{rec}$ (%) | 0.18 | 0.01 | 0.16 | 0.2 |
| $\rho_{ex}$ (%) | 0.51 | 0.02 | 0.47 | 0.55 |
| $\pi_{rec}$ (%) | 13.45 | 3.32 | 5.55 | 18.44 |
| $\pi_{ex}$ (%) | 5.21 | 4.5 | 0.24 | 16.78 |
| $\pi_{non}$ (%) | 0.01 | 0.01 | 0 | 0.05 |
| $\pi$ (%) | 0.07 | 0.03 | 0.02 | 0.14 |
| Number with CHC | 1,078 | 521 | 317 | 2,319 |
| Pr(Recent PWID\|CHC) (%) | 37.25 | 12.32 | 20.42 | 68.37 |
| Pr(Ex-PWID\|CHC) (%) | 41.98 | 17.38 | 4.7 | 69.2 |
| Pr(Non-PWID\|CHC) (%) | 16.06 | 17.3 | 0.65 | 64.46 |

*Notes*: The number of individuals treated with DAAs is taken into account.

*Abbreviations*: $\rho_{rec}$, prevalence of recent PWID (proportion of the population that is recent PWID); $\rho_{ex}$, prevalence of ex-PWID (proportion of the population that is ex-PWID); $\pi_{rec}$, CHC prevalence among recent PWID; $\pi_{ex}$, CHC prevalence among ex-PWID; $\pi_{non}$, CHC prevalence among non-PWID; $\pi$ overall CHC prevalence in Slovenia; LB, Lower Boundary; UP, Upper Boundary

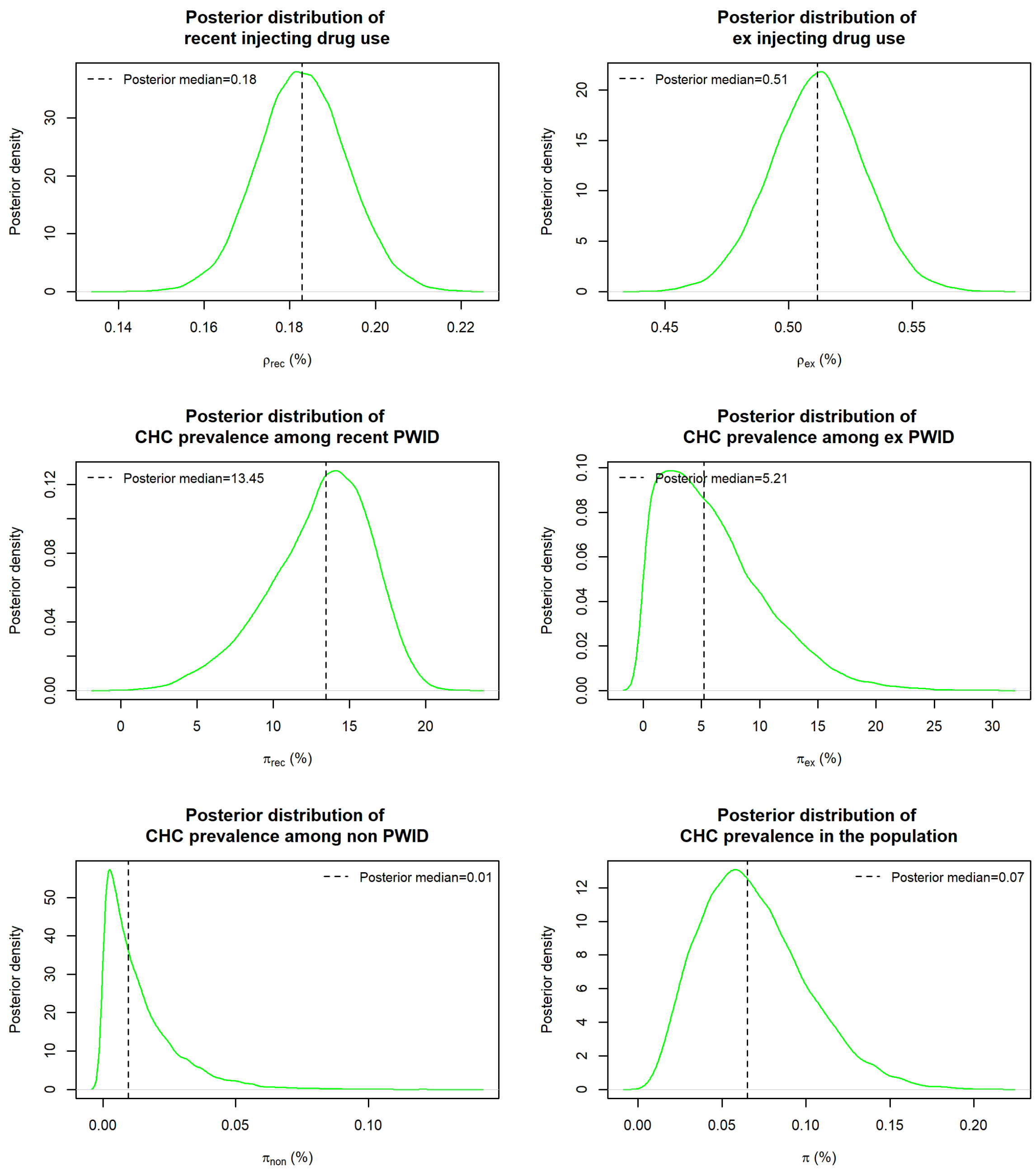


*Figure 1. Posterior distributions of the key model parameters using the approach that assumes no heterogeneity in the estimation of the CHC prevalence in the general population (primarily non-PWID) from different studies (i.e., similar to a fixed-effect meta-analysis), with the number of individuals treated with DAA taken into account.*

# APPENDIX

## Fit of the multi-state Markov model

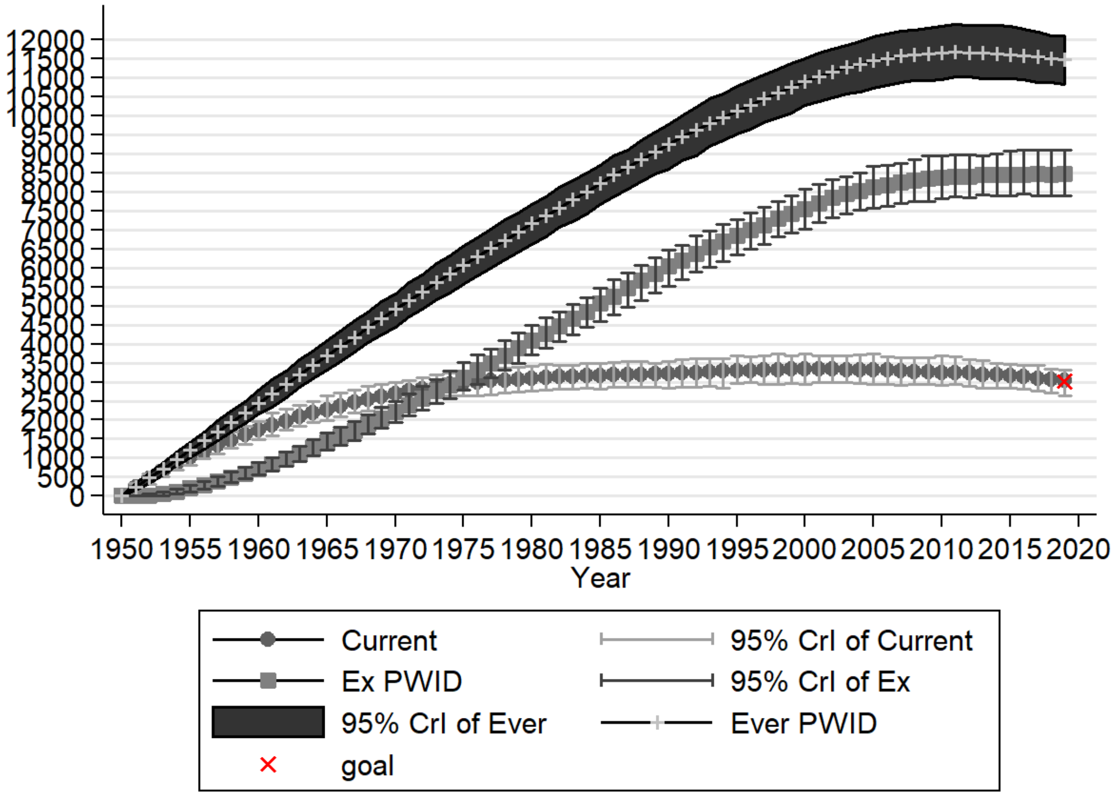


*Figure 2: Model predictions for the numbers of current and ex-PWID (people who inject drugs). The solid black line and shaded grey error bars show the median and 95% credible intervals (95% CrI) for the model projections. For comparison, asterisks indicate the observed country's data.*

## Stan code for Bayesian multiparameter evidence synthesis

```
data {
  int<lower=0> N1579; // Population of 15-79 in `Country`
  int<lower=1> Knon; // Number of studies in the ECDC data for `Country`
  int<lower=1> Kever; // Number of studies in the EMCDDA data for ever users in `Country`
  //int<lower=1> Kcur; // Number of studies in the EMCDDA barometer (for HCV prevalence of PWID) in `Country`
  int<lower=1> Ncured_pwid1517; // Number of cured PWID in 2015-2017
  int<lower=1> Ncured_1819; // Number of cured individuals in 2018-2019
  int<lower=1> Nrho_pwid_daa; // Number of treated individuals in 2020-2021
  int<lower=1> Yrho_pwid_daa; // Number of treated PWID in 2020-2021

  real p_cur_mean; // Prior mean for the prevalence of current use in `Country`
  real<lower=0> p_cur_sd; // Prior sd for the prevalence of current use in `Country`

  real p_ex_mean; // Prior mean for the prevalence of ex-use in `Country`
  real<lower=0> p_ex_sd; // Prior sd for the prevalence of ex-use in `Country`

  //int<lower=0> Nst_hcv_cur[Kcur]; // Number of individuals in the study estimating anti-HCV among recent PWID
  //int<lower=0> Yst_hcv_cur[Kcur]; // Number of HCV+ in the study estimating anti-HCV among recent PWID
  real p_CHC_cur_mean; // Prior mean for the CHC prevalence among recent PWID in `Country`
  real<lower=0> p_CHC_cur_sd; // Prior sd for the CHC prevalence among recent PWID in `Country`
```

```
  int<lower=0> Nst_hcv_ever[Kever]; // Number of individuals in the study estimating anti-HCV among ever IDU
  int<lower=0> Yst_hcv_ever[Kever]; // Number of HCV+ in the study estimating anti-HCV among ever PWID

  int<lower=0> Nst_CHC_non[Knon]; // Number of individuals in the study estimating CHC among non PWID
  int<lower=0> Yst_CHC_non[Knon]; // Number of HCV+ in the study estimating CHC among non PWID

  vector<lower=0>[3] alpha; // parameter of the Diriclet prior

  real HCVclear_mean; // Prior mean for the HCV clearance probability
  real<lower=0> HCVclear_sd; // Prior sd for the HCV clearance probability
}

// Block defining the original parameters
parameters {
  // The parameters to be sampled
  simplex[3] rho; // Prevalence of the three risk groups
  real<lower=0,upper=1> CHCpi_cur; // P(CHC+|Cur)
  real<lower=0,upper=1> CHCpi_ex; // P(CHC+|Ex)
  real<lower=0,upper=1> CHCpi_non; // P(CHC+|Non)
  real<lower=0,upper=1> rho_PWID_DAA; // Pr(PWID|DAA)

  real<lower=0,upper=1-CHCpi_cur> HCVclear; // Probability of HCV clearance; upper bound = 1-prevalence of chronic HCV
}


transformed parameters {
```

```
  // Change scales
  real<lower=0,upper=1> rho_ever;
  real<lower=0,upper=1> CHCpi_ever;
  real<lower=0,upper=1> pi_ever;

  rho_ever = rho[1] + rho[2];
  CHCpi_ever = CHCpi_cur*rho[1]/rho_ever + CHCpi_ex*rho[2]/rho_ever;
  pi_ever = CHCpi_ever/(1-HCVclear);
}


// Binomial regression model
model {
  // Priors
  rho ~ dirichlet(alpha);

  //////////////////////////////
  // Likelihood contributions //
  //////////////////////////////

  // Probability of HCV clearance
  HCVclear ~ normal(HCVclear_mean,HCVclear_sd);

  // Prevalence of current use
  rho[1] ~ normal(p_cur_mean,p_cur_sd);

  // Prevalence of ex-use
  rho[2] ~ normal(p_ex_mean,p_ex_sd);

  // Prevalence of chronic HCV among current users
  CHCpi_cur ~ normal(p_CHC_cur_mean,p_CHC_cur_sd);
```

```
  // Prevalence of HCV among ever users
  Yst_hcv_ever ~ binomial(Nst_hcv_ever,pi_ever);

  // HCV+ among non
  Yst_CHC_non ~ binomial(Nst_CHC_non,CHCpi_non);

  // PWID among all treated
  Yrho_pwid_daa ~ binomial(Nrho_pwid_daa,rho_PWID_DAA);
}



generated quantities {
  // Functions of parameters
  real<lower=0,upper=100> overalCHC;
  real logit_CHCpi_cur;
  real logit_CHCpi_ex;
  real logit_CHCpi_non;
  real logit_rho_cur;
  real logit_rho_ex;
  real logit_rho_non;
  real<lower=0,upper=1> pEverGivenCHC;
  real<lower=0,upper=1> pCurGivenCHC;
  real<lower=0,upper=1> pExGivenCHC;
  real<lower=0,upper=1> pNonGivenCHC;
  real logit_HCVclear;
  real<lower=0> NumberCHC;

  real<lower=0,upper=1> CHCDAApi_non; // P(CHC+|Non) adjusted for DAA
  real CHCDAApi_cur; // P(CHC+|Cur) adjusted for DAA
  real CHCDAApi_ex; // P(CHC+|Ex) adjusted for DAA
  real CHCDAApi_ever; // P(CHC+|Ever) adjusted for DAA
```

```
  real<lower=0,upper=100> overalCHC_DAA;
  real pEverGivenCHC_DAA;
  real pCurGivenCHC_DAA;
  real pExGivenCHC_DAA;
  real pNonGivenCHC_DAA;
  real<lower=0> NumberCHC_DAA;
  real<lower=0> Ncured_pwid;


  // Overall HCV prevalence
  overalCHC = 100*(rho[1]*CHCpi_cur + rho[2]*CHCpi_ex +  rho[3]*CHCpi_non
);
  pEverGivenCHC = CHCpi_ever*rho_ever/(overalCHC/100);
  pCurGivenCHC = CHCpi_cur*rho[1]/(overalCHC/100);
  pExGivenCHC = CHCpi_ex*rho[2]/(overalCHC/100);
  pNonGivenCHC = CHCpi_non*(1-rho_ever)/(overalCHC/100);
  NumberCHC = round(overalCHC*N1579/100);

  Ncured_pwid = Ncured_pwid1517 + Ncured_1819*rho_PWID_DAA;
  CHCDAApi_non = ( N1579*rho[3]*CHCpi_non - 0)/(N1579*rho[3]);
  CHCDAApi_cur = ( N1579*rho[1]*CHCpi_cur - Ncured_pwid*pCurGivenCHC/(pCu
rGivenCHC+pExGivenCHC))/(N1579*rho[1]);
  CHCDAApi_ex = ( N1579*rho[2]*CHCpi_ex - Ncured_pwid*pExGivenCHC/(pCurGi
venCHC+pExGivenCHC))/(N1579*rho[2]);
  CHCDAApi_ever = CHCDAApi_cur*rho[1]/rho_ever + CHCDAApi_ex*rho[2]/rho_e
ver;

  overalCHC_DAA = 100*(rho[1]*CHCDAApi_cur + rho[2]*CHCDAApi_ex + rho[3]*
CHCDAApi_non);
  pEverGivenCHC_DAA = CHCDAApi_ever*rho_ever/(overalCHC_DAA/100);
  pCurGivenCHC_DAA = CHCDAApi_cur*rho[1]/(overalCHC_DAA/100);
  pExGivenCHC_DAA = CHCDAApi_ex*rho[2]/(overalCHC_DAA/100);
```

```
  pNonGivenCHC_DAA = CHCDAApi_non*(1-rho_ever)/(overalCHC_DAA/100);
  NumberCHC_DAA = round(overalCHC_DAA*N1579/100);

  logit_rho_cur = logit(rho[1]);
  logit_rho_ex = logit(rho[2]);
  logit_rho_non = logit(rho[3]);
  logit_CHCpi_cur = logit(CHCpi_cur);
  logit_CHCpi_ex = logit(CHCpi_ex);
  logit_CHCpi_non = logit(CHCpi_non);
  logit_HCVclear = logit(HCVclear);
}
```

**Multi-state Markov model**

```
#include <stdio.h>
#include <stdlib.h>
#include <string.h>
#include <math.h>
#include <time.h>

#define loops 100
int populationPerYearAndAge[2021][66];
double deathRatePerAge[66];
int totalPopulationPerYear[2021];

FILE *F_DeathRate=fopen("`Country`-deathRates.txt","r");
FILE *F_Population=fopen("`Country`-population.txt","r");
FILE *out;

double pa_start = 0.00012;                    // Setting Variable
double pa_step  = 0.000002;                   // Setting Variable
double pa_stop  = 0.000123;                   // Setting Variable

double pa;
double pg = 1.0/11.7;    // Assumed average active injecting career of  11
.7 years
double pk = 0.004*12.0;     // Relapse rate of 0.004/month

struct people{
    int age;
    int state;
};

char filename[100];
struct people person[50000000];
```

```
long int totalPersons=0;

void setPopulationAge()
{
    for( int j=0; j<71; j++){
        for( int i=0; i<51; i++){
            fscanf(F_Population,"%d",&populationPerYearAndAge[1950+j][15+
i]);
        }
    }
}

void setDeathRate()
{
    for( int i=0; i<51; i++){
        fscanf(F_DeathRate,"%lf",&deathRatePerAge[15+i*5]);
        for( int j=0; j<5; j++){
            deathRatePerAge[15+i*5+j] = deathRatePerAge[15+i*5];
        }
    }
}

void getTotalPopulationPerAge()
{
    for( int j=0; j<71; j++){
        for( int i=0; i<51; i++){
            totalPopulationPerYear[1950+j] += populationPerYearAndAge[195
0+j][15+i];
        }
    }
}
```

```
void printTotalPopulationPerAge()
{
    printf("---- - ----------\n");
    printf("Year - Population\n");
    printf("---- - ----------\n");
    for( int i=1950; i<2021; i++){
        printf("%d - %d\n",i,totalPopulationPerYear[i]);
    }
}

void printDeathRatePerAge()
{
    printf("--- - -----\n");
    printf("Age - Rate\n");
    printf("--- - -----\n");
    for( int i=15; i<65; i++){
        printf("%d - %lf\n",i,deathRatePerAge[i]);
    }
}

void printTotalPersonPerState( int year)
{
    int count[4]= {0,0,0,0};
    for( int i=0; i<totalPersons; i++){
        count[person[i].state]++;
    }
    //printf("%d\t%ld\t%ld\t%ld\t%ld\n",year,count[0],count[1],count[2],c
ount[3]);
    if(year==2014)
        printf("%d\t%ld\t%ld\t%ld\t%ld\t%lf\n",year,count[0],count[1],cou
nt[2],count[3],pa);
    if( year>2009){
```

```
        fprintf(out, "%d\t%ld\t%ld\t%ld\t%ld\n",year,count[0],count[1],co
unt[2],count[3]);
    }
}

void initializePopulation()
{
    int cnt=0;
    for( int j=15; j<65; j++){
        for( int i=0; i<populationPerYearAndAge[1950][j]*100; i++){
            person[cnt].age=j;
            person[cnt].state=0;
            cnt++;
        }
    }
    totalPersons = cnt;
}

bool checkRate(double rate)
{
    if (rate <0.001){
        double k = rand()/(1.00+RAND_MAX);
        if(k<0.001){
            rate = rate*1000;
        }
        else{
            return false;
        }
    }
    double k = rand()/(1.00+RAND_MAX);
    if ( k < rate )
        return true;
```

```
    else
        return false;
}

void changeStatusAndAge()
{
    for( int i=0; i<totalPersons; i++){
        // change state
        double k = rand()/(1.00+RAND_MAX);
        if( person[i].state==0){
            if ( checkRate(pa) &&  person[i].age<40){
                person[i].state=1;
            }
        }
        else if( person[i].state==1){
            if ( k < pg ){
                person[i].state=2;
            }
        }
        else if( person[i].state==2){
            if ( k < pk ){
                person[i].state=1;
            }
        }
        // After their 49th year, all active PWID are assumed to cease in
jecting
        if(person[i].age>49 && person[i].state==1){
            person[i].state=2;
        }
        // After their 64th year, remove
        if(person[i].age>64){
            person[i].state=3;
```

```
        }
        // death rate depending on age and state
        k = rand()/(1.00+RAND_MAX);
        if( person[i].state==0){
            if ( k < deathRatePerAge[person[i].age] ){
                person[i].state=3;
            }
        }
        else if( person[i].state==1){
            if ( k < deathRatePerAge[person[i].age] * 13.0 ){
                person[i].state=3;
            }
        }
        else if( person[i].state==2){
            if ( k < deathRatePerAge[person[i].age] ){
                person[i].state=3;
            }
        }
        // increase age one year
        person[i].age++;
    }
}

void addNewPersons(int year)
{
    // add new person [15 years old and non PWID]
    int cnt=totalPersons;
    for( int i=0; i<populationPerYearAndAge[year+1][15]*100; i++){
// 100 instead of 1000 for the population to reduce execution time
        person[cnt].age=15;
        person[cnt].state=0;
        cnt++;
```

```
    }
    // increase the total number of population
    totalPersons = cnt;
}
int main()
{
    srand(time(NULL));

    setPopulationAge();
    setDeathRate();

    getTotalPopulationPerAge();
    pa = pa_start;
    while ( pa < pa_stop){

        snprintf(filename, 100, "result_%lf.txt",pa);
        out=fopen(filename,"w");

        for( int iter=0; iter<loops ;iter++){
            initializePopulation();
            for( int year=1950; year<2020; year++){
                printTotalPersonPerState(year);
                changeStatusAndAge();
                addNewPersons(year);
            }
        }

        fclose(out);
        pa = pa + pa_step;
    }
```

```
}
```

# REFERENCES

European Centre for Disease Prevention and Control (ECDC). 2016. "Systematic Review on Hepatitis b and c Prevalence in the EU/EEA." https://www.ecdc.europa.eu/sites/default/files/media/en/publications/Publications/systematic-review-hepatitis-B-C-prevalence.pdf.

———. 2021. "Hepatitis c Prevalence Database." https://www.ecdc.europa.eu/en/all-topics-z/hepatitis-c/tools/hepatitis-c-prevalence-database.

Grebely, Jason, Sarah Larney, Amy Peacock, Samantha Colledge, Janni Leung, Matthew Hickman, Peter Vickerman, et al. 2019. "Global, Regional, and Country-Level Estimates of Hepatitis c Infection Among People Who Have Recently Injected Drugs." *Addiction* 114 (1): 150–66. https://doi.org/https://doi.org/10.1111/add.14393.

Hines, Lindsey A., Adam Trickey, Janni Leung, Sarah Larney, Amy Peacock, Louisa Degenhardt, Samantha Colledge, et al. 2020. "Associations Between National Development Indicators and the Age Profile of People Who Inject Drugs: Results from a Global Systematic Review and Meta-Analysis." *The Lancet Global Health* 8 (1): e76–91. https://doi.org/10.1016/S2214-109X(19)30462-0.

Kvaternik, I, and Rostohar K. 2021. "Estimate of the Number of High Risk Opioid Users. In: Jandl m (Editor) Report on the Drug Situation 2021 of the Republic of Slovenia." https://www.nijz.si/sl/publikacije.

Kvaternik, I, and Zerjal Z. 2021. "Harm Reduction Services: Availability,access and Trends. In: Jandl m (Editor) Report on the Drug Situation 2021 of the Republic of Slovenia." https://www.nijz.si/sl/publikacije.

Maticic, Mojca, Zala Pirnat, Jelka Meglic Volkar, Sergeja Gregorcic, Mojca Rajter, Janja Prah, Tadeja Kotar, et al. 2019. "THU-160-Outcomes of the National Strategy on Hepatitis c Treatment with Direct Acting Antivirals in a Real-Life Setting: Results from a National Survey in Slovenia." *Journal of Hepatology* 70 (1): e232. https://doi.org/10.1016/S0618-8278(19)30435-9.

Maticic, M., Z. Pirnat, J. Zorman, J. Volkar, S. Gregorcic, M. Rajter, J. Prah, et al., eds. 2019. *The Comparison of Hepatitis c Treatment Outcomes with Direct Acting Antivirals Between People Who Inject Drugs (PWID) and Non-PWID: Results from a National Survey in Slovenia*. ECCMID.

McDonald, Scott A., Rosmawati Mohamed, Maznah Dahlui, Herlianna Naning, and Adeeba Kamarulzaman. 2014. "Bridging the Data Gaps in the Epidemiology of Hepatitis c Virus Infection in Malaysia Using Multi-Parameter Evidence Synthesis." *BMC Infectious Diseases* 14 (1): 564. https://doi.org/10.1186/s12879-014-0564-6.

Micallef, J. M., J. M. Kaldor, and G. J. Dore. 2006. "Spontaneous Viral Clearance Following Acute Hepatitis c Infection: A Systematic Review of Longitudinal Studies." *Journal of Viral Hepatitis* 13 (1): 34–41. https://doi.org/https://doi.org/10.1111/j.1365-2893.2005.00651.x.

Presanis, Anne M., O. Noel Gill, Timothy R. Chadborn, Caterina Hill, Vivian Hope, Louise Logan, Brian D. Rice, Valerie C. Delpech, A. E. Ades, and Daniela De Angelis. 2010. "Insights into the Rise in HIV Infections, 2001 to 2008: A Bayesian Synthesis of Prevalence Evidence." *AIDS* 24 (18). https://journals.lww.com/aidsonline/Fulltext/2010/11270/Insights_into_the_rise_in_HIV_infections,_2001_to.12.aspx.

Sweeting, M. J., D. De Angelis, M. Hickman, and A. E. Ades. 2008. "Estimating hepatitis C prevalence in England and Wales by synthesizing evidence from multiple data sources. Assessing data conflict and model fit." *Biostatistics* 9 (4): 715–34. https://doi.org/10.1093/biostatistics/kxn004.

Tan, Sarah, Susanna Makela, Daliah Heller, Kevin Konty, Sharon Balter, Tian Zheng, and James H. Stark. 2018. "A Bayesian Evidence Synthesis Approach to Estimate Disease Prevalence in Hard-to-Reach Populations: Hepatitis c in New York City." *Epidemics* 23: 96–109. https://doi.org/https://doi.org/10.1016/j.epidem.2018.01.002.

Thein, Hla-Hla, Qilong Yi, Gregory J. Dore, and Murray D. Krahn. 2008. "Estimation of Stage-Specific Fibrosis Progression Rates in Chronic Hepatitis c Virus Infection: A Meta-Analysis and Meta-Regression." *Hepatology* 48 (2): 418–31. https://doi.org/https://doi.org/10.1002/hep.22375.

Veen, Maaike G. van, Anne M. Presanis, Stefano Conti, Maria Xiridou, Annemarie R. Stengaard, Martin C. Donoghoe, Ard I. van Sighem, Marianne A. van der Sande, and Daniela De Angelis. 2011. "National Estimate of HIV Prevalence in the Netherlands: Comparison and Applicability of Different Estimation Tools." *AIDS* 25 (2). https://journals.lww.com/aidsonline/Fulltext/2011/01140/National_estimate_of_HIV_prevalence_in_the.14.aspx.

# Estimating the prevalence of chronic hepatitis C infection (CHC) in Spain using Bayesian multiparameter evidence synthesis

29/05/2022

## Table of Contents

Introduction ........ 2

Methods ........ 2

Prevalence of ex-PWID ........ 3

Prevalence of recent PWID ........ 3

Prevalence of CHC among recent PWID ........ 3

Prevalence of CHC among ex-PWID ........ 4

Prevalence of CHC among non-PWID ........ 4

Sensitivity analysis considering non-PWID and ex-PWID as a combined group ........ 5

Results ........ 6

Tables and Figures ........ 7

Appendix ........ 12

Fit of the multi-state Markov model ........ 12

Stan code for Bayesian multiparameter evidence synthesis ........ 13

Multi-state Markov model ........ 20

References ........ 28

## INTRODUCTION

Patients with hepatitis C (HCV) infection often remain asymptomatic for decades and even for life, but a proportion of them develop chronic hepatitis, which is a progressive disease (Thein et al. 2008). Because of the largely asymptomatic nature of HCV infection, notification data reflect national screening and testing practices and do not give accurate insights into the prevalence of HCV infection. Although there are studies that have estimated the prevalence of HCV antibodies (anti-HCV) in specific groups such as the general population, first-time blood donors, or people who inject drugs (PWID) at drug treatment centers, those alone cannot be combined to produce national estimates unless some additional information regarding the composition of each HCV risk group and its prevalence in the population are known (Sweeting et al. 2008; McDonald et al. 2014; Tan et al. 2018).

## METHODS

Bayesian multiparameter evidence synthesis (MPES) has been a popular approach to formally estimate anti-HCV or HIV prevalence (Sweeting et al. 2008; McDonald et al. 2014; Tan et al. 2018; Presanis et al. 2010; Veen et al. 2011). In this project, we extend this method to estimating the prevalence of chronic hepatitis C infection (CHC), defined as a HCV-RNA positive result [i.e., active (viremic) infection is used as a proxy of chronic disease], with the population (15-79 years) split into three main non-overlapping risk groups, i.e., recent PWID (those who have injected in the last year; without including probably those injecting for chemsex), ex-PWID, and non-PWID. A unified model is assumed including parameters associated with the CHC prevalence of recent, ex-PWID, and non-PWID (denoted by $\pi_{rec}$, $\pi_{ex}$, and $\pi_{non}$, respectively) and the prevalence of these risk groups in the population (denoted by $\rho_{rec}$, $\rho_{ex}$, and $\rho_{non}$, respectively; $\rho_{rec} + \rho_{ex} + \rho_{non} = 1$). To estimate the CHC prevalence in the whole population, $\pi$, we used the formula:

$$\pi = \pi_{rec}\rho_{rec} + \pi_{ex}\rho_{ex} + \pi_{non}\rho_{non} \quad (1)$$

Bayesian synthesis considers all available data, allowing for an evidence-based estimation of CHC prevalence in the population, with inherent uncertainty properly accounted for. Our aim is to estimate the CHC prevalence for the total (15-79) population of Spain in 2019 using sources of information over the last decade (2010-2019).

## Prevalence of ex-PWID

Data on the prevalence of ex-PWID are sparse and generally often unreliable. To overcome this limitation, we apply the method proposed by McDonald et al. (2014), which requires simulating a multi-state Markov model representing the non-PWID, recent PWID, and ex-PWID. In the multi-state Markov model, the simulation starts in 1950 and examines the population aged 15-79 years old. A certain number of 15-year-old individuals enter the simulation process each year. These numbers of 15-year-old individuals are obtained from the United Nations Statistic Division UNSD, being different for each country. The ageing of the population is also taken into account. Country-specific overall mortality data are retrieved from the Life Tables of the World Health Organization WHO. The duration of injecting career is also country-specific and obtained from the paper of Hines et al. (2020). To estimate the number of ex-PWID, the model was calibrated on the number of recent PWID provided by the Spanish National Plan on Drugs.

After applying the model for Spain, we compute the number (and the corresponding Confidence Interval - CI) of ex-PWID in 2019. The number of ex-PWID is then divided by the population size (15-79 years) in the same year, and a Normal distribution, constrained such that $\rho_{ex} \in (0,1)$, is assumed for $\rho_{ex}$. The standard deviation of this distribution is specified to approximately correspond to the respective CI, i.e. $\left(UB_{\rho_{ex}} - LB_{\rho_{ex}}\right)/(2 \times 1.96)$, where $UB_{\rho_{ex}}$ and $LB_{\rho_{ex}}$ denote the upper and lower limit of the CI.

## Prevalence of recent PWID

The number of recent PWID in Spain in 2019 is also projected by the multi-state Markov model described in the previous subsection. To estimate $\rho_{rec}$, the number of recent PWID is divided by the population size (15-79 years). Similar to the previous subsection, a Normal distribution, constrained such that $\rho_{rec} \in (0,1)$, is assumed for $\rho_{rec}$, with the standard deviation specified to correspond to the CI obtained from the multi-state Markov model.

## Prevalence of CHC among recent PWID

The prevalence (and the 95% CI) of CHC among recent PWID can be informed from studies in the EMCDDA statistical bulletin. If information on the CHC prevalence is available, the Binomial distribution in the model to inform $\pi_{rec}$ is used. Currently, CHC prevalence data on recent PWID

from a study on marginalized people using a mobile unit in Madrid in 2019 (Ryan et al. 2021) were utilized.

## Prevalence of CHC among ex-PWID

Information on CHC prevalence among ex-PWID (i.e., $\pi_{ex}$) is difficult to obtain directly for most countries. To overcome this, nationwide data on the HCV prevalence among ever users could be used. In any case, if an estimate of $\pi_{ever}$ is available, $\pi_{ex}$ can be indirectly estimated since $\pi_{ever}$ is equal to a weighted average of $\pi_{rec}$ and $\pi_{ex}$, as shown by the following formula

$$\pi_{ever} = \frac{\rho_{rec}}{\rho_{rec} + \rho_{ex}} \pi_{rec} + \frac{\rho_{ex}}{\rho_{rec} + \rho_{ex}} \pi_{ex}, \quad (2)$$

or, equivalently,

$$\pi_{ex} = \left(\pi_{ever} - \frac{\rho_{rec}}{\rho_{rec} + \rho_{ex}} \pi_{rec}\right) \times \frac{\rho_{rec} + \rho_{ex}}{\rho_{ex}}, \quad (3)$$

However, in Spain, we used data among ex-PWID (having ever injected drugs but not during the last year) from a study on marginalized people using a mobile unit in Madrid in 2019 (Ryan et al. 2021).

## Prevalence of CHC among non-PWID

To estimate the CHC prevalence among non-PWID in a country (after 2010), the ECDC database (European Centre for Disease Prevention and Control (ECDC) 2021) was used. The ECDC group has carefully and critically collected information from relevant studies across the EU/EEA region in cooperation with the focal points of each country. On the basis of the representativeness of each study and other factors (European Centre for Disease Prevention and Control (ECDC) 2016), a risk of bias score was assigned to each study, with higher values indicating higher-quality studies. When higher-quality studies are available in a country ($\geq 4$), they are used to estimate the overall CHC prevalence in that country among non-PWID. If there are no higher-quality studies, general population estimates with a lower quality ($<4$) are pooled. If no general population prevalence estimates are available, data on the prevalence of HCV in pregnant women could be used as a prevalence measure. Finally, if data on pregnant women are lacking too, first-time blood donor studies could be an option. However, if the

national focal point agrees or recommends, estimates from a neighboring country could be used instead, or we could use any other relevant information provided/suggested by the national focal point.

To estimate the CHC prevalence in the general population, we used data from a national population-based seroprevalence survey that was conducted in 2017–2018 (Estirado Gomez et al. 2021). However, some people may have been treated with direct acting antivirals (DAAs), with the sustained virologic response ($SVR$) estimated to be 96.7% (95% CI: 95.4% to 98.1%)(Lampertico et al. 2020). According to information provided by the national focal point, the total number of individuals having initiated treatment with DAAs in 2018 and 2019 is equal to 42,444. Denoting the proportion of non-PWID among individuals treated with DAAs by $\rho_{non|DAA}$, the CHC prevalence in non-PWID can be estimated by

$$\pi_{non} = \frac{N_{15,79}\rho_{non}\tilde{\pi}_{non} - 42444\rho_{non|DAA}SVR}{N_{15,79}\rho_{non}}. \quad (4)$$

where $\tilde{\pi}_{non}$ denotes the CHC estimate based solely on the data reported in Estirado Gomez et al. (2021). However, the proportion of non-PWID among treated with DAAs, $\rho_{non|DAA}$, is not available in Spain. In this report, we make the assumption that $\rho_{non|DAA}$ is equal to the proportion of non-PWID among CHC-positive individuals, Pr(Non-PWID|CHC), as estimated by our model when information on DAAs is completely ignored. That is, $\rho_{non|DAA} =$ Pr(Non-PWID|CHC) is estimated using recent anti-HCV data among recent and ever PWID from the Spanish National Plan on Drugs (Table 1) and anti-HCV data from Estirado Gomez et al. (2021).

## Sensitivity analysis considering non-PWID and ex-PWID as a combined group

Since ex-PWID may have participated in the general population study (Estirado Gomez et al. 2021), we carried out a further sensitivity analysis considering non-PWID and ex-PWID as a combined group. Thus, assuming that ex-PWID are included proportionally in Estirado Gomez et al. (2021), the overall CHC prevalence can be estimated by

$$\pi = \pi_{rec}\rho_{rec} + \pi_{non/ex}(1 - \rho_{rec}),$$

where $\pi_{non/ex}$ is still informed from Estirado Gomez et al. (2021) (where information on injection history is not available).

# RESULTS

The above-mentioned approach was used to estimate the prevalence of CHC in 2019 (considering ages between 15-79 years: 37,126,059).

The aggregated data used by our approach are briefly presented in Table 1. In Spain, there was 1 study of high quality, which included CHC data (Estirado Gomez et al. 2021). To estimate the CHC prevalence in the general population (primarily non-PWID), we used a fixed-effect meta-analytic approach, with the corresponding results, when information on DAA uptake is ignored, presented in Table 2. The prevalence of recent and ex-PWID was low in Spain (about 0.02% and 0.07%, respectively) corresponding to 9,050 (95% CI: 8,410-9,770) recent PWID and 26,360 (95% CI: 25,290-27,430) ex-PWID in the population. However, the CHC prevalence in these groups was substantial (Table 2) being 27.6% and 21.84%, respectively. This translates to 2,495 (95% CI: 1,850-3,230) and 5,754 (95% CI: 4,550-7,122) recent and ex-PWID aged 15-79 living with CHC infection in Spain in 2019. Ignoring information on DAA uptake, the CHC prevalence in the general population was 0.23% (95% CI: 0.14%-0.35%), much lower than that of the high-risk groups.

When DAAs are considered using Equation (4) assuming that $\rho_{non|DAA}$ is equal to Pr(Non-PWID|CHC) $\approx$ 94.3%, the CHC prevalence in the general population reduces to 0.12% (95% CI: 0.03%-0.25%) (Table 3). Taking all pieces of information into account, the overall CHC prevalence in Spain in 2019 was equal to 0.15% (95% CI: 0.06%-0.27%), which corresponds to 54,676 (95% CI: 21,352-101,774) individuals aged 15-79 years with CHC infection (Table 3).

The results from our model considering non-PWID and ex-PWID as a combined group are presented in Table 4. The overall CHC prevalence estimate appears to be slightly lower, resulting in 47,208 (95% CI: 13,857-94,265) individuals aged 15-79 years with CHC infection.

# TABLES AND FIGURES

*Table 1. Data (real) contributing to the estimation of CHC prevalence in Spain in 2019.*

| Parameter | Number (95%CI) | Numerator | Denominator | Notes | Year of study |
|---|---|---|---|---|---|
| $\rho_{rec}$ | 9,050 (8,410-9,770) | | | Method based on McDonald et al. | 2019 |
| $\rho_{ex}$ | 26,360 (25,290-27,430) | | | Method based on McDonald et al. | 2019 |
| $\pi_{rec}$ | | 37 | 135 | (Ryan et al. 2021) | 2019 |
| $\pi(\text{anti-HCV})_{rec}$ | | 768 | 1,370 | Spanish National Plan on Drugs | 2019 |
| $\pi_{ex}$ | | 61 | 281 | (Ryan et al. 2021) | 2019 |
| $\pi(\text{anti-HCV})_{ever}$ | | 1,878 | 3,414 | Spanish National Plan on Drugs | 2019 |
| $\pi_{non}$ | | 17 | 7,675 | ECDC database (Gomez et al.); Risk of bias>=4 | 2018 |
| $\pi(\text{anti-HCV})_{non}$ | | 66 | 7,675 | ECDC database (Gomez et al.); Risk of bias>=4 | 2018 |

*Notes*: Higher risk of bias score denotes a higher-quality study (range from 0 to 6);

*Table 2. Results from the method assuming no heterogeneity in the estimation of the CHC prevalence in the general population from different studies (i.e., similar to a fixed-effect meta-analysis).*

| Parameter | Posterior median | Posterior sd | LB (2.5%) | UB (97.5%) |
|---|---|---|---|---|
| $\rho_{rec}$ (%) | 0.02 | 0 | 0.02 | 0.03 |
| $\rho_{ex}$ (%) | 0.07 | 0 | 0.07 | 0.07 |
| $\pi_{rec}$ (%) | 27.6 | 3.77 | 20.63 | 35.34 |
| $\pi_{ex}$ (%) | 21.84 | 2.46 | 17.29 | 27.01 |
| $\pi_{non}$ (%) | 0.23 | 0.06 | 0.14 | 0.35 |
| $\pi$ (%) | 0.25 | 0.06 | 0.16 | 0.38 |
| Number with CHC | 93,622 | 20,634 | 59,509 | 139,671 |
| Pr(Recent PWID\|CHC) (%) | 1.49 | 0.19 | 1.17 | 1.91 |
| Pr(Ex-PWID\|CHC) (%) | 4.23 | 0.51 | 3.37 | 5.37 |
| Pr(Non-PWID\|CHC) (%) | 94.29 | 0.69 | 92.75 | 95.43 |

*Notes*: Information on DAAs is not taken into account.

*Abbreviations*: $\rho_{rec}$, prevalence of recent PWID; $\rho_{ex}$, prevalence of ex-PWID; $\pi_{rec}$, CHC prevalence among recent PWID; $\pi_{ex}$, CHC prevalence among ex-PWID; $\pi_{non}$, CHC prevalence among non-PWID; $\pi$ overall CHC prevalence in Spain; LB, Lower Boundary; UP, Upper Boundary

*Table 3. Results from the method assuming no heterogeneity in the estimation of the CHC prevalence in the general population from different studies (i.e., similar to a fixed-effect meta-analysis).*

| Parameter | Posterior median | Posterior sd | LB (2.5%) | UB (97.5%) |
|---|---|---|---|---|
| $\rho_{rec}$ (%) | 0.02 | 0 | 0.02 | 0.03 |
| $\rho_{ex}$ (%) | 0.07 | 0 | 0.07 | 0.07 |
| $\pi_{rec}$ (%) | 27.6 | 3.79 | 20.7 | 35.3 |
| $\pi_{ex}$ (%) | 21.84 | 2.44 | 17.31 | 26.88 |
| $\pi_{non}$ (%) | 0.12 | 0.06 | 0.03 | 0.25 |
| $\pi$ (%) | 0.15 | 0.06 | 0.06 | 0.27 |
| Number with CHC | 54,676 | 20,640 | 21,352 | 101,774 |
| Pr(Recent PWID\|CHC) (%) | 4.58 | 2.64 | 2.28 | 11.93 |
| Pr(Ex-PWID\|CHC) (%) | 10.52 | 5.91 | 5.44 | 27.2 |
| Pr(Non-PWID\|CHC) (%) | 84.87 | 8.4 | 61.16 | 92.06 |

*Notes*: The number of individuals treated with DAAs is taken into account.

*Abbreviations*: $\rho_{rec}$, prevalence of recent PWID; $\rho_{ex}$, prevalence of ex-PWID; $\pi_{rec}$, CHC prevalence among recent PWID; $\pi_{ex}$, CHC prevalence among ex-PWID; $\pi_{non}$, CHC prevalence among non-PWID; $\pi$ overall CHC prevalence in Spain; LB, Lower Boundary; UP, Upper Boundary

*Table 4. Results from the method assuming two groups (recent PWID and ex-PWID/non-PWID) and no heterogeneity in the estimation of the CHC prevalence in the general population from different studies (i.e., similar to a fixed-effect meta-analysis).*

| Parameter | Posterior median | Posterior sd | LB (2.5%) | UB (97.5%) |
|---|---|---|---|---|
| $\rho_{rec}$ (%) | 0.02 | 0 | 0.02 | 0.03 |
| $\pi_{rec}$ (%) | 27.6 | 3.79 | 20.7 | 35.3 |
| $\pi_{non/ex}$ (%) | 0.12 | 0.06 | 0.03 | 0.25 |
| $\pi$ (%) | 0.13 | 0.06 | 0.04 | 0.25 |
| Number with CHC | 47,208 | 20,649 | 13,857 | 94,265 |
| Pr(Recent PWID\|CHC) (%) | 5.3 | 44.34 | 2.45 | 18.18 |
| Pr(Non/Ex-PWID\|CHC) (%) | 94.7 | 44.34 | 81.82 | 97.55 |

*Notes*: The number of individuals treated with DAAs is taken into account.

*Abbreviations*: $\rho_{rec}$, prevalence of recent PWID; $\pi_{rec}$, CHC prevalence among recent PWID; $\pi_{non/ex}$, CHC prevalence among non/ex-PWID; $\pi$ overall CHC prevalence in Spain; LB, Lower Boundary; UP, Upper Boundary

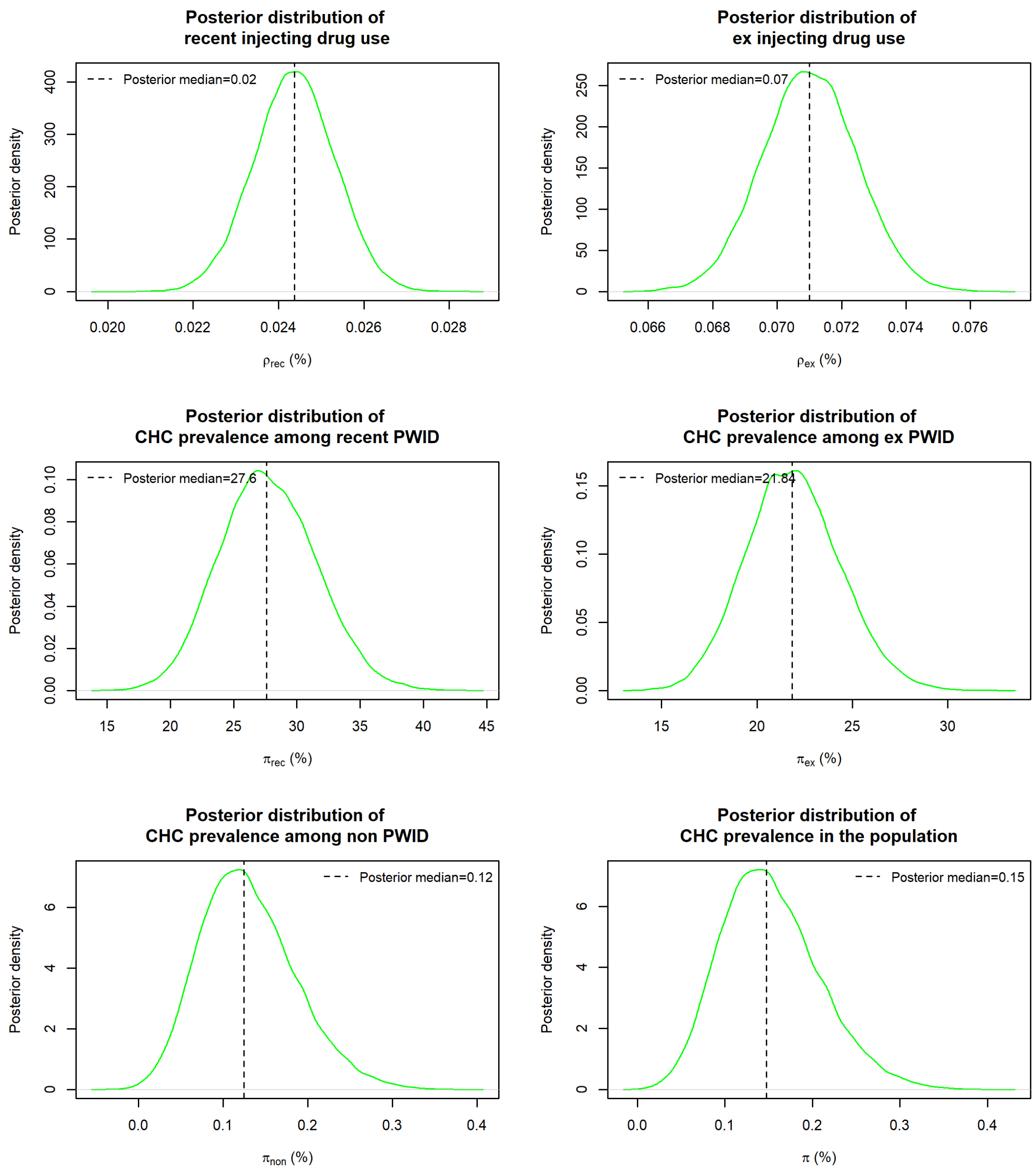


*Figure 1. Posterior distributions of the key model parameters using the approach that assumes no heterogeneity in the estimation of the CHC prevalence in the general population (primarily non-PWID) from different studies (i.e., similar to a fixed-effect meta-analysis), with the number of individuals treated with DAA taken into account.*

# APPENDIX

## Fit of the multi-state Markov model

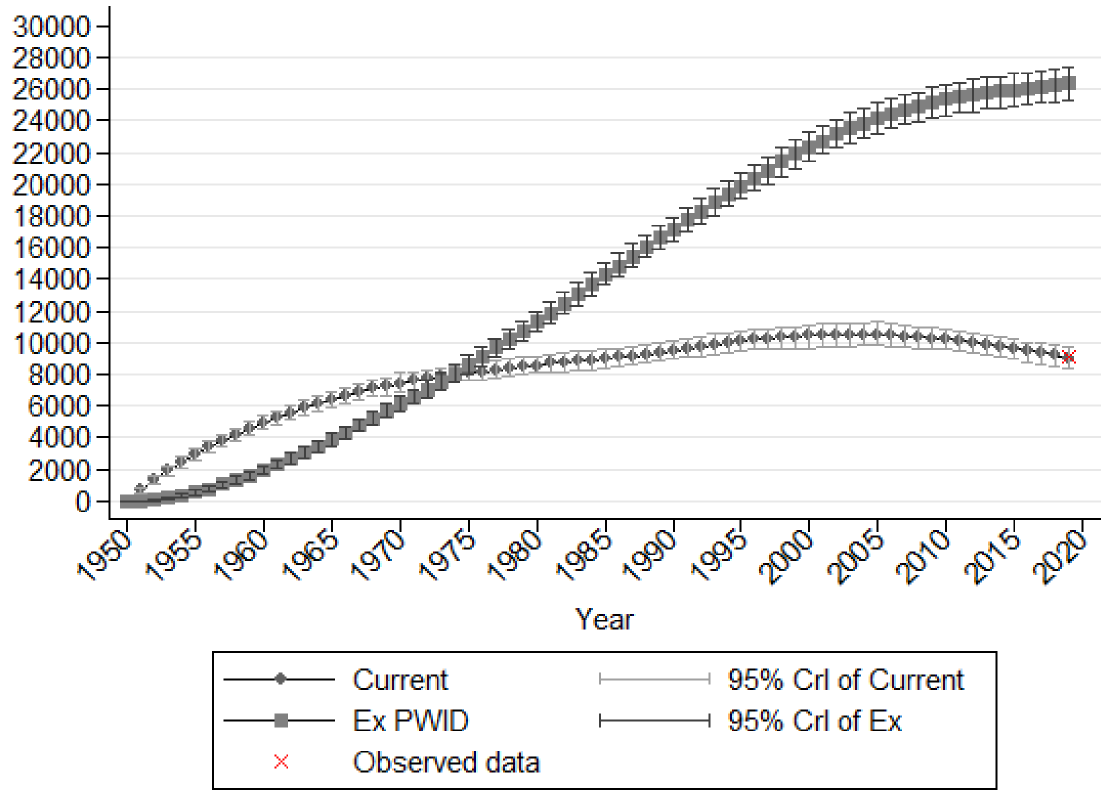


*Figure 2: Model predictions for the numbers of current and ex-PWID (people who inject drugs). The solid black line and shaded grey error bars show the median and 95% credible intervals (95% CrI) for the model projections. For comparison, asterisks indicate the observed country's data.*

## Stan code for Bayesian multiparameter evidence synthesis

```
data {
  int<lower=0> N1579; // Population of 15-64 in `Country`
  int<lower=1> Knon; // Number of studies in the ECDC data for `Country`
  int<lower=1> Kever; // Number of studies in the EMCDDA data for ever us
ers in `Country`
  int<lower=1> Kcur; // Number of studies in the EMCDDA barometer (for HC
V prevalence of PWID) in `Country`
  int<lower=1> Kex; // Number of studies for ex
  int<lower=1> NDAA_non; // Relevant number of DAAs in relation to the no
n-PWID study

  real p_cur_mean; // Prior mean for the prevalence of current use in `Co
untry`
  real<lower=0> p_cur_sd; // Prior sd for the prevalence of current use i
n `Country`

  real p_ex_mean; // Prior mean for the prevalence of ex-use in `Country`
  real<lower=0> p_ex_sd; // Prior sd for the prevalence of ex-use in `Cou
ntry`

  int<lower=0> Nst_hcv_cur[Kcur]; // Number of individuals in the study e
stimating anti-HCV among recent PWID
  int<lower=0> Yst_hcv_cur[Kcur]; // Number of HCV+ in the study estimati
ng anti-HCV among recent PWID

  int<lower=0> Nst_CHC_cur[Kcur]; // Number of individuals in the study e
stimating CHC among recent PWID
  int<lower=0> Yst_CHC_cur[Kcur]; // Number of HCV+ in the study estimati
ng CHC among recent PWID

  int<lower=0> Nst_hcv_ever[Kever]; // Number of individuals in the study
```

```
estimating HCV among ever IDU
  int<lower=0> Yst_hcv_ever[Kever]; // Number of HCV+ in the study estima
ting HCV among ever PWID

  int<lower=0> Nst_CHC_ex[Kex]; // Number of individuals in the study est
imating CHC among ex-PWID
  int<lower=0> Yst_CHC_ex[Kex]; // Number of HCV+ in the study estimating
CHC among ex-PWID

  int<lower=0> Nst_CHC_non[Knon]; // Number of individuals in the study e
stimating CHC among non PWID
  int<lower=0> Yst_CHC_non[Knon]; // Number of HCV+ in the study estimati
ng CHC among non PWID

  int<lower=0> Nst_hcv_non[Knon]; // Number of individuals in the study e
stimating anti-HCV among non PWID
  int<lower=0> Yst_hcv_non[Knon]; // Number of HCV+ in the study estimati
ng anti-HCV among non PWID

  vector<lower=0>[3] alpha; // parameter of the Diriclet prior

  real HCVclear_mean; // Prior mean for the HCV clearance probability
  real<lower=0> HCVclear_sd; // Prior sd for the HCV clearance probabilit
y

  real SVR_mean; // Prior mean for the SVR among non-PWID
  real<lower=0> SVR_sd; // Prior sd for the SVR among non-PWID

  real SVR_PWID_mean; // Prior mean for the SVR among PWID
  real<lower=0> SVR_PWID_sd; // Prior sd for the SVR among PWID
}
```

```
// Block defining the original parameters
parameters {
  // The parameters to be sampled
  simplex[3] rho; // Prevalence of the three risk groups
  real<lower=0,upper=1> CHCpi_cur; // P(CHC+|Cur)
  real<lower=0,upper=1> CHCpi_ex; // P(CHC+|Ex)
  real<lower=0,upper=1> CHCpi_non; // P(CHC+|Non)
  real<lower=0,upper=1> pi_non; // P(anti-HCV|Non)
  real<lower=0,upper=1> pi_cur; // P(anti-HCV|Cur)
  real<lower=0,upper=1> pi_ever; // P(anti-HCV|Ever)

  real<lower=0,upper=1> SVR; // SVR of DAAs among non-PWID
  real<lower=0,upper=1> SVR_PWID; // SVR of DAAs among PWID

  real<lower=0,upper=1-CHCpi_cur> HCVclear; // Probability of HCV clearan
ce; upper bound = 1-prevalence of chronic HCV
}


transformed parameters {
  // Change scales
  real<lower=0,upper=1> rho_ever;
  real<lower=0,upper=1> CHCpi_ever;
  real<lower=0,upper=1> pi_ex;

  rho_ever = rho[1] + rho[2];
  CHCpi_ever = CHCpi_cur*rho[1]/rho_ever + CHCpi_ex*rho[2]/rho_ever;
  pi_ex = (pi_ever - (rho[1]/rho_ever)*pi_cur)*rho_ever/rho[2];
}


// Binomial regression model
```

```
model {
  // Priors
  rho ~ dirichlet(alpha);

  //////////////////////////////
  // Likelihood contributions //
  //////////////////////////////

  // Probability of HCV clearance
  HCVclear ~ normal(HCVclear_mean,HCVclear_sd);

  // Prevalence of current use
  rho[1] ~ normal(p_cur_mean,p_cur_sd);

  // Prevalence of ex-use
  rho[2] ~ normal(p_ex_mean,p_ex_sd);

  // Prevalence of anti-HCV among current users
  Yst_hcv_cur ~ binomial(Nst_hcv_cur,pi_cur);

    // Prevalence of anti-HCV among current users
  Yst_CHC_cur ~ binomial(Nst_CHC_cur,CHCpi_cur);

  // Prevalence of anti-HCV among ever users
  Yst_hcv_ever ~ binomial(Nst_hcv_ever,pi_ever);

    // Prevalence of CHC among ever users
  Yst_CHC_ex ~ binomial(Nst_CHC_ex,CHCpi_ex);

  // HCV+ among non
  Yst_CHC_non ~ binomial(Nst_CHC_non,CHCpi_non);
```

```
  // anti-HCV among non
  Yst_hcv_non ~ binomial(Nst_hcv_non,pi_non);

  // SVR of DAAs among non-PWID
  SVR ~ normal(SVR_mean,SVR_sd);

  // SVR of DAAs among PWID
  SVR_PWID ~ normal(SVR_PWID_mean,SVR_PWID_sd);
}



generated quantities {
  // Functions of parameters
  real<lower=0,upper=100> overalCHC;
  real logit_CHCpi_cur;
  real logit_CHCpi_ex;
  real logit_CHCpi_non;
  real logit_rho_cur;
  real logit_rho_ex;
  real logit_rho_non;
  real<lower=0,upper=1> pEverGivenCHC;
  real<lower=0,upper=1> pCurGivenCHC;
  real<lower=0,upper=1> pExGivenCHC;
  real<lower=0,upper=1> pNonGivenCHC;
  real logit_HCVclear;
  real<lower=0> NumberCHC;

  real<lower=0,upper=1> overalCHC_adj;
  real<lower=0,upper=1> pEverGivenCHC_adj;
  real<lower=0,upper=1> pCurGivenCHC_adj;
  real<lower=0,upper=1> pExGivenCHC_adj;
  real<lower=0,upper=1> pNonGivenCHC_adj;
```

```
  real<upper=1> CHCDAApi_non; // P(CHC+|Non) adjusted for DAA
  real<lower=0,upper=100> overalCHC_DAA;
  real<lower=0,upper=1> pEverGivenCHC_DAA;
  real<lower=0,upper=1> pCurGivenCHC_DAA;
  real<lower=0,upper=1> pExGivenCHC_DAA;
  real<lower=0,upper=1> pNonGivenCHC_DAA;
  real<lower=0> NumberCHC_DAA;

  // Overall HCV prevalence
  overalCHC = 100*(rho[1]*CHCpi_cur + rho[2]*CHCpi_ex +  rho[3]*CHCpi_non
);
  pEverGivenCHC = CHCpi_ever*rho_ever/(overalCHC/100);
  pCurGivenCHC = CHCpi_cur*rho[1]/(overalCHC/100);
  pExGivenCHC = CHCpi_ex*rho[2]/(overalCHC/100);
  pNonGivenCHC = CHCpi_non*(1-rho_ever)/(overalCHC/100);
  NumberCHC = round(overalCHC*N1579/100);

  overalCHC_adj = rho[1]*pi_cur + rho[2]*pi_ex +  rho[3]*pi_non;
  pEverGivenCHC_adj = pi_ever*rho_ever/(overalCHC_adj);
  pCurGivenCHC_adj = pi_cur*rho[1]/(overalCHC_adj);
  pExGivenCHC_adj = pi_ex*rho[2]/(overalCHC_adj);
  pNonGivenCHC_adj = pi_non*rho[3]/(overalCHC_adj);

  CHCDAApi_non = ( N1579*rho[3]*CHCpi_non - NDAA_non*pNonGivenCHC_adj*SVR
)/(N1579*rho[3]);
  overalCHC_DAA = 100*(rho[1]*CHCpi_cur + rho[2]*CHCpi_ex +  rho[3]*CHCDA
Api_non);
  pEverGivenCHC_DAA = CHCpi_ever*rho_ever/(overalCHC_DAA/100);
  pCurGivenCHC_DAA = CHCpi_cur*rho[1]/(overalCHC_DAA/100);
  pExGivenCHC_DAA = CHCpi_ex*rho[2]/(overalCHC_DAA/100);
  pNonGivenCHC_DAA = CHCDAApi_non*(1-rho_ever)/(overalCHC_DAA/100);
```

```
  NumberCHC_DAA = round(overalCHC_DAA*N1579/100);

  logit_rho_cur = logit(rho[1]);
  logit_rho_ex = logit(rho[2]);
  logit_rho_non = logit(rho[3]);
  logit_CHCpi_cur = logit(CHCpi_cur);
  logit_CHCpi_ex = logit(CHCpi_ex);
  logit_CHCpi_non = logit(CHCpi_non);
  logit_HCVclear = logit(HCVclear);
}
```

## Multi-state Markov model

```
#include <stdio.h>
#include <stdlib.h>
#include <string.h>
#include <math.h>
#include <time.h>

#define loops 100
int populationPerYearAndAge[2021][66];
double deathRatePerAge[66];
int totalPopulationPerYear[2021];

FILE *F_DeathRate=fopen("`Country`-deathRates.txt","r");
FILE *F_Population=fopen("`Country`-population.txt","r");
FILE *out;

double pa_start = 0.00012;                      // Setting Variable
double pa_step  = 0.000002;                     // Setting Variable
double pa_stop  = 0.000123;                     // Setting Variable

double pa;
double pg = 1.0/11.7;    // Assumed average active injecting career of  11
.7 years
double pk = 0.004*12.0;     // Relapse rate of 0.004/month

struct people{
    int age;
    int state;
};

char filename[100];
struct people person[50000000];
```

```
long int totalPersons=0;

void setPopulationAge()
{
    for( int j=0; j<71; j++){
        for( int i=0; i<51; i++){
            fscanf(F_Population,"%d",&populationPerYearAndAge[1950+j][15+
i]);
        }
    }
}

void setDeathRate()
{
    for( int i=0; i<51; i++){
        fscanf(F_DeathRate,"%lf",&deathRatePerAge[15+i*5]);
        for( int j=0; j<5; j++){
            deathRatePerAge[15+i*5+j] = deathRatePerAge[15+i*5];
        }
    }
}

void getTotalPopulationPerAge()
{
    for( int j=0; j<71; j++){
        for( int i=0; i<51; i++){
            totalPopulationPerYear[1950+j] += populationPerYearAndAge[195
0+j][15+i];
        }
    }
}
```

```
void printTotalPopulationPerAge()
{
    printf("---- - ----------\n");
    printf("Year - Population\n");
    printf("---- - ----------\n");
    for( int i=1950; i<2021; i++){
        printf("%d - %d\n",i,totalPopulationPerYear[i]);
    }
}

void printDeathRatePerAge()
{
    printf("--- - -----\n");
    printf("Age - Rate\n");
    printf("--- - -----\n");
    for( int i=15; i<65; i++){
        printf("%d - %lf\n",i,deathRatePerAge[i]);
    }
}

void printTotalPersonPerState( int year)
{
    int count[4]= {0,0,0,0};
    for( int i=0; i<totalPersons; i++){
        count[person[i].state]++;
    }
    //printf("%d\t%ld\t%ld\t%ld\t%ld\n",year,count[0],count[1],count[2],c
ount[3]);
    if(year==2014)
        printf("%d\t%ld\t%ld\t%ld\t%ld\t%lf\n",year,count[0],count[1],cou
nt[2],count[3],pa);
    if( year>2009){
```

```
        fprintf(out, "%d\t%ld\t%ld\t%ld\t%ld\n",year,count[0],count[1],co
unt[2],count[3]);
    }
}

void initializePopulation()
{
    int cnt=0;
    for( int j=15; j<65; j++){
        for( int i=0; i<populationPerYearAndAge[1950][j]*100; i++){
            person[cnt].age=j;
            person[cnt].state=0;
            cnt++;
        }
    }
    totalPersons = cnt;
}

bool checkRate(double rate)
{
    if (rate <0.001){
        double k = rand()/(1.00+RAND_MAX);
        if(k<0.001){
            rate = rate*1000;
        }
        else{
            return false;
        }
    }
    double k = rand()/(1.00+RAND_MAX);
    if ( k < rate )
        return true;
```

```
    else
        return false;
}

void changeStatusAndAge()
{
    for( int i=0; i<totalPersons; i++){
        // change state
        double k = rand()/(1.00+RAND_MAX);
        if( person[i].state==0){
            if ( checkRate(pa) &&  person[i].age<40){
                person[i].state=1;
            }
        }
        else if( person[i].state==1){
            if ( k < pg ){
                person[i].state=2;
            }
        }
        else if( person[i].state==2){
            if ( k < pk ){
                person[i].state=1;
            }
        }
        // After their 49th year, all active PWID are assumed to cease in
jecting
        if(person[i].age>49 && person[i].state==1){
            person[i].state=2;
        }
        // After their 64th year, remove
        if(person[i].age>64){
            person[i].state=3;
```

```
        }
        // death rate depending on age and state
        k = rand()/(1.00+RAND_MAX);
        if( person[i].state==0){
            if ( k < deathRatePerAge[person[i].age] ){
                person[i].state=3;
            }
        }
        else if( person[i].state==1){
            if ( k < deathRatePerAge[person[i].age] * 13.0 ){
                person[i].state=3;
            }
        }
        else if( person[i].state==2){
            if ( k < deathRatePerAge[person[i].age] ){
                person[i].state=3;
            }
        }
        // increase age one year
        person[i].age++;
    }
}

void addNewPersons(int year)
{
    // add new person [15 years old and non PWID]
    int cnt=totalPersons;
    for( int i=0; i<populationPerYearAndAge[year+1][15]*100; i++){
// 100 instead of 1000 for the population to reduce execution time
        person[cnt].age=15;
        person[cnt].state=0;
        cnt++;
```

```
    }
    // increase the total number of population
    totalPersons = cnt;
}
int main()
{
    srand(time(NULL));

    setPopulationAge();
    setDeathRate();

    getTotalPopulationPerAge();
    pa = pa_start;
    while ( pa < pa_stop){

        snprintf(filename, 100, "result_%lf.txt",pa);
        out=fopen(filename,"w");

        for( int iter=0; iter<loops ;iter++){
            initializePopulation();
            for( int year=1950; year<2020; year++){
                printTotalPersonPerState(year);
                changeStatusAndAge();
                addNewPersons(year);
            }
        }

        fclose(out);
        pa = pa + pa_step;
    }
```

```
}
```

# REFERENCES


Estirado Gomez, Alicia, Soledad Justo Gil, Aurora Limia, Ana Avellon, Araceli Arce Arnaez, Raquel Gonzalez-Rubio, Asuncion Diaz, Julia del Amo, and the Working group of the HCV prevalence study in Spain in 2017-2018. 2021. “Prevalence and undiagnosed fraction of hepatitis C infection in 2018 in Spain: results from a national population-based survey.” *European Journal of Public Health* 31 (6): 1117–22. https://doi.org/10.1093/eurpub/ckab069.

European Centre for Disease Prevention and Control (ECDC). 2016. “Systematic Review on Hepatitis b and c Prevalence in the EU/EEA.” https://www.ecdc.europa.eu/sites/default/files/media/en/publications/Publications/systematic-review-hepatitis-B-C-prevalence.pdf.

———. 2021. “Hepatitis c Prevalence Database.” https://www.ecdc.europa.eu/en/all-topics-z/hepatitis-c/tools/hepatitis-c-prevalence-database.

Hines, Lindsey A., Adam Trickey, Janni Leung, Sarah Larney, Amy Peacock, Louisa Degenhardt, Samantha Colledge, et al. 2020. “Associations Between National Development Indicators and the Age Profile of People Who Inject Drugs: Results from a Global Systematic Review and Meta-Analysis.” *The Lancet Global Health* 8 (1): e76–91. https://doi.org/10.1016/S2214-109X(19)30462-0.

Lampertico, Pietro, Jose A. Carrión, Michael Curry, Juan Turnes, Markus Cornberg, Francesco Negro, Ashley Brown, et al. 2020. “Real-World Effectiveness and Safety of Glecaprevir/Pibrentasvir for the Treatment of Patients with Chronic HCV Infection: A meta-Analysis.” *Journal of Hepatology* 72 (6): 1112–21. https://doi.org/10.1016/j.jhep.2020.01.025.

McDonald, Scott A., Rosmawati Mohamed, Maznah Dahlui, Herlianna Naning, and Adeeba Kamarulzaman. 2014. “Bridging the Data Gaps in the Epidemiology of Hepatitis c Virus Infection in Malaysia Using Multi-Parameter Evidence Synthesis.” *BMC Infectious Diseases* 14 (1): 564. https://doi.org/10.1186/s12879-014-0564-6.

Presanis, Anne M., O. Noel Gill, Timothy R. Chadborn, Caterina Hill, Vivian Hope, Louise Logan, Brian D. Rice, Valerie C. Delpech, A. E. Ades, and Daniela De Angelis. 2010. “Insights into the Rise

in HIV Infections, 2001 to 2008: A Bayesian Synthesis of Prevalence Evidence." *AIDS* 24 (18). https://journals.lww.com/aidsonline/Fulltext/2010/11270/Insights_into_the_rise_in_HIV_infections,_2001_to.12.aspx.

Ryan, Pablo, Jorge Valencia, Guillermo Cuevas, Juan Torres-Macho, Jesús Troya, Ángel Pueyo, María José Muñoz-Gómez, et al. 2021. "Detection of Active Hepatitis c in a Single Visit and Linkage to Care Among Marginalized People Using a Mobile Unit in Madrid, Spain." *International Journal of Drug Policy* 96 (October): 103424. https://www.sciencedirect.com/science/article/pii/S0955395921003297.

Sweeting, M. J., D. De Angelis, M. Hickman, and A. E. Ades. 2008. "Estimating hepatitis C prevalence in England and Wales by synthesizing evidence from multiple data sources. Assessing data conflict and model fit." *Biostatistics* 9 (4): 715–34. https://doi.org/10.1093/biostatistics/kxn004.

Tan, Sarah, Susanna Makela, Daliah Heller, Kevin Konty, Sharon Balter, Tian Zheng, and James H. Stark. 2018. "A Bayesian Evidence Synthesis Approach to Estimate Disease Prevalence in Hard-to-Reach Populations: Hepatitis c in New York City." *Epidemics* 23: 96–109. https://doi.org/https://doi.org/10.1016/j.epidem.2018.01.002.

Thein, Hla-Hla, Qilong Yi, Gregory J. Dore, and Murray D. Krahn. 2008. "Estimation of Stage-Specific Fibrosis Progression Rates in Chronic Hepatitis c Virus Infection: A Meta-Analysis and Meta-Regression." *Hepatology* 48 (2): 418–31. https://doi.org/https://doi.org/10.1002/hep.22375.

Veen, Maaike G. van, Anne M. Presanis, Stefano Conti, Maria Xiridou, Annemarie R. Stengaard, Martin C. Donoghoe, Ard I. van Sighem, Marianne A. van der Sande, and Daniela De Angelis. 2011. "National Estimate of HIV Prevalence in the Netherlands: Comparison and Applicability of Different Estimation Tools." *AIDS* 25 (2). https://journals.lww.com/aidsonline/Fulltext/2011/01140/National_estimate_of_HIV_prevalence_in_the.14.aspx.

# Estimating the prevalence of chronic hepatitis C infection (CHC) in Sweden using Bayesian multiparameter evidence synthesis

19/06/2022

## Table of Contents

Introduction ..... 2
Methods ..... 2
Prevalence of ex-PWID ..... 3
Prevalence of recent PWID ..... 3
Prevalence of CHC among recent PWID ..... 4
Prevalence of CHC among ex-PWID ..... 4
Prevalence of CHC among non-PWID ..... 5
Sensitivity analysis including migrants from endemic countries ..... 6
Results ..... 6
Limitations ..... 7
Tables and Figures ..... 9
Appendix ..... 14
Fit of the multi-state Markov model ..... 14
Stan code for Bayesian multiparameter evidence synthesis ..... 15
Multi-state Markov model ..... 21
References ..... 29

# INTRODUCTION

Patients with chronic hepatitis C (HCV) infection often remain asymptomatic for decades and even for life, but a proportion of them develop active chronic hepatitis, which is a progressive disease (Thein et al. 2008). Because of the largely asymptomatic nature of HCV infection, notification data reflect national screening and testing practices and do not give accurate insights into the prevalence of HCV infection. Although there are studies that have estimated the prevalence of HCV antibodies (anti-HCV) in specific groups such as the general population, first-time blood donors, or people who inject drugs (PWID) at drug treatment centers, those alone cannot be combined to produce national estimates unless some additional information regarding the composition of each HCV risk group and its prevalence in the population are known (Sweeting et al. 2008; McDonald et al. 2014; Tan et al. 2018).

# METHODS

Bayesian multiparameter evidence synthesis (MPES) has been a popular approach to formally estimate anti-HCV or HIV prevalence (Sweeting et al. 2008; McDonald et al. 2014; Tan et al. 2018; Presanis et al. 2010; Veen et al. 2011). In this project, we extend this method to estimating the CHC prevalence, with the population (15-79 years) split into three main non-overlapping risk groups, i.e., recent PWID (those who have injected in the last year), ex-PWID, and non-PWID. A unified model is assumed including parameters associated with the CHC prevalence of recent, ex-PWID, and non-PWID (denoted by $\pi_{rec}$, $\pi_{ex}$, and $\pi_{non}$, respectively) and the prevalence of these risk groups in the population (denoted by $\rho_{rec}$, $\rho_{ex}$, and $\rho_{non}$, respectively; $\rho_{rec} + \rho_{ex} + \rho_{non} = 1$). To estimate the CHC prevalence in the whole population, $\pi$, we used the formula:

$$\pi = \pi_{rec}\rho_{rec} + \pi_{ex}\rho_{ex} + \pi_{non}\rho_{non} \quad (1)$$

Bayesian synthesis considers all available data, allowing for an evidence-based estimation of CHC prevalence in the population, with inherent uncertainty properly accounted for. Our aim is to estimate the CHC prevalence for the total (15-79) population of Sweden in 2019 using sources of information over the last decade (2010-2019).

## Prevalence of ex-PWID

Data on the prevalence of ex-PWID are sparse and generally often unreliable. To overcome this limitation, we apply the method proposed by McDonald et al. (2014), which requires simulating a multi-state Markov model representing the non-PWID, recent PWID, and ex-PWID. In the multi-state Markov model, the simulation starts in 1950 and examines the population aged 15-79 years old. A certain number of 15-year-old individuals enter the simulation process each year. These numbers of 15-year-old individuals are obtained from the United Nations Statistic Division UNSD, being different for each country. The ageing of the population is also taken into account. Country-specific overall mortality data are retrieved from the Life Tables of the World Health Organization WHO. The duration of injecting career is also country-specific and obtained from the paper of Hines et al. (2020).

To estimate the number of ex-PWID, the model can be calibrated on the number of recent PWID provided in the EMCDDA barometer or the EMCDDA statistical bulletin (the code of the Markov model can be found in the Appendix). If there are no available data in the barometer, estimates from the systematic review of Grebely et al. (2019) could be used. Alternatively, if the national focal points suggest or provide different and updated or more accurate data for calibration purposes, we will consider their advice and adjust the model accordingly.

After applying the model for Sweden, we compute the number (and the corresponding Confidence Interval - CI) of ex-PWID in 2019. The number of ex-PWID is then divided by the population size (15-79 years) in the same year, and a Normal distribution, constrained such that $\rho_{ex} \in (0,1)$, is assumed for $\rho_{ex}$. The standard deviation of this distribution is specified to approximately correspond to the respective CI, i.e. $(UB_{\rho_{ex}} - LB_{\rho_{ex}})/(2 \times 1.96)$, where $UB_{\rho_{ex}}$ and $LB_{\rho_{ex}}$ denote the upper and lower limit of the CI.

## Prevalence of recent PWID

The number of recent PWID in Sweden in 2019 is also projected by the multi-state Markov model described in the previous subsection. To estimate $\rho_{rec}$, the number of recent PWID is divided by the population size (15-79 years). Similar to the previous subsection, a Normal distribution, constrained such that $\rho_{rec} \in (0,1)$, is assumed for $\rho_{rec}$, with the standard deviation specified to correspond to the CI obtained from the multi-state Markov model.

## Prevalence of CHC among recent PWID

The prevalence of CHC among recent PWID was informed by CHC prevalence data from the Stockholm needle exchange programme (NEP) (Kaberg et al. 2018) in 2013-2016, reported also in the country feedback document returned to ECDC by the focal point. The Binomial distribution was used in the model. However, some people may have been treated with direct-acting antivirals (DAAs), with the sustained virologic response ($SVR_{PWID}$) among PWID estimated to be 88% (95% CI: 80% to 93%) (Graf et al. 2019). Based on the information provided by the national focal point, the number of individuals treated with DAAs up to 2019 is equal to 18,300. However, the proportions of each risk group among those treated with DAAs, i.e. $\rho_{rec|DAA}$, $\rho_{ex|DAA}$, and $\rho_{non|DAA}$ ($\rho_{rec|DAA} + \rho_{ex|DAA} + \rho_{non|DAA} = 1$), are not currently available in Sweden. In this report, we make the assumption that $\rho_{rec|DAA}$ is equal to the proportion of recent PWID among CHC-positive individuals, i.e. Pr(Recent PWID|CHC), as estimated by our model when the DAA uptake is ignored (Table 2). Similarly, we assume that $\rho_{ex|DAA}$ = Pr(Ex-PWID|CHC) and $\rho_{non|DAA}$ = Pr(Non-PWID|CHC). Thus, the CHC prevalence among recent PWID, adjusted for DAAs, can be estimated by

$$\pi_{rec} = \frac{N_{15,79}\rho_{rec}\tilde{\pi}_{rec} - N_{DAA}\rho_{rec|DAA}SVR_{PWID}}{N_{15,79}\rho_{rec}}, \quad (2)$$

where $\tilde{\pi}_{rec}$ denotes the CHC estimate derived solely by the data reported in Kaberg et al. (2018).

## Prevalence of CHC among ex-PWID

Information on CHC prevalence among ex-PWID (i.e., $\pi_{ex}$) is difficult to obtain directly. To overcome this, anti-HCV prevalence data from ever PWID in 2013, available in the EMCDDA statistical bulletin, were used. However, as these data refer to the anti-HCV prevalence, they are adjusted according to the procedure described in the previous subsection, i.e.

$$\pi_{ever} = \frac{N_{15,79}\rho_{ever}\pi(\text{anti-HCV})_{ever}(1-\rho_{clear}) - N_{DAA}\rho_{ever|DAA}SVR_{PWID}}{N_{15,79}\rho_{ever}}. \quad (3)$$

where $\pi(\text{anti-HCV})_{ever}$ denotes the anti-HCV prevalence among ever PWID, $\rho_{ever} = \rho_{rec} + \rho_{ex}$, $\rho_{clear}$ denotes the spontaneous viral clearance [assumed to be equal to 0.26 (95% CI 0.22–0.29); (Micallef, Kaldor, and Dore 2006)], and $\rho_{ever|DAA}$ the proportion of ever PWID among

individuals treated with DAAs. Recall that it is assumed that $\rho_{ever|DAA}$ is assumed to be equal to Pr(Ever PWID|CHC), as estimated by our model ignoring the effect of DAAs.

Then, since an estimate of $\pi_{ever}$ is available, $\pi_{ex}$ can be indirectly estimated since $\pi_{ever}$ is equal to a weighted average of $\pi_{rec}$ and $\pi_{ex}$, as shown by the following formula

$$\pi_{ever} = \frac{\rho_{rec}}{\rho_{ever}}\pi_{rec} + \frac{\rho_{ex}}{\rho_{ever}}\pi_{ex}, \quad (4)$$

or, equivalently,

$$\pi_{ex} = \left(\pi_{ever} - \frac{\rho_{rec}}{\rho_{ever}}\pi_{rec}\right) \times \frac{\rho_{ever}}{\rho_{ex}}. \quad (5)$$

## Prevalence of CHC among non-PWID

To estimate the CHC prevalence among non-PWID in a country (after 2010), the ECDC database (European Centre for Disease Prevention and Control (ECDC) 2021) was used. The ECDC group has carefully and critically collected information from relevant studies across the EU/EEA region in cooperation with the focal points of each country. On the basis of the representativeness of each study and other factors (European Centre for Disease Prevention and Control (ECDC) 2016), a risk of bias score was assigned to each study, with higher values indicating higher-quality studies. When higher-quality studies are available in a country (≥ 4), they are used to estimate the overall CHC prevalence in that country among non-PWID. If there are no higher-quality studies, general population estimates with a lower quality (<4) are pooled. If no general population prevalence estimates are available, data on the prevalence of HCV in pregnant women could be used as a prevalence measure. Finally, if data on pregnant women are lacking too, first-time blood donor studies could be an option. However, if the national focal point agrees or recommends, estimates from a neighboring country could be used instead, or we could use any other relevant information provided/suggested by the national focal point.

We directly use CHC prevalence data from studies in the general population, if available. However, CHC data in the general population in Sweden were based on pregnant women and their partners (Millbourn et al. 2020). Most CHC-positive individuals in the study of Millbourn et al. (2020) reported drug use as a risk factor for HCV and were excluded from the calculation

of the prevalence estimate. Thus, after excluding drug users, the resulting population may be very low risk and we did not perform a treatment adjustment as this would lead to CHC prevalence close to zero, which would be unreliable. In principle, we remove individuals cured with DAAs from the general population only if a recent seroprevalence study in the entire general population has been conducted and data are available.

### Sensitivity analysis including migrants from endemic countries

A sensitivity analysis including data for the viremic population among migrants based on the report of the ECDC group (European Centre for Disease Prevention and Control 2016) was carried out. Migrants from endemic countries represent 9.7% of the adult population in Sweden (Table 8 in European Centre for Disease Prevention and Control (2016)), with the respective CHC prevalence being equal to $\pi_{mig} = 1.4\%$ (Table 9 in European Centre for Disease Prevention and Control (2016)). The overall CHC prevalence is now estimated by

$$\pi = \pi_{rec}\rho_{rec} + \pi_{ex}\rho_{ex} + \pi_{mig}\rho_{mig} + \pi_{non}\rho_{non},$$

where $\rho_{rec} + \rho_{ex} + \rho_{mig} + \rho_{non} = 1$. A treatment adjustment similar to that described in the previous subsections was also performed.

## RESULTS

The above-mentioned approach was used to estimate the prevalence of CHC in 2019 (considering ages between 15-79 years: 7,888,323).

The aggregated data used by our approach are briefly presented in Table 1. The multi-state Markov model was calibrated on the estimated prevalence of recent PWID reported in the systematic review of Grebely et al. (2019). In Sweden, there was 1 study on pregnant women and their partners, which included CHC data (Millbourn et al. 2020). To estimate the CHC prevalence in the general population (primarily non-PWID), we used a fixed-effect meta-analytic approach, with the corresponding results, when information on DAAs is ignored, presented in Table 2. However, based on data provided by the focal point, approximately 18,300 individuals were treated with DAAs from 2015 to 2019 in Sweden, with the proportion of recent PWID among the 18,300 treated individuals assumed to be equal to Pr(Recent PWID|CHC) ≈ 19.05% and the corresponding proportion of ex-PWID assumed to be

equal to Pr(Ex-PWID|CHC) ≈ 49.66% (Table 2). Recall that no adjustment for treatment was performed in the general population.

The corresponding results accounting for the DAA uptake are presented in Table 3. The prevalence of recent and ex-PWID was low in Sweden (about 0.1% and 0.2%, respectively) corresponding to 8,000 (95% CI: 7,450-8,700) recent PWID and 15,470 (95% CI: 14,670-16,150) ex-PWID in the population. However, the CHC prevalence in these groups was substantial (Table 3) being 18.77% and 25.39%, respectively. This translates to 1,503 (95% CI: 682-2,380) and 3,934 (95% CI: 1,713-6,284) recent and ex-PWID aged 15-79 living with CHC infection in Sweden in 2019. The CHC prevalence in the general population was 0.09% (95% CI: 0.03%-0.21%), much lower than that of the high-risk groups. Taking all pieces of information into account, the overall CHC prevalence in Sweden in 2019 was equal to 0.16% (95% CI: 0.07%-0.31%), which corresponds to 12,758 (95% CI: 5,174-24,732) individuals aged 15-79 years with CHC infection.

The results from our model including migrants from endemic countries as a separate group are presented in Table 4. However, this analysis comes with possible limitations; that is, including migrants as a separate group is valid only if migrants do not overlap with the remaining groups (recent PWID, ex-PWID, and non-PWID). Thus, if migrants do participate proportionally in the study(ies) in the general population, this analysis may result in biased overall CHC estimates (most probably in higher CHC prevalence estimates).

## Limitations

The analyses reported in this document have certain limitations. First, there is no recent seroprevalence study in the general population in Sweden. Therefore, as a proxy for the general population, a study (Millbourn et al. 2020) among pregnant women and their partners in 2013-2016 was used. Note also that the study of Millbourn et al. (2020) has not been evaluated by ECDC in terms of its risk for bias. Perhaps there is selection bias in how the persons were recruited and the study covers only a fraction of the pregnant women. In conclusion, it is unclear how good this study population is as a proxy for the general population.

Moreover, although the total number of individuals treated with DAAs was adequately estimated, the proportion of the three risk groups (recent, ex, and non-PWID) among those

treated was not available. In this report, we assume that these proportions are equal to the corresponding proportions of the three risk groups among CHC-positive individuals, as estimated by our model when information on DAAs is ignored. This assumption may not be entirely correct. Furthermore, we did not remove individuals cured with DAAs from the CHC-positive non-PWID population, as this would lead to a CHC prevalence among non-PWID that is very close to zero.

Another limitation is that the reinfection risk (mostly among PWIDS) was not considered.

Finally, the estimates provided in this report (without considering migrants as a separate group) are relatively lower compared to a previous estimate of about 20,000 people diagnosed and living with CHC at the end of 2018 (based on case notifications in the national register after adjusting for diseased/emmigrated and removing those who had cleared spontaneously or with DAA), as mentioned by the national focal point.

# TABLES AND FIGURES

*Table 1. Data (real) contributing to the estimation of CHC prevalence in Sweden in 2019.*

| Parameter | Number (95%CI) | Numerator | Denominator | Notes | Year of study |
|---|---|---|---|---|---|
| $\rho_{rec}$ | 8,000 (7,450-8,700) | | | Method based on McDonald et al. | 2019 |
| $\rho_{ex}$ | 15,470 (14,670-16,150) | | | Method based on McDonald et al. | 2019 |
| $\pi_{rec}$ | | 1,332 | 2,320 | Kaberg et al. (2018) | 2013-2016 |
| $\pi(\text{anti-HCV})_{ever}$ | | 60 | 62 | EMCDDA database | 2013 |
| $\pi_{non}$ | | 4† | 4,925 | Millbourn et al. (2020); Risk of bias=NA | 2013-2016 |

*Notes*: Although it looks counter-intuitive, **a higher risk of bias score denotes a higher-quality study** (range from 0 to 6); † After excluding individuals who reported drug use as a risk factor for hepatitis C.

*Table 2. Results from the method assuming no heterogeneity in the estimation of the CHC prevalence in the general population from different studies (i.e., similar to a fixed-effect meta-analysis).*

| Parameter | Posterior median | Posterior sd | LB (2.5%) | UB (97.5%) |
|---|---|---|---|---|
| $\rho_{rec}$ (%) | 0.1 | 0 | 0.09 | 0.11 |
| $\rho_{ex}$ (%) | 0.2 | 0 | 0.19 | 0.21 |
| $\pi_{rec}$ (%) | 57.43 | 1.02 | 55.42 | 59.41 |
| $\pi_{ex}$ (%) | 77.55 | 3.95 | 68.9 | 84.35 |
| $\pi_{non}$ (%) | 0.1 | 0.05 | 0.03 | 0.21 |
| $\pi$ (%) | 0.31 | 0.05 | 0.24 | 0.42 |
| Number with CHC | 24,073 | 3,621 | 18,945 | 32,810 |
| Pr(Recent PWID\|CHC) (%) | 19.05 | 2.75 | 13.89 | 24.43 |
| Pr(Ex-PWID\|CHC) (%) | 49.66 | 6.89 | 36.29 | 62.58 |
| Pr(Non-PWID\|CHC) (%) | 31.21 | 9.39 | 13.7 | 49.6 |

*Notes*: Information on DAAs is not taken into account.

*Abbreviations*: $\rho_{rec}$, prevalence of recent PWID (proportion of the population that is recent PWID); $\rho_{ex}$, prevalence of ex-PWID (proportion of the population that is ex-PWID); $\pi_{rec}$, CHC prevalence among recent PWID; $\pi_{ex}$, CHC prevalence among ex-PWID; $\pi_{non}$, CHC prevalence among non-PWID; $\pi$ overall CHC prevalence in Sweden; LB, Lower Boundary; UP, Upper Boundary

*Table 3. Results from the method assuming no heterogeneity in the estimation of the CHC prevalence in the general population from different studies (i.e., similar to a fixed-effect meta-analysis).*

| Parameter | Posterior median | Posterior sd | LB (2.5%) | UB (97.5%) |
|---|---|---|---|---|
| $\rho_{rec}$ (%) | 0.1 | 0 | 0.09 | 0.11 |
| $\rho_{ex}$ (%) | 0.2 | 0 | 0.19 | 0.21 |
| $\pi_{rec}$ (%) | 18.77 | 5.36 | 8.64 | 29.39 |
| $\pi_{ex}$ (%) | 25.39 | 7.52 | 11.13 | 40.43 |
| $\pi_{non}$ (%) | 0.09 | 0.04 | 0.03 | 0.21 |
| $\pi$ (%) | 0.16 | 0.06 | 0.07 | 0.31 |
| Number with CHC | 12,758 | 5,047 | 5,174 | 24,732 |
| Pr(Recent PWID\|CHC) (%) | 11.63 | 1.34 | 9.3 | 14.57 |
| Pr(Ex-PWID\|CHC) (%) | 30.27 | 3.94 | 23.71 | 39.03 |
| Pr(Non-PWID\|CHC) (%) | 58.11 | 5.12 | 46.83 | 66.65 |

*Notes*: The number of individuals treated with DAAs is taken into account.

*Abbreviations*: $\rho_{rec}$, prevalence of recent PWID (proportion of the population that is recent PWID); $\rho_{ex}$, prevalence of ex-PWID (proportion of the population that is ex-PWID); $\pi_{rec}$, CHC prevalence among recent PWID; $\pi_{ex}$, CHC prevalence among ex-PWID; $\pi_{non}$, CHC prevalence among non-PWID; $\pi$ overall CHC prevalence in Sweden; LB, Lower Boundary; UP, Upper Boundary

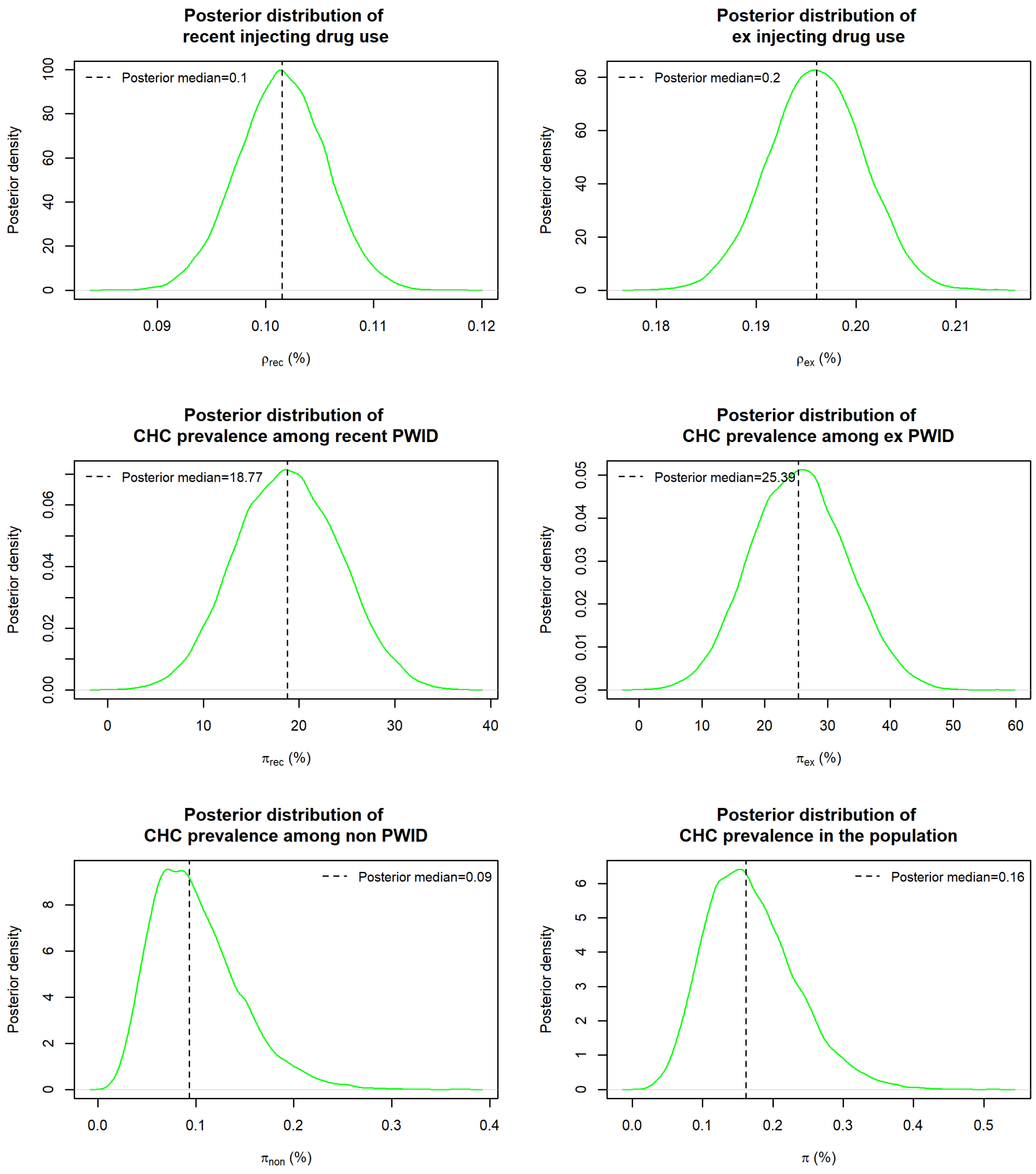


*Figure 1. Posterior distributions of the key model parameters using the approach that assumes no heterogeneity in the estimation of the CHC prevalence in the general population (primarily non-PWID) from different studies (i.e., similar to a fixed-effect meta-analysis), with the number of individuals treated with DAA taken into account.*

*Table 4. Results from the method assuming no heterogeneity in the estimation of the CHC prevalence in the general population (primarily non-PWID) from different studies (i.e., similar to a fixed-effect meta-analysis) including migrants from endemic countries as a separate group.*

| Parameter | Posterior median | Posterior sd | LB (2.5%) | UB (97.5%) |
|---|---|---|---|---|
| $\rho_{rec}$ (%) | 0.1 | 0 | 0.09 | 0.11 |
| $\rho_{ex}$ (%) | 0.2 | 0 | 0.19 | 0.21 |
| $\rho_{mig}$ (%) | 9.7 | 0 | 9.7 | 9.7 |
| $\pi_{rec}$ (%) | 30.34 | 3.19 | 23.58 | 36.12 |
| $\pi_{ex}$ (%) | 40.86 | 4.99 | 30.64 | 50.25 |
| $\pi_{mig}$ (%) | 0.67 | 0.21 | 0.31 | 1.11 |
| $\pi_{non}$ (%) | 0.09 | 0.05 | 0.03 | 0.21 |
| $\pi$ (%) | 0.26 | 0.06 | 0.16 | 0.4 |
| Number with CHC | 20,731 | 4,847 | 12,771 | 31,627 |
| Pr(Recent PWID\|CHC) (%) | 11.68 | 1.6 | 8.95 | 15.14 |
| Pr(Ex-PWID\|CHC) (%) | 30.43 | 4.22 | 23.12 | 39.55 |
| Pr(Mig\|CHC) (%) | 24.79 | 5.56 | 14.05 | 35.67 |
| Pr(Non-PWID\|CHC) (%) | 32.88 | 8.63 | 15.92 | 49.09 |

*Notes*: The number of individuals treated with DAAs is taken into account.

*Abbreviations*: $\rho_{rec}$, prevalence of recent PWID (proportion of the population that is recent PWID); $\rho_{ex}$, prevalence of ex-PWID (proportion of the population that is ex-PWID); $\rho_{mig}$, prevalence of migrants from endemic countries (proportion of the population that belongs to this group); $\pi_{rec}$, CHC prevalence among recent PWID; $\pi_{ex}$, CHC prevalence among ex-PWID; $\pi_{mig}$, CHC prevalence among migrants from endemic countries; $\pi_{non}$, CHC prevalence among non-PWID; $\pi$ overall CHC prevalence in Sweden; LB, Lower Boundary; UP, Upper Boundary

# APPENDIX

## Fit of the multi-state Markov model

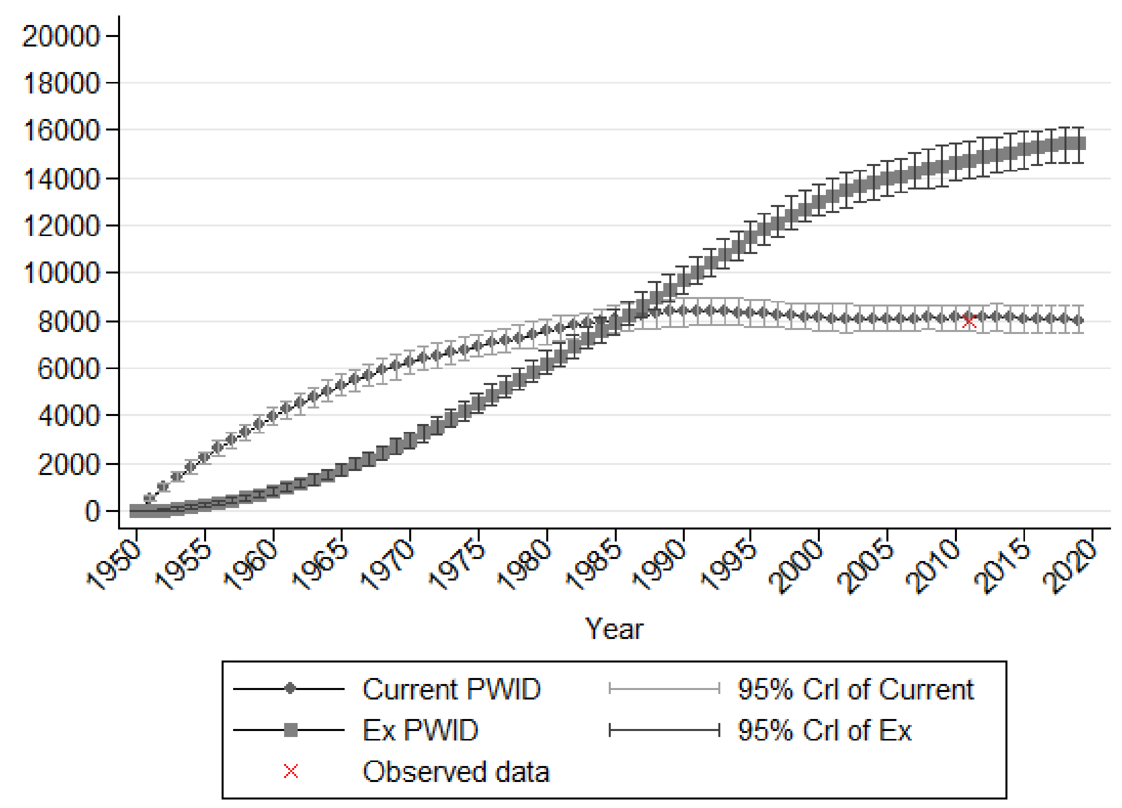


*Figure 2: Model predictions for the numbers of current and ex-PWID (people who inject drugs). The solid black line and shaded grey error bars show the median and 95% credible intervals (95% CrI) for the model projections. For comparison, asterisks indicate the observed country's data.*

## Stan code for Bayesian multiparameter evidence synthesis

```
data {
  int<lower=0> N1579; // Population of 15-79 in `Country`
  int<lower=1> Knon; // Number of studies in the ECDC data for `Country`
  int<lower=1> Kever; // Number of studies in the EMCDDA data for ever us
ers in `Country`
  int<lower=1> Kcur; // Number of studies in the EMCDDA barometer (for HC
V prevalence of PWID) in `Country`
  int<lower=1> NDAA; // Total number of DAAs from 2015 to 2019

  real p_cur_mean; // Prior mean for the prevalence of current use in `Co
untry`
  real<lower=0> p_cur_sd; // Prior sd for the prevalence of current use i
n `Country`

  real p_ex_mean; // Prior mean for the prevalence of ex-use in `Country`
  real<lower=0> p_ex_sd; // Prior sd for the prevalence of ex-use in `Cou
ntry`

  int<lower=0> Nst_CHC_cur[Kcur]; // Number of individuals in the study e
stimating CHC among recent PWID
  int<lower=0> Yst_CHC_cur[Kcur]; // Number of HCV+ in the study estimati
ng CHC among recent PWID

  int<lower=0> Nst_hcv_ever[Kever]; // Number of individuals in the study
estimating anti-HCV among ever IDU
  int<lower=0> Yst_hcv_ever[Kever]; // Number of HCV+ in the study estima
ting anti-HCV among ever PWID

  int<lower=0> Nst_CHC_non[Knon]; // Number of individuals in the study e
stimating CHC among non PWID
  int<lower=0> Yst_CHC_non[Knon]; // Number of HCV+ in the study estimati
```

```
ng CHC among non PWID

  vector<lower=0>[3] alpha; // parameter of the Diriclet prior

  real HCVclear_mean; // Prior mean for the HCV clearance probability
  real<lower=0> HCVclear_sd; // Prior sd for the HCV clearance probabilit
y

  real SVR_mean; // Prior mean for the SVR among non-PWID
  real<lower=0> SVR_sd; // Prior sd for the SVR among non-PWID

  real SVR_PWID_mean; // Prior mean for the SVR among PWID
  real<lower=0> SVR_PWID_sd; // Prior sd for the SVR among PWID
}

// Block defining the original parameters
parameters {
  // The parameters to be sampled
  simplex[3] rho; // Prevalence of the three risk groups
  real<lower=0,upper=1> CHCpi_cur; // P(CHC+|Cur)
  real<lower=0,upper=1> CHCpi_ex; // P(CHC+|Ex)
  real<lower=0,upper=1> CHCpi_non; // P(CHC+|Non)
  real<lower=0,upper=1> SVR; // SVR of DAAs among non-PWID
  real<lower=0,upper=1> SVR_PWID; // SVR of DAAs among PWID

  real<lower=0,upper=1-CHCpi_cur> HCVclear; // Probability of HCV clearan
ce; upper bound = 1-prevalence of chronic HCV
}


transformed parameters {
  // Change scales
```

```
  real<lower=0,upper=1> rho_ever;
  real<lower=0,upper=1> CHCpi_ever;
  real<lower=0,upper=1> pi_ever;
  //real<lower=0,upper=1> pi_non;

  rho_ever = rho[1] + rho[2];
  CHCpi_ever = CHCpi_cur*rho[1]/rho_ever + CHCpi_ex*rho[2]/rho_ever;
  pi_ever = CHCpi_ever/(1-HCVclear);
  //pi_non = CHCpi_non/(1-HCVclear);
}


// Binomial regression model
model {
  // Priors
  rho ~ dirichlet(alpha);

  //////////////////////////////
  // Likelihood contributions //
  //////////////////////////////

  // Probability of HCV clearance
  HCVclear ~ normal(HCVclear_mean,HCVclear_sd);

  // Prevalence of current use
  rho[1] ~ normal(p_cur_mean,p_cur_sd);

  // Prevalence of ex-use
  rho[2] ~ normal(p_ex_mean,p_ex_sd);

  // Prevalence of chronic HCV among current users
  Yst_CHC_cur ~ binomial(Nst_CHC_cur,CHCpi_cur);
```

```
  // Prevalence of HCV among ever users
  Yst_hcv_ever ~ binomial(Nst_hcv_ever,pi_ever);

  // HCV+ among non
  Yst_CHC_non ~ binomial(Nst_CHC_non,CHCpi_non);

  // SVR of DAAs among non-PWID
  SVR ~ normal(SVR_mean,SVR_sd);

  // SVR of DAAs among PWID
  SVR_PWID ~ normal(SVR_PWID_mean,SVR_PWID_sd);
}


generated quantities {
  // Functions of parameters
  real<lower=0,upper=100> overalCHC;
  real logit_CHCpi_cur;
  real logit_CHCpi_ex;
  real logit_CHCpi_non;
  real logit_rho_cur;
  real logit_rho_ex;
  real logit_rho_non;
  real<lower=0,upper=1> pEverGivenCHC;
  real<lower=0,upper=1> pCurGivenCHC;
  real<lower=0,upper=1> pExGivenCHC;
  real<lower=0,upper=1> pNonGivenCHC;
  real logit_HCVclear;
  real<lower=0> NumberCHC;

  real overalCHC_adj;
```

```
  real pEverGivenCHC_adj;
  real pCurGivenCHC_adj;
  real pExGivenCHC_adj;
  real pNonGivenCHC_adj;
  //real CHCpi_non_adj;

  //real<lower=0,upper=1> CHCDAApi_non; // P(CHC+|Non) adjusted for DAA
  real<lower=0,upper=1> CHCDAApi_cur; // P(CHC+|Cur) adjusted for DAA
  real<lower=0,upper=1> CHCDAApi_ex; // P(CHC+|Ex) adjusted for DAA
  real<lower=0,upper=1> CHCDAApi_ever; // P(CHC+|Ever) adjusted for DAA

  real<lower=0,upper=100> overalCHC_DAA;
  real overalCHC_DAA_adj;
  real pEverGivenCHC_DAA;
  real pCurGivenCHC_DAA;
  real pExGivenCHC_DAA;
  real pNonGivenCHC_DAA;
  real<lower=0> NumberCHC_DAA;

  // Overall HCV prevalence
  overalCHC = 100*(rho[1]*CHCpi_cur + rho[2]*CHCpi_ex +  rho[3]*CHCpi_non
);
  pEverGivenCHC = CHCpi_ever*rho_ever/(overalCHC/100);
  pCurGivenCHC = CHCpi_cur*rho[1]/(overalCHC/100);
  pExGivenCHC = CHCpi_ex*rho[2]/(overalCHC/100);
  pNonGivenCHC = CHCpi_non*(1-rho_ever)/(overalCHC/100);
  NumberCHC = round(overalCHC*N1579/100);

  //CHCpi_non_adj = CHCpi_non*10;
  overalCHC_adj = 100*(rho[1]*CHCpi_cur + rho[2]*CHCpi_ex +  rho[3]*CHCpi
_non);
  pEverGivenCHC_adj = CHCpi_ever*rho_ever/(overalCHC_adj/100);
```

```
  pCurGivenCHC_adj = CHCpi_cur*rho[1]/(overalCHC_adj/100);
  pExGivenCHC_adj = CHCpi_ex*rho[2]/(overalCHC_adj/100);
  pNonGivenCHC_adj = CHCpi_non*(1-rho_ever)/(overalCHC_adj/100);

  //CHCDAApi_non = ( N1579*rho[3]*CHCpi_non - NDAA_non*pNonGivenCHC*SVR)/
(N1579*rho[3]);
  CHCDAApi_cur = ( N1579*rho[1]*CHCpi_cur - NDAA*pCurGivenCHC_adj*SVR_PWI
D_mean)/(N1579*rho[1]);
  CHCDAApi_ex = ( N1579*rho[2]*CHCpi_ex - NDAA*pExGivenCHC_adj*SVR_PWID_m
ean)/(N1579*rho[2]);
  CHCDAApi_ever = CHCDAApi_cur*rho[1]/rho_ever + CHCDAApi_ex*rho[2]/rho_e
ver;

  overalCHC_DAA = 100*(rho[1]*CHCDAApi_cur + rho[2]*CHCDAApi_ex +  rho[3]
*CHCpi_non);
  overalCHC_DAA_adj = rho[1]*CHCDAApi_cur + rho[2]*CHCDAApi_ex +  rho[3]*
CHCpi_non;
  pEverGivenCHC_DAA = CHCDAApi_ever*rho_ever/(overalCHC_DAA_adj);
  pCurGivenCHC_DAA = CHCDAApi_cur*rho[1]/(overalCHC_DAA_adj);
  pExGivenCHC_DAA = CHCDAApi_ex*rho[2]/(overalCHC_DAA_adj);
  pNonGivenCHC_DAA = CHCpi_non*(1-rho_ever)/(overalCHC_DAA_adj);
  NumberCHC_DAA = round(overalCHC_DAA*N1579/100);

  logit_rho_cur = logit(rho[1]);
  logit_rho_ex = logit(rho[2]);
  logit_rho_non = logit(rho[3]);
  logit_CHCpi_cur = logit(CHCpi_cur);
  logit_CHCpi_ex = logit(CHCpi_ex);
  logit_CHCpi_non = logit(CHCpi_non);
  logit_HCVclear = logit(HCVclear);
}
```

## Multi-state Markov model

```
#include <stdio.h>
#include <stdlib.h>
#include <string.h>
#include <math.h>
#include <time.h>

#define loops 100
int populationPerYearAndAge[2021][66];
double deathRatePerAge[66];
int totalPopulationPerYear[2021];

FILE *F_DeathRate=fopen("`Country`-deathRates.txt","r");
FILE *F_Population=fopen("`Country`-population.txt","r");
FILE *out;

double pa_start = 0.00012;                      // Setting Variable
double pa_step  = 0.000002;                     // Setting Variable
double pa_stop  = 0.000123;                     // Setting Variable

double pa;
double pg = 1.0/11.7;    // Assumed average active injecting career of  11
.7 years
double pk = 0.004*12.0;     // Relapse rate of 0.004/month

struct people{
    int age;
    int state;
};

char filename[100];
struct people person[50000000];
```

```
long int totalPersons=0;

void setPopulationAge()
{
    for( int j=0; j<71; j++){
        for( int i=0; i<51; i++){
            fscanf(F_Population,"%d",&populationPerYearAndAge[1950+j][15+
i]);
        }
    }
}

void setDeathRate()
{
    for( int i=0; i<51; i++){
        fscanf(F_DeathRate,"%lf",&deathRatePerAge[15+i*5]);
        for( int j=0; j<5; j++){
            deathRatePerAge[15+i*5+j] = deathRatePerAge[15+i*5];
        }
    }
}

void getTotalPopulationPerAge()
{
    for( int j=0; j<71; j++){
        for( int i=0; i<51; i++){
            totalPopulationPerYear[1950+j] += populationPerYearAndAge[195
0+j][15+i];
        }
    }
}
```

```
void printTotalPopulationPerAge()
{
    printf("---- - ----------\n");
    printf("Year - Population\n");
    printf("---- - ----------\n");
    for( int i=1950; i<2021; i++){
        printf("%d - %d\n",i,totalPopulationPerYear[i]);
    }
}

void printDeathRatePerAge()
{
    printf("--- - -----\n");
    printf("Age - Rate\n");
    printf("--- - -----\n");
    for( int i=15; i<65; i++){
        printf("%d - %lf\n",i,deathRatePerAge[i]);
    }
}

void printTotalPersonPerState( int year)
{
    int count[4]= {0,0,0,0};
    for( int i=0; i<totalPersons; i++){
        count[person[i].state]++;
    }
    //printf("%d\t%ld\t%ld\t%ld\t%ld\n",year,count[0],count[1],count[2],c
ount[3]);
    if(year==2014)
        printf("%d\t%ld\t%ld\t%ld\t%ld\t%lf\n",year,count[0],count[1],cou
nt[2],count[3],pa);
    if( year>2009){
```

```
        fprintf(out, "%d\t%ld\t%ld\t%ld\t%ld\n",year,count[0],count[1],co
unt[2],count[3]);
    }
}

void initializePopulation()
{
    int cnt=0;
    for( int j=15; j<65; j++){
        for( int i=0; i<populationPerYearAndAge[1950][j]*100; i++){
            person[cnt].age=j;
            person[cnt].state=0;
            cnt++;
        }
    }
    totalPersons = cnt;
}

bool checkRate(double rate)
{
    if (rate <0.001){
        double k = rand()/(1.00+RAND_MAX);
        if(k<0.001){
            rate = rate*1000;
        }
        else{
            return false;
        }
    }
    double k = rand()/(1.00+RAND_MAX);
    if ( k < rate )
        return true;
```

```
    else
        return false;
}

void changeStatusAndAge()
{
    for( int i=0; i<totalPersons; i++){
        // change state
        double k = rand()/(1.00+RAND_MAX);
        if( person[i].state==0){
            if ( checkRate(pa) &&  person[i].age<40){
                person[i].state=1;
            }
        }
        else if( person[i].state==1){
            if ( k < pg ){
                person[i].state=2;
            }
        }
        else if( person[i].state==2){
            if ( k < pk ){
                person[i].state=1;
            }
        }
        // After their 49th year, all active PWID are assumed to cease in
jecting
        if(person[i].age>49 && person[i].state==1){
            person[i].state=2;
        }
        // After their 64th year, remove
        if(person[i].age>64){
            person[i].state=3;
```

```
        }
        // death rate depending on age and state
        k = rand()/(1.00+RAND_MAX);
        if( person[i].state==0){
            if ( k < deathRatePerAge[person[i].age] ){
                person[i].state=3;
            }
        }
        else if( person[i].state==1){
            if ( k < deathRatePerAge[person[i].age] * 13.0 ){
                person[i].state=3;
            }
        }
        else if( person[i].state==2){
            if ( k < deathRatePerAge[person[i].age] ){
                person[i].state=3;
            }
        }
        // increase age one year
        person[i].age++;
    }
}

void addNewPersons(int year)
{
    // add new person [15 years old and non PWID]
    int cnt=totalPersons;
    for( int i=0; i<populationPerYearAndAge[year+1][15]*100; i++){
// 100 instead of 1000 for the population to reduce execution time
        person[cnt].age=15;
        person[cnt].state=0;
        cnt++;
```

```
    }
    // increase the total number of population
    totalPersons = cnt;
}
int main()
{
    srand(time(NULL));

    setPopulationAge();
    setDeathRate();

    getTotalPopulationPerAge();
    pa = pa_start;
    while ( pa < pa_stop){

        snprintf(filename, 100, "result_%lf.txt",pa);
        out=fopen(filename,"w");

        for( int iter=0; iter<loops ;iter++){
            initializePopulation();
            for( int year=1950; year<2020; year++){
                printTotalPersonPerState(year);
                changeStatusAndAge();
                addNewPersons(year);
            }
        }

        fclose(out);
        pa = pa + pa_step;
    }
```

```
}
```

# REFERENCES

European Centre for Disease Prevention and Control. 2016. "Epidemiological Assessment of Hepatitis b and c Among Migrants in the EU/EEA." https://www.ecdc.europa.eu/sites/default/files/media/en/publications/Publications/epidemiological-assessment-hepatitis-B-and-C-among-migrants-EU-EEA.pdf.

European Centre for Disease Prevention and Control (ECDC). 2016. "Systematic Review on Hepatitis b and c Prevalence in the EU/EEA." https://www.ecdc.europa.eu/sites/default/files/media/en/publications/Publications/systematic-review-hepatitis-B-C-prevalence.pdf.

———. 2021. "Hepatitis c Prevalence Database." https://www.ecdc.europa.eu/en/all-topics-z/hepatitis-c/tools/hepatitis-c-prevalence-database.

Graf, Christiana, Marcus M Mücke, Georg Dultz, Kai-Henrik Peiffer, Alica Kubesch, Patrick Ingiliz, Stefan Zeuzem, Eva Herrmann, and Johannes Vermehren. 2019. "Efficacy of Direct-acting Antivirals for Chronic Hepatitis C Virus Infection in People Who Inject Drugs or Receive Opioid Substitution Therapy: A Systematic Review and Meta-analysis." *Clinical Infectious Diseases* 70 (11): 2355–65. https://doi.org/10.1093/cid/ciz696.

Grebely, Jason, Sarah Larney, Amy Peacock, Samantha Colledge, Janni Leung, Matthew Hickman, Peter Vickerman, et al. 2019. "Global, Regional, and Country-Level Estimates of Hepatitis c Infection Among People Who Have Recently Injected Drugs." *Addiction* 114 (1): 150–66. https://doi.org/https://doi.org/10.1111/add.14393.

Hines, Lindsey A., Adam Trickey, Janni Leung, Sarah Larney, Amy Peacock, Louisa Degenhardt, Samantha Colledge, et al. 2020. "Associations Between National Development Indicators and the Age Profile of People Who Inject Drugs: Results from a Global Systematic Review and Meta-Analysis." *The Lancet Global Health* 8 (1): e76–91. https://doi.org/10.1016/S2214-109X(19)30462-0.

Kaberg, Martin, Georg Naver, Anders Hammarberg, and Ola Weiland. 2018. "Incidence and Spontaneous Clearance of Hepatitis c Virus (HCV) in People Who Inject Drugs at the Stockholm

Needle Exchange—Importance for HCV Elimination.” *Journal of Viral Hepatitis* 25 (12): 1452–61. https://doi.org/https://doi.org/10.1111/jvh.12969.

McDonald, Scott A., Rosmawati Mohamed, Maznah Dahlui, Herlianna Naning, and Adeeba Kamarulzaman. 2014. “Bridging the Data Gaps in the Epidemiology of Hepatitis c Virus Infection in Malaysia Using Multi-Parameter Evidence Synthesis.” *BMC Infectious Diseases* 14 (1): 564. https://doi.org/10.1186/s12879-014-0564-6.

Micallef, J. M., J. M. Kaldor, and G. J. Dore. 2006. “Spontaneous Viral Clearance Following Acute Hepatitis c Infection: A Systematic Review of Longitudinal Studies.” *Journal of Viral Hepatitis* 13 (1): 34–41. https://doi.org/https://doi.org/10.1111/j.1365-2893.2005.00651.x.

Millbourn, Charlotta, Charlotte Lybeck, Afrodite Psaros Einberg, Margareta Nordin, Gudrun Lindh, Ingegerd Hokeberg, Hans Fredlund, et al. 2020. “Anti-HCV Prevalence and Risk Factor-Based Screening for Hepatitis c in Pregnant Women and Their Partners in Sweden.” *Infectious Diseases* 52 (11): 776–85. https://doi.org/10.1080/23744235.2020.1784456.

Presanis, Anne M., O. Noel Gill, Timothy R. Chadborn, Caterina Hill, Vivian Hope, Louise Logan, Brian D. Rice, Valerie C. Delpech, A. E. Ades, and Daniela De Angelis. 2010. “Insights into the Rise in HIV Infections, 2001 to 2008: A Bayesian Synthesis of Prevalence Evidence.” *AIDS* 24 (18). https://journals.lww.com/aidsonline/Fulltext/2010/11270/Insights_into_the_rise_in_HIV_infections,_2001_to.12.aspx.

Sweeting, M. J., D. De Angelis, M. Hickman, and A. E. Ades. 2008. “Estimating hepatitis C prevalence in England and Wales by synthesizing evidence from multiple data sources. Assessing data conflict and model fit.” *Biostatistics* 9 (4): 715–34. https://doi.org/10.1093/biostatistics/kxn004.

Tan, Sarah, Susanna Makela, Daliah Heller, Kevin Konty, Sharon Balter, Tian Zheng, and James H. Stark. 2018. “A Bayesian Evidence Synthesis Approach to Estimate Disease Prevalence in Hard-to-Reach Populations: Hepatitis c in New York City.” *Epidemics* 23: 96–109. https://doi.org/https://doi.org/10.1016/j.epidem.2018.01.002.

Thein, Hla-Hla, Qilong Yi, Gregory J. Dore, and Murray D. Krahn. 2008. “Estimation of Stage-Specific Fibrosis Progression Rates in Chronic Hepatitis c Virus Infection: A Meta-Analysis and

Meta-Regression." *Hepatology* 48 (2): 418–31. https://doi.org/https://doi.org/10.1002/hep.22375.

Veen, Maaike G. van, Anne M. Presanis, Stefano Conti, Maria Xiridou, Annemarie R. Stengaard, Martin C. Donoghoe, Ard I. van Sighem, Marianne A. van der Sande, and Daniela De Angelis. 2011. "National Estimate of HIV Prevalence in the Netherlands: Comparison and Applicability of Different Estimation Tools." *AIDS* 25 (2). https://journals.lww.com/aidsonline/Fulltext/2011/01140/National_estimate_of_HIV_prevalence_in_the.14.aspx.
