# Supplementary material for: Prevalence of chronic HCV infection in EU/EEA countries in 2019 using multiparameter evidence synthesis
Source: Lancet Reg Health Eur. 2023 Dec 13;36:100792. doi: 10.1016/j.lanepe.2023.100792 (PMC10769889; doi:10.1016/j.lanepe.2023.100792)
Supplement: Protocol FINAL [file mmc3.pdf]

Estimating hepatitis C burden in EU/EEA

ECDC Framework Service Contract

TECHNICAL PROPOSAL

Date of submission: June 2021

Submitted by: Dr Georgios Nikolopoulos, Medical School, University of Cyprus

Table of Contents

Background 3
Aims and objectives 4
Methods 5
Summary database and Data collection procedure 5
Main Population of interest 5
Evidence Synthesis method 6
Project tasks and planning 8
Overview of framework contract and included work packages 8
Outline of specific tasks to be performed under each work package 10
Deliverables 11
Organization involved 13
Key personnel 14
Distribution of tasks 16
References 17

Background

Hepatitis C virus infection (HCV) can cause acute and chronic hepatitis, and potentially lead to the development of cirrhosis, liver cancer, or death [1, 2]. It has been estimated that across the European Union (EU) almost 3.2 million people are chronically infected with hepatitis C [1]. In Europe, 15–80% of hepatocellular carcinoma cases are attributed to HCV [3, 4]. In response to this significant problem, the 69th World Health Assembly endorsed the Global Health Sector Strategy (GHSS) for Viral Hepatitis, with the goal to eliminate hepatitis infection as a public health threat by 2030 [5]. The targets include a 65% reduction in liver-related deaths, an 80% reduction of new HCV infections, and 90% of patients with viral hepatitis infection to be diagnosed by 2030 [5].

Patients with chronic HCV infection remain asymptomatic for decades and even for life, but a proportion of them develops active chronic hepatitis, which is a progressive disease. Approximately 20–30% of patients with chronic viral hepatitis develop cirrhosis over a period of 20–30 years and they are then at risk for liver decompensation, development of hepatocellular carcinoma (HCC), and premature liver-related death [6, 7]. Because of the asymptomatic nature of the HCV infection, notification data reflect national screening and testing practices and do not give accurate insights into the prevalence of infection. Thus, to adequately inform primary or secondary prevention efforts, supplementary information such as prevalence data are needed. A recent modelling study highlight that only 8 EU countries are likely to achieve the WHO elimination targets while most other countries were off track by at least 20 years [8].

The difficulties in estimating the magnitude of the infected HCV population in most of the EU/European Economic Area (EAA) countries are the absence of representative general population-based surveys and of studies among hard-to-reach populations at high risk of HCV infection (such as people who inject drugs (PWID)). Although there are studies that have estimated the prevalence of HCV antibodies in specific population groups, such as blood donors or PWID at addiction clinics, those alone cannot be combined to estimate national estimates unless some additional information regarding the composition of each HCV risk group and the prevalence of the risk groups in the population are known [9, 10].

Aims and objectives

This work will build upon work already initiated by the European Centre for Disease Prevention and Control (ECDC) on estimating the prevalence of HCV in EU/EEA. Specifically, we will:

1. Create an electronic database that will compile all the data from the ECDC database.
2. Develop an online tool to collect any additional data for the participated EU/EEA countries. Those data will be imported into the electronic database.
3. Analyze country specific data and produce a thematic progress report that will include the results of the model for each country, a description of the methodological approach, and the challenges or limitations faced with respect to data and/or modeling.
4. Produce a report that will include estimations from all participated countries.
5. Produce the draft of a scientific manuscript to be submitted to a peer-reviewed journal.
6. Develop an interactive EU map that will contain the HCV estimates from our project.

Methods

Summary database and Data collection procedure

During the first 3 months of the project, a summary database that will include all the data provided by ECDC and an interactive/dynamic data collection tool to gather additional data from countries' representatives/national focal points will be developed. The tool will feed the summary database with all the new estimates that do not previously exist in the database. Data storage will be implemented through a single back-end in a University of Cyprus-based (UCY) server.

The data collection tool will be implemented as a Web front-end, which could be filled in online by the countries' representatives/national focal points. The tool will allow logical skips in the questionnaire and easy extraction of the data. The users will connect to the server of UCY via their regular browsing application (Firefox, Chrome, MS Edge, Safari, Opera, etc.), log into the system by using their provided credentials, and fill-in the questionnaire. Changes to these replies will be possible until a pre-specified deadline.

The user-interface (front-end) of the tool will be organised as single page website in order to allow for simplicity and speed in its use. Subsections of the questionnaire will be clearly marked and questions will be presented progressively and depending on answers to previous questions (logical skips). All forms will be validated and checked for logical errors dynamically.

The use of a database combined with a back-end will minimize errors and inconsistencies. It will also allow the users to track the completeness of their replies and easily spot omitted or incomplete questions. At the same time, it will allow the team running the survey to easily identify countries with large amounts of missing/incomplete information. More importantly, the use of a system built around a database will allow for an easy and reliable extraction of the data. Data extraction from the database could be automated using statistical software packages.

Main Population of interest

After the initiation of blood screening (started in the early to mid-1990s), injection drug use became the key risk factor for new hepatitis C infections in the majority of EU countries. HCV is transmitted in this population predominantly by sharing contaminated needles and other injection equipment. Thus, the main population of interest for this proposal will include the following:

- General population (e.g., population that never injected drugs (non-PWID))

- PWID population that will be divided into current PWID and ex-PWID.

Estimates regarding the prevalence in the general population could be retrieved from national health examination surveys, studies in first-time blood donors or pregnant women. Estimates concerning the PWID could be retrieved from samples from harm reduction services, drug treatment services, and studies of ever injectors based on street-based testing or of ever injectors from prisons.

Evidence Synthesis method

Typically, the prevalence of HCV antibodies has been estimated through single studies including specific risk groups, e.g., national health examination surveys, PWID attending clinics, or individuals donating blood. Such group-specific estimates cannot be used to estimate the population-averaged HCV prevalence unless additional information on the prevalence of each group in the population is known. The traditional way of dealing with this issue is to either assume that some of the single studies are representative of the whole population or to make informal adjustments to the prevalence estimates based on the assumed representativeness of each study [11]. Sensitivity analysis could be applied to assess the impact of such ad hoc adjustments, but the incorporation of uncertainty in the final estimates is not trivial.

Bayesian multiparameter evidence synthesis has been a popular approach to formally estimate HCV prevalence [9, 10, 12] and HIV prevalence [13, 14] by combining multiple sources of data in a systematic manner. Focusing on HCV, each available study is linked to three main risk groups, i.e., current PWID, ex-PWID, and non-PWID. Additional stratification by region, gender, and age is also possible, provided that each population size is known. A unified model is assumed including parameters associated with HCV prevalence of current, ex-PWID, and non-PWID (denoted by π_{cur}, π_{ex}, and π_{non}, respectively) and the prevalence of these risk groups in the population (denoted by ρ_{cur}, ρ_{ex}, and ρ_{non}, respectively). Some studies, though, may not provide data to directly inform some of the previous parameters, e.g., because they include data on a mixture of the risk groups. This does not a priori lead to the exclusion of these studies, as, under certain assumptions, they can indirectly inform the parameters of interest [9]. Thus, Bayesian synthesis takes all available data into account, allowing for an evidence-based estimation of HCV prevalence in the three risk groups. Combining risk-specific estimates with each respective population size produces a population-averaged nationwide estimate, with inherent uncertainty properly accounted for. The simplest case would be to have estimates on the number of current and ex-PWID, along with the HCV prevalence in the current PWID, ex-PWID, and the non-PWID population. In this case, the estimation of the overall HCV prevalence is straightforward. However, data on the prevalence of ex-PWID tend to be

sparse. This issue can be resolved by using the method proposed by Sweeting et al.[9], in which the ex-PWID prevalence, ρ_{ex}, is expressed as a function of the current PWID prevalence, ρ_{cur}. This, however, requires that additional auxiliary data on the distribution of injecting duration (*D*), the time from starting injecting to the year of interest (*TSS*), and the age at first use (*AAFU*) are available for users alive in the year of interest. Similarly, the available data may inform about the HCV prevalence of the current PWID and non-PWID populations, but information on the HCV prevalence of ex-PWID may be missing or unreliable. In these cases, information coming from *TSS*, *D*, and *AAFU*, along with data to estimate the HCV prevalence conditionally on *D* and *TSS* among ever users [$\pi_{ever}(TSS,D)$] suffice to obtain the ex-PWID HCV prevalence. Further combinations of available and missing information might occur, though.

The approach proposed by Sweeting et al. [9] jointly models (i) the probability of being ever PWID ($\rho_{cur} + \rho_{ex}$), (ii) the HCV prevalence of ever PWIDs conditional on *D* and *TSS* [$\pi_{ever}(TSS,D)$], (iii) the HCV prevalence in the non-PWID population π_{non}, and (iv) the distributions of *D*, *TSS*, and *AAFU.* Data associated with the parameters of direct interest, e.g. ρ_{cur}, can be expressed as a complex function of the parameters of the models (i)-(iv) using the formulas provided by Sweeting et al. [9]. Under certain assumptions explicitly stated in Sweeting et al. [9], data sources that estimate some parameter with bias can be incorporated in the model as well.

Bayesian synthesis can then make use of all available prevalence data expressed in the form of a numerator and a denominator for each data source and additional auxiliary data on *TSS*, *D*, and *AAFU*. Prevalence data are assumed to follow independent Binomial distribution given the parameters, independently of the data used to estimate the distributions of *TSS*, *D*, and *AAFU*. A joint prior distribution is attached to the full parameter vector of the model and posterior distributions of the parameters can be obtained using Markov chain Monte Carlo (MCMC) methods, e.g. through WinBUGS [15].

Project tasks and planning

Overview of framework contract and included work packages

This framework contract is divided into four packages. In the following section, the specific tasks and deliverables associated with each work package are presented in detail.

Work Package One (WP1): Development of an electronic database and an online data collection tool. Initially, the database will include all the data provided by ECDC. More specifically, it will contain all the available prevalence surveys within EU/EEA and the results of updated and unpublished information that has been collected from EU/EEA countries through a questionnaire in 2019/2020. Upon completion and in order to collect additional data from the experts of each country, an interactive tool will be developed. The tool will be connected to the database. Emphasis will be made on a user-friendly interface, suitable for non-expert users. Training materials covering all major aspects of the interactive tool will be developed.

Work Package Two (WP2): It will focus on additional data gathering from the participating countries. An email from the project manager that will introduce the project will be sent to all ECDC National Focal Points, asking them to participate in the study. A first virtual meeting with each national focal point will be scheduled to present all the available data we have for the given country and to request any additional data from local non-indexed journals, unpublished data, and any other available data that can be used for the model. We will explain to the experts all major aspects of the interactive tool. Minutes and a list of remaining action items will be sent to the experts. At the end of the first virtual meeting, inputs regarding the country's model will be finalized.

Work Package Three (WP3): It will focus on the analysis of the collected data for each country. Quality and validation controls will be applied to the extracted data. We will calibrate the country's model to obtain the best fit to the observed data. Then, a second virtual meeting will be scheduled with each country's expert to discuss the result of the analysis. If experts do not agree with the results of the model, we will update the model as necessary and the new results will be presented in a new meeting.

Work Package Four (WP4): It involves the preparation of the overall report, of a draft of a scientific manuscript, and a virtual meeting with ECDC. Additional analyses will be performed if required. Except for the scientific manuscript, the results of the study will be disseminated to international conferences.

Finally, to better communicate the results, an interactive EU map that will include all the results of the project will be developed. To optimize the communication of the results of the project, social media pages of the University of Cyprus will be used (Facebook, Twitter).

Outline of specific tasks to be performed under each work package

WP1 - Development of an online data collection tool and the summary database

- Develop the database and the online/interactive tool. Initially, the database will be populated with data that the ECDC already has. Then, it will be enriched by the answers of the expert of each country. The tool will be based on an online database, hosted at a University of Cyprus (UCY) server.
- Support countries which have technical difficulties in reporting through the tool.

WP2 - First vistual meetings and additional data gathering

- Schedule the first virtual meetings with national experts.
- Provide a background on the project, model, and methodology.
- Request data from local non-indexed journals, unpublished data, and any other available data (e.g., hospital-level data) that could be useful.
- Minutes and a list of remaining action items will be sent to the experts.

WP3 - Data analysis

- Extract data and ensure data quality by applying checks for inconsistencies.
- Analyses of data to estimate the national HCV prevalence.
- Prepare a draft report for each country.
- Schedule second virtual meetings with the country's experts to discuss the result of the analyses.
- If experts do not agree with the results of the model, we will update the model as necessary and the new results will be resent.

WP4 - Result Dissemination

- Prepare a draft of scientific manuscript reporting results for all countries.
- Prepare an overall, final report.
- Develop an interactive EU map that will include the output of the study.
- Organize a virtual meeting with ECDC to present: a) the estimates collected up to that time point for each of the 30 EU/EEA countries, b) discuss about any project-related or administrative issues, and 3) discuss about further work to be done under the remaining contract period.

Deliverables

WP1 deliverable

1) **Month 3:** An electronic database compiling all the data collected by ECDC.

WP2 deliverable

1) **Month 7:** Minutes of virtual meetings with each country's experts.

WP3 deliverable

1) **Month 8:** Reports with model's estimations for all EU/EEA countries who agreed to participate. It will include the final model used for each country, the building components of the final model, and challenges or limitations faced with respect to data and/or modelling.
2) **Month 8:** The final version of the database that will contain both ECDC data and additional data from the National Focal Points.
3) **Month 8:** Organization of a virtual meeting with ECDC including its minutes.

WP4 deliverable

1) **Month 9:** A draft manuscript will be prepared compiling the main outputs and challenges in producing the national hepatitis C estimates using the MPES method.
2) **Month 9:** Final report
3) **Month 9:** An interactive EU map that will include all the output of the study.

Table 1. Project’s implementation timeline

**Work Package Number / Title**	**D U R A T IO N (Months)**								
	**1**	**2**	**3**	**4**	**5**	**6**	**7**	**8**	**9**
WP1. Development of an online data collection tool and the summary database									
WP2. Collection of additional data from the experts of each country									
WP3. Data analyses									
WP4. Results dissemination									

Composition and involvement of the project team

Organization involved

With around 7.000 students, 113 laboratories and 830 faculty and staff members, the University of Cyprus (UCY), which employs the project coordinator (Dr Georgios Nikolopoulos), is a young and rapidly expanding university (established in 1989). There are 7 faculties, 22 departments, and 16 research units / centres, covering a broad spectrum within the life, social, natural, and engineering sciences. UCY is considered the leading university and the most active research institution in Cyprus. UCY implemented a large number of research projects funded by the European Commission, the national Research and Innovation Foundation, and several public and private Research Organizations. In 2020, research funding from external sources reached €26 million. UCY is currently participating in 348 projects. Of these, 153 are EU-funded projects under Horizon 2020, Erasmus+, Justice, Life, Cost Action, and Interreg. Within the Horizon 2020 program, UCY ranks 1st in Cyprus with 136 research projects and with a total funding of over €64,4 million from January 2014, the start date of H2020 program, until February 2021. Since the establishment of the European Research Council (ERC) in 2007, UCY managed to win 17 ERC projects. More specifically, it was awarded 6 Starting Grants, 3 Consolidator Grants, 2 Advanced Grants, and 6 Proof of Concept Grants. Currently, UCY also implements 22 Marie Sklodowska-Curie projects. Finally, UCY is also one of the eight young universities within "The Young Universities for the Future of Europe (YUFE)".

The Medical School of the University of Cyprus enrolled its first students in 2013. The University of Cyprus, through an international tender, selected consultants from the Medical School of the University King's College, London, for the certification of the curriculum of the Medical School of UCY for preclinical and clinical years. The Medical School has recently moved into a new facility and collaborates with the major public hospitals in Cyprus. The number of faculty increases every year and is being enriched with both senior and junior faculty who are already producing significant research. The mission of the Medical school of the University of Cyprus is to become a centre of excellence in medical education and research, producing outstanding clinicians, researchers, scholars and teachers, and raise the standards of health care in Cyprus. Moreover, the curriculum of the Medical School emphasizes on epidemiology and public health, and seeks to assist the nation in its efforts to reform its health system and to shape its agenda on health and on health-related issues based on evidence from sound research. The laboratory of Medical Statistics, Epidemiology, and Public Health, leaded by Dr Georgios Nikolopoulos, has been involved in studies on evidence synthesis, environmental epidemiology, HIV epidemiology, and HIV and HCV modelling.

Key personnel

Assistant Professor Georgios Nikolopoulos (Overall project leader)

Georgios Nikolopoulos, DDS, MSc, PhD, FACE, CPH (male): Dr Nikolopoulos is Assistant Professor of Epidemiology and Public Health at the Medical School of the University of Cyprus (UCY). He is a graduate of Dentistry from the Dental School of the National and Kapodistrian University (NKUA) of Athens, Greece and holds a MSc in Biostatistics, and a PhD in Epidemiology (HIV and Hepatitis B virus coinfection) from the Department of Hygiene, Epidemiology and Medical Statistics of the Medical School of the same university. His 18-month post-doctoral fellowship was funded, following international competition, by the International AIDS Society and the United States (US) National Institute on Drug Abuse (NIDA), took place at the National Development and Research Institutes (NDRI) in New Yok, US and in Athens, Greece, and combined training on epidemiology, social networks, and qualitative research. Dr Nikolopoulos is fellow member of the American College of Epidemiology (FACE) and certified in Public Health by the US National Board of Public Health Examiners (CPH). He worked as an expert in epidemiology at the Greek Center for Disease Control and Prevention for nearly 15 years with involvement in the epidemiology and prevention of HIV, and in public health responses against SARS, West Nile Virus infection, and pandemic A (H1N1) influenza. He was site principal investigator of a US-NIDA study on HIV prevention and is currently leading a study in Cyprus on environmental epidemiology that is funded by the Cyprus Research and Innovation Foundation. He has also expertise and long-term experience in synthesis of evidence (systematic reviews, meta-analysis, network meta-analysis). Dr Nikolopoulos has published around 175 peer-reviewed articles, has an h-index of 38, and serves as member of the editorial board, academic editor or topic editor of scientific journals including Pathogens, PLoS ONE, Journal of Clinical Medicine, Data in Brief, Epidemiologia, and the Euro-Mediterranean Journal for Environmental Integration. He served as member of the National Committees in Cyprus for HIV and HCV, and is currently serving as member of the Scientific Committee advising the Minister of Health in the Republic of Cyprus on issues related to the COVID-19 pandemic.

Dr Ilias Gountas

Dr. Ilias Gountas MSc, Ph.D. is working at the Medical school of the University of Cyprus as a postdoctoral Biostatistician Research Associate. He studied applied mathematics at the University of Crete and obtained his MSc in Medical statistics from the Medical school of the University of Athens. His Ph.D. was focused on assessing the future burden of Hepatitis C in Greece and investigating/evaluating potential

strategies targeting the elimination of the disease by 2030. An important part of his Ph.D. was also the economic evaluation of the proposed Hepatitis C elimination strategy in Greece. His doctoral dissertation was supported by a scholarship from the Hellenic Foundation for Research and Innovation (ELIDEK). His modeling studies were the foundation of the Greek Hepatitis C Action Plan. He has participated in important scientific groups, such as the Scientific Committee for the creation of the National Hepatitis C Action Plan in Greece and the Committee for the implementation and monitoring of the National Action Plan for the eradication of viral hepatitis in Greece. His recent work revealed an undetected HCV outbreak among PWID of Athens. He is a member of the statistical team of the Nationwide Health Examination survey EMENO (National Morbidity and Risk Factors Survey – funded by EU and National sources) and the Hprolipsis [a Health Examination Survey of hepatitis B, hepatitis C and HIV in the general and vulnerable populations (migrants and Roma) – funded by EU and National sources]. His post-doc focused on the assessing the future burden of HIV and Hepatitis C in Cyprus and investigating/evaluating potential strategies targeting the elimination of the disease by 2030. His post-doc research was funded by the Onisilos funding scheme of the University of Cyprus. He is the author of 14 refereed articles in top-level hepatology and public health journals (in 8 as first author) and is a frequent speaker on the HCV or HIV subject.

Mr. Christos Thomadakis

Mr. Christos Thomadakis, MSc, PhD candidate is working at the Department of Hygiene and Epidemiology of the National and Kapodistrian University of Athens (NKUA) as a Biostatistician Research Associate. He studied Mathematics at the NKUA and obtained his MSc in Biostatistics from the same university in 2015, with his MSc thesis being on Bayesian methods for clustered survival data. His PhD focuses on methodological issues occurring in joint modeling of longitudinal and survival data, and on Bayesian methods for inference and model comparison, motivated by the HIV epidemiology. His PhD work includes applications of the derived methods to accurately estimate the CD4 cell count evolution before and after treatment initiation under incomplete data due to various missing data mechanisms. He has received two different PhD student conference awards from the International society of Clinical Biostatistics (ISCB) in 2016 and the Eastern Mediterranean Region of the International Biometrics Society (EMR-IBS) in 2017. An important part of his research interests has focused on the development of Bayesian methods to accurately estimate the HIV infection date mainly based on longitudinal CD4 and viral load measurements. As a biostatistician, he has been involved in the CASCADE (Concerted Action on SeroConversion to AIDS and Death in Europe) collaboration, the ECDC (European Centre for Disease Prevention and Control)

funded project “Developing Methods to improve Accuracy of HIV estimates in EU/EAA Countries”, and the East Africa IeDEA (International epidemiology Databases to Evaluate AIDS) collaboration. Starting from 2015, he has been working as a teaching assistant for the “Survival Analysis” and “Clinical Trials” courses in the Postgraduate Program of Studies in Biostatistics at the NKUA. He has authored 9 (in 3 being the lead author) peer-reviewed papers in international journals.

Dr Kostantinos Gountas

Dr Kostantinos Gkountas, MSc, PhD electrical and computer engineer, has a great experience in developing code for system modeling, analysis, control and visualization. He holds a PhD degree in the Electrical and Computer Engineering Department of the University of Patras, funded by the Greek State Scholarships Foundation. He had participated in the Horizon2020 program called AEROWORKS, ("Collaborative Aerial Robotic Workers" - http://www.aeroworks2020.eu/) where he contributed to the modeling of a UAV carrying a robotic manipulator. He had participated in "EDBM34" research program: "Design and implementation of a complementary sensing system for physiotherapy procedure monitoring through smart portable devices", co-funded by the European Regional Development Fund (ERDF 2014-2020) of the European Union and by National Resources, where he contributed in developing of a motion sensor for upper limb rehabilitation and in visualization of the obtained results. He has been collaborating with the medical school of the University of Athens since 2014 for which he has created / updated many databases and web-based applications. He has created the interactive interface of the Athens-Multicenter-AIDS-Cohort-Study database and is responsible for all the needed updates.

Distribution of tasks

Dr Georgios Nikolopoulos will be the overall leader of the project and will oversee the progress of the project against the work plan, identify risks, and propose appropriate corrections if necessary. WP1 will be led by Dr Konstantinos Gountas with the support of Dr Ilias Gountas and Dr Georgios Nikolopoulos. WP2 will be led by Dr Georgios Nikolopoulos with the support of Ilias Gountas. WP3 will be led by Mr. Thomadakis Christos and Dr Ilias Gountas with the support of Dr Georgios Nikolopoulos. WP4 will be led by Dr Ilias Gountas with the support of Dr Georgios Nikolopoulos.

References

1. European Union, H.C.V.C., *Hepatitis C virus prevalence and level of intervention required to achieve the WHO targets for elimination in the European Union by 2030: a modelling study.* Lancet Gastroenterol Hepatol, 2017. **2**(5): p. 325-336.
2. Lozano, R., et al., *Global and regional mortality from 235 causes of death for 20 age groups in 1990 and 2010: a systematic analysis for the Global Burden of Disease Study 2010.* Lancet, 2012. **380**(9859): p. 2095-128.
3. Bosetti, C., et al., *Trends in mortality from hepatocellular carcinoma in Europe, 1980-2004.* Hepatology, 2008. **48**(1): p. 137-45.
4. Deuffic-Burban, S., et al., *Predicted effects of treatment for HCV infection vary among European countries.* Gastroenterology, 2012. **143**(4): p. 974-85 e14.
5. WHO. *Global Health Sector Strategy on viral hepatitis, 2016–2021*. 2015 [cited 2015 28/10]; Available from: http://www.who.int/hiv/draft-hep-strategy-2016-2021_en.pdf.
6. Thein, H.H., et al., *Estimation of stage-specific fibrosis progression rates in chronic hepatitis C virus infection: a meta-analysis and meta-regression.* Hepatology, 2008. **48**(2): p. 418-31.
7. Davis, G.L., et al., *Aging of hepatitis C virus (HCV)-infected persons in the United States: a multiple cohort model of HCV prevalence and disease progression.* Gastroenterology, 2010. **138**(2): p. 513-21, 521 e1-6.
8. Gamkrelidze, I., et al., *Progress towards hepatitis C virus elimination in high-income countries: an updated analysis.* Liver Int, 2021.
9. Sweeting, M.J., et al., *Estimating hepatitis C prevalence in England and Wales by synthesizing evidence from multiple data sources. Assessing data conflict and model fit.* Biostatistics, 2008. **9**(4): p. 715-34.
10. McDonald, S.A., et al., *Bridging the data gaps in the epidemiology of hepatitis C virus infection in Malaysia using multi-parameter evidence synthesis.* BMC Infect Dis, 2014. **14**: p. 564.
11. Department of Health. *Hepatitis C Strategy for England*. 2002; Available from: http://www.dh.gov.uk/en/Publicationsandstatistics/Publications/PublicationsPolicyAndGuidance/Browsable/DH−4103274.
12. Tan, S., et al., *A Bayesian evidence synthesis approach to estimate disease prevalence in hard-to-reach populations: hepatitis C in New York City.* Epidemics, 2018. **23**: p. 96-109.
13. Presanis, A.M., et al., *Insights into the rise in HIV infections, 2001 to 2008: a Bayesian synthesis of prevalence evidence.* AIDS, 2010. **24**(18): p. 2849-58.
14. van Veen, M.G., et al., *National estimate of HIV prevalence in the Netherlands: comparison and applicability of different estimation tools.* AIDS, 2011. **25**(2): p. 229-37.
15. D. J. Spiegelhalter, A. Thomas, N. G. Best and D. Lunn, *WinBUGS User's Manual*, in *Cambridge*, MRC. Biostatistics Unit, Editor. 2003.
